# The conserved transcription factor PrlP modulates colonization and pathogenicity of *Streptococcus suis* in response to environmental stress

**DOI:** 10.1371/journal.ppat.1013314

**Published:** 2025-07-18

**Authors:** Yuhua Wang, Ran Liu, Weihua Lv, Kunlong Xia, Xi Lu, Xinyu Gao, Shulin Miao, Anding Zhang

**Affiliations:** 1 National Key Laboratory of Agricultural Microbiology, Hubei Hongshan Laboratory, College of Veterinary Medicine, Huazhong Agricultural University, Wuhan, Hubei, China; 2 Key Laboratory of Preventive Veterinary Medicine in Hubei Province, The Cooperative Innovation Center for Sustainable Pig Production, Wuhan, Hubei, China; 3 Key Laboratory of Development of Veterinary Diagnostic Products, Ministry of Agriculture of the People’s Republic of China, Wuhan, Hubei, China; 4 International Research Center for Animal Disease, Ministry of Science and Technology of the People’s Republic of China, Wuhan, Hubei, China; 5 Guangdong Provincial Key Laboratory of Research on the Technology of Pig-breeding and Pig-disease prevention, Guangzhou, Guangdong, China; Lunds universitet Medicinska fakulteten, SWEDEN

## Abstract

Opportunistic pathogens can cause infections when host defenses are compromised. Among them, *Streptococcus suis* (*S. suis*) colonizes the upper respiratory tract of pigs and causes severe diseases in both swine and humans. Although the pathogenic mechanisms of these bacteria have been partially elucidated, the molecular processes that govern their adaptation, colonization, and pathogenesis remain incompletely understood. In this study, we identified PrlP as a transcriptional repressor in *S. suis* that responds to mildly acidic, oxidative, hyperosmotic, and thermal stresses, and regulates bacterial growth, chain morphology, nasal colonization, and virulence. The C-terminal S24 peptidase domain of PrlP mediates stress-induced self-cleavage to control protein stability, while the N-terminal helix-turn-helix (HTH) DNA-binding domain is essential for its transcriptional regulatory function. Combined ChIP-seq and RNA-seq analyses revealed its binding motif (5′-CCTGAAWCT-3′) and identified *B9H01_08740* as a direct target gene, as further validated by EMSA. Notably, deletion of *B9H01_08740* in the *prlP*-deficient background restored the associated phenotypes. These findings highlight PrlP as a key regulator in *S. suis* that maintains cellular homeostasis in response to stress conditions and modulates target genes such as *B9H01_08740* to promote nasal colonization and virulence. Therefore, this study provides new insights into the regulatory mechanisms of pathogenic bacteria and may aid in the development of targeted strategies against *S. suis* infections.

## Author summary

*Streptococcus suis* (*S. suis*) is a zoonotic pathogen that colonizes the upper respiratory tract of pigs and causes severe diseases in both swine and humans. To persist in this dynamic niche, *S. suis* must withstand diverse environmental challenges. However, the mechanisms by which the bacterium senses and responds to these conditions remain poorly defined. In this study, we identify PrlP, a transcriptional repressor, as a stress-responsive regulator that undergoes self-cleavage to modulate downstream gene expression. We show that PrlP directly regulates *B9H01_08740*, a previously uncharacterized gene essential for stress resistance and colonization. Disruption of this pathway alters chain morphology, reduces stress tolerance, and attenuates virulence in both mouse and *Galleria mellonella* infection models. Transcriptomic and ChIP-seq analyses further reveal that PrlP coordinates a broader regulatory network beyond *B9H01_08740*, highlighting its role in environmental adaptation. Collectively, our findings uncover a multifunctional role for PrlP and suggest its potential as a target for anti-colonization strategies against *S. suis*.

## Introduction

*Streptococcus suis* (*S. suis*) is a major bacterial pathogen in the pig farming industry and an emerging zoonotic agent of global concern [1–3]. In humans, *S. suis* primarily causes meningitis, arthritis, pneumonia, and septicemia, often accompanied by long-term sequelae such as hearing loss [4]. To date, more than 2,000 human cases have been reported worldwide, with mortality rates exceeding 10% in the absence of timely treatment [5,6]. It is the leading cause of adult meningitis in Vietnam [7], ranks second in Thailand [2,8], and fifth in China [9]. Importantly, the nasal and tonsillar cavities of healthy pigs serve as the primary reservoirs for both swine infections and zoonotic transmission [10]. Thus, understanding how *S. suis* adapts, persists, and invades from the upper respiratory tract is crucial for elucidating its pathogenic mechanisms and developing effective control strategies.

The ability of pathogenic microorganisms to colonize host tissues depends on their capacity to precisely sense and dynamically respond to multiple stress conditions. In the upper respiratory tract, *S. suis* must first adapt to the mildly acidic conditions of the nasal mucosa (pH 5.0–6.5) [11], and cope with oxidative stress caused by reactive oxygen species (ROS) released by immune cells [12,13], as well as inflammation-induced osmotic fluctuations and temperature elevation resulting from fever or overcrowding after host invasion [14,15]. These combined stressors create a complex landscape that *S. suis* must overcome during the transition from colonization to infection. Although several virulence factors involved in *S. suis* infection have been systematically identified—such as those contributing to adherence and invasion [16–19], immune evasion, penetration of the blood–brain barrier [20–22], and the induction of cytokine storms leading to high mortality [23–25], the regulatory networks that enable early adaptation and persistent colonization in the nasal cavity, and ultimately lead to systemic infection, remain poorly understood.

To investigate how *S. suis* adapts to acidic and other host-associated stress conditions in the upper respiratory tract, we performed a transposon mutant library screen and identified *B9H01_05155*, an XRE-family transcriptional repressor hereafter referred to as PrlP. Disruption of prlP resulted in pronounced growth defects under acidic conditions. Functional analyses revealed that PrlP undergoes self-cleavage under normal conditions, and this process is significantly inhibited under stress conditions such as acidity. This stress-inhibited cleavage modulates PrlP’s ability to regulate downstream gene expression, including that of *B9H01_08740*. Notably, PrlP is highly conserved across diverse *S. suis* strains, suggesting a shared regulatory mechanism for balancing colonization and virulence. Collectively, our findings uncover a novel regulatory strategy by which *S. suis* senses and responds to host environmental stresses, and establish PrlP as a potential target for therapeutic intervention.

## Results

### Screening of the essential genes of *S. suis* in response to micro acidic conditions of the upper respiratory tract

Colonization of the upper respiratory tract by *S. suis* serves as the primary source of infection in both pigs and humans. However, successful colonization requires adaptation to adverse conditions, including a mildly acidic environment (pH 5.0–6.5) [11]. Therefore, identifying *S. suis* genes that mediate responses to mildly acidic and other environmental stressors is essential for elucidating its colonization strategies in the upper respiratory tract.

**Fig 1:**
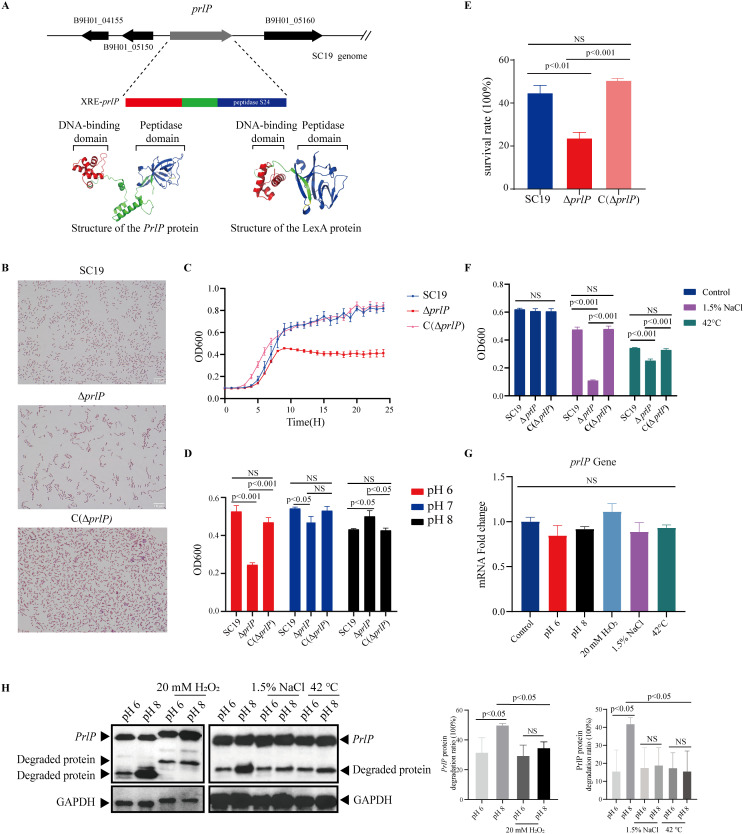
Identification and functional characterization of *prlP*, a key gene involved in *S. suis* growth and survival under environmental stress conditions. (A) Genomic organization and predicted domain structure of PrlP in *S. suis* SC19. The PrlP protein contains an N-terminal helix-turn-helix (HTH) DNA-binding domain and a C-terminal S24 peptidase domain. Structural modeling indicates that PrlP shares conserved features with the LexA-like protease family. (B) Microscopic analysis of SC19, Δ*prlP*, and C(Δ*prlP*) strains. Cells were collected at mid-log phase (OD600 ≈ 0.6), Gram-stained, and visualized under a light microscope. Scale bar = 10 μm. (C) Growth curves of the indicated strains in TSB medium. Overnight cultures were diluted 1:100 and incubated at 37°C with shaking. OD600 was measured hourly over 24 hours. (D) Growth of SC19, Δ*prlP*, and C(Δ*prlP*) under mildly acidic (pH 6.0), neutral (pH 7.0), and alkaline (pH 8.0) conditions. OD600 was measured after 8 hours of incubation at 37°C. (E) Survival of the indicated strains following oxidative stress. Cultures were grown for 8 hours, treated with 20 mM H₂O₂ for 1 hour, and survival was quantified. (F) Growth responses under osmotic (1.5% NaCl) and thermal (42°C) stress. OD600 values were measured after 8 hours of incubation. (G) RT-qPCR analysis of *prlP* expression under stress conditions. SC19 cultures were exposed to pH 6.0, pH 8.0, 20 mM H₂O₂, 1.5% NaCl, or 42°C for 1 hour. No significant changes in *prlP* transcript levels were observed. “Control” indicates untreated TSB medium. (H) Western blot analysis of PrlP protein stability under the same stress conditions. A strain expressing PrlP-Flag was used. Full-length and degraded forms of PrlP were detected; GAPDH served as the loading control. PrlP degradation was quantified by densitometric analysis of Western blot bands, calculated as the ratio of cleaved PrlP to the total PrlP signal. Data represent means ± SD from three independent experiments. Statistical significance was determined using unpaired two-tailed *t*-tests; NS, not significant; *p* < 0.05, significant.

To identify genes essential for acid stress adaptation, we screened a previously constructed *S. suis* SC19 transposon mutant library comprising 1,665 strains [26]. Each mutant was cultured under pH 6.0 and pH 7.0 conditions, and growth was evaluated based on the OD600 ratio (pH 6.0/ pH 7.0). Several mutants exhibited impaired growth under acidic conditions, among which strain No. 53 consistently showed the most pronounced and reproducible growth defect across independent experiments. Sequencing revealed that the transposon in No. 53 had inserted into a gene encoding an uncharacterized XRE-family transcriptional regulator. We designated this gene as *prlP* (S1 Fig in S1 File). Structural prediction suggested that PrlP adopts a conformation similar to members of the LexA-like protein family (Fig 1A). To validate the role of *prlP*, an isogenic deletion mutant (Δ*prlP*) and its complemented strain C(Δ*prlP*) were constructed (S2 Fig in S1 File). Compared with the wild-type (WT) strain, Δ*prlP* exhibited extensive chain elongation and reduced cell density during logarithmic growth; this phenotype was restored by complementation (Fig 1B and 1C). Given that the pH of late-log-phase culture drops to approximately 6.0, the observed growth defect may be associated with increased acid sensitivity. To eliminate pH-related artifacts, further experiments were performed in a more strongly buffered medium. Under neutral pH (7.0), no significant differences in bacterial counts were observed among WT, Δ*prlP*, and C(Δ*prlP*) strains (Fig 1D). In contrast, Δ*prlP* displayed enhanced growth at pH 8.0 and impaired growth at pH 6.0 relative to WT (Fig 1D), indicating that PrlP plays a critical role in the response of *S. suis* to mildly acidic environments within the upper respiratory tract.

In addition to acidic stress in the nasal mucosa, *S. suis* faces oxidative, hyperosmotic, and thermal stresses caused by immune responses and inflammation, which pose significant challenges to colonization and infection. To determine whether PrlP contributes to adaptation beyond acidic stress, we assessed the phenotypes of WT, Δ*prlP*, and complemented C(Δ*prlP*) strains under oxidative (20 mM H₂O₂), hyperosmotic (1.5% NaCl), and thermal (42°C) stress. Under each condition, the Δ*prlP* mutant exhibited significantly impaired growth or survival compared to the WT strain, while complementation restored the wild-type phenotype (Fig 1E and 1F). In addition, *prlP* is also required for the adaptation to stress conditions for another *S. suis* strain, such as the P1/7 strain (S3 Fig in S1 File). These results suggest that PrlP is essential for *S. suis* to tolerate diverse stress conditions commonly encountered in the host environment. Given its ubiquitous presence and high conservation among *S. suis* strains (S1 Table), PrlP-mediated stress adaptation may serve as a core mechanism for successful colonization and persistence in the upper respiratory tract and other hostile niches.

### PrlP responds to stress conditions through self-cleavage

PrlP encodes a 266-amino-acid protein containing two conserved domains characteristic of the LexA-like transcriptional repressor superfamily: an N-terminal helix-turn-helix (HTH) DNA-binding domain, classified as an inhibitory factor, and a C-terminal S24 peptidase domain (Fig 1A) [27]. Notably, LexA-like S24 peptidases possess the ability to catalyze self-cleavage, and the resulting N-terminal fragments are subsequently degraded by other proteases [28–31].

Interestingly, *prlP* transcript levels remained largely unchanged across all tested stress conditions (Fig 1G), suggesting that its function is likely regulated at the post-translational level. To further assess whether PrlP undergoes self-cleavage in response to conditions of stress, we expressed a C-terminal 3 × Flag-tagged PrlP protein in the Δ*prlP* background. Under mildly alkaline conditions (pH 8.0), Western blot analysis revealed a marked reduction in full-length PrlP and the appearance of an ~ 20 kDa cleavage product, corresponding to the predicted N-terminal fragment (Fig 1H). PrlP cleavage was strongly suppressed at pH 6.0 (Fig 1H). Oxidative, osmotic, and heat stress also reduced cleavage at pH 8.0, suggesting PrlP responds to multiple stress signals (Fig 1H). However, no additional inhibition was observed under pH 6.0 (Fig 1H), implying that this response may have a threshold or be condition-dependent. These results suggest that inhibition of PrlP cleavage under stress conditions facilitates *S. suis* adaptation.

### PrlP contributes to the virulence of *S. suis*

**Fig 2:**
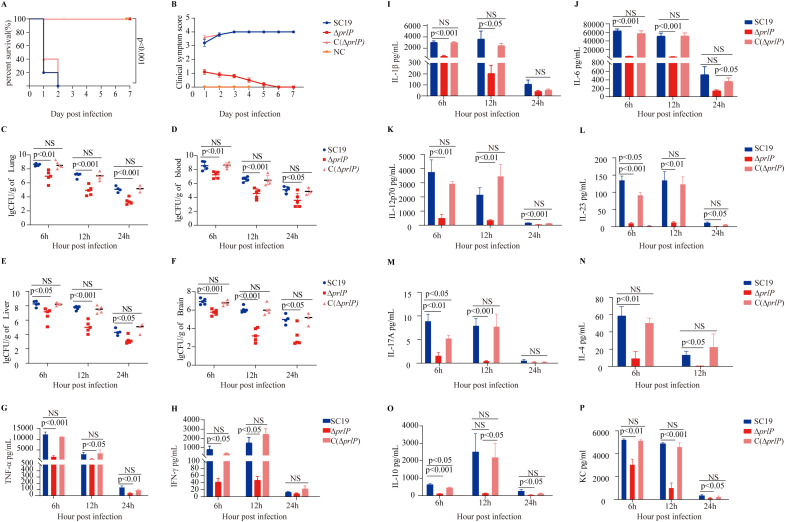
Attenuated virulence of the Δ*prlP* strain in a BALB/c mouse infection model. (A) Survival curves of mice intraperitoneally infected with WT, Δ*prlP*, or C(Δ*prlP*) strains at a dose of 1 × 10⁸ CFU/mouse (log-rank test, *n* = 10). (B) Clinical symptom scores of infected mice assessed over time (two-way repeated measures ANOVA, *n* = 10). (C–F) Bacterial burdens in blood (C), liver (D), lung (E), and brain (F) at 6, 12, and 24 hours post-infection, determined following intraperitoneal injection with 1 × 10⁸ CFU of the indicated strains (unpaired two-tailed *t*-tests, *n* = 5). (G–P) Serum cytokine concentrations were measured at 6, 12, and 24 hours post-infection using ELISA kits (unpaired two-tailed *t*-tests, *n* = 5). Error bars represent mean ± standard deviation (SD). NS, not significant; *p* < 0.05, significant.

Given the role of PrlP in facilitating *S. suis* adaptation to diverse environmental stressors [32,33], we hypothesized that it may also contribute to bacterial colonization and virulence. In a mouse infection model, the WT strain induced severe clinical symptoms and high mortality (Fig 2A and 2B), accompanied by substantial bacterial burdens in multiple tissues (Fig 2C–2F) and elevated levels of proinflammatory cytokines (Fig 2G–2P). In contrast, mice infected with the Δ*prlP* mutant exhibited neither excessive cytokine responses (Fig 2G–2P) nor significant tissue colonization (Fig 2C–2F), and no mortality was observed during the trial (Fig 2A and 2B). Notably, the complementation strain C(Δ*prlP*) fully restored the virulent phenotype, including mortality, bacterial load, and cytokine induction (Fig 2A–2P). Collectively, these results demonstrate that PrlP plays a critical role in the pathogenesis of *S. suis.*

### The N-terminal HTH domain is critical for PrlP-mediated regulation and virulence in *S. suis*

**Fig 3:**
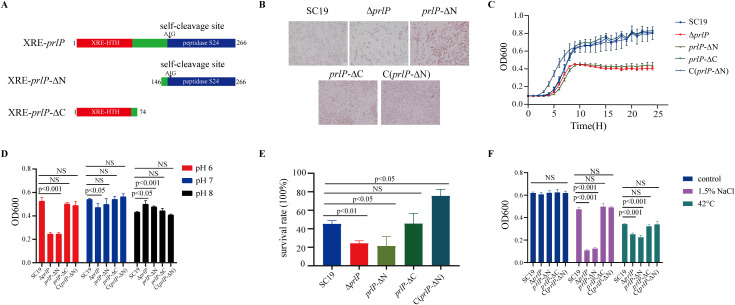
Functional dissection of PrlP domains reveals the essential role of the N-terminal HTH domain in growth, stress tolerance, and survival of *S. suis.* (A) Schematic representation of domain structures in wild-type PrlP (1–266 aa), N-terminal deletion mutant *prlP*-ΔN (146–266 aa), and C-terminal deletion mutant *prlP*-ΔC (1–145 aa). The predicted self-cleavage site (Ala–Gly) is indicated. (B) Cell morphology of SC19, Δ*prlP*, *prlP*-ΔN, *prlP*-ΔC, and the complemented strain C(*prlP*-ΔN), assessed by Gram staining. (C) Growth curves of the indicated strains cultured in TSB at 37°C with shaking. OD600 was recorded hourly over 24 hours. (D) Growth under pH 6.0 (acidic), pH 7.0 (neutral), and pH 8.0 (alkaline) conditions. Strains were inoculated into pH-adjusted TSB and cultured for 8 hours. (E) Survival rates following oxidative stress. Cultures were treated with 20 mM H₂O₂ at 37°C for 1 hour. (F) Growth performance under hyperosmotic (1.5% NaCl) and thermal (42°C) stress for 8 hours. All data represent means ± SD from three independent experiments. Statistical significance was determined by unpaired two-tailed *t*-tests. NS, not significant; *p* < 0.05, significant.

To determine whether the regulatory and virulence functions of PrlP are mediated by its N-terminal HTH domain or C-terminal S24 peptidase domain, we constructed two domain-deletion mutants. The N-terminal HTH domain (amino acids 1–74) was deleted to generate the *prlP*-ΔN mutant (Figs 3A and S4 in S1 File). Given that PrlP self-cleaves at Ala155–Gly156 within the S24 peptidase domain, generating an Ala-COOH terminus that may trigger further degradation of the N-terminal fragment by ClpXP and ClpCP proteases [31], we generated a C-terminal truncation mutant retaining only amino acids 1–145 (*prlP*-ΔC) to prevent such degradation (Figs 3A and S4 in S1 File).

The *prlP*-ΔN mutant exhibited pronounced chain elongation and recapitulated the Δ*prlP* phenotype under multiple stress conditions (Fig 3B–3F). Complementation with the N-terminal domain C(*prlP*-ΔN) fully restored WT characteristics (Fig 3B–3F). In contrast, the *prlP*-ΔC mutant showed growth and stress responses similar to the WT under all tested conditions (Fig 3B–3F). These findings indicate that the N-terminal HTH DNA-binding domain, rather than the C-terminal peptidase domain, is essential for PrlP’s regulatory role in growth, stress tolerance.

**Fig 4:**
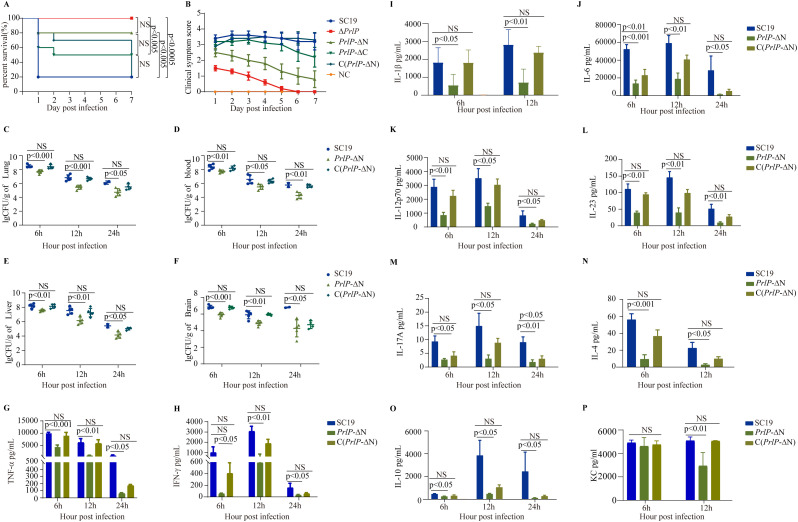
The N-terminal HTH domain of PrlP is required for *S. suis* virulence in a BALB/c mouse infection model. (A) Survival curves of mice intraperitoneally infected with WT, Δ*prlP*, *prlP*-ΔN, *prlP*-ΔC, or C(*prlP*-ΔN) strains (1 × 10⁸ CFU/mouse; log-rank test, *n* = 10). (B) Clinical symptom scores of infected mice (two-way repeated measures ANOVA, *n* = 10). (C–F) Bacterial burdens in blood (C), liver (D), lung (E), and brain (F) at 6, 12, and 24 hours post-infection following inoculation with 1 × 10⁸ CFU of the indicated strains (unpaired two-tailed *t*-tests, *n* = 5). (G–P) Cytokine levels in peritoneal lavage fluids at 6, 12, and 24 hours post-infection, measured using ELISA (unpaired two-tailed *t*-tests, *n* = 5). Error bars represent mean ± SD. NS, not significant; *p* < 0.05, significant.

Given the critical role of PrlP in *S. suis* virulence, we further assessed the individual contributions of its N-terminal HTH domain and C-terminal S24 peptidase domain to pathogenesis in a mouse infection model. The *prlP*-ΔC mutant, which retains the full N-terminal domain, induced severe clinical symptoms and high mortality, and an absence of cytokine storm (Fig 4G–4P), comparable to the WT strain (Fig 4A and 4B). In contrast, mice infected with the *prlP*-ΔN mutant exhibited no mortality, markedly reduced bacterial burdens in the blood, liver, lungs, and brain (Fig 4C–4F), and an absence of cytokine storm (Fig 4G–4P), phenocopying the Δ*prlP* strain. Notably, virulence was fully restored in the complemented strain C(*prlP*-ΔN), as evidenced by increased clinical scores, mortality, bacterial loads, and cytokine levels (Fig 4A–4P). These findings collectively demonstrate that the N-terminal HTH DNA-binding domain is indispensable for *S. suis* growth, stress tolerance, and virulence.

### Autocleavage of PrlP coordinates stress adaptation and virulence in *S. suis*

**Fig 5:**
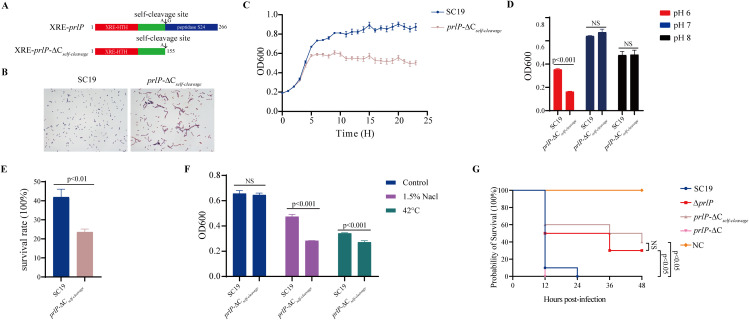
Autocleavage of PrlP coordinates stress adaptation and virulence in *S. suis.* (A) Schematic representation of PrlP domain architecture and cleavage-related mutants. The *prlP-*ΔC_*self-cleavage*_ mutant lacks amino acids 156–266, mimicking the cleaved N-terminal form generated by self-cleavage at Ala155–Gly156. (B) Gram staining of SC19, *prlP-*ΔC_*self-cleavage*_, and *prlP-*ΔC. The *prlP-*ΔC_*self-cleavage*_ mutant exhibits elongated chain morphology, similar to that observed in Δ*prlP* and *prlP-*ΔN strains. (C) Growth curves in TSB at 37°C. The *prlP-*ΔC_*self-cleavage*_ strain displays impaired growth compared to SC19. (D) Growth under acidic (pH 6.0), neutral (pH 7.0), and alkaline (pH 8.0) conditions. The *prlP-*ΔC_*self-cleavage*_ mutant exhibits significantly reduced growth at pH 6.0. (E) Survival following oxidative stress (20 mM H₂O₂ for 1 hour). The *prlP-*ΔC_*self-cleavage*_ mutant shows a marked reduction in survival relative to the wild type. (F) Growth under hyperosmotic (1.5% NaCl) and thermal (42°C) stress. The *prlP-*ΔC_*self-cleavage*_ mutant is highly sensitive to both conditions. (G) Virulence assessment using the *Galleria mellonella* infection model. Larvae (n = 10 per group) were injected into the left posterior proleg using a Hamilton syringe with 2.5 × 10⁶ CFUs of SC19, Δ*prlP*, *prlP*-ΔC_*self-cleavage*_, *prlP*-ΔC, or PBS (negative control, NC). The *prlP-*ΔC_*self-cleavage*_ mutant exhibited significantly attenuated virulence compared to SC19. Larval survival was monitored for 48 hours post-infection, and statistical differences were determined using the log-rank test. Statistical analysis: Data in (C–F) represent means ± SD from three independent experiments and were analyzed by unpaired two-tailed t-tests. Survival curves in (G) were analyzed using the log-rank (Mantel–Cox) test. P < 0.05 was considered statistically significant.

The N-terminal region of PrlP is essential for its regulatory function, in response to stress conditions, based on the *prlP*-ΔC mutant in which the N-terminus cannot be degraded (Figs 3 and 4). Notably, LexA-like proteins undergo self-cleavage between adjacent alanine and glycine residues, which often leads to further degradation of the resulting fragments [31]. To investigate the effect of the N-terminal fragment of PrlP produced by autocleavage, we constructed another C-terminal truncation mutant (*prlP*-ΔC_*self-cleavage*_) by deleting residues 156–266 downstream of the glycine cleavage site (Figs 5A and S5 in S1 File). The mutant strain showed severe growth impairment, abnormal chain elongation, and substantial reductions in both stress resistance and virulence (Fig 5B–5G). These findings suggested that the N-terminal fragment produced by autocleavage may be further degraded, leading to a loss of its regulatory function. This result demonstrated that the dynamic auto-cleavage catalyzed by the C-terminal peptidase domain modulates *S. suis* growth, stress adaptation, and virulence.

### Identification of PrlP regulon by RNA-seq

**Fig 6:**
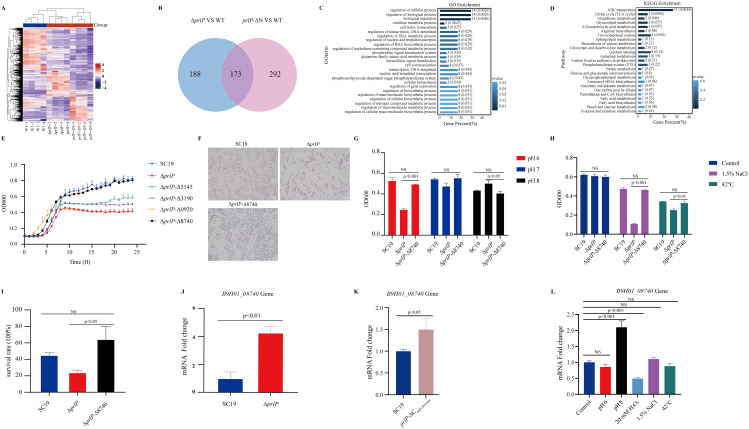
PrlP regulates a broad transcriptional network in *S. suis* and activates expression of *B9H01_08740* through self-cleavage. (A) Heatmap showing transcriptomic profiles of WT, Δ*prlP*, and *prlP*-ΔN strains based on RNA-seq analysis. Firebrick and navy represent upregulated and downregulated genes, respectively. (B) Venn diagram displaying overlapping DEGs between Δ*prlP* vs. WT and *prlP*-ΔN vs. WT. (C) GO enrichment analysis of 173 DEGs, showing functional categories related to metabolism and biosynthesis (generated via www.omicshare.com/tools). (D) KEGG pathway enrichment analysis of the same DEG set, revealing enrichment in ABC transporter, TCA cycle, and amino acid biosynthesis pathways (generated via www.omicshare.com/tools). (E) Growth curves of WT, Δ*prlP*, Δ*prlP*-Δ5145, Δ*prlP*-Δ3190, Δ*prlP*-Δ0920, and Δ*prlP*-Δ8740 strains in TSB at 37°C. (F) Gram staining analysis of cell morphology across the indicated strains. (G) Growth densities of WT, Δ*prlP*, and Δ*prlP*-Δ8740 strains under pH 6.0, 7.0, and 8.0 (unpaired two-tailed *t*-tests, *n* = 3). (H) Growth performance under hyperosmotic (1.5% NaCl) and thermal (42°C) stress conditions. (I) Survival rates of WT, Δ*prlP*, and Δ*prlP*-Δ8740 strains following treatment with 20 mM H₂O₂ for 1 hour at 37°C (*n* = 3). (J) RT-qPCR analysis showing upregulation of *B9H01_08740* in the Δ*prlP* strain relative to WT. (K) RT-qPCR analysis of *B9H01_08740* in *prlP-*ΔC_*self-cleavage*_ strains. Expression was significantly upregulated in *prlP-*ΔC_*self-cleavage*,_ indicating regulation by PrlP self-cleavage activity. (L) Expression of *B9H01_08740* under multiple stress conditions (pH 6.0, pH 8.0, 20 mM H₂O₂, 1.5% NaCl, and 42°C). Alkaline pH (pH 8.0) strongly induced expression, while hydrogen peroxide and heat stress markedly suppressed it. Mid-log phase cells in TSB were used for all stress treatments; “Control” indicates untreated TSB medium. All data represent mean ±SD from three independent experiments. Statistical significance was determined using two-tailed unpaired *t*-tests. NS, not significant; *p* < 0.05, significant.

To further elucidate how PrlP regulates gene expression in response to environmental stress and contributes to virulence, we compared the transcriptomes of WT, Δ*prlP*, and *prlP*-ΔN strains using RNA-seq (Fig 6A). Compared with the WT strain, both Δ*prlP* and *prlP*-ΔN exhibited significant differential expression of 173 genes (fold change ≥ 2.0; adjusted *p* < 0.05), including 76 upregulated and 97 downregulated genes (Fig 6B and S2 Table). These differentially expressed genes (DEGs) were enriched in Gene Ontology (GO) categories related to metabolic and biosynthetic processes (Fig 6C). Kyoto Encyclopedia of Genes and Genomes (KEGG) pathway analysis further revealed significant enrichment in ABC transporters, the citrate cycle, and amino acid biosynthesis pathways (Fig 6D), suggesting that PrlP modulates a broad regulatory network involved in diverse physiological functions in *S. suis*.

Among the 173 DEGs, several genes are associated with cell growth and morphology (e.g., *mreD*, *B9H01_00195*) [34], stress responses (e.g., *ciaR*, *rex*, *argR*) [35–38], and virulence-related functions (e.g., Zn-dependent protease, *SspA-1*, *MstR*) [39–41], as previously reported in other streptococcal species. These findings suggest that PrlP may exert broad regulatory effects on cellular physiology and pathogenicity by modulating genes involved in these functional categories.

### The *B9H01_08740* gene, regulated by PrlP, is essential for stress resistance in *S. suis*

To identify key downstream effectors regulated by PrlP, we initially targeted the top six significantly upregulated DEGs identified from the RNA-seq analysis of the Δ*prlP* mutant for deletion in the same genetic background. Among these, four genes—*B9H01_05145, B9H01_03190, B9H01_08740, or B9H01_00920*—were successfully knocked out based on the Δ*prlP* strain (S6 Fig in S1 File). Interestingly, Δ*prlP*-Δ8740 exhibited markedly improved growth during the logarithmic phase, normalized chain morphology, and enhanced resistance to mildly acidic and other host-relevant stress conditions (Fig 6E–6I). Furthermore, RT-qPCR confirmed that *B9H01_08740* was significantly upregulated in Δ*prlP* and autocleavage-mimicking mutant (*prlP-*ΔC_*self-cleavage*_), indicating transcriptional repression by PrlP (Fig 6J and 6K). Notably, its expression was suppressed under conditions that inhibit PrlP cleavage (Fig 6L). Together, these findings demonstrate that PrlP self-cleavage is necessary for the activation of *B9H01_08740*, which plays a critical role in *S. suis* stress adaptation.

### Identification of PrlP binding motif in the *S. suis* genome

**Fig 7:**
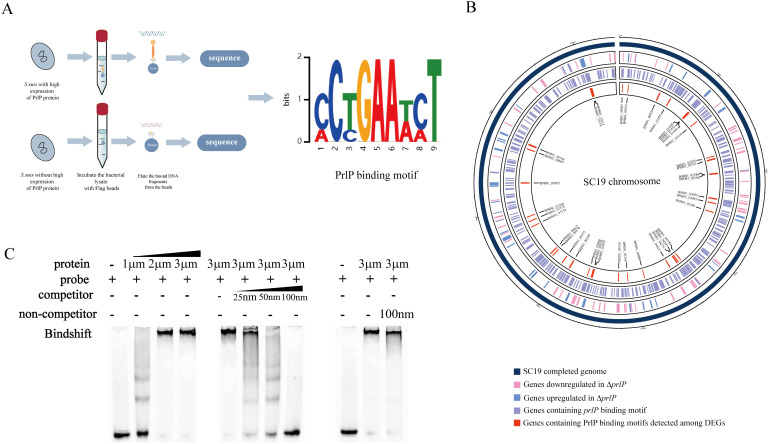
PrlP Binding motif and direct regulation of *B9H01_08740.* (A) ChIP-seq analysis of PrlP binding sites across the *S. suis* genome. PrlP-3 × Flag was expressed in the Δ*prlP* strain, and DNA fragments bound to PrlP were immunoprecipitated using anti-Flag magnetic beads and subjected to high-throughput sequencing. A conserved 9-bp binding motif (5′-CCTGAAWCT-3 ′) was identified using MEME motif analysis. (B) Circos plot integrating ChIP-seq and RNA-seq results. Outer to inner rings: SC19 genome (dark blue), genes downregulated in Δ*prlP* (pink), genes upregulated in Δ*prlP* (blue), genes containing the PrlP-binding motif (purple), and genes both differentially expressed and containing the motif (red), representing putative direct targets of PrlP. (C) EMSA confirming direct binding of PrlP to the upstream region of *B9H01_08740*. Biotin-labeled probe (FAM′-TCGGCAGAATTAGTTTCCGGTGAAGTTTGA) was incubated with purified PrlP-His₈ fusion protein. Shifted bands indicate formation of PrlP–DNA complexes, which were competed by unlabeled specific probe but not by nonspecific control. All figure elements were created by the authors using Adobe Illustrator 2024, without the inclusion of any third-party copyrighted material. This figure is fully compliant with the CC BY 4.0 license.

To identify the unknown binding motif of PrlP, chromatin immunoprecipitation sequencing (ChIP-seq) was performed using α-Flag beads in a mutant strain in which C-terminal 3 × Flag-tagged PrlP was introduced into the Δ*prlP* strain (Fig 7A). A conserved 9-bp binding motif (5′-CCTGAAWCT-3 ′) was identified based on the motif discovery using MEME (Fig 7A). Subsequent FIMO analysis identified 397 genes containing this motif in their upstream regions in the SC19 genome (S3 Table). To further elucidate the downstream genes directly regulated by PrlP, the 397 candidate genes were cross-referenced with the 173 DEGs identified in both Δ*prlP* and *prlP*-ΔN strains. This intersection yielded 37 overlapping genes (Fig 7B and S4 Table), including *B9H01_08740*, suggesting these as the potential direct targets of PrlP.

To further validate whether *B9H01_08740* is directly regulated by PrlP, electrophoretic mobility shift assays (EMSA) were conducted using purified PrlP-His₈ protein (S7 Fig in S1 File). The results showed that PrlP specifically bound to the predicted motif located upstream of the *B9H01_08740* gene. This binding was effectively competed by an excess of unlabeled probe containing the same motif but not by a nonspecific competitor (Fig 7C). These findings confirmed that *B9H01_08740* was a direct target of PrlP.

### Identification of potential target genes affected by *B9H01_08740* for prlP function

Given that *B9H01_08740* restores bacterial growth, normalizes chain morphology, and enhances tolerance to multiple stress conditions in the Δ*prlP* background (Fig 6), it likely serves as a key downstream effector in the PrlP regulatory network.

**Fig 8:**
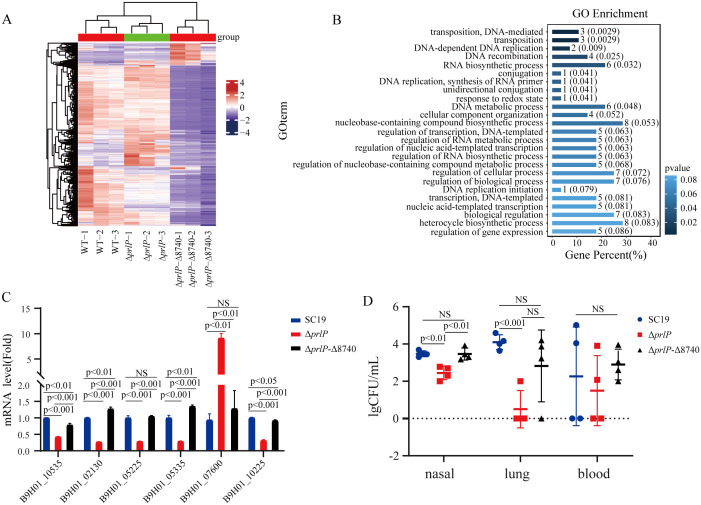
*B9H01_08740* gene influences downstream genes and experimental intranasal infection of ICR mice. (A) Heatmap showing DEGs among WT, Δ*prlP*, and Δ*prlP*-Δ8740 strains based on RNA-seq analysis. Firebrick and navy indicate upregulated and downregulated genes, respectively. (B) GO enrichment analysis of DEGs identified in the Δ*prlP*-Δ8740 vs. Δ*prlP* comparison. (C) RT-qPCR validation of representative DEGs modulated by *B9H01_08740* (two-tailed unpaired *t*-test, *n* = 3). (D) Bacterial burdens in ICR mice 72 hours after intranasal inoculation with 5 × 10⁹ CFUs of WT, Δ*prlP*, or Δ*prlP*-Δ8740 strains. Colony-forming units were determined in TNL, lung, and blood samples (two-tailed unpaired *t*-test, *n* = 4). Data are presented as mean ± standard deviation. NS, not significant; *p* < 0.05, significant.

To identify genes affected by *B9H01_08740* that restore the phenotype of Δ*prlP*, we compared the global transcriptomic profiles of the Δ*prlP*-Δ8740, Δ*prlP*, and WT strains. Relative to the Δ*prlP* strain, a total of 572 genes were differentially expressed in the Δ*prlP*-Δ8740 background, including 267 upregulated and 305 downregulated genes (Fig 8A and S5 Table). Given that PrlP represses *B9H01_08740* expression, we hypothesized that genes exhibiting inverse expression patterns between the Δ*prlP* and Δ*prlP*-Δ8740 strains may represent downstream genes of *B9H01_08740*. By intersecting the 572 DEGs (Δ*prlP*-Δ8740 vs. Δ*prlP*) with the 173 DEGs (Δ*prlP* vs. WT), 107 overlapping genes were identified. Among these, 75 genes were upregulated in Δ*prlP* but downregulated in Δ*prlP*-Δ8740, while 32 showed the opposite trend (S5 Table). GO enrichment analysis revealed that these genes are involved in a broad range of physiological processes in *S. suis*, including metabolism, stress response, and membrane transport (Fig 8B). To validate these results, RT-qPCR was performed to assess the expression of representative genes in the Δ*prlP*-Δ8740, Δ*prlP*, and wild-type strains. These included a membrane protein associated with growth (*B9H01_07600*), a chromosome partitioning protein (*B9H01_10535*), and stress-response regulators such as *ciaR* (*B9H01_05335*), an arginine repressor (*B9H01_10225*), the redox-sensing repressor *rex* (*B9H01_05225*), and a serine/threonine protein kinase (*B9H01_02130*) (Fig 8C). RT-qPCR results confirmed that the expression of these genes is influenced by *B9H01_08740*, further supporting *B9H01_08740* as a downstream target of PrlP that can modulate genes involved in *S. suis* growth, stress adaptation, and virulence.

### *B9H01_08740* is essential for colonization fitness in the nasal tract of *S. suis,* regulated by PrlP

Given that colonizing *S. suis* serves as the origin of both swine and human infections, we directly assessed the role of PrlP in upper respiratory tract colonization using a trachea-nasal lavage (TNL) assay. At 72 hours post-intranasal inoculation with 5 × 10⁹ CFUs, approximately 3 × 10³ CFUs of wild-type bacteria were recovered from TNL samples. In contrast, only approximately 3 × 10² CFUs of the Δ*prlP* strain were recovered under the same conditions (Fig 8D), indicating that PrlP promotes colonization of the upper respiratory tract. Notably, deletion of *B9H01_08740* in the Δ*prlP* background restored colonization capacity to wild-type levels. These results further establish *B9H01_08740* as a critical effector mediating PrlP-dependent colonization fitness of *S. suis* in the nasal tract.

## Discussion

**Fig 9:**
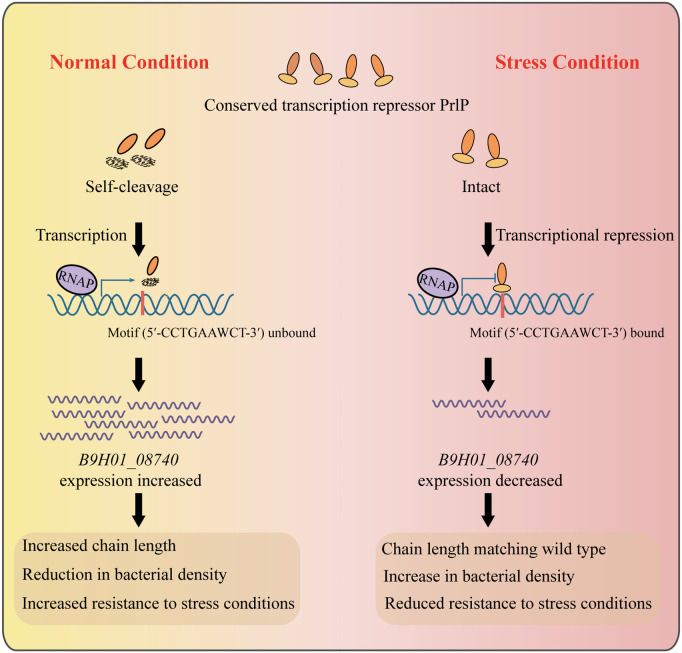
Model for PrlP-mediated transcriptional regulation of *B9H01_08740* in response to environmental stress in *S. suis.* Under normal or mildly alkaline conditions, PrlP undergoes self-cleavage, leading to degradation of its N-terminal DNA-binding domain. As a result, it fails to bind the DNA motif (5′-CCTGAAWCT-3 ′), relieving transcriptional repression and allowing *B9H01_08740* expression. This promotes elongated chain morphology, reduced bacterial density, and increased stress resistance. In contrast, under stress conditions, such as acidic pH, oxidative, hyperosmotic, or thermal stress, PrlP remains intact and binds the motif, thereby repressing *B9H01_08740* transcription. This repression restores wild-type chain length, enhances bacterial proliferation, but reduces stress tolerance. This dynamic regulatory mechanism enables *S. suis* to fine-tune its physiological responses and adapt to fluctuating microenvironments in the upper respiratory tract. All figure elements were created by the authors using Adobe Illustrator 2024, without the inclusion of any third-party copyrighted material. This figure is fully compliant with the CC BY 4.0 license.

As an important opportunistic pathogen in pigs and humans, *S. suis* remains poorly understood in terms of its mechanisms for colonizing the upper respiratory tract and causing disease following invasion [42,43]. In this study, we identified that PrlP responds to stress conditions and regulates nasal colonization and virulence. PrlP employs a stress-induced self-cleavage mechanism mediated by its C-terminal peptidase domain to dynamically control the stability and regulatory activity of its N-terminal DNA-binding domain, thereby modulating the expression of key downstream genes such as *B9H01_08740* and regulating *S. suis* chain morphology, growth, stress adaptation, and virulence (Fig 9). Collectively, these findings reveal a novel mechanism by which *S. suis* responds to stress conditions via PrlP, promoting colonization in the nasal cavity and enhancing virulence following host invasion.

Unlike classical two-component systems such as CiaR/H and SalK/SalR [44,45], which rely on separate sensor and regulator proteins, PrlP integrates both functions within a single LexA-like transcriptional regulator. This dual functionality is supported by its two distinct domains: an N-terminal DNA-binding domain responsible for transcriptional regulation and a C-terminal peptidase domain that mediates self-cleavage. Based on previous studies of LexA-like proteins, the C-terminal domain typically contains a conserved serine-lysine dyad that mediates self-cleavage between adjacent alanine and glycine residues [28–30]. Subsequently, the resulting Val-Ala-Ala-COOH sequence at the cleavage site stimulates the proteases ClpXP and ClpCP to further degrade the N-terminal [31], thereby abolishing its regulatory function. Our study suggested that PrlP may employ a similar mechanism to regulate *S. suis* chain morphology, growth, stress adaptation, and virulence. When external stress is absent, the N-terminal domain of PrlP represses the expression of downstream genes. Upon exposure to stress, the C-terminal domain of PrlP mediates self-cleavage and generates a degradation signal that promotes further degradation of the N-terminal domain, thereby lifting the repression of downstream genes (Figs 3–5). This mechanism enables PrlP to sense stress conditions and dynamically regulate stress adaptation and virulence through a LexA-like pathway (Figs 1 and 2). However, the precise mechanism by which the C-terminal domain senses stress and initiates cleavage remains to be elucidated. Given that *S. suis* encodes several LexA-like regulators, future studies are warranted to elucidate whether this regulatory mode is widespread and how it contributes to broader aspects of bacterial physiology.

Although the binding motifs of LexA proteins have been characterized in various bacteria [46–48], these motifs show significant diversity across different species. The present study firstly characterized the binding motif (5′-CCTGAAWCT-3′) of the PrlP protein, and then identified 37 potential target genes directly regulated by PrlP through an integrative multi-omics approach combining RNA-seq and ChIP-seq data analysis. Among these target genes, knockout of *B9H01_8740* in Δ*prlP* could partially rescue the growth, resistance to mildly acidic, hyperosmotic, and thermal stress conditions, suggesting that *B9H01_08740* was directly regulated by PrlP (Fig 6E–6H). *B9H01_08740* encodes a large protein (1,122 amino acids) with conserved RNase E and YebA superfamily domains (S8 Fig in S1 File), yet lacks identifiable homologs outside of *S. suis*, raising the possibility of species-specific functionality. Consequently, its specific substrates and underlying molecular mechanisms remain to be elucidated. To probe its function, RNA-seq analysis was performed on the Δ*prlP*-Δ8740 strain, revealing broad expression changes in genes involved in growth regulation, cell division, acid resistance, and other stress responses (Fig 8B). These observations further supported that *B9H01_08740* acts as a key mediator connecting PrlP activity to downstream adaptive responses. However, results from the integrative multi-omics approach indicate that *B9H01_08740* is not the sole direct target of PrlP. The broader PrlP-dependent regulatory network and its potential interactions with other transcriptional systems warrant further investigation.

In summary, this study identified PrlP as a conserved transcription factor in *S. suis* that modulates colonization and pathogenicity in response to stress conditions. Our findings reveal a novel stress-responsive regulatory system and provide new insights into the molecular mechanisms underlying bacterial pathogenesis. Importantly, targeting the dynamic sensing and regulatory functions of PrlP offers a promising strategy for infection prevention by disrupting bacterial colonization.

## Materials and methods

### Ethics statement

The experimental infectious studies were performed in strict accordance with the Guide for the Care and Use of Laboratory Animals Monitoring Committee of Hubei Province, China, and the protocol was approved by the Scientific Ethics Committee of Huazhong Agricultural University (Permit Number: HZAUMO-2019–108). All efforts were made to minimize the suffering of the animals.

### Bacterial strains, plasmids, and growth conditions

Bacterial strains and plasmids used in this study are listed in S6 Table. The *S. suis* strains and its isogenic mutants were grown in tryptic soy broth (TSB; BD, Franklin Lakes, NJ, USA) or on tryptic soy agar (TSA; BD, Franklin Lakes, NJ, USA) with 10% (v/v) fetal bovine serum (FBS; Gibco, Grand Island, NY, USA) at 37°C. *E. coli* DH5α, serving as the host strain for cloning, was cultured in lysogeny broth (LB; Gibco, Grand Island, NY, USA) or plated onto LB agar (LB; Gibco, Grand Island, NY, USA) at 37°C. If necessary, antibiotics were added to the media at the following concentrations: spectinomycin (Spc, *E. coli* 50 μg/mL, *S. suis* 100 μg/mL) and kanamycin (Kan, 50 μg/mL).

### Strain construction

All primers used for strain construction are listed in S7 Table. As described in the previous study [49], deletions and insertions were generated using homologous recombination in bacteria. First, upstream and downstream fragments of the target genes were amplified from the SC19 genome. The upstream and downstream fragments were fused as intact fragments by overlapping PCR, and they were infused into the suicide vector pSET4s. The constructed pSET4s plasmids were electroporated into WT using the electroporation system (BTX, Beijing, China) (voltage: 2300 V, cuvette: 2 mm) [50,51]. We screened the single-crossover mutants at 37°C by culturing the bacteria with Spc antibiotic and obtained the double-crossover mutants at 28°C by repeatedly passaging on TSA without Spc. The resulting mutant strain was confirmed by PCR using external primers.

To construct complemented strain, the coding sequence and promoter of the gene were amplified using primers [52] and infused into the vector pSET2. The constructed pSET2 was introduced into SC19 by electroporation (BTX, Beijing, China) (voltage: 2300 V, cuvette: 2 mm).

### Bacterial growth curve assays

Bacterial growth was monitored using a Bioscreen C optical growth analyzer (Lab Systems Helsinki, Vantaa, Finland). Strains of *S. suis* SC19 and its isogenic mutants were cultured overnight in TSB at 37°C. The cultures were subsequently diluted 1:100 to an initial OD600 of 0.01 and transferred into a sterile 100-well microplate. The microplate was incubated at 37°C with continuous shaking at 200 rpm for 24 hours. Optical density at 600 nm (OD600) was measured at 1-hour intervals throughout the incubation period.

### Microscopy image

All strains were collected to an OD600 of 0.6 and then resuspended twice using PBS. Each sample (20 μl) was fixed on glass slides (FANYIGX; Yancheng, China) through flaming. A Gram staining kit (solarbio; Beijing, China) was utilized according to the instructions of the manufacturer.

### Bacterial density assays of *S. suis* in acidic conditions

All strains were incubated overnight, then diluted 1:100 into fresh TSB medium with 20 mM Tris (pH 6.0, pH 7.0, pH 8.0), and incubated at 37°C for 8 hours. After vortexing, 200 µL of the bacterial culture was transferred into a 96-well plate. The optical density at 600 nm was monitored using a Multimode Plate Reader (Tecan, Switzerland).

### Survival assays of *S. suis* in oxidative stress conditions

The bacteria were cultured in TSB at 37°C for 8 hours, and H_2_O_2_ was added to the bacterial suspension to a final concentration of 20 mM. The mixtures were incubated for 1h at 37°C. Serial dilutions of bacteria were plated onto TSA to count viable bacteria before and after exposure to H_2_O_2_. The experiments were conducted in duplicate and repeated three times.

### Bacterial density assays of *S. suis* under hyperosmotic and thermal stress conditions

Overnight cultures of *S. suis* strains were diluted 1:100 into fresh TSB medium with or without 1.5% NaCl and incubated at 37°C for 8 hours. For thermal stress assays, cultures were incubated in standard TSB medium at 42°C for 8 hours. After gentle vortexing, 200 µL of each culture was transferred into a 96-well plate. The optical density at 600 nm (OD600) was measured using a multimode plate reader (Tecan, Switzerland).

### Determination of *PrlP* protein stability

The Δ*prlP* strain harboring the plasmid pSET2-ENO-PrlP-Flag was cultured in 10 mL of TSB medium at 37°C until reaching mid-log phase (OD600 ≈ 0.6). Cells were then centrifuged and resuspended in 10 mL of fresh TSB supplemented with 20 mM Tris buffer (pH 6.0 or pH 8.0), 20 mM H₂O₂ (for oxidative stress), 1.5% NaCl (for hyperosmotic stress), or incubated at 42°C (for heat stress). Cultures were incubated under these conditions for 1 hour. Following treatment, cells were harvested by centrifugation at 12,000 rpm for 10 minutes at 4°C. The resulting bacterial pellets were resuspended in 50 µL of PBS and mixed with 5 × SDS loading buffer. Samples were then boiled at 100°C for 20 minutes. Proteins were separated by 12% SDS-PAGE and transferred for Western blot analysis.

PrlP stability was evaluated by probing for full-length and degraded PrlP using anti-Flag antibodies, with GAPDH serving as a loading control. The degradation ratio of PrlP was quantified by grayscale densitometry and calculated using the following formula:


$$[\frac{D_{0}}{(D_{0}+I_{0})}]\times 100\%,$$

where D₀ represents the intensity ratio of degraded PrlP to GAPDH, and I₀ represents the intensity ratio of intact PrlP to GAPDH. Statistical significance was determined using two-tailed unpaired *t*-tests (n = 3).

### Mouse infection experiment

Four-week-old BALB/c female mice with similar body weights were randomly grouped into groups of 10 and challenged with 0.5 mL of *S. suis* strains (1 × 10^8^ CFUs/mouse) by an intraperitoneal (i.p.) injection to evaluate the pathogenicity of the different strains. A negative control group was set, which was injected with the same volume of PBS. Mouse survival after infection was recorded every 24 h for 7 d. Animals were examined every 8 hours. Health status was rated using a clinical score sheet [53]: 0 = normal response to stimuli; 1 = ruffled coat and slow response to stimuli; 2 = respond only to repeated stimuli; 3 = non-responsive or walking in circles; and 4 = dead. Mice exhibiting extreme lethargy or neurological signs (score = 3) were considered moribund and were humanely euthanized.

In addition to assessing the survival rate of mice and the bacterial burden, the cytokine response of different SC19 strains within mouse was evaluated. Four-week-old BALB/c female mice were randomly separated into groups of 20. WT and mutant strains were challenged with 0.5 mL (1 × 10^8^ CFUs/mouse) by an intraperitoneal (i.p.) injection. Samples were collected at 6 hours, 12 hours, and 24 hours post-infection from each group, with five mice from each group euthanized and dissected at each time point. Blood, liver, lung, and brain tissues were collected under a biosafety cabinet. A portion of the collected blood was immediately serially diluted and plated on TSA plates containing 10% fetal bovine serum. The remaining blood was used to prepare serum. Approximately 0.1 g of liver, lung, and brain tissues were placed in homogenization tubes with PBS, thoroughly homogenized, and then serially diluted and plated on TSA plates containing 10% fetal bovine serum. The plates were incubated overnight at 37°C in an incubator, and the number of bacterial colonies was counted. The remaining serum was used to measure the biochemical parameters of the mice, including the levels of 10 cytokines (TNF-α, IFN-γ, IL-1β, IL-4, IL-6, IL-10, IL-12p70, IL-17A, IL-23, and KC) in mouse serum were measured using the U-plex ultrasensitive multiplex assay kit (Meso Scale Discovery; Shanghai, China).

### *Galleria mellonella* infection experiment

*Galleria mellonella* larvae (purchased from Wuhan Weiwuqiyuan Biotechnology Co., Ltd., Wuhan, China) were stored in the dark at 4°C before use. Larvae weighing between 0.4 and 0.5 g were selected and injected with 25 µL of bacterial suspension (approximately 2.5 × 10⁶ CFUs) into the left posterior proleg using a Hamilton syringe. Each experimental group consisted of 10 larvae. Infections with *S. suis* SC19 served as the positive control, while PBS injections served as the negative control. Larval survival was monitored at 12, 24, 36, and 48 hours post-infection (hpi).

### RNA isolation and RNA-seq

The RNA extraction method was modified based on previously reported methods [54]. All strains were cultured at 37°C to an OD600 of 0.6 and then immediately frozen in liquid nitrogen. Three replicates were performed for each group. For extraction, samples were resuspended by vortexing in 50μL lysozyme (20 mg/mL; Beyotime, Shanghai, China) and incubated for 30 minutes, followed by the addition of 1 mL of TransZol Up (TransGen Biotech; Beijing, China). Total RNA was extracted twice with chloroform–isoamyl alcohol (24:1, v/v) and precipitated in 0.7 volumes of isopropanol. After a washing step with 70% ethanol, the RNA pellet was dissolved in sterile DNase- and RNase-free water (TransGen Biotech; Beijing, China) and quantified by absorbance at 260 and 280 nm. The purity and integrity of RNA were controlled on agarose gels. Each sample’s extracted RNA was divided into two portions: one for sequencing, and the other was stored at −80°C for subsequent quantitative fluorescence analysis.

To investigate gene expression profiles among WT, Δ*prlP*, *prlP*-ΔN, Δ*prlP*-8740, and WT strains, RNA-seq was performed using Personalbio (Shanghai, China) and Majorbio (Shanghai, China). The raw data were trimmed using trim_galore (V. 0.6.7), then, the trimmed reads were mapped to the SC19 genome (NZ_CP020863.1) using HISAT2 (V. 2.2.1). Following alignment, read counts for each gene were generated using FeatureCounts (V. 2.0.3). Gene expression levels were quantified using the Fragments Per Kilobase Million (FPKM) method, which normalizes read counts by gene length and sequencing depth. This analysis approach facilitated the identification of DEGs between experimental conditions. The DESeq R package was employed for statistical analysis of DEGs. The genes with an adjusted p-value < 0.05 and a differential expression of at least twofold were designated as differentially expressed. Subsequently, a heat map depicting Differential Metabolites (DEMs), along with GO enrichment analysis and KEGG enrichment analysis, were conducted using the OmicShare bioinformatics learning platform (www.omicshare.com/tools).

### RT-qPCR assays

Quantitative real-time PCR (RT-qPCR) was conducted to evaluate gene expression under various stress conditions. The experiments were performed in two parts. In the first part, the wild-type *S. suis* strain SC19 was grown to mid-log phase (OD600 ≈ 0.6) in TSB medium and then incubated for 1 hour under different conditions: mildly acidic pH (pH 6.0), mildly alkaline pH (pH 8.0), oxidative stress (20 mM H₂O₂), hyperosmotic stress (1.5% NaCl), or heat stress (42°C). Total RNA was extracted from each condition to assess the expression of *prlP* and *B9H01_08740*. In the second part, total RNA was extracted from various mutant strains grown under standard conditions as well as under the same stress conditions described above (pH 6.0, pH 8.0, 20 mM H₂O₂, 1.5% NaCl, and 42°C). In addition, RNA samples obtained from RNA-seq experiments were used to validate the expression of *B9H01_08740* and other selected differentially expressed genes.

RNA was isolated using the RNAiso Plus reagent (TaKaRa, Dalian, China) according to the manufacturer’s protocol. cDNA synthesis was performed using the M-MLV Reverse Transcriptase Kit (Promega; Madison, WI, USA). Gene-specific primers used for validation are listed in S7 Table. RT-qPCR was performed using ChamQ Blue Universal SYBR qPCR Master Mix (Vazyme; Nanjing, China) on a QuantStudio 5 system (Thermo Fisher Scientific). Transcript levels were normalized to the housekeeping gene *gdh* and calculated using the 2^^–ΔΔCt^ method. All reactions were carried out in biological triplicates, each with technical duplicates.

### ChIP-seq

The ChIP assay was performed as previously described [55]. For the Flag-tagged plasmids, the open reading frame of *prlP* was amplified by PCR from the SC19 genome and cloned into the pSET2 plasmid. The Δ*prlP* strain containing empty pSET2-ENO-3 × Flag was cultured in 40 mL TSB medium until the mid-log phase (OD600 ≈ 0.6). Cultured bacteria were then cross-linked with 1% formaldehyde for 10 min at room temperature. Cross-linking was stopped by the addition of 125 mM glycine. The cell was resuspended in lysis buffer and was sonicated to fragment chromosomal DNA in the range of 0.2–1.0 kb. Insoluble cellular debris was removed by centrifugation at 4°C and the supernatant was used as the input sample in IP experiments. Both control and IP samples were then incubated with 50 μL agarose conjugated anti-FLAG antibodies (Sigma-Aldrich; St. Louis, MO, USA) in an IP buffer. Washing, crosslink reversal, and purification of the ChIP DNA were conducted [56]. DNA fragments (150–250 bp) were selected for library construction and sequencing libraries were prepared using the NEBNext Ultra II DNA Library Prep Kit for Illumina (New England Biolabs; Beverly, MA, USA). The libraries were sequenced using the HiSeq 2000 system (Illumina). Two biological replications have been performed for all ChIP-seq experiments. ChIP-seq reads were mapped to the SC19 genome (NZ_CP020863.1) using Bowtie2 (V. 2.3.4.1). Uniquely mapped reads were retained for the subsequent analyses. Binding peaks (q < 0.01) were identified using MACS2 software (V. 2.1.1). BEDtools (V. 2.26.0) were used to merge and intersect peak intervals. The *PrlP* binding consensus motif was identified using the MEME software with sequences spanning 100 bp to each side of the ChIP-seq peak summits. The FIMO algorithm was then used to determine the occurrence of the *PrlP* motif across the genome and to identify *PrlP*’s target genes.

### EMSA

The binding of the PrlP protein to the *B9H01_08740* gene was detected using native polyacrylamide gel electrophoresis. Purified PrlP-His_8_ (1 mM, 2 mM, 3 mM) was incubated with FAM-labeled DNA probes (FAM’-TCGGCAGAATTAGTTTCCGGTGAAGTTTGA sequence, 50 nM) in a 20 μL incubation system containing EMSA buffer (250 mM NaCl, 2.5 mM DTT, 0.5 mM EDTA, 125 mM Tris, pH 6.0), 10% glycerol, 0.07 mg/mL BSA protein, and ddH_2_O to make up the total volume to 20 μL. After incubating at 30°C for 30 minutes, the samples were loaded onto a native polyacrylamide gel and electrophoresed at 100 V for 120 minutes. Finally, the gels were scanned using Typhoon5 (GE Healthcare, Uppsala, Sweden).

### Intranasal infection of mice

The nasal infection of mice was modified based on previously reported methods [57]. To evaluate the survival rates of WT, Δ*prlP*, and Δ*prlP*-8740 strains in the nasal cavity of mice, we conducted a mouse nasal infection experiment. A total of 16 Four- to six-week-old ICR female mice with similar body weights were randomly divided into four groups. Before experimentation, the mice were given one week to acclimatize to the environment to minimize stress. All strains were collected at an OD600 of 0.6, while the control group was infected with an equal volume of PBS. Before infection, mice were anesthetized via inhalation of isofluran (IsoFlo; Albrecht), and the mice were infected with 5 × 10^9^ (CFUs/mouse). At 72 hours post-infection, all mice were euthanized, and their lungs and blood were collected for bacterial burden analysis as previously described. Tracheonasal lavage (TNL) was used as a measure of nasal bacterial burden. Therefore, 1 mL of PBS was used for retrograde irrigation of the nasal cavity. The lavage fluid was then serially diluted and analyzed for bacterial burden following the previously described methods.

### Statistical analysis

Unless otherwise specified, the data were analyzed using two-tailed, unpaired t-tests. All assays were repeated at least three times, and the data were expressed as the mean ± standard deviations. For the animal infection experiments, comparisons of survival rates and clinical scores were analyzed with a log-rank test or two-way RM ANOVA, respectively, using GraphPad Prism 8.0 For all tests, a value of p < 0.05 was considered the threshold for significance.

## Supporting information

S1 File S1 Fig. Screening for mutants with growth defects under acidic condition. (A) Screening for key genes involved in *S. suis* proliferation under acidic conditions. The S. suis transposon mutant library is cultured under acidic conditions to identify essential genes. The selected mutants are linked to sequencing adaptors, amplified, and then subjected to high-throughput sequencing. Target genes are knocked out and complemented, followed by growth curve analysis to validate their impact on bacterial growth. (B) Screening for *S. suis* mutants with growth defects under acidic conditions. Transposon mutants were cultured in TSB medium adjusted to pH 6.0 and pH 7.0 for 8 hours, and OD600 values were recorded to calculate the growth ratio (pH 6.0/ pH 7.0) as an indicator of acid tolerance. This figure shows the screening data from one representative 96-well plate containing *prlP* group mutants (as an example subset of the overall library). Several mutants exhibited impaired growth under acidic conditions, among which strain No. 53 consistently showed the most pronounced and reproducible growth defect across independent experiments (highlighted in red) and was therefore selected for further analysis. Data are presented as mean ± SD, and statistical analysis was performed using two-tailed unpaired t-tests (n = 3). S2 Fig. Construct *prlP* gene knockout and complementation strains. (A) The confirmation of Δ*prlP* with PCR reaction. The pairs of primers included the external primers (PrlP-L-F/R-R) and the internal primers (PrlP-F/R). S3 Fig. Functional conservation of PrlP in the reference *S. suis* strain P1/7. (A) Growth curves of wild-type P1/7 and the isogenic Δ*prlP* mutant in TSB at 37°C. The Δ*prlP* strain showed significantly impaired growth compared to the wild type. (B) Growth performance under different pH conditions (pH 6.0, 7.0, and 8.0) after 8 h of incubation. The Δ*prlP* mutant exhibited reduced growth under acidic conditions and mild impairment at pH 7.0, similar to the phenotype observed in the SC19 background. (C) Stress tolerance assays under hyperosmotic (1.5% NaCl) and heat (42°C) stress conditions. The Δ*prlP* mutant was more sensitive to both stressors compared to the wild-type P1/7. Data are presented as mean ± SD from three independent experiments, with statistical significance determined by two-tailed unpaired *t*-tests. S4 Fig. Construct PrlP domain-specific knockout and complementation strains. (A) The confirmation of *prlP-*ΔN and *prlP-*ΔC with PCR reaction. The pairs of primers included the primers (PrlP-ΔN-L-F/R-R) and primers (PrlP-ΔC-L-F/R-R). S5 Fig. Construction and verification of the *prlP*-ΔC_*self-cleavage*_ mutants. PCR confirmation of the *prlP*-ΔC_*self-cleavage*_ deletion mutant using external primer pair PrlP-C_*self-cleavage*_-EX-F/R. S6 Fig. Construct double-knockout strains for potential PrlP-regulated genes. (A) The confirmation of Δ*prlP* with PCR reaction. The confirmation of Δ*prlP* with PCR reaction. The pairs of primers included the primers (PrlP-ΔN-L-F/R-R) and primers (PrlP-ΔC-L-F/R-R). S7 Fig. Expression and purification analysis of PrlP. (A) SDS-PAGE and Western blot analysis of the expressed and purified PrlP under denaturing conditions. The gel shows the presence of PrlP at the expected molecular weight. S8 Fig. Structural prediction of *B9H01_08740*. Domain architecture of the *B9H01_08740*-encoded protein. The protein consists of 1,122 amino acids and contains conserved motifs corresponding to the YebA superfamily (residues 222–350) and the ribonuclease E superfamily (residues 532–1002), as predicted by domain analysis.
(RAR)

S1 Table The conservation of the *PrlP* nucleic acid sequence among all strains of *Streptococcus suis.* (DOCX)

S2 Table Expression levels of differentially expressed genes in Δ*prlP* and *prlP*-ΔN compared to WT. (DOCX)

S3 Table Screen for genes containing the *PrlP* protein binding motif on the SC19 genome. (DOCX)

S4 Table Genes directly regulated by *PrlP* in the SC19 genome. (DOCX)

S5 Table Expression levels of genes in Δ*prlP*-Δ*8740* compared to Δ*prlP.* (DOCX)

S6 Table Strains and plasmids used in this study. (DOCX)

S7 Table Primers used in this study. (DOCX)
