# Supplementary material for: The conserved transcription factor PrlP modulates colonization and pathogenicity of Streptococcus suis in response to environmental stress
Source: PLoS Pathog. 2025 Jul 18;21(7):e1013314. doi: 10.1371/journal.ppat.1013314 (PMC12273997; doi:10.1371/journal.ppat.1013314)
Supplement: S1 Table — (DOCX) [file ppat.1013314.s002.docx]

**Table S1.** The conservation of the *PrlP* nucleic acid sequence among all strains of *Streptococcus suis*.

| subject acc | % identity | alignment length | mismatches | gap opens | s. start | s. end |
| --- | --- | --- | --- | --- | --- | --- |
| NZ_LR738721.1 | 100 | 801 | 0 | 0 | 937011 | 937811 |
| NZ_CAAALZ010000009.1 | 100 | 801 | 0 | 0 | 271406 | 272206 |
| NZ_LS483418.1 | 100 | 801 | 0 | 0 | 924577 | 925377 |
| NZ_FWZS01000033.1 | 100 | 801 | 0 | 0 | 259946 | 260746 |
| NZ_FISV01000001.1 | 100 | 801 | 0 | 0 | 30046 | 29246 |
| NZ_FIPV01000002.1 | 100 | 801 | 0 | 0 | 30062 | 29262 |
| NZ_FIOP01000003.1 | 100 | 801 | 0 | 0 | 204495 | 205295 |
| NZ_FION01000002.1 | 100 | 801 | 0 | 0 | 259971 | 260771 |
| NZ_FILH01000001.1 | 100 | 801 | 0 | 0 | 271036 | 271836 |
| NZ_FILA01000003.1 | 100 | 801 | 0 | 0 | 259921 | 260721 |
| NZ_FIJV01000002.1 | 100 | 801 | 0 | 0 | 271026 | 271826 |
| NZ_FIFI01000002.1 | 100 | 801 | 0 | 0 | 30031 | 29231 |
| NZ_FIFD01000002.1 | 100 | 801 | 0 | 0 | 30025 | 29225 |
| NZ_FIOO01000001.1 | 100 | 801 | 0 | 0 | 384030 | 384830 |
| NZ_FIMW01000001.1 | 100 | 801 | 0 | 0 | 343863 | 344663 |
| NZ_FIMP01000001.1 | 100 | 801 | 0 | 0 | 27027 | 26227 |
| NZ_FISO01000002.1 | 100 | 801 | 0 | 0 | 338322 | 339122 |
| NZ_FISS01000001.1 | 100 | 801 | 0 | 0 | 30274 | 29474 |
| NZ_FIQP01000002.1 | 100 | 801 | 0 | 0 | 338422 | 339222 |
| NZ_FITQ01000002.1 | 100 | 801 | 0 | 0 | 30046 | 29246 |
| NZ_FISW01000008.1 | 100 | 801 | 0 | 0 | 30049 | 29249 |
| NZ_FIMG01000002.1 | 100 | 801 | 0 | 0 | 30063 | 29263 |
| NZ_FIMF01000002.1 | 100 | 801 | 0 | 0 | 271126 | 271926 |
| NZ_FILC01000002.1 | 100 | 801 | 0 | 0 | 271181 | 271981 |
| NZ_FIJW01000001.1 | 100 | 801 | 0 | 0 | 341160 | 341960 |
| NZ_FIJT01000002.1 | 100 | 801 | 0 | 0 | 30022 | 29222 |
| NZ_FISJ01000002.1 | 100 | 801 | 0 | 0 | 338310 | 339110 |
| NZ_FIQR01000001.1 | 100 | 801 | 0 | 0 | 30061 | 29261 |
| NZ_FIPW01000002.1 | 100 | 801 | 0 | 0 | 30061 | 29261 |
| NZ_FIKX01000002.1 | 100 | 801 | 0 | 0 | 271173 | 271973 |
| NZ_FIIV01000004.1 | 100 | 801 | 0 | 0 | 118606 | 119406 |
| NZ_FISK01000001.1 | 100 | 801 | 0 | 0 | 30061 | 29261 |
| NZ_FIOM01000001.1 | 100 | 801 | 0 | 0 | 356358 | 357158 |
| NZ_FIQA01000001.1 | 100 | 801 | 0 | 0 | 30063 | 29263 |
| NZ_FIPX01000004.1 | 100 | 801 | 0 | 0 | 30267 | 29467 |
| NZ_FIPF01000002.1 | 100 | 801 | 0 | 0 | 338315 | 339115 |
| NZ_FINP01000001.1 | 100 | 801 | 0 | 0 | 30275 | 29475 |
| NZ_FINM01000001.1 | 100 | 801 | 0 | 0 | 30305 | 29505 |
| NZ_FIKA01000004.1 | 100 | 801 | 0 | 0 | 83493 | 84293 |
| NZ_FIGM01000001.1 | 100 | 801 | 0 | 0 | 321626 | 322426 |
| NZ_FIQQ01000005.1 | 100 | 801 | 0 | 0 | 70880 | 71680 |
| NZ_FIOG01000001.1 | 100 | 801 | 0 | 0 | 30045 | 29245 |
| NZ_FISF01000002.1 | 100 | 801 | 0 | 0 | 271108 | 271908 |
| NZ_FIIZ01000001.1 | 100 | 801 | 0 | 0 | 321761 | 322561 |
| NZ_FITJ01000009.1 | 100 | 801 | 0 | 0 | 60588 | 61388 |
| NZ_FIIW01000001.1 | 100 | 801 | 0 | 0 | 329382 | 330182 |
| NZ_FIFU01000002.1 | 100 | 801 | 0 | 0 | 271228 | 272028 |
| NZ_FISH01000002.1 | 100 | 801 | 0 | 0 | 338308 | 339108 |
| NZ_FISI01000001.1 | 100 | 801 | 0 | 0 | 30360 | 29560 |
| NZ_FIIU01000001.1 | 100 | 801 | 0 | 0 | 329386 | 330186 |
| NZ_FITL01000004.1 | 100 | 801 | 0 | 0 | 35457 | 34657 |
| NZ_FISB01000001.1 | 100 | 801 | 0 | 0 | 410393 | 411193 |
| NZ_FISC01000001.1 | 100 | 801 | 0 | 0 | 410385 | 411185 |
| NZ_FIRZ01000001.1 | 100 | 801 | 0 | 0 | 349600 | 350400 |
| NZ_FIOK01000002.1 | 100 | 801 | 0 | 0 | 334287 | 335087 |
| NZ_FILT01000002.1 | 100 | 801 | 0 | 0 | 276491 | 277291 |
| NZ_FILM01000007.1 | 100 | 801 | 0 | 0 | 117645 | 118445 |
| NZ_FISA01000001.1 | 100 | 801 | 0 | 0 | 30059 | 29259 |
| NZ_FIOF01000001.1 | 100 | 801 | 0 | 0 | 349577 | 350377 |
| NZ_FIOH01000002.1 | 100 | 801 | 0 | 0 | 338319 | 339119 |
| NZ_FINS01000004.1 | 100 | 801 | 0 | 0 | 193216 | 194016 |
| NZ_FINO01000002.1 | 100 | 801 | 0 | 0 | 337904 | 338704 |
| NZ_FINN01000002.1 | 100 | 801 | 0 | 0 | 344259 | 345059 |
| NZ_FINL01000002.1 | 100 | 801 | 0 | 0 | 30062 | 29262 |
| NZ_FIMK01000002.1 | 100 | 801 | 0 | 0 | 259951 | 260751 |
| NZ_FIMJ01000001.1 | 100 | 801 | 0 | 0 | 30274 | 29474 |
| NZ_FIMH01000002.1 | 100 | 801 | 0 | 0 | 271159 | 271959 |
| NZ_FIME01000001.1 | 100 | 801 | 0 | 0 | 30061 | 29261 |
| NZ_FIPP01000002.1 | 100 | 801 | 0 | 0 | 30061 | 29261 |
| NZ_FIPO01000002.1 | 100 | 801 | 0 | 0 | 259974 | 260774 |
| NZ_FIPL01000001.1 | 100 | 801 | 0 | 0 | 30275 | 29475 |
| NZ_FIOD01000001.1 | 100 | 801 | 0 | 0 | 30060 | 29260 |
| NZ_FIOE01000001.1 | 100 | 801 | 0 | 0 | 338632 | 339432 |
| NZ_FIGD01000001.1 | 100 | 801 | 0 | 0 | 271191 | 271991 |
| NZ_FIRY01000001.1 | 100 | 801 | 0 | 0 | 349612 | 350412 |
| NZ_FIPM01000002.1 | 100 | 801 | 0 | 0 | 30061 | 29261 |
| NZ_FIOB01000001.1 | 100 | 801 | 0 | 0 | 30061 | 29261 |
| NZ_FIPB01000002.1 | 100 | 801 | 0 | 0 | 338304 | 339104 |
| NZ_FINU01000001.1 | 100 | 801 | 0 | 0 | 349622 | 350422 |
| NZ_FINH01000002.1 | 100 | 801 | 0 | 0 | 30045 | 29245 |
| NZ_FIHO01000002.1 | 100 | 801 | 0 | 0 | 271065 | 271865 |
| NZ_FINV01000001.1 | 100 | 801 | 0 | 0 | 30063 | 29263 |
| NZ_FIKG01000001.1 | 100 | 801 | 0 | 0 | 267456 | 268256 |
| NZ_FIKH01000001.1 | 100 | 801 | 0 | 0 | 341168 | 341968 |
| NZ_FIQN01000001.1 | 100 | 801 | 0 | 0 | 336911 | 337711 |
| NZ_FIQL01000253.1 | 100 | 801 | 0 | 0 | 938 | 138 |
| NZ_FIPT01000002.1 | 100 | 801 | 0 | 0 | 171660 | 172460 |
| NZ_FIPK01000002.1 | 100 | 801 | 0 | 0 | 30059 | 29259 |
| NZ_FIPH01000001.1 | 100 | 801 | 0 | 0 | 30063 | 29263 |
| NZ_FINI01000002.1 | 100 | 801 | 0 | 0 | 30054 | 29254 |
| NZ_FIMV01000003.1 | 100 | 801 | 0 | 0 | 259975 | 260775 |
| NZ_FIMR01000001.1 | 100 | 801 | 0 | 0 | 338657 | 339457 |
| NZ_FIMQ01000002.1 | 100 | 801 | 0 | 0 | 259976 | 260776 |
| NZ_FIKC01000001.1 | 100 | 801 | 0 | 0 | 286808 | 287608 |
| NZ_FIJN01000002.1 | 100 | 801 | 0 | 0 | 259928 | 260728 |
| NZ_FITG01000001.1 | 100 | 801 | 0 | 0 | 30061 | 29261 |
| NZ_FIQM01000001.1 | 100 | 801 | 0 | 0 | 30054 | 29254 |
| NZ_FIPJ01000001.1 | 100 | 801 | 0 | 0 | 30043 | 29243 |
| NZ_FINT01000001.1 | 100 | 801 | 0 | 0 | 30061 | 29261 |
| NZ_FINJ01000003.1 | 100 | 801 | 0 | 0 | 271164 | 271964 |
| NZ_FILD01000008.1 | 100 | 801 | 0 | 0 | 83502 | 84302 |
| NZ_FIJH01000002.1 | 100 | 801 | 0 | 0 | 276439 | 277239 |
| NZ_FIIT01000001.1 | 100 | 801 | 0 | 0 | 271070 | 271870 |
| NZ_FISQ01000002.1 | 100 | 801 | 0 | 0 | 30063 | 29263 |
| NZ_FIRG01000001.1 | 100 | 801 | 0 | 0 | 30271 | 29471 |
| NZ_FIPN01000001.1 | 100 | 801 | 0 | 0 | 338318 | 339118 |
| NZ_FIOJ01000001.1 | 100 | 801 | 0 | 0 | 30061 | 29261 |
| NZ_FIOI01000003.1 | 100 | 801 | 0 | 0 | 171663 | 172463 |
| NZ_FIRX01000002.1 | 100 | 801 | 0 | 0 | 30148 | 29348 |
| NZ_FIQS01000001.1 | 100 | 801 | 0 | 0 | 338426 | 339226 |
| NZ_FIPG01000003.1 | 100 | 801 | 0 | 0 | 30059 | 29259 |
| NZ_FITK01000004.1 | 100 | 801 | 0 | 0 | 36749 | 35949 |
| NZ_FITE01000003.1 | 100 | 801 | 0 | 0 | 271110 | 271910 |
| NZ_FIQI01000002.1 | 100 | 801 | 0 | 0 | 30047 | 29247 |
| NZ_FIQJ01000001.1 | 100 | 801 | 0 | 0 | 375004 | 375804 |
| NZ_FIPS01000001.1 | 100 | 801 | 0 | 0 | 30062 | 29262 |
| NZ_FINC01000001.1 | 100 | 801 | 0 | 0 | 30048 | 29248 |
| NZ_FIHK01000001.1 | 100 | 801 | 0 | 0 | 271074 | 271874 |
| NZ_FITF01000009.1 | 100 | 801 | 0 | 0 | 30021 | 29221 |
| NZ_FIRV01000002.1 | 100 | 801 | 0 | 0 | 30045 | 29245 |
| NZ_FIOU01000002.1 | 100 | 801 | 0 | 0 | 338315 | 339115 |
| NZ_FIOS01000001.1 | 100 | 801 | 0 | 0 | 337902 | 338702 |
| NZ_FIJO01000001.1 | 100 | 801 | 0 | 0 | 312418 | 313218 |
| NZ_FIFR01000001.1 | 100 | 801 | 0 | 0 | 346876 | 347676 |
| NZ_FITI01000005.1 | 100 | 801 | 0 | 0 | 136157 | 136957 |
| NZ_FIRK01000001.1 | 100 | 801 | 0 | 0 | 360390 | 361190 |
| NZ_FIQB01000001.1 | 100 | 801 | 0 | 0 | 349632 | 350432 |
| NZ_FIQD01000001.1 | 100 | 801 | 0 | 0 | 354909 | 355709 |
| NZ_FITC01000006.1 | 100 | 801 | 0 | 0 | 35750 | 34950 |
| NZ_FIQG01000001.1 | 100 | 801 | 0 | 0 | 30273 | 29473 |
| NZ_FIOZ01000002.1 | 100 | 801 | 0 | 0 | 387084 | 387884 |
| NZ_FINE01000002.1 | 100 | 801 | 0 | 0 | 30061 | 29261 |
| NZ_FIOA01000002.1 | 100 | 801 | 0 | 0 | 271258 | 272058 |
| NZ_FINF01000002.1 | 100 | 801 | 0 | 0 | 30043 | 29243 |
| NZ_FIQO01000001.1 | 100 | 801 | 0 | 0 | 30270 | 29470 |
| NZ_FIQH01000001.1 | 100 | 801 | 0 | 0 | 354788 | 355588 |
| NZ_FIPC01000001.1 | 100 | 801 | 0 | 0 | 338320 | 339120 |
| NZ_FITD01000002.1 | 100 | 801 | 0 | 0 | 330952 | 331752 |
| NZ_FISD01000002.1 | 100 | 801 | 0 | 0 | 30328 | 29528 |
| NZ_FISE01000001.1 | 100 | 801 | 0 | 0 | 39034 | 38234 |
| NZ_FIQU01000001.1 | 100 | 801 | 0 | 0 | 30062 | 29262 |
| NZ_FIPR01000001.1 | 100 | 801 | 0 | 0 | 30060 | 29260 |
| NZ_FILQ01000002.1 | 100 | 801 | 0 | 0 | 265372 | 266172 |
| NZ_FILI01000002.1 | 100 | 801 | 0 | 0 | 271176 | 271976 |
| NZ_FILJ01000002.1 | 100 | 801 | 0 | 0 | 271232 | 272032 |
| NZ_FITB01000001.1 | 100 | 801 | 0 | 0 | 30063 | 29263 |
| NZ_FIRS01000001.1 | 100 | 801 | 0 | 0 | 30272 | 29472 |
| NZ_FIRO01000001.1 | 100 | 801 | 0 | 0 | 338306 | 339106 |
| NZ_FINZ01000002.1 | 100 | 801 | 0 | 0 | 30275 | 29475 |
| NZ_FIRW01000001.1 | 100 | 801 | 0 | 0 | 30274 | 29474 |
| NZ_FIRP01000001.1 | 100 | 801 | 0 | 0 | 349611 | 350411 |
| NZ_FIRM01000002.1 | 100 | 801 | 0 | 0 | 349556 | 350356 |
| NZ_FIRF01000001.1 | 100 | 801 | 0 | 0 | 349606 | 350406 |
| NZ_FIRN01000001.1 | 100 | 801 | 0 | 0 | 30272 | 29472 |
| NZ_FIRL01000002.1 | 100 | 801 | 0 | 0 | 30274 | 29474 |
| NZ_FIRJ01000002.1 | 100 | 801 | 0 | 0 | 30060 | 29260 |
| NZ_FIRE01000001.1 | 100 | 801 | 0 | 0 | 30046 | 29246 |
| NZ_FINY01000001.1 | 100 | 801 | 0 | 0 | 349610 | 350410 |
| NZ_FINR01000001.1 | 100 | 801 | 0 | 0 | 30062 | 29262 |
| NZ_FILZ01000001.1 | 100 | 801 | 0 | 0 | 276168 | 276968 |
| NZ_FILG01000006.1 | 100 | 801 | 0 | 0 | 117615 | 118415 |
| NZ_FIJJ01000002.1 | 100 | 801 | 0 | 0 | 271234 | 272034 |
| NZ_FISG01000002.1 | 100 | 801 | 0 | 0 | 30063 | 29263 |
| NZ_FIRH01000001.1 | 100 | 801 | 0 | 0 | 349620 | 350420 |
| NZ_FILY01000003.1 | 100 | 801 | 0 | 0 | 257980 | 258780 |
| NZ_FIJR01000002.1 | 100 | 801 | 0 | 0 | 271030 | 271830 |
| NZ_FIHC01000002.1 | 100 | 801 | 0 | 0 | 259928 | 260728 |
| NZ_FIGY01000002.1 | 100 | 801 | 0 | 0 | 258692 | 259492 |
| NZ_FIGX01000001.1 | 100 | 801 | 0 | 0 | 357988 | 358788 |
| NZ_FIFK01000001.1 | 100 | 801 | 0 | 0 | 412843 | 413643 |
| NZ_FITA01000001.1 | 100 | 801 | 0 | 0 | 30048 | 29248 |
| NZ_FIRU01000005.1 | 100 | 801 | 0 | 0 | 30722 | 29922 |
| NZ_FIRT01000002.1 | 100 | 801 | 0 | 0 | 349622 | 350422 |
| NZ_FIQZ01000001.1 | 100 | 801 | 0 | 0 | 30270 | 29470 |
| NZ_FIPQ01000002.1 | 100 | 801 | 0 | 0 | 343962 | 344762 |
| NZ_FIPD01000002.1 | 100 | 801 | 0 | 0 | 30061 | 29261 |
| NZ_FIFC01000002.1 | 100 | 801 | 0 | 0 | 30035 | 29235 |
| NZ_FIFB01000002.1 | 100 | 801 | 0 | 0 | 30026 | 29226 |
| NZ_FIND01000002.1 | 100 | 801 | 0 | 0 | 410384 | 411184 |
| NZ_FIMX01000002.1 | 100 | 801 | 0 | 0 | 270217 | 271017 |
| NZ_FIJI01000001.1 | 100 | 801 | 0 | 0 | 341186 | 341986 |
| NZ_FISZ01000001.1 | 100 | 801 | 0 | 0 | 30061 | 29261 |
| NZ_FIMZ01000002.1 | 100 | 801 | 0 | 0 | 271114 | 271914 |
| NZ_FIPZ01000001.1 | 100 | 801 | 0 | 0 | 30275 | 29475 |
| NZ_FIPI01000001.1 | 100 | 801 | 0 | 0 | 338313 | 339113 |
| NZ_FIPE01000006.1 | 100 | 801 | 0 | 0 | 30047 | 29247 |
| NZ_FINK01000001.1 | 100 | 801 | 0 | 0 | 30061 | 29261 |
| NZ_FITH01000005.1 | 100 | 801 | 0 | 0 | 35454 | 34654 |
| NZ_FIRA01000001.1 | 100 | 801 | 0 | 0 | 354669 | 355469 |
| NZ_FIPY01000001.1 | 100 | 801 | 0 | 0 | 343967 | 344767 |
| NZ_FIPU01000002.1 | 100 | 801 | 0 | 0 | 270891 | 271691 |
| NZ_FINX01000002.1 | 100 | 801 | 0 | 0 | 30060 | 29260 |
| NZ_FINW01000003.1 | 100 | 801 | 0 | 0 | 30049 | 29249 |
| NZ_FIMC01000006.1 | 100 | 801 | 0 | 0 | 83475 | 84275 |
| NZ_FILE01000001.1 | 100 | 801 | 0 | 0 | 259920 | 260720 |
| NZ_FIQT01000002.1 | 100 | 801 | 0 | 0 | 271130 | 271930 |
| NZ_FIQC01000007.1 | 100 | 801 | 0 | 0 | 30048 | 29248 |
| NZ_FIQE01000002.1 | 100 | 801 | 0 | 0 | 338324 | 339124 |
| NZ_FIOY01000008.1 | 100 | 801 | 0 | 0 | 30262 | 29462 |
| NZ_FIOX01000002.1 | 100 | 801 | 0 | 0 | 30061 | 29261 |
| NZ_FIRQ01000001.1 | 100 | 801 | 0 | 0 | 271118 | 271918 |
| NZ_FIRD01000001.1 | 100 | 801 | 0 | 0 | 349610 | 350410 |
| NZ_FIOW01000001.1 | 100 | 801 | 0 | 0 | 30054 | 29254 |
| NZ_FIOT01000002.1 | 100 | 801 | 0 | 0 | 30061 | 29261 |
| NZ_FIOL01000001.1 | 100 | 801 | 0 | 0 | 338311 | 339111 |
| NZ_FIKV01000002.1 | 100 | 801 | 0 | 0 | 259925 | 260725 |
| NZ_FIGI01000002.1 | 100 | 801 | 0 | 0 | 258686 | 259486 |
| NZ_FILN01000001.1 | 100 | 801 | 0 | 0 | 286531 | 287331 |
| NZ_FIQW01000001.1 | 100 | 801 | 0 | 0 | 349621 | 350421 |
| NZ_FIKR01000002.1 | 100 | 801 | 0 | 0 | 271031 | 271831 |
| NZ_FISY01000001.1 | 100 | 801 | 0 | 0 | 30061 | 29261 |
| NZ_FIRR01000001.1 | 100 | 801 | 0 | 0 | 349621 | 350421 |
| NZ_FIQF01000001.1 | 100 | 801 | 0 | 0 | 349696 | 350496 |
| NZ_FIOV01000002.1 | 100 | 801 | 0 | 0 | 271302 | 272102 |
| NZ_FILV01000002.1 | 100 | 801 | 0 | 0 | 276354 | 277154 |
| NZ_FIKQ01000003.1 | 100 | 801 | 0 | 0 | 271034 | 271834 |
| NZ_FIFV01000001.1 | 100 | 801 | 0 | 0 | 271224 | 272024 |
| NZ_FIOQ01000001.1 | 100 | 801 | 0 | 0 | 30061 | 29261 |
| NZ_FIMO01000001.1 | 100 | 801 | 0 | 0 | 343970 | 344770 |
| NZ_FILW01000001.1 | 100 | 801 | 0 | 0 | 271158 | 271958 |
| NZ_FILP01000002.1 | 100 | 801 | 0 | 0 | 270766 | 271566 |
| NZ_FIKY01000006.1 | 100 | 801 | 0 | 0 | 114998 | 115798 |
| NZ_FIJY01000002.1 | 100 | 801 | 0 | 0 | 329956 | 330756 |
| NZ_FIST01000004.1 | 100 | 801 | 0 | 0 | 154001 | 154801 |
| NZ_FISP01000001.1 | 100 | 801 | 0 | 0 | 30053 | 29253 |
| NZ_FIRI01000002.1 | 100 | 801 | 0 | 0 | 338308 | 339108 |
| NZ_FINQ01000001.1 | 100 | 801 | 0 | 0 | 349669 | 350469 |
| NZ_FIMY01000001.1 | 100 | 801 | 0 | 0 | 338297 | 339097 |
| NZ_FIMU01000002.1 | 100 | 801 | 0 | 0 | 259976 | 260776 |
| NZ_FIMT01000001.1 | 100 | 801 | 0 | 0 | 343899 | 344699 |
| NZ_FIMI01000002.1 | 100 | 801 | 0 | 0 | 30053 | 29253 |
| NZ_FIKB01000001.1 | 100 | 801 | 0 | 0 | 341744 | 342544 |
| NZ_FISX01000005.1 | 100 | 801 | 0 | 0 | 30062 | 29262 |
| NZ_FISM01000001.1 | 100 | 801 | 0 | 0 | 349702 | 350502 |
| NZ_FISL01000002.1 | 100 | 801 | 0 | 0 | 271420 | 272220 |
| NZ_FIMN01000002.1 | 100 | 801 | 0 | 0 | 30062 | 29262 |
| NZ_FIJF01000003.1 | 100 | 801 | 0 | 0 | 258676 | 259476 |
| NZ_FIJC01000002.1 | 100 | 801 | 0 | 0 | 271158 | 271958 |
| NZ_FISR01000001.1 | 100 | 801 | 0 | 0 | 349704 | 350504 |
| NZ_FIML01000002.1 | 100 | 801 | 0 | 0 | 271249 | 272049 |
| NZ_FISU01000001.1 | 100 | 801 | 0 | 0 | 30062 | 29262 |
| NZ_FISN01000002.1 | 100 | 801 | 0 | 0 | 259755 | 260555 |
| NZ_FIRB01000001.1 | 100 | 801 | 0 | 0 | 30060 | 29260 |
| NZ_FIQX01000001.1 | 100 | 801 | 0 | 0 | 338043 | 338843 |
| NZ_FINB01000001.1 | 100 | 801 | 0 | 0 | 30776 | 29976 |
| NZ_FINA01000001.1 | 100 | 801 | 0 | 0 | 30062 | 29262 |
| NZ_FIMS01000001.1 | 100 | 801 | 0 | 0 | 30054 | 29254 |
| NZ_FIMM01000001.1 | 100 | 801 | 0 | 0 | 30062 | 29262 |
| NZ_CZGR01000020.1 | 100 | 801 | 0 | 0 | 69766 | 70566 |
| NZ_CZGI01000031.1 | 100 | 801 | 0 | 0 | 271522 | 272322 |
| NZ_CZEQ01000036.1 | 100 | 801 | 0 | 0 | 271226 | 272026 |
| NZ_CZET01000039.1 | 100 | 801 | 0 | 0 | 182070 | 182870 |
| NZ_CZGU01000033.1 | 100 | 801 | 0 | 0 | 259949 | 260749 |
| NZ_CZGM01000034.1 | 100 | 801 | 0 | 0 | 271225 | 272025 |
| NZ_CZDT01000033.1 | 100 | 801 | 0 | 0 | 271150 | 271950 |
| NZ_CZDM01000013.1 | 100 | 801 | 0 | 0 | 30289 | 29489 |
| NZ_CZDX01000003.1 | 100 | 801 | 0 | 0 | 7781 | 8581 |
| NZ_CZFP01000010.1 | 100 | 801 | 0 | 0 | 34753 | 33953 |
| NZ_CZDQ01000035.1 | 100 | 801 | 0 | 0 | 271225 | 272025 |
| NZ_CZDV01000035.1 | 100 | 801 | 0 | 0 | 259945 | 260745 |
| NZ_CZEU01000038.1 | 100 | 801 | 0 | 0 | 329390 | 330190 |
| NZ_CZGZ01000040.1 | 100 | 801 | 0 | 0 | 271553 | 272353 |
| NZ_CZDK01000037.1 | 100 | 801 | 0 | 0 | 269425 | 270225 |
| NZ_CZDR01000036.1 | 100 | 801 | 0 | 0 | 30289 | 29489 |
| NZ_CZFK01000013.1 | 100 | 801 | 0 | 0 | 34753 | 33953 |
| NZ_CZFA01000034.1 | 100 | 801 | 0 | 0 | 271527 | 272327 |
| NZ_CZFB01000032.1 | 100 | 801 | 0 | 0 | 271225 | 272025 |
| NZ_CZEI01000035.1 | 100 | 801 | 0 | 0 | 30289 | 29489 |
| NZ_CZDL01000036.1 | 100 | 801 | 0 | 0 | 270551 | 271351 |
| NZ_CZFL01000034.1 | 100 | 801 | 0 | 0 | 30289 | 29489 |
| NZ_CZHD01000039.1 | 100 | 801 | 0 | 0 | 271523 | 272323 |
| NZ_CZEG01000034.1 | 100 | 801 | 0 | 0 | 271493 | 272293 |
| NZ_CZEA01000038.1 | 100 | 801 | 0 | 0 | 259973 | 260773 |
| NZ_CZHC01000044.1 | 100 | 801 | 0 | 0 | 271552 | 272352 |
| NZ_CZFU01000041.1 | 100 | 801 | 0 | 0 | 335363 | 336163 |
| NZ_CZFH01000003.1 | 100 | 801 | 0 | 0 | 30034 | 29234 |
| NZ_CZGX01000046.1 | 100 | 801 | 0 | 0 | 30289 | 29489 |
| NZ_CZGA01000034.1 | 100 | 801 | 0 | 0 | 259946 | 260746 |
| NZ_CZFO01000035.1 | 100 | 801 | 0 | 0 | 260244 | 261044 |
| NZ_CZFG01000036.1 | 100 | 801 | 0 | 0 | 271523 | 272323 |
| NZ_CZEO01000033.1 | 100 | 801 | 0 | 0 | 260247 | 261047 |
| NZ_CZEW01000034.1 | 100 | 801 | 0 | 0 | 260273 | 261073 |
| NZ_CZEM01000020.1 | 100 | 801 | 0 | 0 | 34753 | 33953 |
| NZ_CZER01000041.1 | 100 | 801 | 0 | 0 | 346209 | 347009 |
| NZ_CZDO01000033.1 | 100 | 801 | 0 | 0 | 259947 | 260747 |
| NZ_JASTSK010000001.1 | 100 | 801 | 0 | 0 | 30091 | 29291 |
| NZ_JASTSL010000001.1 | 100 | 801 | 0 | 0 | 30084 | 29284 |
| NZ_JASTSJ010000001.1 | 100 | 801 | 0 | 0 | 30085 | 29285 |
| NZ_JASTSI010000001.1 | 100 | 801 | 0 | 0 | 30084 | 29284 |
| NZ_JASTSH010000001.1 | 100 | 801 | 0 | 0 | 30084 | 29284 |
| NZ_JASTSG010000001.1 | 100 | 801 | 0 | 0 | 30084 | 29284 |
| NZ_JASTSD010000002.1 | 100 | 801 | 0 | 0 | 30084 | 29284 |
| NZ_JASTSF010000007.1 | 100 | 801 | 0 | 0 | 65428 | 66228 |
| NZ_JASTSE010000001.1 | 100 | 801 | 0 | 0 | 30355 | 29555 |
| NZ_JASTSC010000001.1 | 100 | 801 | 0 | 0 | 30085 | 29285 |
| NZ_JASTSB010000007.1 | 100 | 801 | 0 | 0 | 30305 | 29505 |
| NZ_JASTRZ010000001.1 | 100 | 801 | 0 | 0 | 30091 | 29291 |
| NZ_JASTSA010000005.1 | 100 | 801 | 0 | 0 | 30305 | 29505 |
| NZ_JASTRY010000002.1 | 100 | 801 | 0 | 0 | 30084 | 29284 |
| NZ_JASTRX010000001.1 | 100 | 801 | 0 | 0 | 30091 | 29291 |
| NZ_JASTRW010000001.1 | 100 | 801 | 0 | 0 | 30488 | 29688 |
| NZ_JASTRU010000001.1 | 100 | 801 | 0 | 0 | 30296 | 29496 |
| NZ_JASTRS010000001.1 | 100 | 801 | 0 | 0 | 30084 | 29284 |
| NZ_JASTRV010000001.1 | 100 | 801 | 0 | 0 | 30296 | 29496 |
| NZ_JASTRT010000001.1 | 100 | 801 | 0 | 0 | 30668 | 29868 |
| NZ_JASTRQ010000001.1 | 100 | 801 | 0 | 0 | 30090 | 29290 |
| NZ_JASTRP010000001.1 | 100 | 801 | 0 | 0 | 30084 | 29284 |
| NZ_JASTRR010000001.1 | 100 | 801 | 0 | 0 | 30296 | 29496 |
| NZ_JASTRO010000001.1 | 100 | 801 | 0 | 0 | 30084 | 29284 |
| NZ_JASTRN010000001.1 | 100 | 801 | 0 | 0 | 35736 | 34936 |
| NZ_JASTRM010000001.1 | 100 | 801 | 0 | 0 | 395135 | 395935 |
| NZ_JASTRL010000001.1 | 100 | 801 | 0 | 0 | 30091 | 29291 |
| NZ_JASTRK010000001.1 | 100 | 801 | 0 | 0 | 35736 | 34936 |
| NZ_JASTRJ010000001.1 | 100 | 801 | 0 | 0 | 30303 | 29503 |
| NZ_JASTRI010000001.1 | 100 | 801 | 0 | 0 | 30084 | 29284 |
| NZ_JASTRH010000001.1 | 100 | 801 | 0 | 0 | 30519 | 29719 |
| NZ_JASTRG010000001.1 | 100 | 801 | 0 | 0 | 30084 | 29284 |
| NZ_JASTRF010000001.1 | 100 | 801 | 0 | 0 | 30296 | 29496 |
| NZ_JASTRE010000001.1 | 100 | 801 | 0 | 0 | 331813 | 332613 |
| NZ_JASTRC010000001.1 | 100 | 801 | 0 | 0 | 30091 | 29291 |
| NZ_JASTRD010000001.1 | 100 | 801 | 0 | 0 | 30084 | 29284 |
| NZ_JASTRB010000006.1 | 100 | 801 | 0 | 0 | 66265 | 67065 |
| NZ_JASTRA010000001.1 | 100 | 801 | 0 | 0 | 30296 | 29496 |
| NZ_JASTQX010000001.1 | 100 | 801 | 0 | 0 | 30084 | 29284 |
| NZ_JASTQW010000001.1 | 100 | 801 | 0 | 0 | 30084 | 29284 |
| NZ_JASTQZ010000001.1 | 100 | 801 | 0 | 0 | 45871 | 45071 |
| NZ_JASTQY010000001.1 | 100 | 801 | 0 | 0 | 30084 | 29284 |
| NZ_JASTQU010000001.1 | 100 | 801 | 0 | 0 | 30084 | 29284 |
| NZ_JASTQR010000001.1 | 100 | 801 | 0 | 0 | 30091 | 29291 |
| NZ_JASTQV010000001.1 | 100 | 801 | 0 | 0 | 30296 | 29496 |
| NZ_JASTQT010000002.1 | 100 | 801 | 0 | 0 | 30296 | 29496 |
| NZ_JASTQS010000001.1 | 100 | 801 | 0 | 0 | 35523 | 34723 |
| NZ_JASTQP010000001.1 | 100 | 801 | 0 | 0 | 30091 | 29291 |
| NZ_JASTQQ010000002.1 | 100 | 801 | 0 | 0 | 271256 | 272056 |
| NZ_JASTQN010000001.1 | 100 | 801 | 0 | 0 | 410473 | 411273 |
| NZ_JASTQM010000002.1 | 100 | 801 | 0 | 0 | 30305 | 29505 |
| NZ_JASTQO010000017.1 | 100 | 801 | 0 | 0 | 30084 | 29284 |
| NZ_JASTQL010000001.1 | 100 | 801 | 0 | 0 | 30668 | 29868 |
| NZ_JASTQK010000001.1 | 100 | 801 | 0 | 0 | 30093 | 29293 |
| NZ_JASTQJ010000002.1 | 100 | 801 | 0 | 0 | 30084 | 29284 |
| NZ_JASTQH010000001.1 | 100 | 801 | 0 | 0 | 332138 | 332938 |
| NZ_JASTQI010000001.1 | 100 | 801 | 0 | 0 | 46033 | 45233 |
| NZ_JASTQG010000001.1 | 100 | 801 | 0 | 0 | 404843 | 405643 |
| NZ_JASTQF010000001.1 | 100 | 801 | 0 | 0 | 338921 | 339721 |
| NZ_JASTQD010000001.1 | 100 | 801 | 0 | 0 | 45821 | 45021 |
| NZ_JASTQB010000004.1 | 100 | 801 | 0 | 0 | 30084 | 29284 |
| NZ_JASTQC010000001.1 | 100 | 801 | 0 | 0 | 30296 | 29496 |
| NZ_JASTPY010000001.1 | 100 | 801 | 0 | 0 | 35736 | 34936 |
| NZ_JASTQA010000001.1 | 100 | 801 | 0 | 0 | 30084 | 29284 |
| NZ_JASTPZ010000001.1 | 100 | 801 | 0 | 0 | 30288 | 29488 |
| NZ_JASTPX010000001.1 | 100 | 801 | 0 | 0 | 30084 | 29284 |
| NZ_JASTPU010000001.1 | 100 | 801 | 0 | 0 | 30084 | 29284 |
| NZ_JASTPT010000002.1 | 100 | 801 | 0 | 0 | 30084 | 29284 |
| NZ_JASTPV010000002.1 | 100 | 801 | 0 | 0 | 271472 | 272272 |
| NZ_JASTPW010000002.1 | 100 | 801 | 0 | 0 | 30084 | 29284 |
| NZ_JASTPS010000002.1 | 100 | 801 | 0 | 0 | 271252 | 272052 |
| NZ_JASTPR010000006.1 | 100 | 801 | 0 | 0 | 35628 | 34828 |
| NZ_JBCLWR010000011.1 | 100 | 801 | 0 | 0 | 51310 | 52110 |
| NZ_JAVIGK010000004.1 | 100 | 801 | 0 | 0 | 69766 | 70566 |
| NZ_JAVIGJ010000001.1 | 100 | 801 | 0 | 0 | 331965 | 332765 |
| NZ_JAVIGH010000004.1 | 100 | 801 | 0 | 0 | 69791 | 70591 |
| NZ_JAVIGG010000005.1 | 100 | 801 | 0 | 0 | 69791 | 70591 |
| NZ_JAVIGM010000001.1 | 100 | 801 | 0 | 0 | 331710 | 332510 |
| NZ_JAVIGL010000001.1 | 100 | 801 | 0 | 0 | 331730 | 332530 |
| NZ_JAVIGC010000005.1 | 100 | 801 | 0 | 0 | 15100 | 14300 |
| NZ_JAVIGE010000005.1 | 100 | 801 | 0 | 0 | 15080 | 14280 |
| NZ_JAVIGD010000005.1 | 100 | 801 | 0 | 0 | 69834 | 70634 |
| NZ_JAVIGF010000005.1 | 100 | 801 | 0 | 0 | 15080 | 14280 |
| NZ_CP141904.1 | 100 | 801 | 0 | 0 | 1000123 | 999323 |
| NZ_CP139881.1 | 100 | 801 | 0 | 0 | 1860283 | 1861083 |
| NZ_CP139880.1 | 100 | 801 | 0 | 0 | 209710 | 208910 |
| NZ_CP139879.1 | 100 | 801 | 0 | 0 | 209490 | 208690 |
| NZ_JAXKWL010000001.1 | 100 | 801 | 0 | 0 | 1038476 | 1039276 |
| NZ_DASGDK010000002.1 | 100 | 801 | 0 | 0 | 34821 | 34021 |
| NZ_CP139163.1 | 100 | 801 | 0 | 0 | 252140 | 252940 |
| NZ_JAWWZM010000001.1 | 100 | 801 | 0 | 0 | 259947 | 260747 |
| NZ_JAWWZJ010000001.1 | 100 | 801 | 0 | 0 | 332781 | 333581 |
| NZ_JAWWZF010000004.1 | 100 | 801 | 0 | 0 | 169764 | 170564 |
| NZ_JAUTGQ010000003.1 | 100 | 801 | 0 | 0 | 30068 | 29268 |
| NZ_JAUTHY010000018.1 | 100 | 801 | 0 | 0 | 30067 | 29267 |
| NZ_JAUTHX010000001.1 | 100 | 801 | 0 | 0 | 337223 | 338023 |
| NZ_JAUTHL010000008.1 | 100 | 801 | 0 | 0 | 30044 | 29244 |
| NZ_JAUTHJ010000016.1 | 100 | 801 | 0 | 0 | 10386 | 11186 |
| NZ_JAUTHF010000022.1 | 100 | 801 | 0 | 0 | 8005 | 7205 |
| NZ_JAUTHE010000018.1 | 100 | 801 | 0 | 0 | 30118 | 29318 |
| NZ_JAUTHD010000008.1 | 100 | 801 | 0 | 0 | 46789 | 47589 |
| NZ_JAUTGV010000002.1 | 100 | 801 | 0 | 0 | 259866 | 260666 |
| NZ_JAUTGS010000005.1 | 100 | 801 | 0 | 0 | 45805 | 45005 |
| NZ_JAUTGT010000005.1 | 100 | 801 | 0 | 0 | 127008 | 127808 |
| NZ_JAUTGP010000001.1 | 100 | 801 | 0 | 0 | 30068 | 29268 |
| NZ_JAUTGN010000002.1 | 100 | 801 | 0 | 0 | 30068 | 29268 |
| NZ_JAUTFJ010000002.1 | 100 | 801 | 0 | 0 | 30068 | 29268 |
| NZ_JAUTFH010000004.1 | 100 | 801 | 0 | 0 | 66207 | 67007 |
| NZ_JAUTFF010000001.1 | 100 | 801 | 0 | 0 | 259951 | 260751 |
| NZ_JAUTFC010000001.1 | 100 | 801 | 0 | 0 | 45735 | 44935 |
| NZ_JAUTFE010000002.1 | 100 | 801 | 0 | 0 | 35613 | 34813 |
| NZ_JAUTFD010000002.1 | 100 | 801 | 0 | 0 | 260019 | 260819 |
| NZ_JAUTFB010000002.1 | 100 | 801 | 0 | 0 | 35613 | 34813 |
| NZ_JAUTEV010000002.1 | 100 | 801 | 0 | 0 | 35613 | 34813 |
| NZ_JAUTEZ010000002.1 | 100 | 801 | 0 | 0 | 35613 | 34813 |
| NZ_JAUTEX010000002.1 | 100 | 801 | 0 | 0 | 259569 | 260369 |
| NZ_JAUTFA010000010.1 | 100 | 801 | 0 | 0 | 30068 | 29268 |
| NZ_JAUTEY010000002.1 | 100 | 801 | 0 | 0 | 259569 | 260369 |
| NZ_JAUTEU010000002.1 | 100 | 801 | 0 | 0 | 30068 | 29268 |
| NZ_JAUTES010000002.1 | 100 | 801 | 0 | 0 | 259741 | 260541 |
| NZ_JAUTER010000006.1 | 100 | 801 | 0 | 0 | 64010 | 64810 |
| NZ_JAWQLT010000001.1 | 100 | 801 | 0 | 0 | 30288 | 29488 |
| NZ_CP134477.1 | 100 | 801 | 0 | 0 | 1008134 | 1007334 |
| NZ_JAVMBL010000006.1 | 100 | 801 | 0 | 0 | 95913 | 96713 |
| NZ_JAVMBI010000002.1 | 100 | 801 | 0 | 0 | 30084 | 29284 |
| NZ_JAVMBJ010000042.1 | 100 | 801 | 0 | 0 | 2438 | 3238 |
| NZ_JAVMBM010000003.1 | 100 | 801 | 0 | 0 | 35419 | 34619 |
| NZ_JAVMBN010000006.1 | 100 | 801 | 0 | 0 | 91464 | 92264 |
| NZ_JARATX010000009.1 | 100 | 801 | 0 | 0 | 30456 | 29656 |
| NZ_JARATW010000002.1 | 100 | 801 | 0 | 0 | 30460 | 29660 |
| NZ_JARATQ010000001.1 | 100 | 801 | 0 | 0 | 321290 | 322090 |
| NZ_JARATU010000002.1 | 100 | 801 | 0 | 0 | 271303 | 272103 |
| NZ_JARATT010000001.1 | 100 | 801 | 0 | 0 | 30456 | 29656 |
| NZ_JARATP010000002.1 | 100 | 801 | 0 | 0 | 271143 | 271943 |
| NZ_JARATO010000002.1 | 100 | 801 | 0 | 0 | 271420 | 272220 |
| NZ_JARATL010000006.1 | 100 | 801 | 0 | 0 | 90748 | 91548 |
| NZ_JARATM010000002.1 | 100 | 801 | 0 | 0 | 271532 | 272332 |
| NZ_JARATK010000002.1 | 100 | 801 | 0 | 0 | 30456 | 29656 |
| NZ_JARATJ010000006.1 | 100 | 801 | 0 | 0 | 67399 | 68199 |
| NZ_JARATH010000003.1 | 100 | 801 | 0 | 0 | 182121 | 182921 |
| NZ_JARATF010000010.1 | 100 | 801 | 0 | 0 | 51310 | 52110 |
| NZ_JARATG010000017.1 | 100 | 801 | 0 | 0 | 19050 | 19850 |
| NZ_JARATI010000005.1 | 100 | 801 | 0 | 0 | 30091 | 29291 |
| NZ_JARATC010000004.1 | 100 | 801 | 0 | 0 | 30084 | 29284 |
| NZ_JARATB010000004.1 | 100 | 801 | 0 | 0 | 67399 | 68199 |
| NZ_JARASZ010000002.1 | 100 | 801 | 0 | 0 | 182081 | 182881 |
| NZ_JARASY010000011.1 | 100 | 801 | 0 | 0 | 51310 | 52110 |
| NZ_JARASX010000002.1 | 100 | 801 | 0 | 0 | 258862 | 259662 |
| NZ_JARASR010000002.1 | 100 | 801 | 0 | 0 | 30456 | 29656 |
| NZ_JARASV010000003.1 | 100 | 801 | 0 | 0 | 30305 | 29505 |
| NZ_JARASU010000002.1 | 100 | 801 | 0 | 0 | 271201 | 272001 |
| NZ_JARAST010000011.1 | 100 | 801 | 0 | 0 | 30456 | 29656 |
| NZ_JARASP010000001.1 | 100 | 801 | 0 | 0 | 328854 | 329654 |
| NZ_JARASQ010000002.1 | 100 | 801 | 0 | 0 | 30019 | 29219 |
| NZ_JARASO010000001.1 | 100 | 801 | 0 | 0 | 339892 | 340692 |
| NZ_JARASN010000001.1 | 100 | 801 | 0 | 0 | 30019 | 29219 |
| NZ_JARASL010000026.1 | 100 | 801 | 0 | 0 | 4597 | 5397 |
| NZ_JARASM010000002.1 | 100 | 801 | 0 | 0 | 30019 | 29219 |
| NZ_JARASK010000002.1 | 100 | 801 | 0 | 0 | 30091 | 29291 |
| NZ_JAKTCQ010000002.1 | 100 | 801 | 0 | 0 | 259867 | 260667 |
| NZ_JAKTCR010000002.1 | 100 | 801 | 0 | 0 | 271241 | 272041 |
| NZ_JAKTCS010000006.1 | 100 | 801 | 0 | 0 | 96336 | 97136 |
| NZ_JAKTCT010000002.1 | 100 | 801 | 0 | 0 | 260239 | 261039 |
| NZ_JAKTCU010000002.1 | 100 | 801 | 0 | 0 | 258151 | 258951 |
| NZ_JAKTCV010000001.1 | 100 | 801 | 0 | 0 | 30034 | 29234 |
| NZ_JAKTCW010000001.1 | 100 | 801 | 0 | 0 | 384045 | 384845 |
| NZ_JAKTCX010000002.1 | 100 | 801 | 0 | 0 | 259976 | 260776 |
| NZ_JAKTCY010000001.1 | 100 | 801 | 0 | 0 | 338422 | 339222 |
| NZ_JAKTCZ010000001.1 | 100 | 801 | 0 | 0 | 112993 | 112193 |
| NZ_JAKTDA010000001.1 | 100 | 801 | 0 | 0 | 35370 | 34570 |
| NZ_JAKTDB010000001.1 | 100 | 801 | 0 | 0 | 338272 | 339072 |
| NZ_JAKTDC010000006.1 | 100 | 801 | 0 | 0 | 84012 | 84812 |
| NZ_JAKTDE010000002.1 | 100 | 801 | 0 | 0 | 259959 | 260759 |
| NZ_JAKTDF010000006.1 | 100 | 801 | 0 | 0 | 94602 | 95402 |
| NZ_JAKTDG010000002.1 | 100 | 801 | 0 | 0 | 259959 | 260759 |
| NZ_JAKTDH010000002.1 | 100 | 801 | 0 | 0 | 271238 | 272038 |
| NZ_JAKTDI010000006.1 | 100 | 801 | 0 | 0 | 83634 | 84434 |
| NZ_JAKTDK010000001.1 | 100 | 801 | 0 | 0 | 354049 | 354849 |
| NZ_JAKTDJ010000002.1 | 100 | 801 | 0 | 0 | 30278 | 29478 |
| NZ_JAKTDL010000006.1 | 100 | 801 | 0 | 0 | 94662 | 95462 |
| NZ_JAKTDM010000001.1 | 100 | 801 | 0 | 0 | 30041 | 29241 |
| NZ_JAKTDO010000001.1 | 100 | 801 | 0 | 0 | 30246 | 29446 |
| NZ_JAKTDN010000001.1 | 100 | 801 | 0 | 0 | 30034 | 29234 |
| NZ_JAKTDQ010000001.1 | 100 | 801 | 0 | 0 | 30253 | 29453 |
| NZ_JAKTDP010000001.1 | 100 | 801 | 0 | 0 | 30034 | 29234 |
| NZ_JAKTDR010000001.1 | 100 | 801 | 0 | 0 | 338312 | 339112 |
| NZ_JAKTDS010000002.1 | 100 | 801 | 0 | 0 | 344065 | 344865 |
| NZ_JAKTDU010000001.1 | 100 | 801 | 0 | 0 | 349586 | 350386 |
| NZ_JAKTDT010000001.1 | 100 | 801 | 0 | 0 | 338269 | 339069 |
| NZ_JAKTDV010000001.1 | 100 | 801 | 0 | 0 | 349588 | 350388 |
| NZ_JAKTDW010000001.1 | 100 | 801 | 0 | 0 | 338422 | 339222 |
| NZ_JAKTDX010000002.1 | 100 | 801 | 0 | 0 | 271238 | 272038 |
| NZ_JAKTDY010000002.1 | 100 | 801 | 0 | 0 | 30041 | 29241 |
| NZ_JAKTEA010000001.1 | 100 | 801 | 0 | 0 | 30034 | 29234 |
| NZ_JAKTDZ010000002.1 | 100 | 801 | 0 | 0 | 30041 | 29241 |
| NZ_JAKTEC010000001.1 | 100 | 801 | 0 | 0 | 30034 | 29234 |
| NZ_JAKTEB010000002.1 | 100 | 801 | 0 | 0 | 271238 | 272038 |
| NZ_JAKTED010000006.1 | 100 | 801 | 0 | 0 | 82852 | 83652 |
| NZ_JAKTEE010000001.1 | 100 | 801 | 0 | 0 | 30034 | 29234 |
| NZ_JAKTEF010000002.1 | 100 | 801 | 0 | 0 | 30041 | 29241 |
| NZ_JAKTEG010000002.1 | 100 | 801 | 0 | 0 | 30041 | 29241 |
| NZ_JAKTEI010000001.1 | 100 | 801 | 0 | 0 | 30041 | 29241 |
| NZ_JAKTEH010000001.1 | 100 | 801 | 0 | 0 | 308745 | 309545 |
| NZ_JAKTEJ010000002.1 | 100 | 801 | 0 | 0 | 266366 | 267166 |
| NZ_JAKTEK010000002.1 | 100 | 801 | 0 | 0 | 30041 | 29241 |
| NZ_JAKTEL010000002.1 | 100 | 801 | 0 | 0 | 30034 | 29234 |
| NZ_JAKTEM010000002.1 | 100 | 801 | 0 | 0 | 259869 | 260669 |
| NZ_JAKTEN010000002.1 | 100 | 801 | 0 | 0 | 30246 | 29446 |
| NZ_JAKTEO010000002.1 | 100 | 801 | 0 | 0 | 259960 | 260760 |
| NZ_JAKTEP010000002.1 | 100 | 801 | 0 | 0 | 259960 | 260760 |
| NZ_JAKTEQ010000002.1 | 100 | 801 | 0 | 0 | 259960 | 260760 |
| NZ_JAKTER010000001.1 | 100 | 801 | 0 | 0 | 338290 | 339090 |
| NZ_JAKTET010000006.1 | 100 | 801 | 0 | 0 | 30041 | 29241 |
| NZ_JAKTEU010000001.1 | 100 | 801 | 0 | 0 | 30034 | 29234 |
| NZ_JAKTEV010000001.1 | 100 | 801 | 0 | 0 | 276959 | 277759 |
| NZ_JAKTEW010000001.1 | 100 | 801 | 0 | 0 | 276959 | 277759 |
| NZ_JAKTEY010000002.1 | 100 | 801 | 0 | 0 | 30041 | 29241 |
| NZ_JAKTEZ010000001.1 | 100 | 801 | 0 | 0 | 343589 | 344389 |
| NZ_JAKTFA010000002.1 | 100 | 801 | 0 | 0 | 258151 | 258951 |
| NZ_JAKTFB010000020.1 | 100 | 801 | 0 | 0 | 30034 | 29234 |
| NZ_JAKTFC010000007.1 | 100 | 801 | 0 | 0 | 68604 | 69404 |
| NZ_JAKTFD010000002.1 | 100 | 801 | 0 | 0 | 30041 | 29241 |
| NZ_JAKTFE010000002.1 | 100 | 801 | 0 | 0 | 271238 | 272038 |
| NZ_JAKTFG010000006.1 | 100 | 801 | 0 | 0 | 30041 | 29241 |
| NZ_JAKTFF010000001.1 | 100 | 801 | 0 | 0 | 348369 | 349169 |
| NZ_JAKTFI010000006.1 | 100 | 801 | 0 | 0 | 30041 | 29241 |
| NZ_JAKTFH010000002.1 | 100 | 801 | 0 | 0 | 259959 | 260759 |
| NZ_JAKTFJ010000007.1 | 100 | 801 | 0 | 0 | 106335 | 107135 |
| NZ_JAKTFK010000003.1 | 100 | 801 | 0 | 0 | 193397 | 194197 |
| NZ_JAKTFL010000002.1 | 100 | 801 | 0 | 0 | 35369 | 34569 |
| NZ_JAKTFM010000002.1 | 100 | 801 | 0 | 0 | 30041 | 29241 |
| NZ_JAKTFN010000001.1 | 100 | 801 | 0 | 0 | 35370 | 34570 |
| NZ_JAKTFO010000002.1 | 100 | 801 | 0 | 0 | 271236 | 272036 |
| NZ_JAKTFQ010000006.1 | 100 | 801 | 0 | 0 | 94662 | 95462 |
| NZ_JAKTFP010000001.1 | 100 | 801 | 0 | 0 | 259959 | 260759 |
| NZ_JAKTFR010000001.1 | 100 | 801 | 0 | 0 | 30034 | 29234 |
| NZ_JAKTFS010000001.1 | 100 | 801 | 0 | 0 | 35369 | 34569 |
| NZ_JAKTFU010000002.1 | 100 | 801 | 0 | 0 | 259938 | 260738 |
| NZ_JAKTFT010000002.1 | 100 | 801 | 0 | 0 | 265728 | 266528 |
| NZ_JAKTFV010000002.1 | 100 | 801 | 0 | 0 | 30041 | 29241 |
| NZ_JAKTFW010000002.1 | 100 | 801 | 0 | 0 | 271228 | 272028 |
| NZ_JAKTFX010000002.1 | 100 | 801 | 0 | 0 | 271228 | 272028 |
| NZ_JAKTFY010000001.1 | 100 | 801 | 0 | 0 | 36061 | 35261 |
| NZ_JAKTFZ010000001.1 | 100 | 801 | 0 | 0 | 30034 | 29234 |
| NZ_JAKTGA010000002.1 | 100 | 801 | 0 | 0 | 35369 | 34569 |
| NZ_JAKTGB010000001.1 | 100 | 801 | 0 | 0 | 330985 | 331785 |
| NZ_JAKTGD010000002.1 | 100 | 801 | 0 | 0 | 271238 | 272038 |
| NZ_JAKTGE010000001.1 | 100 | 801 | 0 | 0 | 259960 | 260760 |
| NZ_JAKTGF010000002.1 | 100 | 801 | 0 | 0 | 30062 | 29262 |
| NZ_JAKTGG010000001.1 | 100 | 801 | 0 | 0 | 270820 | 271620 |
| NZ_JAKTGH010000002.1 | 100 | 801 | 0 | 0 | 30041 | 29241 |
| NZ_JAKTGI010000002.1 | 100 | 801 | 0 | 0 | 30041 | 29241 |
| NZ_JAKTGJ010000001.1 | 100 | 801 | 0 | 0 | 355332 | 356132 |
| NZ_JAKTGK010000001.1 | 100 | 801 | 0 | 0 | 349579 | 350379 |
| NZ_JAKTGL010000006.1 | 100 | 801 | 0 | 0 | 30050 | 29250 |
| NZ_JAKTGN010000002.1 | 100 | 801 | 0 | 0 | 265712 | 266512 |
| NZ_JAKTGM010000002.1 | 100 | 801 | 0 | 0 | 30041 | 29241 |
| NZ_JAKTGO010000006.1 | 100 | 801 | 0 | 0 | 94643 | 95443 |
| NZ_JAKTGP010000006.1 | 100 | 801 | 0 | 0 | 94643 | 95443 |
| NZ_JAKTGQ010000006.1 | 100 | 801 | 0 | 0 | 94583 | 95383 |
| NZ_JAKTGR010000002.1 | 100 | 801 | 0 | 0 | 259958 | 260758 |
| NZ_JAKTGS010000001.1 | 100 | 801 | 0 | 0 | 30246 | 29446 |
| NZ_JAKTGT010000001.1 | 100 | 801 | 0 | 0 | 337340 | 338140 |
| NZ_JAKTGU010000006.1 | 100 | 801 | 0 | 0 | 30041 | 29241 |
| NZ_JAKTGV010000001.1 | 100 | 801 | 0 | 0 | 30034 | 29234 |
| NZ_JAKTGX010000001.1 | 100 | 801 | 0 | 0 | 327475 | 328275 |
| NZ_JAKTGW010000001.1 | 100 | 801 | 0 | 0 | 333340 | 334140 |
| NZ_JAKTGZ010000003.1 | 100 | 801 | 0 | 0 | 155989 | 156789 |
| NZ_JAKTGY010000001.1 | 100 | 801 | 0 | 0 | 30246 | 29446 |
| NZ_JAKTHA010000002.1 | 100 | 801 | 0 | 0 | 30041 | 29241 |
| NZ_JAKTHB010000001.1 | 100 | 801 | 0 | 0 | 354661 | 355461 |
| NZ_JAKTHC010000006.1 | 100 | 801 | 0 | 0 | 30041 | 29241 |
| NZ_JAKTHD010000002.1 | 100 | 801 | 0 | 0 | 271454 | 272254 |
| NZ_JAKTHE010000001.1 | 100 | 801 | 0 | 0 | 335734 | 336534 |
| NZ_JAKTHF010000002.1 | 100 | 801 | 0 | 0 | 30034 | 29234 |
| NZ_JAKTHG010000002.1 | 100 | 801 | 0 | 0 | 270687 | 271487 |
| NZ_JAKTHH010000001.1 | 100 | 801 | 0 | 0 | 30034 | 29234 |
| NZ_JAKTHI010000001.1 | 100 | 801 | 0 | 0 | 30034 | 29234 |
| NZ_JAKTHJ010000001.1 | 100 | 801 | 0 | 0 | 112671 | 111871 |
| NZ_JAKTHK010000001.1 | 100 | 801 | 0 | 0 | 30124 | 29324 |
| NZ_JAKTHL010000002.1 | 100 | 801 | 0 | 0 | 271238 | 272038 |
| NZ_JAKTHN010000002.1 | 100 | 801 | 0 | 0 | 30278 | 29478 |
| NZ_JAKTHO010000002.1 | 100 | 801 | 0 | 0 | 259974 | 260774 |
| NZ_JAKTHP010000002.1 | 100 | 801 | 0 | 0 | 30041 | 29241 |
| NZ_JAKTHQ010000002.1 | 100 | 801 | 0 | 0 | 259963 | 260763 |
| NZ_JAKTHR010000001.1 | 100 | 801 | 0 | 0 | 35370 | 34570 |
| NZ_JAKTHT010000001.1 | 100 | 801 | 0 | 0 | 30034 | 29234 |
| NZ_JAKTHS010000001.1 | 100 | 801 | 0 | 0 | 259959 | 260759 |
| NZ_JAKTHU010000006.1 | 100 | 801 | 0 | 0 | 30033 | 29233 |
| NZ_CP102748.1 | 100 | 801 | 0 | 0 | 1020672 | 1021472 |
| NZ_CP102746.1 | 100 | 801 | 0 | 0 | 937088 | 937888 |
| NZ_JARBFN010000001.1 | 100 | 801 | 0 | 0 | 30275 | 29475 |
| NZ_JARBFQ010000002.1 | 100 | 801 | 0 | 0 | 30084 | 29284 |
| NZ_JABMDA010000002.1 | 100 | 801 | 0 | 0 | 30084 | 29284 |
| NZ_CP109942.1 | 100 | 801 | 0 | 0 | 1030300 | 1031100 |
| NZ_CP109941.1 | 100 | 801 | 0 | 0 | 942784 | 943584 |
| NZ_CP102154.1 | 100 | 801 | 0 | 0 | 936445 | 937245 |
| NZ_JAMWEI010000001.1 | 100 | 801 | 0 | 0 | 347305 | 348105 |
| NZ_JAMWEN010000002.1 | 100 | 801 | 0 | 0 | 30084 | 29284 |
| NZ_JAMDJI010000001.1 | 100 | 801 | 0 | 0 | 30041 | 29241 |
| NZ_JAMDJH010000001.1 | 100 | 801 | 0 | 0 | 30034 | 29234 |
| NZ_JAMDJE010000002.1 | 100 | 801 | 0 | 0 | 271218 | 272018 |
| NZ_JAMDIZ010000002.1 | 100 | 801 | 0 | 0 | 30034 | 29234 |
| NZ_JAMDIY010000001.1 | 100 | 801 | 0 | 0 | 271207 | 272007 |
| NZ_JAMDIU010000005.1 | 100 | 801 | 0 | 0 | 77867 | 78667 |
| NZ_JAMDIO010000001.1 | 100 | 801 | 0 | 0 | 259939 | 260739 |
| NZ_JAMDIE010000001.1 | 100 | 801 | 0 | 0 | 258115 | 258915 |
| NZ_JAMDHW010000002.1 | 100 | 801 | 0 | 0 | 258115 | 258915 |
| NZ_JAASDZ010000007.1 | 100 | 801 | 0 | 0 | 343583 | 344383 |
| NZ_JALLAM010000002.1 | 100 | 801 | 0 | 0 | 47444 | 46644 |
| NZ_JALLAO010000004.1 | 100 | 801 | 0 | 0 | 30044 | 29244 |
| NZ_CP095463.1 | 100 | 801 | 0 | 0 | 1122866 | 1122066 |
| NZ_JALIDS010000002.1 | 100 | 801 | 0 | 0 | 29978 | 29178 |
| NZ_JALIDV010000002.1 | 100 | 801 | 0 | 0 | 270969 | 271769 |
| NZ_JALIDU010000002.1 | 100 | 801 | 0 | 0 | 29978 | 29178 |
| NZ_JALIDT010000002.1 | 100 | 801 | 0 | 0 | 260178 | 260978 |
| NZ_JALIDR010000002.1 | 100 | 801 | 0 | 0 | 29978 | 29178 |
| NZ_JAKVTI010000001.1 | 100 | 801 | 0 | 0 | 328037 | 328837 |
| NZ_JAKTCE010000001.1 | 100 | 801 | 0 | 0 | 271225 | 272025 |
| NZ_JAKTBU010000002.1 | 100 | 801 | 0 | 0 | 271218 | 272018 |
| NZ_JAKTBX010000001.1 | 100 | 801 | 0 | 0 | 341497 | 342297 |
| NZ_JAKTAK010000001.1 | 100 | 801 | 0 | 0 | 335755 | 336555 |
| NZ_JAKTAL010000001.1 | 100 | 801 | 0 | 0 | 112671 | 111871 |
| NZ_JAKTAN010000002.1 | 100 | 801 | 0 | 0 | 30034 | 29234 |
| NZ_JAKTAR010000002.1 | 100 | 801 | 0 | 0 | 259971 | 260771 |
| NZ_JAKTAS010000002.1 | 100 | 801 | 0 | 0 | 259971 | 260771 |
| NZ_JAKTAQ010000001.1 | 100 | 801 | 0 | 0 | 340984 | 341784 |
| NZ_JAKTAM010000001.1 | 100 | 801 | 0 | 0 | 30034 | 29234 |
| NZ_JAKTAT010000002.1 | 100 | 801 | 0 | 0 | 259971 | 260771 |
| NZ_JAKTBP010000002.1 | 100 | 801 | 0 | 0 | 258115 | 258915 |
| NZ_JAKTBQ010000002.1 | 100 | 801 | 0 | 0 | 258115 | 258915 |
| NZ_JAKTBY010000001.1 | 100 | 801 | 0 | 0 | 335744 | 336544 |
| NZ_JAKTBS010000001.1 | 100 | 801 | 0 | 0 | 30041 | 29241 |
| NZ_JAKTAH010000002.1 | 100 | 801 | 0 | 0 | 30041 | 29241 |
| NZ_JAKTBZ010000001.1 | 100 | 801 | 0 | 0 | 335744 | 336544 |
| NZ_JAKTCA010000001.1 | 100 | 801 | 0 | 0 | 341497 | 342297 |
| NZ_CP091422.1 | 100 | 801 | 0 | 0 | 862859 | 862059 |
| NZ_CP091423.1 | 100 | 801 | 0 | 0 | 1022553 | 1021753 |
| NZ_CP085088.1 | 100 | 801 | 0 | 0 | 907903 | 908703 |
| NZ_JAJBSM010000001.1 | 100 | 801 | 0 | 0 | 936430 | 937230 |
| NZ_CP082948.1 | 100 | 801 | 0 | 0 | 1051738 | 1052538 |
| NZ_CP082201.1 | 100 | 801 | 0 | 0 | 993035 | 993835 |
| NZ_CP082200.1 | 100 | 801 | 0 | 0 | 992483 | 993283 |
| NZ_CP082197.1 | 100 | 801 | 0 | 0 | 819185 | 818385 |
| NZ_CP082199.1 | 100 | 801 | 0 | 0 | 990603 | 991403 |
| NZ_CP082198.1 | 100 | 801 | 0 | 0 | 995043 | 995843 |
| NZ_JAIMDX010000001.1 | 100 | 801 | 0 | 0 | 328399 | 329199 |
| NZ_JAIMDR010000001.1 | 100 | 801 | 0 | 0 | 271263 | 272063 |
| NZ_CP078543.1 | 100 | 801 | 0 | 0 | 1100565 | 1101365 |
| NZ_PXOO01000003.1 | 100 | 801 | 0 | 0 | 96148 | 96948 |
| NZ_PXON01000002.1 | 100 | 801 | 0 | 0 | 331414 | 332214 |
| NZ_PXOP01000001.1 | 100 | 801 | 0 | 0 | 356271 | 357071 |
| NZ_PXOM01000002.1 | 100 | 801 | 0 | 0 | 331409 | 332209 |
| NZ_PXOL01000002.1 | 100 | 801 | 0 | 0 | 35440 | 34640 |
| NZ_PXOI01000002.1 | 100 | 801 | 0 | 0 | 30567 | 29767 |
| NZ_PXOJ01000002.1 | 100 | 801 | 0 | 0 | 126529 | 127329 |
| NZ_PXOK01000002.1 | 100 | 801 | 0 | 0 | 22301 | 21501 |
| NZ_VZMV01000002.1 | 100 | 801 | 0 | 0 | 30065 | 29265 |
| NZ_VZMW01000002.1 | 100 | 801 | 0 | 0 | 30065 | 29265 |
| NZ_VZNA01000010.1 | 100 | 801 | 0 | 0 | 30063 | 29263 |
| NZ_VZNI01000001.1 | 100 | 801 | 0 | 0 | 271242 | 272042 |
| NZ_VZNQ01000001.1 | 100 | 801 | 0 | 0 | 30065 | 29265 |
| NZ_VZNW01000002.1 | 100 | 801 | 0 | 0 | 30065 | 29265 |
| NZ_VZJB01000001.1 | 100 | 801 | 0 | 0 | 338314 | 339114 |
| NZ_VZJC01000001.1 | 100 | 801 | 0 | 0 | 30066 | 29266 |
| NZ_VZJA01000006.1 | 100 | 801 | 0 | 0 | 30270 | 29470 |
| NZ_VZJD01000001.1 | 100 | 801 | 0 | 0 | 30058 | 29258 |
| NZ_VZJH01000001.1 | 100 | 801 | 0 | 0 | 30270 | 29470 |
| NZ_VZJE01000001.1 | 100 | 801 | 0 | 0 | 271262 | 272062 |
| NZ_VZJF01000001.1 | 100 | 801 | 0 | 0 | 112855 | 112055 |
| NZ_VZJG01000001.1 | 100 | 801 | 0 | 0 | 421558 | 422358 |
| NZ_VZJI01000001.1 | 100 | 801 | 0 | 0 | 30065 | 29265 |
| NZ_VZJK01000001.1 | 100 | 801 | 0 | 0 | 403922 | 404722 |
| NZ_VZJM01000001.1 | 100 | 801 | 0 | 0 | 455770 | 456570 |
| NZ_VZJJ01000002.1 | 100 | 801 | 0 | 0 | 30080 | 29280 |
| NZ_VZJN01000001.1 | 100 | 801 | 0 | 0 | 421763 | 422563 |
| NZ_VZJL01000001.1 | 100 | 801 | 0 | 0 | 410488 | 411288 |
| NZ_VZJO01000002.1 | 100 | 801 | 0 | 0 | 30065 | 29265 |
| NZ_VZJR01000006.1 | 100 | 801 | 0 | 0 | 30058 | 29258 |
| NZ_VZJP01000001.1 | 100 | 801 | 0 | 0 | 456023 | 456823 |
| NZ_VZJQ01000001.1 | 100 | 801 | 0 | 0 | 30065 | 29265 |
| NZ_VZJT01000001.1 | 100 | 801 | 0 | 0 | 30058 | 29258 |
| NZ_VZJS01000001.1 | 100 | 801 | 0 | 0 | 30277 | 29477 |
| NZ_VZJU01000002.1 | 100 | 801 | 0 | 0 | 30058 | 29258 |
| NZ_VZJX01000002.1 | 100 | 801 | 0 | 0 | 30070 | 29270 |
| NZ_VZJW01000002.1 | 100 | 801 | 0 | 0 | 30058 | 29258 |
| NZ_VZJY01000002.1 | 100 | 801 | 0 | 0 | 30065 | 29265 |
| NZ_VZJV01000001.1 | 100 | 801 | 0 | 0 | 397726 | 398526 |
| NZ_VZJZ01000001.1 | 100 | 801 | 0 | 0 | 343472 | 344272 |
| NZ_VZKA01000002.1 | 100 | 801 | 0 | 0 | 30065 | 29265 |
| NZ_VZKC01000001.1 | 100 | 801 | 0 | 0 | 30270 | 29470 |
| NZ_VZKB01000002.1 | 100 | 801 | 0 | 0 | 30051 | 29251 |
| NZ_VZKD01000002.1 | 100 | 801 | 0 | 0 | 30065 | 29265 |
| NZ_VZKF01000001.1 | 100 | 801 | 0 | 0 | 332376 | 333176 |
| NZ_VZKH01000002.1 | 100 | 801 | 0 | 0 | 30065 | 29265 |
| NZ_VZKE01000001.1 | 100 | 801 | 0 | 0 | 421725 | 422525 |
| NZ_VZKG01000006.1 | 100 | 801 | 0 | 0 | 30058 | 29258 |
| NZ_VZKI01000006.1 | 100 | 801 | 0 | 0 | 26412 | 27212 |
| NZ_VZKL01000001.1 | 100 | 801 | 0 | 0 | 404894 | 405694 |
| NZ_VZKM01000006.1 | 100 | 801 | 0 | 0 | 30270 | 29470 |
| NZ_VZKK01000001.1 | 100 | 801 | 0 | 0 | 30058 | 29258 |
| NZ_VZKJ01000001.1 | 100 | 801 | 0 | 0 | 403808 | 404608 |
| NZ_VZKN01000005.1 | 100 | 801 | 0 | 0 | 124598 | 125398 |
| NZ_VZKO01000001.1 | 100 | 801 | 0 | 0 | 271263 | 272063 |
| NZ_VZKP01000001.1 | 100 | 801 | 0 | 0 | 259983 | 260783 |
| NZ_VZKQ01000001.1 | 100 | 801 | 0 | 0 | 410404 | 411204 |
| NZ_VZKS01000001.1 | 100 | 801 | 0 | 0 | 270943 | 271743 |
| NZ_VZKR01000001.1 | 100 | 801 | 0 | 0 | 338326 | 339126 |
| NZ_VZKU01000001.1 | 100 | 801 | 0 | 0 | 30058 | 29258 |
| NZ_VZKT01000002.1 | 100 | 801 | 0 | 0 | 29233 | 28433 |
| NZ_VZKV01000001.1 | 100 | 801 | 0 | 0 | 112695 | 111895 |
| NZ_VZKW01000002.1 | 100 | 801 | 0 | 0 | 267088 | 267888 |
| NZ_VZKY01000001.1 | 100 | 801 | 0 | 0 | 399529 | 400329 |
| NZ_VZKX01000001.1 | 100 | 801 | 0 | 0 | 30058 | 29258 |
| NZ_VZKZ01000001.1 | 100 | 801 | 0 | 0 | 30270 | 29470 |
| NZ_VZLB01000001.1 | 100 | 801 | 0 | 0 | 403865 | 404665 |
| NZ_VZLA01000001.1 | 100 | 801 | 0 | 0 | 407973 | 408773 |
| NZ_VZLD01000001.1 | 100 | 801 | 0 | 0 | 30058 | 29258 |
| NZ_VZLC01000001.1 | 100 | 801 | 0 | 0 | 432331 | 433131 |
| NZ_VZLE01000001.1 | 100 | 801 | 0 | 0 | 30058 | 29258 |
| NZ_VZLF01000001.1 | 100 | 801 | 0 | 0 | 331791 | 332591 |
| NZ_VZLH01000001.1 | 100 | 801 | 0 | 0 | 421052 | 421852 |
| NZ_VZLJ01000001.1 | 100 | 801 | 0 | 0 | 432330 | 433130 |
| NZ_VZLI01000002.1 | 100 | 801 | 0 | 0 | 30313 | 29513 |
| NZ_VZLK01000002.1 | 100 | 801 | 0 | 0 | 30065 | 29265 |
| NZ_VZLL01000001.1 | 100 | 801 | 0 | 0 | 30270 | 29470 |
| NZ_VZLM01000001.1 | 100 | 801 | 0 | 0 | 112855 | 112055 |
| NZ_VZLO01000002.1 | 100 | 801 | 0 | 0 | 271273 | 272073 |
| NZ_VZLN01000001.1 | 100 | 801 | 0 | 0 | 112855 | 112055 |
| NZ_VZLQ01000006.1 | 100 | 801 | 0 | 0 | 30065 | 29265 |
| NZ_VZLR01000002.1 | 100 | 801 | 0 | 0 | 193503 | 194303 |
| NZ_VZLP01000001.1 | 100 | 801 | 0 | 0 | 332341 | 333141 |
| NZ_VZLT01000001.1 | 100 | 801 | 0 | 0 | 406645 | 407445 |
| NZ_VZLS01000001.1 | 100 | 801 | 0 | 0 | 324580 | 325380 |
| NZ_VZLV01000001.1 | 100 | 801 | 0 | 0 | 30058 | 29258 |
| NZ_VZLU01000001.1 | 100 | 801 | 0 | 0 | 30058 | 29258 |
| NZ_VZLW01000001.1 | 100 | 801 | 0 | 0 | 410516 | 411316 |
| NZ_VZLX01000001.1 | 100 | 801 | 0 | 0 | 410506 | 411306 |
| NZ_VZLY01000001.1 | 100 | 801 | 0 | 0 | 30058 | 29258 |
| NZ_VZMA01000001.1 | 100 | 801 | 0 | 0 | 410371 | 411171 |
| NZ_VZLZ01000001.1 | 100 | 801 | 0 | 0 | 30058 | 29258 |
| NZ_VZMC01000007.1 | 100 | 801 | 0 | 0 | 24543 | 25343 |
| NZ_VZMB01000001.1 | 100 | 801 | 0 | 0 | 410516 | 411316 |
| NZ_VZMD01000001.1 | 100 | 801 | 0 | 0 | 30270 | 29470 |
| NZ_VZMF01000006.1 | 100 | 801 | 0 | 0 | 30058 | 29258 |
| NZ_VZME01000001.1 | 100 | 801 | 0 | 0 | 30058 | 29258 |
| NZ_VZMG01000001.1 | 100 | 801 | 0 | 0 | 406645 | 407445 |
| NZ_VZMH01000001.1 | 100 | 801 | 0 | 0 | 338060 | 338860 |
| NZ_VZMI01000001.1 | 100 | 801 | 0 | 0 | 30058 | 29258 |
| NZ_VZMJ01000001.1 | 100 | 801 | 0 | 0 | 271262 | 272062 |
| NZ_VZML01000001.1 | 100 | 801 | 0 | 0 | 332179 | 332979 |
| NZ_VZMM01000001.1 | 100 | 801 | 0 | 0 | 30270 | 29470 |
| NZ_VZMN01000005.1 | 100 | 801 | 0 | 0 | 35393 | 34593 |
| NZ_VZMO01000004.1 | 100 | 801 | 0 | 0 | 125098 | 125898 |
| NZ_VZMR01000001.1 | 100 | 801 | 0 | 0 | 30058 | 29258 |
| NZ_VZMT01000001.1 | 100 | 801 | 0 | 0 | 112855 | 112055 |
| NZ_VZMS01000001.1 | 100 | 801 | 0 | 0 | 112855 | 112055 |
| NZ_VZMU01000001.1 | 100 | 801 | 0 | 0 | 30058 | 29258 |
| NZ_JAGUCC010000001.1 | 100 | 801 | 0 | 0 | 29957 | 29157 |
| NZ_JAGUAB010000002.1 | 100 | 801 | 0 | 0 | 259894 | 260694 |
| NZ_JAGUAF010000001.1 | 100 | 801 | 0 | 0 | 276883 | 277683 |
| NZ_JAGUCB010000001.1 | 100 | 801 | 0 | 0 | 29957 | 29157 |
| NZ_JAGUBZ010000001.1 | 100 | 801 | 0 | 0 | 29957 | 29157 |
| NZ_JAGUCD010000002.1 | 100 | 801 | 0 | 0 | 259923 | 260723 |
| NZ_JAGUAT010000001.1 | 100 | 801 | 0 | 0 | 327829 | 328629 |
| NZ_JAGUAV010000001.1 | 100 | 801 | 0 | 0 | 112594 | 111794 |
| NZ_JAGUAR010000001.1 | 100 | 801 | 0 | 0 | 29957 | 29157 |
| NZ_JAGUAP010000002.1 | 100 | 801 | 0 | 0 | 271211 | 272011 |
| NZ_JAGUAO010000001.1 | 100 | 801 | 0 | 0 | 327419 | 328219 |
| NZ_JAGUAQ010000001.1 | 100 | 801 | 0 | 0 | 3709 | 2909 |
| NZ_JAGUCE010000001.1 | 100 | 801 | 0 | 0 | 29960 | 29160 |
| NZ_JAGUAC010000001.1 | 100 | 801 | 0 | 0 | 343185 | 343985 |
| NZ_JAGUBD010000001.1 | 100 | 801 | 0 | 0 | 29957 | 29157 |
| NZ_JAGUBC010000006.1 | 100 | 801 | 0 | 0 | 29957 | 29157 |
| NZ_JAGUBA010000001.1 | 100 | 801 | 0 | 0 | 360091 | 360891 |
| NZ_JAGUBB010000001.1 | 100 | 801 | 0 | 0 | 29957 | 29157 |
| NZ_JAGUAZ010000002.1 | 100 | 801 | 0 | 0 | 29956 | 29156 |
| NZ_JAGUAX010000001.1 | 100 | 801 | 0 | 0 | 112594 | 111794 |
| NZ_JAGUAY010000001.1 | 100 | 801 | 0 | 0 | 29957 | 29157 |
| NZ_JAGUAS010000009.1 | 100 | 801 | 0 | 0 | 29957 | 29157 |
| NZ_JAGUAW010000001.1 | 100 | 801 | 0 | 0 | 327829 | 328629 |
| NZ_JAGUAU010000005.1 | 100 | 801 | 0 | 0 | 112594 | 111794 |
| NZ_JAGUCA010000001.1 | 100 | 801 | 0 | 0 | 29957 | 29157 |
| NZ_JAGUBX010000001.1 | 100 | 801 | 0 | 0 | 29957 | 29157 |
| NZ_JAGUBY010000001.1 | 100 | 801 | 0 | 0 | 29957 | 29157 |
| NZ_JAGUBT010000001.1 | 100 | 801 | 0 | 0 | 327955 | 328755 |
| NZ_JAGUBV010000002.1 | 100 | 801 | 0 | 0 | 271196 | 271996 |
| NZ_JAGUBU010000001.1 | 100 | 801 | 0 | 0 | 29961 | 29161 |
| NZ_JAGUBS010000002.1 | 100 | 801 | 0 | 0 | 29957 | 29157 |
| NZ_JAGUBR010000001.1 | 100 | 801 | 0 | 0 | 29957 | 29157 |
| NZ_JAGUBQ010000001.1 | 100 | 801 | 0 | 0 | 112594 | 111794 |
| NZ_JAGUBM010000002.1 | 100 | 801 | 0 | 0 | 29957 | 29157 |
| NZ_JAGUBO010000001.1 | 100 | 801 | 0 | 0 | 30076 | 29276 |
| NZ_JAGUBN010000001.1 | 100 | 801 | 0 | 0 | 29957 | 29157 |
| NZ_JAGUBH010000001.1 | 100 | 801 | 0 | 0 | 29957 | 29157 |
| NZ_JAGUBG010000002.1 | 100 | 801 | 0 | 0 | 29957 | 29157 |
| NZ_JAGUBE010000001.1 | 100 | 801 | 0 | 0 | 346902 | 347702 |
| NZ_JAGJDV010000002.1 | 100 | 801 | 0 | 0 | 168027 | 168827 |
| NZ_WABV01000002.1 | 100 | 801 | 0 | 0 | 29957 | 29157 |
| NZ_WABX01000002.1 | 100 | 801 | 0 | 0 | 271239 | 272039 |
| NZ_WABY01000002.1 | 100 | 801 | 0 | 0 | 29957 | 29157 |
| NZ_WABW01000002.1 | 100 | 801 | 0 | 0 | 29957 | 29157 |
| NZ_WACB01000002.1 | 100 | 801 | 0 | 0 | 271234 | 272034 |
| NZ_WABZ01000002.1 | 100 | 801 | 0 | 0 | 271242 | 272042 |
| NZ_WACA01000002.1 | 100 | 801 | 0 | 0 | 29957 | 29157 |
| NZ_JAFEIS010000001.1 | 100 | 801 | 0 | 0 | 30084 | 29284 |
| NZ_WCJG01000001.1 | 100 | 801 | 0 | 0 | 29957 | 29157 |
| NZ_WCJF01000001.1 | 100 | 801 | 0 | 0 | 347293 | 348093 |
| NZ_WCJD01000001.1 | 100 | 801 | 0 | 0 | 324452 | 325252 |
| NZ_WCJC01000001.1 | 100 | 801 | 0 | 0 | 324302 | 325102 |
| NZ_WCJB01000001.1 | 100 | 801 | 0 | 0 | 29957 | 29157 |
| NZ_BCEL01000017.1 | 100 | 801 | 0 | 0 | 318763 | 319563 |
| NZ_BCEK01000025.1 | 100 | 801 | 0 | 0 | 30061 | 29261 |
| NZ_BCEI01000022.1 | 100 | 801 | 0 | 0 | 327780 | 328580 |
| NZ_BCDU01000012.1 | 100 | 801 | 0 | 0 | 322044 | 322844 |
| NZ_BCDR01000015.1 | 100 | 801 | 0 | 0 | 322196 | 322996 |
| NZ_BCDN01000016.1 | 100 | 801 | 0 | 0 | 271540 | 272340 |
| NZ_BCDM01000026.1 | 100 | 801 | 0 | 0 | 30242 | 29442 |
| NZ_BCDL01000015.1 | 100 | 801 | 0 | 0 | 30044 | 29244 |
| NZ_BCDK01000005.1 | 100 | 801 | 0 | 0 | 165273 | 166073 |
| NZ_BCDJ01000011.1 | 100 | 801 | 0 | 0 | 322202 | 323002 |
| NZ_BCDI01000007.1 | 100 | 801 | 0 | 0 | 30012 | 29212 |
| NZ_BCDH01000008.1 | 100 | 801 | 0 | 0 | 318744 | 319544 |
| NZ_BCDG01000018.1 | 100 | 801 | 0 | 0 | 30023 | 29223 |
| NZ_BCDE01000026.1 | 100 | 801 | 0 | 0 | 30031 | 29231 |
| NZ_BCDD01000024.1 | 100 | 801 | 0 | 0 | 30013 | 29213 |
| NZ_BCDC01000021.1 | 100 | 801 | 0 | 0 | 322057 | 322857 |
| NZ_BCDB01000019.1 | 100 | 801 | 0 | 0 | 30029 | 29229 |
| NZ_BCDA01000002.1 | 100 | 801 | 0 | 0 | 30017 | 29217 |
| NZ_BCCZ01000008.1 | 100 | 801 | 0 | 0 | 129199 | 129999 |
| NZ_BCCY01000027.1 | 100 | 801 | 0 | 0 | 271494 | 272294 |
| NZ_BCCW01000019.1 | 100 | 801 | 0 | 0 | 265077 | 265877 |
| NZ_BCCU01000016.1 | 100 | 801 | 0 | 0 | 30007 | 29207 |
| NZ_BCCS01000022.1 | 100 | 801 | 0 | 0 | 30023 | 29223 |
| NZ_BCCR01000018.1 | 100 | 801 | 0 | 0 | 277784 | 278584 |
| NZ_BCCQ01000003.1 | 100 | 801 | 0 | 0 | 30033 | 29233 |
| NZ_BCCP01000005.1 | 100 | 801 | 0 | 0 | 271453 | 272253 |
| NZ_BCCE01000011.1 | 100 | 801 | 0 | 0 | 30057 | 29257 |
| NZ_BCCB01000005.1 | 100 | 801 | 0 | 0 | 110796 | 111596 |
| NZ_BCBY01000009.1 | 100 | 801 | 0 | 0 | 30031 | 29231 |
| NZ_CP065431.1 | 100 | 801 | 0 | 0 | 1005728 | 1006528 |
| NZ_CP058742.1 | 100 | 801 | 0 | 0 | 937011 | 937811 |
| NZ_CP030022.1 | 100 | 801 | 0 | 0 | 1005355 | 1006155 |
| NZ_CP030017.1 | 100 | 801 | 0 | 0 | 1003557 | 1004357 |
| NZ_JABXEX010000001.1 | 100 | 801 | 0 | 0 | 29964 | 29164 |
| NZ_JABXEY010000002.1 | 100 | 801 | 0 | 0 | 72539 | 73339 |
| NZ_JABLFH010000001.1 | 100 | 801 | 0 | 0 | 30028 | 29228 |
| NZ_JABLFF010000001.1 | 100 | 801 | 0 | 0 | 30037 | 29237 |
| NZ_JABLFI010000003.1 | 100 | 801 | 0 | 0 | 171410 | 172210 |
| NZ_JABLFK010000005.1 | 100 | 801 | 0 | 0 | 114924 | 115724 |
| NZ_JABLFL010000006.1 | 100 | 801 | 0 | 0 | 83512 | 84312 |
| NZ_JABLFM010000004.1 | 100 | 801 | 0 | 0 | 136806 | 137606 |
| NZ_JABLFP010000001.1 | 100 | 801 | 0 | 0 | 30037 | 29237 |
| NZ_JABLFO010000006.1 | 100 | 801 | 0 | 0 | 117624 | 118424 |
| NZ_JABLFU010000005.1 | 100 | 801 | 0 | 0 | 117624 | 118424 |
| NZ_JABLFX010000004.1 | 100 | 801 | 0 | 0 | 148104 | 148904 |
| NZ_JABLFV010000001.1 | 100 | 801 | 0 | 0 | 102105 | 101305 |
| NZ_JABLGB010000003.1 | 100 | 801 | 0 | 0 | 30037 | 29237 |
| NZ_JABLFY010000003.1 | 100 | 801 | 0 | 0 | 30497 | 29697 |
| NZ_JABLGD010000004.1 | 100 | 801 | 0 | 0 | 30037 | 29237 |
| NZ_JABLGE010000004.1 | 100 | 801 | 0 | 0 | 30037 | 29237 |
| NZ_JABLGG010000001.1 | 100 | 801 | 0 | 0 | 239124 | 239924 |
| NZ_JABLGH010000004.1 | 100 | 801 | 0 | 0 | 30037 | 29237 |
| NZ_JABLGK010000005.1 | 100 | 801 | 0 | 0 | 83499 | 84299 |
| NZ_JABLGN010000005.1 | 100 | 801 | 0 | 0 | 30080 | 29280 |
| NZ_JABLGM010000002.1 | 100 | 801 | 0 | 0 | 271294 | 272094 |
| NZ_JABLGR010000006.1 | 100 | 801 | 0 | 0 | 30080 | 29280 |
| NZ_JABLHH010000001.1 | 100 | 801 | 0 | 0 | 333958 | 334758 |
| NZ_JABLHI010000001.1 | 100 | 801 | 0 | 0 | 341380 | 342180 |
| NZ_JABLHK010000005.1 | 100 | 801 | 0 | 0 | 148241 | 149041 |
| NZ_JABLHP010000005.1 | 100 | 801 | 0 | 0 | 136878 | 137678 |
| NZ_JABLHX010000001.1 | 100 | 801 | 0 | 0 | 343336 | 344136 |
| NZ_JABLHW010000001.1 | 100 | 801 | 0 | 0 | 271354 | 272154 |
| NZ_JABLHY010000003.1 | 100 | 801 | 0 | 0 | 271133 | 271933 |
| NZ_JABLIB010000001.1 | 100 | 801 | 0 | 0 | 271133 | 271933 |
| NZ_JABLIN010000001.1 | 100 | 801 | 0 | 0 | 331856 | 332656 |
| NZ_JABLIR010000001.1 | 100 | 801 | 0 | 0 | 322410 | 323210 |
| NZ_JABLFR010000005.1 | 100 | 801 | 0 | 0 | 115040 | 115840 |
| NZ_JABLIS010000007.1 | 100 | 801 | 0 | 0 | 112995 | 113795 |
| NZ_JABLFQ010000003.1 | 100 | 801 | 0 | 0 | 30037 | 29237 |
| NZ_JABLIZ010000003.1 | 100 | 801 | 0 | 0 | 271018 | 271818 |
| NZ_JABLJJ010000006.1 | 100 | 801 | 0 | 0 | 30087 | 29287 |
| NZ_JABLGV010000003.1 | 100 | 801 | 0 | 0 | 259939 | 260739 |
| NZ_JABLJL010000002.1 | 100 | 801 | 0 | 0 | 343196 | 343996 |
| NZ_JABLJN010000006.1 | 100 | 801 | 0 | 0 | 114974 | 115774 |
| NZ_JABLGS010000006.1 | 100 | 801 | 0 | 0 | 30080 | 29280 |
| NZ_JABLHE010000001.1 | 100 | 801 | 0 | 0 | 331656 | 332456 |
| NZ_JABLGW010000007.1 | 100 | 801 | 0 | 0 | 95173 | 95973 |
| NZ_JABLHA010000001.1 | 100 | 801 | 0 | 0 | 30080 | 29280 |
| NZ_JABLFC010000011.1 | 100 | 801 | 0 | 0 | 34738 | 35538 |
| NZ_JABLHC010000001.1 | 100 | 801 | 0 | 0 | 30078 | 29278 |
| NZ_JABLJB010000002.1 | 100 | 801 | 0 | 0 | 259768 | 260568 |
| NZ_JABLHB010000001.1 | 100 | 801 | 0 | 0 | 259939 | 260739 |
| NZ_JABLJX010000001.1 | 100 | 801 | 0 | 0 | 332094 | 332894 |
| NZ_JABLKD010000001.1 | 100 | 801 | 0 | 0 | 30080 | 29280 |
| NZ_JABLKA010000001.1 | 100 | 801 | 0 | 0 | 343360 | 344160 |
| NZ_JABLKB010000001.1 | 100 | 801 | 0 | 0 | 413381 | 414181 |
| NZ_JABLKH010000001.1 | 100 | 801 | 0 | 0 | 343166 | 343966 |
| NZ_JABLKG010000002.1 | 100 | 801 | 0 | 0 | 30080 | 29280 |
| NZ_JABLKI010000001.1 | 100 | 801 | 0 | 0 | 393936 | 394736 |
| NZ_JABLJT010000001.1 | 100 | 801 | 0 | 0 | 30081 | 29281 |
| NZ_JABLJU010000001.1 | 100 | 801 | 0 | 0 | 347055 | 347855 |
| NZ_JABLQA010000002.1 | 100 | 801 | 0 | 0 | 148138 | 148938 |
| NZ_JABLPY010000002.1 | 100 | 801 | 0 | 0 | 148046 | 148846 |
| NZ_JABLPZ010000001.1 | 100 | 801 | 0 | 0 | 259997 | 260797 |
| NZ_JABLQC010000002.1 | 100 | 801 | 0 | 0 | 259951 | 260751 |
| NZ_JABLPT010000001.1 | 100 | 801 | 0 | 0 | 338323 | 339123 |
| NZ_JABLQB010000002.1 | 100 | 801 | 0 | 0 | 30275 | 29475 |
| NZ_JABLQE010000001.1 | 100 | 801 | 0 | 0 | 343971 | 344771 |
| NZ_JABLQD010000001.1 | 100 | 801 | 0 | 0 | 215190 | 215990 |
| NZ_JABLQG010000001.1 | 100 | 801 | 0 | 0 | 30054 | 29254 |
| NZ_JABLQF010000001.1 | 100 | 801 | 0 | 0 | 271260 | 272060 |
| NZ_JABLQH010000001.1 | 100 | 801 | 0 | 0 | 30054 | 29254 |
| NZ_JABLQI010000001.1 | 100 | 801 | 0 | 0 | 259981 | 260781 |
| NZ_JABLPW010000002.1 | 100 | 801 | 0 | 0 | 269947 | 270747 |
| NZ_JABLQJ010000006.1 | 100 | 801 | 0 | 0 | 136858 | 137658 |
| NZ_JABLQK010000002.1 | 100 | 801 | 0 | 0 | 222794 | 223594 |
| NZ_JABLPV010000001.1 | 100 | 801 | 0 | 0 | 50425 | 49625 |
| NZ_JABLQL010000006.1 | 100 | 801 | 0 | 0 | 65207 | 66007 |
| NZ_JABLPU010000002.1 | 100 | 801 | 0 | 0 | 215191 | 215991 |
| NZ_JABLQM010000001.1 | 100 | 801 | 0 | 0 | 27028 | 26228 |
| NZ_JABLPX010000003.1 | 100 | 801 | 0 | 0 | 148716 | 149516 |
| NZ_JABLQQ010000002.1 | 100 | 801 | 0 | 0 | 270218 | 271018 |
| NZ_JABLQR010000001.1 | 100 | 801 | 0 | 0 | 215787 | 216587 |
| NZ_JABLPS010000001.1 | 100 | 801 | 0 | 0 | 30927 | 30127 |
| NZ_JABLQS010000007.1 | 100 | 801 | 0 | 0 | 93644 | 94444 |
| NZ_JABLQT010000001.1 | 100 | 801 | 0 | 0 | 338326 | 339126 |
| NZ_JABLQU010000002.1 | 100 | 801 | 0 | 0 | 30063 | 29263 |
| NZ_JABLQV010000004.1 | 100 | 801 | 0 | 0 | 148143 | 148943 |
| NZ_JABLQP010000001.1 | 100 | 801 | 0 | 0 | 338309 | 339109 |
| NZ_JABLQN010000003.1 | 100 | 801 | 0 | 0 | 136859 | 137659 |
| NZ_JABLQO010000002.1 | 100 | 801 | 0 | 0 | 221456 | 222256 |
| NZ_JABKSV010000001.1 | 100 | 801 | 0 | 0 | 332050 | 332850 |
| NZ_JABKSU010000001.1 | 100 | 801 | 0 | 0 | 332190 | 332990 |
| NZ_JABKTO010000002.1 | 100 | 801 | 0 | 0 | 259962 | 260762 |
| NZ_JABKTR010000001.1 | 100 | 801 | 0 | 0 | 259961 | 260761 |
| NZ_JABKTQ010000002.1 | 100 | 801 | 0 | 0 | 259975 | 260775 |
| NZ_JABKUV010000001.1 | 100 | 801 | 0 | 0 | 341271 | 342071 |
| NZ_JABKVC010000001.1 | 100 | 801 | 0 | 0 | 329766 | 330566 |
| NZ_JABKSR010000001.1 | 100 | 801 | 0 | 0 | 332065 | 332865 |
| NZ_JABKSS010000002.1 | 100 | 801 | 0 | 0 | 259962 | 260762 |
| NZ_JABKVH010000001.1 | 100 | 801 | 0 | 0 | 329761 | 330561 |
| NZ_JABLRO010000001.1 | 100 | 801 | 0 | 0 | 30054 | 29254 |
| NZ_JABKUI010000001.1 | 100 | 801 | 0 | 0 | 318291 | 319091 |
| NZ_JABKUN010000001.1 | 100 | 801 | 0 | 0 | 329982 | 330782 |
| NZ_JABKZL010000003.1 | 100 | 801 | 0 | 0 | 30033 | 29233 |
| NZ_JABKZN010000003.1 | 100 | 801 | 0 | 0 | 146694 | 147494 |
| NZ_JABKZM010000001.1 | 100 | 801 | 0 | 0 | 230686 | 231486 |
| NZ_JABKZU010000004.1 | 100 | 801 | 0 | 0 | 45765 | 44965 |
| NZ_JABLAG010000011.1 | 100 | 801 | 0 | 0 | 34723 | 35523 |
| NZ_JABKZV010000011.1 | 100 | 801 | 0 | 0 | 30039 | 29239 |
| NZ_JABLAF010000010.1 | 100 | 801 | 0 | 0 | 34723 | 35523 |
| NZ_JABLAU010000011.1 | 100 | 801 | 0 | 0 | 34737 | 35537 |
| NZ_JABLAX010000003.1 | 100 | 801 | 0 | 0 | 30025 | 29225 |
| NZ_JABLBH010000014.1 | 100 | 801 | 0 | 0 | 34738 | 35538 |
| NZ_JABLAM010000003.1 | 100 | 801 | 0 | 0 | 182827 | 183627 |
| NZ_JABLBF010000001.1 | 100 | 801 | 0 | 0 | 30025 | 29225 |
| NZ_JABLBG010000001.1 | 100 | 801 | 0 | 0 | 30037 | 29237 |
| NZ_JABLBT010000003.1 | 100 | 801 | 0 | 0 | 148103 | 148903 |
| NZ_JABLDI010000004.1 | 100 | 801 | 0 | 0 | 30037 | 29237 |
| NZ_JABLDH010000004.1 | 100 | 801 | 0 | 0 | 117624 | 118424 |
| NZ_JABLDK010000012.1 | 100 | 801 | 0 | 0 | 34737 | 35537 |
| NZ_JABLDM010000005.1 | 100 | 801 | 0 | 0 | 30037 | 29237 |
| NZ_JABLDO010000009.1 | 100 | 801 | 0 | 0 | 34739 | 35539 |
| NZ_JABLDR010000009.1 | 100 | 801 | 0 | 0 | 45888 | 46688 |
| NZ_JABLDQ010000002.1 | 100 | 801 | 0 | 0 | 182942 | 183742 |
| NZ_JABLDS010000002.1 | 100 | 801 | 0 | 0 | 182723 | 183523 |
| NZ_JABLDE010000002.1 | 100 | 801 | 0 | 0 | 206270 | 207070 |
| NZ_JABLEE010000002.1 | 100 | 801 | 0 | 0 | 30075 | 29275 |
| NZ_JABLEB010000002.1 | 100 | 801 | 0 | 0 | 189301 | 190101 |
| NZ_JABLEC010000004.1 | 100 | 801 | 0 | 0 | 30037 | 29237 |
| NZ_JABLEF010000008.1 | 100 | 801 | 0 | 0 | 45887 | 46687 |
| NZ_JABLED010000003.1 | 100 | 801 | 0 | 0 | 148824 | 149624 |
| NZ_JABLBU010000002.1 | 100 | 801 | 0 | 0 | 183151 | 183951 |
| NZ_JABLEH010000014.1 | 100 | 801 | 0 | 0 | 10839 | 11639 |
| NZ_JABLEK010000015.1 | 100 | 801 | 0 | 0 | 30037 | 29237 |
| NZ_JABLEJ010000005.1 | 100 | 801 | 0 | 0 | 83500 | 84300 |
| NZ_JABLDX010000003.1 | 100 | 801 | 0 | 0 | 183613 | 184413 |
| NZ_JABLEZ010000001.1 | 100 | 801 | 0 | 0 | 107221 | 106421 |
| NZ_JABLDD010000001.1 | 100 | 801 | 0 | 0 | 206271 | 207071 |
| NZ_JABLEM010000013.1 | 100 | 801 | 0 | 0 | 30037 | 29237 |
| NZ_JABLEY010000002.1 | 100 | 801 | 0 | 0 | 182522 | 183322 |
| NZ_JABLEP010000007.1 | 100 | 801 | 0 | 0 | 34737 | 35537 |
| NZ_JABLEO010000004.1 | 100 | 801 | 0 | 0 | 98361 | 97561 |
| NZ_JAAVLK010000001.1 | 100 | 801 | 0 | 0 | 324473 | 325273 |
| NZ_PKOW01000001.1 | 100 | 801 | 0 | 0 | 456805 | 457605 |
| NZ_PKOX01000002.1 | 100 | 801 | 0 | 0 | 30261 | 29461 |
| NZ_PKOV01000001.1 | 100 | 801 | 0 | 0 | 342698 | 343498 |
| NZ_WODB01000001.1 | 100 | 801 | 0 | 0 | 324473 | 325273 |
| NZ_VIFD01000001.1 | 100 | 801 | 0 | 0 | 730375 | 729575 |
| NZ_VIFA01000001.1 | 100 | 801 | 0 | 0 | 1393865 | 1394665 |
| NZ_VIEY01000001.1 | 100 | 801 | 0 | 0 | 1579707 | 1580507 |
| NZ_VIEJ01000002.1 | 100 | 801 | 0 | 0 | 257987 | 258787 |
| NZ_VIEC01000006.1 | 100 | 801 | 0 | 0 | 117682 | 118482 |
| NZ_VIEE01000002.1 | 100 | 801 | 0 | 0 | 259966 | 260766 |
| NZ_VIEG01000002.1 | 100 | 801 | 0 | 0 | 30084 | 29284 |
| NZ_RZIC01000008.1 | 100 | 801 | 0 | 0 | 5678 | 4878 |
| NZ_RSDH01000005.1 | 100 | 801 | 0 | 0 | 69582 | 70382 |
| NZ_RSDQ01000004.1 | 100 | 801 | 0 | 0 | 15066 | 14266 |
| NZ_RSDI01000004.1 | 100 | 801 | 0 | 0 | 15066 | 14266 |
| NZ_PYUF01000012.1 | 100 | 801 | 0 | 0 | 30084 | 29284 |
| NZ_PQGR01000001.1 | 100 | 801 | 0 | 0 | 334414 | 335214 |
| NZ_PQGQ01000001.1 | 100 | 801 | 0 | 0 | 30018 | 29218 |
| NZ_PQGN01000005.1 | 100 | 801 | 0 | 0 | 114080 | 114880 |
| NZ_PQGO01000001.1 | 100 | 801 | 0 | 0 | 338283 | 339083 |
| NZ_PQGM01000001.1 | 100 | 801 | 0 | 0 | 338033 | 338833 |
| NZ_PQGP01000004.1 | 100 | 801 | 0 | 0 | 140540 | 141340 |
| NZ_POOA01000077.1 | 100 | 801 | 0 | 0 | 52651 | 53451 |
| NZ_POMR01000143.1 | 100 | 801 | 0 | 0 | 29933 | 29133 |
| NZ_POOC01000215.1 | 100 | 801 | 0 | 0 | 29905 | 29105 |
| NZ_POKR01000204.1 | 100 | 801 | 0 | 0 | 29899 | 29099 |
| NZ_POIX01000024.1 | 100 | 801 | 0 | 0 | 30325 | 29525 |
| NZ_POIW01000202.1 | 100 | 801 | 0 | 0 | 29893 | 29093 |
| NZ_JRHU01000014.1 | 100 | 801 | 0 | 0 | 50437 | 51237 |
| NZ_CP024050.1 | 100 | 801 | 0 | 0 | 1051941 | 1052741 |
| NZ_CP016175.1 | 100 | 801 | 0 | 0 | 998451 | 999251 |
| NZ_CP020863.1 | 100 | 801 | 0 | 0 | 1025547 | 1026347 |
| NZ_CP018908.1 | 100 | 801 | 0 | 0 | 1001753 | 1002553 |
| NZ_MNPY01000001.1 | 100 | 801 | 0 | 0 | 30722 | 29922 |
| NZ_LDOJ01000045.1 | 100 | 801 | 0 | 0 | 21659 | 22459 |
| NZ_LDOM01000009.1 | 100 | 801 | 0 | 0 | 175707 | 176507 |
| NZ_CP007497.1 | 100 | 801 | 0 | 0 | 1025372 | 1026172 |
| NZ_CDXN01000001.1 | 100 | 801 | 0 | 0 | 30239 | 29439 |
| NZ_CEBO01000008.1 | 100 | 801 | 0 | 0 | 30027 | 29227 |
| NZ_CEKN01000003.1 | 100 | 801 | 0 | 0 | 30017 | 29217 |
| NZ_CDXH01000034.1 | 100 | 801 | 0 | 0 | 338475 | 339275 |
| NZ_CDXV01000022.1 | 100 | 801 | 0 | 0 | 421798 | 422598 |
| NZ_CEAV01000001.1 | 100 | 801 | 0 | 0 | 338299 | 339099 |
| NZ_CDTX01000011.1 | 100 | 801 | 0 | 0 | 342437 | 343237 |
| NZ_CDUN01000031.1 | 100 | 801 | 0 | 0 | 338289 | 339089 |
| NZ_CEBF01000022.1 | 100 | 801 | 0 | 0 | 421636 | 422436 |
| NZ_CDWS01000018.1 | 100 | 801 | 0 | 0 | 136128 | 136928 |
| NZ_CEHI01000054.1 | 100 | 801 | 0 | 0 | 271233 | 272033 |
| NZ_CEBJ01000035.1 | 100 | 801 | 0 | 0 | 30239 | 29439 |
| NZ_CEBR01000009.1 | 100 | 801 | 0 | 0 | 30025 | 29225 |
| NZ_CEGR01000055.1 | 100 | 801 | 0 | 0 | 145822 | 146622 |
| NZ_CEKP01000034.1 | 100 | 801 | 0 | 0 | 30009 | 29209 |
| NZ_CDWX01000039.1 | 100 | 801 | 0 | 0 | 259917 | 260717 |
| NZ_CEBW01000007.1 | 100 | 801 | 0 | 0 | 387157 | 387957 |
| NZ_CDTR01000005.1 | 100 | 801 | 0 | 0 | 30027 | 29227 |
| NZ_CDXC01000004.1 | 100 | 801 | 0 | 0 | 338298 | 339098 |
| NZ_CDVD01000010.1 | 100 | 801 | 0 | 0 | 330931 | 331731 |
| NZ_CDVX01000056.1 | 100 | 801 | 0 | 0 | 30239 | 29439 |
| NZ_CEBS01000006.1 | 100 | 801 | 0 | 0 | 30030 | 29230 |
| NZ_CDWK01000006.1 | 100 | 801 | 0 | 0 | 30027 | 29227 |
| NZ_CDUE01000001.1 | 100 | 801 | 0 | 0 | 338288 | 339088 |
| NZ_CEJY01000051.1 | 100 | 801 | 0 | 0 | 271252 | 272052 |
| NZ_CDVJ01000025.1 | 100 | 801 | 0 | 0 | 271389 | 272189 |
| NZ_CEJZ01000040.1 | 100 | 801 | 0 | 0 | 30011 | 29211 |
| NZ_CDYB01000001.1 | 100 | 801 | 0 | 0 | 30239 | 29439 |
| NZ_CDXS01000060.1 | 100 | 801 | 0 | 0 | 30239 | 29439 |
| NZ_CDWQ01000017.1 | 100 | 801 | 0 | 0 | 30027 | 29227 |
| NZ_CDUJ01000002.1 | 100 | 801 | 0 | 0 | 30027 | 29227 |
| NZ_CEHF01000017.1 | 100 | 801 | 0 | 0 | 240744 | 241544 |
| NZ_CDWA01000008.1 | 100 | 801 | 0 | 0 | 30027 | 29227 |
| NZ_CDSG01000004.1 | 100 | 801 | 0 | 0 | 30239 | 29439 |
| NZ_CEHG01000030.1 | 100 | 801 | 0 | 0 | 30268 | 29468 |
| NZ_CEDI01000022.1 | 100 | 801 | 0 | 0 | 258688 | 259488 |
| NZ_CEJF01000055.1 | 100 | 801 | 0 | 0 | 258681 | 259481 |
| NZ_CDWE01000028.1 | 100 | 801 | 0 | 0 | 82918 | 83718 |
| NZ_CDWN01000005.1 | 100 | 801 | 0 | 0 | 30027 | 29227 |
| NZ_CDWW01000002.1 | 100 | 801 | 0 | 0 | 136128 | 136928 |
| NZ_CDVG01000016.1 | 100 | 801 | 0 | 0 | 402993 | 403793 |
| NZ_CDSS01000015.1 | 100 | 801 | 0 | 0 | 344135 | 344935 |
| NZ_CECH01000001.1 | 100 | 801 | 0 | 0 | 349602 | 350402 |
| NZ_CDWB01000001.1 | 100 | 801 | 0 | 0 | 30027 | 29227 |
| NZ_CEDQ01000036.1 | 100 | 801 | 0 | 0 | 271070 | 271870 |
| NZ_CEJN01000021.1 | 100 | 801 | 0 | 0 | 321802 | 322602 |
| NZ_CEBI01000005.1 | 100 | 801 | 0 | 0 | 30074 | 29274 |
| NZ_CEIN01000048.1 | 100 | 801 | 0 | 0 | 114908 | 115708 |
| NZ_CDVE01000034.1 | 100 | 801 | 0 | 0 | 30019 | 29219 |
| NZ_CEDL01000052.1 | 100 | 801 | 0 | 0 | 48373 | 49173 |
| NZ_CEBU01000062.1 | 100 | 801 | 0 | 0 | 30027 | 29227 |
| NZ_CDVU01000005.1 | 100 | 801 | 0 | 0 | 349576 | 350376 |
| NZ_CDTD01000036.1 | 100 | 801 | 0 | 0 | 271549 | 272349 |
| NZ_CDUG01000025.1 | 100 | 801 | 0 | 0 | 338291 | 339091 |
| NZ_CDWP01000002.1 | 100 | 801 | 0 | 0 | 193044 | 193844 |
| NZ_CDTT01000003.1 | 100 | 801 | 0 | 0 | 338301 | 339101 |
| NZ_CDUD01000003.1 | 100 | 801 | 0 | 0 | 338291 | 339091 |
| NZ_CDSO01000014.1 | 100 | 801 | 0 | 0 | 410354 | 411154 |
| NZ_CDTQ01000050.1 | 100 | 801 | 0 | 0 | 271096 | 271896 |
| NZ_CDXA01000030.1 | 100 | 801 | 0 | 0 | 354641 | 355441 |
| NZ_CEBL01000006.1 | 100 | 801 | 0 | 0 | 338379 | 339179 |
| NZ_CEKC01000050.1 | 100 | 801 | 0 | 0 | 114798 | 115598 |
| NZ_CEJX01000019.1 | 100 | 801 | 0 | 0 | 30011 | 29211 |
| NZ_CDWC01000007.1 | 100 | 801 | 0 | 0 | 387074 | 387874 |
| NZ_CDXK01000001.1 | 100 | 801 | 0 | 0 | 343942 | 344742 |
| NZ_CDUC01000003.1 | 100 | 801 | 0 | 0 | 30027 | 29227 |
| NZ_CEGQ01000048.1 | 100 | 801 | 0 | 0 | 329387 | 330187 |
| NZ_CDWT01000002.1 | 100 | 801 | 0 | 0 | 110718 | 111518 |
| NZ_CDSW01000010.1 | 100 | 801 | 0 | 0 | 30027 | 29227 |
| NZ_CEBG01000002.1 | 100 | 801 | 0 | 0 | 30027 | 29227 |
| NZ_CDUU01000019.1 | 100 | 801 | 0 | 0 | 338288 | 339088 |
| NZ_CDTJ01000015.1 | 100 | 801 | 0 | 0 | 88999 | 89799 |
| NZ_CDUW01000054.1 | 100 | 801 | 0 | 0 | 30027 | 29227 |
| NZ_CDTO01000005.1 | 100 | 801 | 0 | 0 | 338299 | 339099 |
| NZ_CDTE01000006.1 | 100 | 801 | 0 | 0 | 30027 | 29227 |
| NZ_CEDK01000054.1 | 100 | 801 | 0 | 0 | 259919 | 260719 |
| NZ_CDVQ01000017.1 | 100 | 801 | 0 | 0 | 30027 | 29227 |
| NZ_CDTI01000009.1 | 100 | 801 | 0 | 0 | 30027 | 29227 |
| NZ_CEKB01000019.1 | 100 | 801 | 0 | 0 | 271075 | 271875 |
| NZ_CDWZ01000028.1 | 100 | 801 | 0 | 0 | 30239 | 29439 |
| NZ_CEBC01000035.1 | 100 | 801 | 0 | 0 | 349596 | 350396 |
| NZ_CDTV01000025.1 | 100 | 801 | 0 | 0 | 338302 | 339102 |
| NZ_CDUI01000039.1 | 100 | 801 | 0 | 0 | 30027 | 29227 |
| NZ_CDSX01000036.1 | 100 | 801 | 0 | 0 | 30027 | 29227 |
| NZ_CECK01000041.1 | 100 | 801 | 0 | 0 | 338379 | 339179 |
| NZ_CDVO01000002.1 | 100 | 801 | 0 | 0 | 30027 | 29227 |
| NZ_CDTK01000021.1 | 100 | 801 | 0 | 0 | 338485 | 339285 |
| NZ_CDVW01000011.1 | 100 | 801 | 0 | 0 | 30027 | 29227 |
| NZ_CDVN01000036.1 | 100 | 801 | 0 | 0 | 35356 | 34556 |
| NZ_CEJV01000014.1 | 100 | 801 | 0 | 0 | 341194 | 341994 |
| NZ_CEBB01000005.1 | 100 | 801 | 0 | 0 | 30239 | 29439 |
| NZ_CDXU01000010.1 | 100 | 801 | 0 | 0 | 349670 | 350470 |
| NZ_CEKK01000052.1 | 100 | 801 | 0 | 0 | 271067 | 271867 |
| NZ_CEBM01000016.1 | 100 | 801 | 0 | 0 | 30027 | 29227 |
| NZ_CEHQ01000054.1 | 100 | 801 | 0 | 0 | 49081 | 49881 |
| NZ_CDXX01000052.1 | 100 | 801 | 0 | 0 | 30027 | 29227 |
| NZ_CDWV01000018.1 | 100 | 801 | 0 | 0 | 60570 | 61370 |
| NZ_CEKM01000016.1 | 100 | 801 | 0 | 0 | 271231 | 272031 |
| NZ_CEIF01000042.1 | 100 | 801 | 0 | 0 | 271187 | 271987 |
| NZ_CEIJ01000019.1 | 100 | 801 | 0 | 0 | 30011 | 29211 |
| NZ_CDXO01000034.1 | 100 | 801 | 0 | 0 | 30027 | 29227 |
| NZ_CDWH01000004.1 | 100 | 801 | 0 | 0 | 30027 | 29227 |
| NZ_CEJJ01000050.1 | 100 | 801 | 0 | 0 | 118701 | 119501 |
| NZ_CEJH01000055.1 | 100 | 801 | 0 | 0 | 80914 | 81714 |
| NZ_CDSP01000028.1 | 100 | 801 | 0 | 0 | 344155 | 344955 |
| NZ_CDUO01000051.1 | 100 | 801 | 0 | 0 | 270195 | 270995 |
| NZ_CDUQ01000006.1 | 100 | 801 | 0 | 0 | 30027 | 29227 |
| NZ_CDTM01000008.1 | 100 | 801 | 0 | 0 | 349651 | 350451 |
| NZ_CEKD01000053.1 | 100 | 801 | 0 | 0 | 270938 | 271738 |
| NZ_CDVI01000005.1 | 100 | 801 | 0 | 0 | 343986 | 344786 |
| NZ_CEJU01000003.1 | 100 | 801 | 0 | 0 | 312417 | 313217 |
| NZ_CDSH01000046.1 | 100 | 801 | 0 | 0 | 30027 | 29227 |
| NZ_CDWO01000006.1 | 100 | 801 | 0 | 0 | 30027 | 29227 |
| NZ_CDTZ01000009.1 | 100 | 801 | 0 | 0 | 30027 | 29227 |
| NZ_CDVH01000001.1 | 100 | 801 | 0 | 0 | 30027 | 29227 |
| NZ_CDXG01000037.1 | 100 | 801 | 0 | 0 | 30239 | 29439 |
| NZ_CDVT01000029.1 | 100 | 801 | 0 | 0 | 193200 | 194000 |
| NZ_CDUK01000044.1 | 100 | 801 | 0 | 0 | 30027 | 29227 |
| NZ_CDUF01000004.1 | 100 | 801 | 0 | 0 | 337890 | 338690 |
| NZ_CEDA01000017.1 | 100 | 801 | 0 | 0 | 30023 | 29223 |
| NZ_CDTS01000049.1 | 100 | 801 | 0 | 0 | 30248 | 29448 |
| NZ_CDUM01000052.1 | 100 | 801 | 0 | 0 | 271272 | 272072 |
| NZ_CDTL01000002.1 | 100 | 801 | 0 | 0 | 30027 | 29227 |
| NZ_CDXM01000001.1 | 100 | 801 | 0 | 0 | 337890 | 338690 |
| NZ_CDVA01000020.1 | 100 | 801 | 0 | 0 | 338290 | 339090 |
| NZ_CEIU01000015.1 | 100 | 801 | 0 | 0 | 341216 | 342016 |
| NZ_CEIM01000010.1 | 100 | 801 | 0 | 0 | 271370 | 272170 |
| NZ_CDXW01000003.1 | 100 | 801 | 0 | 0 | 354680 | 355480 |
| NZ_CDUV01000007.1 | 100 | 801 | 0 | 0 | 30027 | 29227 |
| NZ_CECC01000019.1 | 100 | 801 | 0 | 0 | 30027 | 29227 |
| NZ_CEGS01000052.1 | 100 | 801 | 0 | 0 | 92485 | 93285 |
| NZ_CDYC01000152.1 | 100 | 801 | 0 | 0 | 271203 | 272003 |
| NZ_CDYD01000011.1 | 100 | 801 | 0 | 0 | 30027 | 29227 |
| NZ_CDUR01000018.1 | 100 | 801 | 0 | 0 | 73664 | 74464 |
| NZ_CDYO01000046.1 | 100 | 801 | 0 | 0 | 338285 | 339085 |
| NZ_CDTF01000031.1 | 100 | 801 | 0 | 0 | 30027 | 29227 |
| NZ_CDVS01000019.1 | 100 | 801 | 0 | 0 | 343274 | 344074 |
| NZ_CEKJ01000048.1 | 100 | 801 | 0 | 0 | 271183 | 271983 |
| NZ_CDXJ01000008.1 | 100 | 801 | 0 | 0 | 338300 | 339100 |
| NZ_CEKQ01000045.1 | 100 | 801 | 0 | 0 | 83497 | 84297 |
| NZ_CDVP01000012.1 | 100 | 801 | 0 | 0 | 271235 | 272035 |
| NZ_CDYW01000001.1 | 100 | 801 | 0 | 0 | 30239 | 29439 |
| NZ_CDUY01000021.1 | 100 | 801 | 0 | 0 | 30027 | 29227 |
| NZ_CEHA01000052.1 | 100 | 801 | 0 | 0 | 117601 | 118401 |
| NZ_CDUL01000067.1 | 100 | 801 | 0 | 0 | 30239 | 29439 |
| NZ_CDXI01000004.1 | 100 | 801 | 0 | 0 | 338299 | 339099 |
| NZ_CDWR01000006.1 | 100 | 801 | 0 | 0 | 136126 | 136926 |
| NZ_CELM01000028.1 | 100 | 801 | 0 | 0 | 30007 | 29207 |
| NZ_CEEY01000005.1 | 100 | 801 | 0 | 0 | 258686 | 259486 |
| NZ_CDWY01000031.1 | 100 | 801 | 0 | 0 | 30027 | 29227 |
| NZ_CDTB01000010.1 | 100 | 801 | 0 | 0 | 30027 | 29227 |
| NZ_CDVV01000001.1 | 100 | 801 | 0 | 0 | 30027 | 29227 |
| NZ_CEAW01000015.1 | 100 | 801 | 0 | 0 | 30027 | 29227 |
| NZ_CEAY01000048.1 | 100 | 801 | 0 | 0 | 349729 | 350529 |
| NZ_CDVC01000008.1 | 100 | 801 | 0 | 0 | 30027 | 29227 |
| NZ_CDUX01000013.1 | 100 | 801 | 0 | 0 | 30027 | 29227 |
| NZ_CECN01000009.1 | 100 | 801 | 0 | 0 | 30239 | 29439 |
| NZ_CDSU01000007.1 | 100 | 801 | 0 | 0 | 346021 | 346821 |
| NZ_CDWF01000003.1 | 100 | 801 | 0 | 0 | 338298 | 339098 |
| NZ_CDSQ01000157.1 | 100 | 801 | 0 | 0 | 30105 | 29305 |
| NZ_CDXQ01000003.1 | 100 | 801 | 0 | 0 | 349576 | 350376 |
| NZ_CDXP01000002.1 | 100 | 801 | 0 | 0 | 30027 | 29227 |
| NZ_CDXT01000058.1 | 100 | 801 | 0 | 0 | 82634 | 83434 |
| NZ_CECL01000003.1 | 100 | 801 | 0 | 0 | 30239 | 29439 |
| NZ_CEBZ01000023.1 | 100 | 801 | 0 | 0 | 349677 | 350477 |
| NZ_CDWU01000008.1 | 100 | 801 | 0 | 0 | 338298 | 339098 |
| NZ_CEBH01000011.1 | 100 | 801 | 0 | 0 | 30027 | 29227 |
| NZ_CDVK01000014.1 | 100 | 801 | 0 | 0 | 338337 | 339137 |
| NZ_CECQ01000013.1 | 100 | 801 | 0 | 0 | 114804 | 115604 |
| NZ_CEHD01000045.1 | 100 | 801 | 0 | 0 | 83484 | 84284 |
| NZ_CDSZ01000001.1 | 100 | 801 | 0 | 0 | 30027 | 29227 |
| NZ_CEEB01000058.1 | 100 | 801 | 0 | 0 | 114908 | 115708 |
| NZ_CEDX01000065.1 | 100 | 801 | 0 | 0 | 271224 | 272024 |
| NZ_CDUZ01000008.1 | 100 | 801 | 0 | 0 | 338279 | 339079 |
| NZ_CEJI01000053.1 | 100 | 801 | 0 | 0 | 148704 | 149504 |
| NZ_CDST01000005.1 | 100 | 801 | 0 | 0 | 343948 | 344748 |
| NZ_CECT01000010.1 | 100 | 801 | 0 | 0 | 258690 | 259490 |
| NZ_CDSK01000009.1 | 100 | 801 | 0 | 0 | 30027 | 29227 |
| NZ_CECO01000026.1 | 100 | 801 | 0 | 0 | 30239 | 29439 |
| NZ_CEKT01000036.1 | 100 | 801 | 0 | 0 | 30013 | 29213 |
| NZ_CEBP01000059.1 | 100 | 801 | 0 | 0 | 30239 | 29439 |
| NZ_CEBA01000050.1 | 100 | 801 | 0 | 0 | 349596 | 350396 |
| NZ_CDVF01000007.1 | 100 | 801 | 0 | 0 | 338332 | 339132 |
| NZ_CDTC01000006.1 | 100 | 801 | 0 | 0 | 338291 | 339091 |
| NZ_CDSN01000046.1 | 100 | 801 | 0 | 0 | 30027 | 29227 |
| NZ_CDWD01000010.1 | 100 | 801 | 0 | 0 | 271091 | 271891 |
| NZ_CEJR01000055.1 | 100 | 801 | 0 | 0 | 107611 | 108411 |
| NZ_CDXZ01000037.1 | 100 | 801 | 0 | 0 | 30027 | 29227 |
| NZ_CEIR01000051.1 | 100 | 801 | 0 | 0 | 271074 | 271874 |
| NZ_CEIX01000052.1 | 100 | 801 | 0 | 0 | 83485 | 84285 |
| NZ_CEKI01000033.1 | 100 | 801 | 0 | 0 | 30015 | 29215 |
| NZ_CDXE01000010.1 | 100 | 801 | 0 | 0 | 338298 | 339098 |
| NZ_CDXR01000009.1 | 100 | 801 | 0 | 0 | 338282 | 339082 |
| NZ_CEBN01000018.1 | 100 | 801 | 0 | 0 | 30027 | 29227 |
| NZ_CEJB01000049.1 | 100 | 801 | 0 | 0 | 259926 | 260726 |
| NZ_CDSR01000007.1 | 100 | 801 | 0 | 0 | 349693 | 350493 |
| NZ_CDSY01000011.1 | 100 | 801 | 0 | 0 | 271229 | 272029 |
| NZ_CDVL01000005.1 | 100 | 801 | 0 | 0 | 30239 | 29439 |
| NZ_CDXY01000004.1 | 100 | 801 | 0 | 0 | 30027 | 29227 |
| NZ_CECP01000008.1 | 100 | 801 | 0 | 0 | 30022 | 29222 |
| NZ_CDSL01000004.1 | 100 | 801 | 0 | 0 | 30027 | 29227 |
| NZ_CDSM01000005.1 | 100 | 801 | 0 | 0 | 30027 | 29227 |
| NZ_CEJQ01000024.1 | 100 | 801 | 0 | 0 | 321619 | 322419 |
| NZ_CEBK01000053.1 | 100 | 801 | 0 | 0 | 271285 | 272085 |
| NZ_CDUT01000017.1 | 100 | 801 | 0 | 0 | 73664 | 74464 |
| NZ_CDWL01000049.1 | 100 | 801 | 0 | 0 | 30027 | 29227 |
| NZ_CDTA01000049.1 | 100 | 801 | 0 | 0 | 259952 | 260752 |
| NZ_CDUA01000057.1 | 100 | 801 | 0 | 0 | 30239 | 29439 |
| NZ_CDTU01000051.1 | 100 | 801 | 0 | 0 | 30027 | 29227 |
| NZ_CDVM01000004.1 | 100 | 801 | 0 | 0 | 349678 | 350478 |
| NZ_CDXF01000008.1 | 100 | 801 | 0 | 0 | 30106 | 29306 |
| NZ_CEBD01000022.1 | 100 | 801 | 0 | 0 | 30239 | 29439 |
| NZ_CEJE01000003.1 | 100 | 801 | 0 | 0 | 341329 | 342129 |
| NZ_CEIK01000049.1 | 100 | 801 | 0 | 0 | 267602 | 268402 |
| NZ_CDYA01000012.1 | 100 | 801 | 0 | 0 | 30027 | 29227 |
| NZ_CEHE01000034.1 | 100 | 801 | 0 | 0 | 271184 | 271984 |
| NZ_CEAS01000033.1 | 100 | 801 | 0 | 0 | 30239 | 29439 |
| NZ_CDUB01000002.1 | 100 | 801 | 0 | 0 | 30027 | 29227 |
| NZ_CEHL01000049.1 | 100 | 801 | 0 | 0 | 114916 | 115716 |
| NZ_CEIP01000058.1 | 100 | 801 | 0 | 0 | 86198 | 86998 |
| NZ_CEHC01000053.1 | 100 | 801 | 0 | 0 | 260214 | 261014 |
| NZ_CEHZ01000049.1 | 100 | 801 | 0 | 0 | 21747 | 22547 |
| NZ_CDVZ01000003.1 | 100 | 801 | 0 | 0 | 338299 | 339099 |
| NZ_CDWM01000024.1 | 100 | 801 | 0 | 0 | 30025 | 29225 |
| NZ_CDTP01000012.1 | 100 | 801 | 0 | 0 | 30270 | 29470 |
| NZ_CEAT01000011.1 | 100 | 801 | 0 | 0 | 349602 | 350402 |
| NZ_CECX01000008.1 | 100 | 801 | 0 | 0 | 30025 | 29225 |
| NZ_CDSJ01000026.1 | 100 | 801 | 0 | 0 | 259968 | 260768 |
| NZ_CDTN01000002.1 | 100 | 801 | 0 | 0 | 337895 | 338695 |
| NZ_CEBX01000013.1 | 100 | 801 | 0 | 0 | 349598 | 350398 |
| NZ_CEFH01000057.1 | 100 | 801 | 0 | 0 | 90199 | 90999 |
| NZ_CDXL01000003.1 | 100 | 801 | 0 | 0 | 30027 | 29227 |
| NZ_CDVB01000010.1 | 100 | 801 | 0 | 0 | 30027 | 29227 |
| NZ_CEAU01000138.1 | 100 | 801 | 0 | 0 | 271495 | 272295 |
| NZ_CDSI01000025.1 | 100 | 801 | 0 | 0 | 391260 | 392060 |
| NZ_CEBT01000144.1 | 100 | 801 | 0 | 0 | 938 | 138 |
| NZ_CEBE01000005.1 | 100 | 801 | 0 | 0 | 271100 | 271900 |
| NZ_CDWG01000006.1 | 100 | 801 | 0 | 0 | 344205 | 345005 |
| NZ_CDVR01000013.1 | 100 | 801 | 0 | 0 | 30239 | 29439 |
| NZ_CDUP01000009.1 | 100 | 801 | 0 | 0 | 338285 | 339085 |
| NZ_CECD01000008.1 | 100 | 801 | 0 | 0 | 30027 | 29227 |
| NZ_CDTH01000001.1 | 100 | 801 | 0 | 0 | 259928 | 260728 |
| NZ_CDXD01000046.1 | 100 | 801 | 0 | 0 | 30027 | 29227 |
| NZ_CDTG01000007.1 | 100 | 801 | 0 | 0 | 338374 | 339174 |
| NZ_CDSV01000010.1 | 100 | 801 | 0 | 0 | 30027 | 29227 |
| NZ_ALMG01000019.1 | 100 | 801 | 0 | 0 | 132562 | 133362 |
| NZ_ALLS01000018.1 | 100 | 801 | 0 | 0 | 259923 | 260723 |
| NZ_ALLA01000002.1 | 100 | 801 | 0 | 0 | 20945 | 20145 |
| NZ_ALKR01000010.1 | 100 | 801 | 0 | 0 | 30035 | 29235 |
| NZ_ALKP01000002.1 | 100 | 801 | 0 | 0 | 30031 | 29231 |
| NZ_ALNE01000012.1 | 100 | 801 | 0 | 0 | 30031 | 29231 |
| NC_020526.1 | 100 | 801 | 0 | 0 | 1052069 | 1052869 |
| NC_018526.1 | 100 | 801 | 0 | 0 | 921681 | 922481 |
| NC_017622.1 | 100 | 801 | 0 | 0 | 968538 | 969338 |
| NC_017619.1 | 100 | 801 | 0 | 0 | 1001437 | 1002237 |
| NC_017618.1 | 100 | 801 | 0 | 0 | 1049244 | 1050044 |
| NC_012925.1 | 100 | 801 | 0 | 0 | 937062 | 937862 |
| NC_012926.1 | 100 | 801 | 0 | 0 | 941887 | 941087 |
| NC_012924.1 | 100 | 801 | 0 | 0 | 1025474 | 1026274 |
| NC_017617.1 | 100 | 801 | 0 | 0 | 977191 | 977991 |
| NC_009443.1 | 100 | 801 | 0 | 0 | 1025429 | 1026229 |
| NC_009442.1 | 100 | 801 | 0 | 0 | 1025750 | 1026550 |
| LR738721.1 | 100 | 801 | 0 | 0 | 937011 | 937811 |
| CAAALZ010000009.1 | 100 | 801 | 0 | 0 | 271406 | 272206 |
| LS483418.1 | 100 | 801 | 0 | 0 | 924577 | 925377 |
| FWZS01000033.1 | 100 | 801 | 0 | 0 | 259946 | 260746 |
| FISV01000001.1 | 100 | 801 | 0 | 0 | 30046 | 29246 |
| FIPV01000002.1 | 100 | 801 | 0 | 0 | 30062 | 29262 |
| FIOP01000003.1 | 100 | 801 | 0 | 0 | 204495 | 205295 |
| FION01000002.1 | 100 | 801 | 0 | 0 | 259971 | 260771 |
| FILH01000001.1 | 100 | 801 | 0 | 0 | 271036 | 271836 |
| FILA01000003.1 | 100 | 801 | 0 | 0 | 259921 | 260721 |
| FIJV01000002.1 | 100 | 801 | 0 | 0 | 271026 | 271826 |
| FIFI01000002.1 | 100 | 801 | 0 | 0 | 30031 | 29231 |
| FIFD01000002.1 | 100 | 801 | 0 | 0 | 30025 | 29225 |
| FIOO01000001.1 | 100 | 801 | 0 | 0 | 384030 | 384830 |
| FIMW01000001.1 | 100 | 801 | 0 | 0 | 343863 | 344663 |
| FIMP01000001.1 | 100 | 801 | 0 | 0 | 27027 | 26227 |
| FISO01000002.1 | 100 | 801 | 0 | 0 | 338322 | 339122 |
| FISS01000001.1 | 100 | 801 | 0 | 0 | 30274 | 29474 |
| FIQP01000002.1 | 100 | 801 | 0 | 0 | 338422 | 339222 |
| FITQ01000002.1 | 100 | 801 | 0 | 0 | 30046 | 29246 |
| FISW01000008.1 | 100 | 801 | 0 | 0 | 30049 | 29249 |
| FIMG01000002.1 | 100 | 801 | 0 | 0 | 30063 | 29263 |
| FIMF01000002.1 | 100 | 801 | 0 | 0 | 271126 | 271926 |
| FILC01000002.1 | 100 | 801 | 0 | 0 | 271181 | 271981 |
| FIJW01000001.1 | 100 | 801 | 0 | 0 | 341160 | 341960 |
| FIJT01000002.1 | 100 | 801 | 0 | 0 | 30022 | 29222 |
| FISJ01000002.1 | 100 | 801 | 0 | 0 | 338310 | 339110 |
| FIQR01000001.1 | 100 | 801 | 0 | 0 | 30061 | 29261 |
| FIPW01000002.1 | 100 | 801 | 0 | 0 | 30061 | 29261 |
| FIKX01000002.1 | 100 | 801 | 0 | 0 | 271173 | 271973 |
| FIIV01000004.1 | 100 | 801 | 0 | 0 | 118606 | 119406 |
| FISK01000001.1 | 100 | 801 | 0 | 0 | 30061 | 29261 |
| FIOM01000001.1 | 100 | 801 | 0 | 0 | 356358 | 357158 |
| FIQA01000001.1 | 100 | 801 | 0 | 0 | 30063 | 29263 |
| FIPX01000004.1 | 100 | 801 | 0 | 0 | 30267 | 29467 |
| FIPF01000002.1 | 100 | 801 | 0 | 0 | 338315 | 339115 |
| FINP01000001.1 | 100 | 801 | 0 | 0 | 30275 | 29475 |
| FINM01000001.1 | 100 | 801 | 0 | 0 | 30305 | 29505 |
| FIKA01000004.1 | 100 | 801 | 0 | 0 | 83493 | 84293 |
| FIGM01000001.1 | 100 | 801 | 0 | 0 | 321626 | 322426 |
| FIQQ01000005.1 | 100 | 801 | 0 | 0 | 70880 | 71680 |
| FIOG01000001.1 | 100 | 801 | 0 | 0 | 30045 | 29245 |
| FISF01000002.1 | 100 | 801 | 0 | 0 | 271108 | 271908 |
| FIIZ01000001.1 | 100 | 801 | 0 | 0 | 321761 | 322561 |
| FITJ01000009.1 | 100 | 801 | 0 | 0 | 60588 | 61388 |
| FIIW01000001.1 | 100 | 801 | 0 | 0 | 329382 | 330182 |
| FIFU01000002.1 | 100 | 801 | 0 | 0 | 271228 | 272028 |
| FISH01000002.1 | 100 | 801 | 0 | 0 | 338308 | 339108 |
| FISI01000001.1 | 100 | 801 | 0 | 0 | 30360 | 29560 |
| FIIU01000001.1 | 100 | 801 | 0 | 0 | 329386 | 330186 |
| FITL01000004.1 | 100 | 801 | 0 | 0 | 35457 | 34657 |
| FISB01000001.1 | 100 | 801 | 0 | 0 | 410393 | 411193 |
| FISC01000001.1 | 100 | 801 | 0 | 0 | 410385 | 411185 |
| FIRZ01000001.1 | 100 | 801 | 0 | 0 | 349600 | 350400 |
| FIOK01000002.1 | 100 | 801 | 0 | 0 | 334287 | 335087 |
| FILT01000002.1 | 100 | 801 | 0 | 0 | 276491 | 277291 |
| FILM01000007.1 | 100 | 801 | 0 | 0 | 117645 | 118445 |
| FISA01000001.1 | 100 | 801 | 0 | 0 | 30059 | 29259 |
| FIOF01000001.1 | 100 | 801 | 0 | 0 | 349577 | 350377 |
| FIOH01000002.1 | 100 | 801 | 0 | 0 | 338319 | 339119 |
| FINS01000004.1 | 100 | 801 | 0 | 0 | 193216 | 194016 |
| FINO01000002.1 | 100 | 801 | 0 | 0 | 337904 | 338704 |
| FINN01000002.1 | 100 | 801 | 0 | 0 | 344259 | 345059 |
| FINL01000002.1 | 100 | 801 | 0 | 0 | 30062 | 29262 |
| FIMK01000002.1 | 100 | 801 | 0 | 0 | 259951 | 260751 |
| FIMJ01000001.1 | 100 | 801 | 0 | 0 | 30274 | 29474 |
| FIMH01000002.1 | 100 | 801 | 0 | 0 | 271159 | 271959 |
| FIME01000001.1 | 100 | 801 | 0 | 0 | 30061 | 29261 |
| FIPP01000002.1 | 100 | 801 | 0 | 0 | 30061 | 29261 |
| FIPO01000002.1 | 100 | 801 | 0 | 0 | 259974 | 260774 |
| FIPL01000001.1 | 100 | 801 | 0 | 0 | 30275 | 29475 |
| FIOD01000001.1 | 100 | 801 | 0 | 0 | 30060 | 29260 |
| FIOE01000001.1 | 100 | 801 | 0 | 0 | 338632 | 339432 |
| FIGD01000001.1 | 100 | 801 | 0 | 0 | 271191 | 271991 |
| FIRY01000001.1 | 100 | 801 | 0 | 0 | 349612 | 350412 |
| FIPM01000002.1 | 100 | 801 | 0 | 0 | 30061 | 29261 |
| FIOB01000001.1 | 100 | 801 | 0 | 0 | 30061 | 29261 |
| FIPB01000002.1 | 100 | 801 | 0 | 0 | 338304 | 339104 |
| FINU01000001.1 | 100 | 801 | 0 | 0 | 349622 | 350422 |
| FINH01000002.1 | 100 | 801 | 0 | 0 | 30045 | 29245 |
| FIHO01000002.1 | 100 | 801 | 0 | 0 | 271065 | 271865 |
| FINV01000001.1 | 100 | 801 | 0 | 0 | 30063 | 29263 |
| FIKG01000001.1 | 100 | 801 | 0 | 0 | 267456 | 268256 |
| FIKH01000001.1 | 100 | 801 | 0 | 0 | 341168 | 341968 |
| FIQN01000001.1 | 100 | 801 | 0 | 0 | 336911 | 337711 |
| FIQL01000253.1 | 100 | 801 | 0 | 0 | 938 | 138 |
| FIPT01000002.1 | 100 | 801 | 0 | 0 | 171660 | 172460 |
| FIPK01000002.1 | 100 | 801 | 0 | 0 | 30059 | 29259 |
| FIPH01000001.1 | 100 | 801 | 0 | 0 | 30063 | 29263 |
| FINI01000002.1 | 100 | 801 | 0 | 0 | 30054 | 29254 |
| FIMV01000003.1 | 100 | 801 | 0 | 0 | 259975 | 260775 |
| FIMR01000001.1 | 100 | 801 | 0 | 0 | 338657 | 339457 |
| FIMQ01000002.1 | 100 | 801 | 0 | 0 | 259976 | 260776 |
| FIKC01000001.1 | 100 | 801 | 0 | 0 | 286808 | 287608 |
| FIJN01000002.1 | 100 | 801 | 0 | 0 | 259928 | 260728 |
| FITG01000001.1 | 100 | 801 | 0 | 0 | 30061 | 29261 |
| FIQM01000001.1 | 100 | 801 | 0 | 0 | 30054 | 29254 |
| FIPJ01000001.1 | 100 | 801 | 0 | 0 | 30043 | 29243 |
| FINT01000001.1 | 100 | 801 | 0 | 0 | 30061 | 29261 |
| FINJ01000003.1 | 100 | 801 | 0 | 0 | 271164 | 271964 |
| FILD01000008.1 | 100 | 801 | 0 | 0 | 83502 | 84302 |
| FIJH01000002.1 | 100 | 801 | 0 | 0 | 276439 | 277239 |
| FIIT01000001.1 | 100 | 801 | 0 | 0 | 271070 | 271870 |
| FISQ01000002.1 | 100 | 801 | 0 | 0 | 30063 | 29263 |
| FIRG01000001.1 | 100 | 801 | 0 | 0 | 30271 | 29471 |
| FIPN01000001.1 | 100 | 801 | 0 | 0 | 338318 | 339118 |
| FIOJ01000001.1 | 100 | 801 | 0 | 0 | 30061 | 29261 |
| FIOI01000003.1 | 100 | 801 | 0 | 0 | 171663 | 172463 |
| FIRX01000002.1 | 100 | 801 | 0 | 0 | 30148 | 29348 |
| FIQS01000001.1 | 100 | 801 | 0 | 0 | 338426 | 339226 |
| FIPG01000003.1 | 100 | 801 | 0 | 0 | 30059 | 29259 |
| FITK01000004.1 | 100 | 801 | 0 | 0 | 36749 | 35949 |
| FITE01000003.1 | 100 | 801 | 0 | 0 | 271110 | 271910 |
| FIQI01000002.1 | 100 | 801 | 0 | 0 | 30047 | 29247 |
| FIQJ01000001.1 | 100 | 801 | 0 | 0 | 375004 | 375804 |
| FIPS01000001.1 | 100 | 801 | 0 | 0 | 30062 | 29262 |
| FINC01000001.1 | 100 | 801 | 0 | 0 | 30048 | 29248 |
| FIHK01000001.1 | 100 | 801 | 0 | 0 | 271074 | 271874 |
| FITF01000009.1 | 100 | 801 | 0 | 0 | 30021 | 29221 |
| FIRV01000002.1 | 100 | 801 | 0 | 0 | 30045 | 29245 |
| FIOU01000002.1 | 100 | 801 | 0 | 0 | 338315 | 339115 |
| FIOS01000001.1 | 100 | 801 | 0 | 0 | 337902 | 338702 |
| FIJO01000001.1 | 100 | 801 | 0 | 0 | 312418 | 313218 |
| FIFR01000001.1 | 100 | 801 | 0 | 0 | 346876 | 347676 |
| FITI01000005.1 | 100 | 801 | 0 | 0 | 136157 | 136957 |
| FIRK01000001.1 | 100 | 801 | 0 | 0 | 360390 | 361190 |
| FIQB01000001.1 | 100 | 801 | 0 | 0 | 349632 | 350432 |
| FIQD01000001.1 | 100 | 801 | 0 | 0 | 354909 | 355709 |
| FITC01000006.1 | 100 | 801 | 0 | 0 | 35750 | 34950 |
| FIQG01000001.1 | 100 | 801 | 0 | 0 | 30273 | 29473 |
| FIOZ01000002.1 | 100 | 801 | 0 | 0 | 387084 | 387884 |
| FINE01000002.1 | 100 | 801 | 0 | 0 | 30061 | 29261 |
| FIOA01000002.1 | 100 | 801 | 0 | 0 | 271258 | 272058 |
| FINF01000002.1 | 100 | 801 | 0 | 0 | 30043 | 29243 |
| FIQO01000001.1 | 100 | 801 | 0 | 0 | 30270 | 29470 |
| FIQH01000001.1 | 100 | 801 | 0 | 0 | 354788 | 355588 |
| FIPC01000001.1 | 100 | 801 | 0 | 0 | 338320 | 339120 |
| FITD01000002.1 | 100 | 801 | 0 | 0 | 330952 | 331752 |
| FISD01000002.1 | 100 | 801 | 0 | 0 | 30328 | 29528 |
| FISE01000001.1 | 100 | 801 | 0 | 0 | 39034 | 38234 |
| FIQU01000001.1 | 100 | 801 | 0 | 0 | 30062 | 29262 |
| FIPR01000001.1 | 100 | 801 | 0 | 0 | 30060 | 29260 |
| FILQ01000002.1 | 100 | 801 | 0 | 0 | 265372 | 266172 |
| FILI01000002.1 | 100 | 801 | 0 | 0 | 271176 | 271976 |
| FILJ01000002.1 | 100 | 801 | 0 | 0 | 271232 | 272032 |
| FITB01000001.1 | 100 | 801 | 0 | 0 | 30063 | 29263 |
| FIRS01000001.1 | 100 | 801 | 0 | 0 | 30272 | 29472 |
| FIRO01000001.1 | 100 | 801 | 0 | 0 | 338306 | 339106 |
| FINZ01000002.1 | 100 | 801 | 0 | 0 | 30275 | 29475 |
| FIRW01000001.1 | 100 | 801 | 0 | 0 | 30274 | 29474 |
| FIRP01000001.1 | 100 | 801 | 0 | 0 | 349611 | 350411 |
| FIRM01000002.1 | 100 | 801 | 0 | 0 | 349556 | 350356 |
| FIRF01000001.1 | 100 | 801 | 0 | 0 | 349606 | 350406 |
| FIRN01000001.1 | 100 | 801 | 0 | 0 | 30272 | 29472 |
| FIRL01000002.1 | 100 | 801 | 0 | 0 | 30274 | 29474 |
| FIRJ01000002.1 | 100 | 801 | 0 | 0 | 30060 | 29260 |
| FIRE01000001.1 | 100 | 801 | 0 | 0 | 30046 | 29246 |
| FINY01000001.1 | 100 | 801 | 0 | 0 | 349610 | 350410 |
| FINR01000001.1 | 100 | 801 | 0 | 0 | 30062 | 29262 |
| FILZ01000001.1 | 100 | 801 | 0 | 0 | 276168 | 276968 |
| FILG01000006.1 | 100 | 801 | 0 | 0 | 117615 | 118415 |
| FIJJ01000002.1 | 100 | 801 | 0 | 0 | 271234 | 272034 |
| FISG01000002.1 | 100 | 801 | 0 | 0 | 30063 | 29263 |
| FIRH01000001.1 | 100 | 801 | 0 | 0 | 349620 | 350420 |
| FILY01000003.1 | 100 | 801 | 0 | 0 | 257980 | 258780 |
| FIJR01000002.1 | 100 | 801 | 0 | 0 | 271030 | 271830 |
| FIHC01000002.1 | 100 | 801 | 0 | 0 | 259928 | 260728 |
| FIGY01000002.1 | 100 | 801 | 0 | 0 | 258692 | 259492 |
| FIGX01000001.1 | 100 | 801 | 0 | 0 | 357988 | 358788 |
| FIFK01000001.1 | 100 | 801 | 0 | 0 | 412843 | 413643 |
| FITA01000001.1 | 100 | 801 | 0 | 0 | 30048 | 29248 |
| FIRU01000005.1 | 100 | 801 | 0 | 0 | 30722 | 29922 |
| FIRT01000002.1 | 100 | 801 | 0 | 0 | 349622 | 350422 |
| FIQZ01000001.1 | 100 | 801 | 0 | 0 | 30270 | 29470 |
| FIPQ01000002.1 | 100 | 801 | 0 | 0 | 343962 | 344762 |
| FIPD01000002.1 | 100 | 801 | 0 | 0 | 30061 | 29261 |
| FIFC01000002.1 | 100 | 801 | 0 | 0 | 30035 | 29235 |
| FIFB01000002.1 | 100 | 801 | 0 | 0 | 30026 | 29226 |
| FIND01000002.1 | 100 | 801 | 0 | 0 | 410384 | 411184 |
| FIMX01000002.1 | 100 | 801 | 0 | 0 | 270217 | 271017 |
| FIJI01000001.1 | 100 | 801 | 0 | 0 | 341186 | 341986 |
| FISZ01000001.1 | 100 | 801 | 0 | 0 | 30061 | 29261 |
| FIMZ01000002.1 | 100 | 801 | 0 | 0 | 271114 | 271914 |
| FIPZ01000001.1 | 100 | 801 | 0 | 0 | 30275 | 29475 |
| FIPI01000001.1 | 100 | 801 | 0 | 0 | 338313 | 339113 |
| FIPE01000006.1 | 100 | 801 | 0 | 0 | 30047 | 29247 |
| FINK01000001.1 | 100 | 801 | 0 | 0 | 30061 | 29261 |
| FITH01000005.1 | 100 | 801 | 0 | 0 | 35454 | 34654 |
| FIRA01000001.1 | 100 | 801 | 0 | 0 | 354669 | 355469 |
| FIPY01000001.1 | 100 | 801 | 0 | 0 | 343967 | 344767 |
| FIPU01000002.1 | 100 | 801 | 0 | 0 | 270891 | 271691 |
| FINX01000002.1 | 100 | 801 | 0 | 0 | 30060 | 29260 |
| FINW01000003.1 | 100 | 801 | 0 | 0 | 30049 | 29249 |
| FIMC01000006.1 | 100 | 801 | 0 | 0 | 83475 | 84275 |
| FILE01000001.1 | 100 | 801 | 0 | 0 | 259920 | 260720 |
| FIQT01000002.1 | 100 | 801 | 0 | 0 | 271130 | 271930 |
| FIQC01000007.1 | 100 | 801 | 0 | 0 | 30048 | 29248 |
| FIQE01000002.1 | 100 | 801 | 0 | 0 | 338324 | 339124 |
| FIOY01000008.1 | 100 | 801 | 0 | 0 | 30262 | 29462 |
| FIOX01000002.1 | 100 | 801 | 0 | 0 | 30061 | 29261 |
| FIRQ01000001.1 | 100 | 801 | 0 | 0 | 271118 | 271918 |
| FIRD01000001.1 | 100 | 801 | 0 | 0 | 349610 | 350410 |
| FIOW01000001.1 | 100 | 801 | 0 | 0 | 30054 | 29254 |
| FIOT01000002.1 | 100 | 801 | 0 | 0 | 30061 | 29261 |
| FIOL01000001.1 | 100 | 801 | 0 | 0 | 338311 | 339111 |
| FIKV01000002.1 | 100 | 801 | 0 | 0 | 259925 | 260725 |
| FIGI01000002.1 | 100 | 801 | 0 | 0 | 258686 | 259486 |
| FILN01000001.1 | 100 | 801 | 0 | 0 | 286531 | 287331 |
| FIQW01000001.1 | 100 | 801 | 0 | 0 | 349621 | 350421 |
| FIKR01000002.1 | 100 | 801 | 0 | 0 | 271031 | 271831 |
| FISY01000001.1 | 100 | 801 | 0 | 0 | 30061 | 29261 |
| FIRR01000001.1 | 100 | 801 | 0 | 0 | 349621 | 350421 |
| FIQF01000001.1 | 100 | 801 | 0 | 0 | 349696 | 350496 |
| FIOV01000002.1 | 100 | 801 | 0 | 0 | 271302 | 272102 |
| FILV01000002.1 | 100 | 801 | 0 | 0 | 276354 | 277154 |
| FIKQ01000003.1 | 100 | 801 | 0 | 0 | 271034 | 271834 |
| FIFV01000001.1 | 100 | 801 | 0 | 0 | 271224 | 272024 |
| FIOQ01000001.1 | 100 | 801 | 0 | 0 | 30061 | 29261 |
| FIMO01000001.1 | 100 | 801 | 0 | 0 | 343970 | 344770 |
| FILW01000001.1 | 100 | 801 | 0 | 0 | 271158 | 271958 |
| FILP01000002.1 | 100 | 801 | 0 | 0 | 270766 | 271566 |
| FIKY01000006.1 | 100 | 801 | 0 | 0 | 114998 | 115798 |
| FIJY01000002.1 | 100 | 801 | 0 | 0 | 329956 | 330756 |
| FIST01000004.1 | 100 | 801 | 0 | 0 | 154001 | 154801 |
| FISP01000001.1 | 100 | 801 | 0 | 0 | 30053 | 29253 |
| FIRI01000002.1 | 100 | 801 | 0 | 0 | 338308 | 339108 |
| FINQ01000001.1 | 100 | 801 | 0 | 0 | 349669 | 350469 |
| FIMY01000001.1 | 100 | 801 | 0 | 0 | 338297 | 339097 |
| FIMU01000002.1 | 100 | 801 | 0 | 0 | 259976 | 260776 |
| FIMT01000001.1 | 100 | 801 | 0 | 0 | 343899 | 344699 |
| FIMI01000002.1 | 100 | 801 | 0 | 0 | 30053 | 29253 |
| FIKB01000001.1 | 100 | 801 | 0 | 0 | 341744 | 342544 |
| FISX01000005.1 | 100 | 801 | 0 | 0 | 30062 | 29262 |
| FISM01000001.1 | 100 | 801 | 0 | 0 | 349702 | 350502 |
| FISL01000002.1 | 100 | 801 | 0 | 0 | 271420 | 272220 |
| FIMN01000002.1 | 100 | 801 | 0 | 0 | 30062 | 29262 |
| FIJF01000003.1 | 100 | 801 | 0 | 0 | 258676 | 259476 |
| FIJC01000002.1 | 100 | 801 | 0 | 0 | 271158 | 271958 |
| FISR01000001.1 | 100 | 801 | 0 | 0 | 349704 | 350504 |
| FIML01000002.1 | 100 | 801 | 0 | 0 | 271249 | 272049 |
| FISU01000001.1 | 100 | 801 | 0 | 0 | 30062 | 29262 |
| FISN01000002.1 | 100 | 801 | 0 | 0 | 259755 | 260555 |
| FIRB01000001.1 | 100 | 801 | 0 | 0 | 30060 | 29260 |
| FIQX01000001.1 | 100 | 801 | 0 | 0 | 338043 | 338843 |
| FINB01000001.1 | 100 | 801 | 0 | 0 | 30776 | 29976 |
| FINA01000001.1 | 100 | 801 | 0 | 0 | 30062 | 29262 |
| FIMS01000001.1 | 100 | 801 | 0 | 0 | 30054 | 29254 |
| FIMM01000001.1 | 100 | 801 | 0 | 0 | 30062 | 29262 |
| CZGR01000020.1 | 100 | 801 | 0 | 0 | 69766 | 70566 |
| CZGI01000031.1 | 100 | 801 | 0 | 0 | 271522 | 272322 |
| CZEQ01000036.1 | 100 | 801 | 0 | 0 | 271226 | 272026 |
| CZET01000039.1 | 100 | 801 | 0 | 0 | 182070 | 182870 |
| CZGU01000033.1 | 100 | 801 | 0 | 0 | 259949 | 260749 |
| CZGM01000034.1 | 100 | 801 | 0 | 0 | 271225 | 272025 |
| CZDT01000033.1 | 100 | 801 | 0 | 0 | 271150 | 271950 |
| CZDM01000013.1 | 100 | 801 | 0 | 0 | 30289 | 29489 |
| CZDX01000003.1 | 100 | 801 | 0 | 0 | 7781 | 8581 |
| CZFP01000010.1 | 100 | 801 | 0 | 0 | 34753 | 33953 |
| CZDQ01000035.1 | 100 | 801 | 0 | 0 | 271225 | 272025 |
| CZDV01000035.1 | 100 | 801 | 0 | 0 | 259945 | 260745 |
| CZEU01000038.1 | 100 | 801 | 0 | 0 | 329390 | 330190 |
| CZGZ01000040.1 | 100 | 801 | 0 | 0 | 271553 | 272353 |
| CZDK01000037.1 | 100 | 801 | 0 | 0 | 269425 | 270225 |
| CZDR01000036.1 | 100 | 801 | 0 | 0 | 30289 | 29489 |
| CZFK01000013.1 | 100 | 801 | 0 | 0 | 34753 | 33953 |
| CZFA01000034.1 | 100 | 801 | 0 | 0 | 271527 | 272327 |
| CZFB01000032.1 | 100 | 801 | 0 | 0 | 271225 | 272025 |
| CZEI01000035.1 | 100 | 801 | 0 | 0 | 30289 | 29489 |
| CZDL01000036.1 | 100 | 801 | 0 | 0 | 270551 | 271351 |
| CZFL01000034.1 | 100 | 801 | 0 | 0 | 30289 | 29489 |
| CZHD01000039.1 | 100 | 801 | 0 | 0 | 271523 | 272323 |
| CZEG01000034.1 | 100 | 801 | 0 | 0 | 271493 | 272293 |
| CZEA01000038.1 | 100 | 801 | 0 | 0 | 259973 | 260773 |
| CZHC01000044.1 | 100 | 801 | 0 | 0 | 271552 | 272352 |
| CZFU01000041.1 | 100 | 801 | 0 | 0 | 335363 | 336163 |
| CZFH01000003.1 | 100 | 801 | 0 | 0 | 30034 | 29234 |
| CZGX01000046.1 | 100 | 801 | 0 | 0 | 30289 | 29489 |
| CZGA01000034.1 | 100 | 801 | 0 | 0 | 259946 | 260746 |
| CZFO01000035.1 | 100 | 801 | 0 | 0 | 260244 | 261044 |
| CZFG01000036.1 | 100 | 801 | 0 | 0 | 271523 | 272323 |
| CZEO01000033.1 | 100 | 801 | 0 | 0 | 260247 | 261047 |
| CZEW01000034.1 | 100 | 801 | 0 | 0 | 260273 | 261073 |
| CZEM01000020.1 | 100 | 801 | 0 | 0 | 34753 | 33953 |
| CZER01000041.1 | 100 | 801 | 0 | 0 | 346209 | 347009 |
| CZDO01000033.1 | 100 | 801 | 0 | 0 | 259947 | 260747 |
| JASTSK010000001.1 | 100 | 801 | 0 | 0 | 30091 | 29291 |
| JASTSL010000001.1 | 100 | 801 | 0 | 0 | 30084 | 29284 |
| JASTSJ010000001.1 | 100 | 801 | 0 | 0 | 30085 | 29285 |
| JASTSI010000001.1 | 100 | 801 | 0 | 0 | 30084 | 29284 |
| JASTSH010000001.1 | 100 | 801 | 0 | 0 | 30084 | 29284 |
| JASTSG010000001.1 | 100 | 801 | 0 | 0 | 30084 | 29284 |
| JASTSD010000002.1 | 100 | 801 | 0 | 0 | 30084 | 29284 |
| JASTSF010000007.1 | 100 | 801 | 0 | 0 | 65428 | 66228 |
| JASTSE010000001.1 | 100 | 801 | 0 | 0 | 30355 | 29555 |
| JASTSC010000001.1 | 100 | 801 | 0 | 0 | 30085 | 29285 |
| JASTSB010000007.1 | 100 | 801 | 0 | 0 | 30305 | 29505 |
| JASTRZ010000001.1 | 100 | 801 | 0 | 0 | 30091 | 29291 |
| JASTSA010000005.1 | 100 | 801 | 0 | 0 | 30305 | 29505 |
| JASTRY010000002.1 | 100 | 801 | 0 | 0 | 30084 | 29284 |
| JASTRX010000001.1 | 100 | 801 | 0 | 0 | 30091 | 29291 |
| JASTRW010000001.1 | 100 | 801 | 0 | 0 | 30488 | 29688 |
| JASTRU010000001.1 | 100 | 801 | 0 | 0 | 30296 | 29496 |
| JASTRS010000001.1 | 100 | 801 | 0 | 0 | 30084 | 29284 |
| JASTRV010000001.1 | 100 | 801 | 0 | 0 | 30296 | 29496 |
| JASTRT010000001.1 | 100 | 801 | 0 | 0 | 30668 | 29868 |
| JASTRQ010000001.1 | 100 | 801 | 0 | 0 | 30090 | 29290 |
| JASTRP010000001.1 | 100 | 801 | 0 | 0 | 30084 | 29284 |
| JASTRR010000001.1 | 100 | 801 | 0 | 0 | 30296 | 29496 |
| JASTRO010000001.1 | 100 | 801 | 0 | 0 | 30084 | 29284 |
| JASTRN010000001.1 | 100 | 801 | 0 | 0 | 35736 | 34936 |
| JASTRM010000001.1 | 100 | 801 | 0 | 0 | 395135 | 395935 |
| JASTRL010000001.1 | 100 | 801 | 0 | 0 | 30091 | 29291 |
| JASTRK010000001.1 | 100 | 801 | 0 | 0 | 35736 | 34936 |
| JASTRJ010000001.1 | 100 | 801 | 0 | 0 | 30303 | 29503 |
| JASTRI010000001.1 | 100 | 801 | 0 | 0 | 30084 | 29284 |
| JASTRH010000001.1 | 100 | 801 | 0 | 0 | 30519 | 29719 |
| JASTRG010000001.1 | 100 | 801 | 0 | 0 | 30084 | 29284 |
| JASTRF010000001.1 | 100 | 801 | 0 | 0 | 30296 | 29496 |
| JASTRE010000001.1 | 100 | 801 | 0 | 0 | 331813 | 332613 |
| JASTRC010000001.1 | 100 | 801 | 0 | 0 | 30091 | 29291 |
| JASTRD010000001.1 | 100 | 801 | 0 | 0 | 30084 | 29284 |
| JASTRB010000006.1 | 100 | 801 | 0 | 0 | 66265 | 67065 |
| JASTRA010000001.1 | 100 | 801 | 0 | 0 | 30296 | 29496 |
| JASTQX010000001.1 | 100 | 801 | 0 | 0 | 30084 | 29284 |
| JASTQW010000001.1 | 100 | 801 | 0 | 0 | 30084 | 29284 |
| JASTQZ010000001.1 | 100 | 801 | 0 | 0 | 45871 | 45071 |
| JASTQY010000001.1 | 100 | 801 | 0 | 0 | 30084 | 29284 |
| JASTQU010000001.1 | 100 | 801 | 0 | 0 | 30084 | 29284 |
| JASTQR010000001.1 | 100 | 801 | 0 | 0 | 30091 | 29291 |
| JASTQV010000001.1 | 100 | 801 | 0 | 0 | 30296 | 29496 |
| JASTQT010000002.1 | 100 | 801 | 0 | 0 | 30296 | 29496 |
| JASTQS010000001.1 | 100 | 801 | 0 | 0 | 35523 | 34723 |
| JASTQP010000001.1 | 100 | 801 | 0 | 0 | 30091 | 29291 |
| JASTQQ010000002.1 | 100 | 801 | 0 | 0 | 271256 | 272056 |
| JASTQN010000001.1 | 100 | 801 | 0 | 0 | 410473 | 411273 |
| JASTQM010000002.1 | 100 | 801 | 0 | 0 | 30305 | 29505 |
| JASTQO010000017.1 | 100 | 801 | 0 | 0 | 30084 | 29284 |
| JASTQL010000001.1 | 100 | 801 | 0 | 0 | 30668 | 29868 |
| JASTQK010000001.1 | 100 | 801 | 0 | 0 | 30093 | 29293 |
| JASTQJ010000002.1 | 100 | 801 | 0 | 0 | 30084 | 29284 |
| JASTQH010000001.1 | 100 | 801 | 0 | 0 | 332138 | 332938 |
| JASTQI010000001.1 | 100 | 801 | 0 | 0 | 46033 | 45233 |
| JASTQG010000001.1 | 100 | 801 | 0 | 0 | 404843 | 405643 |
| JASTQF010000001.1 | 100 | 801 | 0 | 0 | 338921 | 339721 |
| JASTQD010000001.1 | 100 | 801 | 0 | 0 | 45821 | 45021 |
| JASTQB010000004.1 | 100 | 801 | 0 | 0 | 30084 | 29284 |
| JASTQC010000001.1 | 100 | 801 | 0 | 0 | 30296 | 29496 |
| JASTPY010000001.1 | 100 | 801 | 0 | 0 | 35736 | 34936 |
| JASTQA010000001.1 | 100 | 801 | 0 | 0 | 30084 | 29284 |
| JASTPZ010000001.1 | 100 | 801 | 0 | 0 | 30288 | 29488 |
| JASTPX010000001.1 | 100 | 801 | 0 | 0 | 30084 | 29284 |
| JASTPU010000001.1 | 100 | 801 | 0 | 0 | 30084 | 29284 |
| JASTPT010000002.1 | 100 | 801 | 0 | 0 | 30084 | 29284 |
| JASTPV010000002.1 | 100 | 801 | 0 | 0 | 271472 | 272272 |
| JASTPW010000002.1 | 100 | 801 | 0 | 0 | 30084 | 29284 |
| JASTPS010000002.1 | 100 | 801 | 0 | 0 | 271252 | 272052 |
| JASTPR010000006.1 | 100 | 801 | 0 | 0 | 35628 | 34828 |
| JBCLWR010000011.1 | 100 | 801 | 0 | 0 | 51310 | 52110 |
| JAVIGK010000004.1 | 100 | 801 | 0 | 0 | 69766 | 70566 |
| JAVIGJ010000001.1 | 100 | 801 | 0 | 0 | 331965 | 332765 |
| JAVIGH010000004.1 | 100 | 801 | 0 | 0 | 69791 | 70591 |
| JAVIGG010000005.1 | 100 | 801 | 0 | 0 | 69791 | 70591 |
| JAVIGM010000001.1 | 100 | 801 | 0 | 0 | 331710 | 332510 |
| JAVIGL010000001.1 | 100 | 801 | 0 | 0 | 331730 | 332530 |
| JAVIGC010000005.1 | 100 | 801 | 0 | 0 | 15100 | 14300 |
| JAVIGE010000005.1 | 100 | 801 | 0 | 0 | 15080 | 14280 |
| JAVIGD010000005.1 | 100 | 801 | 0 | 0 | 69834 | 70634 |
| JAVIGF010000005.1 | 100 | 801 | 0 | 0 | 15080 | 14280 |
| CP141904.1 | 100 | 801 | 0 | 0 | 1000123 | 999323 |
| DATSUD010000001.1 | 100 | 801 | 0 | 0 | 30168 | 29368 |
| DATSRX010000001.1 | 100 | 801 | 0 | 0 | 29950 | 29150 |
| CP139881.1 | 100 | 801 | 0 | 0 | 1860283 | 1861083 |
| CP139880.1 | 100 | 801 | 0 | 0 | 209710 | 208910 |
| CP139879.1 | 100 | 801 | 0 | 0 | 209490 | 208690 |
| JAXKWL010000001.1 | 100 | 801 | 0 | 0 | 1038476 | 1039276 |
| DASGYX010000001.1 | 100 | 801 | 0 | 0 | 34600 | 33800 |
| DASGYV010000002.1 | 100 | 801 | 0 | 0 | 265601 | 266401 |
| DASGYU010000001.1 | 100 | 801 | 0 | 0 | 342520 | 343320 |
| DASGYS010000001.1 | 100 | 801 | 0 | 0 | 338194 | 338994 |
| DASGYQ010000001.1 | 100 | 801 | 0 | 0 | 335339 | 336139 |
| DASGYP010000001.1 | 100 | 801 | 0 | 0 | 29956 | 29156 |
| DASGYM010000001.1 | 100 | 801 | 0 | 0 | 335339 | 336139 |
| DASGYN010000001.1 | 100 | 801 | 0 | 0 | 335339 | 336139 |
| DASGYH010000001.1 | 100 | 801 | 0 | 0 | 29956 | 29156 |
| DASGYF010000001.1 | 100 | 801 | 0 | 0 | 335339 | 336139 |
| DASGXW010000001.1 | 100 | 801 | 0 | 0 | 29956 | 29156 |
| DASGXP010000001.1 | 100 | 801 | 0 | 0 | 335226 | 336026 |
| DASGXJ010000002.1 | 100 | 801 | 0 | 0 | 260066 | 260866 |
| DASGXD010000001.1 | 100 | 801 | 0 | 0 | 30164 | 29364 |
| DASGXC010000001.1 | 100 | 801 | 0 | 0 | 30168 | 29368 |
| DASGXB010000001.1 | 100 | 801 | 0 | 0 | 342487 | 343287 |
| DASGXA010000002.1 | 100 | 801 | 0 | 0 | 271344 | 272144 |
| DASGWZ010000001.1 | 100 | 801 | 0 | 0 | 29956 | 29156 |
| DASGWX010000002.1 | 100 | 801 | 0 | 0 | 265908 | 266708 |
| DASGWW010000001.1 | 100 | 801 | 0 | 0 | 29956 | 29156 |
| DASGWT010000004.1 | 100 | 801 | 0 | 0 | 29956 | 29156 |
| DASGWS010000001.1 | 100 | 801 | 0 | 0 | 29956 | 29156 |
| DASGWU010000005.1 | 100 | 801 | 0 | 0 | 94524 | 95324 |
| DASGWR010000001.1 | 100 | 801 | 0 | 0 | 264550 | 265350 |
| DASGWQ010000001.1 | 100 | 801 | 0 | 0 | 29956 | 29156 |
| DASGWP010000002.1 | 100 | 801 | 0 | 0 | 259911 | 260711 |
| DASGWO010000001.1 | 100 | 801 | 0 | 0 | 276899 | 277699 |
| DASGWM010000001.1 | 100 | 801 | 0 | 0 | 332233 | 333033 |
| DASGWN010000006.1 | 100 | 801 | 0 | 0 | 95792 | 96592 |
| DASGWL010000001.1 | 100 | 801 | 0 | 0 | 276924 | 277724 |
| DASGWK010000001.1 | 100 | 801 | 0 | 0 | 112753 | 111953 |
| DASGWJ010000001.1 | 100 | 801 | 0 | 0 | 342462 | 343262 |
| DASGWH010000001.1 | 100 | 801 | 0 | 0 | 112753 | 111953 |
| DASGWI010000005.1 | 100 | 801 | 0 | 0 | 94535 | 95335 |
| DASGWG010000002.1 | 100 | 801 | 0 | 0 | 265601 | 266401 |
| DASGWF010000001.1 | 100 | 801 | 0 | 0 | 30255 | 29455 |
| DASGWB010000001.1 | 100 | 801 | 0 | 0 | 29956 | 29156 |
| DASGRX010000002.1 | 100 | 801 | 0 | 0 | 35292 | 34492 |
| DASGRV010000002.1 | 100 | 801 | 0 | 0 | 35292 | 34492 |
| DASGMQ010000001.1 | 100 | 801 | 0 | 0 | 29956 | 29156 |
| DASGMS010000001.1 | 100 | 801 | 0 | 0 | 29956 | 29156 |
| DASGMO010000001.1 | 100 | 801 | 0 | 0 | 29956 | 29156 |
| DASGMP010000001.1 | 100 | 801 | 0 | 0 | 29956 | 29156 |
| DASGMR010000001.1 | 100 | 801 | 0 | 0 | 29956 | 29156 |
| DASGMT010000001.1 | 100 | 801 | 0 | 0 | 29956 | 29156 |
| DASGMN010000001.1 | 100 | 801 | 0 | 0 | 29956 | 29156 |
| DASGMJ010000001.1 | 100 | 801 | 0 | 0 | 29956 | 29156 |
| DASGMM010000001.1 | 100 | 801 | 0 | 0 | 29956 | 29156 |
| DASGMI010000001.1 | 100 | 801 | 0 | 0 | 29956 | 29156 |
| DASGMH010000001.1 | 100 | 801 | 0 | 0 | 29956 | 29156 |
| DASGMK010000001.1 | 100 | 801 | 0 | 0 | 29956 | 29156 |
| DASGMG010000001.1 | 100 | 801 | 0 | 0 | 29956 | 29156 |
| DASGML010000001.1 | 100 | 801 | 0 | 0 | 29956 | 29156 |
| DASGME010000001.1 | 100 | 801 | 0 | 0 | 29956 | 29156 |
| DASGLZ010000001.1 | 100 | 801 | 0 | 0 | 29956 | 29156 |
| DASGLW010000001.1 | 100 | 801 | 0 | 0 | 29956 | 29156 |
| DASGMB010000001.1 | 100 | 801 | 0 | 0 | 29956 | 29156 |
| DASGMD010000001.1 | 100 | 801 | 0 | 0 | 29956 | 29156 |
| DASGLX010000001.1 | 100 | 801 | 0 | 0 | 29956 | 29156 |
| DASGLY010000001.1 | 100 | 801 | 0 | 0 | 29956 | 29156 |
| DASGMA010000001.1 | 100 | 801 | 0 | 0 | 29956 | 29156 |
| DASGMF010000001.1 | 100 | 801 | 0 | 0 | 29956 | 29156 |
| DASGMC010000001.1 | 100 | 801 | 0 | 0 | 29956 | 29156 |
| DASGLV010000001.1 | 100 | 801 | 0 | 0 | 29956 | 29156 |
| DASGLT010000001.1 | 100 | 801 | 0 | 0 | 29956 | 29156 |
| DASGLU010000001.1 | 100 | 801 | 0 | 0 | 29956 | 29156 |
| DASGLQ010000001.1 | 100 | 801 | 0 | 0 | 29956 | 29156 |
| DASGLR010000001.1 | 100 | 801 | 0 | 0 | 29956 | 29156 |
| DASGLS010000001.1 | 100 | 801 | 0 | 0 | 29956 | 29156 |
| DASGLP010000002.1 | 100 | 801 | 0 | 0 | 271243 | 272043 |
| DASGLN010000001.1 | 100 | 801 | 0 | 0 | 29956 | 29156 |
| DASGLO010000001.1 | 100 | 801 | 0 | 0 | 29956 | 29156 |
| DASGLM010000001.1 | 100 | 801 | 0 | 0 | 29956 | 29156 |
| DASGLL010000001.1 | 100 | 801 | 0 | 0 | 29956 | 29156 |
| DASGLK010000001.1 | 100 | 801 | 0 | 0 | 29956 | 29156 |
| DASGLJ010000001.1 | 100 | 801 | 0 | 0 | 29956 | 29156 |
| DASGLH010000001.1 | 100 | 801 | 0 | 0 | 29956 | 29156 |
| DASGLI010000001.1 | 100 | 801 | 0 | 0 | 29956 | 29156 |
| DASGLG010000001.1 | 100 | 801 | 0 | 0 | 29956 | 29156 |
| DASGLF010000001.1 | 100 | 801 | 0 | 0 | 29956 | 29156 |
| DASGLE010000001.1 | 100 | 801 | 0 | 0 | 29956 | 29156 |
| DASGLD010000001.1 | 100 | 801 | 0 | 0 | 29956 | 29156 |
| DASGLB010000001.1 | 100 | 801 | 0 | 0 | 29956 | 29156 |
| DASGLC010000001.1 | 100 | 801 | 0 | 0 | 29956 | 29156 |
| DASGLA010000001.1 | 100 | 801 | 0 | 0 | 29956 | 29156 |
| DASGKZ010000001.1 | 100 | 801 | 0 | 0 | 29956 | 29156 |
| DASGKX010000001.1 | 100 | 801 | 0 | 0 | 29956 | 29156 |
| DASGKY010000001.1 | 100 | 801 | 0 | 0 | 29956 | 29156 |
| DASGKW010000001.1 | 100 | 801 | 0 | 0 | 29956 | 29156 |
| DASGKV010000001.1 | 100 | 801 | 0 | 0 | 29956 | 29156 |
| DASGKT010000001.1 | 100 | 801 | 0 | 0 | 29956 | 29156 |
| DASGKU010000001.1 | 100 | 801 | 0 | 0 | 29956 | 29156 |
| DASGKS010000001.1 | 100 | 801 | 0 | 0 | 29956 | 29156 |
| DASGKR010000001.1 | 100 | 801 | 0 | 0 | 29956 | 29156 |
| DASGKQ010000001.1 | 100 | 801 | 0 | 0 | 29956 | 29156 |
| DASGKP010000001.1 | 100 | 801 | 0 | 0 | 29956 | 29156 |
| DASGKN010000001.1 | 100 | 801 | 0 | 0 | 29956 | 29156 |
| DASGKO010000001.1 | 100 | 801 | 0 | 0 | 29956 | 29156 |
| DASGKK010000001.1 | 100 | 801 | 0 | 0 | 29956 | 29156 |
| DASGKL010000001.1 | 100 | 801 | 0 | 0 | 11081 | 10281 |
| DASGKM010000001.1 | 100 | 801 | 0 | 0 | 29956 | 29156 |
| DASGKH010000001.1 | 100 | 801 | 0 | 0 | 29956 | 29156 |
| DASGKJ010000013.1 | 100 | 801 | 0 | 0 | 17560 | 18360 |
| DASGKI010000001.1 | 100 | 801 | 0 | 0 | 29956 | 29156 |
| DASGKG010000001.1 | 100 | 801 | 0 | 0 | 29956 | 29156 |
| DASGKF010000001.1 | 100 | 801 | 0 | 0 | 29956 | 29156 |
| DASGKE010000001.1 | 100 | 801 | 0 | 0 | 29956 | 29156 |
| DASGKC010000001.1 | 100 | 801 | 0 | 0 | 29956 | 29156 |
| DASGKD010000001.1 | 100 | 801 | 0 | 0 | 29956 | 29156 |
| DASGJX010000001.1 | 100 | 801 | 0 | 0 | 29956 | 29156 |
| DASGKA010000001.1 | 100 | 801 | 0 | 0 | 29956 | 29156 |
| DASGKB010000001.1 | 100 | 801 | 0 | 0 | 29956 | 29156 |
| DASGJY010000001.1 | 100 | 801 | 0 | 0 | 29956 | 29156 |
| DASGJZ010000001.1 | 100 | 801 | 0 | 0 | 29956 | 29156 |
| DASGJW010000001.1 | 100 | 801 | 0 | 0 | 29956 | 29156 |
| DASGJU010000001.1 | 100 | 801 | 0 | 0 | 29956 | 29156 |
| DASGJV010000001.1 | 100 | 801 | 0 | 0 | 29956 | 29156 |
| DASGJT010000001.1 | 100 | 801 | 0 | 0 | 29956 | 29156 |
| DASGJS010000001.1 | 100 | 801 | 0 | 0 | 29956 | 29156 |
| DASGJP010000001.1 | 100 | 801 | 0 | 0 | 29956 | 29156 |
| DASGJQ010000001.1 | 100 | 801 | 0 | 0 | 29956 | 29156 |
| DASGJR010000001.1 | 100 | 801 | 0 | 0 | 29956 | 29156 |
| DASGJM010000001.1 | 100 | 801 | 0 | 0 | 29956 | 29156 |
| DASGJN010000001.1 | 100 | 801 | 0 | 0 | 29956 | 29156 |
| DASGJO010000001.1 | 100 | 801 | 0 | 0 | 29956 | 29156 |
| DASGJL010000001.1 | 100 | 801 | 0 | 0 | 29956 | 29156 |
| DASGJJ010000001.1 | 100 | 801 | 0 | 0 | 29956 | 29156 |
| DASGJI010000001.1 | 100 | 801 | 0 | 0 | 29956 | 29156 |
| DASGJK010000005.1 | 100 | 801 | 0 | 0 | 78090 | 78890 |
| DASGJH010000001.1 | 100 | 801 | 0 | 0 | 29956 | 29156 |
| DASGJG010000001.1 | 100 | 801 | 0 | 0 | 29956 | 29156 |
| DASGJF010000001.1 | 100 | 801 | 0 | 0 | 29956 | 29156 |
| DASGJD010000001.1 | 100 | 801 | 0 | 0 | 29956 | 29156 |
| DASGJC010000001.1 | 100 | 801 | 0 | 0 | 276927 | 277727 |
| DASGJB010000004.1 | 100 | 801 | 0 | 0 | 114851 | 115651 |
| DASGIY010000005.1 | 100 | 801 | 0 | 0 | 103572 | 104372 |
| DASGIW010000005.1 | 100 | 801 | 0 | 0 | 114850 | 115650 |
| DASGIT010000005.1 | 100 | 801 | 0 | 0 | 103572 | 104372 |
| DASGIS010000006.1 | 100 | 801 | 0 | 0 | 94005 | 94805 |
| DASGIO010000001.1 | 100 | 801 | 0 | 0 | 29956 | 29156 |
| DASGIM010000004.1 | 100 | 801 | 0 | 0 | 103460 | 104260 |
| DASGIN010000005.1 | 100 | 801 | 0 | 0 | 103572 | 104372 |
| DASGIK010000002.1 | 100 | 801 | 0 | 0 | 259374 | 260174 |
| DASGIG010000001.1 | 100 | 801 | 0 | 0 | 29956 | 29156 |
| DASGII010000001.1 | 100 | 801 | 0 | 0 | 29956 | 29156 |
| DASGIE010000005.1 | 100 | 801 | 0 | 0 | 114857 | 115657 |
| DASGHZ010000003.1 | 100 | 801 | 0 | 0 | 271125 | 271925 |
| DASGHY010000002.1 | 100 | 801 | 0 | 0 | 259886 | 260686 |
| DASGHR010000002.1 | 100 | 801 | 0 | 0 | 29956 | 29156 |
| DASGHP010000005.1 | 100 | 801 | 0 | 0 | 114851 | 115651 |
| DASGHQ010000005.1 | 100 | 801 | 0 | 0 | 114851 | 115651 |
| DASGHM010000004.1 | 100 | 801 | 0 | 0 | 114851 | 115651 |
| DASGHG010000004.1 | 100 | 801 | 0 | 0 | 136188 | 136988 |
| DASGHC010000005.1 | 100 | 801 | 0 | 0 | 112872 | 113672 |
| DASGGX010000002.1 | 100 | 801 | 0 | 0 | 259867 | 260667 |
| DASGHA010000002.1 | 100 | 801 | 0 | 0 | 265399 | 266199 |
| DASGGV010000002.1 | 100 | 801 | 0 | 0 | 271176 | 271976 |
| DASGGW010000003.1 | 100 | 801 | 0 | 0 | 166258 | 167058 |
| DASGGU010000002.1 | 100 | 801 | 0 | 0 | 259867 | 260667 |
| DASGGO010000002.1 | 100 | 801 | 0 | 0 | 270894 | 271694 |
| DASGGM010000002.1 | 100 | 801 | 0 | 0 | 259644 | 260444 |
| DASGGH010000005.1 | 100 | 801 | 0 | 0 | 94004 | 94804 |
| DASGGF010000005.1 | 100 | 801 | 0 | 0 | 114851 | 115651 |
| DASGGD010000005.1 | 100 | 801 | 0 | 0 | 114851 | 115651 |
| DASGGB010000005.1 | 100 | 801 | 0 | 0 | 114851 | 115651 |
| DASGFW010000005.1 | 100 | 801 | 0 | 0 | 114851 | 115651 |
| DASGFV010000001.1 | 100 | 801 | 0 | 0 | 29956 | 29156 |
| DASGFS010000002.1 | 100 | 801 | 0 | 0 | 259867 | 260667 |
| DASGFO010000002.1 | 100 | 801 | 0 | 0 | 30112 | 29312 |
| DASGFP010000001.1 | 100 | 801 | 0 | 0 | 29956 | 29156 |
| DASGFM010000005.1 | 100 | 801 | 0 | 0 | 114851 | 115651 |
| DASGFI010000005.1 | 100 | 801 | 0 | 0 | 114851 | 115651 |
| DASGFJ010000005.1 | 100 | 801 | 0 | 0 | 114739 | 115539 |
| DASGFG010000001.1 | 100 | 801 | 0 | 0 | 321894 | 322694 |
| DASGDL010000002.1 | 100 | 801 | 0 | 0 | 34871 | 34071 |
| DASGDK010000002.1 | 100 | 801 | 0 | 0 | 34821 | 34021 |
| DASGDC010000002.1 | 100 | 801 | 0 | 0 | 271176 | 271976 |
| DASFUV010000002.1 | 100 | 801 | 0 | 0 | 271361 | 272161 |
| DASFUA010000001.1 | 100 | 801 | 0 | 0 | 241289 | 242089 |
| DASFTW010000003.1 | 100 | 801 | 0 | 0 | 124450 | 125250 |
| DASFTZ010000002.1 | 100 | 801 | 0 | 0 | 118073 | 118873 |
| DASFTY010000003.1 | 100 | 801 | 0 | 0 | 109978 | 110778 |
| DASFTX010000014.1 | 100 | 801 | 0 | 0 | 24755 | 25555 |
| DASFTU010000014.1 | 100 | 801 | 0 | 0 | 25711 | 26511 |
| DASFQT010000004.1 | 100 | 801 | 0 | 0 | 123598 | 124398 |
| DASFQA010000001.1 | 100 | 801 | 0 | 0 | 144329 | 145129 |
| DASFPW010000001.1 | 100 | 801 | 0 | 0 | 302406 | 303206 |
| DASFPT010000001.1 | 100 | 801 | 0 | 0 | 302409 | 303209 |
| DASFPB010000005.1 | 100 | 801 | 0 | 0 | 90856 | 91656 |
| DASFPA010000013.1 | 100 | 801 | 0 | 0 | 14397 | 15197 |
| DASFNV010000001.1 | 100 | 801 | 0 | 0 | 29956 | 29156 |
| DASFNP010000002.1 | 100 | 801 | 0 | 0 | 259649 | 260449 |
| DASFNM010000023.1 | 100 | 801 | 0 | 0 | 14357 | 15157 |
| DASFMR010000011.1 | 100 | 801 | 0 | 0 | 13391 | 14191 |
| DASFMO010000019.1 | 100 | 801 | 0 | 0 | 29956 | 29156 |
| DASFMA010000004.1 | 100 | 801 | 0 | 0 | 88650 | 89450 |
| DASFLN010000002.1 | 100 | 801 | 0 | 0 | 209052 | 209852 |
| DASFLF010000006.1 | 100 | 801 | 0 | 0 | 98125 | 98925 |
| DASFKY010000010.1 | 100 | 801 | 0 | 0 | 34147 | 34947 |
| DASFKX010000002.1 | 100 | 801 | 0 | 0 | 35397 | 34597 |
| DASFIT010000010.1 | 100 | 801 | 0 | 0 | 34147 | 34947 |
| DASFII010000016.1 | 100 | 801 | 0 | 0 | 36571 | 35771 |
| DASFIH010000020.1 | 100 | 801 | 0 | 0 | 30501 | 29701 |
| DASFGH010000020.1 | 100 | 801 | 0 | 0 | 30004 | 29204 |
| DASFFM010000002.1 | 100 | 801 | 0 | 0 | 106076 | 106876 |
| DASFFF010000005.1 | 100 | 801 | 0 | 0 | 92332 | 93132 |
| DASFFG010000007.1 | 100 | 801 | 0 | 0 | 74275 | 75075 |
| DASFFD010000001.1 | 100 | 801 | 0 | 0 | 259970 | 260770 |
| DASFFC010000011.1 | 100 | 801 | 0 | 0 | 34145 | 34945 |
| DASFEY010000006.1 | 100 | 801 | 0 | 0 | 82082 | 82882 |
| DASFFA010000004.1 | 100 | 801 | 0 | 0 | 82081 | 82881 |
| DASEZY010000001.1 | 100 | 801 | 0 | 0 | 29956 | 29156 |
| DASEZV010000008.1 | 100 | 801 | 0 | 0 | 34122 | 34922 |
| DASEZX010000019.1 | 100 | 801 | 0 | 0 | 30443 | 29643 |
| DASEZW010000001.1 | 100 | 801 | 0 | 0 | 29956 | 29156 |
| DASEZU010000001.1 | 100 | 801 | 0 | 0 | 29956 | 29156 |
| DASEZQ010000013.1 | 100 | 801 | 0 | 0 | 34148 | 34948 |
| DASEZT010000001.1 | 100 | 801 | 0 | 0 | 29956 | 29156 |
| DASEZP010000001.1 | 100 | 801 | 0 | 0 | 29956 | 29156 |
| DASELA010000002.1 | 100 | 801 | 0 | 0 | 271324 | 272124 |
| DASEYN010000017.1 | 100 | 801 | 0 | 0 | 34212 | 35012 |
| DASEYM010000001.1 | 100 | 801 | 0 | 0 | 30220 | 29420 |
| DASEYF010000001.1 | 100 | 801 | 0 | 0 | 29956 | 29156 |
| DASEYE010000014.1 | 100 | 801 | 0 | 0 | 29956 | 29156 |
| DASEVN010000014.1 | 100 | 801 | 0 | 0 | 8015 | 8815 |
| DASEVM010000013.1 | 100 | 801 | 0 | 0 | 14399 | 15199 |
| DASEVL010000013.1 | 100 | 801 | 0 | 0 | 29956 | 29156 |
| DASEPB010000027.1 | 100 | 801 | 0 | 0 | 20870 | 20070 |
| DASENV010000001.1 | 100 | 801 | 0 | 0 | 29956 | 29156 |
| DASENS010000006.1 | 100 | 801 | 0 | 0 | 52538 | 53338 |
| DASENM010000002.1 | 100 | 801 | 0 | 0 | 148045 | 148845 |
| DASELQ010000005.1 | 100 | 801 | 0 | 0 | 94524 | 95324 |
| DASELS010000002.1 | 100 | 801 | 0 | 0 | 260066 | 260866 |
| DASELU010000004.1 | 100 | 801 | 0 | 0 | 94524 | 95324 |
| DASELL010000002.1 | 100 | 801 | 0 | 0 | 260066 | 260866 |
| DASELT010000002.1 | 100 | 801 | 0 | 0 | 260070 | 260870 |
| DASELO010000002.1 | 100 | 801 | 0 | 0 | 257440 | 258240 |
| DASELR010000005.1 | 100 | 801 | 0 | 0 | 94524 | 95324 |
| DASELN010000002.1 | 100 | 801 | 0 | 0 | 260066 | 260866 |
| DASELP010000007.1 | 100 | 801 | 0 | 0 | 68650 | 69450 |
| DASELM010000001.1 | 100 | 801 | 0 | 0 | 342487 | 343287 |
| DASELK010000005.1 | 100 | 801 | 0 | 0 | 106257 | 107057 |
| DASELJ010000003.1 | 100 | 801 | 0 | 0 | 193256 | 194056 |
| DASELF010000001.1 | 100 | 801 | 0 | 0 | 276902 | 277702 |
| DASELH010000002.1 | 100 | 801 | 0 | 0 | 271343 | 272143 |
| DASELI010000002.1 | 100 | 801 | 0 | 0 | 35291 | 34491 |
| DASELG010000002.1 | 100 | 801 | 0 | 0 | 271345 | 272145 |
| DASELD010000004.1 | 100 | 801 | 0 | 0 | 124936 | 125736 |
| DASELC010000002.1 | 100 | 801 | 0 | 0 | 271334 | 272134 |
| DASELB010000005.1 | 100 | 801 | 0 | 0 | 30260 | 29460 |
| DASELE010000007.1 | 100 | 801 | 0 | 0 | 30491 | 29691 |
| DASEKY010000004.1 | 100 | 801 | 0 | 0 | 124496 | 125296 |
| DASEKZ010000002.1 | 100 | 801 | 0 | 0 | 271346 | 272146 |
| DASEKX010000002.1 | 100 | 801 | 0 | 0 | 260066 | 260866 |
| DASEKV010000002.1 | 100 | 801 | 0 | 0 | 35291 | 34491 |
| DASEKW010000001.1 | 100 | 801 | 0 | 0 | 30435 | 29635 |
| DASEKU010000005.1 | 100 | 801 | 0 | 0 | 83581 | 84381 |
| DASEKS010000001.1 | 100 | 801 | 0 | 0 | 359656 | 360456 |
| DASEKT010000001.1 | 100 | 801 | 0 | 0 | 271345 | 272145 |
| DASEKQ010000001.1 | 100 | 801 | 0 | 0 | 276894 | 277694 |
| DASEKR010000002.1 | 100 | 801 | 0 | 0 | 271345 | 272145 |
| DASEKM010000005.1 | 100 | 801 | 0 | 0 | 29955 | 29155 |
| DASEKN010000009.1 | 100 | 801 | 0 | 0 | 21991 | 22791 |
| DASEKO010000007.1 | 100 | 801 | 0 | 0 | 71086 | 71886 |
| DASEKJ010000018.1 | 100 | 801 | 0 | 0 | 30503 | 29703 |
| DASEKK010000007.1 | 100 | 801 | 0 | 0 | 29956 | 29156 |
| DASEKL010000006.1 | 100 | 801 | 0 | 0 | 29956 | 29156 |
| DASEKH010000005.1 | 100 | 801 | 0 | 0 | 29956 | 29156 |
| DASEKI010000006.1 | 100 | 801 | 0 | 0 | 29956 | 29156 |
| DASEKE010000006.1 | 100 | 801 | 0 | 0 | 29956 | 29156 |
| DASEKG010000005.1 | 100 | 801 | 0 | 0 | 35291 | 34491 |
| DASEKF010000006.1 | 100 | 801 | 0 | 0 | 29956 | 29156 |
| DASEKD010000014.1 | 100 | 801 | 0 | 0 | 14255 | 15055 |
| DASEKB010000001.1 | 100 | 801 | 0 | 0 | 260082 | 260882 |
| DASEKC010000006.1 | 100 | 801 | 0 | 0 | 29956 | 29156 |
| DASEJZ010000004.1 | 100 | 801 | 0 | 0 | 114837 | 115637 |
| DASEJX010000002.1 | 100 | 801 | 0 | 0 | 260089 | 260889 |
| DASEJY010000002.1 | 100 | 801 | 0 | 0 | 260082 | 260882 |
| DASEJW010000005.1 | 100 | 801 | 0 | 0 | 114739 | 115539 |
| DASEJU010000001.1 | 100 | 801 | 0 | 0 | 324325 | 325125 |
| DASEJR010000015.1 | 100 | 801 | 0 | 0 | 29956 | 29156 |
| DASEJQ010000010.1 | 100 | 801 | 0 | 0 | 29956 | 29156 |
| DASEJP010000012.1 | 100 | 801 | 0 | 0 | 29971 | 29171 |
| DASEJN010000020.1 | 100 | 801 | 0 | 0 | 29956 | 29156 |
| DASEJC010000014.1 | 100 | 801 | 0 | 0 | 29956 | 29156 |
| DASEIW010000015.1 | 100 | 801 | 0 | 0 | 29956 | 29156 |
| DASEIU010000045.1 | 100 | 801 | 0 | 0 | 1307 | 507 |
| DASEIV010000022.1 | 100 | 801 | 0 | 0 | 29956 | 29156 |
| DASEIT010000015.1 | 100 | 801 | 0 | 0 | 29956 | 29156 |
| DASEIS010000016.1 | 100 | 801 | 0 | 0 | 29956 | 29156 |
| DASEIP010000012.1 | 100 | 801 | 0 | 0 | 29956 | 29156 |
| DASEII010000046.1 | 100 | 801 | 0 | 0 | 10716 | 9916 |
| DASEIF010000014.1 | 100 | 801 | 0 | 0 | 4663 | 5463 |
| DASEIE010000017.1 | 100 | 801 | 0 | 0 | 28620 | 27820 |
| DASEIA010000019.1 | 100 | 801 | 0 | 0 | 20079 | 19279 |
| DASEHX010000017.1 | 100 | 801 | 0 | 0 | 30403 | 29603 |
| DASEGU010000001.1 | 100 | 801 | 0 | 0 | 29956 | 29156 |
| DASEFJ010000002.1 | 100 | 801 | 0 | 0 | 271361 | 272161 |
| DASEEK010000009.1 | 100 | 801 | 0 | 0 | 52538 | 53338 |
| DASEDZ010000001.1 | 100 | 801 | 0 | 0 | 335972 | 336772 |
| DASEEA010000001.1 | 100 | 801 | 0 | 0 | 335972 | 336772 |
| DASEDW010000001.1 | 100 | 801 | 0 | 0 | 406640 | 407440 |
| DASEDU010000001.1 | 100 | 801 | 0 | 0 | 406640 | 407440 |
| DASEDC010000001.1 | 100 | 801 | 0 | 0 | 347264 | 348064 |
| DASECJ010000001.1 | 100 | 801 | 0 | 0 | 343298 | 344098 |
| DASECH010000004.1 | 100 | 801 | 0 | 0 | 34639 | 33839 |
| DASECD010000003.1 | 100 | 801 | 0 | 0 | 34639 | 33839 |
| DASECB010000004.1 | 100 | 801 | 0 | 0 | 34639 | 33839 |
| DASEBU010000003.1 | 100 | 801 | 0 | 0 | 34646 | 33846 |
| DASCLT010000001.1 | 100 | 801 | 0 | 0 | 276929 | 277729 |
| DARZUC010000002.1 | 100 | 801 | 0 | 0 | 271186 | 271986 |
| DARZTB010000002.1 | 100 | 801 | 0 | 0 | 271476 | 272276 |
| DARZTA010000001.1 | 100 | 801 | 0 | 0 | 277044 | 277844 |
| DARZSQ010000001.1 | 100 | 801 | 0 | 0 | 343368 | 344168 |
| DARZSE010000001.1 | 100 | 801 | 0 | 0 | 35291 | 34491 |
| DARZSC010000001.1 | 100 | 801 | 0 | 0 | 343333 | 344133 |
| DARZSD010000001.1 | 100 | 801 | 0 | 0 | 343338 | 344138 |
| DARZSB010000001.1 | 100 | 801 | 0 | 0 | 343338 | 344138 |
| DARZQT010000001.1 | 100 | 801 | 0 | 0 | 347358 | 348158 |
| DARZPV010000001.1 | 100 | 801 | 0 | 0 | 343033 | 343833 |
| DARZPG010000002.1 | 100 | 801 | 0 | 0 | 271257 | 272057 |
| DARZNY010000001.1 | 100 | 801 | 0 | 0 | 20918 | 20118 |
| DARZNR010000003.1 | 100 | 801 | 0 | 0 | 29956 | 29156 |
| DARZMI010000001.1 | 100 | 801 | 0 | 0 | 29956 | 29156 |
| DARZLZ010000007.1 | 100 | 801 | 0 | 0 | 82213 | 83013 |
| DARZLT010000002.1 | 100 | 801 | 0 | 0 | 270762 | 271562 |
| DARZLQ010000002.1 | 100 | 801 | 0 | 0 | 270658 | 271458 |
| DARZKS010000001.1 | 100 | 801 | 0 | 0 | 276933 | 277733 |
| DARZKO010000001.1 | 100 | 801 | 0 | 0 | 346005 | 346805 |
| DARZKJ010000001.1 | 100 | 801 | 0 | 0 | 29956 | 29156 |
| DARZJY010000002.1 | 100 | 801 | 0 | 0 | 271056 | 271856 |
| DARZJP010000001.1 | 100 | 801 | 0 | 0 | 29956 | 29156 |
| DARZJN010000002.1 | 100 | 801 | 0 | 0 | 271146 | 271946 |
| DARZJH010000002.1 | 100 | 801 | 0 | 0 | 271357 | 272157 |
| DARZJD010000001.1 | 100 | 801 | 0 | 0 | 29956 | 29156 |
| DARZJG010000002.1 | 100 | 801 | 0 | 0 | 271330 | 272130 |
| DARZJF010000002.1 | 100 | 801 | 0 | 0 | 260023 | 260823 |
| DARZIX010000002.1 | 100 | 801 | 0 | 0 | 241081 | 241881 |
| DARZIG010000001.1 | 100 | 801 | 0 | 0 | 341459 | 342259 |
| DARZHZ010000001.1 | 100 | 801 | 0 | 0 | 331930 | 332730 |
| DARZHW010000001.1 | 100 | 801 | 0 | 0 | 342988 | 343788 |
| DARZHQ010000002.1 | 100 | 801 | 0 | 0 | 271182 | 271982 |
| DARZHO010000015.1 | 100 | 801 | 0 | 0 | 143 | 943 |
| DARZHM010000002.1 | 100 | 801 | 0 | 0 | 271348 | 272148 |
| DARZHL010000001.1 | 100 | 801 | 0 | 0 | 343293 | 344093 |
| DARZHJ010000001.1 | 100 | 801 | 0 | 0 | 60166 | 59366 |
| DARZGB010000001.1 | 100 | 801 | 0 | 0 | 29956 | 29156 |
| DARZGC010000002.1 | 100 | 801 | 0 | 0 | 260060 | 260860 |
| DARZFV010000001.1 | 100 | 801 | 0 | 0 | 276900 | 277700 |
| DARZFJ010000002.1 | 100 | 801 | 0 | 0 | 265626 | 266426 |
| DARZEH010000001.1 | 100 | 801 | 0 | 0 | 276919 | 277719 |
| DARZEI010000002.1 | 100 | 801 | 0 | 0 | 271351 | 272151 |
| DARZEK010000001.1 | 100 | 801 | 0 | 0 | 276929 | 277729 |
| DARZEB010000002.1 | 100 | 801 | 0 | 0 | 34534 | 33734 |
| DARZDZ010000002.1 | 100 | 801 | 0 | 0 | 271336 | 272136 |
| DARZDX010000001.1 | 100 | 801 | 0 | 0 | 276904 | 277704 |
| DARZED010000001.1 | 100 | 801 | 0 | 0 | 343233 | 344033 |
| DARZDY010000002.1 | 100 | 801 | 0 | 0 | 271336 | 272136 |
| DARZDW010000001.1 | 100 | 801 | 0 | 0 | 276929 | 277729 |
| DARZDV010000001.1 | 100 | 801 | 0 | 0 | 276926 | 277726 |
| DARZDP010000002.1 | 100 | 801 | 0 | 0 | 271176 | 271976 |
| DARZCC010000001.1 | 100 | 801 | 0 | 0 | 29956 | 29156 |
| DARZCE010000023.1 | 100 | 801 | 0 | 0 | 20870 | 20070 |
| DARZBI010000001.1 | 100 | 801 | 0 | 0 | 330736 | 331536 |
| DARZBF010000002.1 | 100 | 801 | 0 | 0 | 271175 | 271975 |
| DARZBA010000001.1 | 100 | 801 | 0 | 0 | 29956 | 29156 |
| DARZAV010000004.1 | 100 | 801 | 0 | 0 | 34639 | 33839 |
| DARZAS010000024.1 | 100 | 801 | 0 | 0 | 20870 | 20070 |
| DARZAQ010000002.1 | 100 | 801 | 0 | 0 | 271361 | 272161 |
| DARZAK010000001.1 | 100 | 801 | 0 | 0 | 29956 | 29156 |
| DARYYV010000002.1 | 100 | 801 | 0 | 0 | 263025 | 263825 |
| DARYYW010000003.1 | 100 | 801 | 0 | 0 | 29956 | 29156 |
| CP139163.1 | 100 | 801 | 0 | 0 | 252140 | 252940 |
| JAWWZM010000001.1 | 100 | 801 | 0 | 0 | 259947 | 260747 |
| JAWWZJ010000001.1 | 100 | 801 | 0 | 0 | 332781 | 333581 |
| JAWWZF010000004.1 | 100 | 801 | 0 | 0 | 169764 | 170564 |
| JAUTGQ010000003.1 | 100 | 801 | 0 | 0 | 30068 | 29268 |
| JAUTHY010000018.1 | 100 | 801 | 0 | 0 | 30067 | 29267 |
| JAUTHX010000001.1 | 100 | 801 | 0 | 0 | 337223 | 338023 |
| JAUTHL010000008.1 | 100 | 801 | 0 | 0 | 30044 | 29244 |
| JAUTHJ010000016.1 | 100 | 801 | 0 | 0 | 10386 | 11186 |
| JAUTHF010000022.1 | 100 | 801 | 0 | 0 | 8005 | 7205 |
| JAUTHE010000018.1 | 100 | 801 | 0 | 0 | 30118 | 29318 |
| JAUTHD010000008.1 | 100 | 801 | 0 | 0 | 46789 | 47589 |
| JAUTGV010000002.1 | 100 | 801 | 0 | 0 | 259866 | 260666 |
| JAUTGS010000005.1 | 100 | 801 | 0 | 0 | 45805 | 45005 |
| JAUTGT010000005.1 | 100 | 801 | 0 | 0 | 127008 | 127808 |
| JAUTGP010000001.1 | 100 | 801 | 0 | 0 | 30068 | 29268 |
| JAUTGN010000002.1 | 100 | 801 | 0 | 0 | 30068 | 29268 |
| JAUTFJ010000002.1 | 100 | 801 | 0 | 0 | 30068 | 29268 |
| JAUTFH010000004.1 | 100 | 801 | 0 | 0 | 66207 | 67007 |
| JAUTFF010000001.1 | 100 | 801 | 0 | 0 | 259951 | 260751 |
| JAUTFC010000001.1 | 100 | 801 | 0 | 0 | 45735 | 44935 |
| JAUTFE010000002.1 | 100 | 801 | 0 | 0 | 35613 | 34813 |
| JAUTFD010000002.1 | 100 | 801 | 0 | 0 | 260019 | 260819 |
| JAUTFB010000002.1 | 100 | 801 | 0 | 0 | 35613 | 34813 |
| JAUTEV010000002.1 | 100 | 801 | 0 | 0 | 35613 | 34813 |
| JAUTEZ010000002.1 | 100 | 801 | 0 | 0 | 35613 | 34813 |
| JAUTEX010000002.1 | 100 | 801 | 0 | 0 | 259569 | 260369 |
| JAUTFA010000010.1 | 100 | 801 | 0 | 0 | 30068 | 29268 |
| JAUTEY010000002.1 | 100 | 801 | 0 | 0 | 259569 | 260369 |
| JAUTEU010000002.1 | 100 | 801 | 0 | 0 | 30068 | 29268 |
| JAUTES010000002.1 | 100 | 801 | 0 | 0 | 259741 | 260541 |
| JAUTER010000006.1 | 100 | 801 | 0 | 0 | 64010 | 64810 |
| JAWQLT010000001.1 | 100 | 801 | 0 | 0 | 30288 | 29488 |
| CP134477.1 | 100 | 801 | 0 | 0 | 1008134 | 1007334 |
| JAVMBL010000006.1 | 100 | 801 | 0 | 0 | 95913 | 96713 |
| JAVMBI010000002.1 | 100 | 801 | 0 | 0 | 30084 | 29284 |
| JAVMBJ010000042.1 | 100 | 801 | 0 | 0 | 2438 | 3238 |
| JAVMBM010000003.1 | 100 | 801 | 0 | 0 | 35419 | 34619 |
| JAVMBN010000006.1 | 100 | 801 | 0 | 0 | 91464 | 92264 |
| JARATX010000009.1 | 100 | 801 | 0 | 0 | 30456 | 29656 |
| JARATW010000002.1 | 100 | 801 | 0 | 0 | 30460 | 29660 |
| JARATQ010000001.1 | 100 | 801 | 0 | 0 | 321290 | 322090 |
| JARATU010000002.1 | 100 | 801 | 0 | 0 | 271303 | 272103 |
| JARATT010000001.1 | 100 | 801 | 0 | 0 | 30456 | 29656 |
| JARATP010000002.1 | 100 | 801 | 0 | 0 | 271143 | 271943 |
| JARATO010000002.1 | 100 | 801 | 0 | 0 | 271420 | 272220 |
| JARATL010000006.1 | 100 | 801 | 0 | 0 | 90748 | 91548 |
| JARATM010000002.1 | 100 | 801 | 0 | 0 | 271532 | 272332 |
| JARATK010000002.1 | 100 | 801 | 0 | 0 | 30456 | 29656 |
| JARATJ010000006.1 | 100 | 801 | 0 | 0 | 67399 | 68199 |
| JARATH010000003.1 | 100 | 801 | 0 | 0 | 182121 | 182921 |
| JARATF010000010.1 | 100 | 801 | 0 | 0 | 51310 | 52110 |
| JARATG010000017.1 | 100 | 801 | 0 | 0 | 19050 | 19850 |
| JARATI010000005.1 | 100 | 801 | 0 | 0 | 30091 | 29291 |
| JARATC010000004.1 | 100 | 801 | 0 | 0 | 30084 | 29284 |
| JARATB010000004.1 | 100 | 801 | 0 | 0 | 67399 | 68199 |
| JARASZ010000002.1 | 100 | 801 | 0 | 0 | 182081 | 182881 |
| JARASY010000011.1 | 100 | 801 | 0 | 0 | 51310 | 52110 |
| JARASX010000002.1 | 100 | 801 | 0 | 0 | 258862 | 259662 |
| JARASR010000002.1 | 100 | 801 | 0 | 0 | 30456 | 29656 |
| JARASV010000003.1 | 100 | 801 | 0 | 0 | 30305 | 29505 |
| JARASU010000002.1 | 100 | 801 | 0 | 0 | 271201 | 272001 |
| JARAST010000011.1 | 100 | 801 | 0 | 0 | 30456 | 29656 |
| JARASP010000001.1 | 100 | 801 | 0 | 0 | 328854 | 329654 |
| JARASQ010000002.1 | 100 | 801 | 0 | 0 | 30019 | 29219 |
| JARASO010000001.1 | 100 | 801 | 0 | 0 | 339892 | 340692 |
| JARASN010000001.1 | 100 | 801 | 0 | 0 | 30019 | 29219 |
| JARASL010000026.1 | 100 | 801 | 0 | 0 | 4597 | 5397 |
| JARASM010000002.1 | 100 | 801 | 0 | 0 | 30019 | 29219 |
| JARASK010000002.1 | 100 | 801 | 0 | 0 | 30091 | 29291 |
| JAKTCQ010000002.1 | 100 | 801 | 0 | 0 | 259867 | 260667 |
| JAKTCR010000002.1 | 100 | 801 | 0 | 0 | 271241 | 272041 |
| JAKTCS010000006.1 | 100 | 801 | 0 | 0 | 96336 | 97136 |
| JAKTCT010000002.1 | 100 | 801 | 0 | 0 | 260239 | 261039 |
| JAKTCU010000002.1 | 100 | 801 | 0 | 0 | 258151 | 258951 |
| JAKTCV010000001.1 | 100 | 801 | 0 | 0 | 30034 | 29234 |
| JAKTCW010000001.1 | 100 | 801 | 0 | 0 | 384045 | 384845 |
| JAKTCX010000002.1 | 100 | 801 | 0 | 0 | 259976 | 260776 |
| JAKTCY010000001.1 | 100 | 801 | 0 | 0 | 338422 | 339222 |
| JAKTCZ010000001.1 | 100 | 801 | 0 | 0 | 112993 | 112193 |
| JAKTDA010000001.1 | 100 | 801 | 0 | 0 | 35370 | 34570 |
| JAKTDB010000001.1 | 100 | 801 | 0 | 0 | 338272 | 339072 |
| JAKTDC010000006.1 | 100 | 801 | 0 | 0 | 84012 | 84812 |
| JAKTDE010000002.1 | 100 | 801 | 0 | 0 | 259959 | 260759 |
| JAKTDF010000006.1 | 100 | 801 | 0 | 0 | 94602 | 95402 |
| JAKTDG010000002.1 | 100 | 801 | 0 | 0 | 259959 | 260759 |
| JAKTDH010000002.1 | 100 | 801 | 0 | 0 | 271238 | 272038 |
| JAKTDI010000006.1 | 100 | 801 | 0 | 0 | 83634 | 84434 |
| JAKTDK010000001.1 | 100 | 801 | 0 | 0 | 354049 | 354849 |
| JAKTDJ010000002.1 | 100 | 801 | 0 | 0 | 30278 | 29478 |
| JAKTDL010000006.1 | 100 | 801 | 0 | 0 | 94662 | 95462 |
| JAKTDM010000001.1 | 100 | 801 | 0 | 0 | 30041 | 29241 |
| JAKTDO010000001.1 | 100 | 801 | 0 | 0 | 30246 | 29446 |
| JAKTDN010000001.1 | 100 | 801 | 0 | 0 | 30034 | 29234 |
| JAKTDQ010000001.1 | 100 | 801 | 0 | 0 | 30253 | 29453 |
| JAKTDP010000001.1 | 100 | 801 | 0 | 0 | 30034 | 29234 |
| JAKTDR010000001.1 | 100 | 801 | 0 | 0 | 338312 | 339112 |
| JAKTDS010000002.1 | 100 | 801 | 0 | 0 | 344065 | 344865 |
| JAKTDU010000001.1 | 100 | 801 | 0 | 0 | 349586 | 350386 |
| JAKTDT010000001.1 | 100 | 801 | 0 | 0 | 338269 | 339069 |
| JAKTDV010000001.1 | 100 | 801 | 0 | 0 | 349588 | 350388 |
| JAKTDW010000001.1 | 100 | 801 | 0 | 0 | 338422 | 339222 |
| JAKTDX010000002.1 | 100 | 801 | 0 | 0 | 271238 | 272038 |
| JAKTDY010000002.1 | 100 | 801 | 0 | 0 | 30041 | 29241 |
| JAKTEA010000001.1 | 100 | 801 | 0 | 0 | 30034 | 29234 |
| JAKTDZ010000002.1 | 100 | 801 | 0 | 0 | 30041 | 29241 |
| JAKTEC010000001.1 | 100 | 801 | 0 | 0 | 30034 | 29234 |
| JAKTEB010000002.1 | 100 | 801 | 0 | 0 | 271238 | 272038 |
| JAKTED010000006.1 | 100 | 801 | 0 | 0 | 82852 | 83652 |
| JAKTEE010000001.1 | 100 | 801 | 0 | 0 | 30034 | 29234 |
| JAKTEF010000002.1 | 100 | 801 | 0 | 0 | 30041 | 29241 |
| JAKTEG010000002.1 | 100 | 801 | 0 | 0 | 30041 | 29241 |
| JAKTEI010000001.1 | 100 | 801 | 0 | 0 | 30041 | 29241 |
| JAKTEH010000001.1 | 100 | 801 | 0 | 0 | 308745 | 309545 |
| JAKTEJ010000002.1 | 100 | 801 | 0 | 0 | 266366 | 267166 |
| JAKTEK010000002.1 | 100 | 801 | 0 | 0 | 30041 | 29241 |
| JAKTEL010000002.1 | 100 | 801 | 0 | 0 | 30034 | 29234 |
| JAKTEM010000002.1 | 100 | 801 | 0 | 0 | 259869 | 260669 |
| JAKTEN010000002.1 | 100 | 801 | 0 | 0 | 30246 | 29446 |
| JAKTEO010000002.1 | 100 | 801 | 0 | 0 | 259960 | 260760 |
| JAKTEP010000002.1 | 100 | 801 | 0 | 0 | 259960 | 260760 |
| JAKTEQ010000002.1 | 100 | 801 | 0 | 0 | 259960 | 260760 |
| JAKTER010000001.1 | 100 | 801 | 0 | 0 | 338290 | 339090 |
| JAKTET010000006.1 | 100 | 801 | 0 | 0 | 30041 | 29241 |
| JAKTEU010000001.1 | 100 | 801 | 0 | 0 | 30034 | 29234 |
| JAKTEV010000001.1 | 100 | 801 | 0 | 0 | 276959 | 277759 |
| JAKTEW010000001.1 | 100 | 801 | 0 | 0 | 276959 | 277759 |
| JAKTEY010000002.1 | 100 | 801 | 0 | 0 | 30041 | 29241 |
| JAKTEZ010000001.1 | 100 | 801 | 0 | 0 | 343589 | 344389 |
| JAKTFA010000002.1 | 100 | 801 | 0 | 0 | 258151 | 258951 |
| JAKTFB010000020.1 | 100 | 801 | 0 | 0 | 30034 | 29234 |
| JAKTFC010000007.1 | 100 | 801 | 0 | 0 | 68604 | 69404 |
| JAKTFD010000002.1 | 100 | 801 | 0 | 0 | 30041 | 29241 |
| JAKTFE010000002.1 | 100 | 801 | 0 | 0 | 271238 | 272038 |
| JAKTFG010000006.1 | 100 | 801 | 0 | 0 | 30041 | 29241 |
| JAKTFF010000001.1 | 100 | 801 | 0 | 0 | 348369 | 349169 |
| JAKTFI010000006.1 | 100 | 801 | 0 | 0 | 30041 | 29241 |
| JAKTFH010000002.1 | 100 | 801 | 0 | 0 | 259959 | 260759 |
| JAKTFJ010000007.1 | 100 | 801 | 0 | 0 | 106335 | 107135 |
| JAKTFK010000003.1 | 100 | 801 | 0 | 0 | 193397 | 194197 |
| JAKTFL010000002.1 | 100 | 801 | 0 | 0 | 35369 | 34569 |
| JAKTFM010000002.1 | 100 | 801 | 0 | 0 | 30041 | 29241 |
| JAKTFN010000001.1 | 100 | 801 | 0 | 0 | 35370 | 34570 |
| JAKTFO010000002.1 | 100 | 801 | 0 | 0 | 271236 | 272036 |
| JAKTFQ010000006.1 | 100 | 801 | 0 | 0 | 94662 | 95462 |
| JAKTFP010000001.1 | 100 | 801 | 0 | 0 | 259959 | 260759 |
| JAKTFR010000001.1 | 100 | 801 | 0 | 0 | 30034 | 29234 |
| JAKTFS010000001.1 | 100 | 801 | 0 | 0 | 35369 | 34569 |
| JAKTFU010000002.1 | 100 | 801 | 0 | 0 | 259938 | 260738 |
| JAKTFT010000002.1 | 100 | 801 | 0 | 0 | 265728 | 266528 |
| JAKTFV010000002.1 | 100 | 801 | 0 | 0 | 30041 | 29241 |
| JAKTFW010000002.1 | 100 | 801 | 0 | 0 | 271228 | 272028 |
| JAKTFX010000002.1 | 100 | 801 | 0 | 0 | 271228 | 272028 |
| JAKTFY010000001.1 | 100 | 801 | 0 | 0 | 36061 | 35261 |
| JAKTFZ010000001.1 | 100 | 801 | 0 | 0 | 30034 | 29234 |
| JAKTGA010000002.1 | 100 | 801 | 0 | 0 | 35369 | 34569 |
| JAKTGB010000001.1 | 100 | 801 | 0 | 0 | 330985 | 331785 |
| JAKTGD010000002.1 | 100 | 801 | 0 | 0 | 271238 | 272038 |
| JAKTGE010000001.1 | 100 | 801 | 0 | 0 | 259960 | 260760 |
| JAKTGF010000002.1 | 100 | 801 | 0 | 0 | 30062 | 29262 |
| JAKTGG010000001.1 | 100 | 801 | 0 | 0 | 270820 | 271620 |
| JAKTGH010000002.1 | 100 | 801 | 0 | 0 | 30041 | 29241 |
| JAKTGI010000002.1 | 100 | 801 | 0 | 0 | 30041 | 29241 |
| JAKTGJ010000001.1 | 100 | 801 | 0 | 0 | 355332 | 356132 |
| JAKTGK010000001.1 | 100 | 801 | 0 | 0 | 349579 | 350379 |
| JAKTGL010000006.1 | 100 | 801 | 0 | 0 | 30050 | 29250 |
| JAKTGN010000002.1 | 100 | 801 | 0 | 0 | 265712 | 266512 |
| JAKTGM010000002.1 | 100 | 801 | 0 | 0 | 30041 | 29241 |
| JAKTGO010000006.1 | 100 | 801 | 0 | 0 | 94643 | 95443 |
| JAKTGP010000006.1 | 100 | 801 | 0 | 0 | 94643 | 95443 |
| JAKTGQ010000006.1 | 100 | 801 | 0 | 0 | 94583 | 95383 |
| JAKTGR010000002.1 | 100 | 801 | 0 | 0 | 259958 | 260758 |
| JAKTGS010000001.1 | 100 | 801 | 0 | 0 | 30246 | 29446 |
| JAKTGT010000001.1 | 100 | 801 | 0 | 0 | 337340 | 338140 |
| JAKTGU010000006.1 | 100 | 801 | 0 | 0 | 30041 | 29241 |
| JAKTGV010000001.1 | 100 | 801 | 0 | 0 | 30034 | 29234 |
| JAKTGX010000001.1 | 100 | 801 | 0 | 0 | 327475 | 328275 |
| JAKTGW010000001.1 | 100 | 801 | 0 | 0 | 333340 | 334140 |
| JAKTGZ010000003.1 | 100 | 801 | 0 | 0 | 155989 | 156789 |
| JAKTGY010000001.1 | 100 | 801 | 0 | 0 | 30246 | 29446 |
| JAKTHA010000002.1 | 100 | 801 | 0 | 0 | 30041 | 29241 |
| JAKTHB010000001.1 | 100 | 801 | 0 | 0 | 354661 | 355461 |
| JAKTHC010000006.1 | 100 | 801 | 0 | 0 | 30041 | 29241 |
| JAKTHD010000002.1 | 100 | 801 | 0 | 0 | 271454 | 272254 |
| JAKTHE010000001.1 | 100 | 801 | 0 | 0 | 335734 | 336534 |
| JAKTHF010000002.1 | 100 | 801 | 0 | 0 | 30034 | 29234 |
| JAKTHG010000002.1 | 100 | 801 | 0 | 0 | 270687 | 271487 |
| JAKTHH010000001.1 | 100 | 801 | 0 | 0 | 30034 | 29234 |
| JAKTHI010000001.1 | 100 | 801 | 0 | 0 | 30034 | 29234 |
| JAKTHJ010000001.1 | 100 | 801 | 0 | 0 | 112671 | 111871 |
| JAKTHK010000001.1 | 100 | 801 | 0 | 0 | 30124 | 29324 |
| JAKTHL010000002.1 | 100 | 801 | 0 | 0 | 271238 | 272038 |
| JAKTHN010000002.1 | 100 | 801 | 0 | 0 | 30278 | 29478 |
| JAKTHO010000002.1 | 100 | 801 | 0 | 0 | 259974 | 260774 |
| JAKTHP010000002.1 | 100 | 801 | 0 | 0 | 30041 | 29241 |
| JAKTHQ010000002.1 | 100 | 801 | 0 | 0 | 259963 | 260763 |
| JAKTHR010000001.1 | 100 | 801 | 0 | 0 | 35370 | 34570 |
| JAKTHT010000001.1 | 100 | 801 | 0 | 0 | 30034 | 29234 |
| JAKTHS010000001.1 | 100 | 801 | 0 | 0 | 259959 | 260759 |
| JAKTHU010000006.1 | 100 | 801 | 0 | 0 | 30033 | 29233 |
| CP102748.1 | 100 | 801 | 0 | 0 | 1020672 | 1021472 |
| CP102746.1 | 100 | 801 | 0 | 0 | 937088 | 937888 |
| JARBFN010000001.1 | 100 | 801 | 0 | 0 | 30275 | 29475 |
| JARBFQ010000002.1 | 100 | 801 | 0 | 0 | 30084 | 29284 |
| JABMDA010000002.1 | 100 | 801 | 0 | 0 | 30084 | 29284 |
| CP109942.1 | 100 | 801 | 0 | 0 | 1030300 | 1031100 |
| CP109941.1 | 100 | 801 | 0 | 0 | 942784 | 943584 |
| CP102154.1 | 100 | 801 | 0 | 0 | 936445 | 937245 |
| JAMWEI010000001.1 | 100 | 801 | 0 | 0 | 347305 | 348105 |
| JAMWEN010000002.1 | 100 | 801 | 0 | 0 | 30084 | 29284 |
| JAMDJI010000001.1 | 100 | 801 | 0 | 0 | 30041 | 29241 |
| JAMDJH010000001.1 | 100 | 801 | 0 | 0 | 30034 | 29234 |
| JAMDJE010000002.1 | 100 | 801 | 0 | 0 | 271218 | 272018 |
| JAMDIZ010000002.1 | 100 | 801 | 0 | 0 | 30034 | 29234 |
| JAMDIY010000001.1 | 100 | 801 | 0 | 0 | 271207 | 272007 |
| JAMDIU010000005.1 | 100 | 801 | 0 | 0 | 77867 | 78667 |
| JAMDIO010000001.1 | 100 | 801 | 0 | 0 | 259939 | 260739 |
| JAMDIE010000001.1 | 100 | 801 | 0 | 0 | 258115 | 258915 |
| JAMDHW010000002.1 | 100 | 801 | 0 | 0 | 258115 | 258915 |
| JAASDZ010000007.1 | 100 | 801 | 0 | 0 | 343583 | 344383 |
| JALLAM010000002.1 | 100 | 801 | 0 | 0 | 47444 | 46644 |
| JALLAO010000004.1 | 100 | 801 | 0 | 0 | 30044 | 29244 |
| CP095463.1 | 100 | 801 | 0 | 0 | 1122866 | 1122066 |
| JALIDS010000002.1 | 100 | 801 | 0 | 0 | 29978 | 29178 |
| JALIDV010000002.1 | 100 | 801 | 0 | 0 | 270969 | 271769 |
| JALIDU010000002.1 | 100 | 801 | 0 | 0 | 29978 | 29178 |
| JALIDT010000002.1 | 100 | 801 | 0 | 0 | 260178 | 260978 |
| JALIDR010000002.1 | 100 | 801 | 0 | 0 | 29978 | 29178 |
| JAKVTI010000001.1 | 100 | 801 | 0 | 0 | 328037 | 328837 |
| JAKTCE010000001.1 | 100 | 801 | 0 | 0 | 271225 | 272025 |
| JAKTBU010000002.1 | 100 | 801 | 0 | 0 | 271218 | 272018 |
| JAKTBX010000001.1 | 100 | 801 | 0 | 0 | 341497 | 342297 |
| JAKTAK010000001.1 | 100 | 801 | 0 | 0 | 335755 | 336555 |
| JAKTAL010000001.1 | 100 | 801 | 0 | 0 | 112671 | 111871 |
| JAKTAN010000002.1 | 100 | 801 | 0 | 0 | 30034 | 29234 |
| JAKTAR010000002.1 | 100 | 801 | 0 | 0 | 259971 | 260771 |
| JAKTAS010000002.1 | 100 | 801 | 0 | 0 | 259971 | 260771 |
| JAKTAQ010000001.1 | 100 | 801 | 0 | 0 | 340984 | 341784 |
| JAKTAM010000001.1 | 100 | 801 | 0 | 0 | 30034 | 29234 |
| JAKTAT010000002.1 | 100 | 801 | 0 | 0 | 259971 | 260771 |
| JAKTBP010000002.1 | 100 | 801 | 0 | 0 | 258115 | 258915 |
| JAKTBQ010000002.1 | 100 | 801 | 0 | 0 | 258115 | 258915 |
| JAKTBY010000001.1 | 100 | 801 | 0 | 0 | 335744 | 336544 |
| JAKTBS010000001.1 | 100 | 801 | 0 | 0 | 30041 | 29241 |
| JAKTAH010000002.1 | 100 | 801 | 0 | 0 | 30041 | 29241 |
| JAKTBZ010000001.1 | 100 | 801 | 0 | 0 | 335744 | 336544 |
| JAKTCA010000001.1 | 100 | 801 | 0 | 0 | 341497 | 342297 |
| CP091422.1 | 100 | 801 | 0 | 0 | 862859 | 862059 |
| CP091423.1 | 100 | 801 | 0 | 0 | 1022553 | 1021753 |
| CP085088.1 | 100 | 801 | 0 | 0 | 907903 | 908703 |
| JAJBSM010000001.1 | 100 | 801 | 0 | 0 | 936430 | 937230 |
| CP082948.1 | 100 | 801 | 0 | 0 | 1051738 | 1052538 |
| CP082201.1 | 100 | 801 | 0 | 0 | 993035 | 993835 |
| CP082200.1 | 100 | 801 | 0 | 0 | 992483 | 993283 |
| CP082197.1 | 100 | 801 | 0 | 0 | 819185 | 818385 |
| CP082199.1 | 100 | 801 | 0 | 0 | 990603 | 991403 |
| CP082198.1 | 100 | 801 | 0 | 0 | 995043 | 995843 |
| JAIMDX010000001.1 | 100 | 801 | 0 | 0 | 328399 | 329199 |
| JAIMDR010000001.1 | 100 | 801 | 0 | 0 | 271263 | 272063 |
| CP078543.1 | 100 | 801 | 0 | 0 | 1100565 | 1101365 |
| PXOO01000003.1 | 100 | 801 | 0 | 0 | 96148 | 96948 |
| PXON01000002.1 | 100 | 801 | 0 | 0 | 331414 | 332214 |
| PXOP01000001.1 | 100 | 801 | 0 | 0 | 356271 | 357071 |
| PXOM01000002.1 | 100 | 801 | 0 | 0 | 331409 | 332209 |
| PXOL01000002.1 | 100 | 801 | 0 | 0 | 35440 | 34640 |
| PXOI01000002.1 | 100 | 801 | 0 | 0 | 30567 | 29767 |
| PXOJ01000002.1 | 100 | 801 | 0 | 0 | 126529 | 127329 |
| PXOK01000002.1 | 100 | 801 | 0 | 0 | 22301 | 21501 |
| VZMV01000002.1 | 100 | 801 | 0 | 0 | 30065 | 29265 |
| VZMW01000002.1 | 100 | 801 | 0 | 0 | 30065 | 29265 |
| VZNA01000010.1 | 100 | 801 | 0 | 0 | 30063 | 29263 |
| VZNI01000001.1 | 100 | 801 | 0 | 0 | 271242 | 272042 |
| VZNQ01000001.1 | 100 | 801 | 0 | 0 | 30065 | 29265 |
| VZNW01000002.1 | 100 | 801 | 0 | 0 | 30065 | 29265 |
| VZJB01000001.1 | 100 | 801 | 0 | 0 | 338314 | 339114 |
| VZJC01000001.1 | 100 | 801 | 0 | 0 | 30066 | 29266 |
| VZJA01000006.1 | 100 | 801 | 0 | 0 | 30270 | 29470 |
| VZJD01000001.1 | 100 | 801 | 0 | 0 | 30058 | 29258 |
| VZJH01000001.1 | 100 | 801 | 0 | 0 | 30270 | 29470 |
| VZJE01000001.1 | 100 | 801 | 0 | 0 | 271262 | 272062 |
| VZJF01000001.1 | 100 | 801 | 0 | 0 | 112855 | 112055 |
| VZJG01000001.1 | 100 | 801 | 0 | 0 | 421558 | 422358 |
| VZJI01000001.1 | 100 | 801 | 0 | 0 | 30065 | 29265 |
| VZJK01000001.1 | 100 | 801 | 0 | 0 | 403922 | 404722 |
| VZJM01000001.1 | 100 | 801 | 0 | 0 | 455770 | 456570 |
| VZJJ01000002.1 | 100 | 801 | 0 | 0 | 30080 | 29280 |
| VZJN01000001.1 | 100 | 801 | 0 | 0 | 421763 | 422563 |
| VZJL01000001.1 | 100 | 801 | 0 | 0 | 410488 | 411288 |
| VZJO01000002.1 | 100 | 801 | 0 | 0 | 30065 | 29265 |
| VZJR01000006.1 | 100 | 801 | 0 | 0 | 30058 | 29258 |
| VZJP01000001.1 | 100 | 801 | 0 | 0 | 456023 | 456823 |
| VZJQ01000001.1 | 100 | 801 | 0 | 0 | 30065 | 29265 |
| VZJT01000001.1 | 100 | 801 | 0 | 0 | 30058 | 29258 |
| VZJS01000001.1 | 100 | 801 | 0 | 0 | 30277 | 29477 |
| VZJU01000002.1 | 100 | 801 | 0 | 0 | 30058 | 29258 |
| VZJX01000002.1 | 100 | 801 | 0 | 0 | 30070 | 29270 |
| VZJW01000002.1 | 100 | 801 | 0 | 0 | 30058 | 29258 |
| VZJY01000002.1 | 100 | 801 | 0 | 0 | 30065 | 29265 |
| VZJV01000001.1 | 100 | 801 | 0 | 0 | 397726 | 398526 |
| VZJZ01000001.1 | 100 | 801 | 0 | 0 | 343472 | 344272 |
| VZKA01000002.1 | 100 | 801 | 0 | 0 | 30065 | 29265 |
| VZKC01000001.1 | 100 | 801 | 0 | 0 | 30270 | 29470 |
| VZKB01000002.1 | 100 | 801 | 0 | 0 | 30051 | 29251 |
| VZKD01000002.1 | 100 | 801 | 0 | 0 | 30065 | 29265 |
| VZKF01000001.1 | 100 | 801 | 0 | 0 | 332376 | 333176 |
| VZKH01000002.1 | 100 | 801 | 0 | 0 | 30065 | 29265 |
| VZKE01000001.1 | 100 | 801 | 0 | 0 | 421725 | 422525 |
| VZKG01000006.1 | 100 | 801 | 0 | 0 | 30058 | 29258 |
| VZKI01000006.1 | 100 | 801 | 0 | 0 | 26412 | 27212 |
| VZKL01000001.1 | 100 | 801 | 0 | 0 | 404894 | 405694 |
| VZKM01000006.1 | 100 | 801 | 0 | 0 | 30270 | 29470 |
| VZKK01000001.1 | 100 | 801 | 0 | 0 | 30058 | 29258 |
| VZKJ01000001.1 | 100 | 801 | 0 | 0 | 403808 | 404608 |
| VZKN01000005.1 | 100 | 801 | 0 | 0 | 124598 | 125398 |
| VZKO01000001.1 | 100 | 801 | 0 | 0 | 271263 | 272063 |
| VZKP01000001.1 | 100 | 801 | 0 | 0 | 259983 | 260783 |
| VZKQ01000001.1 | 100 | 801 | 0 | 0 | 410404 | 411204 |
| VZKS01000001.1 | 100 | 801 | 0 | 0 | 270943 | 271743 |
| VZKR01000001.1 | 100 | 801 | 0 | 0 | 338326 | 339126 |
| VZKU01000001.1 | 100 | 801 | 0 | 0 | 30058 | 29258 |
| VZKT01000002.1 | 100 | 801 | 0 | 0 | 29233 | 28433 |
| VZKV01000001.1 | 100 | 801 | 0 | 0 | 112695 | 111895 |
| VZKW01000002.1 | 100 | 801 | 0 | 0 | 267088 | 267888 |
| VZKY01000001.1 | 100 | 801 | 0 | 0 | 399529 | 400329 |
| VZKX01000001.1 | 100 | 801 | 0 | 0 | 30058 | 29258 |
| VZKZ01000001.1 | 100 | 801 | 0 | 0 | 30270 | 29470 |
| VZLB01000001.1 | 100 | 801 | 0 | 0 | 403865 | 404665 |
| VZLA01000001.1 | 100 | 801 | 0 | 0 | 407973 | 408773 |
| VZLD01000001.1 | 100 | 801 | 0 | 0 | 30058 | 29258 |
| VZLC01000001.1 | 100 | 801 | 0 | 0 | 432331 | 433131 |
| VZLE01000001.1 | 100 | 801 | 0 | 0 | 30058 | 29258 |
| VZLF01000001.1 | 100 | 801 | 0 | 0 | 331791 | 332591 |
| VZLH01000001.1 | 100 | 801 | 0 | 0 | 421052 | 421852 |
| VZLJ01000001.1 | 100 | 801 | 0 | 0 | 432330 | 433130 |
| VZLI01000002.1 | 100 | 801 | 0 | 0 | 30313 | 29513 |
| VZLK01000002.1 | 100 | 801 | 0 | 0 | 30065 | 29265 |
| VZLL01000001.1 | 100 | 801 | 0 | 0 | 30270 | 29470 |
| VZLM01000001.1 | 100 | 801 | 0 | 0 | 112855 | 112055 |
| VZLO01000002.1 | 100 | 801 | 0 | 0 | 271273 | 272073 |
| VZLN01000001.1 | 100 | 801 | 0 | 0 | 112855 | 112055 |
| VZLQ01000006.1 | 100 | 801 | 0 | 0 | 30065 | 29265 |
| VZLR01000002.1 | 100 | 801 | 0 | 0 | 193503 | 194303 |
| VZLP01000001.1 | 100 | 801 | 0 | 0 | 332341 | 333141 |
| VZLT01000001.1 | 100 | 801 | 0 | 0 | 406645 | 407445 |
| VZLS01000001.1 | 100 | 801 | 0 | 0 | 324580 | 325380 |
| VZLV01000001.1 | 100 | 801 | 0 | 0 | 30058 | 29258 |
| VZLU01000001.1 | 100 | 801 | 0 | 0 | 30058 | 29258 |
| VZLW01000001.1 | 100 | 801 | 0 | 0 | 410516 | 411316 |
| VZLX01000001.1 | 100 | 801 | 0 | 0 | 410506 | 411306 |
| VZLY01000001.1 | 100 | 801 | 0 | 0 | 30058 | 29258 |
| VZMA01000001.1 | 100 | 801 | 0 | 0 | 410371 | 411171 |
| VZLZ01000001.1 | 100 | 801 | 0 | 0 | 30058 | 29258 |
| VZMC01000007.1 | 100 | 801 | 0 | 0 | 24543 | 25343 |
| VZMB01000001.1 | 100 | 801 | 0 | 0 | 410516 | 411316 |
| VZMD01000001.1 | 100 | 801 | 0 | 0 | 30270 | 29470 |
| VZMF01000006.1 | 100 | 801 | 0 | 0 | 30058 | 29258 |
| VZME01000001.1 | 100 | 801 | 0 | 0 | 30058 | 29258 |
| VZMG01000001.1 | 100 | 801 | 0 | 0 | 406645 | 407445 |
| VZMH01000001.1 | 100 | 801 | 0 | 0 | 338060 | 338860 |
| VZMI01000001.1 | 100 | 801 | 0 | 0 | 30058 | 29258 |
| VZMJ01000001.1 | 100 | 801 | 0 | 0 | 271262 | 272062 |
| VZML01000001.1 | 100 | 801 | 0 | 0 | 332179 | 332979 |
| VZMM01000001.1 | 100 | 801 | 0 | 0 | 30270 | 29470 |
| VZMN01000005.1 | 100 | 801 | 0 | 0 | 35393 | 34593 |
| VZMO01000004.1 | 100 | 801 | 0 | 0 | 125098 | 125898 |
| VZMR01000001.1 | 100 | 801 | 0 | 0 | 30058 | 29258 |
| VZMT01000001.1 | 100 | 801 | 0 | 0 | 112855 | 112055 |
| VZMS01000001.1 | 100 | 801 | 0 | 0 | 112855 | 112055 |
| VZMU01000001.1 | 100 | 801 | 0 | 0 | 30058 | 29258 |
| JAGUCC010000001.1 | 100 | 801 | 0 | 0 | 29957 | 29157 |
| JAGUAB010000002.1 | 100 | 801 | 0 | 0 | 259894 | 260694 |
| JAGUAF010000001.1 | 100 | 801 | 0 | 0 | 276883 | 277683 |
| JAGUCB010000001.1 | 100 | 801 | 0 | 0 | 29957 | 29157 |
| JAGUBZ010000001.1 | 100 | 801 | 0 | 0 | 29957 | 29157 |
| JAGUCD010000002.1 | 100 | 801 | 0 | 0 | 259923 | 260723 |
| JAGUAT010000001.1 | 100 | 801 | 0 | 0 | 327829 | 328629 |
| JAGUAV010000001.1 | 100 | 801 | 0 | 0 | 112594 | 111794 |
| JAGUAR010000001.1 | 100 | 801 | 0 | 0 | 29957 | 29157 |
| JAGUAP010000002.1 | 100 | 801 | 0 | 0 | 271211 | 272011 |
| JAGUAO010000001.1 | 100 | 801 | 0 | 0 | 327419 | 328219 |
| JAGUAQ010000001.1 | 100 | 801 | 0 | 0 | 3709 | 2909 |
| JAGUCE010000001.1 | 100 | 801 | 0 | 0 | 29960 | 29160 |
| JAGUAC010000001.1 | 100 | 801 | 0 | 0 | 343185 | 343985 |
| JAGUBD010000001.1 | 100 | 801 | 0 | 0 | 29957 | 29157 |
| JAGUBC010000006.1 | 100 | 801 | 0 | 0 | 29957 | 29157 |
| JAGUBA010000001.1 | 100 | 801 | 0 | 0 | 360091 | 360891 |
| JAGUBB010000001.1 | 100 | 801 | 0 | 0 | 29957 | 29157 |
| JAGUAZ010000002.1 | 100 | 801 | 0 | 0 | 29956 | 29156 |
| JAGUAX010000001.1 | 100 | 801 | 0 | 0 | 112594 | 111794 |
| JAGUAY010000001.1 | 100 | 801 | 0 | 0 | 29957 | 29157 |
| JAGUAS010000009.1 | 100 | 801 | 0 | 0 | 29957 | 29157 |
| JAGUAW010000001.1 | 100 | 801 | 0 | 0 | 327829 | 328629 |
| JAGUAU010000005.1 | 100 | 801 | 0 | 0 | 112594 | 111794 |
| JAGUCA010000001.1 | 100 | 801 | 0 | 0 | 29957 | 29157 |
| JAGUBX010000001.1 | 100 | 801 | 0 | 0 | 29957 | 29157 |
| JAGUBY010000001.1 | 100 | 801 | 0 | 0 | 29957 | 29157 |
| JAGUBT010000001.1 | 100 | 801 | 0 | 0 | 327955 | 328755 |
| JAGUBV010000002.1 | 100 | 801 | 0 | 0 | 271196 | 271996 |
| JAGUBU010000001.1 | 100 | 801 | 0 | 0 | 29961 | 29161 |
| JAGUBS010000002.1 | 100 | 801 | 0 | 0 | 29957 | 29157 |
| JAGUBR010000001.1 | 100 | 801 | 0 | 0 | 29957 | 29157 |
| JAGUBQ010000001.1 | 100 | 801 | 0 | 0 | 112594 | 111794 |
| JAGUBM010000002.1 | 100 | 801 | 0 | 0 | 29957 | 29157 |
| JAGUBO010000001.1 | 100 | 801 | 0 | 0 | 30076 | 29276 |
| JAGUBN010000001.1 | 100 | 801 | 0 | 0 | 29957 | 29157 |
| JAGUBH010000001.1 | 100 | 801 | 0 | 0 | 29957 | 29157 |
| JAGUBG010000002.1 | 100 | 801 | 0 | 0 | 29957 | 29157 |
| JAGUBE010000001.1 | 100 | 801 | 0 | 0 | 346902 | 347702 |
| JAGJDV010000002.1 | 100 | 801 | 0 | 0 | 168027 | 168827 |
| WABV01000002.1 | 100 | 801 | 0 | 0 | 29957 | 29157 |
| WABX01000002.1 | 100 | 801 | 0 | 0 | 271239 | 272039 |
| WABY01000002.1 | 100 | 801 | 0 | 0 | 29957 | 29157 |
| WABW01000002.1 | 100 | 801 | 0 | 0 | 29957 | 29157 |
| WACB01000002.1 | 100 | 801 | 0 | 0 | 271234 | 272034 |
| WABZ01000002.1 | 100 | 801 | 0 | 0 | 271242 | 272042 |
| WACA01000002.1 | 100 | 801 | 0 | 0 | 29957 | 29157 |
| JAFEIS010000001.1 | 100 | 801 | 0 | 0 | 30084 | 29284 |
| WCJG01000001.1 | 100 | 801 | 0 | 0 | 29957 | 29157 |
| WCJF01000001.1 | 100 | 801 | 0 | 0 | 347293 | 348093 |
| WCJD01000001.1 | 100 | 801 | 0 | 0 | 324452 | 325252 |
| WCJC01000001.1 | 100 | 801 | 0 | 0 | 324302 | 325102 |
| WCJB01000001.1 | 100 | 801 | 0 | 0 | 29957 | 29157 |
| BCEL01000017.1 | 100 | 801 | 0 | 0 | 318763 | 319563 |
| BCEK01000025.1 | 100 | 801 | 0 | 0 | 30061 | 29261 |
| BCEI01000022.1 | 100 | 801 | 0 | 0 | 327780 | 328580 |
| BCDU01000012.1 | 100 | 801 | 0 | 0 | 322044 | 322844 |
| BCDR01000015.1 | 100 | 801 | 0 | 0 | 322196 | 322996 |
| BCDN01000016.1 | 100 | 801 | 0 | 0 | 271540 | 272340 |
| BCDM01000026.1 | 100 | 801 | 0 | 0 | 30242 | 29442 |
| BCDL01000015.1 | 100 | 801 | 0 | 0 | 30044 | 29244 |
| BCDK01000005.1 | 100 | 801 | 0 | 0 | 165273 | 166073 |
| BCDJ01000011.1 | 100 | 801 | 0 | 0 | 322202 | 323002 |
| BCDI01000007.1 | 100 | 801 | 0 | 0 | 30012 | 29212 |
| BCDH01000008.1 | 100 | 801 | 0 | 0 | 318744 | 319544 |
| BCDG01000018.1 | 100 | 801 | 0 | 0 | 30023 | 29223 |
| BCDE01000026.1 | 100 | 801 | 0 | 0 | 30031 | 29231 |
| BCDD01000024.1 | 100 | 801 | 0 | 0 | 30013 | 29213 |
| BCDC01000021.1 | 100 | 801 | 0 | 0 | 322057 | 322857 |
| BCDB01000019.1 | 100 | 801 | 0 | 0 | 30029 | 29229 |
| BCDA01000002.1 | 100 | 801 | 0 | 0 | 30017 | 29217 |
| BCCZ01000008.1 | 100 | 801 | 0 | 0 | 129199 | 129999 |
| BCCY01000027.1 | 100 | 801 | 0 | 0 | 271494 | 272294 |
| BCCW01000019.1 | 100 | 801 | 0 | 0 | 265077 | 265877 |
| BCCU01000016.1 | 100 | 801 | 0 | 0 | 30007 | 29207 |
| BCCS01000022.1 | 100 | 801 | 0 | 0 | 30023 | 29223 |
| BCCR01000018.1 | 100 | 801 | 0 | 0 | 277784 | 278584 |
| BCCQ01000003.1 | 100 | 801 | 0 | 0 | 30033 | 29233 |
| BCCP01000005.1 | 100 | 801 | 0 | 0 | 271453 | 272253 |
| BCCE01000011.1 | 100 | 801 | 0 | 0 | 30057 | 29257 |
| BCCB01000005.1 | 100 | 801 | 0 | 0 | 110796 | 111596 |
| BCBY01000009.1 | 100 | 801 | 0 | 0 | 30031 | 29231 |
| CP065431.1 | 100 | 801 | 0 | 0 | 1005728 | 1006528 |
| CP058742.1 | 100 | 801 | 0 | 0 | 937011 | 937811 |
| CP030022.1 | 100 | 801 | 0 | 0 | 1005355 | 1006155 |
| CP030017.1 | 100 | 801 | 0 | 0 | 1003557 | 1004357 |
| JABXEX010000001.1 | 100 | 801 | 0 | 0 | 29964 | 29164 |
| JABXEY010000002.1 | 100 | 801 | 0 | 0 | 72539 | 73339 |
| JABLQW010000002.1 | 100 | 801 | 0 | 0 | 259755 | 260555 |
| JABLQX010000002.1 | 100 | 801 | 0 | 0 | 216968 | 217768 |
| JABLQZ010000005.1 | 100 | 801 | 0 | 0 | 136263 | 137063 |
| JABLQY010000003.1 | 100 | 801 | 0 | 0 | 226472 | 227272 |
| JABLRA010000002.1 | 100 | 801 | 0 | 0 | 271119 | 271919 |
| JABLRB010000002.1 | 100 | 801 | 0 | 0 | 30056 | 29256 |
| JABLRC010000001.1 | 100 | 801 | 0 | 0 | 338319 | 339119 |
| JABLRE010000002.1 | 100 | 801 | 0 | 0 | 102093 | 101293 |
| JABLRD010000001.1 | 100 | 801 | 0 | 0 | 355330 | 356130 |
| JABLRF010000005.1 | 100 | 801 | 0 | 0 | 136157 | 136957 |
| JABLRG010000006.1 | 100 | 801 | 0 | 0 | 69030 | 68230 |
| JABLRH010000001.1 | 100 | 801 | 0 | 0 | 330960 | 331760 |
| JABLRI010000001.1 | 100 | 801 | 0 | 0 | 30054 | 29254 |
| JABLRJ010000005.1 | 100 | 801 | 0 | 0 | 94819 | 95619 |
| JABLRK010000003.1 | 100 | 801 | 0 | 0 | 128936 | 129736 |
| JABLRL010000002.1 | 100 | 801 | 0 | 0 | 215788 | 216588 |
| JABLRM010000005.1 | 100 | 801 | 0 | 0 | 30054 | 29254 |
| JABLRQ010000001.1 | 100 | 801 | 0 | 0 | 332039 | 332839 |
| JABLRR010000001.1 | 100 | 801 | 0 | 0 | 332049 | 332849 |
| JABLRN010000001.1 | 100 | 801 | 0 | 0 | 274970 | 275770 |
| JABLRS010000002.1 | 100 | 801 | 0 | 0 | 45816 | 45016 |
| JABLRT010000001.1 | 100 | 801 | 0 | 0 | 332069 | 332869 |
| JABLRU010000001.1 | 100 | 801 | 0 | 0 | 339932 | 340732 |
| JABLFH010000001.1 | 100 | 801 | 0 | 0 | 30028 | 29228 |
| JABLFF010000001.1 | 100 | 801 | 0 | 0 | 30037 | 29237 |
| JABLFI010000003.1 | 100 | 801 | 0 | 0 | 171410 | 172210 |
| JABLFK010000005.1 | 100 | 801 | 0 | 0 | 114924 | 115724 |
| JABLFL010000006.1 | 100 | 801 | 0 | 0 | 83512 | 84312 |
| JABLFM010000004.1 | 100 | 801 | 0 | 0 | 136806 | 137606 |
| JABLFP010000001.1 | 100 | 801 | 0 | 0 | 30037 | 29237 |
| JABLFO010000006.1 | 100 | 801 | 0 | 0 | 117624 | 118424 |
| JABLFU010000005.1 | 100 | 801 | 0 | 0 | 117624 | 118424 |
| JABLFX010000004.1 | 100 | 801 | 0 | 0 | 148104 | 148904 |
| JABLFV010000001.1 | 100 | 801 | 0 | 0 | 102105 | 101305 |
| JABLGB010000003.1 | 100 | 801 | 0 | 0 | 30037 | 29237 |
| JABLFY010000003.1 | 100 | 801 | 0 | 0 | 30497 | 29697 |
| JABLGD010000004.1 | 100 | 801 | 0 | 0 | 30037 | 29237 |
| JABLGE010000004.1 | 100 | 801 | 0 | 0 | 30037 | 29237 |
| JABLGG010000001.1 | 100 | 801 | 0 | 0 | 239124 | 239924 |
| JABLGH010000004.1 | 100 | 801 | 0 | 0 | 30037 | 29237 |
| JABLGK010000005.1 | 100 | 801 | 0 | 0 | 83499 | 84299 |
| JABLGN010000005.1 | 100 | 801 | 0 | 0 | 30080 | 29280 |
| JABLGM010000002.1 | 100 | 801 | 0 | 0 | 271294 | 272094 |
| JABLGR010000006.1 | 100 | 801 | 0 | 0 | 30080 | 29280 |
| JABLHH010000001.1 | 100 | 801 | 0 | 0 | 333958 | 334758 |
| JABLHI010000001.1 | 100 | 801 | 0 | 0 | 341380 | 342180 |
| JABLHK010000005.1 | 100 | 801 | 0 | 0 | 148241 | 149041 |
| JABLHP010000005.1 | 100 | 801 | 0 | 0 | 136878 | 137678 |
| JABLHX010000001.1 | 100 | 801 | 0 | 0 | 343336 | 344136 |
| JABLHW010000001.1 | 100 | 801 | 0 | 0 | 271354 | 272154 |
| JABLHY010000003.1 | 100 | 801 | 0 | 0 | 271133 | 271933 |
| JABLIB010000001.1 | 100 | 801 | 0 | 0 | 271133 | 271933 |
| JABLIN010000001.1 | 100 | 801 | 0 | 0 | 331856 | 332656 |
| JABLIH010000002.1 | 100 | 801 | 0 | 0 | 276312 | 277112 |
| JABLIQ010000003.1 | 100 | 801 | 0 | 0 | 260076 | 260876 |
| JABLIR010000001.1 | 100 | 801 | 0 | 0 | 322410 | 323210 |
| JABLIT010000002.1 | 100 | 801 | 0 | 0 | 271300 | 272100 |
| JABLFR010000005.1 | 100 | 801 | 0 | 0 | 115040 | 115840 |
| JABLIS010000007.1 | 100 | 801 | 0 | 0 | 112995 | 113795 |
| JABLIU010000002.1 | 100 | 801 | 0 | 0 | 259991 | 260791 |
| JABLFQ010000003.1 | 100 | 801 | 0 | 0 | 30037 | 29237 |
| JABLIZ010000003.1 | 100 | 801 | 0 | 0 | 271018 | 271818 |
| JABLJH010000006.1 | 100 | 801 | 0 | 0 | 94128 | 94928 |
| JABLJJ010000006.1 | 100 | 801 | 0 | 0 | 30087 | 29287 |
| JABLGV010000003.1 | 100 | 801 | 0 | 0 | 259939 | 260739 |
| JABLJL010000002.1 | 100 | 801 | 0 | 0 | 343196 | 343996 |
| JABLJN010000006.1 | 100 | 801 | 0 | 0 | 114974 | 115774 |
| JABLGS010000006.1 | 100 | 801 | 0 | 0 | 30080 | 29280 |
| JABLHE010000001.1 | 100 | 801 | 0 | 0 | 331656 | 332456 |
| JABLGW010000007.1 | 100 | 801 | 0 | 0 | 95173 | 95973 |
| JABLHA010000001.1 | 100 | 801 | 0 | 0 | 30080 | 29280 |
| JABLFC010000011.1 | 100 | 801 | 0 | 0 | 34738 | 35538 |
| JABLHC010000001.1 | 100 | 801 | 0 | 0 | 30078 | 29278 |
| JABLJB010000002.1 | 100 | 801 | 0 | 0 | 259768 | 260568 |
| JABLHB010000001.1 | 100 | 801 | 0 | 0 | 259939 | 260739 |
| JABLJX010000001.1 | 100 | 801 | 0 | 0 | 332094 | 332894 |
| JABLKD010000001.1 | 100 | 801 | 0 | 0 | 30080 | 29280 |
| JABLKA010000001.1 | 100 | 801 | 0 | 0 | 343360 | 344160 |
| JABLKB010000001.1 | 100 | 801 | 0 | 0 | 413381 | 414181 |
| JABLKH010000001.1 | 100 | 801 | 0 | 0 | 343166 | 343966 |
| JABLKG010000002.1 | 100 | 801 | 0 | 0 | 30080 | 29280 |
| JABLKI010000001.1 | 100 | 801 | 0 | 0 | 393936 | 394736 |
| JABLJT010000001.1 | 100 | 801 | 0 | 0 | 30081 | 29281 |
| JABLJU010000001.1 | 100 | 801 | 0 | 0 | 347055 | 347855 |
| JABLQA010000002.1 | 100 | 801 | 0 | 0 | 148138 | 148938 |
| JABLPY010000002.1 | 100 | 801 | 0 | 0 | 148046 | 148846 |
| JABLPZ010000001.1 | 100 | 801 | 0 | 0 | 259997 | 260797 |
| JABLQC010000002.1 | 100 | 801 | 0 | 0 | 259951 | 260751 |
| JABLPT010000001.1 | 100 | 801 | 0 | 0 | 338323 | 339123 |
| JABLQB010000002.1 | 100 | 801 | 0 | 0 | 30275 | 29475 |
| JABLQE010000001.1 | 100 | 801 | 0 | 0 | 343971 | 344771 |
| JABLQD010000001.1 | 100 | 801 | 0 | 0 | 215190 | 215990 |
| JABLQG010000001.1 | 100 | 801 | 0 | 0 | 30054 | 29254 |
| JABLQF010000001.1 | 100 | 801 | 0 | 0 | 271260 | 272060 |
| JABLQH010000001.1 | 100 | 801 | 0 | 0 | 30054 | 29254 |
| JABLQI010000001.1 | 100 | 801 | 0 | 0 | 259981 | 260781 |
| JABLPW010000002.1 | 100 | 801 | 0 | 0 | 269947 | 270747 |
| JABLQJ010000006.1 | 100 | 801 | 0 | 0 | 136858 | 137658 |
| JABLQK010000002.1 | 100 | 801 | 0 | 0 | 222794 | 223594 |
| JABLPV010000001.1 | 100 | 801 | 0 | 0 | 50425 | 49625 |
| JABLQL010000006.1 | 100 | 801 | 0 | 0 | 65207 | 66007 |
| JABLPU010000002.1 | 100 | 801 | 0 | 0 | 215191 | 215991 |
| JABLQM010000001.1 | 100 | 801 | 0 | 0 | 27028 | 26228 |
| JABLPX010000003.1 | 100 | 801 | 0 | 0 | 148716 | 149516 |
| JABLQQ010000002.1 | 100 | 801 | 0 | 0 | 270218 | 271018 |
| JABLQR010000001.1 | 100 | 801 | 0 | 0 | 215787 | 216587 |
| JABLPS010000001.1 | 100 | 801 | 0 | 0 | 30927 | 30127 |
| JABLQS010000007.1 | 100 | 801 | 0 | 0 | 93644 | 94444 |
| JABLQT010000001.1 | 100 | 801 | 0 | 0 | 338326 | 339126 |
| JABLQU010000002.1 | 100 | 801 | 0 | 0 | 30063 | 29263 |
| JABLQV010000004.1 | 100 | 801 | 0 | 0 | 148143 | 148943 |
| JABLQP010000001.1 | 100 | 801 | 0 | 0 | 338309 | 339109 |
| JABLQN010000003.1 | 100 | 801 | 0 | 0 | 136859 | 137659 |
| JABLQO010000002.1 | 100 | 801 | 0 | 0 | 221456 | 222256 |
| JABKSV010000001.1 | 100 | 801 | 0 | 0 | 332050 | 332850 |
| JABKSU010000001.1 | 100 | 801 | 0 | 0 | 332190 | 332990 |
| JABKTO010000002.1 | 100 | 801 | 0 | 0 | 259962 | 260762 |
| JABKTR010000001.1 | 100 | 801 | 0 | 0 | 259961 | 260761 |
| JABKTQ010000002.1 | 100 | 801 | 0 | 0 | 259975 | 260775 |
| JABKUV010000001.1 | 100 | 801 | 0 | 0 | 341271 | 342071 |
| JABKVC010000001.1 | 100 | 801 | 0 | 0 | 329766 | 330566 |
| JABKSR010000001.1 | 100 | 801 | 0 | 0 | 332065 | 332865 |
| JABKSS010000002.1 | 100 | 801 | 0 | 0 | 259962 | 260762 |
| JABKVH010000001.1 | 100 | 801 | 0 | 0 | 329761 | 330561 |
| JABLRO010000001.1 | 100 | 801 | 0 | 0 | 30054 | 29254 |
| JABKUI010000001.1 | 100 | 801 | 0 | 0 | 318291 | 319091 |
| JABKUN010000001.1 | 100 | 801 | 0 | 0 | 329982 | 330782 |
| JABKZL010000003.1 | 100 | 801 | 0 | 0 | 30033 | 29233 |
| JABKZN010000003.1 | 100 | 801 | 0 | 0 | 146694 | 147494 |
| JABKZM010000001.1 | 100 | 801 | 0 | 0 | 230686 | 231486 |
| JABKZU010000004.1 | 100 | 801 | 0 | 0 | 45765 | 44965 |
| JABLAG010000011.1 | 100 | 801 | 0 | 0 | 34723 | 35523 |
| JABKZV010000011.1 | 100 | 801 | 0 | 0 | 30039 | 29239 |
| JABLAF010000010.1 | 100 | 801 | 0 | 0 | 34723 | 35523 |
| JABLAU010000011.1 | 100 | 801 | 0 | 0 | 34737 | 35537 |
| JABLAX010000003.1 | 100 | 801 | 0 | 0 | 30025 | 29225 |
| JABLBH010000014.1 | 100 | 801 | 0 | 0 | 34738 | 35538 |
| JABLAM010000003.1 | 100 | 801 | 0 | 0 | 182827 | 183627 |
| JABLBF010000001.1 | 100 | 801 | 0 | 0 | 30025 | 29225 |
| JABLBG010000001.1 | 100 | 801 | 0 | 0 | 30037 | 29237 |
| JABLBT010000003.1 | 100 | 801 | 0 | 0 | 148103 | 148903 |
| JABLDI010000004.1 | 100 | 801 | 0 | 0 | 30037 | 29237 |
| JABLDH010000004.1 | 100 | 801 | 0 | 0 | 117624 | 118424 |
| JABLDK010000012.1 | 100 | 801 | 0 | 0 | 34737 | 35537 |
| JABLDM010000005.1 | 100 | 801 | 0 | 0 | 30037 | 29237 |
| JABLDO010000009.1 | 100 | 801 | 0 | 0 | 34739 | 35539 |
| JABLDR010000009.1 | 100 | 801 | 0 | 0 | 45888 | 46688 |
| JABLDQ010000002.1 | 100 | 801 | 0 | 0 | 182942 | 183742 |
| JABLDS010000002.1 | 100 | 801 | 0 | 0 | 182723 | 183523 |
| JABLDE010000002.1 | 100 | 801 | 0 | 0 | 206270 | 207070 |
| JABLEE010000002.1 | 100 | 801 | 0 | 0 | 30075 | 29275 |
| JABLEB010000002.1 | 100 | 801 | 0 | 0 | 189301 | 190101 |
| JABLEC010000004.1 | 100 | 801 | 0 | 0 | 30037 | 29237 |
| JABLEF010000008.1 | 100 | 801 | 0 | 0 | 45887 | 46687 |
| JABLED010000003.1 | 100 | 801 | 0 | 0 | 148824 | 149624 |
| JABLBU010000002.1 | 100 | 801 | 0 | 0 | 183151 | 183951 |
| JABLEH010000014.1 | 100 | 801 | 0 | 0 | 10839 | 11639 |
| JABLEK010000015.1 | 100 | 801 | 0 | 0 | 30037 | 29237 |
| JABLEJ010000005.1 | 100 | 801 | 0 | 0 | 83500 | 84300 |
| JABLDX010000003.1 | 100 | 801 | 0 | 0 | 183613 | 184413 |
| JABLEZ010000001.1 | 100 | 801 | 0 | 0 | 107221 | 106421 |
| JABLDD010000001.1 | 100 | 801 | 0 | 0 | 206271 | 207071 |
| JABLEM010000013.1 | 100 | 801 | 0 | 0 | 30037 | 29237 |
| JABLEY010000002.1 | 100 | 801 | 0 | 0 | 182522 | 183322 |
| JABLEP010000007.1 | 100 | 801 | 0 | 0 | 34737 | 35537 |
| JABLEO010000004.1 | 100 | 801 | 0 | 0 | 98361 | 97561 |
| JAAVLK010000001.1 | 100 | 801 | 0 | 0 | 324473 | 325273 |
| PKOW01000001.1 | 100 | 801 | 0 | 0 | 456805 | 457605 |
| PKOX01000002.1 | 100 | 801 | 0 | 0 | 30261 | 29461 |
| PKOV01000001.1 | 100 | 801 | 0 | 0 | 342698 | 343498 |
| WODB01000001.1 | 100 | 801 | 0 | 0 | 324473 | 325273 |
| VIFJ01000001.1 | 100 | 801 | 0 | 0 | 730011 | 729211 |
| VIFD01000001.1 | 100 | 801 | 0 | 0 | 730375 | 729575 |
| VIFA01000001.1 | 100 | 801 | 0 | 0 | 1393865 | 1394665 |
| VIEY01000001.1 | 100 | 801 | 0 | 0 | 1579707 | 1580507 |
| VIEW01000001.1 | 100 | 801 | 0 | 0 | 998785 | 999585 |
| VIEJ01000002.1 | 100 | 801 | 0 | 0 | 257987 | 258787 |
| VIEC01000006.1 | 100 | 801 | 0 | 0 | 117682 | 118482 |
| VIEE01000002.1 | 100 | 801 | 0 | 0 | 259966 | 260766 |
| VIEG01000002.1 | 100 | 801 | 0 | 0 | 30084 | 29284 |
| RZIC01000008.1 | 100 | 801 | 0 | 0 | 5678 | 4878 |
| RSDH01000005.1 | 100 | 801 | 0 | 0 | 69582 | 70382 |
| RSDQ01000004.1 | 100 | 801 | 0 | 0 | 15066 | 14266 |
| RSDI01000004.1 | 100 | 801 | 0 | 0 | 15066 | 14266 |
| PYUF01000012.1 | 100 | 801 | 0 | 0 | 30084 | 29284 |
| PQGR01000001.1 | 100 | 801 | 0 | 0 | 334414 | 335214 |
| PQGQ01000001.1 | 100 | 801 | 0 | 0 | 30018 | 29218 |
| PQGN01000005.1 | 100 | 801 | 0 | 0 | 114080 | 114880 |
| PQGO01000001.1 | 100 | 801 | 0 | 0 | 338283 | 339083 |
| PQGM01000001.1 | 100 | 801 | 0 | 0 | 338033 | 338833 |
| PQGP01000004.1 | 100 | 801 | 0 | 0 | 140540 | 141340 |
| POOA01000077.1 | 100 | 801 | 0 | 0 | 52651 | 53451 |
| POMR01000143.1 | 100 | 801 | 0 | 0 | 29933 | 29133 |
| POOC01000215.1 | 100 | 801 | 0 | 0 | 29905 | 29105 |
| POKR01000204.1 | 100 | 801 | 0 | 0 | 29899 | 29099 |
| POIX01000024.1 | 100 | 801 | 0 | 0 | 30325 | 29525 |
| POIW01000202.1 | 100 | 801 | 0 | 0 | 29893 | 29093 |
| JRHU01000014.1 | 100 | 801 | 0 | 0 | 50437 | 51237 |
| ASBK01000032.1 | 100 | 801 | 0 | 0 | 1 | 801 |
| ASBG01000032.1 | 100 | 801 | 0 | 0 | 1 | 801 |
| ASBF01000329.1 | 100 | 801 | 0 | 0 | 1 | 801 |
| ASBD01000032.1 | 100 | 801 | 0 | 0 | 1 | 801 |
| ASBC01000032.1 | 100 | 801 | 0 | 0 | 1 | 801 |
| ASAZ01000032.1 | 100 | 801 | 0 | 0 | 1 | 801 |
| ASAX01000305.1 | 100 | 801 | 0 | 0 | 1 | 801 |
| ASAV01000327.1 | 100 | 801 | 0 | 0 | 1 | 801 |
| ASAY01000329.1 | 100 | 801 | 0 | 0 | 1 | 801 |
| ASAS01000329.1 | 100 | 801 | 0 | 0 | 1 | 801 |
| ASAU01000328.1 | 100 | 801 | 0 | 0 | 1 | 801 |
| ASAT01000032.1 | 100 | 801 | 0 | 0 | 1 | 801 |
| ASCE01000032.1 | 100 | 801 | 0 | 0 | 1 | 801 |
| ASBJ01000329.1 | 100 | 801 | 0 | 0 | 1 | 801 |
| ASBI01000032.1 | 100 | 801 | 0 | 0 | 1 | 801 |
| ASBH01000032.1 | 100 | 801 | 0 | 0 | 1 | 801 |
| ASBE01000329.1 | 100 | 801 | 0 | 0 | 1 | 801 |
| ASAW01000032.1 | 100 | 801 | 0 | 0 | 1 | 801 |
| ASBB01000328.1 | 100 | 801 | 0 | 0 | 1 | 801 |
| ASAR01000500.1 | 100 | 801 | 0 | 0 | 1 | 801 |
| ASBA01000329.1 | 100 | 801 | 0 | 0 | 1 | 801 |
| CP024050.1 | 100 | 801 | 0 | 0 | 1051941 | 1052741 |
| NHOL01000041.1 | 100 | 801 | 0 | 0 | 2940 | 3740 |
| CP016175.1 | 100 | 801 | 0 | 0 | 998451 | 999251 |
| CP020863.1 | 100 | 801 | 0 | 0 | 1025547 | 1026347 |
| AYSB01000007.1 | 100 | 801 | 0 | 0 | 84139 | 84939 |
| CP018908.1 | 100 | 801 | 0 | 0 | 1001753 | 1002553 |
| MNPY01000001.1 | 100 | 801 | 0 | 0 | 30722 | 29922 |
| LDOJ01000045.1 | 100 | 801 | 0 | 0 | 21659 | 22459 |
| LDOM01000009.1 | 100 | 801 | 0 | 0 | 175707 | 176507 |
| CP007497.1 | 100 | 801 | 0 | 0 | 1025372 | 1026172 |
| CDXN01000001.1 | 100 | 801 | 0 | 0 | 30239 | 29439 |
| CEBO01000008.1 | 100 | 801 | 0 | 0 | 30027 | 29227 |
| CEKN01000003.1 | 100 | 801 | 0 | 0 | 30017 | 29217 |
| CDXH01000034.1 | 100 | 801 | 0 | 0 | 338475 | 339275 |
| CDXV01000022.1 | 100 | 801 | 0 | 0 | 421798 | 422598 |
| CEAV01000001.1 | 100 | 801 | 0 | 0 | 338299 | 339099 |
| CDTX01000011.1 | 100 | 801 | 0 | 0 | 342437 | 343237 |
| CDUN01000031.1 | 100 | 801 | 0 | 0 | 338289 | 339089 |
| CEBF01000022.1 | 100 | 801 | 0 | 0 | 421636 | 422436 |
| CDWS01000018.1 | 100 | 801 | 0 | 0 | 136128 | 136928 |
| CEHI01000054.1 | 100 | 801 | 0 | 0 | 271233 | 272033 |
| CEBJ01000035.1 | 100 | 801 | 0 | 0 | 30239 | 29439 |
| CEBR01000009.1 | 100 | 801 | 0 | 0 | 30025 | 29225 |
| CEGR01000055.1 | 100 | 801 | 0 | 0 | 145822 | 146622 |
| CEKP01000034.1 | 100 | 801 | 0 | 0 | 30009 | 29209 |
| CDWX01000039.1 | 100 | 801 | 0 | 0 | 259917 | 260717 |
| CEBW01000007.1 | 100 | 801 | 0 | 0 | 387157 | 387957 |
| CDTR01000005.1 | 100 | 801 | 0 | 0 | 30027 | 29227 |
| CDXC01000004.1 | 100 | 801 | 0 | 0 | 338298 | 339098 |
| CDVD01000010.1 | 100 | 801 | 0 | 0 | 330931 | 331731 |
| CDVX01000056.1 | 100 | 801 | 0 | 0 | 30239 | 29439 |
| CEBS01000006.1 | 100 | 801 | 0 | 0 | 30030 | 29230 |
| CDWK01000006.1 | 100 | 801 | 0 | 0 | 30027 | 29227 |
| CDUE01000001.1 | 100 | 801 | 0 | 0 | 338288 | 339088 |
| CEJY01000051.1 | 100 | 801 | 0 | 0 | 271252 | 272052 |
| CDVJ01000025.1 | 100 | 801 | 0 | 0 | 271389 | 272189 |
| CEJZ01000040.1 | 100 | 801 | 0 | 0 | 30011 | 29211 |
| CDYB01000001.1 | 100 | 801 | 0 | 0 | 30239 | 29439 |
| CDXS01000060.1 | 100 | 801 | 0 | 0 | 30239 | 29439 |
| CDWQ01000017.1 | 100 | 801 | 0 | 0 | 30027 | 29227 |
| CDUJ01000002.1 | 100 | 801 | 0 | 0 | 30027 | 29227 |
| CEHF01000017.1 | 100 | 801 | 0 | 0 | 240744 | 241544 |
| CDWA01000008.1 | 100 | 801 | 0 | 0 | 30027 | 29227 |
| CDSG01000004.1 | 100 | 801 | 0 | 0 | 30239 | 29439 |
| CEHG01000030.1 | 100 | 801 | 0 | 0 | 30268 | 29468 |
| CEDI01000022.1 | 100 | 801 | 0 | 0 | 258688 | 259488 |
| CEJF01000055.1 | 100 | 801 | 0 | 0 | 258681 | 259481 |
| CDWE01000028.1 | 100 | 801 | 0 | 0 | 82918 | 83718 |
| CDWN01000005.1 | 100 | 801 | 0 | 0 | 30027 | 29227 |
| CDWW01000002.1 | 100 | 801 | 0 | 0 | 136128 | 136928 |
| CDVG01000016.1 | 100 | 801 | 0 | 0 | 402993 | 403793 |
| CDSS01000015.1 | 100 | 801 | 0 | 0 | 344135 | 344935 |
| CECH01000001.1 | 100 | 801 | 0 | 0 | 349602 | 350402 |
| CDWB01000001.1 | 100 | 801 | 0 | 0 | 30027 | 29227 |
| CEDQ01000036.1 | 100 | 801 | 0 | 0 | 271070 | 271870 |
| CEJN01000021.1 | 100 | 801 | 0 | 0 | 321802 | 322602 |
| CEBI01000005.1 | 100 | 801 | 0 | 0 | 30074 | 29274 |
| CEIN01000048.1 | 100 | 801 | 0 | 0 | 114908 | 115708 |
| CDVE01000034.1 | 100 | 801 | 0 | 0 | 30019 | 29219 |
| CEDL01000052.1 | 100 | 801 | 0 | 0 | 48373 | 49173 |
| CEBU01000062.1 | 100 | 801 | 0 | 0 | 30027 | 29227 |
| CDVU01000005.1 | 100 | 801 | 0 | 0 | 349576 | 350376 |
| CDTD01000036.1 | 100 | 801 | 0 | 0 | 271549 | 272349 |
| CDUG01000025.1 | 100 | 801 | 0 | 0 | 338291 | 339091 |
| CDWP01000002.1 | 100 | 801 | 0 | 0 | 193044 | 193844 |
| CDTT01000003.1 | 100 | 801 | 0 | 0 | 338301 | 339101 |
| CDUD01000003.1 | 100 | 801 | 0 | 0 | 338291 | 339091 |
| CDSO01000014.1 | 100 | 801 | 0 | 0 | 410354 | 411154 |
| CDTQ01000050.1 | 100 | 801 | 0 | 0 | 271096 | 271896 |
| CDXA01000030.1 | 100 | 801 | 0 | 0 | 354641 | 355441 |
| CEBL01000006.1 | 100 | 801 | 0 | 0 | 338379 | 339179 |
| CEKC01000050.1 | 100 | 801 | 0 | 0 | 114798 | 115598 |
| CEJX01000019.1 | 100 | 801 | 0 | 0 | 30011 | 29211 |
| CDWC01000007.1 | 100 | 801 | 0 | 0 | 387074 | 387874 |
| CDXK01000001.1 | 100 | 801 | 0 | 0 | 343942 | 344742 |
| CDUC01000003.1 | 100 | 801 | 0 | 0 | 30027 | 29227 |
| CEGQ01000048.1 | 100 | 801 | 0 | 0 | 329387 | 330187 |
| CDWT01000002.1 | 100 | 801 | 0 | 0 | 110718 | 111518 |
| CDSW01000010.1 | 100 | 801 | 0 | 0 | 30027 | 29227 |
| CEBG01000002.1 | 100 | 801 | 0 | 0 | 30027 | 29227 |
| CDUU01000019.1 | 100 | 801 | 0 | 0 | 338288 | 339088 |
| CDTJ01000015.1 | 100 | 801 | 0 | 0 | 88999 | 89799 |
| CDUW01000054.1 | 100 | 801 | 0 | 0 | 30027 | 29227 |
| CDTO01000005.1 | 100 | 801 | 0 | 0 | 338299 | 339099 |
| CDTE01000006.1 | 100 | 801 | 0 | 0 | 30027 | 29227 |
| CEDK01000054.1 | 100 | 801 | 0 | 0 | 259919 | 260719 |
| CDVQ01000017.1 | 100 | 801 | 0 | 0 | 30027 | 29227 |
| CDTI01000009.1 | 100 | 801 | 0 | 0 | 30027 | 29227 |
| CEKB01000019.1 | 100 | 801 | 0 | 0 | 271075 | 271875 |
| CDWZ01000028.1 | 100 | 801 | 0 | 0 | 30239 | 29439 |
| CEBC01000035.1 | 100 | 801 | 0 | 0 | 349596 | 350396 |
| CDTV01000025.1 | 100 | 801 | 0 | 0 | 338302 | 339102 |
| CDUI01000039.1 | 100 | 801 | 0 | 0 | 30027 | 29227 |
| CDSX01000036.1 | 100 | 801 | 0 | 0 | 30027 | 29227 |
| CECK01000041.1 | 100 | 801 | 0 | 0 | 338379 | 339179 |
| CDVO01000002.1 | 100 | 801 | 0 | 0 | 30027 | 29227 |
| CDTK01000021.1 | 100 | 801 | 0 | 0 | 338485 | 339285 |
| CDVW01000011.1 | 100 | 801 | 0 | 0 | 30027 | 29227 |
| CDVN01000036.1 | 100 | 801 | 0 | 0 | 35356 | 34556 |
| CEJV01000014.1 | 100 | 801 | 0 | 0 | 341194 | 341994 |
| CEBB01000005.1 | 100 | 801 | 0 | 0 | 30239 | 29439 |
| CDXU01000010.1 | 100 | 801 | 0 | 0 | 349670 | 350470 |
| CEKK01000052.1 | 100 | 801 | 0 | 0 | 271067 | 271867 |
| CEBM01000016.1 | 100 | 801 | 0 | 0 | 30027 | 29227 |
| CEHQ01000054.1 | 100 | 801 | 0 | 0 | 49081 | 49881 |
| CDXX01000052.1 | 100 | 801 | 0 | 0 | 30027 | 29227 |
| CDWV01000018.1 | 100 | 801 | 0 | 0 | 60570 | 61370 |
| CEKM01000016.1 | 100 | 801 | 0 | 0 | 271231 | 272031 |
| CEIF01000042.1 | 100 | 801 | 0 | 0 | 271187 | 271987 |
| CEIJ01000019.1 | 100 | 801 | 0 | 0 | 30011 | 29211 |
| CDXO01000034.1 | 100 | 801 | 0 | 0 | 30027 | 29227 |
| CDWH01000004.1 | 100 | 801 | 0 | 0 | 30027 | 29227 |
| CEJJ01000050.1 | 100 | 801 | 0 | 0 | 118701 | 119501 |
| CEJH01000055.1 | 100 | 801 | 0 | 0 | 80914 | 81714 |
| CDSP01000028.1 | 100 | 801 | 0 | 0 | 344155 | 344955 |
| CDUO01000051.1 | 100 | 801 | 0 | 0 | 270195 | 270995 |
| CDUQ01000006.1 | 100 | 801 | 0 | 0 | 30027 | 29227 |
| CDTM01000008.1 | 100 | 801 | 0 | 0 | 349651 | 350451 |
| CEKD01000053.1 | 100 | 801 | 0 | 0 | 270938 | 271738 |
| CDVI01000005.1 | 100 | 801 | 0 | 0 | 343986 | 344786 |
| CEJU01000003.1 | 100 | 801 | 0 | 0 | 312417 | 313217 |
| CDSH01000046.1 | 100 | 801 | 0 | 0 | 30027 | 29227 |
| CDWO01000006.1 | 100 | 801 | 0 | 0 | 30027 | 29227 |
| CDTZ01000009.1 | 100 | 801 | 0 | 0 | 30027 | 29227 |
| CDVH01000001.1 | 100 | 801 | 0 | 0 | 30027 | 29227 |
| CDXG01000037.1 | 100 | 801 | 0 | 0 | 30239 | 29439 |
| CDVT01000029.1 | 100 | 801 | 0 | 0 | 193200 | 194000 |
| CDUK01000044.1 | 100 | 801 | 0 | 0 | 30027 | 29227 |
| CDUF01000004.1 | 100 | 801 | 0 | 0 | 337890 | 338690 |
| CEDA01000017.1 | 100 | 801 | 0 | 0 | 30023 | 29223 |
| CDTS01000049.1 | 100 | 801 | 0 | 0 | 30248 | 29448 |
| CDUM01000052.1 | 100 | 801 | 0 | 0 | 271272 | 272072 |
| CDTL01000002.1 | 100 | 801 | 0 | 0 | 30027 | 29227 |
| CDXM01000001.1 | 100 | 801 | 0 | 0 | 337890 | 338690 |
| CDVA01000020.1 | 100 | 801 | 0 | 0 | 338290 | 339090 |
| CEIU01000015.1 | 100 | 801 | 0 | 0 | 341216 | 342016 |
| CEIM01000010.1 | 100 | 801 | 0 | 0 | 271370 | 272170 |
| CDXW01000003.1 | 100 | 801 | 0 | 0 | 354680 | 355480 |
| CDUV01000007.1 | 100 | 801 | 0 | 0 | 30027 | 29227 |
| CECC01000019.1 | 100 | 801 | 0 | 0 | 30027 | 29227 |
| CEGS01000052.1 | 100 | 801 | 0 | 0 | 92485 | 93285 |
| CDYC01000152.1 | 100 | 801 | 0 | 0 | 271203 | 272003 |
| CDYD01000011.1 | 100 | 801 | 0 | 0 | 30027 | 29227 |
| CDUR01000018.1 | 100 | 801 | 0 | 0 | 73664 | 74464 |
| CDYO01000046.1 | 100 | 801 | 0 | 0 | 338285 | 339085 |
| CDTF01000031.1 | 100 | 801 | 0 | 0 | 30027 | 29227 |
| CDVS01000019.1 | 100 | 801 | 0 | 0 | 343274 | 344074 |
| CEKJ01000048.1 | 100 | 801 | 0 | 0 | 271183 | 271983 |
| CDXJ01000008.1 | 100 | 801 | 0 | 0 | 338300 | 339100 |
| CEKQ01000045.1 | 100 | 801 | 0 | 0 | 83497 | 84297 |
| CDVP01000012.1 | 100 | 801 | 0 | 0 | 271235 | 272035 |
| CDYW01000001.1 | 100 | 801 | 0 | 0 | 30239 | 29439 |
| CDUY01000021.1 | 100 | 801 | 0 | 0 | 30027 | 29227 |
| CEHA01000052.1 | 100 | 801 | 0 | 0 | 117601 | 118401 |
| CDUL01000067.1 | 100 | 801 | 0 | 0 | 30239 | 29439 |
| CDXI01000004.1 | 100 | 801 | 0 | 0 | 338299 | 339099 |
| CDWR01000006.1 | 100 | 801 | 0 | 0 | 136126 | 136926 |
| CELM01000028.1 | 100 | 801 | 0 | 0 | 30007 | 29207 |
| CEEY01000005.1 | 100 | 801 | 0 | 0 | 258686 | 259486 |
| CDWY01000031.1 | 100 | 801 | 0 | 0 | 30027 | 29227 |
| CDTB01000010.1 | 100 | 801 | 0 | 0 | 30027 | 29227 |
| CDVV01000001.1 | 100 | 801 | 0 | 0 | 30027 | 29227 |
| CEAW01000015.1 | 100 | 801 | 0 | 0 | 30027 | 29227 |
| CEAY01000048.1 | 100 | 801 | 0 | 0 | 349729 | 350529 |
| CDVC01000008.1 | 100 | 801 | 0 | 0 | 30027 | 29227 |
| CDUX01000013.1 | 100 | 801 | 0 | 0 | 30027 | 29227 |
| CECN01000009.1 | 100 | 801 | 0 | 0 | 30239 | 29439 |
| CDSU01000007.1 | 100 | 801 | 0 | 0 | 346021 | 346821 |
| CDWF01000003.1 | 100 | 801 | 0 | 0 | 338298 | 339098 |
| CDSQ01000157.1 | 100 | 801 | 0 | 0 | 30105 | 29305 |
| CDXQ01000003.1 | 100 | 801 | 0 | 0 | 349576 | 350376 |
| CDXP01000002.1 | 100 | 801 | 0 | 0 | 30027 | 29227 |
| CDXT01000058.1 | 100 | 801 | 0 | 0 | 82634 | 83434 |
| CECL01000003.1 | 100 | 801 | 0 | 0 | 30239 | 29439 |
| CEBZ01000023.1 | 100 | 801 | 0 | 0 | 349677 | 350477 |
| CDWU01000008.1 | 100 | 801 | 0 | 0 | 338298 | 339098 |
| CEBH01000011.1 | 100 | 801 | 0 | 0 | 30027 | 29227 |
| CDVK01000014.1 | 100 | 801 | 0 | 0 | 338337 | 339137 |
| CECQ01000013.1 | 100 | 801 | 0 | 0 | 114804 | 115604 |
| CEHD01000045.1 | 100 | 801 | 0 | 0 | 83484 | 84284 |
| CDSZ01000001.1 | 100 | 801 | 0 | 0 | 30027 | 29227 |
| CEEB01000058.1 | 100 | 801 | 0 | 0 | 114908 | 115708 |
| CEDX01000065.1 | 100 | 801 | 0 | 0 | 271224 | 272024 |
| CDUZ01000008.1 | 100 | 801 | 0 | 0 | 338279 | 339079 |
| CEJI01000053.1 | 100 | 801 | 0 | 0 | 148704 | 149504 |
| CDST01000005.1 | 100 | 801 | 0 | 0 | 343948 | 344748 |
| CECT01000010.1 | 100 | 801 | 0 | 0 | 258690 | 259490 |
| CDSK01000009.1 | 100 | 801 | 0 | 0 | 30027 | 29227 |
| CECO01000026.1 | 100 | 801 | 0 | 0 | 30239 | 29439 |
| CEKT01000036.1 | 100 | 801 | 0 | 0 | 30013 | 29213 |
| CEBP01000059.1 | 100 | 801 | 0 | 0 | 30239 | 29439 |
| CEBA01000050.1 | 100 | 801 | 0 | 0 | 349596 | 350396 |
| CDVF01000007.1 | 100 | 801 | 0 | 0 | 338332 | 339132 |
| CDTC01000006.1 | 100 | 801 | 0 | 0 | 338291 | 339091 |
| CDSN01000046.1 | 100 | 801 | 0 | 0 | 30027 | 29227 |
| CDWD01000010.1 | 100 | 801 | 0 | 0 | 271091 | 271891 |
| CEJR01000055.1 | 100 | 801 | 0 | 0 | 107611 | 108411 |
| CDXZ01000037.1 | 100 | 801 | 0 | 0 | 30027 | 29227 |
| CEIR01000051.1 | 100 | 801 | 0 | 0 | 271074 | 271874 |
| CEIX01000052.1 | 100 | 801 | 0 | 0 | 83485 | 84285 |
| CEKI01000033.1 | 100 | 801 | 0 | 0 | 30015 | 29215 |
| CDXE01000010.1 | 100 | 801 | 0 | 0 | 338298 | 339098 |
| CDXR01000009.1 | 100 | 801 | 0 | 0 | 338282 | 339082 |
| CEBN01000018.1 | 100 | 801 | 0 | 0 | 30027 | 29227 |
| CEJB01000049.1 | 100 | 801 | 0 | 0 | 259926 | 260726 |
| CDSR01000007.1 | 100 | 801 | 0 | 0 | 349693 | 350493 |
| CDSY01000011.1 | 100 | 801 | 0 | 0 | 271229 | 272029 |
| CDVL01000005.1 | 100 | 801 | 0 | 0 | 30239 | 29439 |
| CDXY01000004.1 | 100 | 801 | 0 | 0 | 30027 | 29227 |
| CECP01000008.1 | 100 | 801 | 0 | 0 | 30022 | 29222 |
| CDSL01000004.1 | 100 | 801 | 0 | 0 | 30027 | 29227 |
| CDSM01000005.1 | 100 | 801 | 0 | 0 | 30027 | 29227 |
| CEJQ01000024.1 | 100 | 801 | 0 | 0 | 321619 | 322419 |
| CEBK01000053.1 | 100 | 801 | 0 | 0 | 271285 | 272085 |
| CDUT01000017.1 | 100 | 801 | 0 | 0 | 73664 | 74464 |
| CDWL01000049.1 | 100 | 801 | 0 | 0 | 30027 | 29227 |
| CDTA01000049.1 | 100 | 801 | 0 | 0 | 259952 | 260752 |
| CDUA01000057.1 | 100 | 801 | 0 | 0 | 30239 | 29439 |
| CDTU01000051.1 | 100 | 801 | 0 | 0 | 30027 | 29227 |
| CDVM01000004.1 | 100 | 801 | 0 | 0 | 349678 | 350478 |
| CDXF01000008.1 | 100 | 801 | 0 | 0 | 30106 | 29306 |
| CEBD01000022.1 | 100 | 801 | 0 | 0 | 30239 | 29439 |
| CEJE01000003.1 | 100 | 801 | 0 | 0 | 341329 | 342129 |
| CEIK01000049.1 | 100 | 801 | 0 | 0 | 267602 | 268402 |
| CDYA01000012.1 | 100 | 801 | 0 | 0 | 30027 | 29227 |
| CEHE01000034.1 | 100 | 801 | 0 | 0 | 271184 | 271984 |
| CEAS01000033.1 | 100 | 801 | 0 | 0 | 30239 | 29439 |
| CDUB01000002.1 | 100 | 801 | 0 | 0 | 30027 | 29227 |
| CEHL01000049.1 | 100 | 801 | 0 | 0 | 114916 | 115716 |
| CEIP01000058.1 | 100 | 801 | 0 | 0 | 86198 | 86998 |
| CEHC01000053.1 | 100 | 801 | 0 | 0 | 260214 | 261014 |
| CEHZ01000049.1 | 100 | 801 | 0 | 0 | 21747 | 22547 |
| CDVZ01000003.1 | 100 | 801 | 0 | 0 | 338299 | 339099 |
| CDWM01000024.1 | 100 | 801 | 0 | 0 | 30025 | 29225 |
| CDTP01000012.1 | 100 | 801 | 0 | 0 | 30270 | 29470 |
| CEAT01000011.1 | 100 | 801 | 0 | 0 | 349602 | 350402 |
| CECX01000008.1 | 100 | 801 | 0 | 0 | 30025 | 29225 |
| CDSJ01000026.1 | 100 | 801 | 0 | 0 | 259968 | 260768 |
| CDTN01000002.1 | 100 | 801 | 0 | 0 | 337895 | 338695 |
| CEBX01000013.1 | 100 | 801 | 0 | 0 | 349598 | 350398 |
| CEFH01000057.1 | 100 | 801 | 0 | 0 | 90199 | 90999 |
| CDXL01000003.1 | 100 | 801 | 0 | 0 | 30027 | 29227 |
| CDVB01000010.1 | 100 | 801 | 0 | 0 | 30027 | 29227 |
| CEAU01000138.1 | 100 | 801 | 0 | 0 | 271495 | 272295 |
| CDSI01000025.1 | 100 | 801 | 0 | 0 | 391260 | 392060 |
| CEBT01000144.1 | 100 | 801 | 0 | 0 | 938 | 138 |
| CEBE01000005.1 | 100 | 801 | 0 | 0 | 271100 | 271900 |
| CDWG01000006.1 | 100 | 801 | 0 | 0 | 344205 | 345005 |
| CDVR01000013.1 | 100 | 801 | 0 | 0 | 30239 | 29439 |
| CDUP01000009.1 | 100 | 801 | 0 | 0 | 338285 | 339085 |
| CECD01000008.1 | 100 | 801 | 0 | 0 | 30027 | 29227 |
| CDTH01000001.1 | 100 | 801 | 0 | 0 | 259928 | 260728 |
| CDXD01000046.1 | 100 | 801 | 0 | 0 | 30027 | 29227 |
| CDTG01000007.1 | 100 | 801 | 0 | 0 | 338374 | 339174 |
| CDSV01000010.1 | 100 | 801 | 0 | 0 | 30027 | 29227 |
| ALMG01000019.1 | 100 | 801 | 0 | 0 | 132562 | 133362 |
| ALLS01000018.1 | 100 | 801 | 0 | 0 | 259923 | 260723 |
| ALLA01000002.1 | 100 | 801 | 0 | 0 | 20945 | 20145 |
| ALKR01000010.1 | 100 | 801 | 0 | 0 | 30035 | 29235 |
| ALKP01000002.1 | 100 | 801 | 0 | 0 | 30031 | 29231 |
| ALNE01000012.1 | 100 | 801 | 0 | 0 | 30031 | 29231 |
| CP003922.1 | 100 | 801 | 0 | 0 | 1052069 | 1052869 |
| CP003736.1 | 100 | 801 | 0 | 0 | 921681 | 922481 |
| CP002570.1 | 100 | 801 | 0 | 0 | 968538 | 969338 |
| CP002640.1 | 100 | 801 | 0 | 0 | 1001437 | 1002237 |
| CP002465.1 | 100 | 801 | 0 | 0 | 1049244 | 1050044 |
| AM946016.1 | 100 | 801 | 0 | 0 | 937062 | 937862 |
| FM252032.1 | 100 | 801 | 0 | 0 | 941887 | 941087 |
| FM252031.1 | 100 | 801 | 0 | 0 | 1025474 | 1026274 |
| CP000837.1 | 100 | 801 | 0 | 0 | 977191 | 977991 |
| CP000408.1 | 100 | 801 | 0 | 0 | 1025429 | 1026229 |
| NZ_FIOC01000001.1 | 99.875 | 801 | 1 | 0 | 338181 | 338981 |
| NZ_FIPA01000001.1 | 99.875 | 801 | 1 | 0 | 338136 | 338936 |
| NZ_FIOR01000001.1 | 99.875 | 801 | 1 | 0 | 338182 | 338982 |
| NZ_JASTQE010000001.1 | 99.875 | 801 | 1 | 0 | 30668 | 29868 |
| NZ_JAUTGR010000006.1 | 99.875 | 801 | 1 | 0 | 30068 | 29268 |
| NZ_JAKTDD010000007.1 | 99.875 | 801 | 1 | 0 | 64461 | 65261 |
| NZ_JAKTGC010000001.1 | 99.875 | 801 | 1 | 0 | 355547 | 356347 |
| NZ_JAMDIC010000002.1 | 99.875 | 801 | 1 | 0 | 271209 | 272009 |
| NZ_VZLG01000001.1 | 99.875 | 801 | 1 | 0 | 30270 | 29470 |
| NZ_VZMK01000001.1 | 99.875 | 801 | 1 | 0 | 343663 | 344463 |
| NZ_JAGUAG010000001.1 | 99.875 | 801 | 1 | 0 | 396655 | 397455 |
| NZ_CP047248.1 | 99.875 | 801 | 1 | 0 | 862784 | 861984 |
| NZ_CDVY01000015.1 | 99.875 | 801 | 1 | 0 | 338144 | 338944 |
| NZ_CDUH01000002.1 | 99.875 | 801 | 1 | 0 | 30050 | 29250 |
| NZ_CDUS01000004.1 | 99.875 | 801 | 1 | 0 | 338117 | 338917 |
| FIOC01000001.1 | 99.875 | 801 | 1 | 0 | 338181 | 338981 |
| FIPA01000001.1 | 99.875 | 801 | 1 | 0 | 338136 | 338936 |
| FIOR01000001.1 | 99.875 | 801 | 1 | 0 | 338182 | 338982 |
| JASTQE010000001.1 | 99.875 | 801 | 1 | 0 | 30668 | 29868 |
| DASGXL010000001.1 | 99.875 | 801 | 1 | 0 | 30168 | 29368 |
| DASGWY010000001.1 | 99.875 | 801 | 1 | 0 | 260066 | 260866 |
| DASGDM010000013.1 | 99.875 | 801 | 1 | 0 | 34591 | 33791 |
| DASGDG010000014.1 | 99.875 | 801 | 1 | 0 | 34591 | 33791 |
| DASGCW010000005.1 | 99.875 | 801 | 1 | 0 | 34630 | 33830 |
| DASGCU010000023.1 | 99.875 | 801 | 1 | 0 | 10716 | 9916 |
| DASGCV010000005.1 | 99.875 | 801 | 1 | 0 | 34631 | 33831 |
| DASGCT010000005.1 | 99.875 | 801 | 1 | 0 | 34629 | 33829 |
| DASGCS010000005.1 | 99.875 | 801 | 1 | 0 | 34630 | 33830 |
| DASGCR010000005.1 | 99.875 | 801 | 1 | 0 | 34630 | 33830 |
| DASGCQ010000008.1 | 99.875 | 801 | 1 | 0 | 34630 | 33830 |
| DASGCM010000005.1 | 99.875 | 801 | 1 | 0 | 34630 | 33830 |
| DASGCK010000014.1 | 99.875 | 801 | 1 | 0 | 34591 | 33791 |
| DASGCL010000015.1 | 99.875 | 801 | 1 | 0 | 28596 | 27796 |
| DASGCJ010000004.1 | 99.875 | 801 | 1 | 0 | 34594 | 33794 |
| DASGCH010000014.1 | 99.875 | 801 | 1 | 0 | 34630 | 33830 |
| DASGBV010000025.1 | 99.875 | 801 | 1 | 0 | 26785 | 25985 |
| DASGBW010000010.1 | 99.875 | 801 | 1 | 0 | 34591 | 33791 |
| DASGBU010000011.1 | 99.875 | 801 | 1 | 0 | 34592 | 33792 |
| DASGAX010000049.1 | 99.875 | 801 | 1 | 0 | 13525 | 12725 |
| DASFWO010000011.1 | 99.875 | 801 | 1 | 0 | 28606 | 27806 |
| DASFWB010000019.1 | 99.875 | 801 | 1 | 0 | 34629 | 33829 |
| DASFUS010000017.1 | 99.875 | 801 | 1 | 0 | 34591 | 33791 |
| DASFUO010000015.1 | 99.875 | 801 | 1 | 0 | 34629 | 33829 |
| DARZTV010000002.1 | 99.875 | 801 | 1 | 0 | 35982 | 35182 |
| JAUTGR010000006.1 | 99.875 | 801 | 1 | 0 | 30068 | 29268 |
| JAKTDD010000007.1 | 99.875 | 801 | 1 | 0 | 64461 | 65261 |
| JAKTGC010000001.1 | 99.875 | 801 | 1 | 0 | 355547 | 356347 |
| JAMDIC010000002.1 | 99.875 | 801 | 1 | 0 | 271209 | 272009 |
| VZLG01000001.1 | 99.875 | 801 | 1 | 0 | 30270 | 29470 |
| VZMK01000001.1 | 99.875 | 801 | 1 | 0 | 343663 | 344463 |
| JAGUAG010000001.1 | 99.875 | 801 | 1 | 0 | 396655 | 397455 |
| CP047248.1 | 99.875 | 801 | 1 | 0 | 862784 | 861984 |
| CDVY01000015.1 | 99.875 | 801 | 1 | 0 | 338144 | 338944 |
| CDUH01000002.1 | 99.875 | 801 | 1 | 0 | 30050 | 29250 |
| CDUS01000004.1 | 99.875 | 801 | 1 | 0 | 338117 | 338917 |
| NZ_JAASBS010000001.1 | 99.75 | 801 | 2 | 0 | 265739 | 266539 |
| JAASBS010000001.1 | 99.75 | 801 | 2 | 0 | 265739 | 266539 |
| NZ_LR594043.1 | 99.75 | 801 | 2 | 0 | 1029439 | 1030239 |
| NZ_CZHB01000030.1 | 99.75 | 801 | 2 | 0 | 34744 | 33944 |
| NZ_CZED01000027.1 | 99.75 | 801 | 2 | 0 | 2880 | 2080 |
| NZ_JAUTFG010000003.1 | 99.75 | 801 | 2 | 0 | 69911 | 70711 |
| NZ_JAUTEW010000004.1 | 99.75 | 801 | 2 | 0 | 34703 | 33903 |
| NZ_JABLJQ010000004.1 | 99.75 | 801 | 2 | 0 | 35650 | 34850 |
| NZ_JABKTX010000004.1 | 99.75 | 801 | 2 | 0 | 69892 | 70692 |
| NZ_JABKWF010000002.1 | 99.75 | 801 | 2 | 0 | 87174 | 86374 |
| NZ_JABKWK010000019.1 | 99.75 | 801 | 2 | 0 | 34753 | 33953 |
| NZ_ALLZ01000009.1 | 99.75 | 801 | 2 | 0 | 2765 | 1965 |
| NC_017950.1 | 99.75 | 801 | 2 | 0 | 890613 | 889813 |
| LR594043.1 | 99.75 | 801 | 2 | 0 | 1029439 | 1030239 |
| CZHB01000030.1 | 99.75 | 801 | 2 | 0 | 34744 | 33944 |
| CZED01000027.1 | 99.75 | 801 | 2 | 0 | 2880 | 2080 |
| DASGXM010000005.1 | 99.75 | 801 | 2 | 0 | 34591 | 33791 |
| DASGFY010000003.1 | 99.75 | 801 | 2 | 0 | 34591 | 33791 |
| DASGDJ010000032.1 | 99.75 | 801 | 2 | 0 | 2693 | 1893 |
| DASGBJ010000038.1 | 99.75 | 801 | 2 | 0 | 919 | 119 |
| DASFUT010000015.1 | 99.75 | 801 | 2 | 0 | 34591 | 33791 |
| DASFTC010000002.1 | 99.75 | 801 | 2 | 0 | 34525 | 33725 |
| DASFOK010000005.1 | 99.75 | 801 | 2 | 0 | 34591 | 33791 |
| DASFNY010000009.1 | 99.75 | 801 | 2 | 0 | 34666 | 33866 |
| DASFNB010000002.1 | 99.75 | 801 | 2 | 0 | 34591 | 33791 |
| DASFMN010000001.1 | 99.75 | 801 | 2 | 0 | 115214 | 116014 |
| DASFJM010000004.1 | 99.75 | 801 | 2 | 0 | 34591 | 33791 |
| DASFGQ010000017.1 | 99.75 | 801 | 2 | 0 | 34666 | 33866 |
| DASEPA010000032.1 | 99.75 | 801 | 2 | 0 | 2692 | 1892 |
| DASEIQ010000042.1 | 100 | 795 | 0 | 0 | 13517 | 12723 |
| DASEIL010000021.1 | 99.75 | 801 | 2 | 0 | 28401 | 27601 |
| DARZHP010000003.1 | 99.75 | 801 | 2 | 0 | 34591 | 33791 |
| JAUTFG010000003.1 | 99.75 | 801 | 2 | 0 | 69911 | 70711 |
| JAUTEW010000004.1 | 99.75 | 801 | 2 | 0 | 34703 | 33903 |
| JABLJQ010000004.1 | 99.75 | 801 | 2 | 0 | 35650 | 34850 |
| JABKTX010000004.1 | 99.75 | 801 | 2 | 0 | 69892 | 70692 |
| JABKWF010000002.1 | 99.75 | 801 | 2 | 0 | 87174 | 86374 |
| JABKWK010000019.1 | 99.75 | 801 | 2 | 0 | 34753 | 33953 |
| ALLZ01000009.1 | 99.75 | 801 | 2 | 0 | 2765 | 1965 |
| CP002651.1 | 99.75 | 801 | 2 | 0 | 890613 | 889813 |
| DASGAT010000011.1 | 99.875 | 797 | 1 | 0 | 28628 | 27832 |
| VIET01000001.1 | 99.75 | 801 | 1 | 1 | 727264 | 726465 |
| VIEM01000001.1 | 99.75 | 801 | 0 | 2 | 993440 | 994238 |
| NZ_JAVIHS010000007.1 | 99.625 | 801 | 3 | 0 | 7302 | 8102 |
| NZ_JAASBU010000048.1 | 99.625 | 801 | 3 | 0 | 60476 | 59676 |
| NZ_JABTYY010000001.1 | 99.625 | 801 | 3 | 0 | 12467 | 13267 |
| NZ_JABKSL010000041.1 | 99.625 | 801 | 3 | 0 | 12496 | 13296 |
| NZ_JABKWG010000005.1 | 99.625 | 801 | 3 | 0 | 69894 | 70694 |
| NZ_ALKU01000002.1 | 99.625 | 801 | 3 | 0 | 191279 | 192079 |
| JAVIHS010000007.1 | 99.625 | 801 | 3 | 0 | 7302 | 8102 |
| DASGVT010000012.1 | 99.625 | 801 | 3 | 0 | 30334 | 29534 |
| DASGVS010000013.1 | 99.625 | 801 | 3 | 0 | 30324 | 29524 |
| DASGUK010000006.1 | 99.625 | 801 | 3 | 0 | 34680 | 33880 |
| DASGUG010000006.1 | 99.625 | 801 | 3 | 0 | 34680 | 33880 |
| DASGUF010000005.1 | 99.625 | 801 | 3 | 0 | 34680 | 33880 |
| DASGSH010000006.1 | 99.625 | 801 | 3 | 0 | 34680 | 33880 |
| DASGCZ010000021.1 | 99.874 | 795 | 1 | 0 | 34585 | 33791 |
| DASFNS010000007.1 | 99.625 | 801 | 3 | 0 | 72 | 872 |
| DASFID010000017.1 | 99.625 | 801 | 3 | 0 | 29496 | 28696 |
| DASFIC010000073.1 | 99.625 | 801 | 3 | 0 | 11578 | 10778 |
| JAASBU010000048.1 | 99.625 | 801 | 3 | 0 | 60476 | 59676 |
| JABTYY010000001.1 | 99.625 | 801 | 3 | 0 | 12467 | 13267 |
| JABKSL010000041.1 | 99.625 | 801 | 3 | 0 | 12496 | 13296 |
| JABKWG010000005.1 | 99.625 | 801 | 3 | 0 | 69894 | 70694 |
| ALKU01000002.1 | 99.625 | 801 | 3 | 0 | 191279 | 192079 |
| VIFI01000001.1 | 99.625 | 801 | 0 | 3 | 1573875 | 1574672 |
| VIEQ01000001.1 | 99.625 | 801 | 0 | 3 | 1386980 | 1387777 |
| VIEO01000001.1 | 99.625 | 801 | 0 | 3 | 1572480 | 1573277 |
| NZ_FIIQ01000022.1 | 99.501 | 801 | 4 | 0 | 30237 | 29437 |
| NZ_FILR01000015.1 | 99.501 | 801 | 4 | 0 | 52661 | 51861 |
| NZ_FIJA01000016.1 | 99.501 | 801 | 4 | 0 | 30729 | 29929 |
| NZ_FILX01000029.1 | 99.501 | 801 | 4 | 0 | 13372 | 12572 |
| NZ_FILB01000011.1 | 99.501 | 801 | 4 | 0 | 1442 | 2242 |
| NZ_CZFI01000020.1 | 99.501 | 801 | 4 | 0 | 30246 | 29446 |
| NZ_JBAPDO010000003.1 | 99.501 | 801 | 4 | 0 | 1384 | 2184 |
| NZ_CP139877.1 | 99.501 | 801 | 4 | 0 | 1171874 | 1172674 |
| NZ_JAXKWI010000001.1 | 99.501 | 801 | 4 | 0 | 1842881 | 1842081 |
| NZ_JAWWZD010000015.1 | 99.501 | 801 | 4 | 0 | 31162 | 31962 |
| NZ_JAUTHO010000020.1 | 99.501 | 801 | 4 | 0 | 1675 | 2475 |
| NZ_JAASFA010000014.1 | 99.501 | 801 | 4 | 0 | 21730 | 20930 |
| NZ_JAGFQW010000016.1 | 99.501 | 801 | 4 | 0 | 14443 | 13643 |
| NZ_JAEVFS010000023.1 | 99.501 | 801 | 4 | 0 | 15798 | 14998 |
| NZ_AP023391.1 | 99.501 | 801 | 4 | 0 | 902904 | 902104 |
| NZ_JABLFJ010000014.1 | 99.501 | 801 | 4 | 0 | 1457 | 2257 |
| NZ_JABLFZ010000014.1 | 99.501 | 801 | 4 | 0 | 1543 | 2343 |
| NZ_JABLGF010000031.1 | 99.501 | 801 | 4 | 0 | 13529 | 14329 |
| NZ_JABLHJ010000017.1 | 99.501 | 801 | 4 | 0 | 21971 | 22771 |
| NZ_JABLLG010000002.1 | 99.501 | 801 | 4 | 0 | 270371 | 269571 |
| NZ_JABLLQ010000004.1 | 99.501 | 801 | 4 | 0 | 115274 | 114474 |
| NZ_JABLLR010000005.1 | 99.501 | 801 | 4 | 0 | 115274 | 114474 |
| NZ_JABLLY010000012.1 | 99.501 | 801 | 4 | 0 | 1552 | 2352 |
| NZ_JABLMV010000002.1 | 99.501 | 801 | 4 | 0 | 1552 | 2352 |
| NZ_JABLMX010000013.1 | 99.501 | 801 | 4 | 0 | 1552 | 2352 |
| NZ_JABLMY010000012.1 | 99.501 | 801 | 4 | 0 | 1552 | 2352 |
| NZ_JABLNJ010000014.1 | 99.501 | 801 | 4 | 0 | 1552 | 2352 |
| NZ_JABLNH010000004.1 | 99.501 | 801 | 4 | 0 | 115274 | 114474 |
| NZ_JABLNU010000012.1 | 99.501 | 801 | 4 | 0 | 1552 | 2352 |
| NZ_JABLNY010000013.1 | 99.501 | 801 | 4 | 0 | 1552 | 2352 |
| NZ_JABLOC010000013.1 | 99.501 | 801 | 4 | 0 | 1552 | 2352 |
| NZ_JABLOD010000013.1 | 99.501 | 801 | 4 | 0 | 1552 | 2352 |
| NZ_JABLMK010000013.1 | 99.501 | 801 | 4 | 0 | 1552 | 2352 |
| NZ_JABLMG010000013.1 | 99.501 | 801 | 4 | 0 | 1552 | 2352 |
| NZ_JABLMO010000012.1 | 99.501 | 801 | 4 | 0 | 1552 | 2352 |
| NZ_JABLNV010000013.1 | 99.501 | 801 | 4 | 0 | 66992 | 66192 |
| NZ_JABLPA010000001.1 | 99.501 | 801 | 4 | 0 | 267936 | 267136 |
| NZ_JABLMM010000013.1 | 99.501 | 801 | 4 | 0 | 1552 | 2352 |
| NZ_JABLPO010000004.1 | 99.501 | 801 | 4 | 0 | 115274 | 114474 |
| NZ_JABKSF010000017.1 | 99.501 | 801 | 4 | 0 | 30292 | 29492 |
| NZ_JABKSO010000018.1 | 99.501 | 801 | 4 | 0 | 30292 | 29492 |
| NZ_JABKTJ010000018.1 | 99.501 | 801 | 4 | 0 | 30299 | 29499 |
| NZ_JABKSD010000018.1 | 99.501 | 801 | 4 | 0 | 1507 | 2307 |
| NZ_JABKSP010000023.1 | 99.501 | 801 | 4 | 0 | 30292 | 29492 |
| NZ_JABKWO010000018.1 | 99.501 | 801 | 4 | 0 | 1503 | 2303 |
| NZ_JABKXT010000002.1 | 99.501 | 801 | 4 | 0 | 159168 | 159968 |
| NZ_JABLCV010000025.1 | 99.501 | 801 | 4 | 0 | 1455 | 2255 |
| NZ_JABLCZ010000023.1 | 99.501 | 801 | 4 | 0 | 1537 | 2337 |
| NZ_JABCRP010000003.1 | 99.501 | 801 | 4 | 0 | 152150 | 151350 |
| NZ_JABCRZ010000003.1 | 99.501 | 801 | 4 | 0 | 152150 | 151350 |
| NZ_CP017088.1 | 99.501 | 801 | 4 | 0 | 920910 | 920110 |
| NZ_CEHB01000006.1 | 99.501 | 801 | 4 | 0 | 13378 | 12578 |
| NZ_CEGZ01000016.1 | 99.501 | 801 | 4 | 0 | 1442 | 2242 |
| NZ_CELX01000032.1 | 99.501 | 801 | 4 | 0 | 30486 | 29686 |
| NZ_CEDV01000005.1 | 99.501 | 801 | 4 | 0 | 30231 | 29431 |
| NZ_CEII01000108.1 | 99.501 | 801 | 4 | 0 | 1440 | 2240 |
| FIIQ01000022.1 | 99.501 | 801 | 4 | 0 | 30237 | 29437 |
| FILR01000015.1 | 99.501 | 801 | 4 | 0 | 52661 | 51861 |
| FIJA01000016.1 | 99.501 | 801 | 4 | 0 | 30729 | 29929 |
| FILX01000029.1 | 99.501 | 801 | 4 | 0 | 13372 | 12572 |
| FILB01000011.1 | 99.501 | 801 | 4 | 0 | 1442 | 2242 |
| CZFI01000020.1 | 99.501 | 801 | 4 | 0 | 30246 | 29446 |
| JBAPDO010000003.1 | 99.501 | 801 | 4 | 0 | 1384 | 2184 |
| CP139877.1 | 99.501 | 801 | 4 | 0 | 1171874 | 1172674 |
| JAXKWI010000001.1 | 99.501 | 801 | 4 | 0 | 1842881 | 1842081 |
| DASGYG010000003.1 | 99.501 | 801 | 4 | 0 | 151593 | 150793 |
| DASGTK010000003.1 | 99.501 | 801 | 4 | 0 | 1383 | 2183 |
| DASGIF010000022.1 | 99.501 | 801 | 4 | 0 | 20906 | 20106 |
| DASGER010000014.1 | 99.501 | 801 | 4 | 0 | 67176 | 66376 |
| DASGEP010000015.1 | 99.501 | 801 | 4 | 0 | 67176 | 66376 |
| DASGEN010000013.1 | 99.501 | 801 | 4 | 0 | 67178 | 66378 |
| DASGEC010000014.1 | 99.501 | 801 | 4 | 0 | 67178 | 66378 |
| DASGDX010000013.1 | 99.501 | 801 | 4 | 0 | 1381 | 2181 |
| DASGDW010000014.1 | 99.501 | 801 | 4 | 0 | 1381 | 2181 |
| DASGDT010000013.1 | 99.501 | 801 | 4 | 0 | 1381 | 2181 |
| DASGDO010000013.1 | 99.501 | 801 | 4 | 0 | 1381 | 2181 |
| DASGDP010000013.1 | 99.501 | 801 | 4 | 0 | 1381 | 2181 |
| DASGAF010000001.1 | 99.501 | 801 | 4 | 0 | 221645 | 222445 |
| DASFZS010000019.1 | 99.501 | 801 | 4 | 0 | 30168 | 29368 |
| DASFZO010000001.1 | 99.501 | 801 | 4 | 0 | 67648 | 66848 |
| DASFXF010000001.1 | 99.501 | 801 | 4 | 0 | 47542 | 46742 |
| DASFOX010000005.1 | 99.501 | 801 | 4 | 0 | 8574 | 9374 |
| DASFMD010000001.1 | 99.501 | 801 | 4 | 0 | 109881 | 109081 |
| DASFHN010000024.1 | 99.501 | 801 | 4 | 0 | 30168 | 29368 |
| DASFFJ010000024.1 | 99.501 | 801 | 4 | 0 | 30168 | 29368 |
| DASFFK010000027.1 | 99.501 | 801 | 4 | 0 | 30168 | 29368 |
| DASFEX010000025.1 | 99.501 | 801 | 4 | 0 | 30175 | 29375 |
| DASFET010000027.1 | 99.501 | 801 | 4 | 0 | 30168 | 29368 |
| DASEMZ010000001.1 | 99.501 | 801 | 4 | 0 | 1383 | 2183 |
| DASEML010000001.1 | 99.501 | 801 | 4 | 0 | 1383 | 2183 |
| DASEMK010000001.1 | 99.501 | 801 | 4 | 0 | 1383 | 2183 |
| DASEHQ010000024.1 | 99.501 | 801 | 4 | 0 | 30168 | 29368 |
| DASEHP010000023.1 | 99.501 | 801 | 4 | 0 | 30168 | 29368 |
| DASEHJ010000002.1 | 99.501 | 801 | 4 | 0 | 1383 | 2183 |
| DASEHH010000002.1 | 99.501 | 801 | 4 | 0 | 1383 | 2183 |
| DASEHA010000003.1 | 99.501 | 801 | 4 | 0 | 1383 | 2183 |
| DASEGY010000021.1 | 99.501 | 801 | 4 | 0 | 30168 | 29368 |
| DASEGX010000001.1 | 99.501 | 801 | 4 | 0 | 1383 | 2183 |
| DASEGS010000002.1 | 99.501 | 801 | 4 | 0 | 31862 | 31062 |
| DASCLS010000001.1 | 99.501 | 801 | 4 | 0 | 207876 | 207076 |
| DARZUO010000005.1 | 99.501 | 801 | 4 | 0 | 34677 | 33877 |
| DARZUK010000002.1 | 99.501 | 801 | 4 | 0 | 93182 | 92382 |
| DARZPY010000001.1 | 99.501 | 801 | 4 | 0 | 289303 | 290103 |
| DARZOG010000012.1 | 99.501 | 801 | 4 | 0 | 1383 | 2183 |
| DARZMP010000001.1 | 99.501 | 801 | 4 | 0 | 157183 | 156383 |
| DARZMR010000001.1 | 99.501 | 801 | 4 | 0 | 115817 | 115017 |
| DARZGW010000003.1 | 99.501 | 801 | 4 | 0 | 1381 | 2181 |
| DARZGJ010000003.1 | 99.501 | 801 | 4 | 0 | 1383 | 2183 |
| DARZGF010000003.1 | 99.501 | 801 | 4 | 0 | 1381 | 2181 |
| DARZGH010000003.1 | 99.501 | 801 | 4 | 0 | 1381 | 2181 |
| DARZCG010000029.1 | 99.501 | 801 | 4 | 0 | 13483 | 12683 |
| DARZCA010000010.1 | 99.501 | 801 | 4 | 0 | 20118 | 19318 |
| DARYYO010000007.1 | 99.501 | 801 | 4 | 0 | 66059 | 65259 |
| JAWWZD010000015.1 | 99.501 | 801 | 4 | 0 | 31162 | 31962 |
| JAUTHO010000020.1 | 99.501 | 801 | 4 | 0 | 1675 | 2475 |
| JAASFA010000014.1 | 99.501 | 801 | 4 | 0 | 21730 | 20930 |
| JAGFQW010000016.1 | 99.501 | 801 | 4 | 0 | 14443 | 13643 |
| JAEVFS010000023.1 | 99.501 | 801 | 4 | 0 | 15798 | 14998 |
| AP023391.1 | 99.501 | 801 | 4 | 0 | 902904 | 902104 |
| JABLRZ010000002.1 | 99.501 | 801 | 4 | 0 | 269220 | 268420 |
| JABLFJ010000014.1 | 99.501 | 801 | 4 | 0 | 1457 | 2257 |
| JABLFZ010000014.1 | 99.501 | 801 | 4 | 0 | 1543 | 2343 |
| JABLGF010000031.1 | 99.501 | 801 | 4 | 0 | 13529 | 14329 |
| JABLHJ010000017.1 | 99.501 | 801 | 4 | 0 | 21971 | 22771 |
| JABLLG010000002.1 | 99.501 | 801 | 4 | 0 | 270371 | 269571 |
| JABLLQ010000004.1 | 99.501 | 801 | 4 | 0 | 115274 | 114474 |
| JABLLR010000005.1 | 99.501 | 801 | 4 | 0 | 115274 | 114474 |
| JABLLY010000012.1 | 99.501 | 801 | 4 | 0 | 1552 | 2352 |
| JABLMV010000002.1 | 99.501 | 801 | 4 | 0 | 1552 | 2352 |
| JABLMX010000013.1 | 99.501 | 801 | 4 | 0 | 1552 | 2352 |
| JABLMY010000012.1 | 99.501 | 801 | 4 | 0 | 1552 | 2352 |
| JABLNJ010000014.1 | 99.501 | 801 | 4 | 0 | 1552 | 2352 |
| JABLNH010000004.1 | 99.501 | 801 | 4 | 0 | 115274 | 114474 |
| JABLNU010000012.1 | 99.501 | 801 | 4 | 0 | 1552 | 2352 |
| JABLNY010000013.1 | 99.501 | 801 | 4 | 0 | 1552 | 2352 |
| JABLOC010000013.1 | 99.501 | 801 | 4 | 0 | 1552 | 2352 |
| JABLOD010000013.1 | 99.501 | 801 | 4 | 0 | 1552 | 2352 |
| JABLMK010000013.1 | 99.501 | 801 | 4 | 0 | 1552 | 2352 |
| JABLMG010000013.1 | 99.501 | 801 | 4 | 0 | 1552 | 2352 |
| JABLMO010000012.1 | 99.501 | 801 | 4 | 0 | 1552 | 2352 |
| JABLNV010000013.1 | 99.501 | 801 | 4 | 0 | 66992 | 66192 |
| JABLPA010000001.1 | 99.501 | 801 | 4 | 0 | 267936 | 267136 |
| JABLMM010000013.1 | 99.501 | 801 | 4 | 0 | 1552 | 2352 |
| JABLPO010000004.1 | 99.501 | 801 | 4 | 0 | 115274 | 114474 |
| JABKSF010000017.1 | 99.501 | 801 | 4 | 0 | 30292 | 29492 |
| JABKSO010000018.1 | 99.501 | 801 | 4 | 0 | 30292 | 29492 |
| JABKTJ010000018.1 | 99.501 | 801 | 4 | 0 | 30299 | 29499 |
| JABKSD010000018.1 | 99.501 | 801 | 4 | 0 | 1507 | 2307 |
| JABKSP010000023.1 | 99.501 | 801 | 4 | 0 | 30292 | 29492 |
| JABKWO010000018.1 | 99.501 | 801 | 4 | 0 | 1503 | 2303 |
| JABKXT010000002.1 | 99.501 | 801 | 4 | 0 | 159168 | 159968 |
| JABLCV010000025.1 | 99.501 | 801 | 4 | 0 | 1455 | 2255 |
| JABLCZ010000023.1 | 99.501 | 801 | 4 | 0 | 1537 | 2337 |
| JABCRP010000003.1 | 99.501 | 801 | 4 | 0 | 152150 | 151350 |
| JABCRZ010000003.1 | 99.501 | 801 | 4 | 0 | 152150 | 151350 |
| CP017088.1 | 99.501 | 801 | 4 | 0 | 920910 | 920110 |
| CEHB01000006.1 | 99.501 | 801 | 4 | 0 | 13378 | 12578 |
| CEGZ01000016.1 | 99.501 | 801 | 4 | 0 | 1442 | 2242 |
| CELX01000032.1 | 99.501 | 801 | 4 | 0 | 30486 | 29686 |
| CEDV01000005.1 | 99.501 | 801 | 4 | 0 | 30231 | 29431 |
| CEII01000108.1 | 99.501 | 801 | 4 | 0 | 1440 | 2240 |
| NZ_JAASBX010000011.1 | 99.376 | 801 | 5 | 0 | 5630 | 6430 |
| DASFFW010000020.1 | 100 | 787 | 0 | 0 | 30075 | 29289 |
| JAASBX010000011.1 | 99.376 | 801 | 5 | 0 | 5630 | 6430 |
| NZ_VZNS01000009.1 | 99.376 | 801 | 5 | 0 | 13592 | 12792 |
| NZ_JABKYI010000003.1 | 99.376 | 801 | 5 | 0 | 80960 | 81760 |
| NZ_JABKYJ010000003.1 | 99.376 | 801 | 5 | 0 | 80964 | 81764 |
| NZ_JABKYN010000002.1 | 99.376 | 801 | 5 | 0 | 134511 | 135311 |
| NZ_VIEZ01000001.1 | 99.376 | 801 | 5 | 0 | 125345 | 126145 |
| NZ_VIEF01000005.1 | 99.376 | 801 | 5 | 0 | 127556 | 128356 |
| NZ_ASCF01000044.1 | 99.376 | 801 | 5 | 0 | 3216 | 2416 |
| NZ_ASAP01000002.1 | 99.376 | 801 | 5 | 0 | 3216 | 2416 |
| NZ_ALNF01000044.1 | 99.376 | 801 | 5 | 0 | 34496 | 33696 |
| DATUBF010000008.1 | 99.376 | 801 | 5 | 0 | 20869 | 20069 |
| DASGRS010000004.1 | 99.376 | 801 | 5 | 0 | 36710 | 37510 |
| DASGOG010000002.1 | 99.376 | 801 | 5 | 0 | 95427 | 96227 |
| DASGNZ010000003.1 | 99.376 | 801 | 5 | 0 | 78982 | 78182 |
| DASGNS010000003.1 | 99.376 | 801 | 5 | 0 | 33770 | 34570 |
| DASGBO010000007.1 | 99.376 | 801 | 5 | 0 | 59839 | 59039 |
| DASFYI010000037.1 | 99.623 | 795 | 3 | 0 | 20884 | 20090 |
| DASFVN010000022.1 | 99.623 | 795 | 3 | 0 | 34586 | 33792 |
| DASFTB010000032.1 | 99.376 | 801 | 5 | 0 | 10596 | 9796 |
| DASFQZ010000010.1 | 99.376 | 801 | 5 | 0 | 40346 | 41146 |
| DASFNL010000038.1 | 99.376 | 801 | 5 | 0 | 15833 | 15033 |
| DASEVG010000008.1 | 99.376 | 801 | 5 | 0 | 13800 | 14600 |
| DASEVF010000004.1 | 99.376 | 801 | 5 | 0 | 55174 | 55974 |
| DASEGD010000045.1 | 99.376 | 801 | 5 | 0 | 2645 | 1845 |
| DASEGC010000088.1 | 99.376 | 801 | 5 | 0 | 2455 | 3255 |
| DASEGB010000090.1 | 99.376 | 801 | 5 | 0 | 3717 | 4517 |
| DASEFO010000002.1 | 99.376 | 801 | 5 | 0 | 115413 | 114613 |
| DARZMV010000002.1 | 99.376 | 801 | 5 | 0 | 242768 | 241968 |
| DARZLB010000001.1 | 99.376 | 801 | 5 | 0 | 14300 | 15100 |
| DARZGA010000001.1 | 99.376 | 801 | 5 | 0 | 107442 | 108242 |
| DARZFB010000019.1 | 99.376 | 801 | 5 | 0 | 2805 | 2005 |
| DARZCB010000001.1 | 99.376 | 801 | 5 | 0 | 60858 | 60058 |
| DARYZX010000016.1 | 99.376 | 801 | 5 | 0 | 54289 | 53489 |
| DARYZQ010000028.1 | 99.376 | 801 | 5 | 0 | 14267 | 13467 |
| DARYZS010000008.1 | 99.376 | 801 | 5 | 0 | 64877 | 64077 |
| DARYYJ010000041.1 | 99.376 | 801 | 5 | 0 | 2759 | 1959 |
| VZNS01000009.1 | 99.376 | 801 | 5 | 0 | 13592 | 12792 |
| JABLRV010000014.1 | 99.376 | 801 | 5 | 0 | 1662 | 2462 |
| JABLRW010000016.1 | 99.376 | 801 | 5 | 0 | 1662 | 2462 |
| JABLRX010000016.1 | 99.376 | 801 | 5 | 0 | 1662 | 2462 |
| JABKYI010000003.1 | 99.376 | 801 | 5 | 0 | 80960 | 81760 |
| JABKYJ010000003.1 | 99.376 | 801 | 5 | 0 | 80964 | 81764 |
| JABKYN010000002.1 | 99.376 | 801 | 5 | 0 | 134511 | 135311 |
| VIEZ01000001.1 | 99.376 | 801 | 5 | 0 | 125345 | 126145 |
| VIEF01000005.1 | 99.376 | 801 | 5 | 0 | 127556 | 128356 |
| ASCF01000044.1 | 99.376 | 801 | 5 | 0 | 3216 | 2416 |
| ASAP01000002.1 | 99.376 | 801 | 5 | 0 | 3216 | 2416 |
| ALNF01000044.1 | 99.376 | 801 | 5 | 0 | 34496 | 33696 |
| NZ_FIGT01000008.1 | 99.251 | 801 | 6 | 0 | 13424 | 12624 |
| NZ_FIGN01000004.1 | 99.251 | 801 | 6 | 0 | 108853 | 109653 |
| NZ_FILL01000002.1 | 99.251 | 801 | 6 | 0 | 47718 | 48518 |
| NZ_FIHE01000005.1 | 99.251 | 801 | 6 | 0 | 110353 | 111153 |
| NZ_FIIP01000013.1 | 99.251 | 801 | 6 | 0 | 30929 | 31729 |
| NZ_FIHL01000031.1 | 99.251 | 801 | 6 | 0 | 12512 | 13312 |
| NZ_FIGQ01000007.1 | 99.251 | 801 | 6 | 0 | 55439 | 54639 |
| NZ_FIGO01000001.1 | 99.251 | 801 | 6 | 0 | 42056 | 41256 |
| NZ_CZFC01000073.1 | 99.251 | 801 | 6 | 0 | 166284 | 165484 |
| NZ_JBAPDH010000003.1 | 99.251 | 801 | 6 | 0 | 173981 | 173181 |
| NZ_JAVIGU010000015.1 | 99.251 | 801 | 6 | 0 | 10254 | 11054 |
| NZ_JAVIHE010000016.1 | 99.251 | 801 | 6 | 0 | 32052 | 32852 |
| NZ_JAVIHH010000003.1 | 99.251 | 801 | 6 | 0 | 106098 | 106898 |
| NZ_JAVIHX010000007.1 | 99.251 | 801 | 6 | 0 | 11507 | 10707 |
| NZ_JAVIIA010000007.1 | 99.251 | 801 | 6 | 0 | 10295 | 9495 |
| NZ_JAVIIB010000001.1 | 99.251 | 801 | 6 | 0 | 91014 | 90214 |
| NZ_CP140109.1 | 99.251 | 801 | 6 | 0 | 1122357 | 1121557 |
| NZ_JAXKWN010000002.1 | 99.251 | 801 | 6 | 0 | 144145 | 144945 |
| NZ_JAUTHT010000004.1 | 99.251 | 801 | 6 | 0 | 20906 | 20106 |
| NZ_JAUTHK010000008.1 | 99.251 | 801 | 6 | 0 | 73911 | 74711 |
| NZ_JAUTHC010000008.1 | 99.251 | 801 | 6 | 0 | 47443 | 48243 |
| NZ_JAUTHB010000001.1 | 99.251 | 801 | 6 | 0 | 240176 | 240976 |
| NZ_CP134469.1 | 99.251 | 801 | 6 | 0 | 1158512 | 1157712 |
| NZ_JANFMC010000011.1 | 99.251 | 801 | 6 | 0 | 41485 | 42285 |
| NZ_JANUHD010000001.1 | 99.251 | 801 | 6 | 0 | 1774042 | 1773242 |
| NZ_JANIFM010000006.1 | 99.251 | 801 | 6 | 0 | 13630 | 12830 |
| NZ_JAASEE010000001.1 | 99.251 | 801 | 6 | 0 | 17662 | 18462 |
| NZ_JAFHCX010000006.1 | 99.251 | 801 | 6 | 0 | 52243 | 51443 |
| NZ_JAFHCW010000006.1 | 99.251 | 801 | 6 | 0 | 57140 | 57940 |
| NZ_JAIMFB010000001.1 | 99.251 | 801 | 6 | 0 | 168586 | 169386 |
| NZ_JAFFHS010000020.1 | 99.251 | 801 | 6 | 0 | 18379 | 19179 |
| NZ_JAFFHQ010000011.1 | 99.251 | 801 | 6 | 0 | 41485 | 42285 |
| NZ_CP065430.1 | 99.251 | 801 | 6 | 0 | 1149459 | 1148659 |
| NZ_JABTZO010000015.1 | 99.251 | 801 | 6 | 0 | 52016 | 51216 |
| NZ_JABTZA010000014.1 | 99.251 | 801 | 6 | 0 | 13463 | 12663 |
| NZ_JABTYZ010000012.1 | 99.251 | 801 | 6 | 0 | 13463 | 12663 |
| NZ_JABTYV010000002.1 | 99.251 | 801 | 6 | 0 | 60379 | 59579 |
| NZ_JABLFT010000001.1 | 99.251 | 801 | 6 | 0 | 235364 | 236164 |
| NZ_JABLHG010000034.1 | 99.251 | 801 | 6 | 0 | 12586 | 13386 |
| NZ_JABLHF010000007.1 | 99.251 | 801 | 6 | 0 | 31171 | 31971 |
| NZ_JABLJF010000010.1 | 99.251 | 801 | 6 | 0 | 33936 | 34736 |
| NZ_JABLLN010000002.1 | 99.251 | 801 | 6 | 0 | 206276 | 207076 |
| NZ_JABLLJ010000011.1 | 99.251 | 801 | 6 | 0 | 53388 | 54188 |
| NZ_JABLLK010000007.1 | 99.251 | 801 | 6 | 0 | 55541 | 54741 |
| NZ_JABLLS010000002.1 | 99.251 | 801 | 6 | 0 | 206276 | 207076 |
| NZ_JABLNB010000013.1 | 99.251 | 801 | 6 | 0 | 47125 | 47925 |
| NZ_JABLNO010000003.1 | 99.251 | 801 | 6 | 0 | 45715 | 46515 |
| NZ_JABLLF010000007.1 | 99.251 | 801 | 6 | 0 | 60380 | 59580 |
| NZ_JABLNS010000002.1 | 99.251 | 801 | 6 | 0 | 153100 | 152300 |
| NZ_JABKRR010000005.1 | 99.251 | 801 | 6 | 0 | 6742 | 7542 |
| NZ_JABKSI010000001.1 | 99.251 | 801 | 6 | 0 | 336905 | 336105 |
| NZ_JABKSM010000011.1 | 99.251 | 801 | 6 | 0 | 11065 | 10265 |
| NZ_JABKUJ010000019.1 | 99.251 | 801 | 6 | 0 | 1505 | 2305 |
| NZ_JABKTH010000003.1 | 99.251 | 801 | 6 | 0 | 31530 | 32330 |
| NZ_JABKZJ010000018.1 | 99.251 | 801 | 6 | 0 | 12479 | 13279 |
| NZ_JABKZI010000016.1 | 99.251 | 801 | 6 | 0 | 12477 | 13277 |
| NZ_JABKZH010000017.1 | 99.251 | 801 | 6 | 0 | 33438 | 32638 |
| NZ_JABLAW010000001.1 | 99.251 | 801 | 6 | 0 | 318107 | 318907 |
| NZ_JABLAZ010000031.1 | 99.251 | 801 | 6 | 0 | 13437 | 12637 |
| NZ_JABLBD010000002.1 | 99.251 | 801 | 6 | 0 | 174653 | 175453 |
| NZ_JABLBJ010000031.1 | 99.251 | 801 | 6 | 0 | 12530 | 13330 |
| NZ_JABLBO010000006.1 | 99.251 | 801 | 6 | 0 | 110365 | 111165 |
| NZ_JABLCW010000002.1 | 99.251 | 801 | 6 | 0 | 105219 | 106019 |
| NZ_JABCRM010000002.1 | 99.251 | 801 | 6 | 0 | 60379 | 59579 |
| NZ_JABCSB010000001.1 | 99.251 | 801 | 6 | 0 | 60379 | 59579 |
| NZ_JABCRY010000002.1 | 99.251 | 801 | 6 | 0 | 60379 | 59579 |
| NZ_JABCRX010000004.1 | 99.251 | 801 | 6 | 0 | 60379 | 59579 |
| NZ_RRZO01000052.1 | 99.251 | 801 | 6 | 0 | 13211 | 12411 |
| NZ_POPI01000479.1 | 99.251 | 801 | 6 | 0 | 1508 | 2308 |
| NZ_POPJ01000022.1 | 99.251 | 801 | 6 | 0 | 1508 | 2308 |
| NZ_POPN01000218.1 | 99.251 | 801 | 6 | 0 | 58238 | 59038 |
| NZ_POPC01000413.1 | 99.251 | 801 | 6 | 0 | 31607 | 30807 |
| NZ_POOO01000035.1 | 99.251 | 801 | 6 | 0 | 32070 | 32870 |
| NZ_POLH01000125.1 | 99.251 | 801 | 6 | 0 | 31711 | 30911 |
| NZ_POLF01000076.1 | 99.251 | 801 | 6 | 0 | 33239 | 34039 |
| NZ_POLE01000040.1 | 99.251 | 801 | 6 | 0 | 33239 | 34039 |
| NZ_POKW01000058.1 | 99.251 | 801 | 6 | 0 | 150325 | 149525 |
| NZ_POLD01000054.1 | 99.251 | 801 | 6 | 0 | 33302 | 34102 |
| NZ_POJT01000031.1 | 99.251 | 801 | 6 | 0 | 64185 | 63385 |
| NZ_POJO01000048.1 | 99.251 | 801 | 6 | 0 | 31576 | 32376 |
| NZ_POJR01000094.1 | 99.251 | 801 | 6 | 0 | 10231 | 9431 |
| NZ_POOM01000086.1 | 99.251 | 801 | 6 | 0 | 149873 | 150673 |
| NZ_POOI01000134.1 | 99.251 | 801 | 6 | 0 | 30130 | 29330 |
| NZ_PONN01000034.1 | 99.251 | 801 | 6 | 0 | 149873 | 150673 |
| NZ_PONM01000031.1 | 99.251 | 801 | 6 | 0 | 149873 | 150673 |
| NZ_POLK01000051.1 | 99.251 | 801 | 6 | 0 | 150325 | 149525 |
| NZ_POLI01000047.1 | 99.251 | 801 | 6 | 0 | 33479 | 34279 |
| NZ_POKX01000058.1 | 99.251 | 801 | 6 | 0 | 31640 | 30840 |
| NZ_POKV01000047.1 | 99.251 | 801 | 6 | 0 | 4333 | 5133 |
| NZ_POKP01000056.1 | 99.251 | 801 | 6 | 0 | 10174 | 9374 |
| NZ_POKO01000216.1 | 99.251 | 801 | 6 | 0 | 70940 | 70140 |
| NZ_POJV01000710.1 | 99.251 | 801 | 6 | 0 | 31695 | 32495 |
| NZ_POJS01000033.1 | 99.251 | 801 | 6 | 0 | 15774 | 14974 |
| NZ_POJQ01000082.1 | 99.251 | 801 | 6 | 0 | 15774 | 14974 |
| NZ_POJP01000036.1 | 99.251 | 801 | 6 | 0 | 64214 | 63414 |
| NZ_POIV01000064.1 | 99.251 | 801 | 6 | 0 | 1507 | 2307 |
| NZ_POID01000093.1 | 99.251 | 801 | 6 | 0 | 44718 | 43918 |
| NZ_ASCA01000056.1 | 99.251 | 801 | 6 | 0 | 89511 | 88711 |
| NZ_CEKE01000067.1 | 99.251 | 801 | 6 | 0 | 47860 | 48660 |
| NZ_CEJS01000028.1 | 99.251 | 801 | 6 | 0 | 53345 | 54145 |
| NZ_CEHU01000023.1 | 99.251 | 801 | 6 | 0 | 42054 | 41254 |
| NZ_CEJK01000007.1 | 99.251 | 801 | 6 | 0 | 110350 | 111150 |
| NZ_CEJP01000015.1 | 99.251 | 801 | 6 | 0 | 30241 | 29441 |
| NZ_CEGO01000047.1 | 99.251 | 801 | 6 | 0 | 108538 | 109338 |
| NZ_CEEX01000072.1 | 99.251 | 801 | 6 | 0 | 12503 | 13303 |
| NZ_CEDT01000290.1 | 99.251 | 801 | 6 | 0 | 33871 | 34671 |
| NZ_ALMJ01000016.1 | 99.251 | 801 | 6 | 0 | 30252 | 29452 |
| NZ_ALLK01000017.1 | 99.251 | 801 | 6 | 0 | 25877 | 26677 |
| NZ_ALLC01000055.1 | 99.251 | 801 | 6 | 0 | 12116 | 12916 |
| FIGT01000008.1 | 99.251 | 801 | 6 | 0 | 13424 | 12624 |
| FIGN01000004.1 | 99.251 | 801 | 6 | 0 | 108853 | 109653 |
| FILL01000002.1 | 99.251 | 801 | 6 | 0 | 47718 | 48518 |
| FIHE01000005.1 | 99.251 | 801 | 6 | 0 | 110353 | 111153 |
| FIIP01000013.1 | 99.251 | 801 | 6 | 0 | 30929 | 31729 |
| FIHL01000031.1 | 99.251 | 801 | 6 | 0 | 12512 | 13312 |
| FIGQ01000007.1 | 99.251 | 801 | 6 | 0 | 55439 | 54639 |
| FIGO01000001.1 | 99.251 | 801 | 6 | 0 | 42056 | 41256 |
| CZFC01000073.1 | 99.251 | 801 | 6 | 0 | 166284 | 165484 |
| JBAPDH010000003.1 | 99.251 | 801 | 6 | 0 | 173981 | 173181 |
| JAVIGU010000015.1 | 99.251 | 801 | 6 | 0 | 10254 | 11054 |
| JAVIHE010000016.1 | 99.251 | 801 | 6 | 0 | 32052 | 32852 |
| JAVIHH010000003.1 | 99.251 | 801 | 6 | 0 | 106098 | 106898 |
| JAVIHX010000007.1 | 99.251 | 801 | 6 | 0 | 11507 | 10707 |
| JAVIIA010000007.1 | 99.251 | 801 | 6 | 0 | 10295 | 9495 |
| JAVIIB010000001.1 | 99.251 | 801 | 6 | 0 | 91014 | 90214 |
| DATUBY010000008.1 | 99.251 | 801 | 6 | 0 | 46955 | 47755 |
| DATUBZ010000009.1 | 99.251 | 801 | 6 | 0 | 47167 | 47967 |
| DATUBP010000001.1 | 99.251 | 801 | 6 | 0 | 251952 | 251152 |
| DATUBK010000002.1 | 99.251 | 801 | 6 | 0 | 31164 | 30364 |
| CP140109.1 | 99.251 | 801 | 6 | 0 | 1122357 | 1121557 |
| JAXKWN010000002.1 | 99.251 | 801 | 6 | 0 | 144145 | 144945 |
| DASGVK010000048.1 | 99.251 | 801 | 6 | 0 | 3359 | 4159 |
| DASGSL010000006.1 | 99.251 | 801 | 6 | 0 | 60603 | 59803 |
| DASGQG010000004.1 | 99.251 | 801 | 6 | 0 | 60689 | 59889 |
| DASGIJ010000005.1 | 99.251 | 801 | 6 | 0 | 31051 | 31851 |
| DASGIH010000034.1 | 99.251 | 801 | 6 | 0 | 13363 | 12563 |
| DASGGJ010000001.1 | 99.251 | 801 | 6 | 0 | 30169 | 29369 |
| DASGBK010000007.1 | 99.251 | 801 | 6 | 0 | 65369 | 64569 |
| DASGBF010000001.1 | 99.251 | 801 | 6 | 0 | 252855 | 253655 |
| DASGBE010000003.1 | 99.251 | 801 | 6 | 0 | 113210 | 112410 |
| DASGAZ010000002.1 | 99.251 | 801 | 6 | 0 | 34116 | 34916 |
| DASFZB010000009.1 | 99.251 | 801 | 6 | 0 | 34364 | 33564 |
| DASFUY010000016.1 | 99.251 | 801 | 6 | 0 | 29584 | 28784 |
| DASFUI010000017.1 | 99.251 | 801 | 6 | 0 | 31706 | 30906 |
| DASFUF010000007.1 | 99.251 | 801 | 6 | 0 | 30224 | 29424 |
| DASFTS010000032.1 | 99.251 | 801 | 6 | 0 | 20880 | 20080 |
| DASFTQ010000025.1 | 99.251 | 801 | 6 | 0 | 20875 | 20075 |
| DASFTM010000017.1 | 99.251 | 801 | 6 | 0 | 20869 | 20069 |
| DASFRZ010000018.1 | 99.251 | 801 | 6 | 0 | 20814 | 20014 |
| DASFQS010000012.1 | 99.251 | 801 | 6 | 0 | 28660 | 27860 |
| DASFIG010000031.1 | 99.251 | 801 | 6 | 0 | 10941 | 10141 |
| DASFHR010000001.1 | 99.251 | 801 | 6 | 0 | 160925 | 160125 |
| DASFEW010000025.1 | 99.251 | 801 | 6 | 0 | 30168 | 29368 |
| DASFEV010000003.1 | 99.251 | 801 | 6 | 0 | 122669 | 121869 |
| DASEVI010000026.1 | 99.251 | 801 | 6 | 0 | 10410 | 9610 |
| DASEOR010000007.1 | 99.251 | 801 | 6 | 0 | 30177 | 29377 |
| DASEOD010000012.1 | 99.251 | 801 | 6 | 0 | 30168 | 29368 |
| DASEHN010000002.1 | 99.251 | 801 | 6 | 0 | 227882 | 227082 |
| DASEHB010000030.1 | 99.251 | 801 | 6 | 0 | 3203 | 2403 |
| DASEGP010000015.1 | 99.251 | 801 | 6 | 0 | 20108 | 19308 |
| DASEGM010000002.1 | 99.251 | 801 | 6 | 0 | 220262 | 219462 |
| DASEGA010000024.1 | 99.251 | 801 | 6 | 0 | 30445 | 29645 |
| DASECP010000001.1 | 99.251 | 801 | 6 | 0 | 109210 | 110010 |
| DASCMC010000026.1 | 99.251 | 801 | 6 | 0 | 20392 | 19592 |
| DASCLO010000025.1 | 99.251 | 801 | 6 | 0 | 20396 | 19596 |
| DASCLM010000008.1 | 99.251 | 801 | 6 | 0 | 47319 | 48119 |
| DARZUM010000057.1 | 99.251 | 801 | 6 | 0 | 13463 | 12663 |
| DARZTN010000012.1 | 99.251 | 801 | 6 | 0 | 61155 | 60355 |
| DARZRX010000005.1 | 99.251 | 801 | 6 | 0 | 47357 | 48157 |
| DARZOV010000010.1 | 99.251 | 801 | 6 | 0 | 47323 | 48123 |
| DARZON010000001.1 | 99.251 | 801 | 6 | 0 | 311529 | 310729 |
| DARZOF010000030.1 | 99.251 | 801 | 6 | 0 | 13810 | 13010 |
| DARZOD010000024.1 | 99.251 | 801 | 6 | 0 | 21158 | 20358 |
| DARZNS010000002.1 | 99.251 | 801 | 6 | 0 | 210462 | 209662 |
| DARZLN010000007.1 | 99.251 | 801 | 6 | 0 | 47170 | 47970 |
| DARZLJ010000015.1 | 99.251 | 801 | 6 | 0 | 46767 | 45967 |
| DARZLL010000074.1 | 99.251 | 801 | 6 | 0 | 6018 | 5218 |
| DARZLH010000004.1 | 99.251 | 801 | 6 | 0 | 47321 | 48121 |
| DARZLG010000012.1 | 99.251 | 801 | 6 | 0 | 47240 | 48040 |
| DARZLE010000004.1 | 99.251 | 801 | 6 | 0 | 66285 | 65485 |
| DARZKU010000003.1 | 99.251 | 801 | 6 | 0 | 47174 | 47974 |
| DARZKP010000011.1 | 99.251 | 801 | 6 | 0 | 47167 | 47967 |
| DARZKQ010000010.1 | 99.251 | 801 | 6 | 0 | 47167 | 47967 |
| DARZKL010000012.1 | 99.251 | 801 | 6 | 0 | 47176 | 47976 |
| DARZKG010000011.1 | 99.251 | 801 | 6 | 0 | 47937 | 48737 |
| DARZKH010000012.1 | 99.251 | 801 | 6 | 0 | 47327 | 48127 |
| DARZKD010000012.1 | 99.251 | 801 | 6 | 0 | 47024 | 47824 |
| DARZKA010000012.1 | 99.251 | 801 | 6 | 0 | 47024 | 47824 |
| DARZHY010000009.1 | 99.251 | 801 | 6 | 0 | 75398 | 76198 |
| DARZHU010000001.1 | 99.251 | 801 | 6 | 0 | 289202 | 290002 |
| DARZHT010000001.1 | 99.251 | 801 | 6 | 0 | 175904 | 175104 |
| DARZHI010000002.1 | 99.251 | 801 | 6 | 0 | 47776 | 48576 |
| DARZHC010000012.1 | 99.251 | 801 | 6 | 0 | 47338 | 48138 |
| DARZGZ010000017.1 | 99.251 | 801 | 6 | 0 | 35009 | 35809 |
| DARZGX010000005.1 | 99.251 | 801 | 6 | 0 | 47337 | 48137 |
| DARZGV010000009.1 | 99.251 | 801 | 6 | 0 | 66285 | 65485 |
| DARZGU010000026.1 | 99.251 | 801 | 6 | 0 | 20345 | 19545 |
| DARZGS010000017.1 | 99.251 | 801 | 6 | 0 | 34543 | 35343 |
| DARZGL010000006.1 | 99.251 | 801 | 6 | 0 | 47340 | 48140 |
| DARZGK010000005.1 | 99.251 | 801 | 6 | 0 | 58687 | 59487 |
| DARZDS010000001.1 | 99.251 | 801 | 6 | 0 | 47979 | 48779 |
| DARZDJ010000016.1 | 99.251 | 801 | 6 | 0 | 33324 | 34124 |
| DARZDD010000009.1 | 99.251 | 801 | 6 | 0 | 47336 | 48136 |
| DARZCZ010000012.1 | 99.251 | 801 | 6 | 0 | 30172 | 29372 |
| DARZCV010000012.1 | 99.251 | 801 | 6 | 0 | 46845 | 47645 |
| DARZCX010000006.1 | 99.251 | 801 | 6 | 0 | 46845 | 47645 |
| DARZCU010000012.1 | 99.251 | 801 | 6 | 0 | 47320 | 48120 |
| DARZCR010000017.1 | 99.251 | 801 | 6 | 0 | 47044 | 47844 |
| DARZCK010000012.1 | 99.251 | 801 | 6 | 0 | 47231 | 48031 |
| DARZBZ010000014.1 | 99.251 | 801 | 6 | 0 | 47320 | 48120 |
| DARZBX010000010.1 | 99.251 | 801 | 6 | 0 | 47167 | 47967 |
| DARZBS010000003.1 | 99.251 | 801 | 6 | 0 | 47166 | 47966 |
| DARZBC010000074.1 | 99.251 | 801 | 6 | 0 | 919 | 119 |
| DARZAI010000008.1 | 99.251 | 801 | 6 | 0 | 45567 | 46367 |
| DARZAH010000013.1 | 99.251 | 801 | 6 | 0 | 34241 | 33441 |
| DARZAJ010000025.1 | 99.251 | 801 | 6 | 0 | 34769 | 33969 |
| DARZAA010000004.1 | 99.251 | 801 | 6 | 0 | 47327 | 48127 |
| DARYZM010000008.1 | 99.251 | 801 | 6 | 0 | 17840 | 18640 |
| DARYZE010000003.1 | 99.251 | 801 | 6 | 0 | 47337 | 48137 |
| DARYZA010000003.1 | 99.251 | 801 | 6 | 0 | 34313 | 35113 |
| DARYZB010000008.1 | 99.251 | 801 | 6 | 0 | 47119 | 47919 |
| JAUTHT010000004.1 | 99.251 | 801 | 6 | 0 | 20906 | 20106 |
| JAUTHK010000008.1 | 99.251 | 801 | 6 | 0 | 73911 | 74711 |
| JAUTHC010000008.1 | 99.251 | 801 | 6 | 0 | 47443 | 48243 |
| JAUTHB010000001.1 | 99.251 | 801 | 6 | 0 | 240176 | 240976 |
| CP134469.1 | 99.251 | 801 | 6 | 0 | 1158512 | 1157712 |
| JANFMC010000011.1 | 99.251 | 801 | 6 | 0 | 41485 | 42285 |
| JANUHD010000001.1 | 99.251 | 801 | 6 | 0 | 1774042 | 1773242 |
| JANIFM010000006.1 | 99.251 | 801 | 6 | 0 | 13630 | 12830 |
| JAASEE010000001.1 | 99.251 | 801 | 6 | 0 | 17662 | 18462 |
| JAFHCX010000006.1 | 99.251 | 801 | 6 | 0 | 52243 | 51443 |
| JAFHCW010000006.1 | 99.251 | 801 | 6 | 0 | 57140 | 57940 |
| JAIMFB010000001.1 | 99.251 | 801 | 6 | 0 | 168586 | 169386 |
| JAFFHS010000020.1 | 99.251 | 801 | 6 | 0 | 18379 | 19179 |
| JAFFHQ010000011.1 | 99.251 | 801 | 6 | 0 | 41485 | 42285 |
| CP065430.1 | 99.251 | 801 | 6 | 0 | 1149459 | 1148659 |
| JABTZO010000015.1 | 99.251 | 801 | 6 | 0 | 52016 | 51216 |
| JABTZA010000014.1 | 99.251 | 801 | 6 | 0 | 13463 | 12663 |
| JABTYZ010000012.1 | 99.251 | 801 | 6 | 0 | 13463 | 12663 |
| JABTYV010000002.1 | 99.251 | 801 | 6 | 0 | 60379 | 59579 |
| JABLSA010000011.1 | 99.251 | 801 | 6 | 0 | 47913 | 48713 |
| JABLFT010000001.1 | 99.251 | 801 | 6 | 0 | 235364 | 236164 |
| JABLHG010000034.1 | 99.251 | 801 | 6 | 0 | 12586 | 13386 |
| JABLHF010000007.1 | 99.251 | 801 | 6 | 0 | 31171 | 31971 |
| JABLJF010000010.1 | 99.251 | 801 | 6 | 0 | 33936 | 34736 |
| JABLLN010000002.1 | 99.251 | 801 | 6 | 0 | 206276 | 207076 |
| JABLLJ010000011.1 | 99.251 | 801 | 6 | 0 | 53388 | 54188 |
| JABLLK010000007.1 | 99.251 | 801 | 6 | 0 | 55541 | 54741 |
| JABLLS010000002.1 | 99.251 | 801 | 6 | 0 | 206276 | 207076 |
| JABLNB010000013.1 | 99.251 | 801 | 6 | 0 | 47125 | 47925 |
| JABLNO010000003.1 | 99.251 | 801 | 6 | 0 | 45715 | 46515 |
| JABLLF010000007.1 | 99.251 | 801 | 6 | 0 | 60380 | 59580 |
| JABLNS010000002.1 | 99.251 | 801 | 6 | 0 | 153100 | 152300 |
| JABKRR010000005.1 | 99.251 | 801 | 6 | 0 | 6742 | 7542 |
| JABKSI010000001.1 | 99.251 | 801 | 6 | 0 | 336905 | 336105 |
| JABKSM010000011.1 | 99.251 | 801 | 6 | 0 | 11065 | 10265 |
| JABKUJ010000019.1 | 99.251 | 801 | 6 | 0 | 1505 | 2305 |
| JABKTH010000003.1 | 99.251 | 801 | 6 | 0 | 31530 | 32330 |
| JABKZJ010000018.1 | 99.251 | 801 | 6 | 0 | 12479 | 13279 |
| JABKZI010000016.1 | 99.251 | 801 | 6 | 0 | 12477 | 13277 |
| JABKZH010000017.1 | 99.251 | 801 | 6 | 0 | 33438 | 32638 |
| JABLAW010000001.1 | 99.251 | 801 | 6 | 0 | 318107 | 318907 |
| JABLAZ010000031.1 | 99.251 | 801 | 6 | 0 | 13437 | 12637 |
| JABLBD010000002.1 | 99.251 | 801 | 6 | 0 | 174653 | 175453 |
| JABLBJ010000031.1 | 99.251 | 801 | 6 | 0 | 12530 | 13330 |
| JABLBO010000006.1 | 99.251 | 801 | 6 | 0 | 110365 | 111165 |
| JABLCW010000002.1 | 99.251 | 801 | 6 | 0 | 105219 | 106019 |
| JABCRM010000002.1 | 99.251 | 801 | 6 | 0 | 60379 | 59579 |
| JABCSB010000001.1 | 99.251 | 801 | 6 | 0 | 60379 | 59579 |
| JABCRY010000002.1 | 99.251 | 801 | 6 | 0 | 60379 | 59579 |
| JABCRX010000004.1 | 99.251 | 801 | 6 | 0 | 60379 | 59579 |
| RRZO01000052.1 | 99.251 | 801 | 6 | 0 | 13211 | 12411 |
| POPI01000479.1 | 99.251 | 801 | 6 | 0 | 1508 | 2308 |
| POPJ01000022.1 | 99.251 | 801 | 6 | 0 | 1508 | 2308 |
| POPN01000218.1 | 99.251 | 801 | 6 | 0 | 58238 | 59038 |
| POPC01000413.1 | 99.251 | 801 | 6 | 0 | 31607 | 30807 |
| POOO01000035.1 | 99.251 | 801 | 6 | 0 | 32070 | 32870 |
| POLH01000125.1 | 99.251 | 801 | 6 | 0 | 31711 | 30911 |
| POLF01000076.1 | 99.251 | 801 | 6 | 0 | 33239 | 34039 |
| POLE01000040.1 | 99.251 | 801 | 6 | 0 | 33239 | 34039 |
| POKW01000058.1 | 99.251 | 801 | 6 | 0 | 150325 | 149525 |
| POLD01000054.1 | 99.251 | 801 | 6 | 0 | 33302 | 34102 |
| POJT01000031.1 | 99.251 | 801 | 6 | 0 | 64185 | 63385 |
| POJO01000048.1 | 99.251 | 801 | 6 | 0 | 31576 | 32376 |
| POJR01000094.1 | 99.251 | 801 | 6 | 0 | 10231 | 9431 |
| POOM01000086.1 | 99.251 | 801 | 6 | 0 | 149873 | 150673 |
| POOI01000134.1 | 99.251 | 801 | 6 | 0 | 30130 | 29330 |
| PONN01000034.1 | 99.251 | 801 | 6 | 0 | 149873 | 150673 |
| PONM01000031.1 | 99.251 | 801 | 6 | 0 | 149873 | 150673 |
| POLK01000051.1 | 99.251 | 801 | 6 | 0 | 150325 | 149525 |
| POLI01000047.1 | 99.251 | 801 | 6 | 0 | 33479 | 34279 |
| POKX01000058.1 | 99.251 | 801 | 6 | 0 | 31640 | 30840 |
| POKV01000047.1 | 99.251 | 801 | 6 | 0 | 4333 | 5133 |
| POKP01000056.1 | 99.251 | 801 | 6 | 0 | 10174 | 9374 |
| POKO01000216.1 | 99.251 | 801 | 6 | 0 | 70940 | 70140 |
| POJV01000710.1 | 99.251 | 801 | 6 | 0 | 31695 | 32495 |
| POJS01000033.1 | 99.251 | 801 | 6 | 0 | 15774 | 14974 |
| POJQ01000082.1 | 99.251 | 801 | 6 | 0 | 15774 | 14974 |
| POJP01000036.1 | 99.251 | 801 | 6 | 0 | 64214 | 63414 |
| POIV01000064.1 | 99.251 | 801 | 6 | 0 | 1507 | 2307 |
| POID01000093.1 | 99.251 | 801 | 6 | 0 | 44718 | 43918 |
| ASCA01000056.1 | 99.251 | 801 | 6 | 0 | 89511 | 88711 |
| CEKE01000067.1 | 99.251 | 801 | 6 | 0 | 47860 | 48660 |
| CEJS01000028.1 | 99.251 | 801 | 6 | 0 | 53345 | 54145 |
| CEHU01000023.1 | 99.251 | 801 | 6 | 0 | 42054 | 41254 |
| CEJK01000007.1 | 99.251 | 801 | 6 | 0 | 110350 | 111150 |
| CEJP01000015.1 | 99.251 | 801 | 6 | 0 | 30241 | 29441 |
| CEGO01000047.1 | 99.251 | 801 | 6 | 0 | 108538 | 109338 |
| CEEX01000072.1 | 99.251 | 801 | 6 | 0 | 12503 | 13303 |
| CEDT01000290.1 | 99.251 | 801 | 6 | 0 | 33871 | 34671 |
| ALMJ01000016.1 | 99.251 | 801 | 6 | 0 | 30252 | 29452 |
| ALLK01000017.1 | 99.251 | 801 | 6 | 0 | 25877 | 26677 |
| ALLC01000055.1 | 99.251 | 801 | 6 | 0 | 12116 | 12916 |
| NZ_CAMUSR010000001.1 | 99.126 | 801 | 7 | 0 | 1130169 | 1130969 |
| NZ_OX352996.1 | 99.126 | 801 | 7 | 0 | 439955 | 439155 |
| NZ_OX352941.1 | 99.126 | 801 | 7 | 0 | 795917 | 796717 |
| NZ_OX352940.1 | 99.126 | 801 | 7 | 0 | 439956 | 439156 |
| NZ_CAMUSF010000001.1 | 99.126 | 801 | 7 | 0 | 1226310 | 1227110 |
| NZ_OX352944.1 | 99.126 | 801 | 7 | 0 | 1043463 | 1044263 |
| NZ_OX352831.1 | 99.126 | 801 | 7 | 0 | 1129990 | 1130790 |
| NZ_OX352806.1 | 99.126 | 801 | 7 | 0 | 1130179 | 1130979 |
| NZ_LR738720.1 | 99.126 | 801 | 7 | 0 | 1130330 | 1131130 |
| NZ_LR738722.1 | 99.126 | 801 | 7 | 0 | 1130170 | 1130970 |
| NZ_FIKM01000007.1 | 99.126 | 801 | 7 | 0 | 114377 | 113577 |
| NZ_FIFT01000013.1 | 99.126 | 801 | 7 | 0 | 66431 | 65631 |
| NZ_FIJD01000013.1 | 99.126 | 801 | 7 | 0 | 65176 | 64376 |
| NZ_FIGS01000004.1 | 99.126 | 801 | 7 | 0 | 199004 | 198204 |
| NZ_FIGG01000001.1 | 99.126 | 801 | 7 | 0 | 441095 | 440295 |
| NZ_FIKN01000013.1 | 99.126 | 801 | 7 | 0 | 65186 | 64386 |
| NZ_FIFZ01000028.1 | 99.126 | 801 | 7 | 0 | 3555 | 4355 |
| NZ_FIHZ01000010.1 | 99.126 | 801 | 7 | 0 | 65191 | 64391 |
| NZ_FIFO01000004.1 | 99.126 | 801 | 7 | 0 | 90948 | 91748 |
| NZ_FIKF01000022.1 | 99.126 | 801 | 7 | 0 | 15111 | 14311 |
| NZ_FIFP01000016.1 | 99.126 | 801 | 7 | 0 | 1436 | 2236 |
| NZ_FIFN01000011.1 | 99.126 | 801 | 7 | 0 | 961 | 161 |
| NZ_FIHI01000001.1 | 99.126 | 801 | 7 | 0 | 213750 | 214550 |
| NZ_FIFM01000003.1 | 99.126 | 801 | 7 | 0 | 127374 | 128174 |
| NZ_FIFJ01000034.1 | 99.126 | 801 | 7 | 0 | 14321 | 13521 |
| NZ_FIJQ01000012.1 | 99.126 | 801 | 7 | 0 | 7766 | 8566 |
| NZ_FIHQ01000018.1 | 99.126 | 801 | 7 | 0 | 7776 | 8576 |
| NZ_FIHT01000011.1 | 99.126 | 801 | 7 | 0 | 7777 | 8577 |
| NZ_FIHR01000020.1 | 99.126 | 801 | 7 | 0 | 7754 | 8554 |
| NZ_FIID01000016.1 | 99.126 | 801 | 7 | 0 | 30250 | 29450 |
| NZ_FIKW01000013.1 | 99.126 | 801 | 7 | 0 | 65186 | 64386 |
| NZ_FIHN01000001.1 | 99.126 | 801 | 7 | 0 | 348898 | 349698 |
| NZ_FILF01000013.1 | 99.126 | 801 | 7 | 0 | 7775 | 8575 |
| NZ_FIHY01000020.1 | 99.126 | 801 | 7 | 0 | 20217 | 19417 |
| NZ_CZHA01000022.1 | 99.126 | 801 | 7 | 0 | 82146 | 82946 |
| NZ_CZEJ01000062.1 | 99.126 | 801 | 7 | 0 | 172522 | 171722 |
| NZ_CZGY01000017.1 | 99.126 | 801 | 7 | 0 | 81311 | 82111 |
| NZ_CZDN01000063.1 | 99.126 | 801 | 7 | 0 | 172522 | 171722 |
| NZ_CZEE01000062.1 | 99.126 | 801 | 7 | 0 | 172527 | 171727 |
| NZ_CZDW01000066.1 | 99.126 | 801 | 7 | 0 | 172523 | 171723 |
| NZ_CZDZ01000066.1 | 99.126 | 801 | 7 | 0 | 55597 | 56397 |
| NZ_CZEV01000003.1 | 99.126 | 801 | 7 | 0 | 14986 | 14186 |
| NZ_CZDU01000020.1 | 99.126 | 801 | 7 | 0 | 7786 | 8586 |
| NZ_CZEH01000063.1 | 99.126 | 801 | 7 | 0 | 61322 | 60522 |
| NZ_CZDY01000010.1 | 99.126 | 801 | 7 | 0 | 48219 | 47419 |
| NZ_CZGP01000020.1 | 99.126 | 801 | 7 | 0 | 14985 | 14185 |
| NZ_CZES01000061.1 | 99.126 | 801 | 7 | 0 | 172522 | 171722 |
| NZ_CZDS01000063.1 | 99.126 | 801 | 7 | 0 | 172522 | 171722 |
| NZ_CZEK01000071.1 | 99.126 | 801 | 7 | 0 | 81312 | 82112 |
| NZ_CP149804.1 | 99.126 | 801 | 7 | 0 | 899787 | 898987 |
| NZ_JAVIGQ010000027.1 | 99.126 | 801 | 7 | 0 | 32113 | 32913 |
| NZ_JAZDVU010000022.1 | 99.126 | 801 | 7 | 0 | 13048 | 12248 |
| NZ_CP139882.1 | 99.126 | 801 | 7 | 0 | 2206645 | 2205845 |
| NZ_JAXKWG010000007.1 | 99.126 | 801 | 7 | 0 | 103061 | 102261 |
| NZ_JAXKWO010000001.1 | 99.126 | 801 | 7 | 0 | 209125 | 208325 |
| NZ_JAWWZK010000001.1 | 99.126 | 801 | 7 | 0 | 1541 | 2341 |
| NZ_JAWWZC010000013.1 | 99.126 | 801 | 7 | 0 | 65193 | 64393 |
| NZ_JAUTGX010000005.1 | 99.126 | 801 | 7 | 0 | 1564 | 2364 |
| NZ_JAUTGH010000012.1 | 99.126 | 801 | 7 | 0 | 7815 | 8615 |
| NZ_JAUTGG010000012.1 | 99.126 | 801 | 7 | 0 | 7815 | 8615 |
| NZ_JAUTFZ010000012.1 | 99.126 | 801 | 7 | 0 | 7815 | 8615 |
| NZ_JAUTFL010000001.1 | 99.126 | 801 | 7 | 0 | 148773 | 147973 |
| NZ_JAUTEO010000001.1 | 99.126 | 801 | 7 | 0 | 163879 | 163079 |
| NZ_JAUTEP010000001.1 | 99.126 | 801 | 7 | 0 | 3983 | 4783 |
| NZ_JAUTEQ010000001.1 | 99.126 | 801 | 7 | 0 | 163879 | 163079 |
| NZ_JAVMBK010000040.1 | 99.126 | 801 | 7 | 0 | 1351 | 2151 |
| NZ_JAVLUW010000001.1 | 99.126 | 801 | 7 | 0 | 127712 | 128512 |
| NZ_JARATE010000010.1 | 99.126 | 801 | 7 | 0 | 7831 | 8631 |
| NZ_JARATD010000021.1 | 99.126 | 801 | 7 | 0 | 7831 | 8631 |
| NZ_JANFME010000005.1 | 99.126 | 801 | 7 | 0 | 57289 | 58089 |
| NZ_JARBFO010000001.1 | 99.126 | 801 | 7 | 0 | 60255 | 59455 |
| NZ_CP102148.1 | 99.126 | 801 | 7 | 0 | 852918 | 852118 |
| NZ_JAMWES010000010.1 | 99.126 | 801 | 7 | 0 | 65193 | 64393 |
| NZ_CP100432.1 | 99.126 | 801 | 7 | 0 | 899528 | 898728 |
| NZ_JAASEX010000010.1 | 99.126 | 801 | 7 | 0 | 55920 | 55120 |
| NZ_JAASEJ010000026.1 | 99.126 | 801 | 7 | 0 | 8185 | 8985 |
| NZ_JAASEB010000012.1 | 99.126 | 801 | 7 | 0 | 65530 | 64730 |
| NZ_JAASEA010000015.1 | 99.126 | 801 | 7 | 0 | 55821 | 55021 |
| NZ_JAASDJ010000014.1 | 99.126 | 801 | 7 | 0 | 8179 | 8979 |
| NZ_JAASCT010000004.1 | 99.126 | 801 | 7 | 0 | 8183 | 8983 |
| NZ_JAASCR010000017.1 | 99.126 | 801 | 7 | 0 | 129830 | 129030 |
| NZ_JAASCL010000013.1 | 99.126 | 801 | 7 | 0 | 55617 | 54817 |
| NZ_JAASCI010000018.1 | 99.126 | 801 | 7 | 0 | 8168 | 8968 |
| NZ_JAASBM010000011.1 | 99.126 | 801 | 7 | 0 | 8131 | 8931 |
| NZ_JAASBK010000017.1 | 99.126 | 801 | 7 | 0 | 10528 | 9728 |
| NZ_JAASBD010000022.1 | 99.126 | 801 | 7 | 0 | 65634 | 64834 |
| NZ_JAKTBO010000011.1 | 99.126 | 801 | 7 | 0 | 65194 | 64394 |
| NZ_JAKOFZ010000023.1 | 99.126 | 801 | 7 | 0 | 7836 | 8636 |
| NZ_JAKOFX010000014.1 | 99.126 | 801 | 7 | 0 | 7849 | 8649 |
| NZ_JAKOFT010000012.1 | 99.126 | 801 | 7 | 0 | 7836 | 8636 |
| NZ_JAKOFQ010000014.1 | 99.126 | 801 | 7 | 0 | 7836 | 8636 |
| NZ_JAKOFP010000014.1 | 99.126 | 801 | 7 | 0 | 7836 | 8636 |
| NZ_JAKOFN010000015.1 | 99.126 | 801 | 7 | 0 | 7836 | 8636 |
| NZ_JAKOFO010000014.1 | 99.126 | 801 | 7 | 0 | 55283 | 54483 |
| NZ_JAKOFM010000016.1 | 99.126 | 801 | 7 | 0 | 7836 | 8636 |
| NZ_JAKOFL010000017.1 | 99.126 | 801 | 7 | 0 | 7836 | 8636 |
| NZ_JAKOFG010000010.1 | 99.126 | 801 | 7 | 0 | 7943 | 8743 |
| NZ_JAKOFK010000013.1 | 99.126 | 801 | 7 | 0 | 65330 | 64530 |
| NZ_JAKOFI010000015.1 | 99.126 | 801 | 7 | 0 | 7836 | 8636 |
| NZ_JAKOFJ010000012.1 | 99.126 | 801 | 7 | 0 | 65330 | 64530 |
| NZ_JAKOFF010000013.1 | 99.126 | 801 | 7 | 0 | 7836 | 8636 |
| NZ_JAKOFE010000010.1 | 99.126 | 801 | 7 | 0 | 7943 | 8743 |
| NZ_JAKOFD010000013.1 | 99.126 | 801 | 7 | 0 | 7836 | 8636 |
| NZ_JAKOFC010000014.1 | 99.126 | 801 | 7 | 0 | 7836 | 8636 |
| NZ_JAKOFA010000013.1 | 99.126 | 801 | 7 | 0 | 7836 | 8636 |
| NZ_JAFLMP010000012.1 | 99.126 | 801 | 7 | 0 | 7836 | 8636 |
| NZ_JAFLMR010000023.1 | 99.126 | 801 | 7 | 0 | 12863 | 12063 |
| NZ_JAFLMQ010000012.1 | 99.126 | 801 | 7 | 0 | 7836 | 8636 |
| NZ_JAFLMO010000011.1 | 99.126 | 801 | 7 | 0 | 7836 | 8636 |
| NZ_JAFHDP010000012.1 | 99.126 | 801 | 7 | 0 | 7836 | 8636 |
| NZ_JAFHDO010000012.1 | 99.126 | 801 | 7 | 0 | 7836 | 8636 |
| NZ_JAFHDJ010000010.1 | 99.126 | 801 | 7 | 0 | 7836 | 8636 |
| NZ_JAFHDK010000012.1 | 99.126 | 801 | 7 | 0 | 7836 | 8636 |
| NZ_CP079193.1 | 99.126 | 801 | 7 | 0 | 861758 | 860958 |
| NZ_CP076517.1 | 99.126 | 801 | 7 | 0 | 906048 | 905248 |
| NZ_VZNM01000011.1 | 99.126 | 801 | 7 | 0 | 65218 | 64418 |
| NZ_WACE01000016.1 | 99.126 | 801 | 7 | 0 | 7709 | 8509 |
| NZ_CP082778.1 | 99.126 | 801 | 7 | 0 | 869213 | 868413 |
| NZ_CP068708.1 | 99.126 | 801 | 7 | 0 | 1113788 | 1114588 |
| NZ_JABTYX010000012.1 | 99.126 | 801 | 7 | 0 | 7799 | 8599 |
| NZ_JABLFD010000012.1 | 99.126 | 801 | 7 | 0 | 7782 | 8582 |
| NZ_JABLFN010000014.1 | 99.126 | 801 | 7 | 0 | 65187 | 64387 |
| NZ_JABLGP010000012.1 | 99.126 | 801 | 7 | 0 | 65847 | 65047 |
| NZ_JABLHM010000024.1 | 99.126 | 801 | 7 | 0 | 7832 | 8632 |
| NZ_JABLHZ010000012.1 | 99.126 | 801 | 7 | 0 | 65443 | 64643 |
| NZ_JABLIA010000033.1 | 99.126 | 801 | 7 | 0 | 7829 | 8629 |
| NZ_JABLIW010000002.1 | 99.126 | 801 | 7 | 0 | 222930 | 223730 |
| NZ_JABLIX010000011.1 | 99.126 | 801 | 7 | 0 | 65239 | 64439 |
| NZ_JABLJG010000010.1 | 99.126 | 801 | 7 | 0 | 65241 | 64441 |
| NZ_JABLGX010000024.1 | 99.126 | 801 | 7 | 0 | 1021 | 221 |
| NZ_JABLHS010000002.1 | 99.126 | 801 | 7 | 0 | 218489 | 219289 |
| NZ_JABLIM010000006.1 | 99.126 | 801 | 7 | 0 | 1448 | 2248 |
| NZ_JABLJE010000011.1 | 99.126 | 801 | 7 | 0 | 65238 | 64438 |
| NZ_JABLHD010000012.1 | 99.126 | 801 | 7 | 0 | 7832 | 8632 |
| NZ_JABLHT010000003.1 | 99.126 | 801 | 7 | 0 | 242018 | 242818 |
| NZ_JABLJY010000027.1 | 99.126 | 801 | 7 | 0 | 7832 | 8632 |
| NZ_JABLLA010000001.1 | 99.126 | 801 | 7 | 0 | 31445 | 32245 |
| NZ_JABLLH010000002.1 | 99.126 | 801 | 7 | 0 | 31591 | 32391 |
| NZ_JABLLI010000001.1 | 99.126 | 801 | 7 | 0 | 106470 | 107270 |
| NZ_JABLLL010000001.1 | 99.126 | 801 | 7 | 0 | 254093 | 254893 |
| NZ_JABLMQ010000033.1 | 99.126 | 801 | 7 | 0 | 995 | 195 |
| NZ_JABLMT010000001.1 | 99.126 | 801 | 7 | 0 | 155408 | 154608 |
| NZ_JABLMR010000001.1 | 99.126 | 801 | 7 | 0 | 555037 | 555837 |
| NZ_JABLMS010000031.1 | 99.126 | 801 | 7 | 0 | 1422 | 2222 |
| NZ_JABLMU010000001.1 | 99.126 | 801 | 7 | 0 | 31935 | 32735 |
| NZ_JABLMW010000002.1 | 99.126 | 801 | 7 | 0 | 199491 | 200291 |
| NZ_JABLLB010000001.1 | 99.126 | 801 | 7 | 0 | 107647 | 108447 |
| NZ_JABLNQ010000001.1 | 99.126 | 801 | 7 | 0 | 31775 | 32575 |
| NZ_JABLLC010000001.1 | 99.126 | 801 | 7 | 0 | 254088 | 254888 |
| NZ_JABLNR010000002.1 | 99.126 | 801 | 7 | 0 | 92338 | 93138 |
| NZ_JABLNT010000032.1 | 99.126 | 801 | 7 | 0 | 1422 | 2222 |
| NZ_JABLLW010000004.1 | 99.126 | 801 | 7 | 0 | 91562 | 92362 |
| NZ_JABLOA010000001.1 | 99.126 | 801 | 7 | 0 | 31507 | 32307 |
| NZ_JABLOB010000001.1 | 99.126 | 801 | 7 | 0 | 31542 | 32342 |
| NZ_JABLOF010000035.1 | 99.126 | 801 | 7 | 0 | 1422 | 2222 |
| NZ_JABLMH010000018.1 | 99.126 | 801 | 7 | 0 | 46750 | 45950 |
| NZ_JABLND010000001.1 | 99.126 | 801 | 7 | 0 | 269839 | 269039 |
| NZ_JABLOW010000016.1 | 99.126 | 801 | 7 | 0 | 52715 | 51915 |
| NZ_JABLNX010000013.1 | 99.126 | 801 | 7 | 0 | 2312 | 1512 |
| NZ_JABLPM010000002.1 | 99.126 | 801 | 7 | 0 | 269876 | 269076 |
| NZ_JABLNW010000033.1 | 99.126 | 801 | 7 | 0 | 1422 | 2222 |
| NZ_JABKST010000009.1 | 99.126 | 801 | 7 | 0 | 7826 | 8626 |
| NZ_JABKSZ010000012.1 | 99.126 | 801 | 7 | 0 | 65241 | 64441 |
| NZ_JABKTA010000022.1 | 99.126 | 801 | 7 | 0 | 2780 | 1980 |
| NZ_JABKTB010000008.1 | 99.126 | 801 | 7 | 0 | 133331 | 132531 |
| NZ_JABKTE010000011.1 | 99.126 | 801 | 7 | 0 | 65895 | 65095 |
| NZ_JABKUG010000011.1 | 99.126 | 801 | 7 | 0 | 65240 | 64440 |
| NZ_JABKUA010000014.1 | 99.126 | 801 | 7 | 0 | 65241 | 64441 |
| NZ_JABKTZ010000007.1 | 99.126 | 801 | 7 | 0 | 91739 | 92539 |
| NZ_JABKYA010000012.1 | 99.126 | 801 | 7 | 0 | 66419 | 65619 |
| NZ_JABKYB010000014.1 | 99.126 | 801 | 7 | 0 | 7832 | 8632 |
| NZ_JABKYD010000019.1 | 99.126 | 801 | 7 | 0 | 34658 | 33858 |
| NZ_JABKYC010000013.1 | 99.126 | 801 | 7 | 0 | 66428 | 65628 |
| NZ_JABKUR010000010.1 | 99.126 | 801 | 7 | 0 | 65240 | 64440 |
| NZ_JABKYE010000004.1 | 99.126 | 801 | 7 | 0 | 198834 | 198034 |
| NZ_JABKYF010000012.1 | 99.126 | 801 | 7 | 0 | 66428 | 65628 |
| NZ_JABKYG010000012.1 | 99.126 | 801 | 7 | 0 | 7831 | 8631 |
| NZ_JABKYK010000011.1 | 99.126 | 801 | 7 | 0 | 65241 | 64441 |
| NZ_JABKVZ010000005.1 | 99.126 | 801 | 7 | 0 | 91739 | 92539 |
| NZ_JABKZP010000038.1 | 99.126 | 801 | 7 | 0 | 4720 | 5520 |
| NZ_JABKZY010000011.1 | 99.126 | 801 | 7 | 0 | 971 | 171 |
| NZ_JABKZZ010000003.1 | 99.126 | 801 | 7 | 0 | 69160 | 68360 |
| NZ_JABKWP010000002.1 | 99.126 | 801 | 7 | 0 | 60712 | 59912 |
| NZ_JABKZX010000002.1 | 99.126 | 801 | 7 | 0 | 163297 | 164097 |
| NZ_JABLAE010000011.1 | 99.126 | 801 | 7 | 0 | 12664 | 13464 |
| NZ_JABLAD010000034.1 | 99.126 | 801 | 7 | 0 | 1397 | 2197 |
| NZ_JABLAQ010000005.1 | 99.126 | 801 | 7 | 0 | 68658 | 67858 |
| NZ_JABLAT010000012.1 | 99.126 | 801 | 7 | 0 | 65191 | 64391 |
| NZ_JABLBN010000001.1 | 99.126 | 801 | 7 | 0 | 132808 | 132008 |
| NZ_JABLBY010000017.1 | 99.126 | 801 | 7 | 0 | 7781 | 8581 |
| NZ_JABLBZ010000027.1 | 99.126 | 801 | 7 | 0 | 15122 | 14322 |
| NZ_JABLCB010000003.1 | 99.126 | 801 | 7 | 0 | 7782 | 8582 |
| NZ_JABLCG010000022.1 | 99.126 | 801 | 7 | 0 | 6103 | 6903 |
| NZ_JABLCH010000002.1 | 99.126 | 801 | 7 | 0 | 65188 | 64388 |
| NZ_JABLBW010000001.1 | 99.126 | 801 | 7 | 0 | 255406 | 256206 |
| NZ_JABLEW010000013.1 | 99.126 | 801 | 7 | 0 | 7781 | 8581 |
| NZ_JABLDA010000009.1 | 99.126 | 801 | 7 | 0 | 7773 | 8573 |
| NZ_JABLEU010000013.1 | 99.126 | 801 | 7 | 0 | 7779 | 8579 |
| NZ_JABLCL010000004.1 | 99.126 | 801 | 7 | 0 | 70458 | 69658 |
| NZ_JABLDW010000011.1 | 99.126 | 801 | 7 | 0 | 7775 | 8575 |
| NZ_JABLEN010000028.1 | 99.126 | 801 | 7 | 0 | 7781 | 8581 |
| NZ_JAAVJW010000025.1 | 99.126 | 801 | 7 | 0 | 15487 | 16287 |
| NZ_JAAVJV010000008.1 | 99.126 | 801 | 7 | 0 | 60478 | 59678 |
| NZ_VIEL01000002.1 | 99.126 | 801 | 7 | 0 | 342153 | 342953 |
| NZ_RRZP01000019.1 | 99.126 | 801 | 7 | 0 | 7913 | 8713 |
| NZ_RRZQ01000007.1 | 99.126 | 801 | 7 | 0 | 15738 | 16538 |
| NZ_PONZ01000490.1 | 99.126 | 801 | 7 | 0 | 2807 | 2007 |
| NZ_POKM01000118.1 | 99.126 | 801 | 7 | 0 | 29938 | 30738 |
| NZ_PONL01000021.1 | 99.126 | 801 | 7 | 0 | 55782 | 56582 |
| NZ_PONK01000081.1 | 99.126 | 801 | 7 | 0 | 55782 | 56582 |
| NZ_POMV01000100.1 | 99.126 | 801 | 7 | 0 | 4975 | 5775 |
| NZ_POJK01000088.1 | 99.126 | 801 | 7 | 0 | 3337 | 2537 |
| NZ_POIO01000311.1 | 99.126 | 801 | 7 | 0 | 52457 | 51657 |
| NZ_CP025043.1 | 99.126 | 801 | 7 | 0 | 1078342 | 1077542 |
| NZ_CEIW01000027.1 | 99.126 | 801 | 7 | 0 | 65173 | 64373 |
| NZ_CEIS01000041.1 | 99.126 | 801 | 7 | 0 | 65174 | 64374 |
| NZ_CEDP01000028.1 | 99.126 | 801 | 7 | 0 | 7764 | 8564 |
| NZ_CEIY01000015.1 | 99.126 | 801 | 7 | 0 | 65172 | 64372 |
| NZ_CEDE01000055.1 | 99.126 | 801 | 7 | 0 | 65166 | 64366 |
| NZ_CEGY01000010.1 | 99.126 | 801 | 7 | 0 | 65172 | 64372 |
| NZ_CEKO01000011.1 | 99.126 | 801 | 7 | 0 | 7760 | 8560 |
| NZ_CEEV01000067.1 | 99.126 | 801 | 7 | 0 | 61275 | 60475 |
| NZ_CEDO01000047.1 | 99.126 | 801 | 7 | 0 | 1438 | 2238 |
| NZ_CECY01000098.1 | 99.126 | 801 | 7 | 0 | 20416 | 19616 |
| NZ_CEEH01000046.1 | 99.126 | 801 | 7 | 0 | 50839 | 50039 |
| NZ_CEIA01000010.1 | 99.126 | 801 | 7 | 0 | 65176 | 64376 |
| NZ_CEHO01000049.1 | 99.126 | 801 | 7 | 0 | 15099 | 14299 |
| NZ_CEHV01000072.1 | 99.126 | 801 | 7 | 0 | 1386 | 2186 |
| NZ_CEEQ01000020.1 | 99.126 | 801 | 7 | 0 | 65180 | 64380 |
| NZ_CEDC01000010.1 | 99.126 | 801 | 7 | 0 | 954 | 154 |
| NZ_CEER01000021.1 | 99.126 | 801 | 7 | 0 | 67534 | 66734 |
| NZ_CEGX01000020.1 | 99.126 | 801 | 7 | 0 | 7766 | 8566 |
| NZ_CEJO01000013.1 | 99.126 | 801 | 7 | 0 | 30242 | 29442 |
| NZ_CEEC01000014.1 | 99.126 | 801 | 7 | 0 | 65174 | 64374 |
| NZ_CEEN01000080.1 | 99.126 | 801 | 7 | 0 | 20202 | 19402 |
| NZ_CEDJ01000039.1 | 99.126 | 801 | 7 | 0 | 81313 | 80513 |
| NZ_CEEZ01000028.1 | 99.126 | 801 | 7 | 0 | 68313 | 67513 |
| NZ_CECR01000027.1 | 99.126 | 801 | 7 | 0 | 14320 | 13520 |
| NC_022665.1 | 99.126 | 801 | 7 | 0 | 899450 | 898650 |
| NZ_ALME01000043.1 | 99.126 | 801 | 7 | 0 | 54661 | 53861 |
| NZ_ALMC01000008.1 | 99.126 | 801 | 7 | 0 | 7776 | 8576 |
| NZ_ALLY01000004.1 | 99.126 | 801 | 7 | 0 | 60670 | 61470 |
| NZ_ALLT01000034.1 | 99.126 | 801 | 7 | 0 | 65181 | 64381 |
| NZ_ALLP01000101.1 | 99.126 | 801 | 7 | 0 | 62952 | 63752 |
| NZ_ALLH01000048.1 | 99.126 | 801 | 7 | 0 | 279983 | 280783 |
| NZ_ALKX01000104.1 | 99.126 | 801 | 7 | 0 | 70845 | 70045 |
| NZ_ALKV01000002.1 | 99.126 | 801 | 7 | 0 | 65189 | 64389 |
| CAMUSR010000001.1 | 99.126 | 801 | 7 | 0 | 1130169 | 1130969 |
| OX352996.1 | 99.126 | 801 | 7 | 0 | 439955 | 439155 |
| OX352941.1 | 99.126 | 801 | 7 | 0 | 795917 | 796717 |
| OX352940.1 | 99.126 | 801 | 7 | 0 | 439956 | 439156 |
| CAMUSF010000001.1 | 99.126 | 801 | 7 | 0 | 1226310 | 1227110 |
| OX352944.1 | 99.126 | 801 | 7 | 0 | 1043463 | 1044263 |
| OX352831.1 | 99.126 | 801 | 7 | 0 | 1129990 | 1130790 |
| OX352806.1 | 99.126 | 801 | 7 | 0 | 1130179 | 1130979 |
| LR738720.1 | 99.126 | 801 | 7 | 0 | 1130330 | 1131130 |
| LR738722.1 | 99.126 | 801 | 7 | 0 | 1130170 | 1130970 |
| FIKM01000007.1 | 99.126 | 801 | 7 | 0 | 114377 | 113577 |
| FIFT01000013.1 | 99.126 | 801 | 7 | 0 | 66431 | 65631 |
| FIJD01000013.1 | 99.126 | 801 | 7 | 0 | 65176 | 64376 |
| FIGS01000004.1 | 99.126 | 801 | 7 | 0 | 199004 | 198204 |
| FIGG01000001.1 | 99.126 | 801 | 7 | 0 | 441095 | 440295 |
| FIKN01000013.1 | 99.126 | 801 | 7 | 0 | 65186 | 64386 |
| FIFZ01000028.1 | 99.126 | 801 | 7 | 0 | 3555 | 4355 |
| FIHZ01000010.1 | 99.126 | 801 | 7 | 0 | 65191 | 64391 |
| FIFO01000004.1 | 99.126 | 801 | 7 | 0 | 90948 | 91748 |
| FIKF01000022.1 | 99.126 | 801 | 7 | 0 | 15111 | 14311 |
| FIFP01000016.1 | 99.126 | 801 | 7 | 0 | 1436 | 2236 |
| FIFN01000011.1 | 99.126 | 801 | 7 | 0 | 961 | 161 |
| FIHI01000001.1 | 99.126 | 801 | 7 | 0 | 213750 | 214550 |
| FIFM01000003.1 | 99.126 | 801 | 7 | 0 | 127374 | 128174 |
| FIFJ01000034.1 | 99.126 | 801 | 7 | 0 | 14321 | 13521 |
| FIJQ01000012.1 | 99.126 | 801 | 7 | 0 | 7766 | 8566 |
| FIHQ01000018.1 | 99.126 | 801 | 7 | 0 | 7776 | 8576 |
| FIHT01000011.1 | 99.126 | 801 | 7 | 0 | 7777 | 8577 |
| FIHR01000020.1 | 99.126 | 801 | 7 | 0 | 7754 | 8554 |
| FIID01000016.1 | 99.126 | 801 | 7 | 0 | 30250 | 29450 |
| FIKW01000013.1 | 99.126 | 801 | 7 | 0 | 65186 | 64386 |
| FIHN01000001.1 | 99.126 | 801 | 7 | 0 | 348898 | 349698 |
| FILF01000013.1 | 99.126 | 801 | 7 | 0 | 7775 | 8575 |
| FIHY01000020.1 | 99.126 | 801 | 7 | 0 | 20217 | 19417 |
| CZHA01000022.1 | 99.126 | 801 | 7 | 0 | 82146 | 82946 |
| CZEJ01000062.1 | 99.126 | 801 | 7 | 0 | 172522 | 171722 |
| CZGY01000017.1 | 99.126 | 801 | 7 | 0 | 81311 | 82111 |
| CZDN01000063.1 | 99.126 | 801 | 7 | 0 | 172522 | 171722 |
| CZEE01000062.1 | 99.126 | 801 | 7 | 0 | 172527 | 171727 |
| CZDW01000066.1 | 99.126 | 801 | 7 | 0 | 172523 | 171723 |
| CZDZ01000066.1 | 99.126 | 801 | 7 | 0 | 55597 | 56397 |
| CZEV01000003.1 | 99.126 | 801 | 7 | 0 | 14986 | 14186 |
| CZDU01000020.1 | 99.126 | 801 | 7 | 0 | 7786 | 8586 |
| CZEH01000063.1 | 99.126 | 801 | 7 | 0 | 61322 | 60522 |
| CZDY01000010.1 | 99.126 | 801 | 7 | 0 | 48219 | 47419 |
| CZGP01000020.1 | 99.126 | 801 | 7 | 0 | 14985 | 14185 |
| CZES01000061.1 | 99.126 | 801 | 7 | 0 | 172522 | 171722 |
| CZDS01000063.1 | 99.126 | 801 | 7 | 0 | 172522 | 171722 |
| CZEK01000071.1 | 99.126 | 801 | 7 | 0 | 81312 | 82112 |
| CP149804.1 | 99.126 | 801 | 7 | 0 | 899787 | 898987 |
| JAVIGQ010000027.1 | 99.126 | 801 | 7 | 0 | 32113 | 32913 |
| JAZDVU010000022.1 | 99.126 | 801 | 7 | 0 | 13048 | 12248 |
| DATUCG010000006.1 | 99.126 | 801 | 7 | 0 | 16913 | 17713 |
| DATUCF010000036.1 | 99.126 | 801 | 7 | 0 | 10421 | 9621 |
| DATUCE010000006.1 | 99.126 | 801 | 7 | 0 | 19409 | 20209 |
| DATUBG010000026.1 | 99.126 | 801 | 7 | 0 | 34787 | 33987 |
| DATTWQ010000003.1 | 99.126 | 801 | 7 | 0 | 19874 | 20674 |
| CP139882.1 | 99.126 | 801 | 7 | 0 | 2206645 | 2205845 |
| JAXKWG010000007.1 | 99.126 | 801 | 7 | 0 | 103061 | 102261 |
| JAXKWO010000001.1 | 99.126 | 801 | 7 | 0 | 209125 | 208325 |
| DASGYR010000015.1 | 99.126 | 801 | 7 | 0 | 7707 | 8507 |
| DASGXG010000013.1 | 99.126 | 801 | 7 | 0 | 7691 | 8491 |
| DASGUQ010000002.1 | 99.126 | 801 | 7 | 0 | 20120 | 19320 |
| DASGUP010000011.1 | 99.126 | 801 | 7 | 0 | 7708 | 8508 |
| DASGTS010000001.1 | 99.126 | 801 | 7 | 0 | 32784 | 33584 |
| DASGTJ010000001.1 | 99.126 | 801 | 7 | 0 | 32630 | 33430 |
| DASGRZ010000015.1 | 99.126 | 801 | 7 | 0 | 21091 | 20291 |
| DASGRU010000015.1 | 99.126 | 801 | 7 | 0 | 21091 | 20291 |
| DASGRR010000012.1 | 99.126 | 801 | 7 | 0 | 7703 | 8503 |
| DASGRO010000019.1 | 99.126 | 801 | 7 | 0 | 30223 | 29423 |
| DASGQM010000001.1 | 99.126 | 801 | 7 | 0 | 289529 | 290329 |
| DASGQI010000001.1 | 99.126 | 801 | 7 | 0 | 69483 | 70283 |
| DASGPP010000004.1 | 99.126 | 801 | 7 | 0 | 32687 | 33487 |
| DASGOT010000004.1 | 99.126 | 801 | 7 | 0 | 32687 | 33487 |
| DASGOH010000002.1 | 99.126 | 801 | 7 | 0 | 20118 | 19318 |
| DASGNP010000004.1 | 99.126 | 801 | 7 | 0 | 21093 | 20293 |
| DASGMX010000009.1 | 99.126 | 801 | 7 | 0 | 7703 | 8503 |
| DASGMW010000001.1 | 99.126 | 801 | 7 | 0 | 210915 | 211715 |
| DASGIZ010000011.1 | 99.126 | 801 | 7 | 0 | 7703 | 8503 |
| DASGIR010000027.1 | 99.126 | 801 | 7 | 0 | 897 | 97 |
| DASGIL010000012.1 | 99.126 | 801 | 7 | 0 | 7708 | 8508 |
| DASGIC010000025.1 | 99.126 | 801 | 7 | 0 | 7703 | 8503 |
| DASGHV010000001.1 | 99.126 | 801 | 7 | 0 | 30339 | 29539 |
| DASGHU010000001.1 | 99.126 | 801 | 7 | 0 | 30339 | 29539 |
| DASGHO010000014.1 | 99.126 | 801 | 7 | 0 | 7703 | 8503 |
| DASGHN010000029.1 | 99.126 | 801 | 7 | 0 | 7700 | 8500 |
| DASGHB010000039.1 | 99.126 | 801 | 7 | 0 | 897 | 97 |
| DASGGT010000025.1 | 99.126 | 801 | 7 | 0 | 7703 | 8503 |
| DASGGS010000015.1 | 99.126 | 801 | 7 | 0 | 7708 | 8508 |
| DASGGL010000010.1 | 99.126 | 801 | 7 | 0 | 7703 | 8503 |
| DASGGK010000010.1 | 99.126 | 801 | 7 | 0 | 7703 | 8503 |
| DASGGI010000011.1 | 99.126 | 801 | 7 | 0 | 7703 | 8503 |
| DASGGC010000021.1 | 99.126 | 801 | 7 | 0 | 7703 | 8503 |
| DASGFR010000028.1 | 99.126 | 801 | 7 | 0 | 7703 | 8503 |
| DASGEK010000039.1 | 99.126 | 801 | 7 | 0 | 897 | 97 |
| DASGEB010000016.1 | 99.126 | 801 | 7 | 0 | 46650 | 45850 |
| DASGDZ010000001.1 | 99.126 | 801 | 7 | 0 | 31141 | 31941 |
| DASGDY010000001.1 | 99.126 | 801 | 7 | 0 | 42612 | 41812 |
| DASGDV010000036.1 | 99.126 | 801 | 7 | 0 | 896 | 96 |
| DASGDU010000011.1 | 99.126 | 801 | 7 | 0 | 897 | 97 |
| DASGDR010000001.1 | 99.126 | 801 | 7 | 0 | 31141 | 31941 |
| DASGDQ010000001.1 | 99.126 | 801 | 7 | 0 | 31141 | 31941 |
| DASGDN010000006.1 | 99.126 | 801 | 7 | 0 | 65253 | 64453 |
| DASGBQ010000014.1 | 99.126 | 801 | 7 | 0 | 20971 | 21771 |
| DASGBR010000002.1 | 99.126 | 801 | 7 | 0 | 7390 | 8190 |
| DASGBP010000005.1 | 99.126 | 801 | 7 | 0 | 85035 | 85835 |
| DASGBM010000010.1 | 99.126 | 801 | 7 | 0 | 40965 | 41765 |
| DASGBD010000024.1 | 99.126 | 801 | 7 | 0 | 34796 | 33996 |
| DASGBB010000008.1 | 99.126 | 801 | 7 | 0 | 48231 | 47431 |
| DASGBC010000006.1 | 99.126 | 801 | 7 | 0 | 48145 | 47345 |
| DASFZV010000033.1 | 99.126 | 801 | 7 | 0 | 21068 | 20268 |
| DASFZK010000001.1 | 99.126 | 801 | 7 | 0 | 251620 | 252420 |
| DASFZD010000005.1 | 99.126 | 801 | 7 | 0 | 98705 | 99505 |
| DASFYX010000023.1 | 99.126 | 801 | 7 | 0 | 34797 | 33997 |
| DASFYY010000002.1 | 99.126 | 801 | 7 | 0 | 90916 | 91716 |
| DASFYV010000031.1 | 99.126 | 801 | 7 | 0 | 897 | 97 |
| DASFYW010000001.1 | 99.126 | 801 | 7 | 0 | 233770 | 232970 |
| DASFYS010000005.1 | 99.126 | 801 | 7 | 0 | 58199 | 57399 |
| DASFYC010000067.1 | 99.126 | 801 | 7 | 0 | 10718 | 9918 |
| DASFYA010000004.1 | 99.126 | 801 | 7 | 0 | 53543 | 52743 |
| DASFXY010000013.1 | 99.126 | 801 | 7 | 0 | 30233 | 29433 |
| DASFXD010000001.1 | 99.126 | 801 | 7 | 0 | 203713 | 202913 |
| DASFWI010000005.1 | 99.126 | 801 | 7 | 0 | 34728 | 33928 |
| DASFVP010000010.1 | 99.126 | 801 | 7 | 0 | 7688 | 6888 |
| DASFUD010000011.1 | 99.126 | 801 | 7 | 0 | 20925 | 20125 |
| DASFTR010000023.1 | 99.126 | 801 | 7 | 0 | 7703 | 8503 |
| DASFRY010000015.1 | 99.126 | 801 | 7 | 0 | 7815 | 8615 |
| DASFRF010000008.1 | 99.126 | 801 | 7 | 0 | 7703 | 8503 |
| DASFPN010000006.1 | 99.126 | 801 | 7 | 0 | 7703 | 8503 |
| DASFOJ010000014.1 | 99.126 | 801 | 7 | 0 | 8471 | 9271 |
| DASFNQ010000013.1 | 99.126 | 801 | 7 | 0 | 7708 | 8508 |
| DASFNK010000008.1 | 99.126 | 801 | 7 | 0 | 7708 | 8508 |
| DASFMC010000009.1 | 99.126 | 801 | 7 | 0 | 7708 | 8508 |
| DASFLH010000014.1 | 99.126 | 801 | 7 | 0 | 7708 | 8508 |
| DASFKS010000041.1 | 99.126 | 801 | 7 | 0 | 15732 | 14932 |
| DASFKR010000017.1 | 99.126 | 801 | 7 | 0 | 11875 | 11075 |
| DASFKK010000012.1 | 99.126 | 801 | 7 | 0 | 7705 | 8505 |
| DASFKE010000015.1 | 99.126 | 801 | 7 | 0 | 7707 | 8507 |
| DASFJP010000009.1 | 99.126 | 801 | 7 | 0 | 7708 | 8508 |
| DASFJE010000001.1 | 99.126 | 801 | 7 | 0 | 220102 | 219302 |
| DASFJA010000013.1 | 99.126 | 801 | 7 | 0 | 7708 | 8508 |
| DASFIJ010000002.1 | 99.126 | 801 | 7 | 0 | 7707 | 8507 |
| DASFHP010000006.1 | 99.126 | 801 | 7 | 0 | 47297 | 48097 |
| DASFGL010000011.1 | 99.126 | 801 | 7 | 0 | 7703 | 8503 |
| DASFGC010000010.1 | 99.126 | 801 | 7 | 0 | 7703 | 8503 |
| DASFGE010000012.1 | 99.126 | 801 | 7 | 0 | 7707 | 8507 |
| DASFFX010000008.1 | 99.126 | 801 | 7 | 0 | 7707 | 8507 |
| DASFFV010000023.1 | 99.126 | 801 | 7 | 0 | 20804 | 20004 |
| DASFFU010000005.1 | 99.126 | 801 | 7 | 0 | 105804 | 105004 |
| DASFFT010000041.1 | 99.126 | 801 | 7 | 0 | 20833 | 20033 |
| DASFFQ010000018.1 | 99.126 | 801 | 7 | 0 | 21093 | 20293 |
| DASFEU010000010.1 | 99.126 | 801 | 7 | 0 | 4661 | 5461 |
| DASFED010000013.1 | 99.126 | 801 | 7 | 0 | 2548 | 3348 |
| DASFEC010000014.1 | 99.126 | 801 | 7 | 0 | 2551 | 3351 |
| DASFDV010000009.1 | 99.126 | 801 | 7 | 0 | 7708 | 8508 |
| DASFDP010000016.1 | 99.126 | 801 | 7 | 0 | 20901 | 20101 |
| DASFDS010000017.1 | 99.126 | 801 | 7 | 0 | 20901 | 20101 |
| DASFBG010000019.1 | 99.126 | 801 | 7 | 0 | 20901 | 20101 |
| DASEOT010000012.1 | 99.126 | 801 | 7 | 0 | 7703 | 8503 |
| DASEOP010000003.1 | 99.126 | 801 | 7 | 0 | 65647 | 64847 |
| DASEOC010000001.1 | 99.126 | 801 | 7 | 0 | 135953 | 135153 |
| DASENY010000006.1 | 99.126 | 801 | 7 | 0 | 28786 | 27986 |
| DASEHK010000027.1 | 99.126 | 801 | 7 | 0 | 5524 | 6324 |
| DASEGR010000035.1 | 99.126 | 801 | 7 | 0 | 893 | 93 |
| DASEFZ010000018.1 | 99.126 | 801 | 7 | 0 | 11700 | 10900 |
| DASEFX010000005.1 | 99.126 | 801 | 7 | 0 | 35280 | 34480 |
| DASEFW010000030.1 | 99.126 | 801 | 7 | 0 | 14908 | 14108 |
| DASEFP010000025.1 | 99.126 | 801 | 7 | 0 | 34787 | 33987 |
| DASEFK010000012.1 | 99.126 | 801 | 7 | 0 | 70873 | 70073 |
| DASEFI010000003.1 | 99.126 | 801 | 7 | 0 | 34788 | 33988 |
| DASEEJ010000024.1 | 99.126 | 801 | 7 | 0 | 34791 | 33991 |
| DASEEC010000003.1 | 99.126 | 801 | 7 | 0 | 199791 | 200591 |
| DASEDV010000012.1 | 99.126 | 801 | 7 | 0 | 15954 | 15154 |
| DASEDI010000015.1 | 99.126 | 801 | 7 | 0 | 28538 | 27738 |
| DASEDH010000001.1 | 99.126 | 801 | 7 | 0 | 29848 | 30648 |
| DASECW010000015.1 | 99.126 | 801 | 7 | 0 | 7703 | 8503 |
| DASECL010000022.1 | 99.126 | 801 | 7 | 0 | 7703 | 8503 |
| DASEBZ010000001.1 | 99.126 | 801 | 7 | 0 | 82896 | 83696 |
| DASEBS010000012.1 | 99.126 | 801 | 7 | 0 | 7704 | 8504 |
| DASEBQ010000001.1 | 99.126 | 801 | 7 | 0 | 88159 | 88959 |
| DASCME010000026.1 | 99.126 | 801 | 7 | 0 | 9946 | 10746 |
| DASCLY010000008.1 | 99.126 | 801 | 7 | 0 | 10615 | 9815 |
| DASCLU010000012.1 | 99.126 | 801 | 7 | 0 | 7705 | 8505 |
| DARZUF010000008.1 | 99.126 | 801 | 7 | 0 | 35666 | 34866 |
| DARZTS010000011.1 | 99.126 | 801 | 7 | 0 | 7707 | 8507 |
| DARZTH010000010.1 | 99.126 | 801 | 7 | 0 | 7708 | 8508 |
| DARZTD010000012.1 | 99.126 | 801 | 7 | 0 | 7700 | 8500 |
| DARZSN010000025.1 | 99.126 | 801 | 7 | 0 | 7703 | 8503 |
| DARZSH010000009.1 | 99.126 | 801 | 7 | 0 | 7703 | 8503 |
| DARZSG010000002.1 | 99.126 | 801 | 7 | 0 | 35709 | 36509 |
| DARZSA010000011.1 | 99.126 | 801 | 7 | 0 | 65114 | 64314 |
| DARZRU010000001.1 | 99.126 | 801 | 7 | 0 | 226420 | 227220 |
| DARZRP010000011.1 | 99.126 | 801 | 7 | 0 | 7704 | 8504 |
| DARZQW010000011.1 | 99.126 | 801 | 7 | 0 | 7705 | 8505 |
| DARZQU010000001.1 | 99.126 | 801 | 7 | 0 | 29858 | 30658 |
| DARZQS010000001.1 | 99.126 | 801 | 7 | 0 | 29858 | 30658 |
| DARZQN010000010.1 | 99.126 | 801 | 7 | 0 | 7703 | 8503 |
| DARZQK010000012.1 | 99.126 | 801 | 7 | 0 | 8584 | 9384 |
| DARZQI010000011.1 | 99.126 | 801 | 7 | 0 | 7703 | 8503 |
| DARZPS010000011.1 | 99.126 | 801 | 7 | 0 | 46971 | 47771 |
| DARZPN010000013.1 | 99.126 | 801 | 7 | 0 | 7704 | 8504 |
| DARZNT010000001.1 | 99.126 | 801 | 7 | 0 | 273356 | 274156 |
| DARZNI010000002.1 | 99.126 | 801 | 7 | 0 | 166991 | 167791 |
| DARZNG010000015.1 | 99.126 | 801 | 7 | 0 | 21958 | 21158 |
| DARZNC010000002.1 | 99.126 | 801 | 7 | 0 | 34788 | 33988 |
| DARZMX010000001.1 | 99.126 | 801 | 7 | 0 | 1383 | 2183 |
| DARZMT010000001.1 | 99.126 | 801 | 7 | 0 | 1383 | 2183 |
| DARZMM010000004.1 | 99.126 | 801 | 7 | 0 | 897 | 97 |
| DARZML010000018.1 | 99.126 | 801 | 7 | 0 | 52795 | 51995 |
| DARZLU010000010.1 | 99.126 | 801 | 7 | 0 | 65114 | 64314 |
| DARZKR010000010.1 | 99.126 | 801 | 7 | 0 | 20182 | 19382 |
| DARZKM010000024.1 | 99.126 | 801 | 7 | 0 | 11798 | 10998 |
| DARZKI010000010.1 | 99.126 | 801 | 7 | 0 | 7708 | 8508 |
| DARZJT010000025.1 | 99.126 | 801 | 7 | 0 | 7864 | 8664 |
| DARZJR010000011.1 | 99.126 | 801 | 7 | 0 | 7703 | 8503 |
| DARZIK010000011.1 | 99.126 | 801 | 7 | 0 | 7703 | 8503 |
| DARZIL010000035.1 | 99.126 | 801 | 7 | 0 | 7703 | 8503 |
| DARZIB010000002.1 | 99.126 | 801 | 7 | 0 | 35709 | 36509 |
| DARZHR010000001.1 | 99.126 | 801 | 7 | 0 | 209183 | 208383 |
| DARZHH010000025.1 | 99.126 | 801 | 7 | 0 | 7707 | 8507 |
| DARZHD010000002.1 | 99.126 | 801 | 7 | 0 | 29863 | 30663 |
| DARZGY010000002.1 | 99.126 | 801 | 7 | 0 | 29863 | 30663 |
| DARZGM010000036.1 | 99.126 | 801 | 7 | 0 | 13918 | 13118 |
| DARZGE010000013.1 | 99.126 | 801 | 7 | 0 | 7703 | 8503 |
| DARZFZ010000001.1 | 99.126 | 801 | 7 | 0 | 34658 | 35458 |
| DARZFK010000001.1 | 99.126 | 801 | 7 | 0 | 273954 | 274754 |
| DARZFL010000001.1 | 99.126 | 801 | 7 | 0 | 81490 | 82290 |
| DARZFG010000017.1 | 99.126 | 801 | 7 | 0 | 20901 | 20101 |
| DARZDQ010000012.1 | 99.126 | 801 | 7 | 0 | 7705 | 8505 |
| DARZDL010000001.1 | 99.126 | 801 | 7 | 0 | 307427 | 308227 |
| DARZDH010000006.1 | 99.126 | 801 | 7 | 0 | 46791 | 47591 |
| DARZCL010000014.1 | 99.126 | 801 | 7 | 0 | 15973 | 15173 |
| DARZCF010000029.1 | 99.126 | 801 | 7 | 0 | 15676 | 14876 |
| DARZBW010000023.1 | 99.126 | 801 | 7 | 0 | 7707 | 8507 |
| DARZBY010000008.1 | 99.126 | 801 | 7 | 0 | 60918 | 60118 |
| DARZBT010000019.1 | 99.126 | 801 | 7 | 0 | 7704 | 8504 |
| DARZBP010000011.1 | 99.126 | 801 | 7 | 0 | 7709 | 8509 |
| DARZBK010000034.1 | 99.126 | 801 | 7 | 0 | 7703 | 8503 |
| DARZBL010000012.1 | 99.126 | 801 | 7 | 0 | 7707 | 8507 |
| DARZBB010000020.1 | 99.126 | 801 | 7 | 0 | 14906 | 14106 |
| DARZAX010000009.1 | 99.126 | 801 | 7 | 0 | 7704 | 8504 |
| DARZAC010000005.1 | 99.126 | 801 | 7 | 0 | 47007 | 47807 |
| DARZAB010000025.1 | 99.126 | 801 | 7 | 0 | 7703 | 8503 |
| DARYZU010000004.1 | 99.126 | 801 | 7 | 0 | 166673 | 165873 |
| DARYYS010000025.1 | 99.126 | 801 | 7 | 0 | 7703 | 8503 |
| DARYYQ010000001.1 | 99.126 | 801 | 7 | 0 | 2703 | 1903 |
| DARYYL010000004.1 | 99.126 | 801 | 7 | 0 | 113441 | 112641 |
| DARYYK010000004.1 | 99.126 | 801 | 7 | 0 | 113441 | 112641 |
| JAWWZK010000001.1 | 99.126 | 801 | 7 | 0 | 1541 | 2341 |
| JAWWZC010000013.1 | 99.126 | 801 | 7 | 0 | 65193 | 64393 |
| JAUTGX010000005.1 | 99.126 | 801 | 7 | 0 | 1564 | 2364 |
| JAUTGH010000012.1 | 99.126 | 801 | 7 | 0 | 7815 | 8615 |
| JAUTGG010000012.1 | 99.126 | 801 | 7 | 0 | 7815 | 8615 |
| JAUTFZ010000012.1 | 99.126 | 801 | 7 | 0 | 7815 | 8615 |
| JAUTFL010000001.1 | 99.126 | 801 | 7 | 0 | 148773 | 147973 |
| JAUTEO010000001.1 | 99.126 | 801 | 7 | 0 | 163879 | 163079 |
| JAUTEP010000001.1 | 99.126 | 801 | 7 | 0 | 3983 | 4783 |
| JAUTEQ010000001.1 | 99.126 | 801 | 7 | 0 | 163879 | 163079 |
| JAVMBK010000040.1 | 99.126 | 801 | 7 | 0 | 1351 | 2151 |
| JAVLUW010000001.1 | 99.126 | 801 | 7 | 0 | 127712 | 128512 |
| JARATE010000010.1 | 99.126 | 801 | 7 | 0 | 7831 | 8631 |
| JARATD010000021.1 | 99.126 | 801 | 7 | 0 | 7831 | 8631 |
| JANFME010000005.1 | 99.126 | 801 | 7 | 0 | 57289 | 58089 |
| JARBFO010000001.1 | 99.126 | 801 | 7 | 0 | 60255 | 59455 |
| CP102148.1 | 99.126 | 801 | 7 | 0 | 852918 | 852118 |
| JAMWES010000010.1 | 99.126 | 801 | 7 | 0 | 65193 | 64393 |
| CP100432.1 | 99.126 | 801 | 7 | 0 | 899528 | 898728 |
| JAASEX010000010.1 | 99.126 | 801 | 7 | 0 | 55920 | 55120 |
| JAASEO010000018.1 | 99.126 | 801 | 7 | 0 | 50429 | 49629 |
| JAASEJ010000026.1 | 99.126 | 801 | 7 | 0 | 8185 | 8985 |
| JAASEB010000012.1 | 99.126 | 801 | 7 | 0 | 65530 | 64730 |
| JAASEA010000015.1 | 99.126 | 801 | 7 | 0 | 55821 | 55021 |
| JAASDJ010000014.1 | 99.126 | 801 | 7 | 0 | 8179 | 8979 |
| JAASCT010000004.1 | 99.126 | 801 | 7 | 0 | 8183 | 8983 |
| JAASCR010000017.1 | 99.126 | 801 | 7 | 0 | 129830 | 129030 |
| JAASCL010000013.1 | 99.126 | 801 | 7 | 0 | 55617 | 54817 |
| JAASCI010000018.1 | 99.126 | 801 | 7 | 0 | 8168 | 8968 |
| JAASBM010000011.1 | 99.126 | 801 | 7 | 0 | 8131 | 8931 |
| JAASBK010000017.1 | 99.126 | 801 | 7 | 0 | 10528 | 9728 |
| JAASBD010000022.1 | 99.126 | 801 | 7 | 0 | 65634 | 64834 |
| JAKTBO010000011.1 | 99.126 | 801 | 7 | 0 | 65194 | 64394 |
| JAKOFZ010000023.1 | 99.126 | 801 | 7 | 0 | 7836 | 8636 |
| JAKOFX010000014.1 | 99.126 | 801 | 7 | 0 | 7849 | 8649 |
| JAKOFT010000012.1 | 99.126 | 801 | 7 | 0 | 7836 | 8636 |
| JAKOFQ010000014.1 | 99.126 | 801 | 7 | 0 | 7836 | 8636 |
| JAKOFP010000014.1 | 99.126 | 801 | 7 | 0 | 7836 | 8636 |
| JAKOFN010000015.1 | 99.126 | 801 | 7 | 0 | 7836 | 8636 |
| JAKOFO010000014.1 | 99.126 | 801 | 7 | 0 | 55283 | 54483 |
| JAKOFM010000016.1 | 99.126 | 801 | 7 | 0 | 7836 | 8636 |
| JAKOFL010000017.1 | 99.126 | 801 | 7 | 0 | 7836 | 8636 |
| JAKOFG010000010.1 | 99.126 | 801 | 7 | 0 | 7943 | 8743 |
| JAKOFK010000013.1 | 99.126 | 801 | 7 | 0 | 65330 | 64530 |
| JAKOFI010000015.1 | 99.126 | 801 | 7 | 0 | 7836 | 8636 |
| JAKOFJ010000012.1 | 99.126 | 801 | 7 | 0 | 65330 | 64530 |
| JAKOFF010000013.1 | 99.126 | 801 | 7 | 0 | 7836 | 8636 |
| JAKOFE010000010.1 | 99.126 | 801 | 7 | 0 | 7943 | 8743 |
| JAKOFD010000013.1 | 99.126 | 801 | 7 | 0 | 7836 | 8636 |
| JAKOFC010000014.1 | 99.126 | 801 | 7 | 0 | 7836 | 8636 |
| JAKOFA010000013.1 | 99.126 | 801 | 7 | 0 | 7836 | 8636 |
| JAFLMP010000012.1 | 99.126 | 801 | 7 | 0 | 7836 | 8636 |
| JAFLMR010000023.1 | 99.126 | 801 | 7 | 0 | 12863 | 12063 |
| JAFLMQ010000012.1 | 99.126 | 801 | 7 | 0 | 7836 | 8636 |
| JAFLMO010000011.1 | 99.126 | 801 | 7 | 0 | 7836 | 8636 |
| JAFHDP010000012.1 | 99.126 | 801 | 7 | 0 | 7836 | 8636 |
| JAFHDO010000012.1 | 99.126 | 801 | 7 | 0 | 7836 | 8636 |
| JAFHDJ010000010.1 | 99.126 | 801 | 7 | 0 | 7836 | 8636 |
| JAFHDK010000012.1 | 99.126 | 801 | 7 | 0 | 7836 | 8636 |
| CP079193.1 | 99.126 | 801 | 7 | 0 | 861758 | 860958 |
| CP076517.1 | 99.126 | 801 | 7 | 0 | 906048 | 905248 |
| VZNM01000011.1 | 99.126 | 801 | 7 | 0 | 65218 | 64418 |
| WACE01000016.1 | 99.126 | 801 | 7 | 0 | 7709 | 8509 |
| CP082778.1 | 99.126 | 801 | 7 | 0 | 869213 | 868413 |
| CP068708.1 | 99.126 | 801 | 7 | 0 | 1113788 | 1114588 |
| JABTYX010000012.1 | 99.126 | 801 | 7 | 0 | 7799 | 8599 |
| JABLFD010000012.1 | 99.126 | 801 | 7 | 0 | 7782 | 8582 |
| JABLFN010000014.1 | 99.126 | 801 | 7 | 0 | 65187 | 64387 |
| JABLGP010000012.1 | 99.126 | 801 | 7 | 0 | 65847 | 65047 |
| JABLHM010000024.1 | 99.126 | 801 | 7 | 0 | 7832 | 8632 |
| JABLHZ010000012.1 | 99.126 | 801 | 7 | 0 | 65443 | 64643 |
| JABLIA010000033.1 | 99.126 | 801 | 7 | 0 | 7829 | 8629 |
| JABLIW010000002.1 | 99.126 | 801 | 7 | 0 | 222930 | 223730 |
| JABLIV010000013.1 | 99.126 | 801 | 7 | 0 | 66429 | 65629 |
| JABLIX010000011.1 | 99.126 | 801 | 7 | 0 | 65239 | 64439 |
| JABLJD010000013.1 | 99.126 | 801 | 7 | 0 | 66430 | 65630 |
| JABLJG010000010.1 | 99.126 | 801 | 7 | 0 | 65241 | 64441 |
| JABLGX010000024.1 | 99.126 | 801 | 7 | 0 | 1021 | 221 |
| JABLJM010000019.1 | 99.126 | 801 | 7 | 0 | 15176 | 14376 |
| JABLHS010000002.1 | 99.126 | 801 | 7 | 0 | 218489 | 219289 |
| JABLIM010000006.1 | 99.126 | 801 | 7 | 0 | 1448 | 2248 |
| JABLJE010000011.1 | 99.126 | 801 | 7 | 0 | 65238 | 64438 |
| JABLHD010000012.1 | 99.126 | 801 | 7 | 0 | 7832 | 8632 |
| JABLHT010000003.1 | 99.126 | 801 | 7 | 0 | 242018 | 242818 |
| JABLJY010000027.1 | 99.126 | 801 | 7 | 0 | 7832 | 8632 |
| JABLLA010000001.1 | 99.126 | 801 | 7 | 0 | 31445 | 32245 |
| JABLLH010000002.1 | 99.126 | 801 | 7 | 0 | 31591 | 32391 |
| JABLLI010000001.1 | 99.126 | 801 | 7 | 0 | 106470 | 107270 |
| JABLLL010000001.1 | 99.126 | 801 | 7 | 0 | 254093 | 254893 |
| JABLMQ010000033.1 | 99.126 | 801 | 7 | 0 | 995 | 195 |
| JABLMT010000001.1 | 99.126 | 801 | 7 | 0 | 155408 | 154608 |
| JABLMR010000001.1 | 99.126 | 801 | 7 | 0 | 555037 | 555837 |
| JABLMS010000031.1 | 99.126 | 801 | 7 | 0 | 1422 | 2222 |
| JABLMU010000001.1 | 99.126 | 801 | 7 | 0 | 31935 | 32735 |
| JABLMW010000002.1 | 99.126 | 801 | 7 | 0 | 199491 | 200291 |
| JABLLB010000001.1 | 99.126 | 801 | 7 | 0 | 107647 | 108447 |
| JABLNQ010000001.1 | 99.126 | 801 | 7 | 0 | 31775 | 32575 |
| JABLLC010000001.1 | 99.126 | 801 | 7 | 0 | 254088 | 254888 |
| JABLNR010000002.1 | 99.126 | 801 | 7 | 0 | 92338 | 93138 |
| JABLNT010000032.1 | 99.126 | 801 | 7 | 0 | 1422 | 2222 |
| JABLLW010000004.1 | 99.126 | 801 | 7 | 0 | 91562 | 92362 |
| JABLOA010000001.1 | 99.126 | 801 | 7 | 0 | 31507 | 32307 |
| JABLOB010000001.1 | 99.126 | 801 | 7 | 0 | 31542 | 32342 |
| JABLOF010000035.1 | 99.126 | 801 | 7 | 0 | 1422 | 2222 |
| JABLMH010000018.1 | 99.126 | 801 | 7 | 0 | 46750 | 45950 |
| JABLND010000001.1 | 99.126 | 801 | 7 | 0 | 269839 | 269039 |
| JABLOW010000016.1 | 99.126 | 801 | 7 | 0 | 52715 | 51915 |
| JABLNX010000013.1 | 99.126 | 801 | 7 | 0 | 2312 | 1512 |
| JABLPM010000002.1 | 99.126 | 801 | 7 | 0 | 269876 | 269076 |
| JABLNW010000033.1 | 99.126 | 801 | 7 | 0 | 1422 | 2222 |
| JABKST010000009.1 | 99.126 | 801 | 7 | 0 | 7826 | 8626 |
| JABKSZ010000012.1 | 99.126 | 801 | 7 | 0 | 65241 | 64441 |
| JABKTA010000022.1 | 99.126 | 801 | 7 | 0 | 2780 | 1980 |
| JABKTB010000008.1 | 99.126 | 801 | 7 | 0 | 133331 | 132531 |
| JABKTE010000011.1 | 99.126 | 801 | 7 | 0 | 65895 | 65095 |
| JABKUG010000011.1 | 99.126 | 801 | 7 | 0 | 65240 | 64440 |
| JABKUA010000014.1 | 99.126 | 801 | 7 | 0 | 65241 | 64441 |
| JABKTZ010000007.1 | 99.126 | 801 | 7 | 0 | 91739 | 92539 |
| JABKYA010000012.1 | 99.126 | 801 | 7 | 0 | 66419 | 65619 |
| JABKYB010000014.1 | 99.126 | 801 | 7 | 0 | 7832 | 8632 |
| JABKYD010000019.1 | 99.126 | 801 | 7 | 0 | 34658 | 33858 |
| JABKYC010000013.1 | 99.126 | 801 | 7 | 0 | 66428 | 65628 |
| JABKUR010000010.1 | 99.126 | 801 | 7 | 0 | 65240 | 64440 |
| JABKYE010000004.1 | 99.126 | 801 | 7 | 0 | 198834 | 198034 |
| JABKYF010000012.1 | 99.126 | 801 | 7 | 0 | 66428 | 65628 |
| JABKYG010000012.1 | 99.126 | 801 | 7 | 0 | 7831 | 8631 |
| JABKYK010000011.1 | 99.126 | 801 | 7 | 0 | 65241 | 64441 |
| JABKVZ010000005.1 | 99.126 | 801 | 7 | 0 | 91739 | 92539 |
| JABKZP010000038.1 | 99.126 | 801 | 7 | 0 | 4720 | 5520 |
| JABKZY010000011.1 | 99.126 | 801 | 7 | 0 | 971 | 171 |
| JABKZZ010000003.1 | 99.126 | 801 | 7 | 0 | 69160 | 68360 |
| JABKWP010000002.1 | 99.126 | 801 | 7 | 0 | 60712 | 59912 |
| JABKZX010000002.1 | 99.126 | 801 | 7 | 0 | 163297 | 164097 |
| JABLAE010000011.1 | 99.126 | 801 | 7 | 0 | 12664 | 13464 |
| JABLAD010000034.1 | 99.126 | 801 | 7 | 0 | 1397 | 2197 |
| JABLAQ010000005.1 | 99.126 | 801 | 7 | 0 | 68658 | 67858 |
| JABLAT010000012.1 | 99.126 | 801 | 7 | 0 | 65191 | 64391 |
| JABLBN010000001.1 | 99.126 | 801 | 7 | 0 | 132808 | 132008 |
| JABLBY010000017.1 | 99.126 | 801 | 7 | 0 | 7781 | 8581 |
| JABLBZ010000027.1 | 99.126 | 801 | 7 | 0 | 15122 | 14322 |
| JABLCB010000003.1 | 99.126 | 801 | 7 | 0 | 7782 | 8582 |
| JABLCG010000022.1 | 99.126 | 801 | 7 | 0 | 6103 | 6903 |
| JABLCH010000002.1 | 99.126 | 801 | 7 | 0 | 65188 | 64388 |
| JABLBW010000001.1 | 99.126 | 801 | 7 | 0 | 255406 | 256206 |
| JABLEW010000013.1 | 99.126 | 801 | 7 | 0 | 7781 | 8581 |
| JABLDA010000009.1 | 99.126 | 801 | 7 | 0 | 7773 | 8573 |
| JABLEU010000013.1 | 99.126 | 801 | 7 | 0 | 7779 | 8579 |
| JABLCL010000004.1 | 99.126 | 801 | 7 | 0 | 70458 | 69658 |
| JABLDW010000011.1 | 99.126 | 801 | 7 | 0 | 7775 | 8575 |
| JABLEN010000028.1 | 99.126 | 801 | 7 | 0 | 7781 | 8581 |
| JAAVJW010000025.1 | 99.126 | 801 | 7 | 0 | 15487 | 16287 |
| JAAVJV010000008.1 | 99.126 | 801 | 7 | 0 | 60478 | 59678 |
| VIFF01000002.1 | 99.126 | 801 | 7 | 0 | 1617128 | 1617928 |
| VIEL01000002.1 | 99.126 | 801 | 7 | 0 | 342153 | 342953 |
| RRZP01000019.1 | 99.126 | 801 | 7 | 0 | 7913 | 8713 |
| RRZQ01000007.1 | 99.126 | 801 | 7 | 0 | 15738 | 16538 |
| PONZ01000490.1 | 99.126 | 801 | 7 | 0 | 2807 | 2007 |
| POKM01000118.1 | 99.126 | 801 | 7 | 0 | 29938 | 30738 |
| PONL01000021.1 | 99.126 | 801 | 7 | 0 | 55782 | 56582 |
| PONK01000081.1 | 99.126 | 801 | 7 | 0 | 55782 | 56582 |
| POMV01000100.1 | 99.126 | 801 | 7 | 0 | 4975 | 5775 |
| POJK01000088.1 | 99.126 | 801 | 7 | 0 | 3337 | 2537 |
| POIO01000311.1 | 99.126 | 801 | 7 | 0 | 52457 | 51657 |
| CP025043.1 | 99.126 | 801 | 7 | 0 | 1078342 | 1077542 |
| CEIW01000027.1 | 99.126 | 801 | 7 | 0 | 65173 | 64373 |
| CEIS01000041.1 | 99.126 | 801 | 7 | 0 | 65174 | 64374 |
| CEDP01000028.1 | 99.126 | 801 | 7 | 0 | 7764 | 8564 |
| CEIY01000015.1 | 99.126 | 801 | 7 | 0 | 65172 | 64372 |
| CEDE01000055.1 | 99.126 | 801 | 7 | 0 | 65166 | 64366 |
| CEGY01000010.1 | 99.126 | 801 | 7 | 0 | 65172 | 64372 |
| CEKO01000011.1 | 99.126 | 801 | 7 | 0 | 7760 | 8560 |
| CEEV01000067.1 | 99.126 | 801 | 7 | 0 | 61275 | 60475 |
| CEDO01000047.1 | 99.126 | 801 | 7 | 0 | 1438 | 2238 |
| CECY01000098.1 | 99.126 | 801 | 7 | 0 | 20416 | 19616 |
| CEEH01000046.1 | 99.126 | 801 | 7 | 0 | 50839 | 50039 |
| CEIA01000010.1 | 99.126 | 801 | 7 | 0 | 65176 | 64376 |
| CEHO01000049.1 | 99.126 | 801 | 7 | 0 | 15099 | 14299 |
| CEHV01000072.1 | 99.126 | 801 | 7 | 0 | 1386 | 2186 |
| CEEQ01000020.1 | 99.126 | 801 | 7 | 0 | 65180 | 64380 |
| CEDC01000010.1 | 99.126 | 801 | 7 | 0 | 954 | 154 |
| CEER01000021.1 | 99.126 | 801 | 7 | 0 | 67534 | 66734 |
| CEGX01000020.1 | 99.126 | 801 | 7 | 0 | 7766 | 8566 |
| CEJO01000013.1 | 99.126 | 801 | 7 | 0 | 30242 | 29442 |
| CEEC01000014.1 | 99.126 | 801 | 7 | 0 | 65174 | 64374 |
| CEEN01000080.1 | 99.126 | 801 | 7 | 0 | 20202 | 19402 |
| CEDJ01000039.1 | 99.126 | 801 | 7 | 0 | 81313 | 80513 |
| CEEZ01000028.1 | 99.126 | 801 | 7 | 0 | 68313 | 67513 |
| CECR01000027.1 | 99.126 | 801 | 7 | 0 | 14320 | 13520 |
| CP006246.1 | 99.126 | 801 | 7 | 0 | 899450 | 898650 |
| ALME01000043.1 | 99.126 | 801 | 7 | 0 | 54661 | 53861 |
| ALMC01000008.1 | 99.126 | 801 | 7 | 0 | 7776 | 8576 |
| ALLY01000004.1 | 99.126 | 801 | 7 | 0 | 60670 | 61470 |
| ALLT01000034.1 | 99.126 | 801 | 7 | 0 | 65181 | 64381 |
| ALLP01000101.1 | 99.126 | 801 | 7 | 0 | 62952 | 63752 |
| ALLH01000048.1 | 99.126 | 801 | 7 | 0 | 279983 | 280783 |
| ALKX01000104.1 | 99.126 | 801 | 7 | 0 | 70845 | 70045 |
| ALKV01000002.1 | 99.126 | 801 | 7 | 0 | 65189 | 64389 |
| VIEP01000001.1 | 99.126 | 801 | 5 | 1 | 124717 | 125515 |
| NZ_JAASBA010000012.1 | 98.876 | 801 | 9 | 0 | 65679 | 64879 |
| JAASBA010000012.1 | 98.876 | 801 | 9 | 0 | 65679 | 64879 |
| NZ_FIGR01000001.1 | 99.001 | 801 | 8 | 0 | 130701 | 131501 |
| NZ_FIGB01000001.1 | 99.001 | 801 | 8 | 0 | 337175 | 337975 |
| NZ_FIFS01000002.1 | 99.001 | 801 | 8 | 0 | 31066 | 31866 |
| NZ_FIIR01000001.1 | 99.001 | 801 | 8 | 0 | 34831 | 34031 |
| NZ_FIIO01000001.1 | 99.001 | 801 | 8 | 0 | 116662 | 115862 |
| NZ_FIFQ01000011.1 | 99.001 | 801 | 8 | 0 | 65325 | 64525 |
| NZ_FIIS01000001.1 | 99.001 | 801 | 8 | 0 | 215215 | 216015 |
| NZ_FIIG01000005.1 | 99.001 | 801 | 8 | 0 | 97001 | 97801 |
| NZ_FIIC01000004.1 | 99.001 | 801 | 8 | 0 | 14317 | 13517 |
| NZ_FIGP01000008.1 | 99.001 | 801 | 8 | 0 | 62736 | 61936 |
| NZ_FIGV01000013.1 | 99.001 | 801 | 8 | 0 | 47880 | 48680 |
| NZ_FIMB01000001.1 | 99.001 | 801 | 8 | 0 | 62929 | 63729 |
| NZ_CZFY01000003.1 | 99.001 | 801 | 8 | 0 | 52031 | 52831 |
| NZ_CZFT01000004.1 | 99.001 | 801 | 8 | 0 | 65194 | 64394 |
| NZ_CZGT01000012.1 | 99.001 | 801 | 8 | 0 | 30640 | 31440 |
| NZ_CZFW01000027.1 | 99.001 | 801 | 8 | 0 | 213167 | 213967 |
| NZ_CZGJ01000044.1 | 99.001 | 801 | 8 | 0 | 212279 | 213079 |
| NZ_CZFR01000011.1 | 99.001 | 801 | 8 | 0 | 7786 | 8586 |
| NZ_JBFNWU010000009.1 | 99.001 | 801 | 8 | 0 | 88475 | 89275 |
| NZ_JBBMMN010000019.1 | 99.001 | 801 | 8 | 0 | 34951 | 35751 |
| NZ_JBBMMM010000019.1 | 99.001 | 801 | 8 | 0 | 34951 | 35751 |
| NZ_JBBMFU010000001.1 | 99.001 | 801 | 8 | 0 | 136506 | 135706 |
| NZ_JBAPGR010000004.1 | 99.001 | 801 | 8 | 0 | 30232 | 29432 |
| NZ_JBAPCN010000014.1 | 99.001 | 801 | 8 | 0 | 35144 | 35944 |
| NZ_JBAPCM010000010.1 | 99.001 | 801 | 8 | 0 | 35067 | 35867 |
| NZ_JBAPCU010000001.1 | 99.001 | 801 | 8 | 0 | 209672 | 208872 |
| NZ_JBAPCV010000001.1 | 99.001 | 801 | 8 | 0 | 210000 | 209200 |
| NZ_JBAPCW010000011.1 | 99.001 | 801 | 8 | 0 | 35298 | 34498 |
| NZ_JBAPGS010000004.1 | 99.001 | 801 | 8 | 0 | 30230 | 29430 |
| NZ_JBAPGT010000014.1 | 99.001 | 801 | 8 | 0 | 30231 | 29431 |
| NZ_JBAPCH010000012.1 | 99.001 | 801 | 8 | 0 | 30230 | 29430 |
| NZ_JBAPCR010000001.1 | 99.001 | 801 | 8 | 0 | 210001 | 209201 |
| NZ_JBAPCE010000019.1 | 99.001 | 801 | 8 | 0 | 30231 | 29431 |
| NZ_JBAPCI010000013.1 | 99.001 | 801 | 8 | 0 | 30170 | 29370 |
| NZ_JBAPDM010000001.1 | 99.001 | 801 | 8 | 0 | 140071 | 140871 |
| NZ_JBAPGX010000018.1 | 99.001 | 801 | 8 | 0 | 30230 | 29430 |
| NZ_JBAPGV010000019.1 | 99.001 | 801 | 8 | 0 | 14733 | 15533 |
| NZ_JBAPCP010000001.1 | 99.001 | 801 | 8 | 0 | 83051 | 82251 |
| NZ_JBAPCQ010000001.1 | 99.001 | 801 | 8 | 0 | 125017 | 125817 |
| NZ_JBAPGQ010000013.1 | 99.001 | 801 | 8 | 0 | 30231 | 29431 |
| NZ_JBAPCT010000001.1 | 99.001 | 801 | 8 | 0 | 125008 | 125808 |
| NZ_JBAPGP010000017.1 | 99.001 | 801 | 8 | 0 | 18913 | 19713 |
| NZ_JBAPCD010000018.1 | 99.001 | 801 | 8 | 0 | 30232 | 29432 |
| NZ_JBAPCK010000012.1 | 99.001 | 801 | 8 | 0 | 30232 | 29432 |
| NZ_JBAPCJ010000012.1 | 99.001 | 801 | 8 | 0 | 30169 | 29369 |
| NZ_JBAPCF010000022.1 | 99.001 | 801 | 8 | 0 | 1013 | 213 |
| NZ_JBAPCL010000014.1 | 99.001 | 801 | 8 | 0 | 35066 | 35866 |
| NZ_JBAPCG010000022.1 | 99.001 | 801 | 8 | 0 | 1013 | 213 |
| NZ_JBAPCS010000001.1 | 99.001 | 801 | 8 | 0 | 209672 | 208872 |
| NZ_JBAPGW010000032.1 | 99.001 | 801 | 8 | 0 | 15800 | 15000 |
| NZ_JBAPGU010000013.1 | 99.001 | 801 | 8 | 0 | 30170 | 29370 |
| NZ_JAVIGN010000001.1 | 99.001 | 801 | 8 | 0 | 52325 | 53125 |
| NZ_JAVIGS010000001.1 | 99.001 | 801 | 8 | 0 | 52305 | 53105 |
| NZ_JAVIGV010000014.1 | 99.001 | 801 | 8 | 0 | 34605 | 33805 |
| NZ_JAVIGX010000002.1 | 99.001 | 801 | 8 | 0 | 93791 | 92991 |
| NZ_JAVIGW010000001.1 | 99.001 | 801 | 8 | 0 | 94147 | 93347 |
| NZ_JAVIHB010000001.1 | 99.001 | 801 | 8 | 0 | 52325 | 53125 |
| NZ_JAVIHA010000001.1 | 99.001 | 801 | 8 | 0 | 93954 | 93154 |
| NZ_JAVIHC010000001.1 | 99.001 | 801 | 8 | 0 | 117260 | 116460 |
| NZ_JAVIHJ010000001.1 | 99.001 | 801 | 8 | 0 | 52984 | 53784 |
| NZ_JAVIHK010000003.1 | 99.001 | 801 | 8 | 0 | 212159 | 212959 |
| NZ_JAVIHP010000001.1 | 99.001 | 801 | 8 | 0 | 52325 | 53125 |
| NZ_JAVIHT010000001.1 | 99.001 | 801 | 8 | 0 | 59081 | 58281 |
| NZ_JAVIHU010000001.1 | 99.001 | 801 | 8 | 0 | 117929 | 117129 |
| NZ_JAVIHW010000002.1 | 99.001 | 801 | 8 | 0 | 220132 | 220932 |
| NZ_JAVIHZ010000001.1 | 99.001 | 801 | 8 | 0 | 52293 | 53093 |
| NZ_JAZDUV010000001.1 | 99.001 | 801 | 8 | 0 | 212204 | 213004 |
| NZ_JAZDVT010000004.1 | 99.001 | 801 | 8 | 0 | 34657 | 33857 |
| NZ_CP141824.1 | 99.001 | 801 | 8 | 0 | 1334857 | 1335657 |
| NZ_CP139875.1 | 99.001 | 801 | 8 | 0 | 1882760 | 1883560 |
| NZ_JAXKWP010000001.1 | 99.001 | 801 | 8 | 0 | 629426 | 630226 |
| NZ_JAUTHW010000001.1 | 99.001 | 801 | 8 | 0 | 54600 | 55400 |
| NZ_CP134474.1 | 99.001 | 801 | 8 | 0 | 1117275 | 1118075 |
| NZ_JARASW010000001.1 | 99.001 | 801 | 8 | 0 | 35676 | 34876 |
| NZ_JANFMK010000010.1 | 99.001 | 801 | 8 | 0 | 30265 | 29465 |
| NZ_CP102137.1 | 99.001 | 801 | 8 | 0 | 1003793 | 1004593 |
| NZ_JAMWER010000014.1 | 99.001 | 801 | 8 | 0 | 34604 | 33804 |
| NZ_JAMZBL010000005.1 | 99.001 | 801 | 8 | 0 | 47802 | 47002 |
| NZ_JAMZBR010000007.1 | 99.001 | 801 | 8 | 0 | 13232 | 14032 |
| NZ_JAMZBM010000002.1 | 99.001 | 801 | 8 | 0 | 18768 | 19568 |
| NZ_JAMYIS010000001.1 | 99.001 | 801 | 8 | 0 | 338249 | 339049 |
| NZ_JAMDIX010000012.1 | 99.001 | 801 | 8 | 0 | 35160 | 35960 |
| NZ_JAMDIT010000008.1 | 99.001 | 801 | 8 | 0 | 34604 | 33804 |
| NZ_JAMDIS010000001.1 | 99.001 | 801 | 8 | 0 | 69057 | 69857 |
| NZ_JAMDIH010000011.1 | 99.001 | 801 | 8 | 0 | 30246 | 29446 |
| NZ_JAMDID010000024.1 | 99.001 | 801 | 8 | 0 | 20245 | 19445 |
| NZ_JAASCY010000008.1 | 99.001 | 801 | 8 | 0 | 212525 | 213325 |
| NZ_JAASCG010000002.1 | 99.001 | 801 | 8 | 0 | 212510 | 213310 |
| NZ_JAKTCI010000014.1 | 99.001 | 801 | 8 | 0 | 30246 | 29446 |
| NZ_JAKTCJ010000001.1 | 99.001 | 801 | 8 | 0 | 125130 | 125930 |
| NZ_JAKTCK010000001.1 | 99.001 | 801 | 8 | 0 | 125130 | 125930 |
| NZ_CP085087.1 | 99.001 | 801 | 8 | 0 | 1143578 | 1144378 |
| NZ_CP085086.1 | 99.001 | 801 | 8 | 0 | 1106564 | 1107364 |
| NZ_JAJBSN010000001.1 | 99.001 | 801 | 8 | 0 | 2106350 | 2105550 |
| NZ_CP082202.1 | 99.001 | 801 | 8 | 0 | 999440 | 998640 |
| NZ_JAIMZF010000006.1 | 99.001 | 801 | 8 | 0 | 65819 | 65019 |
| NZ_JAIMZE010000005.1 | 99.001 | 801 | 8 | 0 | 65819 | 65019 |
| NZ_JAIMDZ010000008.1 | 99.001 | 801 | 8 | 0 | 36627 | 37427 |
| NZ_JAIMDS010000009.1 | 99.001 | 801 | 8 | 0 | 31142 | 31942 |
| NZ_VZMY01000013.1 | 99.001 | 801 | 8 | 0 | 35106 | 35906 |
| NZ_VZNH01000007.1 | 99.001 | 801 | 8 | 0 | 34628 | 33828 |
| NZ_VZNJ01000003.1 | 99.001 | 801 | 8 | 0 | 34618 | 33818 |
| NZ_VZNL01000012.1 | 99.001 | 801 | 8 | 0 | 33150 | 33950 |
| NZ_VZNU01000004.1 | 99.001 | 801 | 8 | 0 | 34463 | 33663 |
| NZ_VZNV01000004.1 | 99.001 | 801 | 8 | 0 | 14430 | 13630 |
| NZ_VZOB01000011.1 | 99.001 | 801 | 8 | 0 | 30270 | 29470 |
| NZ_VZMP01000001.1 | 99.001 | 801 | 8 | 0 | 388019 | 387219 |
| NZ_JAEUXE010000007.1 | 99.001 | 801 | 8 | 0 | 68695 | 67895 |
| NZ_JAEUXF010000014.1 | 99.001 | 801 | 8 | 0 | 30349 | 29549 |
| NZ_AP023392.1 | 99.001 | 801 | 8 | 0 | 895587 | 894787 |
| NZ_JABLGJ010000002.1 | 99.001 | 801 | 8 | 0 | 206674 | 205874 |
| NZ_JABLKY010000001.1 | 99.001 | 801 | 8 | 0 | 118556 | 117756 |
| NZ_JABLMA010000001.1 | 99.001 | 801 | 8 | 0 | 118498 | 117698 |
| NZ_JABLMB010000001.1 | 99.001 | 801 | 8 | 0 | 118405 | 117605 |
| NZ_JABLMD010000002.1 | 99.001 | 801 | 8 | 0 | 118405 | 117605 |
| NZ_JABLMC010000001.1 | 99.001 | 801 | 8 | 0 | 118572 | 117772 |
| NZ_JABLMF010000001.1 | 99.001 | 801 | 8 | 0 | 60480 | 59680 |
| NZ_JABLNL010000006.1 | 99.001 | 801 | 8 | 0 | 31363 | 32163 |
| NZ_JABLNN010000007.1 | 99.001 | 801 | 8 | 0 | 31264 | 32064 |
| NZ_JABLOT010000001.1 | 99.001 | 801 | 8 | 0 | 116744 | 115944 |
| NZ_JABLOZ010000001.1 | 99.001 | 801 | 8 | 0 | 64122 | 64922 |
| NZ_JABLPD010000007.1 | 99.001 | 801 | 8 | 0 | 31330 | 32130 |
| NZ_JABLON010000001.1 | 99.001 | 801 | 8 | 0 | 117198 | 116398 |
| NZ_JABLPR010000001.1 | 99.001 | 801 | 8 | 0 | 84989 | 85789 |
| NZ_JABLPH010000007.1 | 99.001 | 801 | 8 | 0 | 62776 | 61976 |
| NZ_JABLLU010000001.1 | 99.001 | 801 | 8 | 0 | 21187 | 20387 |
| NZ_JABLPF010000015.1 | 99.001 | 801 | 8 | 0 | 31265 | 32065 |
| NZ_JABKRX010000003.1 | 99.001 | 801 | 8 | 0 | 149495 | 150295 |
| NZ_JABKSG010000012.1 | 99.001 | 801 | 8 | 0 | 35821 | 36621 |
| NZ_JABKSW010000003.1 | 99.001 | 801 | 8 | 0 | 64838 | 65638 |
| NZ_JABKTL010000019.1 | 99.001 | 801 | 8 | 0 | 30697 | 31497 |
| NZ_JABKSC010000007.1 | 99.001 | 801 | 8 | 0 | 30710 | 31510 |
| NZ_JABKWD010000001.1 | 99.001 | 801 | 8 | 0 | 455624 | 454824 |
| NZ_JABKWV010000011.1 | 99.001 | 801 | 8 | 0 | 34667 | 33867 |
| NZ_JABKWW010000001.1 | 99.001 | 801 | 8 | 0 | 34667 | 33867 |
| NZ_JABKVW010000002.1 | 99.001 | 801 | 8 | 0 | 88453 | 89253 |
| NZ_JABKZK010000011.1 | 99.001 | 801 | 8 | 0 | 34667 | 33867 |
| NZ_JABLAA010000011.1 | 99.001 | 801 | 8 | 0 | 3648 | 4448 |
| NZ_JABLAB010000023.1 | 99.001 | 801 | 8 | 0 | 21008 | 20208 |
| NZ_JABKXZ010000001.1 | 99.001 | 801 | 8 | 0 | 34653 | 33853 |
| NZ_JABLAL010000003.1 | 99.001 | 801 | 8 | 0 | 36041 | 36841 |
| NZ_JABLAY010000001.1 | 99.001 | 801 | 8 | 0 | 116720 | 115920 |
| NZ_JABLBC010000009.1 | 99.001 | 801 | 8 | 0 | 10832 | 11632 |
| NZ_JABLBE010000007.1 | 99.001 | 801 | 8 | 0 | 17108 | 16308 |
| NZ_JABLCY010000001.1 | 99.001 | 801 | 8 | 0 | 63788 | 64588 |
| NZ_JABLCN010000004.1 | 99.001 | 801 | 8 | 0 | 97003 | 97803 |
| NZ_JABLDB010000001.1 | 99.001 | 801 | 8 | 0 | 214414 | 215214 |
| NZ_JABLCK010000004.1 | 99.001 | 801 | 8 | 0 | 97005 | 97805 |
| NZ_JABLDC010000012.1 | 99.001 | 801 | 8 | 0 | 34847 | 34047 |
| NZ_JABCRV010000001.1 | 99.001 | 801 | 8 | 0 | 212594 | 213394 |
| NZ_VIED01000009.1 | 99.001 | 801 | 8 | 0 | 19278 | 20078 |
| NZ_SSXL01000008.1 | 99.001 | 801 | 8 | 0 | 36627 | 37427 |
| NZ_RSDP01000008.1 | 99.001 | 801 | 8 | 0 | 52280 | 53080 |
| NZ_RSDR01000020.1 | 99.001 | 801 | 8 | 0 | 26660 | 27460 |
| NZ_RSDM01000001.1 | 99.001 | 801 | 8 | 0 | 117226 | 116426 |
| NZ_RSDJ01000001.1 | 99.001 | 801 | 8 | 0 | 123488 | 122688 |
| NZ_RSDD01000001.1 | 99.001 | 801 | 8 | 0 | 117225 | 116425 |
| NZ_RSDF01000001.1 | 99.001 | 801 | 8 | 0 | 52291 | 53091 |
| NZ_POPL01000206.1 | 99.001 | 801 | 8 | 0 | 21017 | 20217 |
| NZ_POPH01000040.1 | 99.001 | 801 | 8 | 0 | 21014 | 20214 |
| NZ_POPK01000064.1 | 99.001 | 801 | 8 | 0 | 21017 | 20217 |
| NZ_POPG01000035.1 | 99.001 | 801 | 8 | 0 | 21017 | 20217 |
| NZ_POOD01000058.1 | 99.001 | 801 | 8 | 0 | 216344 | 217144 |
| NZ_POLT01000512.1 | 99.001 | 801 | 8 | 0 | 35093 | 35893 |
| NZ_POKY01000146.1 | 99.001 | 801 | 8 | 0 | 111997 | 112797 |
| NZ_POKU01000312.1 | 99.001 | 801 | 8 | 0 | 21017 | 20217 |
| NZ_POOE01000039.1 | 99.001 | 801 | 8 | 0 | 216344 | 217144 |
| NZ_POLY01000111.1 | 99.001 | 801 | 8 | 0 | 31197 | 30397 |
| NZ_POLX01000360.1 | 99.001 | 801 | 8 | 0 | 53131 | 52331 |
| NZ_POLV01000089.1 | 99.001 | 801 | 8 | 0 | 35093 | 35893 |
| NZ_POLU01000056.1 | 99.001 | 801 | 8 | 0 | 53130 | 52330 |
| NZ_POLA01000122.1 | 99.001 | 801 | 8 | 0 | 31458 | 32258 |
| NZ_POJE01000071.1 | 99.001 | 801 | 8 | 0 | 14244 | 15044 |
| NZ_CP017142.1 | 99.001 | 801 | 8 | 0 | 835346 | 834546 |
| NZ_CP015557.1 | 99.001 | 801 | 8 | 0 | 858926 | 858126 |
| NZ_CEEP01000028.1 | 99.001 | 801 | 8 | 0 | 215200 | 216000 |
| NZ_CEGP01000017.1 | 99.001 | 801 | 8 | 0 | 62632 | 61832 |
| NZ_CEDZ01000047.1 | 99.001 | 801 | 8 | 0 | 65292 | 64492 |
| NZ_CEIE01000004.1 | 99.001 | 801 | 8 | 0 | 47863 | 48663 |
| NZ_CEHW01000046.1 | 99.001 | 801 | 8 | 0 | 14314 | 13514 |
| NZ_CEEK01000107.1 | 99.001 | 801 | 8 | 0 | 34828 | 34028 |
| NZ_CEGT01000052.1 | 99.001 | 801 | 8 | 0 | 178168 | 177368 |
| NZ_CEDB01000013.1 | 99.001 | 801 | 8 | 0 | 130803 | 131603 |
| NZ_CEKF01000010.1 | 99.001 | 801 | 8 | 0 | 69787 | 68987 |
| NZ_CEHK01000037.1 | 99.001 | 801 | 8 | 0 | 22498 | 23298 |
| NZ_CEDF01000108.1 | 99.001 | 801 | 8 | 0 | 14326 | 13526 |
| NZ_CEDS01000009.1 | 99.001 | 801 | 8 | 0 | 116661 | 115861 |
| NZ_ALND01000023.1 | 99.001 | 801 | 8 | 0 | 66465 | 65665 |
| NZ_ALNB01000021.1 | 99.001 | 801 | 8 | 0 | 65377 | 64577 |
| NZ_ALMQ01000004.1 | 99.001 | 801 | 8 | 0 | 151021 | 150221 |
| NZ_ALLM01000003.1 | 99.001 | 801 | 8 | 0 | 34363 | 33563 |
| NZ_ALMT01000059.1 | 99.001 | 801 | 8 | 0 | 59942 | 59142 |
| FIGR01000001.1 | 99.001 | 801 | 8 | 0 | 130701 | 131501 |
| FIGB01000001.1 | 99.001 | 801 | 8 | 0 | 337175 | 337975 |
| FIFS01000002.1 | 99.001 | 801 | 8 | 0 | 31066 | 31866 |
| FIIR01000001.1 | 99.001 | 801 | 8 | 0 | 34831 | 34031 |
| FIIO01000001.1 | 99.001 | 801 | 8 | 0 | 116662 | 115862 |
| FIFQ01000011.1 | 99.001 | 801 | 8 | 0 | 65325 | 64525 |
| FIIS01000001.1 | 99.001 | 801 | 8 | 0 | 215215 | 216015 |
| FIIG01000005.1 | 99.001 | 801 | 8 | 0 | 97001 | 97801 |
| FIIC01000004.1 | 99.001 | 801 | 8 | 0 | 14317 | 13517 |
| FIGP01000008.1 | 99.001 | 801 | 8 | 0 | 62736 | 61936 |
| FIGV01000013.1 | 99.001 | 801 | 8 | 0 | 47880 | 48680 |
| FIMB01000001.1 | 99.001 | 801 | 8 | 0 | 62929 | 63729 |
| CZFY01000003.1 | 99.001 | 801 | 8 | 0 | 52031 | 52831 |
| CZFT01000004.1 | 99.001 | 801 | 8 | 0 | 65194 | 64394 |
| CZGT01000012.1 | 99.001 | 801 | 8 | 0 | 30640 | 31440 |
| CZFW01000027.1 | 99.001 | 801 | 8 | 0 | 213167 | 213967 |
| CZGJ01000044.1 | 99.001 | 801 | 8 | 0 | 212279 | 213079 |
| CZFR01000011.1 | 99.001 | 801 | 8 | 0 | 7786 | 8586 |
| JBFNWU010000009.1 | 99.001 | 801 | 8 | 0 | 88475 | 89275 |
| JBBMMN010000019.1 | 99.001 | 801 | 8 | 0 | 34951 | 35751 |
| JBBMMM010000019.1 | 99.001 | 801 | 8 | 0 | 34951 | 35751 |
| JBBMFU010000001.1 | 99.001 | 801 | 8 | 0 | 136506 | 135706 |
| JBAPGR010000004.1 | 99.001 | 801 | 8 | 0 | 30232 | 29432 |
| JBAPCN010000014.1 | 99.001 | 801 | 8 | 0 | 35144 | 35944 |
| JBAPCM010000010.1 | 99.001 | 801 | 8 | 0 | 35067 | 35867 |
| JBAPCU010000001.1 | 99.001 | 801 | 8 | 0 | 209672 | 208872 |
| JBAPCV010000001.1 | 99.001 | 801 | 8 | 0 | 210000 | 209200 |
| JBAPCW010000011.1 | 99.001 | 801 | 8 | 0 | 35298 | 34498 |
| JBAPGS010000004.1 | 99.001 | 801 | 8 | 0 | 30230 | 29430 |
| JBAPGT010000014.1 | 99.001 | 801 | 8 | 0 | 30231 | 29431 |
| JBAPCH010000012.1 | 99.001 | 801 | 8 | 0 | 30230 | 29430 |
| JBAPCR010000001.1 | 99.001 | 801 | 8 | 0 | 210001 | 209201 |
| JBAPCE010000019.1 | 99.001 | 801 | 8 | 0 | 30231 | 29431 |
| JBAPCI010000013.1 | 99.001 | 801 | 8 | 0 | 30170 | 29370 |
| JBAPDM010000001.1 | 99.001 | 801 | 8 | 0 | 140071 | 140871 |
| JBAPGX010000018.1 | 99.001 | 801 | 8 | 0 | 30230 | 29430 |
| JBAPGV010000019.1 | 99.001 | 801 | 8 | 0 | 14733 | 15533 |
| JBAPCP010000001.1 | 99.001 | 801 | 8 | 0 | 83051 | 82251 |
| JBAPCQ010000001.1 | 99.001 | 801 | 8 | 0 | 125017 | 125817 |
| JBAPGQ010000013.1 | 99.001 | 801 | 8 | 0 | 30231 | 29431 |
| JBAPCT010000001.1 | 99.001 | 801 | 8 | 0 | 125008 | 125808 |
| JBAPGP010000017.1 | 99.001 | 801 | 8 | 0 | 18913 | 19713 |
| JBAPCD010000018.1 | 99.001 | 801 | 8 | 0 | 30232 | 29432 |
| JBAPCK010000012.1 | 99.001 | 801 | 8 | 0 | 30232 | 29432 |
| JBAPCJ010000012.1 | 99.001 | 801 | 8 | 0 | 30169 | 29369 |
| JBAPCF010000022.1 | 99.001 | 801 | 8 | 0 | 1013 | 213 |
| JBAPCL010000014.1 | 99.001 | 801 | 8 | 0 | 35066 | 35866 |
| JBAPCG010000022.1 | 99.001 | 801 | 8 | 0 | 1013 | 213 |
| JBAPCS010000001.1 | 99.001 | 801 | 8 | 0 | 209672 | 208872 |
| JBAPGW010000032.1 | 99.001 | 801 | 8 | 0 | 15800 | 15000 |
| JBAPGU010000013.1 | 99.001 | 801 | 8 | 0 | 30170 | 29370 |
| JAVIGN010000001.1 | 99.001 | 801 | 8 | 0 | 52325 | 53125 |
| JAVIGS010000001.1 | 99.001 | 801 | 8 | 0 | 52305 | 53105 |
| JAVIGV010000014.1 | 99.001 | 801 | 8 | 0 | 34605 | 33805 |
| JAVIGX010000002.1 | 99.001 | 801 | 8 | 0 | 93791 | 92991 |
| JAVIGW010000001.1 | 99.001 | 801 | 8 | 0 | 94147 | 93347 |
| JAVIHB010000001.1 | 99.001 | 801 | 8 | 0 | 52325 | 53125 |
| JAVIHA010000001.1 | 99.001 | 801 | 8 | 0 | 93954 | 93154 |
| JAVIHC010000001.1 | 99.001 | 801 | 8 | 0 | 117260 | 116460 |
| JAVIHJ010000001.1 | 99.001 | 801 | 8 | 0 | 52984 | 53784 |
| JAVIHK010000003.1 | 99.001 | 801 | 8 | 0 | 212159 | 212959 |
| JAVIHP010000001.1 | 99.001 | 801 | 8 | 0 | 52325 | 53125 |
| JAVIHT010000001.1 | 99.001 | 801 | 8 | 0 | 59081 | 58281 |
| JAVIHU010000001.1 | 99.001 | 801 | 8 | 0 | 117929 | 117129 |
| JAVIHW010000002.1 | 99.001 | 801 | 8 | 0 | 220132 | 220932 |
| JAVIHZ010000001.1 | 99.001 | 801 | 8 | 0 | 52293 | 53093 |
| JAZDUV010000001.1 | 99.001 | 801 | 8 | 0 | 212204 | 213004 |
| JAZDVT010000004.1 | 99.001 | 801 | 8 | 0 | 34657 | 33857 |
| CP141824.1 | 99.001 | 801 | 8 | 0 | 1334857 | 1335657 |
| DATTWS010000001.1 | 99.001 | 801 | 8 | 0 | 307670 | 308470 |
| DATTWR010000002.1 | 99.001 | 801 | 8 | 0 | 140687 | 141487 |
| DATSRT010000017.1 | 99.001 | 801 | 8 | 0 | 15838 | 15038 |
| DATSRW010000012.1 | 99.001 | 801 | 8 | 0 | 7708 | 8508 |
| CP139875.1 | 99.001 | 801 | 8 | 0 | 1882760 | 1883560 |
| JAXKWP010000001.1 | 99.001 | 801 | 8 | 0 | 629426 | 630226 |
| DASGYK010000002.1 | 99.001 | 801 | 8 | 0 | 53586 | 52786 |
| DASGYD010000001.1 | 99.001 | 801 | 8 | 0 | 136051 | 135251 |
| DASGXZ010000017.1 | 99.001 | 801 | 8 | 0 | 3002 | 2202 |
| DASGXY010000004.1 | 99.001 | 801 | 8 | 0 | 74642 | 75442 |
| DASGXU010000016.1 | 99.001 | 801 | 8 | 0 | 21033 | 20233 |
| DASGXO010000001.1 | 99.001 | 801 | 8 | 0 | 105721 | 104921 |
| DASGXE010000001.1 | 99.001 | 801 | 8 | 0 | 96281 | 97081 |
| DASGWD010000038.1 | 99.001 | 801 | 8 | 0 | 3204 | 2404 |
| DASGWC010000039.1 | 99.001 | 801 | 8 | 0 | 3204 | 2404 |
| DASGWE010000016.1 | 99.001 | 801 | 8 | 0 | 34631 | 33831 |
| DASGWA010000038.1 | 99.001 | 801 | 8 | 0 | 3204 | 2404 |
| DASGVZ010000034.1 | 99.001 | 801 | 8 | 0 | 7693 | 8493 |
| DASGVR010000044.1 | 99.001 | 801 | 8 | 0 | 3204 | 2404 |
| DASGVI010000016.1 | 99.001 | 801 | 8 | 0 | 34631 | 33831 |
| DASGVE010000037.1 | 99.001 | 801 | 8 | 0 | 3204 | 2404 |
| DASGVB010000006.1 | 99.001 | 801 | 8 | 0 | 50045 | 49245 |
| DASGUM010000037.1 | 99.001 | 801 | 8 | 0 | 3204 | 2404 |
| DASGST010000036.1 | 99.001 | 801 | 8 | 0 | 7693 | 8493 |
| DASGSS010000034.1 | 99.001 | 801 | 8 | 0 | 7693 | 8493 |
| DASGSR010000038.1 | 99.001 | 801 | 8 | 0 | 3204 | 2404 |
| DASGSP010000024.1 | 99.001 | 801 | 8 | 0 | 15538 | 14738 |
| DASGSI010000006.1 | 99.001 | 801 | 8 | 0 | 53440 | 52640 |
| DASGSF010000039.1 | 99.001 | 801 | 8 | 0 | 3204 | 2404 |
| DASGSC010000008.1 | 99.001 | 801 | 8 | 0 | 60773 | 61573 |
| DASGRW010000038.1 | 99.001 | 801 | 8 | 0 | 3204 | 2404 |
| DASGRH010000038.1 | 99.001 | 801 | 8 | 0 | 3204 | 2404 |
| DASGRC010000039.1 | 99.001 | 801 | 8 | 0 | 3204 | 2404 |
| DASGPV010000002.1 | 99.001 | 801 | 8 | 0 | 36122 | 35322 |
| DASGPW010000004.1 | 99.001 | 801 | 8 | 0 | 32881 | 33681 |
| DASGPS010000005.1 | 99.001 | 801 | 8 | 0 | 32881 | 33681 |
| DASGPQ010000001.1 | 99.001 | 801 | 8 | 0 | 254155 | 254955 |
| DASGPR010000001.1 | 99.001 | 801 | 8 | 0 | 254155 | 254955 |
| DASGPL010000006.1 | 99.001 | 801 | 8 | 0 | 53496 | 52696 |
| DASGPM010000006.1 | 99.001 | 801 | 8 | 0 | 53440 | 52640 |
| DASGPD010000016.1 | 99.001 | 801 | 8 | 0 | 34631 | 33831 |
| DASGPF010000016.1 | 99.001 | 801 | 8 | 0 | 34631 | 33831 |
| DASGPA010000025.1 | 99.001 | 801 | 8 | 0 | 30234 | 29434 |
| DASGPB010000017.1 | 99.001 | 801 | 8 | 0 | 34631 | 33831 |
| DASGPC010000016.1 | 99.001 | 801 | 8 | 0 | 34631 | 33831 |
| DASGOY010000015.1 | 99.001 | 801 | 8 | 0 | 34631 | 33831 |
| DASGOW010000003.1 | 99.001 | 801 | 8 | 0 | 111443 | 110643 |
| DASGOO010000001.1 | 99.001 | 801 | 8 | 0 | 36122 | 35322 |
| DASGNL010000041.1 | 99.001 | 801 | 8 | 0 | 3202 | 2402 |
| DASGNK010000039.1 | 99.001 | 801 | 8 | 0 | 3204 | 2404 |
| DASGNJ010000038.1 | 99.001 | 801 | 8 | 0 | 3204 | 2404 |
| DASGNG010000039.1 | 99.001 | 801 | 8 | 0 | 3204 | 2404 |
| DASGNI010000039.1 | 99.001 | 801 | 8 | 0 | 3204 | 2404 |
| DASGNC010000039.1 | 99.001 | 801 | 8 | 0 | 3204 | 2404 |
| DASGMY010000038.1 | 99.001 | 801 | 8 | 0 | 3204 | 2404 |
| DASGMU010000034.1 | 99.001 | 801 | 8 | 0 | 3207 | 2407 |
| DASGMV010000035.1 | 99.001 | 801 | 8 | 0 | 3204 | 2404 |
| DASGEI010000002.1 | 99.001 | 801 | 8 | 0 | 31154 | 31954 |
| DASGEH010000001.1 | 99.001 | 801 | 8 | 0 | 63903 | 64703 |
| DASGEF010000001.1 | 99.001 | 801 | 8 | 0 | 63903 | 64703 |
| DASGEG010000001.1 | 99.001 | 801 | 8 | 0 | 63903 | 64703 |
| DASGED010000001.1 | 99.001 | 801 | 8 | 0 | 60382 | 59582 |
| DASGBH010000010.1 | 99.001 | 801 | 8 | 0 | 53724 | 52924 |
| DASGAY010000001.1 | 99.001 | 801 | 8 | 0 | 126486 | 127286 |
| DASGAW010000002.1 | 99.001 | 801 | 8 | 0 | 60003 | 59203 |
| DASGAI010000004.1 | 99.001 | 801 | 8 | 0 | 64608 | 63808 |
| DASGAE010000057.1 | 99.001 | 801 | 8 | 0 | 13568 | 12768 |
| DASGAA010000009.1 | 99.001 | 801 | 8 | 0 | 67072 | 66272 |
| DASFYZ010000008.1 | 99.001 | 801 | 8 | 0 | 46354 | 45554 |
| DASFYR010000023.1 | 99.001 | 801 | 8 | 0 | 13886 | 13086 |
| DASFYJ010000012.1 | 99.001 | 801 | 8 | 0 | 40774 | 39974 |
| DASFYG010000065.1 | 99.001 | 801 | 8 | 0 | 13886 | 13086 |
| DASFYF010000002.1 | 99.001 | 801 | 8 | 0 | 46438 | 45638 |
| DASFVA010000094.1 | 99.123 | 798 | 7 | 0 | 2653 | 1856 |
| DASFUN010000065.1 | 99.001 | 801 | 8 | 0 | 10690 | 9890 |
| DASFUB010000062.1 | 99.001 | 801 | 8 | 0 | 7174 | 6374 |
| DASFRI010000012.1 | 99.001 | 801 | 8 | 0 | 10121 | 9321 |
| DASFRB010000045.1 | 99.001 | 801 | 8 | 0 | 8891 | 9691 |
| DASFQR010000003.1 | 99.001 | 801 | 8 | 0 | 112055 | 111255 |
| DASFQE010000006.1 | 99.001 | 801 | 8 | 0 | 7708 | 8508 |
| DASFQB010000003.1 | 99.001 | 801 | 8 | 0 | 48090 | 48890 |
| DASFOO010000006.1 | 99.001 | 801 | 8 | 0 | 13318 | 14118 |
| DASFLQ010000016.1 | 99.001 | 801 | 8 | 0 | 34631 | 33831 |
| DASFIS010000009.1 | 99.001 | 801 | 8 | 0 | 56295 | 55495 |
| DASFIE010000016.1 | 99.001 | 801 | 8 | 0 | 20416 | 19616 |
| DASFHX010000002.1 | 99.001 | 801 | 8 | 0 | 62890 | 63690 |
| DASFHQ010000005.1 | 99.001 | 801 | 8 | 0 | 119672 | 118872 |
| DASFHO010000013.1 | 99.001 | 801 | 8 | 0 | 30168 | 29368 |
| DASFEZ010000013.1 | 99.001 | 801 | 8 | 0 | 1502 | 2302 |
| DASFDX010000001.1 | 99.001 | 801 | 8 | 0 | 35288 | 34488 |
| DASFDL010000005.1 | 99.001 | 801 | 8 | 0 | 108150 | 107350 |
| DASEVE010000025.1 | 99.001 | 801 | 8 | 0 | 10708 | 9908 |
| DASEVB010000002.1 | 99.001 | 801 | 8 | 0 | 13837 | 14637 |
| DASEUZ010000012.1 | 99.001 | 801 | 8 | 0 | 53186 | 52386 |
| DASEUN010000003.1 | 99.001 | 801 | 8 | 0 | 108161 | 107361 |
| DASEUM010000005.1 | 99.001 | 801 | 8 | 0 | 16179 | 15379 |
| DASEUF010000001.1 | 99.001 | 801 | 8 | 0 | 134593 | 133793 |
| DASEUG010000004.1 | 99.001 | 801 | 8 | 0 | 2688 | 1888 |
| DASETM010000001.1 | 99.001 | 801 | 8 | 0 | 179064 | 178264 |
| DASEOE010000012.1 | 99.001 | 801 | 8 | 0 | 34288 | 33488 |
| DASENK010000010.1 | 99.001 | 801 | 8 | 0 | 30167 | 29367 |
| DASENL010000010.1 | 99.001 | 801 | 8 | 0 | 30167 | 29367 |
| DASENJ010000011.1 | 99.001 | 801 | 8 | 0 | 30167 | 29367 |
| DASENH010000011.1 | 99.001 | 801 | 8 | 0 | 30167 | 29367 |
| DASENI010000009.1 | 99.001 | 801 | 8 | 0 | 30168 | 29368 |
| DASENG010000014.1 | 99.001 | 801 | 8 | 0 | 30168 | 29368 |
| DASENE010000009.1 | 99.001 | 801 | 8 | 0 | 30168 | 29368 |
| DASEND010000011.1 | 99.001 | 801 | 8 | 0 | 30168 | 29368 |
| DASENF010000009.1 | 99.001 | 801 | 8 | 0 | 30168 | 29368 |
| DASENA010000011.1 | 99.001 | 801 | 8 | 0 | 30168 | 29368 |
| DASENC010000011.1 | 99.001 | 801 | 8 | 0 | 30168 | 29368 |
| DASEMV010000011.1 | 99.001 | 801 | 8 | 0 | 30168 | 29368 |
| DASEMW010000013.1 | 99.001 | 801 | 8 | 0 | 30168 | 29368 |
| DASEMX010000011.1 | 99.001 | 801 | 8 | 0 | 30168 | 29368 |
| DASEMT010000003.1 | 99.001 | 801 | 8 | 0 | 30168 | 29368 |
| DASEMU010000012.1 | 99.001 | 801 | 8 | 0 | 30168 | 29368 |
| DASEMS010000012.1 | 99.001 | 801 | 8 | 0 | 30168 | 29368 |
| DASEMR010000011.1 | 99.001 | 801 | 8 | 0 | 30168 | 29368 |
| DASEMP010000011.1 | 99.001 | 801 | 8 | 0 | 30168 | 29368 |
| DASEMQ010000001.1 | 99.001 | 801 | 8 | 0 | 178977 | 178177 |
| DASEMN010000011.1 | 99.001 | 801 | 8 | 0 | 30168 | 29368 |
| DASEMO010000014.1 | 99.001 | 801 | 8 | 0 | 30168 | 29368 |
| DASEMM010000011.1 | 99.001 | 801 | 8 | 0 | 30168 | 29368 |
| DASEMJ010000001.1 | 99.001 | 801 | 8 | 0 | 183958 | 183158 |
| DASEMH010000012.1 | 99.001 | 801 | 8 | 0 | 30168 | 29368 |
| DASEMI010000001.1 | 99.001 | 801 | 8 | 0 | 183958 | 183158 |
| DASEMG010000002.1 | 99.001 | 801 | 8 | 0 | 1384 | 2184 |
| DASEME010000011.1 | 99.001 | 801 | 8 | 0 | 30168 | 29368 |
| DASEMF010000011.1 | 99.001 | 801 | 8 | 0 | 30168 | 29368 |
| DASEMD010000002.1 | 99.001 | 801 | 8 | 0 | 1383 | 2183 |
| DASEMB010000012.1 | 99.001 | 801 | 8 | 0 | 30168 | 29368 |
| DASEMC010000012.1 | 99.001 | 801 | 8 | 0 | 30168 | 29368 |
| DASELW010000016.1 | 99.001 | 801 | 8 | 0 | 30168 | 29368 |
| DASELZ010000012.1 | 99.001 | 801 | 8 | 0 | 30168 | 29368 |
| DASELY010000011.1 | 99.001 | 801 | 8 | 0 | 30168 | 29368 |
| DASELV010000010.1 | 99.001 | 801 | 8 | 0 | 30168 | 29368 |
| DASELX010000011.1 | 99.001 | 801 | 8 | 0 | 30168 | 29368 |
| DASEJV010000011.1 | 99.001 | 801 | 8 | 0 | 30168 | 29368 |
| DASEHZ010000009.1 | 99.001 | 801 | 8 | 0 | 27029 | 26229 |
| DASEFG010000010.1 | 99.001 | 801 | 8 | 0 | 7705 | 8505 |
| DASEEF010000001.1 | 99.001 | 801 | 8 | 0 | 212007 | 212807 |
| DASEDQ010000001.1 | 99.001 | 801 | 8 | 0 | 276959 | 277759 |
| DASEDP010000002.1 | 99.001 | 801 | 8 | 0 | 271380 | 272180 |
| DASECM010000018.1 | 99.001 | 801 | 8 | 0 | 19452 | 18652 |
| DASEBV010000012.1 | 99.001 | 801 | 8 | 0 | 7708 | 8508 |
| DASEBY010000006.1 | 99.001 | 801 | 8 | 0 | 85062 | 85862 |
| DASCLQ010000008.1 | 99.001 | 801 | 8 | 0 | 34529 | 33729 |
| DASCLN010000018.1 | 99.001 | 801 | 8 | 0 | 34525 | 33725 |
| DARZUB010000021.1 | 99.001 | 801 | 8 | 0 | 7736 | 8536 |
| DARZUD010000003.1 | 99.001 | 801 | 8 | 0 | 148821 | 149621 |
| DARZUA010000019.1 | 99.001 | 801 | 8 | 0 | 7703 | 8503 |
| DARZTQ010000023.1 | 99.001 | 801 | 8 | 0 | 7708 | 8508 |
| DARZTK010000001.1 | 99.001 | 801 | 8 | 0 | 211451 | 212251 |
| DARZST010000017.1 | 99.001 | 801 | 8 | 0 | 34631 | 33831 |
| DARZRW010000016.1 | 99.001 | 801 | 8 | 0 | 37381 | 36581 |
| DARZRD010000013.1 | 99.001 | 801 | 8 | 0 | 7708 | 8508 |
| DARZQR010000013.1 | 99.001 | 801 | 8 | 0 | 7704 | 8504 |
| DARZQP010000015.1 | 99.001 | 801 | 8 | 0 | 34631 | 33831 |
| DARZQE010000001.1 | 99.001 | 801 | 8 | 0 | 34530 | 33730 |
| DARZQB010000012.1 | 99.001 | 801 | 8 | 0 | 7704 | 8504 |
| DARZPU010000009.1 | 99.001 | 801 | 8 | 0 | 34526 | 33726 |
| DARZPR010000017.1 | 99.001 | 801 | 8 | 0 | 7745 | 8545 |
| DARZPP010000001.1 | 99.001 | 801 | 8 | 0 | 212080 | 212880 |
| DARZPF010000043.1 | 99.001 | 801 | 8 | 0 | 7708 | 8508 |
| DARZPA010000001.1 | 99.001 | 801 | 8 | 0 | 277484 | 278284 |
| DARZOY010000019.1 | 99.001 | 801 | 8 | 0 | 34631 | 33831 |
| DARZOU010000013.1 | 99.001 | 801 | 8 | 0 | 7703 | 8503 |
| DARZOQ010000017.1 | 99.001 | 801 | 8 | 0 | 34525 | 33725 |
| DARZOI010000016.1 | 99.001 | 801 | 8 | 0 | 34525 | 33725 |
| DARZLP010000023.1 | 99.001 | 801 | 8 | 0 | 34319 | 33519 |
| DARZLF010000011.1 | 99.001 | 801 | 8 | 0 | 35614 | 34814 |
| DARZKC010000023.1 | 99.001 | 801 | 8 | 0 | 30016 | 29216 |
| DARZJW010000005.1 | 99.001 | 801 | 8 | 0 | 33974 | 34774 |
| DARZJX010000002.1 | 99.001 | 801 | 8 | 0 | 212082 | 212882 |
| DARZJV010000015.1 | 99.001 | 801 | 8 | 0 | 34526 | 33726 |
| DARZJS010000006.1 | 99.001 | 801 | 8 | 0 | 34526 | 33726 |
| DARZJL010000034.1 | 99.001 | 801 | 8 | 0 | 7704 | 8504 |
| DARZIV010000001.1 | 99.001 | 801 | 8 | 0 | 212083 | 212883 |
| DARZIS010000021.1 | 99.001 | 801 | 8 | 0 | 20806 | 20006 |
| DARZIA010000018.1 | 99.001 | 801 | 8 | 0 | 7703 | 8503 |
| DARZHE010000001.1 | 99.001 | 801 | 8 | 0 | 212066 | 212866 |
| DARZGR010000025.1 | 99.001 | 801 | 8 | 0 | 35285 | 34485 |
| DARZFW010000005.1 | 99.001 | 801 | 8 | 0 | 89005 | 89805 |
| DARZFO010000013.1 | 99.001 | 801 | 8 | 0 | 7708 | 8508 |
| DARZFN010000008.1 | 99.001 | 801 | 8 | 0 | 34522 | 33722 |
| DARZFH010000014.1 | 99.001 | 801 | 8 | 0 | 7708 | 8508 |
| DARZFA010000012.1 | 99.001 | 801 | 8 | 0 | 34631 | 33831 |
| DARZDO010000018.1 | 99.001 | 801 | 8 | 0 | 7708 | 8508 |
| DARZDN010000001.1 | 99.001 | 801 | 8 | 0 | 212039 | 212839 |
| DARZDM010000013.1 | 99.001 | 801 | 8 | 0 | 7707 | 8507 |
| DARZDG010000010.1 | 99.001 | 801 | 8 | 0 | 35004 | 34204 |
| DARZDI010000012.1 | 99.001 | 801 | 8 | 0 | 34712 | 33912 |
| DARZDA010000013.1 | 99.001 | 801 | 8 | 0 | 7708 | 8508 |
| DARZCW010000012.1 | 99.001 | 801 | 8 | 0 | 45741 | 44941 |
| DARZCP010000012.1 | 99.001 | 801 | 8 | 0 | 7708 | 8508 |
| DARZCI010000004.1 | 99.001 | 801 | 8 | 0 | 10037 | 9237 |
| DARZCD010000005.1 | 99.001 | 801 | 8 | 0 | 161651 | 160851 |
| DARZBU010000018.1 | 99.001 | 801 | 8 | 0 | 34526 | 33726 |
| DARZBR010000002.1 | 99.001 | 801 | 8 | 0 | 219757 | 220557 |
| DARZBH010000020.1 | 99.001 | 801 | 8 | 0 | 20806 | 20006 |
| DARZBD010000014.1 | 99.001 | 801 | 8 | 0 | 18978 | 19778 |
| DARZAF010000013.1 | 99.001 | 801 | 8 | 0 | 42215 | 41415 |
| DARYZY010000020.1 | 99.001 | 801 | 8 | 0 | 18652 | 19452 |
| DARYZL010000001.1 | 99.001 | 801 | 8 | 0 | 63602 | 64402 |
| DARYYP010000017.1 | 99.001 | 801 | 8 | 0 | 34631 | 33831 |
| JAUTHW010000001.1 | 99.001 | 801 | 8 | 0 | 54600 | 55400 |
| CP134474.1 | 99.001 | 801 | 8 | 0 | 1117275 | 1118075 |
| JARASW010000001.1 | 99.001 | 801 | 8 | 0 | 35676 | 34876 |
| JANFMK010000010.1 | 99.001 | 801 | 8 | 0 | 30265 | 29465 |
| CP102137.1 | 99.001 | 801 | 8 | 0 | 1003793 | 1004593 |
| JAMWER010000014.1 | 99.001 | 801 | 8 | 0 | 34604 | 33804 |
| JAMZBL010000005.1 | 99.001 | 801 | 8 | 0 | 47802 | 47002 |
| JAMZBR010000007.1 | 99.001 | 801 | 8 | 0 | 13232 | 14032 |
| JAMZBM010000002.1 | 99.001 | 801 | 8 | 0 | 18768 | 19568 |
| JAMYIS010000001.1 | 99.001 | 801 | 8 | 0 | 338249 | 339049 |
| JAMDIX010000012.1 | 99.001 | 801 | 8 | 0 | 35160 | 35960 |
| JAMDIT010000008.1 | 99.001 | 801 | 8 | 0 | 34604 | 33804 |
| JAMDIS010000001.1 | 99.001 | 801 | 8 | 0 | 69057 | 69857 |
| JAMDIH010000011.1 | 99.001 | 801 | 8 | 0 | 30246 | 29446 |
| JAMDID010000024.1 | 99.001 | 801 | 8 | 0 | 20245 | 19445 |
| JAASCY010000008.1 | 99.001 | 801 | 8 | 0 | 212525 | 213325 |
| JAASCM010000001.1 | 99.001 | 801 | 8 | 0 | 20140 | 19340 |
| JAASCG010000002.1 | 99.001 | 801 | 8 | 0 | 212510 | 213310 |
| JAKTCI010000014.1 | 99.001 | 801 | 8 | 0 | 30246 | 29446 |
| JAKTCJ010000001.1 | 99.001 | 801 | 8 | 0 | 125130 | 125930 |
| JAKTCK010000001.1 | 99.001 | 801 | 8 | 0 | 125130 | 125930 |
| CP085087.1 | 99.001 | 801 | 8 | 0 | 1143578 | 1144378 |
| CP085086.1 | 99.001 | 801 | 8 | 0 | 1106564 | 1107364 |
| JAJBSN010000001.1 | 99.001 | 801 | 8 | 0 | 2106350 | 2105550 |
| CP082202.1 | 99.001 | 801 | 8 | 0 | 999440 | 998640 |
| JAIMZF010000006.1 | 99.001 | 801 | 8 | 0 | 65819 | 65019 |
| JAIMZE010000005.1 | 99.001 | 801 | 8 | 0 | 65819 | 65019 |
| JAIMDZ010000008.1 | 99.001 | 801 | 8 | 0 | 36627 | 37427 |
| JAIMDS010000009.1 | 99.001 | 801 | 8 | 0 | 31142 | 31942 |
| VZMY01000013.1 | 99.001 | 801 | 8 | 0 | 35106 | 35906 |
| VZNH01000007.1 | 99.001 | 801 | 8 | 0 | 34628 | 33828 |
| VZNJ01000003.1 | 99.001 | 801 | 8 | 0 | 34618 | 33818 |
| VZNL01000012.1 | 99.001 | 801 | 8 | 0 | 33150 | 33950 |
| VZNU01000004.1 | 99.001 | 801 | 8 | 0 | 34463 | 33663 |
| VZNV01000004.1 | 99.001 | 801 | 8 | 0 | 14430 | 13630 |
| VZOB01000011.1 | 99.001 | 801 | 8 | 0 | 30270 | 29470 |
| VZMP01000001.1 | 99.001 | 801 | 8 | 0 | 388019 | 387219 |
| JAEUXE010000007.1 | 99.001 | 801 | 8 | 0 | 68695 | 67895 |
| JAEUXF010000014.1 | 99.001 | 801 | 8 | 0 | 30349 | 29549 |
| AP023392.1 | 99.001 | 801 | 8 | 0 | 895587 | 894787 |
| JABLGJ010000002.1 | 99.001 | 801 | 8 | 0 | 206674 | 205874 |
| JABLKY010000001.1 | 99.001 | 801 | 8 | 0 | 118556 | 117756 |
| JABLMA010000001.1 | 99.001 | 801 | 8 | 0 | 118498 | 117698 |
| JABLMB010000001.1 | 99.001 | 801 | 8 | 0 | 118405 | 117605 |
| JABLMD010000002.1 | 99.001 | 801 | 8 | 0 | 118405 | 117605 |
| JABLMC010000001.1 | 99.001 | 801 | 8 | 0 | 118572 | 117772 |
| JABLMF010000001.1 | 99.001 | 801 | 8 | 0 | 60480 | 59680 |
| JABLNL010000006.1 | 99.001 | 801 | 8 | 0 | 31363 | 32163 |
| JABLNN010000007.1 | 99.001 | 801 | 8 | 0 | 31264 | 32064 |
| JABLOT010000001.1 | 99.001 | 801 | 8 | 0 | 116744 | 115944 |
| JABLOZ010000001.1 | 99.001 | 801 | 8 | 0 | 64122 | 64922 |
| JABLPD010000007.1 | 99.001 | 801 | 8 | 0 | 31330 | 32130 |
| JABLON010000001.1 | 99.001 | 801 | 8 | 0 | 117198 | 116398 |
| JABLPR010000001.1 | 99.001 | 801 | 8 | 0 | 84989 | 85789 |
| JABLPH010000007.1 | 99.001 | 801 | 8 | 0 | 62776 | 61976 |
| JABLLU010000001.1 | 99.001 | 801 | 8 | 0 | 21187 | 20387 |
| JABLPF010000015.1 | 99.001 | 801 | 8 | 0 | 31265 | 32065 |
| JABKRX010000003.1 | 99.001 | 801 | 8 | 0 | 149495 | 150295 |
| JABKSG010000012.1 | 99.001 | 801 | 8 | 0 | 35821 | 36621 |
| JABKSW010000003.1 | 99.001 | 801 | 8 | 0 | 64838 | 65638 |
| JABKTL010000019.1 | 99.001 | 801 | 8 | 0 | 30697 | 31497 |
| JABKSC010000007.1 | 99.001 | 801 | 8 | 0 | 30710 | 31510 |
| JABKWD010000001.1 | 99.001 | 801 | 8 | 0 | 455624 | 454824 |
| JABKWV010000011.1 | 99.001 | 801 | 8 | 0 | 34667 | 33867 |
| JABKWW010000001.1 | 99.001 | 801 | 8 | 0 | 34667 | 33867 |
| JABKVW010000002.1 | 99.001 | 801 | 8 | 0 | 88453 | 89253 |
| JABKZK010000011.1 | 99.001 | 801 | 8 | 0 | 34667 | 33867 |
| JABLAA010000011.1 | 99.001 | 801 | 8 | 0 | 3648 | 4448 |
| JABLAB010000023.1 | 99.001 | 801 | 8 | 0 | 21008 | 20208 |
| JABKXZ010000001.1 | 99.001 | 801 | 8 | 0 | 34653 | 33853 |
| JABLAL010000003.1 | 99.001 | 801 | 8 | 0 | 36041 | 36841 |
| JABLAY010000001.1 | 99.001 | 801 | 8 | 0 | 116720 | 115920 |
| JABLBC010000009.1 | 99.001 | 801 | 8 | 0 | 10832 | 11632 |
| JABLBE010000007.1 | 99.001 | 801 | 8 | 0 | 17108 | 16308 |
| JABLCY010000001.1 | 99.001 | 801 | 8 | 0 | 63788 | 64588 |
| JABLCN010000004.1 | 99.001 | 801 | 8 | 0 | 97003 | 97803 |
| JABLDB010000001.1 | 99.001 | 801 | 8 | 0 | 214414 | 215214 |
| JABLCK010000004.1 | 99.001 | 801 | 8 | 0 | 97005 | 97805 |
| JABLDC010000012.1 | 99.001 | 801 | 8 | 0 | 34847 | 34047 |
| JABCRV010000001.1 | 99.001 | 801 | 8 | 0 | 212594 | 213394 |
| VIEX01000007.1 | 99.001 | 801 | 8 | 0 | 1600044 | 1600844 |
| VIED01000009.1 | 99.001 | 801 | 8 | 0 | 19278 | 20078 |
| SSXL01000008.1 | 99.001 | 801 | 8 | 0 | 36627 | 37427 |
| RSDP01000008.1 | 99.001 | 801 | 8 | 0 | 52280 | 53080 |
| RSDR01000020.1 | 99.001 | 801 | 8 | 0 | 26660 | 27460 |
| RSDM01000001.1 | 99.001 | 801 | 8 | 0 | 117226 | 116426 |
| RSDJ01000001.1 | 99.001 | 801 | 8 | 0 | 123488 | 122688 |
| RSDD01000001.1 | 99.001 | 801 | 8 | 0 | 117225 | 116425 |
| RSDF01000001.1 | 99.001 | 801 | 8 | 0 | 52291 | 53091 |
| POPL01000206.1 | 99.001 | 801 | 8 | 0 | 21017 | 20217 |
| POPH01000040.1 | 99.001 | 801 | 8 | 0 | 21014 | 20214 |
| POPK01000064.1 | 99.001 | 801 | 8 | 0 | 21017 | 20217 |
| POPG01000035.1 | 99.001 | 801 | 8 | 0 | 21017 | 20217 |
| POOD01000058.1 | 99.001 | 801 | 8 | 0 | 216344 | 217144 |
| POLT01000512.1 | 99.001 | 801 | 8 | 0 | 35093 | 35893 |
| POKY01000146.1 | 99.001 | 801 | 8 | 0 | 111997 | 112797 |
| POKU01000312.1 | 99.001 | 801 | 8 | 0 | 21017 | 20217 |
| POKF01000784.1 | 99.001 | 801 | 8 | 0 | 218679 | 219479 |
| POOE01000039.1 | 99.001 | 801 | 8 | 0 | 216344 | 217144 |
| POLY01000111.1 | 99.001 | 801 | 8 | 0 | 31197 | 30397 |
| POLX01000360.1 | 99.001 | 801 | 8 | 0 | 53131 | 52331 |
| POLV01000089.1 | 99.001 | 801 | 8 | 0 | 35093 | 35893 |
| POLU01000056.1 | 99.001 | 801 | 8 | 0 | 53130 | 52330 |
| POLA01000122.1 | 99.001 | 801 | 8 | 0 | 31458 | 32258 |
| POJE01000071.1 | 99.001 | 801 | 8 | 0 | 14244 | 15044 |
| CP017142.1 | 99.001 | 801 | 8 | 0 | 835346 | 834546 |
| CP015557.1 | 99.001 | 801 | 8 | 0 | 858926 | 858126 |
| CEEP01000028.1 | 99.001 | 801 | 8 | 0 | 215200 | 216000 |
| CEGP01000017.1 | 99.001 | 801 | 8 | 0 | 62632 | 61832 |
| CEDZ01000047.1 | 99.001 | 801 | 8 | 0 | 65292 | 64492 |
| CEIE01000004.1 | 99.001 | 801 | 8 | 0 | 47863 | 48663 |
| CEHW01000046.1 | 99.001 | 801 | 8 | 0 | 14314 | 13514 |
| CEEK01000107.1 | 99.001 | 801 | 8 | 0 | 34828 | 34028 |
| CEGT01000052.1 | 99.001 | 801 | 8 | 0 | 178168 | 177368 |
| CEDB01000013.1 | 99.001 | 801 | 8 | 0 | 130803 | 131603 |
| CEKF01000010.1 | 99.001 | 801 | 8 | 0 | 69787 | 68987 |
| CEHK01000037.1 | 99.001 | 801 | 8 | 0 | 22498 | 23298 |
| CEDF01000108.1 | 99.001 | 801 | 8 | 0 | 14326 | 13526 |
| CEDS01000009.1 | 99.001 | 801 | 8 | 0 | 116661 | 115861 |
| ALND01000023.1 | 99.001 | 801 | 8 | 0 | 66465 | 65665 |
| ALNB01000021.1 | 99.001 | 801 | 8 | 0 | 65377 | 64577 |
| ALMQ01000004.1 | 99.001 | 801 | 8 | 0 | 151021 | 150221 |
| ALLM01000003.1 | 99.001 | 801 | 8 | 0 | 34363 | 33563 |
| ALMT01000059.1 | 99.001 | 801 | 8 | 0 | 59942 | 59142 |
| NZ_JAASBH010000027.1 | 98.876 | 801 | 9 | 0 | 10627 | 9827 |
| DASGAL010000071.1 | 99 | 800 | 8 | 0 | 10715 | 9916 |
| JAASBH010000027.1 | 98.876 | 801 | 9 | 0 | 10627 | 9827 |
| NZ_JAASEW010000021.1 | 98.876 | 801 | 9 | 0 | 16460 | 17260 |
| NZ_LR738723.1 | 98.876 | 801 | 9 | 0 | 981525 | 980725 |
| NZ_FIIA01000004.1 | 98.876 | 801 | 9 | 0 | 20681 | 19881 |
| NZ_FIFF01000031.1 | 98.876 | 801 | 9 | 0 | 20681 | 19881 |
| NZ_FIFE01000002.1 | 98.876 | 801 | 9 | 0 | 125379 | 126179 |
| NZ_FIIY01000004.1 | 98.876 | 801 | 9 | 0 | 48814 | 49614 |
| NZ_FIKO01000016.1 | 98.876 | 801 | 9 | 0 | 15810 | 16610 |
| NZ_FIJU01000013.1 | 98.876 | 801 | 9 | 0 | 17447 | 18247 |
| NZ_FIGF01000004.1 | 98.876 | 801 | 9 | 0 | 107757 | 108557 |
| NZ_FIKP01000026.1 | 98.876 | 801 | 9 | 0 | 7222 | 8022 |
| NZ_FIKK01000024.1 | 98.876 | 801 | 9 | 0 | 7963 | 8763 |
| NZ_FIGC01000004.1 | 98.876 | 801 | 9 | 0 | 107677 | 108477 |
| NZ_FILK01000001.1 | 98.876 | 801 | 9 | 0 | 252617 | 253417 |
| NZ_FIII01000033.1 | 98.876 | 801 | 9 | 0 | 20652 | 19852 |
| NZ_FIFY01000005.1 | 98.876 | 801 | 9 | 0 | 20681 | 19881 |
| NZ_FIJB01000004.1 | 98.876 | 801 | 9 | 0 | 20616 | 19816 |
| NZ_FIHJ01000004.1 | 98.876 | 801 | 9 | 0 | 20616 | 19816 |
| NZ_FIHF01000031.1 | 98.876 | 801 | 9 | 0 | 7071 | 7871 |
| NZ_FIJG01000002.1 | 98.876 | 801 | 9 | 0 | 107748 | 108548 |
| NZ_FIIJ01000001.1 | 98.876 | 801 | 9 | 0 | 20667 | 19867 |
| NZ_FIFH01000003.1 | 98.876 | 801 | 9 | 0 | 62179 | 62979 |
| NZ_FIKE01000001.1 | 98.876 | 801 | 9 | 0 | 252623 | 253423 |
| NZ_FIIK01000031.1 | 98.876 | 801 | 9 | 0 | 7073 | 7873 |
| NZ_FIIH01000030.1 | 98.876 | 801 | 9 | 0 | 20658 | 19858 |
| NZ_FIJP01000009.1 | 98.876 | 801 | 9 | 0 | 20560 | 19760 |
| NZ_FIJS01000010.1 | 98.876 | 801 | 9 | 0 | 15789 | 14989 |
| NZ_FIFG01000006.1 | 98.876 | 801 | 9 | 0 | 20162 | 19362 |
| NZ_FIIF01000022.1 | 98.876 | 801 | 9 | 0 | 1457 | 2257 |
| NZ_FIIB01000026.1 | 98.876 | 801 | 9 | 0 | 20158 | 19358 |
| NZ_FIKD01000004.1 | 98.876 | 801 | 9 | 0 | 109920 | 110720 |
| NZ_FIJM01000019.1 | 98.876 | 801 | 9 | 0 | 3007 | 2207 |
| NZ_FIJL01000023.1 | 98.876 | 801 | 9 | 0 | 34452 | 35252 |
| NZ_FIGZ01000001.1 | 98.876 | 801 | 9 | 0 | 162814 | 163614 |
| NZ_FIMA01000001.1 | 98.876 | 801 | 9 | 0 | 252607 | 253407 |
| NZ_FILO01000017.1 | 98.876 | 801 | 9 | 0 | 30229 | 29429 |
| NZ_FIGE01000004.1 | 98.876 | 801 | 9 | 0 | 107745 | 108545 |
| NZ_FIKU01000001.1 | 98.876 | 801 | 9 | 0 | 20681 | 19881 |
| NZ_FIKZ01000024.1 | 98.876 | 801 | 9 | 0 | 20573 | 19773 |
| NZ_FIHV01000005.1 | 98.876 | 801 | 9 | 0 | 111026 | 111826 |
| NZ_FIJE01000020.1 | 98.876 | 801 | 9 | 0 | 14667 | 15467 |
| NZ_FILU01000003.1 | 98.876 | 801 | 9 | 0 | 20667 | 19867 |
| NZ_FIFX01000005.1 | 98.876 | 801 | 9 | 0 | 20681 | 19881 |
| NZ_FIJZ01000001.1 | 98.876 | 801 | 9 | 0 | 20681 | 19881 |
| NZ_FIHU01000004.1 | 98.876 | 801 | 9 | 0 | 107743 | 108543 |
| NZ_FIIL01000001.1 | 98.876 | 801 | 9 | 0 | 20667 | 19867 |
| NZ_FIFL01000002.1 | 98.876 | 801 | 9 | 0 | 108741 | 109541 |
| NZ_CZGV01000003.1 | 98.876 | 801 | 9 | 0 | 30122 | 29322 |
| NZ_CZFE01000055.1 | 98.876 | 801 | 9 | 0 | 29900 | 30700 |
| NZ_CZGK01000007.1 | 98.876 | 801 | 9 | 0 | 29781 | 30581 |
| NZ_CZGQ01000002.1 | 98.876 | 801 | 9 | 0 | 30166 | 29366 |
| NZ_CZDP01000024.1 | 98.876 | 801 | 9 | 0 | 29898 | 30698 |
| NZ_CZFZ01000071.1 | 98.876 | 801 | 9 | 0 | 20662 | 19862 |
| NZ_CZFV01000020.1 | 98.876 | 801 | 9 | 0 | 17434 | 18234 |
| NZ_CZEC01000066.1 | 98.876 | 801 | 9 | 0 | 29819 | 30619 |
| NZ_CZEB01000021.1 | 98.876 | 801 | 9 | 0 | 17434 | 18234 |
| NZ_CZFF01000017.1 | 98.876 | 801 | 9 | 0 | 14182 | 14982 |
| NZ_CZGH01000010.1 | 98.876 | 801 | 9 | 0 | 34660 | 33860 |
| NZ_CZEZ01000004.1 | 98.876 | 801 | 9 | 0 | 44369 | 43569 |
| NZ_CZGL01000022.1 | 98.876 | 801 | 9 | 0 | 3875 | 4675 |
| NZ_CZFN01000018.1 | 98.876 | 801 | 9 | 0 | 3875 | 4675 |
| NZ_CZEP01000023.1 | 98.876 | 801 | 9 | 0 | 30122 | 29322 |
| NZ_CZGF01000043.1 | 98.876 | 801 | 9 | 0 | 29796 | 30596 |
| NZ_CZFQ01000015.1 | 98.876 | 801 | 9 | 0 | 20664 | 19864 |
| NZ_CZEN01000003.1 | 98.876 | 801 | 9 | 0 | 44369 | 43569 |
| NZ_CZGC01000001.1 | 98.876 | 801 | 9 | 0 | 15730 | 14930 |
| NZ_CZGG01000033.1 | 98.876 | 801 | 9 | 0 | 29797 | 30597 |
| NZ_CZFS01000004.1 | 98.876 | 801 | 9 | 0 | 44369 | 43569 |
| NZ_CZFM01000005.1 | 98.876 | 801 | 9 | 0 | 44369 | 43569 |
| NZ_CZFJ01000005.1 | 98.876 | 801 | 9 | 0 | 4682 | 5482 |
| NZ_CZFD01000001.1 | 98.876 | 801 | 9 | 0 | 44369 | 43569 |
| NZ_CZEX01000019.1 | 98.876 | 801 | 9 | 0 | 30122 | 29322 |
| NZ_CZEL01000016.1 | 98.876 | 801 | 9 | 0 | 3875 | 4675 |
| NZ_CZGB01000002.1 | 98.876 | 801 | 9 | 0 | 30116 | 29316 |
| NZ_CZGW01000023.1 | 98.876 | 801 | 9 | 0 | 14344 | 15144 |
| NZ_CZGS01000032.1 | 98.876 | 801 | 9 | 0 | 3870 | 4670 |
| NZ_CZGO01000017.1 | 98.876 | 801 | 9 | 0 | 4814 | 5614 |
| NZ_CZGN01000003.1 | 98.876 | 801 | 9 | 0 | 44369 | 43569 |
| NZ_CZGE01000019.1 | 98.876 | 801 | 9 | 0 | 30166 | 29366 |
| NZ_CZGD01000015.1 | 98.876 | 801 | 9 | 0 | 20661 | 19861 |
| NZ_CZFX01000001.1 | 98.876 | 801 | 9 | 0 | 29899 | 30699 |
| NZ_CZEY01000062.1 | 98.876 | 801 | 9 | 0 | 188955 | 189755 |
| NZ_CP152119.1 | 98.876 | 801 | 9 | 0 | 1005168 | 1005968 |
| NZ_JBAPCO010000014.1 | 98.876 | 801 | 9 | 0 | 35145 | 35945 |
| NZ_JBAPDG010000006.1 | 98.876 | 801 | 9 | 0 | 20779 | 21579 |
| NZ_JAVIGI010000001.1 | 98.876 | 801 | 9 | 0 | 185083 | 185883 |
| NZ_JAVIGP010000010.1 | 98.876 | 801 | 9 | 0 | 52259 | 51459 |
| NZ_JAVIGR010000015.1 | 98.876 | 801 | 9 | 0 | 20675 | 19875 |
| NZ_JAVIGT010000002.1 | 98.876 | 801 | 9 | 0 | 36642 | 37442 |
| NZ_JAVIGY010000002.1 | 98.876 | 801 | 9 | 0 | 135289 | 136089 |
| NZ_JAVIGZ010000003.1 | 98.876 | 801 | 9 | 0 | 56576 | 57376 |
| NZ_JAVIHG010000002.1 | 98.876 | 801 | 9 | 0 | 128379 | 127579 |
| NZ_JAVIHF010000001.1 | 98.876 | 801 | 9 | 0 | 30054 | 30854 |
| NZ_JAVIHL010000015.1 | 98.876 | 801 | 9 | 0 | 14729 | 15529 |
| NZ_JAVIHN010000014.1 | 98.876 | 801 | 9 | 0 | 14729 | 15529 |
| NZ_JAVIHQ010000001.1 | 98.876 | 801 | 9 | 0 | 220683 | 219883 |
| NZ_JAVIHY010000002.1 | 98.876 | 801 | 9 | 0 | 128230 | 127430 |
| NZ_JAVIIC010000017.1 | 98.876 | 801 | 9 | 0 | 30032 | 30832 |
| NZ_JAVIIF010000001.1 | 98.876 | 801 | 9 | 0 | 36634 | 37434 |
| NZ_JAVIGB010000001.1 | 98.876 | 801 | 9 | 0 | 20675 | 19875 |
| NZ_JAZDUS010000017.1 | 98.876 | 801 | 9 | 0 | 1511 | 2311 |
| NZ_CP139878.1 | 98.876 | 801 | 9 | 0 | 2230872 | 2230072 |
| NZ_CP139876.1 | 98.876 | 801 | 9 | 0 | 638852 | 639652 |
| NZ_JAXKWQ010000001.1 | 98.876 | 801 | 9 | 0 | 1067828 | 1068628 |
| NZ_JAWWZL010000041.1 | 98.876 | 801 | 9 | 0 | 14694 | 15494 |
| NZ_JAWWZI010000023.1 | 98.876 | 801 | 9 | 0 | 20254 | 19454 |
| NZ_JAWWZH010000015.1 | 98.876 | 801 | 9 | 0 | 29721 | 30521 |
| NZ_JAUTIF010000021.1 | 98.876 | 801 | 9 | 0 | 13526 | 14326 |
| NZ_JAUTIE010000001.1 | 98.876 | 801 | 9 | 0 | 259074 | 259874 |
| NZ_JAUTID010000001.1 | 98.876 | 801 | 9 | 0 | 188671 | 189471 |
| NZ_JAUTIC010000001.1 | 98.876 | 801 | 9 | 0 | 223794 | 224594 |
| NZ_JAUTIB010000001.1 | 98.876 | 801 | 9 | 0 | 20671 | 19871 |
| NZ_JAUTIA010000001.1 | 98.876 | 801 | 9 | 0 | 257925 | 258725 |
| NZ_JAUTHZ010000022.1 | 98.876 | 801 | 9 | 0 | 10857 | 11657 |
| NZ_JAUTHU010000001.1 | 98.876 | 801 | 9 | 0 | 3900 | 3100 |
| NZ_JAUTHS010000001.1 | 98.876 | 801 | 9 | 0 | 90075 | 89275 |
| NZ_JAUTHM010000010.1 | 98.876 | 801 | 9 | 0 | 63837 | 63037 |
| NZ_JAUTHH010000008.1 | 98.876 | 801 | 9 | 0 | 34716 | 33916 |
| NZ_JAUTHI010000004.1 | 98.876 | 801 | 9 | 0 | 29811 | 30611 |
| NZ_JAUTHG010000001.1 | 98.876 | 801 | 9 | 0 | 127975 | 127175 |
| NZ_JAUTGU010000001.1 | 98.876 | 801 | 9 | 0 | 189375 | 190175 |
| NZ_JAUTGL010000031.1 | 98.876 | 801 | 9 | 0 | 20695 | 19895 |
| NZ_JAUTGO010000001.1 | 98.876 | 801 | 9 | 0 | 20675 | 19875 |
| NZ_JAUTGM010000023.1 | 98.876 | 801 | 9 | 0 | 20695 | 19895 |
| NZ_JAUTGI010000021.1 | 98.876 | 801 | 9 | 0 | 14727 | 15527 |
| NZ_JAUTGJ010000004.1 | 98.876 | 801 | 9 | 0 | 47975 | 48775 |
| NZ_JAUTGK010000022.1 | 98.876 | 801 | 9 | 0 | 14729 | 15529 |
| NZ_JAUTGF010000021.1 | 98.876 | 801 | 9 | 0 | 20695 | 19895 |
| NZ_JAUTGD010000021.1 | 98.876 | 801 | 9 | 0 | 14728 | 15528 |
| NZ_JAUTGE010000018.1 | 98.876 | 801 | 9 | 0 | 36457 | 35657 |
| NZ_JAUTGC010000020.1 | 98.876 | 801 | 9 | 0 | 14728 | 15528 |
| NZ_JAUTGB010000021.1 | 98.876 | 801 | 9 | 0 | 14729 | 15529 |
| NZ_JAUTGA010000022.1 | 98.876 | 801 | 9 | 0 | 20675 | 19875 |
| NZ_JAUTFW010000029.1 | 98.876 | 801 | 9 | 0 | 20984 | 20184 |
| NZ_JAUTFX010000021.1 | 98.876 | 801 | 9 | 0 | 14728 | 15528 |
| NZ_JAUTFY010000022.1 | 98.876 | 801 | 9 | 0 | 14728 | 15528 |
| NZ_JAUTFV010000029.1 | 98.876 | 801 | 9 | 0 | 20984 | 20184 |
| NZ_JAUTFT010000003.1 | 98.876 | 801 | 9 | 0 | 61799 | 62599 |
| NZ_JAUTFS010000018.1 | 98.876 | 801 | 9 | 0 | 19167 | 18367 |
| NZ_JAUTFR010000004.1 | 98.876 | 801 | 9 | 0 | 14826 | 15626 |
| NZ_JAUTFQ010000003.1 | 98.876 | 801 | 9 | 0 | 80830 | 81630 |
| NZ_JAUTFO010000015.1 | 98.876 | 801 | 9 | 0 | 14828 | 15628 |
| NZ_JAUTFN010000006.1 | 98.876 | 801 | 9 | 0 | 13653 | 14453 |
| NZ_JAUTFP010000029.1 | 98.876 | 801 | 9 | 0 | 4734 | 5534 |
| NZ_JAUTFM010000003.1 | 98.876 | 801 | 9 | 0 | 19189 | 18389 |
| NZ_JAUTFK010000003.1 | 98.876 | 801 | 9 | 0 | 113642 | 112842 |
| NZ_JAUTFI010000009.1 | 98.876 | 801 | 9 | 0 | 53647 | 54447 |
| NZ_JAUTET010000001.1 | 98.876 | 801 | 9 | 0 | 20696 | 19896 |
| NZ_JAUTEM010000014.1 | 98.876 | 801 | 9 | 0 | 34602 | 33802 |
| NZ_JAUTEN010000014.1 | 98.876 | 801 | 9 | 0 | 18912 | 19712 |
| NZ_JAUTEI010000004.1 | 98.876 | 801 | 9 | 0 | 45678 | 46478 |
| NZ_CP134488.1 | 98.876 | 801 | 9 | 0 | 1249062 | 1249862 |
| NZ_JARATV010000009.1 | 98.876 | 801 | 9 | 0 | 29792 | 30592 |
| NZ_JARATS010000019.1 | 98.876 | 801 | 9 | 0 | 13536 | 14336 |
| NZ_JARATR010000008.1 | 98.876 | 801 | 9 | 0 | 20655 | 19855 |
| NZ_JARATN010000010.1 | 98.876 | 801 | 9 | 0 | 29839 | 30639 |
| NZ_JARASS010000002.1 | 98.876 | 801 | 9 | 0 | 296271 | 295471 |
| NZ_JASFZV010000001.1 | 98.876 | 801 | 9 | 0 | 20657 | 19857 |
| NZ_JAKTES010000001.1 | 98.876 | 801 | 9 | 0 | 15078 | 15878 |
| NZ_JAKTEX010000001.1 | 98.876 | 801 | 9 | 0 | 188647 | 189447 |
| NZ_JARBFM010000021.1 | 98.876 | 801 | 9 | 0 | 30787 | 29987 |
| NZ_JAKTAJ010000004.1 | 98.876 | 801 | 9 | 0 | 20643 | 19843 |
| NZ_CP110141.1 | 98.876 | 801 | 9 | 0 | 1042273 | 1041473 |
| NZ_CP095182.1 | 98.876 | 801 | 9 | 0 | 1234708 | 1235508 |
| NZ_CP102152.1 | 98.876 | 801 | 9 | 0 | 1179541 | 1180341 |
| NZ_CP102141.1 | 98.876 | 801 | 9 | 0 | 1122988 | 1123788 |
| NZ_CP102143.1 | 98.876 | 801 | 9 | 0 | 1239861 | 1240661 |
| NZ_CP102140.1 | 98.876 | 801 | 9 | 0 | 1148301 | 1149101 |
| NZ_CP102094.1 | 98.876 | 801 | 9 | 0 | 892574 | 891774 |
| NZ_JANILN010000001.1 | 98.876 | 801 | 9 | 0 | 193802 | 194602 |
| NZ_JANILI010000001.1 | 98.876 | 801 | 9 | 0 | 21885 | 21085 |
| NZ_JAMWEK010000005.1 | 98.876 | 801 | 9 | 0 | 2096 | 1296 |
| NZ_JAMWEO010000023.1 | 98.876 | 801 | 9 | 0 | 20254 | 19454 |
| NZ_CP101844.1 | 98.876 | 801 | 9 | 0 | 767163 | 766363 |
| NZ_JAMZBN010000008.1 | 98.876 | 801 | 9 | 0 | 20663 | 19863 |
| NZ_JAMZBQ010000023.1 | 98.876 | 801 | 9 | 0 | 14221 | 15021 |
| NZ_JAMZBG010000012.1 | 98.876 | 801 | 9 | 0 | 49925 | 50725 |
| NZ_JAMZBF010000018.1 | 98.876 | 801 | 9 | 0 | 20663 | 19863 |
| NZ_JAMZBH010000024.1 | 98.876 | 801 | 9 | 0 | 14172 | 14972 |
| NZ_JAMZBK010000009.1 | 98.876 | 801 | 9 | 0 | 49883 | 50683 |
| NZ_JAMZBI010000024.1 | 98.876 | 801 | 9 | 0 | 14260 | 15060 |
| NZ_JAMZBJ010000011.1 | 98.876 | 801 | 9 | 0 | 41577 | 42377 |
| NZ_JAMZBO010000024.1 | 98.876 | 801 | 9 | 0 | 20663 | 19863 |
| NZ_CP100418.1 | 98.876 | 801 | 9 | 0 | 1067731 | 1068531 |
| NZ_CP100330.1 | 98.876 | 801 | 9 | 0 | 1065401 | 1066201 |
| NZ_CP100328.1 | 98.876 | 801 | 9 | 0 | 1065414 | 1066214 |
| NZ_CP100326.1 | 98.876 | 801 | 9 | 0 | 1067726 | 1068526 |
| NZ_CP100336.1 | 98.876 | 801 | 9 | 0 | 1065399 | 1066199 |
| NZ_CP100334.1 | 98.876 | 801 | 9 | 0 | 1067740 | 1068540 |
| NZ_CP100332.1 | 98.876 | 801 | 9 | 0 | 1067687 | 1068487 |
| NZ_CP100338.1 | 98.876 | 801 | 9 | 0 | 1067740 | 1068540 |
| NZ_CP100340.1 | 98.876 | 801 | 9 | 0 | 1067698 | 1068498 |
| NZ_JAMWGQ010000011.1 | 98.876 | 801 | 9 | 0 | 29697 | 30497 |
| NZ_JAMWGP010000024.1 | 98.876 | 801 | 9 | 0 | 20947 | 20147 |
| NZ_JAMWGR010000025.1 | 98.876 | 801 | 9 | 0 | 20947 | 20147 |
| NZ_JAMWGS010000011.1 | 98.876 | 801 | 9 | 0 | 20663 | 19863 |
| NZ_JAMWGU010000024.1 | 98.876 | 801 | 9 | 0 | 20947 | 20147 |
| NZ_JAMWGT010000024.1 | 98.876 | 801 | 9 | 0 | 20947 | 20147 |
| NZ_JAMWGV010000011.1 | 98.876 | 801 | 9 | 0 | 20663 | 19863 |
| NZ_JAMDJJ010000006.1 | 98.876 | 801 | 9 | 0 | 20661 | 19861 |
| NZ_JAMDJK010000003.1 | 98.876 | 801 | 9 | 0 | 20643 | 19843 |
| NZ_JAMDJF010000001.1 | 98.876 | 801 | 9 | 0 | 247130 | 247930 |
| NZ_JAMDJG010000006.1 | 98.876 | 801 | 9 | 0 | 20643 | 19843 |
| NZ_JAMDJD010000007.1 | 98.876 | 801 | 9 | 0 | 21316 | 20516 |
| NZ_JAMDJC010000006.1 | 98.876 | 801 | 9 | 0 | 31936 | 31136 |
| NZ_JAMDIW010000036.1 | 98.876 | 801 | 9 | 0 | 982 | 182 |
| NZ_JAMDIQ010000003.1 | 98.876 | 801 | 9 | 0 | 107711 | 108511 |
| NZ_JAMDIR010000003.1 | 98.876 | 801 | 9 | 0 | 20587 | 19787 |
| NZ_JAMDIM010000025.1 | 98.876 | 801 | 9 | 0 | 13488 | 14288 |
| NZ_JAMDIK010000002.1 | 98.876 | 801 | 9 | 0 | 20641 | 19841 |
| NZ_JAMDIJ010000025.1 | 98.876 | 801 | 9 | 0 | 14692 | 15492 |
| NZ_JAMDHX010000004.1 | 98.876 | 801 | 9 | 0 | 21268 | 20468 |
| NZ_JAASFD010000010.1 | 98.876 | 801 | 9 | 0 | 259916 | 260716 |
| NZ_JAASFB010000008.1 | 98.876 | 801 | 9 | 0 | 20927 | 20127 |
| NZ_JAASEZ010000005.1 | 98.876 | 801 | 9 | 0 | 202085 | 202885 |
| NZ_JAASEV010000014.1 | 98.876 | 801 | 9 | 0 | 129884 | 129084 |
| NZ_JAASEU010000021.1 | 98.876 | 801 | 9 | 0 | 99411 | 100211 |
| NZ_JAASER010000028.1 | 98.876 | 801 | 9 | 0 | 74419 | 75219 |
| NZ_JAASES010000011.1 | 98.876 | 801 | 9 | 0 | 172862 | 172062 |
| NZ_JAASET010000008.1 | 98.876 | 801 | 9 | 0 | 222566 | 223366 |
| NZ_JAASEQ010000037.1 | 98.876 | 801 | 9 | 0 | 10629 | 11429 |
| NZ_JAASEN010000025.1 | 98.876 | 801 | 9 | 0 | 74067 | 74867 |
| NZ_JAASEM010000003.1 | 98.876 | 801 | 9 | 0 | 260209 | 261009 |
| NZ_JAASEL010000037.1 | 98.876 | 801 | 9 | 0 | 14485 | 13685 |
| NZ_JAASDU010000032.1 | 98.876 | 801 | 9 | 0 | 16448 | 17248 |
| NZ_JAASDQ010000006.1 | 98.876 | 801 | 9 | 0 | 77795 | 78595 |
| NZ_JAASDO010000018.1 | 98.876 | 801 | 9 | 0 | 30330 | 29530 |
| NZ_JAASDL010000013.1 | 98.876 | 801 | 9 | 0 | 21225 | 20425 |
| NZ_JAASDC010000007.1 | 98.876 | 801 | 9 | 0 | 30619 | 29819 |
| NZ_JAASDE010000006.1 | 98.876 | 801 | 9 | 0 | 97548 | 98348 |
| NZ_JAASDA010000004.1 | 98.876 | 801 | 9 | 0 | 21300 | 20500 |
| NZ_JAASCX010000002.1 | 98.876 | 801 | 9 | 0 | 60731 | 61531 |
| NZ_JAASCV010000030.1 | 98.876 | 801 | 9 | 0 | 1966 | 2766 |
| NZ_JAASCS010000002.1 | 98.876 | 801 | 9 | 0 | 129821 | 129021 |
| NZ_JAASCJ010000027.1 | 98.876 | 801 | 9 | 0 | 21195 | 20395 |
| NZ_JAASCH010000003.1 | 98.876 | 801 | 9 | 0 | 34166 | 33366 |
| NZ_JAASCC010000006.1 | 98.876 | 801 | 9 | 0 | 21272 | 20472 |
| NZ_JAASCA010000008.1 | 98.876 | 801 | 9 | 0 | 66249 | 65449 |
| NZ_JAASBZ010000019.1 | 98.876 | 801 | 9 | 0 | 15049 | 15849 |
| NZ_JAASBW010000001.1 | 98.876 | 801 | 9 | 0 | 260104 | 260904 |
| NZ_JAASBR010000011.1 | 98.876 | 801 | 9 | 0 | 21210 | 20410 |
| NZ_JAASBP010000010.1 | 98.876 | 801 | 9 | 0 | 20877 | 20077 |
| NZ_JAASBC010000018.1 | 98.876 | 801 | 9 | 0 | 21091 | 20291 |
| NZ_CP095162.1 | 98.876 | 801 | 9 | 0 | 1021096 | 1021896 |
| NZ_JAKTCG010000006.1 | 98.876 | 801 | 9 | 0 | 88356 | 89156 |
| NZ_JAKTAV010000026.1 | 98.876 | 801 | 9 | 0 | 13486 | 14286 |
| NZ_JAKTAI010000004.1 | 98.876 | 801 | 9 | 0 | 107768 | 108568 |
| NZ_JAKTAO010000004.1 | 98.876 | 801 | 9 | 0 | 107769 | 108569 |
| NZ_JAKTAP010000006.1 | 98.876 | 801 | 9 | 0 | 20643 | 19843 |
| NZ_JAKTBR010000004.1 | 98.876 | 801 | 9 | 0 | 34594 | 33794 |
| NZ_JAKTCM010000019.1 | 98.876 | 801 | 9 | 0 | 20665 | 19865 |
| NZ_JAKTCL010000007.1 | 98.876 | 801 | 9 | 0 | 65217 | 66017 |
| NZ_JAKTCN010000020.1 | 98.876 | 801 | 9 | 0 | 20661 | 19861 |
| NZ_JAKOFW010000021.1 | 98.876 | 801 | 9 | 0 | 37379 | 38179 |
| NZ_JAKOFY010000018.1 | 98.876 | 801 | 9 | 0 | 37379 | 38179 |
| NZ_JAKOFV010000018.1 | 98.876 | 801 | 9 | 0 | 1079 | 279 |
| NZ_JAKOFU010000021.1 | 98.876 | 801 | 9 | 0 | 37379 | 38179 |
| NZ_JAKOFS010000024.1 | 98.876 | 801 | 9 | 0 | 30422 | 31222 |
| NZ_JAKOFR010000021.1 | 98.876 | 801 | 9 | 0 | 37379 | 38179 |
| NZ_JAKOFH010000026.1 | 98.876 | 801 | 9 | 0 | 1472 | 672 |
| NZ_JAKOFB010000022.1 | 98.876 | 801 | 9 | 0 | 1472 | 672 |
| NZ_CP085085.1 | 98.876 | 801 | 9 | 0 | 1182455 | 1183255 |
| NZ_JAJDOQ010000001.1 | 98.876 | 801 | 9 | 0 | 810592 | 809792 |
| NZ_JAFLMN010000008.1 | 98.876 | 801 | 9 | 0 | 20768 | 19968 |
| NZ_JAFLMM010000013.1 | 98.876 | 801 | 9 | 0 | 50229 | 51029 |
| NZ_JAFLML010000010.1 | 98.876 | 801 | 9 | 0 | 50213 | 51013 |
| NZ_JAFLMK010000010.1 | 98.876 | 801 | 9 | 0 | 50211 | 51011 |
| NZ_JAFLMG010000004.1 | 98.876 | 801 | 9 | 0 | 21406 | 20606 |
| NZ_JAFLMH010000003.1 | 98.876 | 801 | 9 | 0 | 20736 | 19936 |
| NZ_JAFLME010000005.1 | 98.876 | 801 | 9 | 0 | 126447 | 127247 |
| NZ_JAFLMD010000005.1 | 98.876 | 801 | 9 | 0 | 126446 | 127246 |
| NZ_JAFLMF010000005.1 | 98.876 | 801 | 9 | 0 | 126447 | 127247 |
| NZ_JAFLMB010000022.1 | 98.876 | 801 | 9 | 0 | 14741 | 15541 |
| NZ_JAFLMC010000022.1 | 98.876 | 801 | 9 | 0 | 17153 | 17953 |
| NZ_JAFLLZ010000018.1 | 98.876 | 801 | 9 | 0 | 14742 | 15542 |
| NZ_JAFLMA010000008.1 | 98.876 | 801 | 9 | 0 | 29839 | 30639 |
| NZ_JAFLLX010000021.1 | 98.876 | 801 | 9 | 0 | 14745 | 15545 |
| NZ_JAFLLY010000022.1 | 98.876 | 801 | 9 | 0 | 14742 | 15542 |
| NZ_JAFLLW010000021.1 | 98.876 | 801 | 9 | 0 | 14744 | 15544 |
| NZ_JAFLLT010000020.1 | 98.876 | 801 | 9 | 0 | 20709 | 19909 |
| NZ_JAFLLV010000020.1 | 98.876 | 801 | 9 | 0 | 14745 | 15545 |
| NZ_JAFLLS010000012.1 | 98.876 | 801 | 9 | 0 | 29839 | 30639 |
| NZ_JAFLLU010000022.1 | 98.876 | 801 | 9 | 0 | 14745 | 15545 |
| NZ_JAFLLR010000021.1 | 98.876 | 801 | 9 | 0 | 14745 | 15545 |
| NZ_JAFHDQ010000006.1 | 98.876 | 801 | 9 | 0 | 113641 | 112841 |
| NZ_JAFHDN010000021.1 | 98.876 | 801 | 9 | 0 | 13940 | 14740 |
| NZ_JAFHDM010000021.1 | 98.876 | 801 | 9 | 0 | 13940 | 14740 |
| NZ_JAFHDL010000021.1 | 98.876 | 801 | 9 | 0 | 13940 | 14740 |
| NZ_JAFHDI010000012.1 | 98.876 | 801 | 9 | 0 | 20665 | 19865 |
| NZ_JAFHDG010000011.1 | 98.876 | 801 | 9 | 0 | 20665 | 19865 |
| NZ_JAFHDH010000010.1 | 98.876 | 801 | 9 | 0 | 49983 | 50783 |
| NZ_JAFHDE010000049.1 | 98.876 | 801 | 9 | 0 | 13453 | 14253 |
| NZ_JAFHDB010000038.1 | 98.876 | 801 | 9 | 0 | 1031 | 231 |
| NZ_JAFHCY010000038.1 | 98.876 | 801 | 9 | 0 | 1031 | 231 |
| NZ_JAHUUO010000010.1 | 98.876 | 801 | 9 | 0 | 49850 | 50650 |
| NZ_VZMX01000022.1 | 98.876 | 801 | 9 | 0 | 14716 | 15516 |
| NZ_VZMZ01000022.1 | 98.876 | 801 | 9 | 0 | 20665 | 19865 |
| NZ_VZND01000006.1 | 98.876 | 801 | 9 | 0 | 65240 | 66040 |
| NZ_VZNE01000002.1 | 98.876 | 801 | 9 | 0 | 114703 | 115503 |
| NZ_VZNG01000002.1 | 98.876 | 801 | 9 | 0 | 229013 | 229813 |
| NZ_VZNF01000006.1 | 98.876 | 801 | 9 | 0 | 65693 | 66493 |
| NZ_VZNO01000006.1 | 98.876 | 801 | 9 | 0 | 21324 | 20524 |
| NZ_VZNT01000018.1 | 98.876 | 801 | 9 | 0 | 14709 | 15509 |
| NZ_VZNX01000006.1 | 98.876 | 801 | 9 | 0 | 20685 | 19885 |
| NZ_VZOA01000001.1 | 98.876 | 801 | 9 | 0 | 247206 | 248006 |
| NZ_VZNZ01000001.1 | 98.876 | 801 | 9 | 0 | 20665 | 19865 |
| NZ_VZOC01000001.1 | 98.876 | 801 | 9 | 0 | 20665 | 19865 |
| NZ_VZMQ01000006.1 | 98.876 | 801 | 9 | 0 | 66162 | 66962 |
| NZ_VZNC01000007.1 | 98.876 | 801 | 9 | 0 | 63527 | 64327 |
| NZ_JAGUAL010000001.1 | 98.876 | 801 | 9 | 0 | 20564 | 19764 |
| NZ_JAGUAE010000007.1 | 98.876 | 801 | 9 | 0 | 90036 | 90836 |
| NZ_JAGUAK010000001.1 | 98.876 | 801 | 9 | 0 | 255498 | 256298 |
| NZ_JAGUAM010000004.1 | 98.876 | 801 | 9 | 0 | 20564 | 19764 |
| NZ_JAGUAN010000004.1 | 98.876 | 801 | 9 | 0 | 20564 | 19764 |
| NZ_JAGUAJ010000004.1 | 98.876 | 801 | 9 | 0 | 20564 | 19764 |
| NZ_JAGUAI010000007.1 | 98.876 | 801 | 9 | 0 | 90036 | 90836 |
| NZ_JAGUBW010000004.1 | 98.876 | 801 | 9 | 0 | 20564 | 19764 |
| NZ_JAGUBP010000004.1 | 98.876 | 801 | 9 | 0 | 107692 | 108492 |
| NZ_JAGUBK010000004.1 | 98.876 | 801 | 9 | 0 | 20564 | 19764 |
| NZ_JAGUBJ010000004.1 | 98.876 | 801 | 9 | 0 | 107691 | 108491 |
| NZ_JAGUBI010000004.1 | 98.876 | 801 | 9 | 0 | 20564 | 19764 |
| NZ_JAGUBL010000004.1 | 98.876 | 801 | 9 | 0 | 107691 | 108491 |
| NZ_JAGUBF010000006.1 | 98.876 | 801 | 9 | 0 | 20564 | 19764 |
| NZ_WACC01000019.1 | 98.876 | 801 | 9 | 0 | 16888 | 17688 |
| NZ_JAGFVH010000001.1 | 98.876 | 801 | 9 | 0 | 247102 | 247902 |
| NZ_CP071697.1 | 98.876 | 801 | 9 | 0 | 1046400 | 1045600 |
| NZ_JAFFHE010000006.1 | 98.876 | 801 | 9 | 0 | 20664 | 19864 |
| NZ_JAFEIW010000012.1 | 98.876 | 801 | 9 | 0 | 49880 | 50680 |
| NZ_JAFEIV010000012.1 | 98.876 | 801 | 9 | 0 | 49880 | 50680 |
| NZ_JAFEIX010000013.1 | 98.876 | 801 | 9 | 0 | 20663 | 19863 |
| NZ_JAFEIY010000010.1 | 98.876 | 801 | 9 | 0 | 20663 | 19863 |
| NZ_JAFEIZ010000012.1 | 98.876 | 801 | 9 | 0 | 49880 | 50680 |
| NZ_JAEUXH010000007.1 | 98.876 | 801 | 9 | 0 | 65719 | 66519 |
| NZ_JAEUXG010000004.1 | 98.876 | 801 | 9 | 0 | 107691 | 108491 |
| NZ_WCJE01000042.1 | 98.876 | 801 | 9 | 0 | 5569 | 6369 |
| NZ_BCEO01000020.1 | 98.876 | 801 | 9 | 0 | 20825 | 20025 |
| NZ_BCEN01000003.1 | 98.876 | 801 | 9 | 0 | 20636 | 19836 |
| NZ_BCEM01000003.1 | 98.876 | 801 | 9 | 0 | 138579 | 139379 |
| NZ_BCEJ01000016.1 | 98.876 | 801 | 9 | 0 | 189305 | 190105 |
| NZ_BCEH01000041.1 | 98.876 | 801 | 9 | 0 | 46041 | 46841 |
| NZ_BCEG01000003.1 | 98.876 | 801 | 9 | 0 | 46023 | 46823 |
| NZ_BCEF01000064.1 | 98.876 | 801 | 9 | 0 | 46309 | 47109 |
| NZ_BCEE01000017.1 | 98.876 | 801 | 9 | 0 | 188872 | 189672 |
| NZ_BCED01000020.1 | 98.876 | 801 | 9 | 0 | 20860 | 20060 |
| NZ_BCEC01000027.1 | 98.876 | 801 | 9 | 0 | 20896 | 20096 |
| NZ_BCEB01000011.1 | 98.876 | 801 | 9 | 0 | 20896 | 20096 |
| NZ_BCEA01000005.1 | 98.876 | 801 | 9 | 0 | 20642 | 19842 |
| NZ_BCDZ01000018.1 | 98.876 | 801 | 9 | 0 | 138969 | 139769 |
| NZ_BCDY01000021.1 | 98.876 | 801 | 9 | 0 | 13948 | 14748 |
| NZ_BCDX01000024.1 | 98.876 | 801 | 9 | 0 | 74008 | 74808 |
| NZ_BCDW01000030.1 | 98.876 | 801 | 9 | 0 | 46036 | 46836 |
| NZ_BCDV01000018.1 | 98.876 | 801 | 9 | 0 | 20634 | 19834 |
| NZ_BCDT01000052.1 | 98.876 | 801 | 9 | 0 | 133991 | 134791 |
| NZ_BCDS01000001.1 | 98.876 | 801 | 9 | 0 | 20930 | 20130 |
| NZ_BCDQ01000007.1 | 98.876 | 801 | 9 | 0 | 20864 | 20064 |
| NZ_BCDP01000029.1 | 98.876 | 801 | 9 | 0 | 14224 | 15024 |
| NZ_BCDO01000051.1 | 98.876 | 801 | 9 | 0 | 20880 | 20080 |
| NZ_BCDF01000008.1 | 98.876 | 801 | 9 | 0 | 20807 | 20007 |
| NZ_BCCX01000019.1 | 98.876 | 801 | 9 | 0 | 20896 | 20096 |
| NZ_BCCV01000013.1 | 98.876 | 801 | 9 | 0 | 20628 | 19828 |
| NZ_BCCT01000014.1 | 98.876 | 801 | 9 | 0 | 20792 | 19992 |
| NZ_BCCO01000054.1 | 98.876 | 801 | 9 | 0 | 1601 | 2401 |
| NZ_CP058741.1 | 98.876 | 801 | 9 | 0 | 1150481 | 1151281 |
| NZ_CP031377.1 | 98.876 | 801 | 9 | 0 | 1091784 | 1092584 |
| NZ_CP030020.1 | 98.876 | 801 | 9 | 0 | 1114647 | 1115447 |
| NZ_JABXEW010000001.1 | 98.876 | 801 | 9 | 0 | 180077 | 180877 |
| NZ_JABXEZ010000001.1 | 98.876 | 801 | 9 | 0 | 180077 | 180877 |
| NZ_JABXEV010000003.1 | 98.876 | 801 | 9 | 0 | 20585 | 19785 |
| NZ_JABXET010000006.1 | 98.876 | 801 | 9 | 0 | 107691 | 108491 |
| NZ_JABXEU010000006.1 | 98.876 | 801 | 9 | 0 | 20585 | 19785 |
| NZ_JABTZH010000015.1 | 98.876 | 801 | 9 | 0 | 66319 | 67119 |
| NZ_JABLFG010000021.1 | 98.876 | 801 | 9 | 0 | 7200 | 8000 |
| NZ_JABLFW010000019.1 | 98.876 | 801 | 9 | 0 | 1453 | 2253 |
| NZ_JABLGC010000004.1 | 98.876 | 801 | 9 | 0 | 20636 | 19836 |
| NZ_JABLGO010000013.1 | 98.876 | 801 | 9 | 0 | 28749 | 29549 |
| NZ_JABLGQ010000007.1 | 98.876 | 801 | 9 | 0 | 74370 | 75170 |
| NZ_JABLHL010000001.1 | 98.876 | 801 | 9 | 0 | 140422 | 141222 |
| NZ_JABLHQ010000006.1 | 98.876 | 801 | 9 | 0 | 20707 | 19907 |
| NZ_JABLHR010000004.1 | 98.876 | 801 | 9 | 0 | 20707 | 19907 |
| NZ_JABLIC010000003.1 | 98.876 | 801 | 9 | 0 | 20707 | 19907 |
| NZ_JABLIF010000001.1 | 98.876 | 801 | 9 | 0 | 203778 | 204578 |
| NZ_JABLIG010000004.1 | 98.876 | 801 | 9 | 0 | 107811 | 108611 |
| NZ_JABLIE010000004.1 | 98.876 | 801 | 9 | 0 | 109924 | 110724 |
| NZ_JABLIO010000001.1 | 98.876 | 801 | 9 | 0 | 21629 | 20829 |
| NZ_JABLIP010000001.1 | 98.876 | 801 | 9 | 0 | 204645 | 205445 |
| NZ_JABLIY010000011.1 | 98.876 | 801 | 9 | 0 | 16523 | 15723 |
| NZ_JABLFS010000001.1 | 98.876 | 801 | 9 | 0 | 21789 | 20989 |
| NZ_JABLJK010000020.1 | 98.876 | 801 | 9 | 0 | 20710 | 19910 |
| NZ_JABLGZ010000033.1 | 98.876 | 801 | 9 | 0 | 7802 | 8602 |
| NZ_JABLHU010000002.1 | 98.876 | 801 | 9 | 0 | 20707 | 19907 |
| NZ_JABLIK010000033.1 | 98.876 | 801 | 9 | 0 | 7122 | 7922 |
| NZ_JABLIJ010000031.1 | 98.876 | 801 | 9 | 0 | 7122 | 7922 |
| NZ_JABLIL010000006.1 | 98.876 | 801 | 9 | 0 | 20707 | 19907 |
| NZ_JABLGI010000001.1 | 98.876 | 801 | 9 | 0 | 252615 | 253415 |
| NZ_JABLJC010000020.1 | 98.876 | 801 | 9 | 0 | 20710 | 19910 |
| NZ_JABLGY010000031.1 | 98.876 | 801 | 9 | 0 | 22118 | 21318 |
| NZ_JABLJO010000004.1 | 98.876 | 801 | 9 | 0 | 20288 | 19488 |
| NZ_JABLJR010000020.1 | 98.876 | 801 | 9 | 0 | 14739 | 15539 |
| NZ_JABLJP010000004.1 | 98.876 | 801 | 9 | 0 | 20919 | 20119 |
| NZ_JABLJW010000020.1 | 98.876 | 801 | 9 | 0 | 14740 | 15540 |
| NZ_JABLKC010000001.1 | 98.876 | 801 | 9 | 0 | 253588 | 254388 |
| NZ_JABLKE010000004.1 | 98.876 | 801 | 9 | 0 | 20919 | 20119 |
| NZ_JABLJZ010000003.1 | 98.876 | 801 | 9 | 0 | 21239 | 20439 |
| NZ_JABLKF010000001.1 | 98.876 | 801 | 9 | 0 | 253568 | 254368 |
| NZ_JABLKX010000006.1 | 98.876 | 801 | 9 | 0 | 102393 | 103193 |
| NZ_JABLLZ010000025.1 | 98.876 | 801 | 9 | 0 | 20193 | 19393 |
| NZ_JABLME010000011.1 | 98.876 | 801 | 9 | 0 | 61014 | 60214 |
| NZ_JABLKJ010000004.1 | 98.876 | 801 | 9 | 0 | 20811 | 20011 |
| NZ_JABLMZ010000002.1 | 98.876 | 801 | 9 | 0 | 189620 | 188820 |
| NZ_JABLNA010000002.1 | 98.876 | 801 | 9 | 0 | 188835 | 188035 |
| NZ_JABLNK010000020.1 | 98.876 | 801 | 9 | 0 | 1561 | 2361 |
| NZ_JABLNZ010000022.1 | 98.876 | 801 | 9 | 0 | 1561 | 2361 |
| NZ_JABLOE010000021.1 | 98.876 | 801 | 9 | 0 | 1561 | 2361 |
| NZ_JABLOG010000031.1 | 98.876 | 801 | 9 | 0 | 20684 | 19884 |
| NZ_JABLMN010000001.1 | 98.876 | 801 | 9 | 0 | 101678 | 100878 |
| NZ_JABLPI010000019.1 | 98.876 | 801 | 9 | 0 | 1561 | 2361 |
| NZ_JABLMJ010000001.1 | 98.876 | 801 | 9 | 0 | 101678 | 100878 |
| NZ_JABLPJ010000017.1 | 98.876 | 801 | 9 | 0 | 1561 | 2361 |
| NZ_JABLPK010000019.1 | 98.876 | 801 | 9 | 0 | 1561 | 2361 |
| NZ_JABLPN010000018.1 | 98.876 | 801 | 9 | 0 | 1561 | 2361 |
| NZ_JABLPP010000020.1 | 98.876 | 801 | 9 | 0 | 1487 | 2287 |
| NZ_JABKSE010000001.1 | 98.876 | 801 | 9 | 0 | 333894 | 334694 |
| NZ_JABKRO010000002.1 | 98.876 | 801 | 9 | 0 | 129474 | 128674 |
| NZ_JABKSX010000006.1 | 98.876 | 801 | 9 | 0 | 69100 | 69900 |
| NZ_JABKSY010000001.1 | 98.876 | 801 | 9 | 0 | 201798 | 202598 |
| NZ_JABKTD010000016.1 | 98.876 | 801 | 9 | 0 | 30561 | 29761 |
| NZ_JABKTK010000002.1 | 98.876 | 801 | 9 | 0 | 41194 | 41994 |
| NZ_JABKTP010000002.1 | 98.876 | 801 | 9 | 0 | 21629 | 20829 |
| NZ_JABKTT010000004.1 | 98.876 | 801 | 9 | 0 | 20711 | 19911 |
| NZ_JABKTS010000011.1 | 98.876 | 801 | 9 | 0 | 66237 | 67037 |
| NZ_JABKUC010000009.1 | 98.876 | 801 | 9 | 0 | 73800 | 74600 |
| NZ_JABKUE010000001.1 | 98.876 | 801 | 9 | 0 | 20686 | 19886 |
| NZ_JABKTV010000004.1 | 98.876 | 801 | 9 | 0 | 106364 | 107164 |
| NZ_JABKUF010000004.1 | 98.876 | 801 | 9 | 0 | 16077 | 16877 |
| NZ_JABKUK010000011.1 | 98.876 | 801 | 9 | 0 | 20659 | 19859 |
| NZ_JABKUX010000004.1 | 98.876 | 801 | 9 | 0 | 21066 | 20266 |
| NZ_JABKUY010000001.1 | 98.876 | 801 | 9 | 0 | 199983 | 200783 |
| NZ_JABKVA010000001.1 | 98.876 | 801 | 9 | 0 | 208452 | 209252 |
| NZ_JABKUZ010000003.1 | 98.876 | 801 | 9 | 0 | 129857 | 129057 |
| NZ_JABKVD010000003.1 | 98.876 | 801 | 9 | 0 | 20707 | 19907 |
| NZ_JABKVG010000018.1 | 98.876 | 801 | 9 | 0 | 20735 | 19935 |
| NZ_JABKVI010000024.1 | 98.876 | 801 | 9 | 0 | 13532 | 14332 |
| NZ_JABKTI010000011.1 | 98.876 | 801 | 9 | 0 | 50029 | 50829 |
| NZ_JABKTY010000010.1 | 98.876 | 801 | 9 | 0 | 20652 | 19852 |
| NZ_JABKWN010000002.1 | 98.876 | 801 | 9 | 0 | 211892 | 211092 |
| NZ_JABKUT010000008.1 | 98.876 | 801 | 9 | 0 | 20630 | 19830 |
| NZ_JABKUW010000005.1 | 98.876 | 801 | 9 | 0 | 20707 | 19907 |
| NZ_JABKVL010000030.1 | 98.876 | 801 | 9 | 0 | 3650 | 4450 |
| NZ_JABKVK010000010.1 | 98.876 | 801 | 9 | 0 | 20636 | 19836 |
| NZ_JABKVN010000001.1 | 98.876 | 801 | 9 | 0 | 140122 | 140922 |
| NZ_JABKVO010000011.1 | 98.876 | 801 | 9 | 0 | 20688 | 19888 |
| NZ_JABKVS010000003.1 | 98.876 | 801 | 9 | 0 | 20705 | 19905 |
| NZ_JABKVR010000001.1 | 98.876 | 801 | 9 | 0 | 253586 | 254386 |
| NZ_JABKVU010000008.1 | 98.876 | 801 | 9 | 0 | 29788 | 30588 |
| NZ_JABKVV010000014.1 | 98.876 | 801 | 9 | 0 | 20659 | 19859 |
| NZ_JABKWH010000006.1 | 98.876 | 801 | 9 | 0 | 20707 | 19907 |
| NZ_JABKWJ010000001.1 | 98.876 | 801 | 9 | 0 | 194812 | 195612 |
| NZ_JABKWI010000002.1 | 98.876 | 801 | 9 | 0 | 20884 | 20084 |
| NZ_JABKWM010000002.1 | 98.876 | 801 | 9 | 0 | 16078 | 16878 |
| NZ_JABKWT010000001.1 | 98.876 | 801 | 9 | 0 | 228567 | 229367 |
| NZ_JABKXL010000016.1 | 98.876 | 801 | 9 | 0 | 30512 | 29712 |
| NZ_JABKXM010000002.1 | 98.876 | 801 | 9 | 0 | 61691 | 62491 |
| NZ_JABKXO010000016.1 | 98.876 | 801 | 9 | 0 | 21507 | 22307 |
| NZ_JABKXQ010000019.1 | 98.876 | 801 | 9 | 0 | 1513 | 2313 |
| NZ_JABKYL010000003.1 | 98.876 | 801 | 9 | 0 | 20707 | 19907 |
| NZ_JABKUS010000016.1 | 98.876 | 801 | 9 | 0 | 30509 | 29709 |
| NZ_JABKYM010000004.1 | 98.876 | 801 | 9 | 0 | 20710 | 19910 |
| NZ_JABKVX010000008.1 | 98.876 | 801 | 9 | 0 | 20688 | 19888 |
| NZ_JABKWA010000011.1 | 98.876 | 801 | 9 | 0 | 20628 | 19828 |
| NZ_JABKZO010000013.1 | 98.876 | 801 | 9 | 0 | 20638 | 19838 |
| NZ_JABKWQ010000001.1 | 98.876 | 801 | 9 | 0 | 266678 | 267478 |
| NZ_JABKWR010000001.1 | 98.876 | 801 | 9 | 0 | 20710 | 19910 |
| NZ_JABKZQ010000051.1 | 98.876 | 801 | 9 | 0 | 10744 | 11544 |
| NZ_JABKZS010000026.1 | 98.876 | 801 | 9 | 0 | 7074 | 7874 |
| NZ_JABKZT010000027.1 | 98.876 | 801 | 9 | 0 | 20637 | 19837 |
| NZ_JABKXY010000017.1 | 98.876 | 801 | 9 | 0 | 1513 | 2313 |
| NZ_JABKXX010000002.1 | 98.876 | 801 | 9 | 0 | 201757 | 200957 |
| NZ_JABLAJ010000004.1 | 98.876 | 801 | 9 | 0 | 108741 | 109541 |
| NZ_JABLAI010000005.1 | 98.876 | 801 | 9 | 0 | 108741 | 109541 |
| NZ_JABKZG010000002.1 | 98.876 | 801 | 9 | 0 | 20707 | 19907 |
| NZ_JABKZD010000001.1 | 98.876 | 801 | 9 | 0 | 253577 | 254377 |
| NZ_JABKZF010000002.1 | 98.876 | 801 | 9 | 0 | 22142 | 21342 |
| NZ_JABKZC010000001.1 | 98.876 | 801 | 9 | 0 | 253577 | 254377 |
| NZ_JABKZA010000001.1 | 98.876 | 801 | 9 | 0 | 21629 | 20829 |
| NZ_JABKZB010000001.1 | 98.876 | 801 | 9 | 0 | 253577 | 254377 |
| NZ_JABKZE010000001.1 | 98.876 | 801 | 9 | 0 | 253825 | 254625 |
| NZ_JABKZW010000001.1 | 98.876 | 801 | 9 | 0 | 20634 | 19834 |
| NZ_JABLAK010000002.1 | 98.876 | 801 | 9 | 0 | 20657 | 19857 |
| NZ_JABLBB010000001.1 | 98.876 | 801 | 9 | 0 | 53858 | 53058 |
| NZ_JABLBM010000024.1 | 98.876 | 801 | 9 | 0 | 7072 | 7872 |
| NZ_JABLAN010000003.1 | 98.876 | 801 | 9 | 0 | 20635 | 19835 |
| NZ_JABLAO010000003.1 | 98.876 | 801 | 9 | 0 | 20634 | 19834 |
| NZ_JABLBS010000003.1 | 98.876 | 801 | 9 | 0 | 107759 | 108559 |
| NZ_JABLCC010000004.1 | 98.876 | 801 | 9 | 0 | 108741 | 109541 |
| NZ_JABLCD010000003.1 | 98.876 | 801 | 9 | 0 | 20635 | 19835 |
| NZ_JABLCP010000030.1 | 98.876 | 801 | 9 | 0 | 7072 | 7872 |
| NZ_JABLCI010000003.1 | 98.876 | 801 | 9 | 0 | 20660 | 19860 |
| NZ_JABLCR010000028.1 | 98.876 | 801 | 9 | 0 | 7072 | 7872 |
| NZ_JABLCQ010000027.1 | 98.876 | 801 | 9 | 0 | 7072 | 7872 |
| NZ_JABLCS010000003.1 | 98.876 | 801 | 9 | 0 | 107764 | 108564 |
| NZ_JABLCT010000006.1 | 98.876 | 801 | 9 | 0 | 79580 | 80380 |
| NZ_JABLCO010000027.1 | 98.876 | 801 | 9 | 0 | 15770 | 14970 |
| NZ_JABLDG010000025.1 | 98.876 | 801 | 9 | 0 | 14067 | 14867 |
| NZ_JABLDL010000004.1 | 98.876 | 801 | 9 | 0 | 20635 | 19835 |
| NZ_JABLDN010000013.1 | 98.876 | 801 | 9 | 0 | 22467 | 23267 |
| NZ_JABLCJ010000008.1 | 98.876 | 801 | 9 | 0 | 3660 | 4460 |
| NZ_JABLDP010000002.1 | 98.876 | 801 | 9 | 0 | 20660 | 19860 |
| NZ_JABLDZ010000008.1 | 98.876 | 801 | 9 | 0 | 53015 | 53815 |
| NZ_JABLDY010000013.1 | 98.876 | 801 | 9 | 0 | 17430 | 18230 |
| NZ_JABLET010000024.1 | 98.876 | 801 | 9 | 0 | 7965 | 8765 |
| NZ_JABLEX010000026.1 | 98.876 | 801 | 9 | 0 | 7198 | 7998 |
| NZ_JABLEV010000019.1 | 98.876 | 801 | 9 | 0 | 20660 | 19860 |
| NZ_JABLEA010000014.1 | 98.876 | 801 | 9 | 0 | 50061 | 50861 |
| NZ_JABLDV010000020.1 | 98.876 | 801 | 9 | 0 | 34568 | 35368 |
| NZ_JABLDU010000022.1 | 98.876 | 801 | 9 | 0 | 2998 | 2198 |
| NZ_JABLEI010000002.1 | 98.876 | 801 | 9 | 0 | 20882 | 20082 |
| NZ_JABLEL010000001.1 | 98.876 | 801 | 9 | 0 | 20660 | 19860 |
| NZ_JABCRU010000030.1 | 98.876 | 801 | 9 | 0 | 20661 | 19861 |
| NZ_JABCRS010000005.1 | 98.876 | 801 | 9 | 0 | 65691 | 66491 |
| NZ_VKJM01000009.1 | 98.876 | 801 | 9 | 0 | 21092 | 20292 |
| NZ_CP041994.1 | 98.876 | 801 | 9 | 0 | 221631 | 220831 |
| NZ_VIEH01000003.1 | 98.876 | 801 | 9 | 0 | 138350 | 139150 |
| NZ_RSDN01000001.1 | 98.876 | 801 | 9 | 0 | 20661 | 19861 |
| NZ_RSDL01000008.1 | 98.876 | 801 | 9 | 0 | 5894 | 5094 |
| NZ_RSDE01000002.1 | 98.876 | 801 | 9 | 0 | 20641 | 19841 |
| NZ_CP025419.1 | 98.876 | 801 | 9 | 0 | 1233667 | 1234467 |
| NZ_POPW01000093.1 | 98.876 | 801 | 9 | 0 | 36331 | 37131 |
| NZ_POPP01000288.1 | 98.876 | 801 | 9 | 0 | 66091 | 66891 |
| NZ_POPM01000680.1 | 98.876 | 801 | 9 | 0 | 66091 | 66891 |
| NZ_POQC01000165.1 | 98.876 | 801 | 9 | 0 | 45775 | 44975 |
| NZ_POPR01000027.1 | 98.876 | 801 | 9 | 0 | 66091 | 66891 |
| NZ_POPV01000030.1 | 98.876 | 801 | 9 | 0 | 20679 | 19879 |
| NZ_POPS01000029.1 | 98.876 | 801 | 9 | 0 | 40240 | 39440 |
| NZ_POPQ01000032.1 | 98.876 | 801 | 9 | 0 | 66091 | 66891 |
| NZ_POPF01000290.1 | 98.876 | 801 | 9 | 0 | 5101 | 5901 |
| NZ_PONV01000064.1 | 98.876 | 801 | 9 | 0 | 28849 | 29649 |
| NZ_PONS01000047.1 | 98.876 | 801 | 9 | 0 | 28849 | 29649 |
| NZ_PONW01000046.1 | 98.876 | 801 | 9 | 0 | 28849 | 29649 |
| NZ_PONT01000053.1 | 98.876 | 801 | 9 | 0 | 28849 | 29649 |
| NZ_POLQ01000093.1 | 98.876 | 801 | 9 | 0 | 36097 | 36897 |
| NZ_PONU01000056.1 | 98.876 | 801 | 9 | 0 | 28849 | 29649 |
| NZ_PONP01000033.1 | 98.876 | 801 | 9 | 0 | 29914 | 29114 |
| NZ_PONR01000042.1 | 98.876 | 801 | 9 | 0 | 28849 | 29649 |
| NZ_PONO01000483.1 | 98.876 | 801 | 9 | 0 | 28849 | 29649 |
| NZ_POLR01000040.1 | 98.876 | 801 | 9 | 0 | 21675 | 20875 |
| NZ_POKZ01000113.1 | 98.876 | 801 | 9 | 0 | 29890 | 29090 |
| NZ_POJI01000034.1 | 98.876 | 801 | 9 | 0 | 61901 | 61101 |
| NZ_POJB01000071.1 | 98.876 | 801 | 9 | 0 | 46109 | 46909 |
| NZ_ASBO01000002.1 | 98.876 | 801 | 9 | 0 | 12595 | 13395 |
| NZ_CP024126.1 | 98.876 | 801 | 9 | 0 | 1203575 | 1204375 |
| NZ_CP012731.1 | 98.876 | 801 | 9 | 0 | 2115615 | 2116415 |
| NZ_CP012911.1 | 98.876 | 801 | 9 | 0 | 1114876 | 1115676 |
| NZ_LDOH01000051.1 | 98.876 | 801 | 9 | 0 | 20872 | 20072 |
| NZ_LDOI01000057.1 | 98.876 | 801 | 9 | 0 | 2886 | 2086 |
| NZ_CP011419.1 | 98.876 | 801 | 9 | 0 | 1242562 | 1243362 |
| NZ_CEIB01000005.1 | 98.876 | 801 | 9 | 0 | 14378 | 13578 |
| NZ_CEID01000022.1 | 98.876 | 801 | 9 | 0 | 20856 | 20056 |
| NZ_CEEM01000008.1 | 98.876 | 801 | 9 | 0 | 20610 | 19810 |
| NZ_CEIV01000062.1 | 98.876 | 801 | 9 | 0 | 34549 | 35349 |
| NZ_CEHS01000011.1 | 98.876 | 801 | 9 | 0 | 107749 | 108549 |
| NZ_CEIO01000097.1 | 98.876 | 801 | 9 | 0 | 21191 | 20391 |
| NZ_CEGU01000004.1 | 98.876 | 801 | 9 | 0 | 20620 | 19820 |
| NZ_CEET01000015.1 | 98.876 | 801 | 9 | 0 | 20616 | 19816 |
| NZ_CEEG01000016.1 | 98.876 | 801 | 9 | 0 | 7055 | 7855 |
| NZ_CEDN01000063.1 | 98.876 | 801 | 9 | 0 | 1447 | 2247 |
| NZ_CEHT01000134.1 | 98.876 | 801 | 9 | 0 | 66035 | 66835 |
| NZ_CEDW01000048.1 | 98.876 | 801 | 9 | 0 | 20616 | 19816 |
| NZ_CEKR01000004.1 | 98.876 | 801 | 9 | 0 | 252595 | 253395 |
| NZ_CEIT01000027.1 | 98.876 | 801 | 9 | 0 | 7180 | 7980 |
| NZ_CECV01000002.1 | 98.876 | 801 | 9 | 0 | 20630 | 19830 |
| NZ_CECU01000004.1 | 98.876 | 801 | 9 | 0 | 73860 | 74660 |
| NZ_CEHH01000004.1 | 98.876 | 801 | 9 | 0 | 252596 | 253396 |
| NZ_CEIC01000108.1 | 98.876 | 801 | 9 | 0 | 4150 | 3350 |
| NZ_CEEE01000002.1 | 98.876 | 801 | 9 | 0 | 20620 | 19820 |
| NZ_CEIZ01000056.1 | 98.876 | 801 | 9 | 0 | 139859 | 140659 |
| NZ_CEKA01000038.1 | 98.876 | 801 | 9 | 0 | 1440 | 2240 |
| NZ_CECZ01000002.1 | 98.876 | 801 | 9 | 0 | 20630 | 19830 |
| NZ_CEEJ01000033.1 | 98.876 | 801 | 9 | 0 | 53844 | 53044 |
| NZ_CEHR01000022.1 | 98.876 | 801 | 9 | 0 | 20618 | 19818 |
| NZ_CEFE01000034.1 | 98.876 | 801 | 9 | 0 | 9863 | 10663 |
| NZ_CEFD01000045.1 | 98.876 | 801 | 9 | 0 | 20612 | 19812 |
| NZ_CEFB01000002.1 | 98.876 | 801 | 9 | 0 | 6172 | 5372 |
| NZ_CEDM01000005.1 | 98.876 | 801 | 9 | 0 | 107749 | 108549 |
| NZ_CECS01000166.1 | 98.876 | 801 | 9 | 0 | 21195 | 20395 |
| NZ_CEES01000042.1 | 98.876 | 801 | 9 | 0 | 7055 | 7855 |
| NZ_CEKG01000016.1 | 98.876 | 801 | 9 | 0 | 20622 | 19822 |
| NZ_CEHN01000044.1 | 98.876 | 801 | 9 | 0 | 20148 | 19348 |
| NZ_CELL01000069.1 | 98.876 | 801 | 9 | 0 | 29682 | 30482 |
| NZ_CEHY01000006.1 | 98.876 | 801 | 9 | 0 | 107739 | 108539 |
| NZ_CEJA01000009.1 | 98.876 | 801 | 9 | 0 | 20574 | 19774 |
| NZ_CEJW01000048.1 | 98.876 | 801 | 9 | 0 | 252595 | 253395 |
| NZ_CEJG01000021.1 | 98.876 | 801 | 9 | 0 | 53006 | 53806 |
| NZ_CEKL01000124.1 | 98.876 | 801 | 9 | 0 | 34965 | 35765 |
| NZ_CEJL01000006.1 | 98.876 | 801 | 9 | 0 | 43620 | 44420 |
| NZ_CEFA01000081.1 | 98.876 | 801 | 9 | 0 | 20626 | 19826 |
| NZ_CEEI01000073.1 | 98.876 | 801 | 9 | 0 | 17726 | 16926 |
| NZ_CEJT01000096.1 | 98.876 | 801 | 9 | 0 | 20618 | 19818 |
| NZ_CELN01000015.1 | 98.876 | 801 | 9 | 0 | 14674 | 15474 |
| NZ_CEKH01000004.1 | 98.876 | 801 | 9 | 0 | 19169 | 18369 |
| NZ_CP008921.1 | 98.876 | 801 | 9 | 0 | 1156650 | 1157450 |
| NC_022516.1 | 98.876 | 801 | 9 | 0 | 1100565 | 1101365 |
| NZ_ALNC01000010.1 | 98.876 | 801 | 9 | 0 | 49850 | 50650 |
| NZ_ALNA01000053.1 | 98.876 | 801 | 9 | 0 | 90834 | 91634 |
| NZ_ALMV01000095.1 | 98.876 | 801 | 9 | 0 | 19598 | 18798 |
| NZ_ALMU01000008.1 | 98.876 | 801 | 9 | 0 | 78191 | 78991 |
| NZ_ALML01000009.1 | 98.876 | 801 | 9 | 0 | 20788 | 19988 |
| NZ_ALMH01000069.1 | 98.876 | 801 | 9 | 0 | 19601 | 18801 |
| NZ_ALMF01000059.1 | 98.876 | 801 | 9 | 0 | 253038 | 253838 |
| NZ_ALMA01000027.1 | 98.876 | 801 | 9 | 0 | 221149 | 220349 |
| NZ_ALLU01000033.1 | 98.876 | 801 | 9 | 0 | 2204 | 3004 |
| NZ_ALLI01000017.1 | 98.876 | 801 | 9 | 0 | 14954 | 15754 |
| NZ_ALLF01000026.1 | 98.876 | 801 | 9 | 0 | 21049 | 20249 |
| NZ_ALLE01000002.1 | 98.876 | 801 | 9 | 0 | 40327 | 41127 |
| NZ_ALLD01000042.1 | 98.876 | 801 | 9 | 0 | 137209 | 138009 |
| NZ_ALKS01000005.1 | 98.876 | 801 | 9 | 0 | 40322 | 41122 |
| NC_017620.1 | 98.876 | 801 | 9 | 0 | 1226035 | 1226835 |
| NZ_CP002007.2 | 98.876 | 801 | 9 | 0 | 1109770 | 1108970 |
| LR738723.1 | 98.876 | 801 | 9 | 0 | 981525 | 980725 |
| FIIA01000004.1 | 98.876 | 801 | 9 | 0 | 20681 | 19881 |
| FIFF01000031.1 | 98.876 | 801 | 9 | 0 | 20681 | 19881 |
| FIFE01000002.1 | 98.876 | 801 | 9 | 0 | 125379 | 126179 |
| FIIY01000004.1 | 98.876 | 801 | 9 | 0 | 48814 | 49614 |
| FIKO01000016.1 | 98.876 | 801 | 9 | 0 | 15810 | 16610 |
| FIJU01000013.1 | 98.876 | 801 | 9 | 0 | 17447 | 18247 |
| FIGF01000004.1 | 98.876 | 801 | 9 | 0 | 107757 | 108557 |
| FIKP01000026.1 | 98.876 | 801 | 9 | 0 | 7222 | 8022 |
| FIKK01000024.1 | 98.876 | 801 | 9 | 0 | 7963 | 8763 |
| FIGC01000004.1 | 98.876 | 801 | 9 | 0 | 107677 | 108477 |
| FILK01000001.1 | 98.876 | 801 | 9 | 0 | 252617 | 253417 |
| FIII01000033.1 | 98.876 | 801 | 9 | 0 | 20652 | 19852 |
| FIFY01000005.1 | 98.876 | 801 | 9 | 0 | 20681 | 19881 |
| FIJB01000004.1 | 98.876 | 801 | 9 | 0 | 20616 | 19816 |
| FIHJ01000004.1 | 98.876 | 801 | 9 | 0 | 20616 | 19816 |
| FIHF01000031.1 | 98.876 | 801 | 9 | 0 | 7071 | 7871 |
| FIJG01000002.1 | 98.876 | 801 | 9 | 0 | 107748 | 108548 |
| FIIJ01000001.1 | 98.876 | 801 | 9 | 0 | 20667 | 19867 |
| FIFH01000003.1 | 98.876 | 801 | 9 | 0 | 62179 | 62979 |
| FIKE01000001.1 | 98.876 | 801 | 9 | 0 | 252623 | 253423 |
| FIIK01000031.1 | 98.876 | 801 | 9 | 0 | 7073 | 7873 |
| FIIH01000030.1 | 98.876 | 801 | 9 | 0 | 20658 | 19858 |
| FIJP01000009.1 | 98.876 | 801 | 9 | 0 | 20560 | 19760 |
| FIJS01000010.1 | 98.876 | 801 | 9 | 0 | 15789 | 14989 |
| FIFG01000006.1 | 98.876 | 801 | 9 | 0 | 20162 | 19362 |
| FIIF01000022.1 | 98.876 | 801 | 9 | 0 | 1457 | 2257 |
| FIIB01000026.1 | 98.876 | 801 | 9 | 0 | 20158 | 19358 |
| FIKD01000004.1 | 98.876 | 801 | 9 | 0 | 109920 | 110720 |
| FIJM01000019.1 | 98.876 | 801 | 9 | 0 | 3007 | 2207 |
| FIJL01000023.1 | 98.876 | 801 | 9 | 0 | 34452 | 35252 |
| FIGZ01000001.1 | 98.876 | 801 | 9 | 0 | 162814 | 163614 |
| FIMA01000001.1 | 98.876 | 801 | 9 | 0 | 252607 | 253407 |
| FILO01000017.1 | 98.876 | 801 | 9 | 0 | 30229 | 29429 |
| FIGE01000004.1 | 98.876 | 801 | 9 | 0 | 107745 | 108545 |
| FIKU01000001.1 | 98.876 | 801 | 9 | 0 | 20681 | 19881 |
| FIKZ01000024.1 | 98.876 | 801 | 9 | 0 | 20573 | 19773 |
| FIHV01000005.1 | 98.876 | 801 | 9 | 0 | 111026 | 111826 |
| FIJE01000020.1 | 98.876 | 801 | 9 | 0 | 14667 | 15467 |
| FILU01000003.1 | 98.876 | 801 | 9 | 0 | 20667 | 19867 |
| FIFX01000005.1 | 98.876 | 801 | 9 | 0 | 20681 | 19881 |
| FIJZ01000001.1 | 98.876 | 801 | 9 | 0 | 20681 | 19881 |
| FIHU01000004.1 | 98.876 | 801 | 9 | 0 | 107743 | 108543 |
| FIIL01000001.1 | 98.876 | 801 | 9 | 0 | 20667 | 19867 |
| FIFL01000002.1 | 98.876 | 801 | 9 | 0 | 108741 | 109541 |
| CZGV01000003.1 | 98.876 | 801 | 9 | 0 | 30122 | 29322 |
| CZFE01000055.1 | 98.876 | 801 | 9 | 0 | 29900 | 30700 |
| CZGK01000007.1 | 98.876 | 801 | 9 | 0 | 29781 | 30581 |
| CZGQ01000002.1 | 98.876 | 801 | 9 | 0 | 30166 | 29366 |
| CZDP01000024.1 | 98.876 | 801 | 9 | 0 | 29898 | 30698 |
| CZFZ01000071.1 | 98.876 | 801 | 9 | 0 | 20662 | 19862 |
| CZFV01000020.1 | 98.876 | 801 | 9 | 0 | 17434 | 18234 |
| CZEC01000066.1 | 98.876 | 801 | 9 | 0 | 29819 | 30619 |
| CZEB01000021.1 | 98.876 | 801 | 9 | 0 | 17434 | 18234 |
| CZFF01000017.1 | 98.876 | 801 | 9 | 0 | 14182 | 14982 |
| CZGH01000010.1 | 98.876 | 801 | 9 | 0 | 34660 | 33860 |
| CZEZ01000004.1 | 98.876 | 801 | 9 | 0 | 44369 | 43569 |
| CZGL01000022.1 | 98.876 | 801 | 9 | 0 | 3875 | 4675 |
| CZFN01000018.1 | 98.876 | 801 | 9 | 0 | 3875 | 4675 |
| CZEP01000023.1 | 98.876 | 801 | 9 | 0 | 30122 | 29322 |
| CZGF01000043.1 | 98.876 | 801 | 9 | 0 | 29796 | 30596 |
| CZFQ01000015.1 | 98.876 | 801 | 9 | 0 | 20664 | 19864 |
| CZEN01000003.1 | 98.876 | 801 | 9 | 0 | 44369 | 43569 |
| CZGC01000001.1 | 98.876 | 801 | 9 | 0 | 15730 | 14930 |
| CZGG01000033.1 | 98.876 | 801 | 9 | 0 | 29797 | 30597 |
| CZFS01000004.1 | 98.876 | 801 | 9 | 0 | 44369 | 43569 |
| CZFM01000005.1 | 98.876 | 801 | 9 | 0 | 44369 | 43569 |
| CZFJ01000005.1 | 98.876 | 801 | 9 | 0 | 4682 | 5482 |
| CZFD01000001.1 | 98.876 | 801 | 9 | 0 | 44369 | 43569 |
| CZEX01000019.1 | 98.876 | 801 | 9 | 0 | 30122 | 29322 |
| CZEL01000016.1 | 98.876 | 801 | 9 | 0 | 3875 | 4675 |
| CZGB01000002.1 | 98.876 | 801 | 9 | 0 | 30116 | 29316 |
| CZGW01000023.1 | 98.876 | 801 | 9 | 0 | 14344 | 15144 |
| CZGS01000032.1 | 98.876 | 801 | 9 | 0 | 3870 | 4670 |
| CZGO01000017.1 | 98.876 | 801 | 9 | 0 | 4814 | 5614 |
| CZGN01000003.1 | 98.876 | 801 | 9 | 0 | 44369 | 43569 |
| CZGE01000019.1 | 98.876 | 801 | 9 | 0 | 30166 | 29366 |
| CZGD01000015.1 | 98.876 | 801 | 9 | 0 | 20661 | 19861 |
| CZFX01000001.1 | 98.876 | 801 | 9 | 0 | 29899 | 30699 |
| CZEY01000062.1 | 98.876 | 801 | 9 | 0 | 188955 | 189755 |
| CP152119.1 | 98.876 | 801 | 9 | 0 | 1005168 | 1005968 |
| JBAPCO010000014.1 | 98.876 | 801 | 9 | 0 | 35145 | 35945 |
| JBAPDG010000006.1 | 98.876 | 801 | 9 | 0 | 20779 | 21579 |
| JAVIGI010000001.1 | 98.876 | 801 | 9 | 0 | 185083 | 185883 |
| JAVIGP010000010.1 | 98.876 | 801 | 9 | 0 | 52259 | 51459 |
| JAVIGR010000015.1 | 98.876 | 801 | 9 | 0 | 20675 | 19875 |
| JAVIGT010000002.1 | 98.876 | 801 | 9 | 0 | 36642 | 37442 |
| JAVIGY010000002.1 | 98.876 | 801 | 9 | 0 | 135289 | 136089 |
| JAVIGZ010000003.1 | 98.876 | 801 | 9 | 0 | 56576 | 57376 |
| JAVIHG010000002.1 | 98.876 | 801 | 9 | 0 | 128379 | 127579 |
| JAVIHF010000001.1 | 98.876 | 801 | 9 | 0 | 30054 | 30854 |
| JAVIHL010000015.1 | 98.876 | 801 | 9 | 0 | 14729 | 15529 |
| JAVIHN010000014.1 | 98.876 | 801 | 9 | 0 | 14729 | 15529 |
| JAVIHQ010000001.1 | 98.876 | 801 | 9 | 0 | 220683 | 219883 |
| JAVIHY010000002.1 | 98.876 | 801 | 9 | 0 | 128230 | 127430 |
| JAVIIC010000017.1 | 98.876 | 801 | 9 | 0 | 30032 | 30832 |
| JAVIIF010000001.1 | 98.876 | 801 | 9 | 0 | 36634 | 37434 |
| JAVIGB010000001.1 | 98.876 | 801 | 9 | 0 | 20675 | 19875 |
| JAZDUS010000017.1 | 98.876 | 801 | 9 | 0 | 1511 | 2311 |
| DATTWT010000004.1 | 98.876 | 801 | 9 | 0 | 20795 | 19995 |
| DATSSF010000001.1 | 98.876 | 801 | 9 | 0 | 20667 | 19867 |
| DATSSC010000011.1 | 98.876 | 801 | 9 | 0 | 20676 | 19876 |
| DATSRZ010000002.1 | 98.876 | 801 | 9 | 0 | 15532 | 14732 |
| DATSSB010000003.1 | 98.876 | 801 | 9 | 0 | 20795 | 19995 |
| DATSRU010000018.1 | 98.876 | 801 | 9 | 0 | 30036 | 29236 |
| DATSRV010000005.1 | 98.876 | 801 | 9 | 0 | 30036 | 29236 |
| DATSRS010000022.1 | 98.876 | 801 | 9 | 0 | 20563 | 19763 |
| CP139878.1 | 98.876 | 801 | 9 | 0 | 2230872 | 2230072 |
| CP139876.1 | 98.876 | 801 | 9 | 0 | 638852 | 639652 |
| JAXKWQ010000001.1 | 98.876 | 801 | 9 | 0 | 1067828 | 1068628 |
| DASGYW010000013.1 | 98.876 | 801 | 9 | 0 | 20515 | 19715 |
| DASGYT010000001.1 | 98.876 | 801 | 9 | 0 | 188580 | 189380 |
| DASGYJ010000005.1 | 98.876 | 801 | 9 | 0 | 20663 | 19863 |
| DASGYE010000005.1 | 98.876 | 801 | 9 | 0 | 20563 | 19763 |
| DASGYB010000006.1 | 98.876 | 801 | 9 | 0 | 20687 | 19887 |
| DASGXR010000019.1 | 98.876 | 801 | 9 | 0 | 20563 | 19763 |
| DASGXK010000004.1 | 98.876 | 801 | 9 | 0 | 20585 | 19785 |
| DASGXF010000001.1 | 98.876 | 801 | 9 | 0 | 188581 | 189381 |
| DASGXI010000005.1 | 98.876 | 801 | 9 | 0 | 113135 | 112335 |
| DASGWV010000022.1 | 98.876 | 801 | 9 | 0 | 20507 | 19707 |
| DASGVY010000001.1 | 98.876 | 801 | 9 | 0 | 20652 | 19852 |
| DASGVU010000001.1 | 98.876 | 801 | 9 | 0 | 20658 | 19858 |
| DASGUS010000005.1 | 98.876 | 801 | 9 | 0 | 20663 | 19863 |
| DASGUJ010000010.1 | 98.876 | 801 | 9 | 0 | 20639 | 19839 |
| DASGUH010000003.1 | 98.876 | 801 | 9 | 0 | 45003 | 44203 |
| DASGUC010000001.1 | 98.876 | 801 | 9 | 0 | 20667 | 19867 |
| DASGTY010000001.1 | 98.876 | 801 | 9 | 0 | 20667 | 19867 |
| DASGTX010000007.1 | 98.876 | 801 | 9 | 0 | 20522 | 19722 |
| DASGTT010000001.1 | 98.876 | 801 | 9 | 0 | 20667 | 19867 |
| DASGTO010000001.1 | 98.876 | 801 | 9 | 0 | 20667 | 19867 |
| DASGTQ010000008.1 | 98.876 | 801 | 9 | 0 | 20507 | 19707 |
| DASGTE010000002.1 | 98.876 | 801 | 9 | 0 | 20566 | 19766 |
| DASGSZ010000008.1 | 98.876 | 801 | 9 | 0 | 20507 | 19707 |
| DASGSU010000008.1 | 98.876 | 801 | 9 | 0 | 20507 | 19707 |
| DASGSK010000009.1 | 98.876 | 801 | 9 | 0 | 20526 | 19726 |
| DASGSG010000126.1 | 98.876 | 801 | 9 | 0 | 2165 | 2965 |
| DASGRP010000008.1 | 98.876 | 801 | 9 | 0 | 20507 | 19707 |
| DASGQZ010000001.1 | 98.876 | 801 | 9 | 0 | 188565 | 189365 |
| DASGQX010000019.1 | 98.876 | 801 | 9 | 0 | 20507 | 19707 |
| DASGQR010000008.1 | 98.876 | 801 | 9 | 0 | 20507 | 19707 |
| DASGQO010000008.1 | 98.876 | 801 | 9 | 0 | 20507 | 19707 |
| DASGQS010000008.1 | 98.876 | 801 | 9 | 0 | 20507 | 19707 |
| DASGQP010000007.1 | 98.876 | 801 | 9 | 0 | 20507 | 19707 |
| DASGQK010000001.1 | 98.876 | 801 | 9 | 0 | 20646 | 19846 |
| DASGQL010000008.1 | 98.876 | 801 | 9 | 0 | 20507 | 19707 |
| DASGQJ010000008.1 | 98.876 | 801 | 9 | 0 | 20507 | 19707 |
| DASGQB010000004.1 | 98.876 | 801 | 9 | 0 | 92668 | 91868 |
| DASGPU010000001.1 | 98.876 | 801 | 9 | 0 | 92668 | 91868 |
| DASGPI010000009.1 | 98.876 | 801 | 9 | 0 | 20639 | 19839 |
| DASGPJ010000016.1 | 98.876 | 801 | 9 | 0 | 20563 | 19763 |
| DASGPH010000009.1 | 98.876 | 801 | 9 | 0 | 20639 | 19839 |
| DASGPG010000003.1 | 98.876 | 801 | 9 | 0 | 105815 | 106615 |
| DASGOR010000013.1 | 98.876 | 801 | 9 | 0 | 20563 | 19763 |
| DASGNX010000001.1 | 98.876 | 801 | 9 | 0 | 190192 | 190992 |
| DASGNO010000001.1 | 98.876 | 801 | 9 | 0 | 188236 | 187436 |
| DASGMZ010000001.1 | 98.876 | 801 | 9 | 0 | 190192 | 190992 |
| DASGNA010000016.1 | 98.876 | 801 | 9 | 0 | 20563 | 19763 |
| DASGJA010000019.1 | 98.876 | 801 | 9 | 0 | 20639 | 19839 |
| DASGIX010000008.1 | 98.876 | 801 | 9 | 0 | 20563 | 19763 |
| DASGIQ010000030.1 | 98.876 | 801 | 9 | 0 | 20563 | 19763 |
| DASGIP010000031.1 | 98.876 | 801 | 9 | 0 | 20563 | 19763 |
| DASGID010000001.1 | 98.876 | 801 | 9 | 0 | 20566 | 19766 |
| DASGHW010000004.1 | 98.876 | 801 | 9 | 0 | 20583 | 19783 |
| DASGHX010000006.1 | 98.876 | 801 | 9 | 0 | 20563 | 19763 |
| DASGHT010000008.1 | 98.876 | 801 | 9 | 0 | 20563 | 19763 |
| DASGHL010000004.1 | 98.876 | 801 | 9 | 0 | 20583 | 19783 |
| DASGHJ010000004.1 | 98.876 | 801 | 9 | 0 | 20583 | 19783 |
| DASGHI010000004.1 | 98.876 | 801 | 9 | 0 | 20563 | 19763 |
| DASGHH010000001.1 | 98.876 | 801 | 9 | 0 | 20583 | 19783 |
| DASGHF010000032.1 | 98.876 | 801 | 9 | 0 | 20563 | 19763 |
| DASGHE010000033.1 | 98.876 | 801 | 9 | 0 | 20563 | 19763 |
| DASGHD010000004.1 | 98.876 | 801 | 9 | 0 | 20563 | 19763 |
| DASGGZ010000001.1 | 98.876 | 801 | 9 | 0 | 252624 | 253424 |
| DASGGY010000001.1 | 98.876 | 801 | 9 | 0 | 20563 | 19763 |
| DASGGQ010000009.1 | 98.876 | 801 | 9 | 0 | 20565 | 19765 |
| DASGGR010000033.1 | 98.876 | 801 | 9 | 0 | 865 | 65 |
| DASGGN010000018.1 | 98.876 | 801 | 9 | 0 | 20563 | 19763 |
| DASGGE010000018.1 | 98.876 | 801 | 9 | 0 | 14613 | 15413 |
| DASGFZ010000004.1 | 98.876 | 801 | 9 | 0 | 20563 | 19763 |
| DASGGA010000003.1 | 98.876 | 801 | 9 | 0 | 20164 | 19364 |
| DASGFX010000019.1 | 98.876 | 801 | 9 | 0 | 20633 | 19833 |
| DASGFT010000018.1 | 98.876 | 801 | 9 | 0 | 14613 | 15413 |
| DASGFQ010000003.1 | 98.876 | 801 | 9 | 0 | 20583 | 19783 |
| DASGFN010000001.1 | 98.876 | 801 | 9 | 0 | 252626 | 253426 |
| DASGFL010000004.1 | 98.876 | 801 | 9 | 0 | 20563 | 19763 |
| DASGFK010000001.1 | 98.876 | 801 | 9 | 0 | 252535 | 253335 |
| DASGFH010000004.1 | 98.876 | 801 | 9 | 0 | 20563 | 19763 |
| DASGES010000003.1 | 98.876 | 801 | 9 | 0 | 31376 | 30576 |
| DASGEO010000071.1 | 98.876 | 801 | 9 | 0 | 2737 | 1937 |
| DASGEO010000001.1 | 98.876 | 801 | 9 | 0 | 115697 | 114897 |
| DASGEJ010000001.1 | 98.876 | 801 | 9 | 0 | 115697 | 114897 |
| DASGEE010000009.1 | 98.876 | 801 | 9 | 0 | 1382 | 2182 |
| DASGDS010000024.1 | 98.876 | 801 | 9 | 0 | 30167 | 29367 |
| DASGDI010000022.1 | 98.876 | 801 | 9 | 0 | 20209 | 19409 |
| DASGDH010000009.1 | 98.876 | 801 | 9 | 0 | 49918 | 50718 |
| DASGDD010000002.1 | 98.876 | 801 | 9 | 0 | 142092 | 142892 |
| DASGDB010000023.1 | 98.876 | 801 | 9 | 0 | 20563 | 19763 |
| DASGDA010000016.1 | 98.876 | 801 | 9 | 0 | 20507 | 19707 |
| DASGCX010000002.1 | 98.876 | 801 | 9 | 0 | 143653 | 144453 |
| DASGCY010000002.1 | 98.876 | 801 | 9 | 0 | 143653 | 144453 |
| DASGCP010000026.1 | 98.876 | 801 | 9 | 0 | 20174 | 19374 |
| DASGCO010000019.1 | 98.876 | 801 | 9 | 0 | 20173 | 19373 |
| DASGCN010000026.1 | 98.876 | 801 | 9 | 0 | 20173 | 19373 |
| DASGCI010000006.1 | 98.876 | 801 | 9 | 0 | 20507 | 19707 |
| DASGCF010000018.1 | 98.876 | 801 | 9 | 0 | 20563 | 19763 |
| DASGCG010000021.1 | 98.876 | 801 | 9 | 0 | 20583 | 19783 |
| DASGCE010000012.1 | 98.876 | 801 | 9 | 0 | 20565 | 19765 |
| DASGCD010000002.1 | 98.876 | 801 | 9 | 0 | 72690 | 71890 |
| DASGCC010000003.1 | 98.876 | 801 | 9 | 0 | 20173 | 19373 |
| DASGCA010000018.1 | 98.876 | 801 | 9 | 0 | 20565 | 19765 |
| DASGBY010000015.1 | 98.876 | 801 | 9 | 0 | 20173 | 19373 |
| DASGBX010000016.1 | 98.876 | 801 | 9 | 0 | 20565 | 19765 |
| DASGBS010000061.1 | 98.876 | 801 | 9 | 0 | 10770 | 9970 |
| DASGBT010000011.1 | 98.876 | 801 | 9 | 0 | 20507 | 19707 |
| DASGAV010000011.1 | 98.876 | 801 | 9 | 0 | 20509 | 19709 |
| DASGAU010000019.1 | 98.876 | 801 | 9 | 0 | 20173 | 19373 |
| DASFKJ010000022.1 | 98.876 | 801 | 9 | 0 | 20795 | 19995 |
| DASGAS010000020.1 | 98.876 | 801 | 9 | 0 | 20173 | 19373 |
| DASGAQ010000048.1 | 98.876 | 801 | 9 | 0 | 10767 | 9967 |
| DASGAO010000077.1 | 98.876 | 801 | 9 | 0 | 10732 | 9932 |
| DASGAP010000006.1 | 98.876 | 801 | 9 | 0 | 20663 | 19863 |
| DASFZL010000004.1 | 98.876 | 801 | 9 | 0 | 50342 | 49542 |
| DASFZJ010000010.1 | 98.876 | 801 | 9 | 0 | 50031 | 49231 |
| DASFZI010000010.1 | 98.876 | 801 | 9 | 0 | 50031 | 49231 |
| DASFZH010000005.1 | 98.876 | 801 | 9 | 0 | 50032 | 49232 |
| DASFZE010000018.1 | 98.876 | 801 | 9 | 0 | 31684 | 30884 |
| DASFYU010000164.1 | 98.876 | 801 | 9 | 0 | 926 | 126 |
| DASFYE010000020.1 | 98.876 | 801 | 9 | 0 | 31694 | 30894 |
| DASFXX010000002.1 | 98.876 | 801 | 9 | 0 | 20583 | 19783 |
| DASFXI010000009.1 | 98.876 | 801 | 9 | 0 | 36689 | 35889 |
| DASFWQ010000005.1 | 98.876 | 801 | 9 | 0 | 20507 | 19707 |
| DASFWM010000010.1 | 98.876 | 801 | 9 | 0 | 20507 | 19707 |
| DASFWN010000074.1 | 98.876 | 801 | 9 | 0 | 10696 | 9896 |
| DASFWD010000001.1 | 98.876 | 801 | 9 | 0 | 188562 | 189362 |
| DASFWC010000024.1 | 98.876 | 801 | 9 | 0 | 20795 | 19995 |
| DASFWA010000006.1 | 98.876 | 801 | 9 | 0 | 20527 | 19727 |
| DASFVY010000003.1 | 98.876 | 801 | 9 | 0 | 39990 | 39190 |
| DASFVX010000014.1 | 98.876 | 801 | 9 | 0 | 20516 | 19716 |
| DASFVW010000026.1 | 98.876 | 801 | 9 | 0 | 20173 | 19373 |
| DASFVU010000012.1 | 98.876 | 801 | 9 | 0 | 20527 | 19727 |
| DASFVT010000012.1 | 98.876 | 801 | 9 | 0 | 20510 | 19710 |
| DASFVR010000002.1 | 98.876 | 801 | 9 | 0 | 142091 | 142891 |
| DASFVS010000002.1 | 98.876 | 801 | 9 | 0 | 142091 | 142891 |
| DASFVQ010000002.1 | 98.876 | 801 | 9 | 0 | 142091 | 142891 |
| DASFVM010000002.1 | 98.876 | 801 | 9 | 0 | 142091 | 142891 |
| DASFVK010000003.1 | 98.876 | 801 | 9 | 0 | 39364 | 40164 |
| DASFVJ010000013.1 | 98.876 | 801 | 9 | 0 | 20563 | 19763 |
| DASFVL010000016.1 | 98.876 | 801 | 9 | 0 | 20507 | 19707 |
| DASFVI010000011.1 | 98.876 | 801 | 9 | 0 | 20563 | 19763 |
| DASFVG010000054.1 | 98.876 | 801 | 9 | 0 | 10376 | 9576 |
| DASFVF010000011.1 | 98.876 | 801 | 9 | 0 | 20563 | 19763 |
| DASFVE010000002.1 | 98.876 | 801 | 9 | 0 | 143653 | 144453 |
| DASFVB010000002.1 | 98.876 | 801 | 9 | 0 | 142089 | 142889 |
| DASFUW010000013.1 | 98.876 | 801 | 9 | 0 | 20507 | 19707 |
| DASFUU010000024.1 | 98.876 | 801 | 9 | 0 | 20563 | 19763 |
| DASFUR010000002.1 | 98.876 | 801 | 9 | 0 | 143653 | 144453 |
| DASFUQ010000002.1 | 98.876 | 801 | 9 | 0 | 142091 | 142891 |
| DASFUP010000001.1 | 98.876 | 801 | 9 | 0 | 188562 | 189362 |
| DASFUM010000009.1 | 98.876 | 801 | 9 | 0 | 20175 | 19375 |
| DASFUL010000002.1 | 98.876 | 801 | 9 | 0 | 142102 | 142902 |
| DASFUJ010000008.1 | 98.876 | 801 | 9 | 0 | 20563 | 19763 |
| DASFUE010000001.1 | 98.876 | 801 | 9 | 0 | 20562 | 19762 |
| DASFUC010000075.1 | 98.876 | 801 | 9 | 0 | 2695 | 1895 |
| DASFTV010000038.1 | 98.876 | 801 | 9 | 0 | 882 | 82 |
| DASFTN010000046.1 | 98.876 | 801 | 9 | 0 | 886 | 86 |
| DASFTP010000017.1 | 98.876 | 801 | 9 | 0 | 20563 | 19763 |
| DASFTL010000049.1 | 98.876 | 801 | 9 | 0 | 887 | 87 |
| DASFTG010000019.1 | 98.876 | 801 | 9 | 0 | 20795 | 19995 |
| DASFTH010000006.1 | 98.876 | 801 | 9 | 0 | 15366 | 14566 |
| DASFTK010000001.1 | 98.876 | 801 | 9 | 0 | 20563 | 19763 |
| DASFTJ010000028.1 | 98.876 | 801 | 9 | 0 | 20619 | 19819 |
| DASFTF010000024.1 | 98.876 | 801 | 9 | 0 | 15681 | 14881 |
| DASFTE010000009.1 | 98.876 | 801 | 9 | 0 | 20563 | 19763 |
| DASFSZ010000036.1 | 98.876 | 801 | 9 | 0 | 887 | 87 |
| DASFSY010000001.1 | 98.876 | 801 | 9 | 0 | 230555 | 231355 |
| DASFTA010000128.1 | 98.876 | 801 | 9 | 0 | 6001 | 5201 |
| DASFSV010000011.1 | 98.876 | 801 | 9 | 0 | 20507 | 19707 |
| DASFSX010000013.1 | 98.876 | 801 | 9 | 0 | 20587 | 19787 |
| DASFSW010000063.1 | 98.876 | 801 | 9 | 0 | 10099 | 9299 |
| DASFSU010000013.1 | 98.876 | 801 | 9 | 0 | 20507 | 19707 |
| DASFST010000013.1 | 98.876 | 801 | 9 | 0 | 20507 | 19707 |
| DASFSS010000016.1 | 98.876 | 801 | 9 | 0 | 20863 | 20063 |
| DASFSR010000050.1 | 98.876 | 801 | 9 | 0 | 19366 | 18566 |
| DASFSR010000007.1 | 98.876 | 801 | 9 | 0 | 127346 | 126546 |
| DASFSQ010000015.1 | 98.876 | 801 | 9 | 0 | 20507 | 19707 |
| DASFSP010000009.1 | 98.876 | 801 | 9 | 0 | 20563 | 19763 |
| DASFSN010000147.1 | 98.876 | 801 | 9 | 0 | 2695 | 1895 |
| DASFSL010000013.1 | 98.876 | 801 | 9 | 0 | 20507 | 19707 |
| DASFSM010000013.1 | 98.876 | 801 | 9 | 0 | 20507 | 19707 |
| DASFSJ010000015.1 | 98.876 | 801 | 9 | 0 | 20507 | 19707 |
| DASFSI010000045.1 | 98.876 | 801 | 9 | 0 | 885 | 85 |
| DASFSK010000012.1 | 98.876 | 801 | 9 | 0 | 20507 | 19707 |
| DASFSH010000014.1 | 98.876 | 801 | 9 | 0 | 20507 | 19707 |
| DASFSG010000043.1 | 98.876 | 801 | 9 | 0 | 13435 | 12635 |
| DASFSE010000017.1 | 98.876 | 801 | 9 | 0 | 20507 | 19707 |
| DASFSF010000026.1 | 98.876 | 801 | 9 | 0 | 20563 | 19763 |
| DASFSD010000035.1 | 98.876 | 801 | 9 | 0 | 887 | 87 |
| DASFSC010000018.1 | 98.876 | 801 | 9 | 0 | 20918 | 20118 |
| DASFSA010000001.1 | 98.876 | 801 | 9 | 0 | 2706 | 1906 |
| DASFRX010000005.1 | 98.876 | 801 | 9 | 0 | 20563 | 19763 |
| DASFRV010000015.1 | 98.876 | 801 | 9 | 0 | 20863 | 20063 |
| DASFRS010000006.1 | 98.876 | 801 | 9 | 0 | 20563 | 19763 |
| DASFRW010000007.1 | 98.876 | 801 | 9 | 0 | 20918 | 20118 |
| DASFRT010000020.1 | 98.876 | 801 | 9 | 0 | 13701 | 12901 |
| DASFRO010000013.1 | 98.876 | 801 | 9 | 0 | 20507 | 19707 |
| DASFRQ010000014.1 | 98.876 | 801 | 9 | 0 | 20526 | 19726 |
| DASFRP010000018.1 | 98.876 | 801 | 9 | 0 | 20507 | 19707 |
| DASFRN010000024.1 | 98.876 | 801 | 9 | 0 | 13494 | 12694 |
| DASFRR010000099.1 | 98.876 | 801 | 9 | 0 | 2114 | 1314 |
| DASFRM010000022.1 | 98.876 | 801 | 9 | 0 | 20563 | 19763 |
| DASFRJ010000003.1 | 98.876 | 801 | 9 | 0 | 20563 | 19763 |
| DASFRH010000011.1 | 98.876 | 801 | 9 | 0 | 20507 | 19707 |
| DASFRG010000008.1 | 98.876 | 801 | 9 | 0 | 20563 | 19763 |
| DASFRE010000001.1 | 98.876 | 801 | 9 | 0 | 20563 | 19763 |
| DASFRD010000038.1 | 98.876 | 801 | 9 | 0 | 886 | 86 |
| DASFQY010000001.1 | 98.876 | 801 | 9 | 0 | 20563 | 19763 |
| DASFRA010000006.1 | 98.876 | 801 | 9 | 0 | 20564 | 19764 |
| DASFQP010000020.1 | 98.876 | 801 | 9 | 0 | 20563 | 19763 |
| DASFQV010000185.1 | 98.876 | 801 | 9 | 0 | 1707 | 907 |
| DASFQO010000015.1 | 98.876 | 801 | 9 | 0 | 20563 | 19763 |
| DASFQM010000014.1 | 98.876 | 801 | 9 | 0 | 20563 | 19763 |
| DASFQL010000039.1 | 98.876 | 801 | 9 | 0 | 6891 | 6091 |
| DASFQK010000001.1 | 98.876 | 801 | 9 | 0 | 228842 | 229642 |
| DASFQG010000011.1 | 98.876 | 801 | 9 | 0 | 20507 | 19707 |
| DASFQI010000001.1 | 98.876 | 801 | 9 | 0 | 20563 | 19763 |
| DASFQH010000005.1 | 98.876 | 801 | 9 | 0 | 20563 | 19763 |
| DASFQF010000007.1 | 98.876 | 801 | 9 | 0 | 20563 | 19763 |
| DASFPZ010000010.1 | 98.876 | 801 | 9 | 0 | 20563 | 19763 |
| DASFPY010000002.1 | 98.876 | 801 | 9 | 0 | 50971 | 51771 |
| DASFPU010000016.1 | 98.876 | 801 | 9 | 0 | 20507 | 19707 |
| DASFPR010000003.1 | 98.876 | 801 | 9 | 0 | 20563 | 19763 |
| DASFPS010000011.1 | 98.876 | 801 | 9 | 0 | 20563 | 19763 |
| DASFPP010000070.1 | 98.876 | 801 | 9 | 0 | 1752 | 952 |
| DASFPO010000047.1 | 98.876 | 801 | 9 | 0 | 6648 | 5848 |
| DASFPM010000057.1 | 98.876 | 801 | 9 | 0 | 2694 | 1894 |
| DASFPL010000009.1 | 98.876 | 801 | 9 | 0 | 20563 | 19763 |
| DASFPK010000021.1 | 98.876 | 801 | 9 | 0 | 20586 | 19786 |
| DASFPG010000002.1 | 98.876 | 801 | 9 | 0 | 129350 | 128550 |
| DASFPE010000002.1 | 98.876 | 801 | 9 | 0 | 129350 | 128550 |
| DASFPD010000007.1 | 98.876 | 801 | 9 | 0 | 20739 | 19939 |
| DASFPC010000018.1 | 98.876 | 801 | 9 | 0 | 20509 | 19709 |
| DASFOY010000025.1 | 98.876 | 801 | 9 | 0 | 20586 | 19786 |
| DASFOW010000002.1 | 98.876 | 801 | 9 | 0 | 129350 | 128550 |
| DASFOP010000002.1 | 98.876 | 801 | 9 | 0 | 129348 | 128548 |
| DASFON010000002.1 | 98.876 | 801 | 9 | 0 | 112837 | 112037 |
| DASFOG010000011.1 | 98.876 | 801 | 9 | 0 | 20563 | 19763 |
| DASFOH010000026.1 | 98.876 | 801 | 9 | 0 | 20795 | 19995 |
| DASFOD010000002.1 | 98.876 | 801 | 9 | 0 | 110874 | 111674 |
| DASFOE010000004.1 | 98.876 | 801 | 9 | 0 | 20795 | 19995 |
| DASFOF010000014.1 | 98.876 | 801 | 9 | 0 | 20736 | 19936 |
| DASFOC010000012.1 | 98.876 | 801 | 9 | 0 | 20795 | 19995 |
| DASFOB010000001.1 | 98.876 | 801 | 9 | 0 | 165034 | 165834 |
| DASFNN010000003.1 | 98.876 | 801 | 9 | 0 | 20795 | 19995 |
| DASFNI010000010.1 | 98.876 | 801 | 9 | 0 | 15953 | 16753 |
| DASFNH010000002.1 | 98.876 | 801 | 9 | 0 | 20583 | 19783 |
| DASFNC010000024.1 | 98.876 | 801 | 9 | 0 | 20795 | 19995 |
| DASFMX010000006.1 | 98.876 | 801 | 9 | 0 | 20723 | 19923 |
| DASFMY010000027.1 | 98.876 | 801 | 9 | 0 | 20723 | 19923 |
| DASFMV010000003.1 | 98.876 | 801 | 9 | 0 | 125983 | 125183 |
| DASFMU010000001.1 | 98.876 | 801 | 9 | 0 | 191270 | 192070 |
| DASFMS010000005.1 | 98.876 | 801 | 9 | 0 | 20723 | 19923 |
| DASFMP010000021.1 | 98.876 | 801 | 9 | 0 | 20795 | 19995 |
| DASFMJ010000002.1 | 98.876 | 801 | 9 | 0 | 129350 | 128550 |
| DASFMG010000013.1 | 98.876 | 801 | 9 | 0 | 50226 | 51026 |
| DASFMF010000003.1 | 98.876 | 801 | 9 | 0 | 20795 | 19995 |
| DASFME010000019.1 | 98.876 | 801 | 9 | 0 | 30388 | 29588 |
| DASFLZ010000009.1 | 98.876 | 801 | 9 | 0 | 20723 | 19923 |
| DASFLX010000019.1 | 98.876 | 801 | 9 | 0 | 20863 | 20063 |
| DASFLW010000002.1 | 98.876 | 801 | 9 | 0 | 20723 | 19923 |
| DASFLU010000002.1 | 98.876 | 801 | 9 | 0 | 20723 | 19923 |
| DASFLS010000020.1 | 98.876 | 801 | 9 | 0 | 20795 | 19995 |
| DASFLP010000021.1 | 98.876 | 801 | 9 | 0 | 20736 | 19936 |
| DASFLM010000021.1 | 98.876 | 801 | 9 | 0 | 20563 | 19763 |
| DASFLG010000004.1 | 98.876 | 801 | 9 | 0 | 20795 | 19995 |
| DASFLE010000020.1 | 98.876 | 801 | 9 | 0 | 20563 | 19763 |
| DASFLA010000006.1 | 98.876 | 801 | 9 | 0 | 20723 | 19923 |
| DASFKU010000002.1 | 98.876 | 801 | 9 | 0 | 15953 | 16753 |
| DASFKV010000015.1 | 98.876 | 801 | 9 | 0 | 30387 | 29587 |
| DASFKO010000004.1 | 98.876 | 801 | 9 | 0 | 20795 | 19995 |
| DASFKM010000017.1 | 98.876 | 801 | 9 | 0 | 1007 | 207 |
| DASFKL010000002.1 | 98.876 | 801 | 9 | 0 | 20723 | 19923 |
| DASFKI010000019.1 | 98.876 | 801 | 9 | 0 | 20795 | 19995 |
| DASFKG010000020.1 | 98.876 | 801 | 9 | 0 | 20794 | 19994 |
| DASFKC010000020.1 | 98.876 | 801 | 9 | 0 | 20795 | 19995 |
| DASFKB010000003.1 | 98.876 | 801 | 9 | 0 | 20795 | 19995 |
| DASFKD010000004.1 | 98.876 | 801 | 9 | 0 | 20583 | 19783 |
| DASFJY010000022.1 | 98.876 | 801 | 9 | 0 | 20794 | 19994 |
| DASFKA010000006.1 | 98.876 | 801 | 9 | 0 | 20723 | 19923 |
| DASFJZ010000002.1 | 98.876 | 801 | 9 | 0 | 20723 | 19923 |
| DASFJX010000016.1 | 98.876 | 801 | 9 | 0 | 30387 | 29587 |
| DASFJV010000017.1 | 98.876 | 801 | 9 | 0 | 30387 | 29587 |
| DASFJU010000002.1 | 98.876 | 801 | 9 | 0 | 138811 | 139611 |
| DASFJT010000021.1 | 98.876 | 801 | 9 | 0 | 20736 | 19936 |
| DASFJS010000002.1 | 98.876 | 801 | 9 | 0 | 188409 | 187609 |
| DASFJO010000025.1 | 98.876 | 801 | 9 | 0 | 20795 | 19995 |
| DASFJN010000017.1 | 98.876 | 801 | 9 | 0 | 21074 | 20274 |
| DASFJL010000008.1 | 98.876 | 801 | 9 | 0 | 20535 | 19735 |
| DASFJJ010000022.1 | 98.876 | 801 | 9 | 0 | 20795 | 19995 |
| DASFJF010000003.1 | 98.876 | 801 | 9 | 0 | 129356 | 128556 |
| DASFJB010000004.1 | 98.876 | 801 | 9 | 0 | 20631 | 19831 |
| DASFIX010000018.1 | 98.876 | 801 | 9 | 0 | 20563 | 19763 |
| DASFIY010000020.1 | 98.876 | 801 | 9 | 0 | 20795 | 19995 |
| DASFIZ010000016.1 | 98.876 | 801 | 9 | 0 | 30386 | 29586 |
| DASFIV010000002.1 | 98.876 | 801 | 9 | 0 | 20736 | 19936 |
| DASFIW010000043.1 | 98.876 | 801 | 9 | 0 | 947 | 147 |
| DASFIU010000002.1 | 98.876 | 801 | 9 | 0 | 20795 | 19995 |
| DASFIQ010000018.1 | 98.876 | 801 | 9 | 0 | 30387 | 29587 |
| DASFIR010000005.1 | 98.876 | 801 | 9 | 0 | 20723 | 19923 |
| DASFIO010000002.1 | 98.876 | 801 | 9 | 0 | 129349 | 128549 |
| DASFIL010000002.1 | 98.876 | 801 | 9 | 0 | 20563 | 19763 |
| DASFIK010000021.1 | 98.876 | 801 | 9 | 0 | 20795 | 19995 |
| DASFIF010000025.1 | 98.876 | 801 | 9 | 0 | 20573 | 19773 |
| DASFHV010000003.1 | 98.876 | 801 | 9 | 0 | 20563 | 19763 |
| DASFHU010000059.1 | 98.876 | 801 | 9 | 0 | 2672 | 1872 |
| DASFHK010000020.1 | 98.876 | 801 | 9 | 0 | 20564 | 19764 |
| DASFHJ010000030.1 | 98.876 | 801 | 9 | 0 | 20209 | 19409 |
| DASFHM010000020.1 | 98.876 | 801 | 9 | 0 | 2745 | 1945 |
| DASFHH010000024.1 | 98.876 | 801 | 9 | 0 | 20563 | 19763 |
| DASFHI010000018.1 | 98.876 | 801 | 9 | 0 | 20507 | 19707 |
| DASFHF010000012.1 | 98.876 | 801 | 9 | 0 | 20583 | 19783 |
| DASFHG010000014.1 | 98.876 | 801 | 9 | 0 | 20507 | 19707 |
| DASFHE010000022.1 | 98.876 | 801 | 9 | 0 | 20563 | 19763 |
| DASFHC010000011.1 | 98.876 | 801 | 9 | 0 | 20563 | 19763 |
| DASFHD010000022.1 | 98.876 | 801 | 9 | 0 | 20507 | 19707 |
| DASFHA010000014.1 | 98.876 | 801 | 9 | 0 | 20507 | 19707 |
| DASFGX010000024.1 | 98.876 | 801 | 9 | 0 | 20583 | 19783 |
| DASFGW010000001.1 | 98.876 | 801 | 9 | 0 | 20185 | 19385 |
| DASFGV010000016.1 | 98.876 | 801 | 9 | 0 | 20563 | 19763 |
| DASFGR010000002.1 | 98.876 | 801 | 9 | 0 | 129350 | 128550 |
| DASFGO010000007.1 | 98.876 | 801 | 9 | 0 | 15953 | 16753 |
| DASFGK010000001.1 | 98.876 | 801 | 9 | 0 | 40247 | 41047 |
| DASFGD010000010.1 | 98.876 | 801 | 9 | 0 | 49702 | 50502 |
| DASFFY010000009.1 | 98.876 | 801 | 9 | 0 | 20510 | 19710 |
| DASFFP010000023.1 | 98.876 | 801 | 9 | 0 | 2690 | 1890 |
| DASFFO010000003.1 | 98.876 | 801 | 9 | 0 | 113529 | 112729 |
| DASFFN010000002.1 | 98.876 | 801 | 9 | 0 | 113529 | 112729 |
| DASFFL010000002.1 | 98.876 | 801 | 9 | 0 | 113519 | 112719 |
| DASFFE010000011.1 | 98.876 | 801 | 9 | 0 | 20563 | 19763 |
| DASFFB010000018.1 | 98.876 | 801 | 9 | 0 | 20563 | 19763 |
| DASFEO010000014.1 | 98.876 | 801 | 9 | 0 | 20563 | 19763 |
| DASFEJ010000016.1 | 98.876 | 801 | 9 | 0 | 30387 | 29587 |
| DASFEG010000001.1 | 98.876 | 801 | 9 | 0 | 188229 | 187429 |
| DASFEH010000002.1 | 98.876 | 801 | 9 | 0 | 20563 | 19763 |
| DASFEF010000017.1 | 98.876 | 801 | 9 | 0 | 30387 | 29587 |
| DASFDT010000024.1 | 98.876 | 801 | 9 | 0 | 20877 | 20077 |
| DASFDU010000015.1 | 98.876 | 801 | 9 | 0 | 30036 | 29236 |
| DASFDR010000002.1 | 98.876 | 801 | 9 | 0 | 113528 | 112728 |
| DASFDO010000019.1 | 98.876 | 801 | 9 | 0 | 30036 | 29236 |
| DASFDQ010000018.1 | 98.876 | 801 | 9 | 0 | 20877 | 20077 |
| DASFDK010000021.1 | 98.876 | 801 | 9 | 0 | 30035 | 29235 |
| DASFDM010000005.1 | 98.876 | 801 | 9 | 0 | 63904 | 63104 |
| DASFDN010000003.1 | 98.876 | 801 | 9 | 0 | 113529 | 112729 |
| DASFDI010000026.1 | 98.876 | 801 | 9 | 0 | 20867 | 20067 |
| DASFDJ010000026.1 | 98.876 | 801 | 9 | 0 | 20867 | 20067 |
| DASFDG010000013.1 | 98.876 | 801 | 9 | 0 | 30037 | 29237 |
| DASFDH010000013.1 | 98.876 | 801 | 9 | 0 | 30037 | 29237 |
| DASFDF010000012.1 | 98.876 | 801 | 9 | 0 | 30037 | 29237 |
| DASFDE010000012.1 | 98.876 | 801 | 9 | 0 | 20877 | 20077 |
| DASFCW010000019.1 | 98.876 | 801 | 9 | 0 | 30036 | 29236 |
| DASFCU010000019.1 | 98.876 | 801 | 9 | 0 | 30036 | 29236 |
| DASFCT010000018.1 | 98.876 | 801 | 9 | 0 | 30036 | 29236 |
| DASFCS010000022.1 | 98.876 | 801 | 9 | 0 | 20877 | 20077 |
| DASFBM010000021.1 | 98.876 | 801 | 9 | 0 | 30038 | 29238 |
| DASFBL010000002.1 | 98.876 | 801 | 9 | 0 | 113529 | 112729 |
| DASFBK010000006.1 | 98.876 | 801 | 9 | 0 | 30036 | 29236 |
| DASFBI010000020.1 | 98.876 | 801 | 9 | 0 | 30038 | 29238 |
| DASFBJ010000002.1 | 98.876 | 801 | 9 | 0 | 113529 | 112729 |
| DASFBH010000033.1 | 98.876 | 801 | 9 | 0 | 20840 | 20040 |
| DASFBF010000002.1 | 98.876 | 801 | 9 | 0 | 113529 | 112729 |
| DASFBC010000017.1 | 98.876 | 801 | 9 | 0 | 30036 | 29236 |
| DASFBD010000028.1 | 98.876 | 801 | 9 | 0 | 20877 | 20077 |
| DASFBA010000001.1 | 98.876 | 801 | 9 | 0 | 113529 | 112729 |
| DASFBE010000011.1 | 98.876 | 801 | 9 | 0 | 63950 | 63150 |
| DASFBB010000003.1 | 98.876 | 801 | 9 | 0 | 113426 | 112626 |
| DASFAZ010000013.1 | 98.876 | 801 | 9 | 0 | 44290 | 43490 |
| DASFAX010000014.1 | 98.876 | 801 | 9 | 0 | 44290 | 43490 |
| DASFAW010000011.1 | 98.876 | 801 | 9 | 0 | 44290 | 43490 |
| DASFAY010000001.1 | 98.876 | 801 | 9 | 0 | 151574 | 150774 |
| DASFAU010000042.1 | 98.876 | 801 | 9 | 0 | 15036 | 14236 |
| DASFAT010000011.1 | 98.876 | 801 | 9 | 0 | 44290 | 43490 |
| DASFAV010000013.1 | 98.876 | 801 | 9 | 0 | 44291 | 43491 |
| DASFAR010000025.1 | 98.876 | 801 | 9 | 0 | 30036 | 29236 |
| DASFAS010000013.1 | 98.876 | 801 | 9 | 0 | 44290 | 43490 |
| DASFAQ010000011.1 | 98.876 | 801 | 9 | 0 | 44290 | 43490 |
| DASFAP010000014.1 | 98.876 | 801 | 9 | 0 | 15649 | 14849 |
| DASFAO010000017.1 | 98.876 | 801 | 9 | 0 | 20877 | 20077 |
