# Supplementary material for: The conserved transcription factor PrlP modulates colonization and pathogenicity of Streptococcus suis in response to environmental stress
Source: PLoS Pathog. 2025 Jul 18;21(7):e1013314. doi: 10.1371/journal.ppat.1013314 (PMC12273997; doi:10.1371/journal.ppat.1013314)
Supplement: S2 Table — (DOCX) [file ppat.1013314.s003.docx]

**Table S2.** Expression levels of differentially expressed genes in Δ*prlP* and *prlP*-ΔN compared to WT

| **Code for ORF(SC84)** | **Code for ORF(SC19)** | **Functional annotation** | **log2FoldChange** | **padj** |
| --- | --- | --- | --- | --- |
| SSUSC84_RS05020 | B9H01_05145 | hypothetical protein SSUSC84_0949 | 5.551050154 | 4.2281E-173 |
| SSUSC84_RS05025 | B9H01_05150 | hypothetical protein SSU98_1066 | 5.27830411 | 4.25227E-90 |
| SSUSC84_RS03095 | B9H01_03190 | hypothetical protein SSU05_0636 | 4.866338708 | 6.4284E-162 |
| SSUSC84_RS03090 | B9H01_03185 | hypothetical protein NJAUSS_0698 | 4.647902184 | 3.131E-102 |
| SSUSC84_RS08530 | B9H01_08740 | ribonucleases G and E | 4.255241565 | 4.9975E-176 |
| SSUSC84_RS00900 | B9H01_00920 | endopeptidase | 4.176369347 | 1.8642E-175 |
| SSUSC84_RS03085 | B9H01_03180 | hypothetical protein SSUSC84_0566 | 4.129841089 | 4.6654E-166 |
| SSUSC84_RS04095 | B9H01_04200 | hypothetical protein NJAUSS_0894 | 3.892643118 | 8.4145E-125 |
| SSUSC84_RS09870 | B9H01_10110 | cell wall anchor domain-containing protein | 3.816698355 | 1.7883E-142 |
| SSUSC84_RS03080 | B9H01_03175 | hypothetical protein SSUSC84_0565 | 3.792796159 | 1.9894E-119 |
| SSUSC84_RS09865 | B9H01_10105 | hypothetical protein SSU05_2101 | 3.781679709 | 6.67583E-28 |
| SSUSC84_RS03075 | B9H01_03170 | 6-phosphogluconolactonase/glucosamine-6-phosphate isomerase/deaminase | 3.665474689 | 4.8986E-109 |
| SSUSC84_RS09880 | NA | hypothetical protein SSUSC84_1909 | 3.506936929 | 0.050828636 |
| SSUSC84_RS09875 | B9H01_10115 | hypothetical protein SSU05_2104 | 3.468448223 | 2.2469E-109 |
| SSUSC84_RS01405 | B9H01_01435 | methyl-accepting chemotaxis protein | 3.44154174 | 5.4313E-105 |
| SSUSC84_RS07840 | B9H01_08035 | hypothetical protein SSU05_1664 | 3.433179732 | 1.666E-118 |
| SSUSC84_RS07810 | B9H01_08010 | hypothetical protein SSU05_1659 | 3.387526402 | 2.25994E-98 |
| SSUSC84_RS09860 | B9H01_10100 | putative pilus subunit protein | 3.219699464 | 1.68618E-87 |
| SSUSC84_RS01410 | B9H01_01440 | methyl-accepting chemotaxis protein | 3.192828472 | 9.4789E-111 |
| SSUSC84_RS06470 | B9H01_06615 | surface antigen SP1 | 3.122694997 | 1.5443E-109 |
| SSUSC84_RS08335 | B9H01_08540 | metal ABC transporter periplasmic protein/surface antigen | 3.048967614 | 2.62865E-90 |
| SSUSC84_RS09855 | B9H01_10095 | hypothetical protein SSU05_2099 | 2.890527166 | 8.50567E-19 |
| SSUSC84_RS06440 | B9H01_06585 | amino acid ABC transporter permease | 2.855581507 | 3.3127E-85 |
| SSUSC84_RS06435 | B9H01_06580 | amino acid ABC transporter permease | 2.81635122 | 8.06702E-42 |
| SSUSC84_RS10590 | B9H01_07770 | Iron-containing alcohol dehydrogenase | 2.792289529 | 2.77808E-51 |
| SSUSC84_RS01400 | B9H01_01430 | surface-anchored protein | 2.782444055 | 5.1476E-90 |
| SSUSC84_RS03145 | B9H01_03245 | ferrichrome transport permease | 2.604402925 | 1.38581E-63 |
| SSUSC84_RS07405 | B9H01_07600 | hypothetical protein SSU05_1575 | 2.536060455 | 9.21509E-46 |
| SSUSC84_RS06430 | B9H01_06575 | glutamine ABC transporter substrate-binding protein | 2.515601787 | 4.61674E-63 |
| SSUSC84_RS03140 | B9H01_03240 | ferrichrome transport permease | 2.45065171 | 1.41537E-29 |
| SSUSC84_RS07830 | B9H01_08030 | methyl-accepting chemotaxis protein | 2.437323204 | 9.28594E-69 |
| SSUSC84_RS08325 | B9H01_08530 | metal ABC transporter ATPase | 2.147010711 | 1.60268E-47 |
| SSUSC84_RS09700 | B9H01_09930 | amino acid ABC transporter ATP-binding protein | 2.146170735 | 4.66531E-56 |
| SSUSC84_RS07825 | B9H01_08025 | hypothetical protein SSU98_1673 | 2.094465188 | 3.36183E-19 |
| SSUSC84_RS01075 | B9H01_01095 | hypothetical protein SSU05_0196 | 2.009161302 | 3.79392E-50 |
| SSUSC84_RS00185 | NA | hypothetical protein | 2.00349399 | 3.52234E-36 |
| SSUSC84_RS06425 | B9H01_06570 | polar amino acid ABC transporter ATPase | 1.96775386 | 8.76697E-22 |
| SSUSC84_RS07580 | B9H01_07775 | phage shock protein C, PspC | 1.95220356 | 7.86896E-32 |
| SSUSC84_RS06985 | B9H01_07170 | ABC transporter ATP-binding protein | 1.941741154 | 2.69353E-38 |
| SSUSC84_RS06990 | B9H01_07175 | ABC transporter permease | 1.938447458 | 1.3346E-22 |
| SSUSC84_RS06230 | B9H01_06375 | hypothetical protein SSU05_1320 | 1.904648919 | 5.93913E-26 |
| SSUSC84_RS08930 | B9H01_09140 | hypothetical protein SSU05_1904 | 1.884095973 | 4.06753E-26 |
| SSUSC84_RS06995 | B9H01_07180 | ABC transporter periplasmic protein | 1.880494718 | 5.07536E-23 |
| SSUSC84_RS07285 | B9H01_07475 | hypothetical protein SSU05_1548 | 1.840652165 | 8.09021E-12 |
| SSUSC84_RS05030 | B9H01_05155 | transcriptional regulator | 1.799982486 | 4.42399E-34 |
| SSUSC84_RS05935 | B9H01_06075 | ABC transporter permease | 1.78473564 | 3.17279E-21 |
| SSUSC84_RS07990 | B9H01_08185 | SpoU rRNA methylase family protein | 1.748102576 | 1.60398E-07 |
| SSUSC84_RS02510 | B9H01_02580 | hypothetical protein SSU05_0509 | 1.712278 | 6.04282E-29 |
| SSUSC84_RS09695 | B9H01_09925 | transcriptional regulator | 1.663708451 | 9.43822E-29 |
| SSUSC84_RS05930 | B9H01_06070 | hypothetical protein SSU98_1270 | 1.641055576 | 2.00242E-19 |
| SSUSC84_RS08420 | B9H01_08625 | hypothetical protein | 1.617537064 | 3.3644E-20 |
| SSUSC84_RS05680 | B9H01_05810 | isocitrate dehydrogenase | 1.615034118 | 1.28813E-13 |
| SSUSC84_RS03825 | B9H01_03930 | multidrug ABC transporter ATPase and permease | 1.611043949 | 3.95559E-09 |
| SSUSC84_RS00190 | NA | hypothetical protein | 1.598028998 | 7.11035E-07 |
| SSUSC84_RS03885 | B9H01_03990 | cell envelope proteinase | 1.571023035 | 1.24704E-23 |
| SSUSC84_RS05685 | B9H01_05815 | citrate synthase | 1.557021592 | 2.19666E-08 |
| SSUSC84_RS04670 | B9H01_04795 | cobalt ABC transporter permease CbiQ and related transporters | 1.553348704 | 0.028549849 |
| SSUSC84_RS05920 | B9H01_06060 | ABC transporter ATPase | 1.553016124 | 3.10934E-06 |
| SSUSC84_RS07080 | B9H01_07270 | transposase | 1.52959779 | 1.09638E-07 |
| SSUSC84_RS03880 | B9H01_03990 | subtilisin-like serine protease | 1.527894794 | 4.18142E-15 |
| SSUSC84_RS04755 | B9H01_04880 | hypothetical protein SSUSC84_0897 | 1.520100721 | 1.29639E-06 |
| SSUSC84_RS06880 | B9H01_07055 | serine/threonine ABC transporter SstT | 1.513662018 | 1.46484E-10 |
| SSUSC84_RS08425 | B9H01_08630 | hypothetical protein SSU05_1793 | 1.508229058 | 3.69156E-09 |
| SSUSC84_RS08460 | B9H01_08665 | 3-oxoacyl-ACP synthase | 1.461816754 | 2.33229E-27 |
| SSUSC84_RS07010 | B9H01_07195 | transcription antiterminator | 1.420345024 | 1.48091E-05 |
| SSUSC84_RS07000 | B9H01_07185 | beta-glucosidase/6-phospho-beta-glucosidase/beta- galactosidase | 1.418785668 | 3.06789E-15 |
| SSUSC84_RS05925 | B9H01_06065 | ABC transporter permease | 1.408214618 | 2.04436E-11 |
| SSUSC84_RS03745 | B9H01_03850 | C-P lyase regulatory protein | 1.387075359 | 0.002221183 |
| SSUSC84_RS04760 | B9H01_04885 | NAD(FAD)-dependent dehydrogenase | 1.364866114 | 2.1578E-11 |
| SSUSC84_RS10440 | NA | hypothetical protein SSUST1_1133 | 1.353542141 | 0.007346775 |
| SSUSC84_RS01665 | B9H01_01700 | laminin binding protein | 1.341874625 | 0.006362155 |
| SSUSC84_RS07005 | B9H01_07190 | phosphotransferase system IIC component, glucose/maltose/N-acetylglucosamine-specific | 1.295561665 | 7.8613E-14 |
| SSUSC84_RS04865 | B9H01_04990 | 1,4-alpha-glucan branching enzyme | 1.252774187 | 7.71994E-17 |
| SSUSC84_RS01670 | B9H01_01705 | hypothetical protein SSU98_0327 | 1.226863288 | 0.000364627 |
| SSUSC84_RS05690 | B9H01_05820 | aconitate hydratase | 1.16870742 | 1.20986E-07 |
| SSUSC84_RS09645 | B9H01_09875 | L-ascorbate 6-phosphate lactonase | 1.016474432 | 0.000242581 |
| SSUSC84_RS04080 | B9H01_04185 | NADP-dependent L-serine/L-allo-threonine dehydrogenase | -1.020209246 | 6.84331E-13 |
| SSUSC84_RS05805 | B9H01_05940 | hypothetical protein SSU98_1243 | -1.061285701 | 0.001078799 |
| SSUSC84_RS05815 | B9H01_05950 | hypothetical protein SSU98_1245 | -1.080990514 | 0.059973684 |
| SSUSC84_RS05755 | B9H01_05885 | phosphotransferase system, mannose/fructose/N-acetylgalactosamine-specific component IIC | -1.09766511 | 0.000392247 |
| SSUSC84_RS03030 | B9H01_03125 | arginine deiminase | -1.099942993 | 7.02638E-10 |
| SSUSC84_RS06025 | B9H01_06170 | cell wall biosynthesis glycosyltransferase | -1.111392091 | 3.16638E-14 |
| SSUSC84_RS02655 | B9H01_02725 | multidrug ABC transporter ATPase | -1.129165882 | 5.54976E-13 |
| SSUSC84_RS02800 | B9H01_02875 | hypothetical protein SSUSC84_0507 | -1.151410837 | 7.51565E-12 |
| SSUSC84_RS00840 | B9H01_00860 | hypothetical protein SSUSC84_0133 | -1.156695624 | 1.77287E-07 |
| SSUSC84_RS09185 | B9H01_09410 | dihydroxyacetone kinase | -1.164735993 | 4.91301E-17 |
| SSUSC84_RS00045 | NA |  | -1.169030768 | 0.022793246 |
| SSUSC84_RS07980 | B9H01_08175 | membrane-associated phospholipid phosphatase | -1.183897606 | 3.77128E-06 |
| SSUSC84_RS02230 | B9H01_02285 | phosphotransferase system, mannose/fructose/N-acetylgalactosamine-specific component IIB | -1.193685793 | 0.001392756 |
| SSUSC84_RS02520 | B9H01_02590 | glutathione reductase | -1.215086245 | 5.13307E-18 |
| SSUSC84_RS00220 | NA | hypothetical protein | -1.222711691 | 0.021793725 |
| SSUSC84_RS02075 | B9H01_02130 | serine/threonine protein kinase | -1.224203526 | 1.07935E-19 |
| SSUSC84_RS06955 | B9H01_07140 | hypothetical protein SSUSC84_1329 | -1.22540607 | 3.6538E-11 |
| SSUSC84_RS06030 | B9H01_06175 | hypothetical protein SSU05_1276 | -1.245800123 | 4.63411E-17 |
| SSUSC84_RS05765 | B9H01_05895 | unsaturated glucuronyl hydrolase | -1.263237957 | 0.000264254 |
| SSUSC84_RS03040 | B9H01_03135 | ornithine carbamoyltransferase | -1.264366653 | 1.30958E-19 |
| SSUSC84_RS05760 | B9H01_05890 | phosphotransferase system, mannose/fructose/N-acetylgalactosamine-specific component IIB | -1.280518343 | 0.001296814 |
| SSUSC84_RS07330 | B9H01_07520 | alpha-galactosidase | -1.294701302 | 1.26712E-10 |
| SSUSC84_RS04075 | B9H01_04180 | lytic murein transglycosylase | -1.312741012 | 1.01409E-12 |
| SSUSC84_RS05180 | B9H01_05300 | pantothenate kinase | -1.324203039 | 6.00145E-13 |
| SSUSC84_RS05105 | B9H01_05230 | hypothetical protein SSU98_1083 | -1.329407953 | 5.8109E-09 |
| SSUSC84_RS05495 | B9H01_05625 | glucuronate isomerase | -1.338800284 | 3.9941E-06 |
| SSUSC84_RS02065 | B9H01_02120 | tRNA and rRNA cytosine-C5-methylase | -1.34080883 | 4.92575E-23 |
| SSUSC84_RS03000 | B9H01_03095 | multi antimicrobial extrusion (MATE) family transporter | -1.359065882 | 7.02119E-24 |
| SSUSC84_RS05855 | B9H01_05990 | hypothetical protein SSU05_1237 | -1.380277513 | 5.04888E-23 |
| SSUSC84_RS03035 | B9H01_03130 | histone acetyltransferase HPA2-like acetyltransferase | -1.38360569 | 2.82832E-11 |
| SSUSC84_RS04480 | B9H01_04605 | DNA helicase | -1.389153966 | 4.22342E-22 |
| SSUSC84_RS07065 | B9H01_07250 | hypothetical protein SSU98_1514 | -1.40436219 | 2.04436E-11 |
| SSUSC84_RS00845 | B9H01_00865 | Thiol-disulfide isomerase and thioredoxin | -1.420172079 | 0.018136834 |
| SSUSC84_RS10230 | B9H01_10475 | recombination protein F | -1.431637025 | 3.09521E-17 |
| SSUSC84_RS03680 | B9H01_03785 | hypothetical protein SSU05_0768 | -1.456345152 | 0.002911725 |
| SSUSC84_RS05850 | B9H01_05985 | alanyl-tRNA synthetase | -1.473708105 | 5.58453E-28 |
| SSUSC84_RS07290 | B9H01_07480 | hypothetical protein SSU98_1559 | -1.496034067 | 8.61611E-11 |
| SSUSC84_RS04085 | B9H01_04190 | translation initiation factor 1 | -1.503330115 | 5.08532E-24 |
| SSUSC84_RS05075 | B9H01_05200 | adenylate cyclase family protein | -1.503380779 | 1.7109E-10 |
| SSUSC84_RS04335 | B9H01_04445 | NisR | -1.532247742 | 1.46981E-14 |
| SSUSC84_RS01575 | B9H01_01610 | ferric uptake regulator family protein | -1.555902141 | 8.98629E-26 |
| SSUSC84_RS05585 | B9H01_05715 | lipid kinase | -1.555931532 | 4.63264E-24 |
| SSUSC84_RS06095 | B9H01_06240 | phosphoglycerol transferase/alkaline phosphatase superfamily protein | -1.560508672 | 4.27835E-21 |
| SSUSC84_RS02825 | B9H01_10625 | hypothetical protein SSUSC84_0512 | -1.569899748 | 0.008417302 |
| SSUSC84_RS02060 | B9H01_02115 | methionyl-tRNA formyltransferase | -1.579352853 | 6.11078E-31 |
| SSUSC84_RS02070 | B9H01_02125 | serine/threonine protein phosphatase | -1.58886446 | 2.5143E-16 |
| SSUSC84_RS10470 | B9H01_04315 | Abortive infection bacteriophage resistance related protein | -1.623570545 | 2.25202E-17 |
| SSUSC84_RS09305 | B9H01_09530 | surface-anchored serine protease | -1.626953415 | 9.0133E-34 |
| SSUSC84_RS06960 | B9H01_07145 | Zn-dependent protease | -1.634153001 | 2.82407E-21 |
| SSUSC84_RS03575 | B9H01_03680 | phage holin protein | -1.655200071 | 9.74748E-06 |
| SSUSC84_RS01930 | B9H01_01980 | transcriptional regulator | -1.693364714 | 3.75782E-24 |
| SSUSC84_RS00270 | NA | hypothetical protein | -1.713116368 | 2.43128E-15 |
| SSUSC84_RS10425 | NA | hypothetical protein | -1.729731591 | 0.002159595 |
| SSUSC84_RS09795 | B9H01_10030 | metal-dependent transcriptional regulator | -1.730622583 | 6.84774E-37 |
| SSUSC84_RS09195 | B9H01_09420 | glycerol uptake facilitator and related permease (major Intrinsic protein family) | -1.779485211 | 0.000721026 |
| SSUSC84_RS00265 | NA |  | -1.801760864 | 8.97071E-31 |
| SSUSC84_RS09980 | B9H01_10225 | arginine repressor | -1.815268571 | 3.45777E-31 |
| SSUSC84_RS02995 | B9H01_03085 | hypothetical protein SSU98_0616 | -1.88461799 | 6.89563E-30 |
| SSUSC84_RS05100 | B9H01_05225 | redox-sensing transcriptional repressor Rex | -1.899848645 | 5.26875E-29 |
| SSUSC84_RS01970 | B9H01_02020 | hypothetical protein SSUSC84_0349 | -1.930618336 | 2.40736E-44 |
| SSUSC84_RS02280 | B9H01_02335 | hypothetical protein SSUSC84_0398 | -1.938796959 | 1.92833E-43 |
| SSUSC84_RS07190 | B9H01_07380 | Gp21 protein | -2.022419015 | 9.36382E-39 |
| SSUSC84_RS07610 | B9H01_07805 | hypothetical protein SSU05_1615 | -2.071049973 | 7.2525E-19 |
| SSUSC84_RS02885 | B9H01_02960 | transposase | -2.12045155 | 1.12429E-12 |
| SSUSC84_RS07185 | B9H01_07375 | transcriptional regulator | -2.125994411 | 3.70558E-39 |
| SSUSC84_RS04315 | B9H01_04425 | phage integrase family site specific recombinase | -2.159208938 | 1.71352E-19 |
| SSUSC84_RS02920 | B9H01_03005 | hypothetical protein SSUSC84_0532 | -2.176425109 | 6.83713E-17 |
| SSUSC84_RS09190 | B9H01_09415 | phosphotransferase mannnose-specific family component IIA | -2.218523737 | 1.32422E-12 |
| SSUSC84_RS04935 | B9H01_05060 | hypothetical protein SSUSC84_0933 | -2.274510967 | 6.1776E-11 |
| SSUSC84_RS08240 | B9H01_08440 | hypothetical protein SSU05_1751 | -2.289018413 | 0.000179429 |
| SSUSC84_RS10225 | B9H01_10470 | S4 domain-containing protein YaaA | -2.33573285 | 1.67426E-27 |
| SSUSC84_RS04090 | B9H01_04195 | hypothetical protein SSU05_0857 | -2.360345419 | 2.172E-13 |
| SSUSC84_RS01915 | B9H01_01965 | ATPases with chaperone activity, ATP-binding protein | -2.435860623 | 1.91335E-22 |
| SSUSC84_RS02990 | B9H01_03080 | hypothetical protein SSU98_0615 | -2.580226139 | 3.00908E-08 |
| SSUSC84_RS04165 | B9H01_04270 | hypothetical protein SSUSC84_0777 | -2.603515037 | 7.60778E-17 |
| SSUSC84_RS05000 | B9H01_05120 | sugar metabolism transcriptional regulator | -2.620291368 | 3.68159E-60 |
| SSUSC84_RS10395 | B9H01_10560 | hypothetical protein | -2.638189878 | 0.000241431 |
| SSUSC84_RS04170 | B9H01_04275 | cytoplasmic protein | -2.850404442 | 2.82757E-23 |
| SSUSC84_RS04140 | B9H01_04245 | hypothetical protein SSU05_0866 | -3.031908751 | 1.64997E-68 |
| SSUSC84_RS09715 | B9H01_09945 | surface antigen | -3.160435572 | 2.99567E-57 |
| SSUSC84_RS05200 | B9H01_05330 | Signal transduction histidine kinase | -3.212714128 | 1.21E-103 |
| SSUSC84_RS00700 | B9H01_00715 | hypothetical protein SSUSC84_0104 | -3.227069791 | 1.85134E-28 |
| SSUSC84_RS10290 | B9H01_10535 | chromosome partitioning protein | -3.3280082 | 1.12998E-97 |
| SSUSC84_RS05195 | B9H01_05320 | transglutaminase | -3.391401091 | 4.3385E-122 |
| SSUSC84_RS05205 | B9H01_05335 | response regulator | -3.585759504 | 1.50451E-91 |
| SSUSC84_RS03710 | B9H01_03815 | hypothetical protein SSUSC84_0687 | -3.671979204 | 8.53625E-28 |
| SSUSC84_RS08865 | B9H01_09080 | exopolysaccharide biosynthesis protein related to N-acetylglucosamine-1-phosphodiester alpha-N-acetylglucosaminidase | -3.74443689 | 3.17952E-06 |
| SSUSC84_RS03570 | B9H01_03675 | hypothetical protein SSU05_0744 | -4.07254378 | 1.6244E-165 |
| SSUSC84_RS09630 | B9H01_09855 | hypothetical protein SSUSC84_1859 | -4.230521631 | 1.09618E-52 |
| SSUSC84_RS08860 | B9H01_09075 | cell wall biosynthesis glycosyltransferase | -4.615017123 | 6.05339E-10 |
| SSUSC84_RS10285 | B9H01_10530 | trypsin-like serine protease | -4.84164841 | 1.2271E-221 |
| SSUSC84_RS05210 | B9H01_05340 | hypothetical protein SSU05_1097 | -6.282351736 | 0 |
| SSUSC84_RS08650 | B9H01_08860 | lipoprotein | -6.512313739 | 0 |
| SSUSC84_RS02120 | B9H01_02175 | amidophosphoribosyltransferase | -6.986139207 | 1.47651E-50 |
| SSUSC84_RS03715 | B9H01_03820 | hypothetical protein SSUSC84_0688 | -7.06367311 | 2.33219E-74 |
| SSUSC84_RS05440 | B9H01_05570 | Spx family transcriptional regulator | -7.68536628 | 2.0613E-228 |
| SSUSC84_RS05435 | B9H01_05565 | hypothetical protein SSU05_1146 | -7.729361123 | 2.5366E-147 |
