# Supplementary material for: The conserved transcription factor PrlP modulates colonization and pathogenicity of Streptococcus suis in response to environmental stress
Source: PLoS Pathog. 2025 Jul 18;21(7):e1013314. doi: 10.1371/journal.ppat.1013314 (PMC12273997; doi:10.1371/journal.ppat.1013314)
Supplement: S3 Table — (DOCX) [file ppat.1013314.s004.docx]

**Table S3.** Screen for genes containing the *PrlP* protein binding motif on the SC19 genome

| **Binding motif** | **motif location** | **name** | **Functional annotation** |
| --- | --- | --- | --- |
| CCTGAAWCT | INSIDE | B9H01_00040 | transcription-repair coupling factor |
| CCTGAAWCT | INSIDE | B9H01_00045 | RNA-binding S4 domain-containing protein |
| CCTGAAWCT | OVERLAP | B9H01_00050 | septum formation initiator family protein |
| CCTGAAWCT | BEFORE | B9H01_00060 | serine hydrolase |
| CCTGAAWCT | INSIDE | B9H01_00200 | CHAP domain-containing protein |
| CCTGAAWCT | INSIDE | B9H01_00205 | ribose-phosphate diphosphokinase |
| CCTGAAWCT | INSIDE | B9H01_00205 | ribose-phosphate diphosphokinase |
| CCTGAAWCT | INSIDE | B9H01_00210 | pyridoxal phosphate-dependent aminotransferase |
| CCTGAAWCT | INSIDE | B9H01_00220 | phosphate acyltransferase PlsX |
| CCTGAAWCT | INSIDE | B9H01_00225 | acyl carrier protein |
| CCTGAAWCT | INSIDE | B9H01_00225 | acyl carrier protein |
| CCTGAAWCT | INSIDE | B9H01_00240 | amidophosphoribosyltransferase |
| CCTGAAWCT | INSIDE | B9H01_00285 | adenylosuccinate lyase |
| CCTGAAWCT | INSIDE | B9H01_00340 | helix-turn-helix domain-containing protein |
| CCTGAAWCT | INSIDE | B9H01_00370 | Heme/copper-type cytochrome/quinol oxidase subunit 1 |
| CCTGAAWCT | INSIDE | B9H01_00445 | 50S ribosomal protein L3 |
| CCTGAAWCT | INSIDE | B9H01_00475 | 30S ribosomal protein S3 |
| CCTGAAWCT | INSIDE | B9H01_00560 | adenylate kinase |
| CCTGAAWCT | INSIDE | B9H01_00580 | 30S ribosomal protein S11 |
| CCTGAAWCT | INSIDE | B9H01_00690 | replication initiation factor domain-containing protein |
| CCTGAAWCT | BEFORE | B9H01_00720 | DUF2971 domain-containing protein |
| CCTGAAWCT | INSIDE | B9H01_00720 | DUF2971 domain-containing protein |
| CCTGAAWCT | BEFORE | B9H01_00760 | heavy-metal-associated domain-containing protein |
| CCTGAAWCT | INSIDE | B9H01_00870 | DUF1002 domain-containing protein |
| CCTGAAWCT | INSIDE | B9H01_00920 | M13 family metallopeptidase |
| CCTGAAWCT | BEFORE | B9H01_00925 | type I glyceraldehyde-3-phosphate dehydrogenase |
| CCTGAAWCT | INSIDE | B9H01_00990 | carbohydrate ABC transporter permease |
| CCTGAAWCT | BEFORE | B9H01_01015 | YSIRK-type signal peptide-containing protein |
| CCTGAAWCT | INSIDE | B9H01_01035 | ABC transporter ATP-binding protein |
| CCTGAAWCT | INSIDE | B9H01_01065 | PTS ascorbate transporter subunit IIC |
| CCTGAAWCT | INSIDE | B9H01_01070 | transketolase |
| CCTGAAWCT | BEFORE | B9H01_01095 | YSIRK signal domain/LPXTG anchor domain surface protein |
| CCTGAAWCT | INSIDE | B9H01_01095 | YSIRK signal domain/LPXTG anchor domain surface protein |
| CCTGAAWCT | BEFORE | B9H01_01105 | Xaa-Pro dipeptidyl-peptidase |
| CCTGAAWCT | INSIDE | B9H01_01125 | formate C-acetyltransferase |
| CCTGAAWCT | INSIDE | B9H01_01145 | tagatose-6-phosphate kinase |
| CCTGAAWCT | INSIDE | B9H01_01145 | tagatose-6-phosphate kinase |
| CCTGAAWCT | INSIDE | B9H01_01160 | ROK family protein |
| CCTGAAWCT | INSIDE | B9H01_01165 | PTS sugar transporter subunit IIC |
| CCTGAAWCT | INSIDE | B9H01_01175 | right-handed parallel beta-helix repeat-containing protein |
| CCTGAAWCT | INSIDE | B9H01_01175 | right-handed parallel beta-helix repeat-containing protein |
| CCTGAAWCT | INSIDE | B9H01_01175 | right-handed parallel beta-helix repeat-containing protein |
| CCTGAAWCT | INSIDE | B9H01_01175 | right-handed parallel beta-helix repeat-containing protein |
| CCTGAAWCT | INSIDE | B9H01_01175 | right-handed parallel beta-helix repeat-containing protein |
| CCTGAAWCT | INSIDE | B9H01_01180 | hypothetical protein |
| CCTGAAWCT | INSIDE | B9H01_01215 | HAD-IC family P-type ATPase |
| CCTGAAWCT | INSIDE | B9H01_01220 | ATP-dependent RecD-like DNA helicase |
| CCTGAAWCT | INSIDE | B9H01_01255 | trehalose operon repressor |
| CCTGAAWCT | INSIDE | B9H01_01255 | trehalose operon repressor |
| CCTGAAWCT | INSIDE | B9H01_01305 | DUF1430 domain-containing protein |
| CCTGAAWCT | BEFORE | B9H01_01315 | sodium:alanine symporter family protein |
| CCTGAAWCT | INSIDE | B9H01_01385 | DUF1700 domain-containing protein |
| CCTGAAWCT | OVERLAP | B9H01_01390 | PadR family transcriptional regulator |
| CCTGAAWCT | BEFORE | B9H01_01410 | aquaporin family protein |
| CCTGAAWCT | INSIDE | B9H01_01410 | aquaporin family protein |
| CCTGAAWCT | INSIDE | B9H01_01415 | DUF853 family protein |
| CCTGAAWCT | INSIDE | B9H01_01430 | LPXTG cell wall anchor domain-containing protein |
| CCTGAAWCT | INSIDE | B9H01_01430 | LPXTG cell wall anchor domain-containing protein |
| CCTGAAWCT | INSIDE | B9H01_01430 | LPXTG cell wall anchor domain-containing protein |
| CCTGAAWCT | INSIDE | B9H01_01430 | LPXTG cell wall anchor domain-containing protein |
| CCTGAAWCT | INSIDE | B9H01_01430 | LPXTG cell wall anchor domain-containing protein |
| CCTGAAWCT | INSIDE | B9H01_01430 | LPXTG cell wall anchor domain-containing protein |
| CCTGAAWCT | INSIDE | B9H01_01435 | methyl-accepting chemotaxis protein |
| CCTGAAWCT | INSIDE | B9H01_01440 | LPXTG cell wall anchor domain-containing protein |
| CCTGAAWCT | INSIDE | B9H01_01440 | LPXTG cell wall anchor domain-containing protein |
| CCTGAAWCT | INSIDE | B9H01_01440 | LPXTG cell wall anchor domain-containing protein |
| CCTGAAWCT | INSIDE | B9H01_01460 | alcohol dehydrogenase AdhP |
| CCTGAAWCT | BEFORE | B9H01_01505 | mevalonate kinase |
| CCTGAAWCT | INSIDE | B9H01_01530 | ATP-binding cassette domain-containing protein |
| CCTGAAWCT | INSIDE | B9H01_01565 | molecular chaperone DnaJ |
| CCTGAAWCT | INSIDE | B9H01_01580 | amidase |
| CCTGAAWCT | INSIDE | B9H01_01610 | transcriptional repressor |
| CCTGAAWCT | BEFORE | B9H01_01615 | tRNA pseudouridine(38-40) synthase TruA |
| CCTGAAWCT | INSIDE | B9H01_01615 | tRNA pseudouridine(38-40) synthase TruA |
| CCTGAAWCT | INSIDE | B9H01_01645 | Rrf2 family transcriptional regulator |
| CCTGAAWCT | INSIDE | B9H01_01675 | TetR family transcriptional regulator |
| CCTGAAWCT | INSIDE | B9H01_01690 | trigger factor |
| CCTGAAWCT | BEFORE | B9H01_01775 | Cof-type HAD-IIB family hydrolase |
| CCTGAAWCT | INSIDE | B9H01_01780 | haloacid dehalogenase |
| CCTGAAWCT | INSIDE | B9H01_01805 | cysteine hydrolase |
| CCTGAAWCT | INSIDE | B9H01_01870 | YqeG family HAD IIIA-type phosphatase |
| CCTGAAWCT | INSIDE | B9H01_01945 | ATP-binding protein |
| CCTGAAWCT | INSIDE | B9H01_01945 | ATP-binding protein |
| CCTGAAWCT | INSIDE | B9H01_01945 | ATP-binding protein |
| CCTGAAWCT | INSIDE | B9H01_01990 | PTS transporter subunit EIIC |
| CCTGAAWCT | INSIDE | B9H01_01990 | PTS transporter subunit EIIC |
| CCTGAAWCT | BEFORE | B9H01_02025 | rhodanese-related sulfurtransferase |
| CCTGAAWCT | INSIDE | B9H01_02035 | ABC transporter ATP-binding protein |
| CCTGAAWCT | INSIDE | B9H01_02055 | penicillin-binding protein PBP1a |
| CCTGAAWCT | INSIDE | B9H01_02055 | penicillin-binding protein PBP1a |
| CCTGAAWCT | INSIDE | B9H01_02110 | primosomal protein N' |
| CCTGAAWCT | INSIDE | B9H01_02140 | sensor histidine kinase |
| CCTGAAWCT | INSIDE | B9H01_02150 | Cof-type HAD-IIB family hydrolase |
| CCTGAAWCT | INSIDE | B9H01_02260 | DUF402 domain-containing protein |
| CCTGAAWCT | INSIDE | B9H01_02265 | DUF960 domain-containing protein |
| CCTGAAWCT | INSIDE | B9H01_02275 | GntR family transcriptional regulator |
| CCTGAAWCT | INSIDE | B9H01_02285 | PTS system mannose/fructose/N-acetylgalactosamine-transporter subunit IIB |
| CCTGAAWCT | INSIDE | B9H01_02335 | hypothetical protein |
| CCTGAAWCT | INSIDE | B9H01_02335 | hypothetical protein |
| CCTGAAWCT | INSIDE | B9H01_02370 | translational GTPase TypA |
| CCTGAAWCT | INSIDE | B9H01_02395 | InlB B-repeat-containing protein |
| CCTGAAWCT | INSIDE | B9H01_02395 | InlB B-repeat-containing protein |
| CCTGAAWCT | INSIDE | B9H01_02425 | FtsQ-type POTRA domain-containing protein |
| CCTGAAWCT | BEFORE | B9H01_02465 | GNAT family N-acetyltransferase |
| CCTGAAWCT | INSIDE | B9H01_02465 | GNAT family N-acetyltransferase |
| CCTGAAWCT | INSIDE | B9H01_02470 | isoleucine--tRNA ligase |
| CCTGAAWCT | INSIDE | B9H01_02560 | U32 family peptidase |
| CCTGAAWCT | INSIDE | B9H01_02580 | biotin transporter BioY |
| CCTGAAWCT | INSIDE | B9H01_02590 | glutathione-disulfide reductase |
| CCTGAAWCT | INSIDE | B9H01_02625 | histidine phosphatase family protein |
| CCTGAAWCT | INSIDE | B9H01_02670 | RNA polymerase sigma factor |
| CCTGAAWCT | INSIDE | B9H01_02680 | elongation factor Tu |
| CCTGAAWCT | INSIDE | B9H01_02715 | adenosine deaminase |
| CCTGAAWCT | INSIDE | B9H01_02735 | DNA polymerase III subunit alpha |
| CCTGAAWCT | INSIDE | B9H01_02735 | DNA polymerase III subunit alpha |
| CCTGAAWCT | INSIDE | B9H01_02750 | IdeS family IgM protease |
| CCTGAAWCT | INSIDE | B9H01_02750 | IdeS family IgM protease |
| CCTGAAWCT | INSIDE | B9H01_02750 | IdeS family IgM protease |
| CCTGAAWCT | INSIDE | B9H01_02750 | IdeS family IgM protease |
| CCTGAAWCT | INSIDE | B9H01_02750 | IdeS family IgM protease |
| CCTGAAWCT | INSIDE | B9H01_02750 | IdeS family IgM protease |
| CCTGAAWCT | INSIDE | B9H01_02750 | IdeS family IgM protease |
| CCTGAAWCT | INSIDE | B9H01_02770 | glutamine--fructose-6-phosphate transaminase (isomerizing) |
| CCTGAAWCT | BEFORE | B9H01_02795 | MBL fold metallo-hydrolase |
| CCTGAAWCT | INSIDE | B9H01_02825 | DegV family protein |
| CCTGAAWCT | BEFORE | B9H01_02835 | peroxide stress protein YaaA |
| CCTGAAWCT | INSIDE | B9H01_02835 | peroxide stress protein YaaA |
| CCTGAAWCT | INSIDE | B9H01_02865 | glycosyltransferase family 1 protein |
| CCTGAAWCT | INSIDE | B9H01_03005 | hypothetical protein |
| CCTGAAWCT | INSIDE | B9H01_03005 | hypothetical protein |
| CCTGAAWCT | INSIDE | B9H01_03005 | hypothetical protein |
| CCTGAAWCT | INSIDE | B9H01_03020 | prephenate dehydratase |
| CCTGAAWCT | INSIDE | B9H01_03030 | 23S rRNA (uracil(1939)-C(5))-methyltransferase RlmD |
| CCTGAAWCT | INSIDE | B9H01_03040 | UDP-galactopyranose mutase |
| CCTGAAWCT | INSIDE | B9H01_03065 | pyridoxal phosphate-dependent aminotransferase |
| CCTGAAWCT | BEFORE | B9H01_03095 | MATE family efflux transporter |
| CCTGAAWCT | INSIDE | B9H01_03100 | RidA family protein |
| CCTGAAWCT | BEFORE | B9H01_03160 | arginine repressor |
| CCTGAAWCT | INSIDE | B9H01_03165 | tRNA preQ1(34) S-adenosylmethionine ribosyltransferase-isomerase QueA |
| CCTGAAWCT | INSIDE | B9H01_03205 | D-alanyl-lipoteichoic acid biosynthesis protein DltB |
| CCTGAAWCT | INSIDE | B9H01_03205 | D-alanyl-lipoteichoic acid biosynthesis protein DltB |
| CCTGAAWCT | INSIDE | B9H01_03230 | rRNA pseudouridine synthase |
| CCTGAAWCT | INSIDE | B9H01_03240 | iron ABC transporter permease |
| CCTGAAWCT | INSIDE | B9H01_03285 | hypothetical protein |
| CCTGAAWCT | INSIDE | B9H01_03305 | DNA polymerase III subunit delta' |
| CCTGAAWCT | INSIDE | B9H01_03330 | phosphoglycerate dehydrogenase |
| CCTGAAWCT | INSIDE | B9H01_03355 | hypothetical protein |
| CCTGAAWCT | INSIDE | B9H01_03355 | hypothetical protein |
| CCTGAAWCT | INSIDE | B9H01_03415 | DEAD/DEAH box helicase family protein |
| CCTGAAWCT | INSIDE | B9H01_03460 | type I restriction endonuclease subunit R |
| CCTGAAWCT | INSIDE | B9H01_03460 | type I restriction endonuclease subunit R |
| CCTGAAWCT | INSIDE | B9H01_03505 | glycyl-radical enzyme activating protein |
| CCTGAAWCT | INSIDE | B9H01_03530 | PTS sugar transporter subunit IIC |
| CCTGAAWCT | INSIDE | B9H01_03530 | PTS sugar transporter subunit IIC |
| CCTGAAWCT | INSIDE | B9H01_03535 | glycyl radical protein |
| CCTGAAWCT | INSIDE | B9H01_03555 | aspartate-semialdehyde dehydrogenase |
| CCTGAAWCT | INSIDE | B9H01_03580 | type 1 glycerol-3-phosphate oxidase |
| CCTGAAWCT | INSIDE | B9H01_03615 | DNA helicase PcrA |
| CCTGAAWCT | INSIDE | B9H01_03625 | bifunctional hydroxymethylpyrimidine kinase/phosphomethylpyrimidine kinase |
| CCTGAAWCT | INSIDE | B9H01_03700 | ABC transporter ATP-binding protein |
| CCTGAAWCT | INSIDE | B9H01_03770 | DNA topoisomerase IV subunit A |
| CCTGAAWCT | INSIDE | B9H01_03800 | 30S ribosomal protein S1 |
| CCTGAAWCT | INSIDE | B9H01_03805 | tRNA-Arg |
| CCTGAAWCT | INSIDE | B9H01_03810 | threonine aldolase |
| CCTGAAWCT | INSIDE | B9H01_03830 | tRNA 4-thiouridine(8) synthase ThiI |
| CCTGAAWCT | INSIDE | B9H01_03895 | carbamoyl-phosphate synthase large subunit |
| CCTGAAWCT | INSIDE | B9H01_03895 | carbamoyl-phosphate synthase large subunit |
| CCTGAAWCT | INSIDE | B9H01_03935 | polysaccharide deacetylase family protein |
| CCTGAAWCT | INSIDE | B9H01_03945 | homoserine kinase |
| CCTGAAWCT | INSIDE | B9H01_03970 | ABC transporter substrate-binding protein |
| CCTGAAWCT | INSIDE | B9H01_03990 | S8 family serine peptidase |
| CCTGAAWCT | INSIDE | B9H01_04010 | MATE family efflux transporter |
| CCTGAAWCT | INSIDE | B9H01_04060 | CCA tRNA nucleotidyltransferase |
| CCTGAAWCT | INSIDE | B9H01_04065 | ABC-F family ATP-binding cassette domain-containing protein |
| CCTGAAWCT | INSIDE | B9H01_04080 | hypothetical protein |
| CCTGAAWCT | INSIDE | B9H01_04090 | thymidylate synthase |
| CCTGAAWCT | INSIDE | B9H01_04135 | excalibur calcium-binding domain-containing protein |
| CCTGAAWCT | INSIDE | B9H01_04135 | excalibur calcium-binding domain-containing protein |
| CCTGAAWCT | INSIDE | B9H01_04160 | threonylcarbamoyl-AMP synthase |
| CCTGAAWCT | INSIDE | B9H01_04170 | serine hydroxymethyltransferase |
| CCTGAAWCT | INSIDE | B9H01_04190 | translation initiation factor 1 |
| CCTGAAWCT | INSIDE | B9H01_04205 | hypothetical protein |
| CCTGAAWCT | INSIDE | B9H01_04205 | hypothetical protein |
| CCTGAAWCT | INSIDE | B9H01_04220 | site-specific integrase |
| CCTGAAWCT | INSIDE | B9H01_04220 | site-specific integrase |
| CCTGAAWCT | INSIDE | B9H01_04225 | DUF3173 domain-containing protein |
| CCTGAAWCT | INSIDE | B9H01_04245 | hypothetical protein |
| CCTGAAWCT | INSIDE | B9H01_04255 | membrane protein |
| CCTGAAWCT | INSIDE | B9H01_04265 | hypothetical protein |
| CCTGAAWCT | INSIDE | B9H01_04275 | hypothetical protein |
| CCTGAAWCT | INSIDE | B9H01_04295 | signal recognition particle protein |
| CCTGAAWCT | INSIDE | B9H01_04305 | DUF3307 domain-containing protein |
| CCTGAAWCT | INSIDE | B9H01_04375 | ABC transporter ATP-binding protein |
| CCTGAAWCT | INSIDE | B9H01_04390 | carbamoyl phosphate synthase large subunit |
| CCTGAAWCT | INSIDE | B9H01_04425 | site-specific integrase |
| CCTGAAWCT | INSIDE | B9H01_04450 | ABC transporter permease |
| CCTGAAWCT | BEFORE | B9H01_04495 | ATP-binding protein |
| CCTGAAWCT | INSIDE | B9H01_04495 | ATP-binding protein |
| CCTGAAWCT | INSIDE | B9H01_04500 | tyrosine-type recombinase/integrase |
| CCTGAAWCT | INSIDE | B9H01_04580 | replication initiation factor domain-containing protein |
| CCTGAAWCT | INSIDE | B9H01_04590 | DNA translocase FtsK |
| CCTGAAWCT | INSIDE | B9H01_04655 | ATP-binding protein |
| CCTGAAWCT | INSIDE | B9H01_04675 | ATP-binding cassette domain-containing protein |
| CCTGAAWCT | INSIDE | B9H01_04695 | recombinase family protein |
| CCTGAAWCT | INSIDE | B9H01_04750 | DEAD/DEAH box helicase family protein |
| CCTGAAWCT | INSIDE | B9H01_04750 | DEAD/DEAH box helicase family protein |
| CCTGAAWCT | INSIDE | B9H01_04750 | DEAD/DEAH box helicase family protein |
| CCTGAAWCT | INSIDE | B9H01_04765 | LPXTG cell wall anchor domain-containing protein |
| CCTGAAWCT | INSIDE | B9H01_04785 | ATPase AAA |
| CCTGAAWCT | INSIDE | B9H01_04815 | CPBP family intramembrane metalloprotease |
| CCTGAAWCT | INSIDE | B9H01_04840 | replication initiator protein A |
| CCTGAAWCT | BEFORE | B9H01_04850 | 50S ribosomal protein L7/L12 |
| CCTGAAWCT | INSIDE | B9H01_04880 | rhodanese-like domain-containing protein |
| CCTGAAWCT | INSIDE | B9H01_04925 | 5'-nucleotidase C-terminal domain-containing protein |
| CCTGAAWCT | INSIDE | B9H01_04925 | 5'-nucleotidase C-terminal domain-containing protein |
| CCTGAAWCT | INSIDE | B9H01_04925 | 5'-nucleotidase C-terminal domain-containing protein |
| CCTGAAWCT | INSIDE | B9H01_04930 | dihydroorotase |
| CCTGAAWCT | INSIDE | B9H01_04945 | orotate phosphoribosyltransferase |
| CCTGAAWCT | INSIDE | B9H01_04995 | transporter substrate-binding domain-containing protein |
| CCTGAAWCT | INSIDE | B9H01_05025 | DUF2971 domain-containing protein |
| CCTGAAWCT | INSIDE | B9H01_05030 | excinuclease ABC subunit B |
| CCTGAAWCT | INSIDE | B9H01_05070 | aldose 1-epimerase family protein |
| CCTGAAWCT | INSIDE | B9H01_05075 | 6-phospho-beta-galactosidase |
| CCTGAAWCT | INSIDE | B9H01_05150 | hypothetical protein |
| CCTGAAWCT | INSIDE | B9H01_05165 | PaaI family thioesterase |
| CCTGAAWCT | INSIDE | B9H01_05185 | RluA family pseudouridine synthase |
| CCTGAAWCT | BEFORE | B9H01_05205 | ribose-phosphate diphosphokinase |
| CCTGAAWCT | INSIDE | B9H01_05245 | DNA gyrase subunit A |
| CCTGAAWCT | INSIDE | B9H01_05260 | ABC transporter permease |
| CCTGAAWCT | INSIDE | B9H01_05260 | ABC transporter permease |
| CCTGAAWCT | INSIDE | B9H01_05320 | transglutaminase |
| CCTGAAWCT | INSIDE | B9H01_05345 | IS110 family transposase |
| CCTGAAWCT | INSIDE | B9H01_05390 | UPF0223 family protein |
| CCTGAAWCT | INSIDE | B9H01_05455 | DUF2130 domain-containing protein |
| CCTGAAWCT | BEFORE | B9H01_05575 | hypothetical protein |
| CCTGAAWCT | INSIDE | B9H01_05585 | phenylalanine--tRNA ligase subunit beta |
| CCTGAAWCT | INSIDE | B9H01_05605 | beta-hexosamidase |
| CCTGAAWCT | INSIDE | B9H01_05630 | bifunctional 4-hydroxy-2-oxoglutarate aldolase/2-dehydro-3-deoxy-phosphogluconate aldolase |
| CCTGAAWCT | INSIDE | B9H01_05645 | sugar kinase |
| CCTGAAWCT | INSIDE | B9H01_05695 | F0F1 ATP synthase subunit delta |
| CCTGAAWCT | INSIDE | B9H01_05730 | TIGR03943 family protein |
| CCTGAAWCT | INSIDE | B9H01_05760 | HAD family hydrolase |
| CCTGAAWCT | INSIDE | B9H01_05785 | MFS transporter |
| CCTGAAWCT | INSIDE | B9H01_05810 | NADP-dependent isocitrate dehydrogenase |
| CCTGAAWCT | INSIDE | B9H01_05820 | aconitate hydratase AcnA |
| CCTGAAWCT | INSIDE | B9H01_05825 | glutaredoxin-like protein NrdH |
| CCTGAAWCT | INSIDE | B9H01_05850 | alginate lyase family protein |
| CCTGAAWCT | INSIDE | B9H01_05855 | LPXTG cell wall anchor domain-containing protein |
| CCTGAAWCT | INSIDE | B9H01_05870 | YSIRK-type signal peptide-containing protein |
| CCTGAAWCT | INSIDE | B9H01_05890 | PTS system mannose/fructose/N-acetylgalactosamine-transporter subunit IIB |
| CCTGAAWCT | INSIDE | B9H01_05905 | bifunctional 4-hydroxy-2-oxoglutarate aldolase/2-dehydro-3-deoxy-phosphogluconate aldolase |
| CCTGAAWCT | INSIDE | B9H01_05965 | DUF3267 domain-containing protein |
| CCTGAAWCT | INSIDE | B9H01_06015 | oligoendopeptidase F |
| CCTGAAWCT | INSIDE | B9H01_06045 | shikimate dehydrogenase |
| CCTGAAWCT | INSIDE | B9H01_06090 | N-acetylglucosamine-6-phosphate deacetylase |
| CCTGAAWCT | INSIDE | B9H01_06095 | YlbF/YmcA family competence regulator |
| CCTGAAWCT | INSIDE | B9H01_06110 | type I 3-dehydroquinate dehydratase |
| CCTGAAWCT | INSIDE | B9H01_06120 | LTA synthase family protein |
| CCTGAAWCT | INSIDE | B9H01_06190 | glycosyltransferase |
| CCTGAAWCT | INSIDE | B9H01_06200 | glycosyltransferase family 8 protein |
| CCTGAAWCT | INSIDE | B9H01_06215 | rhamnan synthesis protein F |
| CCTGAAWCT | INSIDE | B9H01_06215 | rhamnan synthesis protein F |
| CCTGAAWCT | INSIDE | B9H01_06240 | alkaline phosphatase family protein |
| CCTGAAWCT | INSIDE | B9H01_06255 | LPXTG cell wall anchor domain-containing protein |
| CCTGAAWCT | INSIDE | B9H01_06270 | hypothetical protein |
| CCTGAAWCT | INSIDE | B9H01_06295 | tRNA (adenine-N(1))-methyltransferase |
| CCTGAAWCT | INSIDE | B9H01_06325 | single-stranded-DNA-specific exonuclease RecJ |
| CCTGAAWCT | INSIDE | B9H01_06375 | cobalamin-independent methionine synthase II family protein |
| CCTGAAWCT | INSIDE | B9H01_06390 | DUF1801 domain-containing protein |
| CCTGAAWCT | INSIDE | B9H01_06435 | 50S ribosomal protein L11 |
| CCTGAAWCT | INSIDE | B9H01_06450 | DNA translocase FtsK |
| CCTGAAWCT | INSIDE | B9H01_06475 | LacI family DNA-binding transcriptional regulator |
| CCTGAAWCT | INSIDE | B9H01_06490 | DUF2500 domain-containing protein |
| CCTGAAWCT | INSIDE | B9H01_06510 | cation-translocating P-type ATPase |
| CCTGAAWCT | INSIDE | B9H01_06550 | ATP-binding cassette domain-containing protein |
| CCTGAAWCT | INSIDE | B9H01_06570 | amino acid ABC transporter ATP-binding protein |
| CCTGAAWCT | INSIDE | B9H01_06590 | threonine--tRNA ligase |
| CCTGAAWCT | INSIDE | B9H01_06615 | LPXTG cell wall anchor domain-containing protein |
| CCTGAAWCT | INSIDE | B9H01_06625 | aminopeptidase P family protein |
| CCTGAAWCT | INSIDE | B9H01_06650 | DNA polymerase I |
| CCTGAAWCT | INSIDE | B9H01_06660 | FtsX-like permease family protein |
| CCTGAAWCT | INSIDE | B9H01_06680 | copper-translocating P-type ATPase |
| CCTGAAWCT | INSIDE | B9H01_06685 | C69 family dipeptidase |
| CCTGAAWCT | BEFORE | B9H01_06740 | GTPase Era |
| CCTGAAWCT | BEFORE | B9H01_06770 | cholesterol-dependent cytolysin suilysin |
| CCTGAAWCT | INSIDE | B9H01_06770 | cholesterol-dependent cytolysin suilysin |
| CCTGAAWCT | INSIDE | B9H01_06890 | ISAs1 family transposase |
| CCTGAAWCT | INSIDE | B9H01_06910 | DNA primase |
| CCTGAAWCT | INSIDE | B9H01_06910 | DNA primase |
| CCTGAAWCT | INSIDE | B9H01_06990 | restriction endonuclease subunit S |
| CCTGAAWCT | INSIDE | B9H01_07005 | DEAD/DEAH box helicase family protein |
| CCTGAAWCT | INSIDE | B9H01_07005 | DEAD/DEAH box helicase family protein |
| CCTGAAWCT | INSIDE | B9H01_07050 | dipeptidase PepV |
| CCTGAAWCT | BEFORE | B9H01_07170 | ABC transporter ATP-binding protein |
| CCTGAAWCT | INSIDE | B9H01_07200 | fibronectin/fibrinogen-binding protein |
| CCTGAAWCT | INSIDE | B9H01_07200 | fibronectin/fibrinogen-binding protein |
| CCTGAAWCT | INSIDE | B9H01_07270 | IS110 family transposase |
| CCTGAAWCT | INSIDE | B9H01_07285 | DNA topoisomerase (ATP-hydrolyzing) subunit B |
| CCTGAAWCT | INSIDE | B9H01_07285 | DNA topoisomerase (ATP-hydrolyzing) subunit B |
| CCTGAAWCT | INSIDE | B9H01_07380 | DUF3800 domain-containing protein |
| CCTGAAWCT | INSIDE | B9H01_07430 | bifunctional metallophosphatase/5'-nucleotidase |
| CCTGAAWCT | INSIDE | B9H01_07460 | ABC transporter ATP-binding protein |
| CCTGAAWCT | INSIDE | B9H01_07505 | carbohydrate ABC transporter permease |
| CCTGAAWCT | INSIDE | B9H01_07605 | PH domain-containing protein |
| CCTGAAWCT | INSIDE | B9H01_07610 | pneumococcal-type histidine triad protein |
| CCTGAAWCT | INSIDE | B9H01_07630 | 5'-methylthioadenosine/adenosylhomocysteine nucleosidase |
| CCTGAAWCT | INSIDE | B9H01_07670 | hypothetical protein |
| CCTGAAWCT | INSIDE | B9H01_07710 | DUF2974 domain-containing protein |
| CCTGAAWCT | INSIDE | B9H01_07720 | nuclear transport factor 2 family protein |
| CCTGAAWCT | INSIDE | B9H01_07820 | GNAT family N-acetyltransferase |
| CCTGAAWCT | INSIDE | B9H01_07835 | methionine adenosyltransferase |
| CCTGAAWCT | INSIDE | B9H01_07865 | DNA polymerase III subunit gamma/tau |
| CCTGAAWCT | INSIDE | B9H01_07950 | IS110 family transposase |
| CCTGAAWCT | INSIDE | B9H01_07965 | SGNH/GDSL hydrolase family protein |
| CCTGAAWCT | INSIDE | B9H01_08015 | HAMP domain-containing histidine kinase |
| CCTGAAWCT | INSIDE | B9H01_08035 | YSIRK-type signal peptide-containing protein |
| CCTGAAWCT | INSIDE | B9H01_08065 | methyltransferase domain-containing protein |
| CCTGAAWCT | INSIDE | B9H01_08090 | 23S rRNA (adenine(2503)-C(2))-methyltransferase RlmN |
| CCTGAAWCT | INSIDE | B9H01_08235 | glutamate racemase |
| CCTGAAWCT | INSIDE | B9H01_08250 | aminopeptidase C |
| CCTGAAWCT | INSIDE | B9H01_08250 | aminopeptidase C |
| CCTGAAWCT | INSIDE | B9H01_08270 | acylphosphatase |
| CCTGAAWCT | INSIDE | B9H01_08295 | UDP-N-acetylmuramate--L-alanine ligase |
| CCTGAAWCT | INSIDE | B9H01_08340 | replication initiation/membrane attachment protein |
| CCTGAAWCT | INSIDE | B9H01_08380 | DEAD/DEAH box helicase |
| CCTGAAWCT | INSIDE | B9H01_08380 | DEAD/DEAH box helicase |
| CCTGAAWCT | INSIDE | B9H01_08395 | penicillin-binding protein PBP2X |
| CCTGAAWCT | INSIDE | B9H01_08435 | hypothetical protein |
| CCTGAAWCT | INSIDE | B9H01_08555 | methylenetetrahydrofolate reductase [NAD(P)H] |
| CCTGAAWCT | INSIDE | B9H01_08585 | DUF956 family protein |
| CCTGAAWCT | INSIDE | B9H01_08590 | serine--tRNA ligase |
| CCTGAAWCT | INSIDE | B9H01_08595 | type I restriction-modification system subunit M |
| CCTGAAWCT | INSIDE | B9H01_08605 | type I restriction endonuclease subunit R |
| CCTGAAWCT | INSIDE | B9H01_08665 | beta-ketoacyl-ACP synthase II |
| CCTGAAWCT | OVERLAP | B9H01_08665 | beta-ketoacyl-ACP synthase II |
| CCTGAAWCT | INSIDE | B9H01_08675 | ACP S-malonyltransferase |
| CCTGAAWCT | INSIDE | B9H01_08735 | preprotein translocase subunit SecA |
| CCTGAAWCT | INSIDE | B9H01_08740 | ribonuclease |
| CCTGAAWCT | INSIDE | B9H01_08740 | ribonuclease |
| CCTGAAWCT | INSIDE | B9H01_08775 | elongation factor P |
| CCTGAAWCT | INSIDE | B9H01_08785 | excinuclease ABC subunit UvrA |
| CCTGAAWCT | INSIDE | B9H01_08840 | dihydrolipoamide acetyltransferase |
| CCTGAAWCT | INSIDE | B9H01_08845 | alpha-ketoacid dehydrogenase subunit beta |
| CCTGAAWCT | INSIDE | B9H01_08865 | lipoprotein |
| CCTGAAWCT | INSIDE | B9H01_08930 | methyl-accepting chemotaxis protein |
| CCTGAAWCT | INSIDE | B9H01_08955 | Asp23/Gls24 family envelope stress response protein |
| CCTGAAWCT | INSIDE | B9H01_08960 | IS110 family transposase |
| CCTGAAWCT | INSIDE | B9H01_09030 | undecaprenyl-diphosphate phosphatase |
| CCTGAAWCT | INSIDE | B9H01_09085 | hypothetical protein |
| CCTGAAWCT | INSIDE | B9H01_09090 | dihydroxy-acid dehydratase |
| CCTGAAWCT | BEFORE | B9H01_09160 | helix-turn-helix domain-containing protein |
| CCTGAAWCT | INSIDE | B9H01_09165 | response regulator transcription factor |
| CCTGAAWCT | INSIDE | B9H01_09180 | IS110 family transposase |
| CCTGAAWCT | INSIDE | B9H01_09230 | bacterial Ig-like domain-containing protein |
| CCTGAAWCT | BEFORE | B9H01_09270 | GNAT family N-acetyltransferase |
| CCTGAAWCT | INSIDE | B9H01_09285 | PTS transporter subunit EIIC |
| CCTGAAWCT | INSIDE | B9H01_09285 | PTS transporter subunit EIIC |
| CCTGAAWCT | BEFORE | B9H01_09345 | Sua5/YciO/YrdC/YwlC family protein |
| CCTGAAWCT | INSIDE | B9H01_09365 | 2-isopropylmalate synthase |
| CCTGAAWCT | INSIDE | B9H01_09385 | LemA family protein |
| CCTGAAWCT | INSIDE | B9H01_09445 | preprotein translocase subunit YajC |
| CCTGAAWCT | BEFORE | B9H01_09455 | bifunctional glutamate--cysteine ligase GshA/glutathione synthetase GshB |
| CCTGAAWCT | INSIDE | B9H01_09460 | LPXTG cell wall anchor domain-containing protein |
| CCTGAAWCT | INSIDE | B9H01_09465 | Hsp33 family molecular chaperone HslO |
| CCTGAAWCT | INSIDE | B9H01_09570 | phosphotransferase |
| CCTGAAWCT | INSIDE | B9H01_09570 | phosphotransferase |
| CCTGAAWCT | INSIDE | B9H01_09580 | DNA recombination protein RmuC |
| CCTGAAWCT | INSIDE | B9H01_09590 | ribulose-phosphate 3-epimerase |
| CCTGAAWCT | INSIDE | B9H01_09630 | membrane protein |
| CCTGAAWCT | INSIDE | B9H01_09790 | ABC transporter ATP-binding protein |
| CCTGAAWCT | INSIDE | B9H01_09830 | 2,3,4,5-tetrahydropyridine-2,6-dicarboxylate N-acetyltransferase |
| CCTGAAWCT | INSIDE | B9H01_09870 | transketolase |
| CCTGAAWCT | INSIDE | B9H01_09870 | transketolase |
| CCTGAAWCT | INSIDE | B9H01_09895 | L-ribulose-5-phosphate 3-epimerase |
| CCTGAAWCT | INSIDE | B9H01_09920 | pullulanase |
| CCTGAAWCT | INSIDE | B9H01_09985 | 6-phospho-beta-glucosidase |
| CCTGAAWCT | INSIDE | B9H01_10040 | CPBP family intramembrane metalloprotease |
| CCTGAAWCT | BEFORE | B9H01_10045 | response regulator transcription factor |
| CCTGAAWCT | INSIDE | B9H01_10045 | response regulator transcription factor |
| CCTGAAWCT | BEFORE | B9H01_10070 | heme-binding protein SntA |
| CCTGAAWCT | INSIDE | B9H01_10070 | heme-binding protein SntA |
| CCTGAAWCT | INSIDE | B9H01_10070 | heme-binding protein SntA |
| CCTGAAWCT | INSIDE | B9H01_10105 | isopeptide-forming domain-containing fimbrial protein |
| CCTGAAWCT | INSIDE | B9H01_10110 | discoidin domain-containing protein |
| CCTGAAWCT | INSIDE | B9H01_10115 | carboxypeptidase regulatory-like domain-containing protein |
| CCTGAAWCT | INSIDE | B9H01_10115 | carboxypeptidase regulatory-like domain-containing protein |
| CCTGAAWCT | INSIDE | B9H01_10135 | 50S ribosomal protein L11 methyltransferase |
| CCTGAAWCT | INSIDE | B9H01_10280 | type I pullulanase |
| CCTGAAWCT | INSIDE | B9H01_10290 | YitT family protein |
| CCTGAAWCT | INSIDE | B9H01_10310 | glycosyltransferase family 2 protein |
| CCTGAAWCT | OVERLAP | B9H01_10315 | glycoside hydrolase family 3 C-terminal domain-containing protein |
| CCTGAAWCT | INSIDE | B9H01_10340 | metallophosphoesterase |
| CCTGAAWCT | INSIDE | B9H01_10400 | NUDIX hydrolase |
| CCTGAAWCT | INSIDE | B9H01_10485 | tryptophan--tRNA ligase |
| CCTGAAWCT | INSIDE | B9H01_10490 | YitT family protein |
| CCTGAAWCT | INSIDE | B9H01_10500 | YfhO family protein |
| CCTGAAWCT | INSIDE | B9H01_10500 | YfhO family protein |
