# Supplementary material for: The conserved transcription factor PrlP modulates colonization and pathogenicity of Streptococcus suis in response to environmental stress
Source: PLoS Pathog. 2025 Jul 18;21(7):e1013314. doi: 10.1371/journal.ppat.1013314 (PMC12273997; doi:10.1371/journal.ppat.1013314)
Supplement: S4 Table — (DOCX) [file ppat.1013314.s005.docx]

**T****able S4.** Genes directly regulated by PrlP in the SC19 genome

| Name | motif location | log2Fold Change | p-value | Functional annotation |
| --- | --- | --- | --- | --- |
| B9H01_05150 | INSIDE | 5.55 | 4.2E-173 | hypothetical protein |
| B9H01_08740 | INSIDE | 4.26 | 5E-176 | ribonuclease |
| B9H01_00920 | INSIDE | 4.18 | 1.9E-175 | M13 family metallopeptidase |
| B9H01_10110 | INSIDE | 3.82 | 1.8E-142 | discoidin domain-containing protein |
| B9H01_10115 | INSIDE | 3.47 | 2.2E-109 | carboxypeptidase regulatory-like domain-containing protein |
| B9H01_01435 | INSIDE | 3.44 | 5.4E-105 | methyl-accepting chemotaxis protein |
| B9H01_08035 | INSIDE | 3.43 | 1.7E-118 | YSIRK-type signal peptide-containing protein |
| B9H01_01440 | INSIDE | 3.19 | 9.5E-111 | LPXTG cell wall anchor domain-containing protein |
| B9H01_06615 | INSIDE | 3.12 | 1.5E-109 | LPXTG cell wall anchor domain-containing protein |
| B9H01_01430 | INSIDE | 2.78 | 5.15E-90 | LPXTG cell wall anchor domain-containing protein |
| B9H01_03240 | INSIDE | 2.45 | 1.42E-29 | iron ABC transporter permease |
| B9H01_01095 | BEFORE | 2.01 | 3.79E-50 | YSIRK signal domain/LPXTG anchor domain surface protein |
| B9H01_06570 | INSIDE | 1.97 | 8.77E-22 | amino acid ABC transporter ATP-binding protein |
| B9H01_07170 | BEFORE | 1.94 | 2.69E-38 | ABC transporter ATP-binding protein |
| B9H01_06375 | INSIDE | 1.90 | 5.94E-26 | cobalamin-independent methionine synthase II family protein |
| B9H01_02580 | INSIDE | 1.71 | 6.04E-29 | biotin transporter BioY |
| B9H01_05810 | INSIDE | 1.62 | 1.29E-13 | NADP-dependent isocitrate dehydrogenase |
| B9H01_07270 | INSIDE | 1.53 | 1.1E-07 | IS110 family transposase |
| B9H01_04880 | INSIDE | 1.52 | 1.3E-06 | rhodanese-like domain-containing protein |
| B9H01_08665 | INSIDE | 1.46 | 2.33E-27 | beta-ketoacyl-ACP synthase II |
| B9H01_05820 | INSIDE | 1.17 | 1.21E-07 | aconitate hydratase AcnA |
| B9H01_02285 | INSIDE | -1.19 | 0.001393 | PTS system mannose/fructose/N-acetylgalactosamine-transporter subunit IIB |
| B9H01_02590 | INSIDE | -1.22 | 5.13E-18 | glutathione-disulfide reductase |
| B9H01_05890 | INSIDE | -1.28 | 0.001297 | PTS system mannose/fructose/N-acetylgalactosamine-transporter subunit IIB |
| B9H01_03095 | BEFORE | -1.36 | 7.02E-24 | MATE family efflux transporter |
| B9H01_04190 | INSIDE | -1.50 | 5.09E-24 | translation initiation factor 1 |
| B9H01_01610 | INSIDE | -1.56 | 8.99E-26 | transcriptional repressor |
| B9H01_06240 | INSIDE | -1.56 | 4.28E-21 | alkaline phosphatase family protein |
| B9H01_02335 | INSIDE | -1.94 | 1.93E-43 | hypothetical protein |
| B9H01_07380 | INSIDE | -2.02 | 9.36E-39 | DUF3800 domain-containing protein |
| B9H01_04425 | INSIDE | -2.16 | 1.71E-19 | site-specific integrase |
| B9H01_03005 | INSIDE | -2.18 | 6.84E-17 | hypothetical protein |
| B9H01_04275 | INSIDE | -2.85 | 2.83E-23 | hypothetical protein |
| B9H01_04245 | INSIDE | -3.03 | 1.65E-68 | hypothetical protein |
| B9H01_05320 | INSIDE | -3.39 | 4.3E-122 | transglutaminase |
| B9H01_00045 | INSIDE | -1.17 | 0.022793 | RNA-binding S4 domain-containing protein |
| B9H01_10225 | INSIDE | -1.80 | 8.97E-31 | acyl carrier protein |
