# Supplementary material for: The conserved transcription factor PrlP modulates colonization and pathogenicity of Streptococcus suis in response to environmental stress
Source: PLoS Pathog. 2025 Jul 18;21(7):e1013314. doi: 10.1371/journal.ppat.1013314 (PMC12273997; doi:10.1371/journal.ppat.1013314)
Supplement: S5 Table — (DOCX) [file ppat.1013314.s006.docx]

**Table S5.** Expression levels of genes in Δ*prlP-*Δ*8740* compared to Δ*prlP*

| **Name** | **log2Fold**  **Change** | **pvalue** | **padj** | **Functional annotation** |
| --- | --- | --- | --- | --- |
| B9H01_07505 | 8.17 | 1.2E-118 | 1.7E-117 | sugar ABC transporter permease |
| B9H01_07510 | 7.69 | 9.85E-71 | 7.07E-70 | sugar ABC transporter permease |
| B9H01_00980 | 6.47 | 5E-208 | 1.8E-206 | sugar ABC transporter periplasmic protein |
| B9H01_08860 | 6.16 | 2.1E-305 | 1.2E-303 | lipoprotein |
| B9H01_01390 | 6.09 | 5.48E-24 | 1.61E-23 | PadR family transcriptional regulator |
| B9H01_03050 | 5.49 | 1.4E-212 | 5E-211 | transcriptional regulator |
| B9H01_01355 | 5.45 | 4.1E-157 | 8.4E-156 | hypothetical protein SSUSC84_0227 |
| B9H01_02295 | 5.40 | 1.7E-131 | 2.6E-130 | phosphotransferase system |
| B9H01_09715 | 5.29 | 5.76E-27 | 1.84E-26 | hypothetical protein SSUSC84_1831 |
| B9H01_00990 | 5.18 | 1.75E-89 | 1.58E-88 | sugar ABC transporter permease |
| B9H01_07250 | 5.14 | 3.8E-163 | 9E-162 | hypothetical protein SSU98_1514 |
| B9H01_05880 | 5.12 | 3.2E-222 | 1.2E-220 | phosphotransferase system |
| B9H01_02285 | 5.03 | 1.3E-278 | 6.3E-277 | phosphotransferase system |
| B9H01_01370 | 5.01 | 2.8E-188 | 8.5E-187 | transcriptional regulator |
| B9H01_05625 | 4.99 | 1.6E-196 | 5.4E-195 | glucuronate isomerase |
| B9H01_06160 | 4.93 | 2.66E-44 | 1.19E-43 | ferredoxin |
| B9H01_02280 | 4.85 | 2.4E-263 | 1.1E-261 | beta-galactosidase |
| B9H01_02050 | 4.82 | 4.6E-113 | 5.6E-112 | 16S rRNA methyltransferase GidB |
| B9H01_04985 | 4.81 | 2.1E-256 | 9.3E-255 | glycogen synthase |
| B9H01_09295 | 4.77 | 1.57E-77 | 1.24E-76 | transcriptional regulator |
| B9H01_03045 | 4.77 | 6.5E-224 | 2.6E-222 | NADPH-dependent FMN reductase |
| B9H01_02690 | 4.68 | 1.9E-257 | 8.8E-256 | haloacid dehalogenase-like hydrolase |
| B9H01_03345 | 4.65 | 1.7E-130 | 2.6E-129 | Co/Zn/Cd cation transporter |
| B9H01_00985 | 4.61 | 3.4E-118 | 4.5E-117 | binding-protein-dependent transport system membrane protein |
| B9H01_00975 | 4.57 | 3.1E-194 | 1E-192 | transcriptional regulator |
| B9H01_01870 | 4.55 | 6E-251 | 2.7E-249 | HAD superfamily hydrolase |
| B9H01_05450 | 4.45 | 1.5E-105 | 1.7E-104 | histone acetyltransferase HPA2-like acetyltransferase |
| B9H01_05065 | 4.44 | 8.86E-49 | 4.33E-48 | hypothetical protein SSU05_1034 |
| B9H01_02290 | 4.40 | 4.6E-154 | 9.3E-153 | phosphotransferase system |
| B9H01_05455 | 4.39 | 3.9E-293 | 2.1E-291 | hypothetical protein SSU05_1124 |
| B9H01_05300 | 4.37 | 1.6E-182 | 4.5E-181 | pantothenate kinase |
| B9H01_05200 | 4.34 | 4.08E-77 | 3.2E-76 | adenylate cyclase family protein |
| B9H01_04970 | 4.33 | 2.49E-90 | 2.31E-89 | LysR family transcriptional regulator |
| B9H01_00955 | 4.32 | 1.81E-92 | 1.73E-91 | hypothetical protein SSU05_0162 |
| B9H01_05490 | 4.28 | 1.19E-48 | 5.75E-48 | transcriptional regulator |
| B9H01_08415 | 4.24 | 3.36E-40 | 1.39E-39 | hemolysin-like protein |
| B9H01_05630 | 4.24 | 1.4E-109 | 1.6E-108 | 2-dehydro-3-deoxy-6-phosphogalactonate aldolase |
| B9H01_02060 | 4.24 | 1E-148 | 2E-147 | Holliday junction-specific endonuclease |
| B9H01_05620 | 4.23 | 3E-154 | 6.1E-153 | A Chain A |
| B9H01_04975 | 4.22 | 9.9E-289 | 5.3E-287 | ADP-glucose pyrophosphorylase |
| B9H01_02175 | 4.21 | 1.44E-15 | 3.45E-15 | amidophosphoribosyltransferase |
| B9H01_08410 | 4.21 | 8.34E-30 | 2.84E-29 | transcriptional regulator |
| B9H01_05960 | 4.15 | 2.46E-43 | 1.09E-42 | Cro/CI family transcriptional regulator |
| B9H01_06510 | 4.13 | 3.8E-166 | 9.4E-165 | cation transport ATPase |
| B9H01_05980 | 4.13 | 4.18E-33 | 1.53E-32 | hypothetical protein SSUSC84_1108 |
| B9H01_08380 | 4.12 | 3.9E-306 | 2.4E-304 | superfamily II DNA/RNA helicase |
| B9H01_02300 | 4.10 | 2.19E-73 | 1.63E-72 | phosphotransferase system |
| B9H01_01165 | 4.07 | 1.47E-68 | 1.03E-67 | phosphotransferase system cellobiose-specific component IIC |
| B9H01_05110 | 4.06 | 1.91E-25 | 5.89E-25 | ribose 5-phosphate isomerase RpiB |
| B9H01_05060 | 4.05 | 3.8E-110 | 4.6E-109 | hypothetical protein SSUSC84_0933 |
| B9H01_08925 | 4.04 | 4.1E-231 | 1.7E-229 | diadenosine tetraphosphate (Ap4A) hydrolase and other HIT family hydrolases |
| B9H01_05575 | 4.03 | 2.8E-105 | 3.1E-104 | hypothetical protein SSU05_1148 |
| B9H01_06035 | 4.02 | 1.5E-206 | 5E-205 | phospho-2-dehydro-3-deoxyheptonate aldolase |
| B9H01_05605 | 4.01 | 6.2E-225 | 2.5E-223 | hypothetical protein SSU05_1154 |
| B9H01_05335 | 4.01 | 1.8E-184 | 5.1E-183 | response regulator |
| B9H01_09955 | 3.97 | 2.27E-44 | 1.02E-43 | hypothetical protein SSU05_2072 |
| B9H01_06240 | 3.92 | 4.7E-162 | 1.1E-160 | phosphoglycerol transferase/alkaline phosphatase superfamily protein |
| B9H01_08155 | 3.91 | 3.6E-130 | 5.4E-129 | MF3-like protein |
| B9H01_01975 | 3.90 | 5E-172 | 1.3E-170 | glycogen phosphorylase |
| B9H01_01170 | 3.89 | 2.4E-118 | 3.2E-117 | hypothetical protein SSU05_0213 |
| B9H01_01845 | 3.85 | 1.6E-272 | 8E-271 | galactokinase |
| B9H01_03890 | 3.83 | 4.4E-138 | 7.3E-137 | carbamoyl phosphate synthase small subunit |
| B9H01_10535 | 3.83 | 7.2E-105 | 7.8E-104 | chromosome partitioning protein |
| B9H01_04180 | 3.82 | 9.5E-143 | 1.7E-141 | lytic murein transglycosylase |
| B9H01_06685 | 3.80 | 7.1E-199 | 2.4E-197 | amylase-binding protein B |
| B9H01_04030 | 3.80 | 3.1E-188 | 9.2E-187 | DeoR family regulatory protein |
| B9H01_01360 | 3.78 | 7.92E-96 | 8.03E-95 | hypothetical protein SSUSC84_0228 |
| B9H01_05340 | 3.76 | 1.3E-153 | 2.7E-152 | hypothetical protein SSU05_1097 |
| B9H01_01060 | 3.74 | 4.13E-23 | 1.19E-22 | phosphotransferase system |
| B9H01_05225 | 3.72 | 1.9E-115 | 2.4E-114 | redox-sensing transcriptional repressor Rex |
| B9H01_01970 | 3.72 | 9.6E-178 | 2.6E-176 | 4-alpha-glucanotransferase |
| B9H01_01175 | 3.72 | 1E-111 | 1.3E-110 | sugar ABC transporter periplasmic protein |
| B9H01_09415 | 3.71 | 7.4E-110 | 8.8E-109 | phosphotransferase mannnose-specific family component IIA |
| B9H01_06850 | 3.71 | 9.13E-42 | 3.91E-41 | hypothetical protein SSUSC84_1276 |
| B9H01_10265 | 3.71 | 9.92E-76 | 7.55E-75 | maltose/maltodextrin ABC transport system permease protein |
| B9H01_08435 | 3.69 | 1.3E-107 | 1.5E-106 | hypothetical protein SSU05_1750 |
| B9H01_06855 | 3.67 | 1.7E-48 | 8.22E-48 | hypothetical protein SSU05_1417 |
| B9H01_03135 | 3.66 | 3.6E-189 | 1.1E-187 | ornithine carbamoyltransferase |
| B9H01_01460 | 3.65 | 2.2E-109 | 2.6E-108 | alcohol dehydrogenase |
| B9H01_03150 | 3.64 | 1.6E-245 | 6.8E-244 | hypothetical protein SSU05_0629 |
| B9H01_05565 | 3.64 | 2.04E-61 | 1.24E-60 | hypothetical protein SSU05_1146 |
| B9H01_08370 | 3.63 | 2.9E-207 | 1E-205 | hypothetical protein SSU05_1737 |
| B9H01_05825 | 3.63 | 3.64E-52 | 1.9E-51 | NrdH-redoxin |
| B9H01_08430 | 3.62 | 7E-47 | 3.26E-46 | major facilitator superfamily permease |
| B9H01_05600 | 3.61 | 9E-185 | 2.6E-183 | beta-glucosidase-related glycosidase |
| B9H01_05890 | 3.59 | 1.28E-77 | 1.02E-76 | phosphotransferase system |
| B9H01_10225 | 3.57 | 8.4E-120 | 1.2E-118 | arginine repressor |
| B9H01_04165 | 3.56 | 2.07E-66 | 1.39E-65 | hypothetical protein SSU05_0851 |
| B9H01_05895 | 3.50 | 1.34E-71 | 9.7E-71 | unsaturated glucuronyl hydrolase |
| B9H01_01145 | 3.49 | 1.9E-160 | 4.3E-159 | fructose-1-phosphate kinase-like protein |
| B9H01_05505 | 3.46 | 6.7E-137 | 1.1E-135 | hypothetical protein SSUSC84_1017 |
| B9H01_08365 | 3.42 | 3.7E-182 | 1E-180 | hypothetical protein SSU05_1736 |
| B9H01_04890 | 3.39 | 5.55E-76 | 4.26E-75 | hypothetical protein SSU05_0993 |
| B9H01_05875 | 3.35 | 4.5E-25 | 1.38E-24 | preprotein translocase subunit YajC |
| B9H01_02955 | 3.35 | 1.61E-35 | 6.08E-35 | IS66 family element |
| B9H01_06010 | 3.35 | 2.3E-131 | 3.6E-130 | hypothetical protein SSU05_1242 |
| B9H01_09855 | 3.35 | 4.98E-46 | 2.28E-45 | hypothetical protein SSUSC84_1859 |
| B9H01_02645 | 3.35 | 2E-119 | 2.7E-118 | Lyzozyme M1 (1 |
| B9H01_08050 | 3.35 | 2.3E-137 | 3.8E-136 | pyruvate-formate lyase activating enzyme |
| B9H01_10070 | 3.31 | 1.4E-176 | 3.7E-175 | bifunctional 2&apos; |
| B9H01_05615 | 3.31 | 1.2E-119 | 1.6E-118 | D-mannonate oxidoreductase |
| B9H01_01610 | 3.29 | 5.1E-108 | 5.9E-107 | ferric uptake regulator family protein |
| B9H01_01645 | 3.28 | 2.29E-29 | 7.66E-29 | transcription regulation protein |
| B9H01_07525 | 3.28 | 2.23E-41 | 9.46E-41 | AraC family transcriptional regulator |
| B9H01_10250 | 3.26 | 2E-187 | 5.9E-186 | 4-alpha-glucanotransferase |
| B9H01_01965 | 3.25 | 3.3E-238 | 1.4E-236 | ATPases with chaperone activity |
| B9H01_03740 | 3.25 | 3.8E-74 | 2.85E-73 | glycerol-3-phosphate acyltransferase PlsY |
| B9H01_01150 | 3.25 | 6.2E-99 | 6.5E-98 | HhH-GPD superfamily base excision DNA repair protein |
| B9H01_04705 | 3.25 | 5.01E-70 | 3.56E-69 | hypothetical protein SSU05_0955 |
| B9H01_06045 | 3.24 | 2.1E-103 | 2.2E-102 | shikimate 5-dehydrogenase |
| B9H01_09965 | 3.23 | 4.69E-31 | 1.63E-30 | phosphotransferase system cellobiose-specific component IIB |
| B9H01_08125 | 3.23 | 6.5E-13 | 1.45E-12 | hypothetical protein SSUSC84_1521 |
| B9H01_05475 | 3.20 | 7.5E-160 | 1.7E-158 | peptidase T |
| B9H01_04895 | 3.19 | 9.57E-94 | 9.33E-93 | hypothetical protein SSU05_0994 |
| B9H01_04690 | 3.18 | 3.8E-31 | 1.33E-30 | recombinase |
| B9H01_02730 | 3.18 | 3.33E-97 | 3.45E-96 | transcriptional regulator |
| B9H01_08160 | 3.15 | 2E-125 | 2.8E-124 | phosphotyrosine protein phosphatase |
| B9H01_09810 | 3.15 | 1.3E-132 | 2.1E-131 | glucose-1-phosphate-uridylyltransferase |
| B9H01_03610 | 3.12 | 6E-105 | 6.6E-104 | NADH oxidase |
| B9H01_02835 | 3.11 | 8.61E-94 | 8.43E-93 | hypothetical protein SSUSC84_0499 |
| B9H01_04950 | 3.09 | 3.49E-36 | 1.34E-35 | hypothetical protein SSU05_1008 |
| B9H01_04095 | 3.09 | 3.7E-132 | 5.8E-131 | dihydrofolate reductase |
| B9H01_04945 | 3.07 | 4.05E-87 | 3.62E-86 | orotate phosphoribosyltransferase |
| B9H01_06335 | 3.07 | 1.7E-147 | 3.2E-146 | hypothetical protein SSU05_1311 |
| B9H01_07805 | 3.07 | 2.85E-59 | 1.64E-58 | hypothetical protein SSU05_1615 |
| B9H01_06375 | 3.06 | 5.7E-105 | 6.3E-104 | hypothetical protein SSU05_1320 |
| B9H01_08170 | 3.06 | 1.6E-163 | 3.9E-162 | acyltransferase family protein |
| B9H01_05470 | 3.05 | 2.8E-120 | 3.9E-119 | HAD superfamily hydrolase |
| B9H01_02305 | 3.04 | 4.98E-79 | 4.03E-78 | galactose mutarotase-like protein |
| B9H01_06980 | 3.04 | 5.03E-64 | 3.17E-63 | phosphopentomutase |
| B9H01_00995 | 3.04 | 2.55E-88 | 2.29E-87 | alpha-galactosidase |
| B9H01_04035 | 3.03 | 2.9E-189 | 9.2E-188 | fructose-1-phosphate kinase-like protein |
| B9H01_00010 | 3.03 | 1.18E-98 | 1.23E-97 | DNA polymerase III subunit beta |
| B9H01_09505 | 3.01 | 1.5E-126 | 2.1E-125 | hypothetical protein SSU05_1978 |
| B9H01_05955 | 3.01 | 1.84E-84 | 1.6E-83 | hypothetical protein SSU98_1246 |
| B9H01_05085 | 2.99 | 3.08E-14 | 7.06E-14 | phosphotransferase system cellobiose-specific component IIA |
| B9H01_04750 | 2.99 | 8.81E-87 | 7.8E-86 | hypothetical protein SSUSC84_0870 |
| B9H01_10030 | 2.98 | 8.5E-152 | 1.7E-150 | metal-dependent transcriptional regulator |
| B9H01_06975 | 2.97 | 7.42E-80 | 6.13E-79 | arsenate reductase |
| B9H01_01660 | 2.95 | 2.09E-27 | 6.76E-27 | dehydrogenase |
| B9H01_09970 | 2.94 | 5.06E-95 | 5.11E-94 | transcriptional antiterminator |
| B9H01_01380 | 2.94 | 1.14E-96 | 1.17E-95 | hypothetical protein SSU05_0262 |
| B9H01_04925 | 2.92 | 2.7E-138 | 4.5E-137 | 5&apos;-nucleotidase |
| B9H01_03700 | 2.91 | 5.45E-69 | 3.85E-68 | multidrug ABC transporter ATPase |
| B9H01_05740 | 2.90 | 5.59E-47 | 2.61E-46 | hypothetical protein SSU05_1185 |
| B9H01_01385 | 2.89 | 5E-94 | 4.93E-93 | hypothetical protein SSU05_0263 |
| B9H01_02795 | 2.88 | 4.38E-94 | 4.34E-93 | Zn-dependent hydrolase |
| B9H01_08845 | 2.88 | 1.3E-139 | 2.2E-138 | pyruvate/2-oxoglutarate dehydrogenase complex |
| B9H01_06120 | 2.87 | 1.06E-91 | 1E-90 | sulfatase |
| B9H01_03510 | 2.85 | 1.66E-63 | 1.04E-62 | sugar metabolism transcriptional regulator |
| B9H01_08755 | 2.85 | 3.12E-78 | 2.5E-77 | Beta-fructosidases (levanase/invertase) |
| B9H01_05725 | 2.85 | 1.63E-41 | 6.95E-41 | permease |
| B9H01_03600 | 2.84 | 2.2E-105 | 2.4E-104 | oligoendopeptidase F |
| B9H01_07435 | 2.84 | 6.74E-67 | 4.58E-66 | manganese-dependent superoxide dismutase |
| B9H01_04935 | 2.81 | 5.4E-126 | 7.7E-125 | uracil-DNA glycosylase |
| B9H01_05495 | 2.79 | 3.9E-107 | 4.4E-106 | hypothetical protein SSU05_1132 |
| B9H01_03545 | 2.79 | 4.23E-64 | 2.7E-63 | glycerol dehydrogenase |
| B9H01_07015 | 2.77 | 3.61E-65 | 2.35E-64 | hypothetical protein SSU05_1455 |
| B9H01_09280 | 2.77 | 5.55E-92 | 5.26E-91 | outer surface protein |
| B9H01_10530 | 2.75 | 4.7E-118 | 6.2E-117 | trypsin-like serine protease |
| B9H01_01560 | 2.74 | 3.1E-152 | 6.2E-151 | molecular chaperone DnaK |
| B9H01_09290 | 2.72 | 1.3E-106 | 1.5E-105 | glucokinase regulatory protein |
| B9H01_05530 | 2.72 | 2.75E-41 | 1.16E-40 | cation efflux family protein |
| B9H01_05520 | 2.72 | 1.49E-18 | 3.8E-18 | hypothetical protein SSU05_1137 |
| B9H01_02000 | 2.72 | 7.27E-15 | 1.7E-14 | transcriptional regulator |
| B9H01_05290 | 2.71 | 2.4E-141 | 4.1E-140 | pyrimidine-nucleoside phosphorylase |
| B9H01_10080 | 2.70 | 6.91E-91 | 6.49E-90 | sortase-like protein |
| B9H01_04015 | 2.68 | 7.55E-61 | 4.52E-60 | 16S rRNA-processing protein RimM |
| B9H01_10905 | 2.65 | 3.69E-15 | 8.72E-15 | hypothetical protein SSU05_1747 |
| B9H01_08175 | 2.64 | 2.18E-38 | 8.76E-38 | membrane-associated phospholipid phosphatase |
| B9H01_08775 | 2.64 | 3.44E-72 | 2.51E-71 | elongation factor P |
| B9H01_09225 | 2.63 | 4.17E-24 | 1.23E-23 | alpha-1 |
| B9H01_05775 | 2.63 | 5.65E-41 | 2.36E-40 | hypothetical protein SSU05_1193 |
| B9H01_01465 | 2.62 | 2.11E-79 | 1.72E-78 | bifunctional acetaldehyde-CoA/alcohol dehydrogenase |
| B9H01_05840 | 2.62 | 2.1E-105 | 2.3E-104 | ribonucleotide-diphosphate reductase subunit beta |
| B9H01_03440 | 2.62 | 3.33E-28 | 1.08E-27 | phosphopantothenate--cysteine ligase |
| B9H01_06965 | 2.61 | 8.24E-61 | 4.92E-60 | purine nucleoside phosphorylase |
| B9H01_08210 | 2.61 | 3.29E-56 | 1.83E-55 | segregation and condensation protein A |
| B9H01_05845 | 2.60 | 3.81E-77 | 3.01E-76 | transcriptional regulator |
| B9H01_09705 | 2.59 | 1.67E-37 | 6.61E-37 | argininosuccinate lyase |
| B9H01_08565 | 2.58 | 7.02E-42 | 3.01E-41 | HAD superfamily hydrolase |
| B9H01_07120 | 2.57 | 7.14E-60 | 4.19E-59 | DNA-binding membrane protein |
| B9H01_03540 | 2.55 | 5.17E-10 | 1.04E-09 | fructose-6-phosphate aldolase |
| B9H01_05295 | 2.54 | 3.16E-64 | 2.02E-63 | 16S RNA G1207 methylase RsmC |
| B9H01_07585 | 2.51 | 1.2E-15 | 2.87E-15 | transcriptional regulator |
| B9H01_06620 | 2.50 | 2.79E-81 | 2.33E-80 | catabolite control protein A |
| B9H01_09015 | 2.48 | 2.29E-51 | 1.18E-50 | ABC transporter ATPase |
| B9H01_06460 | 2.48 | 1.73E-50 | 8.72E-50 | extracellular solute-binding protein |
| B9H01_05075 | 2.48 | 3.94E-54 | 2.14E-53 | 6-phospho-beta-galactosidase |
| B9H01_03060 | 2.47 | 3.52E-76 | 2.71E-75 | hypothetical protein SSU98_0612 |
| B9H01_05210 | 2.46 | 1.04E-68 | 7.3E-68 | cysteine sulfinate desulfinase/cysteine desulfurase |
| B9H01_04720 | 2.46 | 4.2E-35 | 1.59E-34 | adenine phosphoribosyltransferase |
| B9H01_00750 | 2.46 | 2.85E-67 | 1.95E-66 | transcriptional regulator |
| B9H01_03120 | 2.45 | 8.98E-49 | 4.38E-48 | cAMP-binding protein - catabolite gene activator and regulatory subunit of cAMP-dependent protein kinases |
| B9H01_01980 | 2.45 | 1.8E-58 | 1.02E-57 | transcriptional regulator |
| B9H01_04155 | 2.44 | 5.32E-66 | 3.51E-65 | methylase of polypeptide chain release factors |
| B9H01_04430 | 2.41 | 0.000144 | 0.00022 | hypothetical protein SSU05_0904 |
| B9H01_01885 | 2.40 | 2.19E-72 | 1.6E-71 | RNA-binding protein |
| B9H01_03550 | 2.40 | 5.29E-70 | 3.74E-69 | glycerol dehydrogenase |
| B9H01_03160 | 2.39 | 5.84E-60 | 3.44E-59 | arginine repressor |
| B9H01_08850 | 2.38 | 4.33E-64 | 2.76E-63 | pyruvate dehydrogenase E1 component |
| B9H01_07100 | 2.37 | 1.31E-76 | 1.02E-75 | UDP-N-acetylmuramyl tripeptide synthase |
| B9H01_09950 | 2.36 | 3.13E-23 | 9.05E-23 | PTS system transporter subunit IIC |
| B9H01_04040 | 2.36 | 9.3E-111 | 1.1E-109 | phosphotransferase system |
| B9H01_07540 | 2.35 | 1.24E-12 | 2.73E-12 | hypothetical protein SSU98_1572 |
| B9H01_08220 | 2.35 | 1.32E-57 | 7.46E-57 | hypothetical protein SSU05_1703 |
| B9H01_08890 | 2.35 | 1.32E-63 | 8.3E-63 | transcription elongation factor NusA |
| B9H01_05940 | 2.35 | 1.8E-31 | 6.34E-31 | hypothetical protein SSU98_1243 |
| B9H01_06680 | 2.33 | 2.5E-115 | 3.2E-114 | copper-transporting ATPase |
| B9H01_04290 | 2.33 | 4.32E-13 | 9.62E-13 | DNA-binding protein |
| B9H01_00270 | 2.33 | 3.91E-36 | 1.5E-35 | phosphoribosylaminoimidazole carboxylase ATPase subunit |
| B9H01_01600 | 2.32 | 5.02E-24 | 1.48E-23 | hypothetical protein SSU98_0304 |
| B9H01_09420 | 2.31 | 2.23E-46 | 1.03E-45 | glycerol uptake facilitator and related permease (major Intrinsic protein family) |
| B9H01_07740 | 2.31 | 1.98E-62 | 1.22E-61 | Signal recognition particle GTPase |
| B9H01_07160 | 2.30 | 1.57E-53 | 8.45E-53 | hypothetical protein SSU05_1481 |
| B9H01_01555 | 2.30 | 4.48E-77 | 3.5E-76 | heat shock protein GrpE |
| B9H01_06800 | 2.29 | 2.18E-37 | 8.57E-37 | ferrous iron transport protein A |
| B9H01_06755 | 2.29 | 8.61E-37 | 3.35E-36 | transcriptional regulator |
| B9H01_07565 | 2.29 | 3.08E-30 | 1.07E-29 | hypothetical protein SSUSC84_1412 |
| B9H01_04620 | 2.28 | 2.55E-25 | 7.83E-25 | transcriptional regulator |
| B9H01_08250 | 2.27 | 3.18E-50 | 1.59E-49 | cysteine aminopeptidase C |
| B9H01_04340 | 2.27 | 1.97E-51 | 1.02E-50 | response regulator |
| B9H01_06925 | 2.27 | 1.38E-57 | 7.78E-57 | 30S ribosomal protein S21 |
| B9H01_00205 | 2.26 | 5.2E-55 | 2.85E-54 | ribose-phosphate pyrophosphokinase |
| B9H01_09520 | 2.26 | 8.99E-25 | 2.71E-24 | hypothetical protein SSU05_1981 |
| B9H01_01650 | 2.26 | 5.16E-33 | 1.89E-32 | NADPH:quinone reductase and related Zn-dependent oxidoreductase |
| B9H01_06325 | 2.25 | 2.81E-82 | 2.38E-81 | single-stranded DNA-specific exonuclease |
| B9H01_03480 | 2.25 | 4.18E-17 | 1.04E-16 | hypothetical protein SSU05_0699 |
| B9H01_10470 | 2.25 | 3.37E-28 | 1.1E-27 | S4 domain-containing protein YaaA |
| B9H01_05525 | 2.25 | 1.9E-13 | 4.26E-13 | hypothetical protein SSUSC84_1021 |
| B9H01_04425 | 2.25 | 7.61E-27 | 2.41E-26 | phage integrase family site specific recombinase |
| B9H01_08150 | 2.24 | 2.53E-51 | 1.3E-50 | Dpr |
| B9H01_05090 | 2.23 | 4.71E-16 | 1.14E-15 | transcriptional antiterminator |
| B9H01_01185 | 2.23 | 2.27E-19 | 5.95E-19 | ABC transporter |
| B9H01_03080 | 2.23 | 1.23E-40 | 5.12E-40 | hypothetical protein SSU98_0615 |
| B9H01_06130 | 2.22 | 1.25E-68 | 8.74E-68 | histidine triad protein |
| B9H01_03835 | 2.22 | 4.91E-56 | 2.72E-55 | branched-chain amino acid transport system carrier protein |
| B9H01_02275 | 2.22 | 2.72E-52 | 1.43E-51 | transcriptional regulator |
| B9H01_02485 | 2.21 | 5.31E-81 | 4.43E-80 | ATPases with chaperone activity |
| B9H01_05850 | 2.20 | 8.54E-21 | 2.3E-20 | heparinase II/III-like protein |
| B9H01_00005 | 2.20 | 2.37E-47 | 1.12E-46 | chromosomal replication initiation protein |
| B9H01_02140 | 2.18 | 1.33E-50 | 6.73E-50 | Signal transduction histidine kinase |
| B9H01_09650 | 2.18 | 4.59E-17 | 1.14E-16 | antitoxin of toxin-antitoxin stability system |
| B9H01_09285 | 2.17 | 6.42E-51 | 3.29E-50 | sugar phosphotransferase system (PTS) |
| B9H01_08320 | 2.16 | 3.55E-65 | 2.32E-64 | GTP-binding protein EngA |
| B9H01_02720 | 2.16 | 3.21E-47 | 1.51E-46 | hypothetical protein SSUST1_1276 |
| B9H01_01160 | 2.16 | 1.56E-28 | 5.13E-28 | repressor protein |
| B9H01_06400 | 2.16 | 1.15E-32 | 4.16E-32 | hypothetical protein SSUSC84_1191 |
| B9H01_04190 | 2.15 | 2.48E-38 | 9.95E-38 | translation initiation factor 1 |
| B9H01_04605 | 2.13 | 1.78E-58 | 1.02E-57 | DNA helicase |
| B9H01_02130 | 2.12 | 2.43E-71 | 1.75E-70 | serine/threonine protein kinase |
| B9H01_03665 | 2.11 | 5.11E-09 | 9.83E-09 | cobalt ABC transporter ATP-binding protein |
| B9H01_02755 | 2.10 | 2.47E-70 | 1.76E-69 | FAD dependent oxidoreductase |
| B9H01_01190 | 2.09 | 2.9E-27 | 9.28E-27 | nitrate/sulfonate/bicarbonate ABC transporter ATPase |
| B9H01_04700 | 2.09 | 4.59E-05 | 7.25E-05 | hypothetical protein SSU05_0954 |
| B9H01_03085 | 2.09 | 1.96E-60 | 1.17E-59 | hypothetical protein SSU98_0616 |
| B9H01_06015 | 2.08 | 3.88E-58 | 2.2E-57 | oligoendopeptidase F |
| B9H01_02135 | 2.07 | 6.23E-51 | 3.2E-50 | hypothetical protein SSUJS14_0397 |
| B9H01_10500 | 2.06 | 2.16E-47 | 1.02E-46 | hypothetical protein SSU05_2188 |
| B9H01_04610 | 2.06 | 7.83E-60 | 4.57E-59 | hypothetical protein SSU05_0935 |
| B9H01_07855 | 2.04 | 3.68E-41 | 1.55E-40 | biotin--protein ligase |
| B9H01_01245 | 2.03 | 8.1E-45 | 3.65E-44 | glycosidase |
| B9H01_05950 | 2.02 | 1.02E-22 | 2.91E-22 | hypothetical protein SSU98_1245 |
| B9H01_03470 | 2.02 | 6.34E-50 | 3.17E-49 | HsdS |
| B9H01_02125 | 2.02 | 3.21E-63 | 2E-62 | serine/threonine protein phosphatase |
| B9H01_01890 | 2.01 | 1.3E-49 | 6.48E-49 | nicotinic acid mononucleotide adenylyltransferase |
| B9H01_01225 | 2.01 | 1.11E-55 | 6.11E-55 | Signal peptidase I |
| B9H01_07180 | -2.01 | 4.74E-09 | 9.14E-09 | ABC transporter periplasmic protein |
| B9H01_07660 | -2.01 | 3.2E-52 | 1.68E-51 | 3-ketoacyl-ACP reductase |
| B9H01_04560 | -2.01 | 7.26E-06 | 0.000012 | hypothetical protein SPCG_0166 |
| B9H01_00475 | -2.02 | 3.45E-47 | 1.62E-46 | 30S ribosomal protein S3 |
| B9H01_05665 | -2.02 | 1.57E-63 | 9.85E-63 | UDP-N-acetylglucosamine 1-carboxyvinyltransferase |
| B9H01_09320 | -2.03 | 4.55E-31 | 1.58E-30 | 16S rRNA uridine-516 pseudouridylate synthase family protein |
| B9H01_01415 | -2.03 | 8.06E-55 | 4.38E-54 | hypothetical protein SSUSC84_0239 |
| B9H01_09425 | -2.03 | 8.29E-65 | 5.36E-64 | prolyl-tRNA synthetase |
| B9H01_00490 | -2.04 | 3.51E-59 | 2.01E-58 | 30S ribosomal protein S17 |
| B9H01_06345 | -2.04 | 1.62E-38 | 6.53E-38 | ribonuclease Z |
| B9H01_02310 | -2.04 | 4.22E-15 | 9.94E-15 | hypothetical protein |
| B9H01_08480 | -2.05 | 3.03E-40 | 1.26E-39 | Trk family potassium uptake protein |
| B9H01_06780 | -2.05 | 6.69E-51 | 3.42E-50 | ABC transporter |
| B9H01_08100 | -2.06 | 5.26E-48 | 2.52E-47 | secreted protein containing a PDZ domain protein |
| B9H01_00325 | -2.07 | 4.35E-62 | 2.66E-61 | Holliday junction DNA helicase RuvB |
| B9H01_09690 | -2.07 | 1.75E-50 | 8.83E-50 | RNA-binding protein |
| B9H01_00485 | -2.08 | 1.57E-35 | 5.94E-35 | 50S ribosomal protein L29 |
| B9H01_01590 | -2.09 | 4.46E-29 | 1.47E-28 | amino acid ABC transporter permease |
| B9H01_04350 | -2.09 | 5.23E-21 | 1.44E-20 | hypothetical protein SSUSC84_0792 |
| B9H01_02360 | -2.09 | 2.07E-28 | 6.79E-28 | hypothetical protein SSU05_0466 |
| B9H01_09110 | -2.09 | 2E-63 | 1.25E-62 | 30S ribosomal protein S9 |
| B9H01_02085 | -2.10 | 1.66E-65 | 1.09E-64 | hypothetical protein SSUA7_0379 |
| B9H01_00960 | -2.11 | 4.19E-46 | 1.92E-45 | molecular chaperone |
| B9H01_00785 | -2.12 | 3.01E-60 | 1.78E-59 | DNA-directed RNA polymerase subunit beta&apos; |
| B9H01_09070 | -2.12 | 1.33E-55 | 7.33E-55 | acetolactate synthase catalytic subunit |
| B9H01_04115 | -2.12 | 4.43E-47 | 2.08E-46 | nucleoside diphosphate kinase |
| B9H01_08180 | -2.13 | 3.67E-21 | 1.01E-20 | hypothetical protein SSUBM407_1580 |
| B9H01_04050 | -2.14 | 4.1E-61 | 2.48E-60 | hypothetical protein SSU05_0827 |
| B9H01_06495 | -2.14 | 6.14E-34 | 2.28E-33 | peptidyl-prolyl cis-trans isomerase |
| B9H01_05710 | -2.14 | 2.08E-29 | 6.98E-29 | F0F1 ATP synthase subunit C |
| B9H01_00560 | -2.14 | 6.99E-21 | 1.9E-20 | adenylate kinase |
| B9H01_00710 | -2.14 | 0.003641 | 0.005067 | hypothetical protein SSU05_0106 |
| B9H01_02605 | -2.15 | 6E-48 | 2.86E-47 | peptide ABC transporter permease |
| B9H01_07195 | -2.15 | 4.28E-05 | 6.79E-05 | transcription antiterminator |
| B9H01_01475 | -2.15 | 1.39E-57 | 7.79E-57 | ABC transporter ATPase/permease |
| B9H01_06150 | -2.15 | 8.48E-75 | 6.39E-74 | cytidylate kinase |
| B9H01_02500 | -2.15 | 2.05E-26 | 6.43E-26 | amino acid ABC transporter ATP-binding protein |
| B9H01_07310 | -2.15 | 0.000171 | 0.000259 | virulence-associated protein E |
| B9H01_03920 | -2.16 | 4.33E-17 | 1.08E-16 | RNA-binding protein |
| B9H01_07420 | -2.16 | 1.59E-23 | 4.63E-23 | hypothetical protein SSU05_1537 |
| B9H01_07590 | -2.16 | 4.33E-75 | 3.27E-74 | superfamily II DNA/RNA helicase |
| B9H01_04365 | -2.17 | 9.54E-12 | 2.06E-11 | bacteroiocin operon protein |
| B9H01_08505 | -2.17 | 1.68E-70 | 1.2E-69 | glycyl-tRNA synthetase subunit beta |
| B9H01_01630 | -2.17 | 1.79E-36 | 6.91E-36 | hypothetical protein SSU05_0314 |
| B9H01_01725 | -2.17 | 1.08E-90 | 1.01E-89 | fructose-bisphosphate aldolase |
| B9H01_04555 | -2.17 | 0.000119 | 0.000182 | conjugative transposon membrane protein |
| B9H01_09895 | -2.18 | 0.007821 | 0.010614 | L-xylulose 5-phosphate 3-epimerase |
| B9H01_09090 | -2.18 | 2.25E-52 | 1.19E-51 | dihydroxy-acid dehydratase |
| B9H01_06545 | -2.19 | 9.93E-08 | 1.79E-07 | ABC transporter ATPase |
| B9H01_01540 | -2.19 | 2.5E-27 | 8.03E-27 | hypothetical protein SSU05_0296 |
| B9H01_05245 | -2.19 | 3.46E-90 | 3.19E-89 | DNA gyrase subunit A |
| B9H01_07075 | -2.20 | 1.06E-36 | 4.11E-36 | methyltransferase |
| B9H01_07665 | -2.20 | 1.65E-22 | 4.68E-22 | hypothetical protein SSU05_1588 |
| B9H01_03930 | -2.21 | 7.49E-55 | 4.1E-54 | multidrug ABC transporter ATPase and permease |
| B9H01_08460 | -2.23 | 1.27E-47 | 6.03E-47 | polyprenyl synthetase |
| B9H01_05555 | -2.23 | 5.36E-47 | 2.5E-46 | GTP cyclohydrolase I |
| B9H01_09445 | -2.24 | 6.81E-33 | 2.48E-32 | preprotein translocase subunit |
| B9H01_08690 | -2.24 | 9.11E-60 | 5.3E-59 | 3-oxoacyl-ACP synthase |
| B9H01_03455 | -2.27 | 1.05E-43 | 4.64E-43 | pyridine nucleotide-disulfide oxidoreductase |
| B9H01_04805 | -2.27 | 3.07E-06 | 5.21E-06 | Type IV secretory pathway |
| B9H01_03950 | -2.28 | 3.14E-26 | 9.79E-26 | UDP-N-acetylenolpyruvoylglucosamine reductase |
| B9H01_07925 | -2.28 | 4.74E-48 | 2.29E-47 | 3-hydroxy-3-methylglutaryl CoA synthase |
| B9H01_04785 | -2.30 | 4.63E-07 | 8.08E-07 | hypothetical protein SSUSC84_0877 |
| B9H01_09845 | -2.31 | 1.59E-31 | 5.61E-31 | serine/threonine protein phosphatase |
| B9H01_07835 | -2.31 | 1.77E-79 | 1.45E-78 | S-adenosylmethionine synthetase |
| B9H01_01435 | -2.33 | 1.33E-38 | 5.39E-38 | methyl-accepting chemotaxis protein |
| B9H01_06020 | -2.34 | 6.45E-05 | 0.000101 | competence protein |
| B9H01_08665 | -2.35 | 7.81E-55 | 4.26E-54 | 3-oxoacyl-ACP synthase |
| B9H01_01605 | -2.35 | 3.73E-79 | 3.03E-78 | Heavy metal-(Cd/Co/Hg/Pb/Zn)-translocating P-type ATPase |
| B9H01_03500 | -2.35 | 2.56E-10 | 5.22E-10 | hypothetical protein SSU05_0704 |
| B9H01_10485 | -2.36 | 3.72E-27 | 1.19E-26 | tryptophanyl-tRNA synthetase II |
| B9H01_05125 | -2.36 | 0.005295 | 0.007283 | hypothetical protein SSU05_1046 |
| B9H01_09365 | -2.36 | 1.66E-21 | 4.6E-21 | 2-isopropylmalate synthase |
| B9H01_01535 | -2.38 | 3.04E-08 | 5.64E-08 | hypothetical protein SSU05_0295 |
| B9H01_06670 | -2.39 | 8.9E-91 | 8.32E-90 | hypothetical protein SSUSC84_1245 |
| B9H01_02505 | -2.39 | 1.09E-53 | 5.88E-53 | bifunctional 5 |
| B9H01_03925 | -2.39 | 4.91E-57 | 2.75E-56 | ABC transporter ATP-binding protein |
| B9H01_00855 | -2.39 | 6.49E-83 | 5.53E-82 | cellulase M-like protein |
| B9H01_09395 | -2.39 | 1.07E-67 | 7.37E-67 | DhaKLM operon coactivator DhaQ |
| B9H01_00355 | -2.41 | 4.65E-73 | 3.43E-72 | transposase |
| B9H01_00695 | -2.42 | 0.025807 | 0.033066 | hypothetical protein SSU05_0103 |
| B9H01_08965 | -2.44 | 1.52E-57 | 8.52E-57 | peptide ABC transporter permease |
| B9H01_04065 | -2.44 | 2.89E-78 | 2.32E-77 | ABC transporter ATPase |
| B9H01_09540 | -2.45 | 0.00099 | 0.001435 | preprotein translocase subunit SecE |
| B9H01_02350 | -2.45 | 1.07E-50 | 5.45E-50 | hypothetical protein SSU05_0464 |
| B9H01_08280 | -2.45 | 0.000307 | 0.000459 | transcription elongation factor GreA |
| B9H01_04640 | -2.45 | 8.5E-07 | 1.47E-06 | DNA primase (type) |
| B9H01_10135 | -2.46 | 6.57E-62 | 4E-61 | ribosomal protein L11 methyltransferase |
| B9H01_00910 | -2.47 | 2.77E-50 | 1.39E-49 | 30S ribosomal protein S7 |
| B9H01_08635 | -2.47 | 3.65E-42 | 1.58E-41 | histone acetyltransferase HPA2-like acetyltransferase |
| B9H01_07630 | -2.47 | 1.28E-46 | 5.91E-46 | 5&apos;-methylthioadenosine/S-adenosylhomocysteine nucleosidase |
| B9H01_04780 | -2.47 | 9.77E-12 | 2.1E-11 | Tn5252 |
| B9H01_00030 | -2.49 | 2.68E-76 | 2.08E-75 | GTP-binding protein |
| B9H01_07240 | -2.50 | 1.32E-14 | 3.06E-14 | hypothetical protein SSU05_1501 |
| B9H01_04840 | -2.51 | 0.000144 | 0.00022 | hypothetical protein SSU05_0981 |
| B9H01_07825 | -2.51 | 4.76E-48 | 2.29E-47 | UDP-N-acetylglucosamine 1-carboxyvinyltransferase |
| B9H01_02435 | -2.51 | 1.62E-79 | 1.33E-78 | cell division protein FtsZ |
| B9H01_09915 | -2.51 | 6.43E-06 | 1.06E-05 | PTS system ascorbate-specific transporter subunit IIC |
| B9H01_00460 | -2.52 | 6.89E-85 | 6.07E-84 | 50S ribosomal protein L2 |
| B9H01_03270 | -2.53 | 0.00015 | 0.000228 | DNA uptake protein and related DNA-binding proteins |
| B9H01_07190 | -2.53 | 1.2E-20 | 3.22E-20 | phosphotransferase system IIC component |
| B9H01_08010 | -2.53 | 2.2E-07 | 3.92E-07 | hypothetical protein SSU05_1659 |
| B9H01_10310 | -2.53 | 6.6E-24 | 1.93E-23 | glycosyl transferase |
| B9H01_09905 | -2.54 | 0.011947 | 0.01582 | phosphotransferase system mannitol/fructose-specific IIA domain-containing protein |
| B9H01_09745 | -2.56 | 2.32E-94 | 2.31E-93 | glutamyl-tRNA synthetase |
| B9H01_01020 | -2.57 | 2.2E-110 | 2.7E-109 | Epf-like protein |
| B9H01_07470 | -2.57 | 1.21E-37 | 4.79E-37 | branched chain amino acid ABC transporter permease |
| B9H01_09375 | -2.58 | 8.85E-68 | 6.14E-67 | leucyl aminopeptidase (aminopeptidase T) |
| B9H01_08490 | -2.58 | 1E-65 | 6.6E-65 | DinF |
| B9H01_04375 | -2.58 | 2.26E-28 | 7.4E-28 | multidrug ABC transporter ATPase |
| B9H01_05680 | -2.59 | 4E-104 | 4.3E-103 | F0F1 ATP synthase subunit beta |
| B9H01_03255 | -2.59 | 1.28E-68 | 8.96E-68 | cobalamin/Fe3+-siderophores ABC transporter ATPase |
| B9H01_01470 | -2.59 | 1.4E-115 | 1.8E-114 | Threonine synthase |
| B9H01_00540 | -2.60 | 2.72E-39 | 1.11E-38 | 30S ribosomal protein S5 |
| B9H01_08530 | -2.60 | 1.3E-24 | 3.89E-24 | metal ABC transporter ATPase |
| B9H01_07460 | -2.61 | 1.43E-42 | 6.23E-42 | branched-chain amino acid ABC transporter ATPase |
| B9H01_01620 | -2.61 | 7.56E-67 | 5.12E-66 | phosphomethylpyrimidine kinase |
| B9H01_09195 | -2.62 | 2.04E-05 | 3.29E-05 | sugar ABC transporter permease |
| B9H01_07115 | -2.63 | 6.79E-61 | 4.08E-60 | SAM-dependent methyltransferase |
| B9H01_00190 | -2.63 | 4.92E-22 | 1.37E-21 | rod shape-determining protein MreC |
| B9H01_10370 | -2.64 | 1.07E-59 | 6.2E-59 | replicative DNA helicase |
| B9H01_05675 | -2.64 | 1.75E-94 | 1.75E-93 | F0F1 ATP synthase subunit epsilon |
| B9H01_06985 | -2.64 | 9.09E-72 | 6.6E-71 | ribose-5-phosphate isomerase A |
| B9H01_10375 | -2.64 | 1.17E-33 | 4.34E-33 | 50S ribosomal protein L9 |
| B9H01_10055 | -2.64 | 5.65E-25 | 1.72E-24 | hypothetical protein SSU05_2092 |
| B9H01_01340 | -2.65 | 1.15E-66 | 7.76E-66 | dihydroorotate dehydrogenase 1A |
| B9H01_05395 | -2.65 | 8.64E-51 | 4.4E-50 | Spx family transcriptional regulator |
| B9H01_02095 | -2.66 | 6.75E-97 | 6.95E-96 | hypothetical protein SSU05_0421 |
| B9H01_02560 | -2.66 | 8.17E-62 | 4.97E-61 | collagenase-like protease |
| B9H01_06060 | -2.67 | 1.1E-22 | 3.14E-22 | ABC transporter ATPase |
| B9H01_02165 | -2.67 | 6.71E-48 | 3.19E-47 | hypothetical protein SSU05_0436 |
| B9H01_02705 | -2.67 | 6.97E-36 | 2.65E-35 | hypothetical protein SSU05_0535 |
| B9H01_08675 | -2.67 | 9.2E-136 | 1.5E-134 | malonyl CoA-acyl carrier protein transacylase |
| B9H01_08515 | -2.67 | 9.49E-32 | 3.37E-31 | Phage envelope protein |
| B9H01_01860 | -2.68 | 3.18E-40 | 1.32E-39 | CutC family protein |
| B9H01_10320 | -2.68 | 0.00097 | 0.001408 | hypothetical protein SSU05_2146 |
| B9H01_07050 | -2.69 | 1.4E-132 | 2.2E-131 | dipeptidase PepV |
| B9H01_07275 | -2.70 | 1.62E-29 | 5.47E-29 | haloacid dehalogenase-like hydrolase |
| B9H01_10440 | -2.70 | 2.85E-34 | 1.07E-33 | cobalt transporter ATP-binding subunit |
| B9H01_00875 | -2.71 | 1.5E-107 | 1.7E-106 | EMAP domain-containing protein |
| B9H01_00970 | -2.73 | 7.13E-85 | 6.26E-84 | DNA-binding/iron metalloprotein/AP endonuclease |
| B9H01_01335 | -2.74 | 2.85E-66 | 1.9E-65 | glutamate dehydrogenase |
| B9H01_00935 | -2.74 | 1.58E-16 | 3.86E-16 | hypothetical protein SSU05_0158 |
| B9H01_05550 | -2.74 | 9.3E-111 | 1.1E-109 | dihydropteroate synthase |
| B9H01_07575 | -2.75 | 7.38E-32 | 2.64E-31 | histone acetyltransferase HPA2-like acetyltransferase |
| B9H01_04905 | -2.75 | 5.98E-63 | 3.7E-62 | ribonuclease HII |
| B9H01_04590 | -2.76 | 0.000105 | 0.000161 | DNA translocase FtsK |
| B9H01_08780 | -2.78 | 3E-117 | 3.9E-116 | metallopeptidase |
| B9H01_09785 | -2.78 | 4.55E-54 | 2.46E-53 | deoxyuridine 5&apos;-triphosphate nucleotidohydrolase |
| B9H01_02025 | -2.78 | 5.99E-60 | 3.53E-59 | Rhodanese domain protein |
| B9H01_06295 | -2.79 | 5.71E-29 | 1.88E-28 | hypothetical protein SSUSC84_1169 |
| B9H01_01855 | -2.80 | 1.68E-19 | 4.43E-19 | hypothetical protein SSUSC84_0318 |
| B9H01_09240 | -2.82 | 3.1E-104 | 3.4E-103 | cysteinyl-tRNA synthetase |
| B9H01_00580 | -2.83 | 3.54E-66 | 2.34E-65 | 30S ribosomal protein S11 |
| B9H01_05165 | -2.83 | 4.83E-22 | 1.35E-21 | hypothetical protein SSU05_1053 |
| B9H01_03830 | -2.84 | 2.39E-67 | 1.64E-66 | thiamine biosynthesis protein ThiI |
| B9H01_09940 | -2.84 | 4.3E-101 | 4.6E-100 | amino acid ABC transporter periplasmic protein |
| B9H01_10130 | -2.84 | 1.52E-62 | 9.36E-62 | 16S ribosomal RNA methyltransferase RsmE |
| B9H01_09255 | -2.85 | 1.73E-41 | 7.37E-41 | serine acetyltransferase |
| B9H01_07875 | -2.86 | 1.07E-15 | 2.59E-15 | hypothetical protein SSU05_1629 |
| B9H01_09725 | -2.86 | 2.65E-53 | 1.42E-52 | glyoxalase/bleomycin resistance protein/dioxygenase superfamily protein |
| B9H01_10390 | -2.87 | 2.75E-29 | 9.16E-29 | MutT/NUDIX hydrolase family protein |
| B9H01_08660 | -2.88 | 4.56E-90 | 4.18E-89 | acetyl-CoA carboxylase biotin carboxyl carrier protein subunit |
| B9H01_09260 | -2.88 | 5.8E-134 | 9.2E-133 | polynucleotide phosphorylase/polyadenylase |
| B9H01_00805 | -2.88 | 9.39E-06 | 1.54E-05 | Type II secretory pathway |
| B9H01_01400 | -2.89 | 2.11E-40 | 8.79E-40 | effector of murein hydrolase |
| B9H01_07815 | -2.90 | 5.11E-82 | 4.32E-81 | methionine aminopeptidase |
| B9H01_07205 | -2.90 | 4.53E-96 | 4.62E-95 | alpha-acetolactate decarboxylase |
| B9H01_10415 | -2.93 | 1.8E-114 | 2.3E-113 | tRNA-specific 2-thiouridylase MnmA |
| B9H01_03335 | -2.93 | 3.68E-49 | 1.81E-48 | methylated DNA-protein cysteine methyltransferase |
| B9H01_03575 | -2.95 | 5.65E-06 | 9.42E-06 | glycerol kinase |
| B9H01_01445 | -2.97 | 9.43E-68 | 6.52E-67 | 50S ribosomal protein L33 |
| B9H01_05430 | -2.99 | 8.64E-14 | 1.96E-13 | hypothetical protein SSU05_1118 |
| B9H01_07080 | -2.99 | 1.5E-113 | 1.9E-112 | hypothetical protein SSU05_1467 |
| B9H01_01455 | -3.00 | 7.85E-87 | 6.98E-86 | histidyl-tRNA synthetase |
| B9H01_00225 | -3.00 | 9.17E-13 | 2.03E-12 | acyl carrier protein |
| B9H01_07950 | -3.01 | 9.86E-05 | 0.000152 | transposase |
| B9H01_04380 | -3.02 | 1.26E-11 | 2.7E-11 | hypothetical protein SSU05_0895 |
| B9H01_09635 | -3.02 | 2E-145 | 3.6E-144 | dimethyladenosine transferase |
| B9H01_09450 | -3.02 | 3E-129 | 4.5E-128 | adenylosuccinate synthase |
| B9H01_04360 | -3.03 | 2.13E-66 | 1.42E-65 | peptide ABC transporter ATPase |
| B9H01_02545 | -3.04 | 4.99E-17 | 1.24E-16 | mercuric resisitant regulatory protein |
| B9H01_07930 | -3.04 | 4.2E-99 | 4.46E-98 | hydroxymethylglutaryl-CoA reductase |
| B9H01_04055 | -3.04 | 5.1E-121 | 7E-120 | dihydrodipicolinate reductase |
| B9H01_01440 | -3.04 | 3.5E-161 | 7.9E-160 | methyl-accepting chemotaxis protein |
| B9H01_02365 | -3.06 | 2.4E-145 | 4.3E-144 | asparagine synthetase AsnA |
| B9H01_07340 | -3.07 | 0.00028 | 0.00042 | hypothetical protein SSUSC84_1368 |
| B9H01_02375 | -3.09 | 2.53E-53 | 1.36E-52 | hypothetical protein SSU05_0469 |
| B9H01_06025 | -3.11 | 6.3E-158 | 1.4E-156 | methionyl-tRNA synthetase |
| B9H01_00575 | -3.12 | 1.36E-93 | 1.32E-92 | 30S ribosomal protein S13 |
| B9H01_01430 | -3.16 | 1.2E-157 | 2.6E-156 | surface-anchored protein |
| B9H01_08650 | -3.16 | 2.52E-92 | 2.4E-91 | acetyl-CoA carboxylase biotin carboxylase subunit |
| B9H01_04910 | -3.17 | 2.44E-81 | 2.05E-80 | ribosomal biogenesis GTPase |
| B9H01_03825 | -3.18 | 1.27E-77 | 1.01E-76 | cysteine sulfinate desulfinase/cysteine desulfurase |
| B9H01_00940 | -3.18 | 3.79E-80 | 3.14E-79 | transcriptional regulator |
| B9H01_10295 | -3.18 | 2.1E-139 | 3.5E-138 | aspartyl-tRNA synthetase |
| B9H01_08110 | -3.19 | 1.5E-88 | 1.35E-87 | hypothetical protein SSU05_1681 |
| B9H01_10115 | -3.19 | 8.53E-77 | 6.64E-76 | hypothetical protein SSU05_2104 |
| B9H01_07335 | -3.20 | 0.00228 | 0.003222 | hypothetical protein SSU05_1519 |
| B9H01_03170 | -3.20 | 1.42E-42 | 6.22E-42 | 6-phosphogluconolactonase/glucosamine-6-phosphate isomerase/deaminase |
| B9H01_08685 | -3.22 | 8.3E-136 | 1.3E-134 | acyl carrier protein |
| B9H01_06135 | -3.23 | 1.3E-92 | 1.25E-91 | 50S ribosomal protein L20 |
| B9H01_10385 | -3.23 | 3.5E-128 | 5.2E-127 | tRNA uridine 5-carboxymethylaminomethyl modification enzyme GidA |
| B9H01_08535 | -3.26 | 6.82E-79 | 5.5E-78 | peptidase |
| B9H01_09250 | -3.27 | 2.06E-84 | 1.79E-83 | uridine phosphorylase |
| B9H01_09815 | -3.28 | 4.94E-74 | 3.69E-73 | hypothetical protein SSU05_2042 |
| B9H01_00550 | -3.30 | 1E-173 | 2.6E-172 | 50S ribosomal protein L15 |
| B9H01_00450 | -3.31 | 1.5E-164 | 3.6E-163 | 50S ribosomal protein L4 |
| B9H01_07175 | -3.32 | 1.48E-20 | 3.98E-20 | ABC transporter permease |
| B9H01_06615 | -3.32 | 1.5E-148 | 2.9E-147 | surface antigen SP1 |
| B9H01_07085 | -3.33 | 1.7E-146 | 3.2E-145 | phosphoglucosamine mutase |
| B9H01_09765 | -3.33 | 1.4E-143 | 2.5E-142 | hypothetical protein SSUST1_1923 |
| B9H01_03185 | -3.35 | 9.5E-23 | 2.72E-22 | hypothetical protein NJAUSS_0698 |
| B9H01_07185 | -3.35 | 2.83E-22 | 7.97E-22 | beta-glucosidase/6-phospho-beta-glucosidase/beta- galactosidase |
| B9H01_08645 | -3.38 | 1.3E-145 | 2.5E-144 | acetyl-CoA carboxylase subunit beta |
| B9H01_08070 | -3.40 | 4.71E-08 | 8.67E-08 | type II secretory pathway |
| B9H01_00920 | -3.41 | 2.4E-157 | 5E-156 | endopeptidase |
| B9H01_10395 | -3.41 | 1.27E-45 | 5.78E-45 | MutT/NudX family protein () |
| B9H01_06690 | -3.42 | 9.11E-63 | 5.62E-62 | Tellurite resistance protein tehB |
| B9H01_09235 | -3.45 | 1.01E-98 | 1.05E-97 | hypothetical protein SSU05_1923 |
| B9H01_06485 | -3.51 | 3.89E-29 | 1.29E-28 | glyoxalase/bleomycin resistance protein/dioxygenase superfamily protein |
| B9H01_04900 | -3.53 | 1.11E-92 | 1.07E-91 | hypothetical protein SSU05_0995 |
| B9H01_02315 | -3.56 | 2.47E-53 | 1.32E-52 | hypothetical protein SSUSC84_0394 |
| B9H01_00590 | -3.56 | 3.84E-67 | 2.62E-66 | 50S ribosomal protein L17 |
| B9H01_10095 | -3.56 | 4.81E-65 | 3.12E-64 | hypothetical protein SSU05_2099 |
| B9H01_07455 | -3.57 | 8.1E-114 | 1E-112 | branched-chain amino acid ABC transporter ATPase |
| B9H01_06700 | -3.59 | 7.1E-165 | 1.7E-163 | SsrA-binding protein |
| B9H01_01410 | -3.62 | 4.77E-64 | 3.03E-63 | hypothetical protein SSU98_0262 |
| B9H01_08785 | -3.64 | 1.1E-142 | 2E-141 | excinuclease ABC subunit A |
| B9H01_07365 | -3.64 | 0.000366 | 0.000544 | hypothetical protein SSU05_1525 |
| B9H01_07600 | -3.65 | 2.88E-12 | 6.29E-12 | hypothetical protein SSU05_1575 |
| B9H01_06420 | -3.67 | 2.6E-142 | 4.5E-141 | glutathione S-transferase |
| B9H01_09360 | -3.67 | 1.84E-43 | 8.17E-43 | 3-isopropylmalate dehydrogenase |
| B9H01_10110 | -3.68 | 1.41E-84 | 1.23E-83 | cell wall anchor domain-containing protein |
| B9H01_09040 | -3.68 | 6.9E-126 | 9.9E-125 | amino acid ABC transporter ATP-binding protein |
| B9H01_04550 | -3.69 | 0.004862 | 0.006697 | Tn916 |
| B9H01_02010 | -3.69 | 7.4E-109 | 8.7E-108 | hypothetical protein SSU05_0403 |
| B9H01_07330 | -3.71 | 0.000907 | 0.00132 | hypothetical protein SSU05_1518 |
| B9H01_04070 | -3.79 | 2.71E-31 | 9.51E-31 | hypothetical protein SSU05_0831 |
| B9H01_09830 | -3.85 | 2.2E-152 | 4.4E-151 | 2 |
| B9H01_08500 | -3.92 | 4.09E-51 | 2.1E-50 | hypothetical protein TL13_1564 |
| B9H01_09825 | -3.92 | 3.5E-121 | 4.9E-120 | hippurate hydrolase-like protein |
| B9H01_08655 | -3.97 | 7.8E-162 | 1.8E-160 | (3R)-hydroxymyristoyl-ACP dehydratase |
| B9H01_04800 | -4.00 | 0.029651 | 0.037775 | hypothetical protein SAG1290 |
| B9H01_06290 | -4.02 | 3.16E-66 | 2.1E-65 | hypothetical protein SSU05_1303 |
| B9H01_06525 | -4.04 | 8.54E-67 | 5.76E-66 | OsmC-like protein |
| B9H01_04625 | -4.04 | 0.008886 | 0.011954 | ATPases with chaperone activity |
| B9H01_06585 | -4.08 | 3.77E-84 | 3.25E-83 | amino acid ABC transporter permease |
| B9H01_04505 | -4.09 | 0.022334 | 0.028726 | A Chain A |
| B9H01_02585 | -4.10 | 8.4E-114 | 1.1E-112 | hypothetical protein SSU05_0510 |
| B9H01_06430 | -4.11 | 5.2E-183 | 1.5E-181 | 50S ribosomal protein L1 |
| B9H01_09350 | -4.11 | 1.28E-16 | 3.16E-16 | isopropylmalate isomerase small subunit |
| B9H01_03265 | -4.15 | 1.33E-89 | 1.21E-88 | 1-acyl-sn-glycerol-3-phosphate acyltransferase |
| B9H01_03560 | -4.16 | 1.3E-102 | 1.4E-101 | dihydrodipicolinate synthase |
| B9H01_06865 | -4.20 | 0.017005 | 0.022197 | hypothetical protein SSU05_1198 |
| B9H01_04595 | -4.21 | 0.01737 | 0.022629 | Tn916 hypothetical protein |
| B9H01_03780 | -4.22 | 2.7E-191 | 8.6E-190 | branched-chain amino acid aminotransferase |
| B9H01_08620 | -4.26 | 1.22E-51 | 6.34E-51 | hypothetical protein SSU05_1792 |
| B9H01_09355 | -4.26 | 1.07E-89 | 9.79E-89 | 3-isopropylmalate dehydratase large subunit |
| B9H01_00685 | -4.27 | 0.003796 | 0.005276 | hypothetical protein SSU05_0101 |
| B9H01_07325 | -4.28 | 0.016393 | 0.021454 | phage membrane protein |
| B9H01_09180 | -4.29 | 0.003242 | 0.004524 | transposase |
| B9H01_07650 | -4.38 | 1.45E-94 | 1.45E-93 | hypothetical protein SSU05_1585 |
| B9H01_02160 | -4.39 | 2.5E-294 | 1.4E-292 | cysteine synthase |
| B9H01_08640 | -4.40 | 1E-171 | 2.5E-170 | acetyl-CoA carboxylase subunit alpha |
| B9H01_08540 | -4.40 | 2.55E-45 | 1.15E-44 | metal ABC transporter periplasmic protein/surface antigen |
| B9H01_03175 | -4.42 | 5.43E-61 | 3.27E-60 | hypothetical protein SSUSC84_0565 |
| B9H01_03555 | -4.44 | 9.4E-122 | 1.3E-120 | aspartate-semialdehyde dehydrogenase |
| B9H01_03945 | -4.51 | 2E-144 | 3.6E-143 | homoserine kinase |
| B9H01_01730 | -4.52 | 3E-181 | 8.2E-180 | 50S ribosomal protein L28 |
| B9H01_03845 | -4.53 | 9.8E-302 | 5.6E-300 | 50S ribosomal protein L27 |
| B9H01_04515 | -4.56 | 0.001311 | 0.001887 | Tn916 hypothetical protein |
| B9H01_05580 | -4.64 | 0.000045 | 7.13E-05 | hypothetical protein SSU05_1149 |
| B9H01_03800 | -4.68 | 1.2E-210 | 4.2E-209 | 30S ribosomal protein S1 |
| B9H01_00195 | -4.68 | 2.77E-14 | 6.37E-14 | hypothetical protein SSUSC84_0018 |
| B9H01_08810 | -4.69 | 3.7E-129 | 5.4E-128 | single-stranded DNA-binding protein |
| B9H01_07345 | -4.74 | 0.002922 | 0.004089 | hypothetical protein SSU05_1521 |
| B9H01_06865 | -4.75 | 0.004572 | 0.00632 | hypothetical protein SSU05_1198 |
| B9H01_02515 | -4.75 | 0.004174 | 0.005778 | hypothetical protein SSU05_0498 |
| B9H01_04005 | -4.76 | 1.7E-135 | 2.8E-134 | guanosine 5&apos;-monophosphate oxidoreductase |
| B9H01_04850 | -4.80 | 6E-214 | 2.3E-212 | 50S ribosomal protein L7/L12 |
| B9H01_07475 | -4.88 | 1.3E-130 | 1.9E-129 | hypothetical protein SSU05_1548 |
| B9H01_08035 | -4.90 | 1.8E-158 | 3.9E-157 | hypothetical protein SSU05_1664 |
| B9H01_09345 | -4.95 | 4.89E-64 | 3.09E-63 | translation factor (SUA5) |
| B9H01_03190 | -4.95 | 2.4E-26 | 7.53E-26 | hypothetical protein SSU05_0636 |
| B9H01_04580 | -5.06 | 9.8E-06 | 1.61E-05 | hypothetical protein SPCG_1322 |
| B9H01_08820 | -5.08 | 0.000826 | 0.001205 | hypothetical protein SSU05_1198 |
| B9H01_09770 | -5.09 | 8.7E-214 | 3.3E-212 | hypothetical protein SSU05_2032 |
| B9H01_04600 | -5.09 | 0.000961 | 0.001396 | hypothetical protein |
| B9H01_10365 | -5.09 | 6.7E-18 | 1.69E-17 | hypothetical protein SSU05_2157 |
| B9H01_06480 | -5.15 | 3.37E-73 | 2.5E-72 | hypothetical protein SSU98_1358 |
| B9H01_08545 | -5.16 | 3.5E-288 | 1.8E-286 | glutamine amidotransferase |
| B9H01_02015 | -5.20 | 2.3E-159 | 5.1E-158 | hypothetical protein SSU05_0404 |
| B9H01_07355 | -5.36 | 0.000478 | 0.000708 | hypothetical protein SSU05_1523 |
| B9H01_07170 | -5.39 | 4.28E-75 | 3.25E-74 | ABC transporter ATP-binding protein |
| B9H01_02525 | -5.44 | 0.000276 | 0.000414 | hypothetical protein SSU05_0500 |
| B9H01_08525 | -5.48 | 6.3E-83 | 5.39E-82 | metal ion ABC transporter permease |
| B9H01_07370 | -5.72 | 5.69E-05 | 8.95E-05 | transcriptional regulator |
| B9H01_04545 | -5.74 | 2.15E-07 | 3.82E-07 | Tn916 hypothetical protein |
| B9H01_00830 | -6.26 | 5.86E-06 | 9.77E-06 | hypothetical protein SSU05_0132 |
| B9H01_09935 | -6.69 | 8.1E-266 | 3.9E-264 | amino acid ABC transporter permease |
| B9H01_03180 | -6.71 | 1.1E-167 | 2.8E-166 | hypothetical protein SSUSC84_0566 |
| B9H01_08815 | -7.95 | 5.4E-45 | 2.44E-44 | 30S ribosomal protein S6 |
| B9H01_08805 | -9.81 | 2.48E-22 | 7E-22 | 30S ribosomal protein S18 |
| B9H01_08740 | -14.13 | 8.13E-33 | 2.95E-32 | ribonucleases G and E |

Intersection of Upregulated Genes in ΔPrlP-Δ8740 compared to ΔPrlP and Downregulated Genes in ΔPrlP compared to WT

| Name | log2FoldChange | | pvalue | padj | Functional annotation |
| --- | --- | --- | --- | --- | --- |
|  | ΔPrlP-Δ8740vsΔPrlP | ΔPrlP vsWT |  |  |  |
| B9H01_00270 | 2.33 | -1.05 | 3.91E-36 | 1.5E-35 | 5-(carboxyamino)imidazole ribonucleotide synthase |
| B9H01_01610 | 3.29 | -5.03 | 5.07E-108 | 5.88E-107 | adhesion protein |
| B9H01_01980 | 2.45 | -1.58 | 1.8E-58 | 1.02E-57 | transcriptional regulator |
| B9H01_02125 | 2.02 | -1.03 | 3.21E-63 | 2E-62 | Ser/Thr phosphatase |
| B9H01_02130 | 2.12 | -1.03 | 2.43E-71 | 1.75E-70 | serine/threonine protein kinase |
| B9H01_02175 | 4.21 | -3.99 | 1.44E-15 | 3.45E-15 | amidophosphoribosyltransferase |
| B9H01_02955 | 3.35 | -2.28 | 1.61E-35 | 6.08E-35 | hypothetical protein |
| B9H01_03080 | 2.23 | -1.24 | 1.23E-40 | 5.12E-40 | hypothetical protein |
| B9H01_03085 | 2.09 | -1.05 | 1.96E-60 | 1.17E-59 | hypothetical protein |
| B9H01_04165 | 3.56 | -1.07 | 2.07E-66 | 1.39E-65 | N-acetyltransferase |
| B9H01_04180 | 3.82 | -1.29 | 9.5E-143 | 1.68E-141 | hypothetical protein |
| B9H01_04190 | 2.15 | -1.32 | 2.48E-38 | 9.95E-38 | translation initiation factor 1 |
| B9H01_04425 | 2.25 | -1.79 | 7.61E-27 | 2.41E-26 | site-specific integrase |
| B9H01_04605 | 2.13 | -1.06 | 1.78E-58 | 1.02E-57 | DNA helicase |
| B9H01_05060 | 4.05 | -1.43 | 3.83E-110 | 4.6E-109 | hypothetical protein |
| B9H01_05200 | 4.34 | -1.25 | 4.08E-77 | 3.2E-76 | adenylate cyclase |
| B9H01_05225 | 3.72 | -1.06 | 1.9E-115 | 2.44E-114 | redox-sensing transcriptional repressor Rex |
| B9H01_05300 | 4.37 | -1.12 | 1.61E-182 | 4.51E-181 | type I pantothenate kinase |
| B9H01_05335 | 4.01 | -3.32 | 1.76E-184 | 5.09E-183 | ciaR |
| B9H01_05340 | 3.76 | -4.57 | 1.34E-153 | 2.7E-152 | hypothetical protein |
| B9H01_05565 | 3.64 | -5.06 | 2.04E-61 | 1.24E-60 | DUF4956 domain-containing protein |
| B9H01_06240 | 3.92 | -1.4 | 4.71E-162 | 1.09E-160 | alkaline phosphatase family protein |
| B9H01_07250 | 5.14 | -1.25 | 3.84E-163 | 9.01E-162 | hypothetical protein |
| B9H01_07805 | 3.07 | -1.18 | 2.85E-59 | 1.64E-58 | sugar translocase |
| B9H01_08175 | 2.64 | -1.47 | 2.18E-38 | 8.76E-38 | phosphatase PAP2 family protein |
| B9H01_08860 | 6.16 | -5.06 | 2.08E-305 | 1.23E-303 | lipoprotein |
| B9H01_09855 | 3.35 | -2.74 | 4.98E-46 | 2.28E-45 | hypothetical protein |
| B9H01_10030 | 2.98 | -1.4 | 8.45E-152 | 1.66E-150 | MarR family transcriptional regulator |
| B9H01_10225 | 3.57 | -1.03 | 8.43E-120 | 1.16E-118 | arginine repressor |
| B9H01_10470 | 2.25 | -1.79 | 3.37E-28 | 1.1E-27 | hypothetical protein |
| B9H01_10530 | 2.75 | -0.79 | 4.72E-118 | 6.19E-117 | serine protease |
| B9H01_10535 | 3.83 | -2.84 | 7.18E-105 | 7.83E-104 | chromosome partitioning protein |

Intersection of Downregulated Genes in ΔPrlP-Δ8740 compared to ΔPrlP and Upregulated Genes in ΔPrlP compared to WT

| Name | log2FoldChange | | pvalue | padj | Fuction annotation |
| --- | --- | --- | --- | --- | --- |
|  | ΔPrlP-Δ8740vsΔPrlP | ΔPrlP vsWT |  |  |  |
| B9H01_00190 | -2.63 | 2.25 | 4.92E-22 | 1.37E-21 | rod shape-determining protein MreC |
| B9H01_00195 | -4.68 | 2.57 | 2.77E-14 | 6.37E-14 | rod shape-determining protein |
| B9H01_00355 | -2.41 | 2.07 | 4.65E-73 | 3.43E-72 | transposase |
| B9H01_00805 | -2.41 | 3.42 | 9.39E-06 | 1.54E-05 | competence protein CglB |
| B9H01_00830 | -6.26 | 4.43 | 5.86E-06 | 9.77E-06 | hypothetical protein |
| B9H01_00920 | -3.41 | 29.39 | 2.4E-157 | 5E-156 | endopeptidase |
| B9H01_01430 | -3.16 | 8.53 | 1.2E-157 | 2.6E-156 | surface-anchored protein |
| B9H01_01435 | -2.33 | 17.25 | 1.33E-38 | 5.39E-38 | methyl-accepting chemotaxis protein |
| B9H01_01440 | -3.04 | 17.73 | 3.5E-161 | 7.9E-160 | LPXTG cell wall anchor domain-containing |
| B9H01_02515 | -4.75 | 3.14 | 0.004174 | 0.005778 | hypothetical protein |
| B9H01_02520 | -2.68 | 6.26 | 0.058811 | 0.072543 | signal peptidase I |
| B9H01_02525 | -5.44 | 4.79 | 0.000276 | 0.000414 | hypothetical protein |
| B9H01_02530 | -3.01 | 3.86 | 0.241487 | 0.274393 | transposase |
| B9H01_03170 | -3.20 | 7.27 | 1.42E-42 | 6.22E-42 | glucosamine-6-phosphate deaminase |
| B9H01_03175 | -6.71 | 16.17 | 5.43E-61 | 3.27E-60 | hypothetical protein |
| B9H01_03180 | -6.71 | 14.21 | 1.1E-167 | 2.8E-166 | hypothetical protein |
| B9H01_03185 | -3.35 | 16.43 | 9.5E-23 | 2.72E-22 | hypothetical protein |
| B9H01_03190 | -4.95 | 19.05 | 2.4E-26 | 7.53E-26 | hypothetical protein |
| B9H01_03575 | -2.95 | 5.97 | 5.65E-06 | 9.42E-06 | glycerol kinase |
| B9H01_03930 | -2.21 | 2.02 | 7.49E-55 | 4.1E-54 | multidrug ABC transporter ATP-binding protein |
| B9H01_04360 | -3.03 | 4.36 | 2.13E-66 | 1.42E-65 | ABC transporter ATP-binding protein |
| B9H01_04365 | -2.17 | 6.93 | 9.54E-12 | 2.06E-11 | ABC transporter permease |
| B9H01_04375 | -2.58 | 6.67 | 2.26E-28 | 7.4E-28 | bacteriocin |
| B9H01_04380 | -3.02 | 7.85 | 1.26E-11 | 2.7E-11 | lipoprotein |
| B9H01_04515 | -4.56 | 6.04 | 0.001311 | 0.001887 | hypothetical protein |
| B9H01_04545 | -5.74 | 6.35 | 2.15E-07 | 3.82E-07 | conjugal transfer protein |
| B9H01_04550 | -3.69 | 3.04 | 0.004862 | 0.006697 | bifunctional lysozyme/C40 family peptidase |
| B9H01_04555 | -2.17 | 4.08 | 0.000119 | 0.000182 | membrane protein |
| B9H01_04560 | -2.01 | 4.62 | 7.26E-06 | 0.000012 | ATP/GTP-binding protein |
| B9H01_04580 | -5.06 | 4.49 | 9.8E-06 | 1.61E-05 | MobT family relaxase |
| B9H01_04590 | -2.76 | 4.15 | 0.000105 | 0.000161 | cell division protein FtsK |
| B9H01_04595 | -4.21 | 5.10 | 0.01737 | 0.022629 | hypothetical protein |
| B9H01_04600 | -5.09 | 3.05 | 0.000961 | 0.001396 | hypothetical protein |
| B9H01_04625 | -4.04 | 2.82 | 0.008886 | 0.011954 | hypothetical protein |
| B9H01_04640 | -2.45 | 3.78 | 8.5E-07 | 1.47E-06 | DNA primase |
| B9H01_04780 | -2.47 | 3.80 | 9.77E-12 | 2.1E-11 | amidase |
| B9H01_04785 | -2.30 | 4.05 | 4.63E-07 | 8.08E-07 | ATPase AAA |
| B9H01_04805 | -2.27 | 4.95 | 3.07E-06 | 5.21E-06 | conjugal transfer protein TraG |
| B9H01_04840 | -2.51 | 5.02 | 0.000144 | 0.00022 | hypothetical protein |
| B9H01_05125 | -2.36 | 2.73 | 0.005295 | 0.007283 | hypothetical protein |
| B9H01_07685 | -2.41 | 4.63 | 0.358372 | 0.395405 | IS200/IS605 family transposase |
| B9H01_06020 | -2.34 | 2.61 | 6.45E-05 | 0.000101 | competence protein CoiA |
| B9H01_06060 | -2.67 | 2.94 | 1.1E-22 | 3.14E-22 | ABC transporter ATP-binding protein |
| B9H01_06585 | -4.08 | 3.24 | 3.77E-84 | 3.25E-83 | glutamine ABC transporter permease |
| B9H01_06615 | -3.32 | 8.32 | 1.5E-148 | 2.9E-147 | SP1 surface-anchored protein |
| B9H01_07170 | -5.39 | 2.79 | 4.28E-75 | 3.25E-74 | ABC transporter ATP-binding protein |
| B9H01_07175 | -3.32 | 2.73 | 1.48E-20 | 3.98E-20 | ABC transporter permease |
| B9H01_07180 | -2.01 | 3.14 | 4.74E-09 | 9.14E-09 | ABC transport system membrane interstitial protein |
| B9H01_07185 | -3.35 | 3.17 | 2.83E-22 | 7.97E-22 | 6-phospho-beta-glucosidase |
| B9H01_07190 | -2.53 | 3.22 | 1.2E-20 | 3.22E-20 | PTS beta-glucoside transporter subunit EIIBCA |
| B9H01_07195 | -2.15 | 3.20 | 4.28E-05 | 6.79E-05 | transcription antiterminator BglG |
| B9H01_07310 | -2.15 | 2.04 | 0.000171 | 0.000259 | primase |
| B9H01_07330 | -3.71 | 4.26 | 0.000907 | 0.00132 | hypothetical protein |
| B9H01_07335 | -3.20 | 3.29 | 0.00228 | 0.003222 | hypothetical protein |
| B9H01_07340 | -3.07 | 3.42 | 0.00028 | 0.00042 | hypothetical protein |
| B9H01_07355 | -5.36 | 3.22 | 0.000478 | 0.000708 | hypothetical protein |
| B9H01_07365 | -3.64 | 4.16 | 0.000366 | 0.000544 | hypothetical protein |
| B9H01_07370 | -5.72 | 2.69 | 5.69E-05 | 8.95E-05 | transcriptional regulator |
| B9H01_07475 | -4.88 | 2.11 | 1.3E-130 | 1.9E-129 | branched-chain amino acid ABC transporter substrate-binding protein |
| B9H01_07600 | -3.65 | 2.52 | 2.88E-12 | 6.29E-12 | membrane protein |
| B9H01_07950 | -3.01 | 7.15 | 9.86E-05 | 0.000152 | IS110 family transposase |
| B9H01_08010 | -2.53 | 10.47 | 2.2E-07 | 3.92E-07 | isoprenylcysteine carboxyl methyltransferase |
| B9H01_08035 | -4.90 | 19.16 | 1.8E-158 | 3.9E-157 | hypothetical protein |
| B9H01_08070 | -3.40 | 8.39 | 4.71E-08 | 8.67E-08 | prepilin peptidase |
| B9H01_08530 | -2.60 | 2.05 | 1.3E-24 | 3.89E-24 | methionine ABC transporter ATP-binding protein |
| B9H01_08540 | -4.40 | 2.89 | 2.55E-45 | 1.15E-44 | lipoprotein |
| B9H01_08665 | -2.35 | 1.98 | 7.81E-55 | 4.26E-54 | beta-ketoacyl-[acyl-carrier-protein] synthase II |
| B9H01_08740 | -14.13 | 24.19 | 8.13E-33 | 2.95E-32 | ribonuclease |
| B9H01_09195 | -2.62 | 3.51 | 2.04E-05 | 3.29E-05 | sugar ABC transporter permease |
| B9H01_09895 | -2.18 | 2.50 | 0.007821 | 0.010614 | L-xylulose 5-phosphate 3-epimerase |
| B9H01_09905 | -2.54 | 3.33 | 0.011947 | 0.01582 | PTS ascorbate transporter subunit IIA |
| B9H01_09915 | -2.51 | 6.23 | 6.43E-06 | 1.06E-05 | PTS ascorbate transporter subunit IIC |
| B9H01_10095 | -3.56 | 8.80 | 4.81E-65 | 3.12E-64 | hypothetical protein |
| B9H01_10110 | -3.68 | 14.55 | 1.41E-84 | 1.23E-83 | cell wall anchor |
| B9H01_10115 | -3.19 | 14.48 | 8.53E-77 | 6.64E-76 | hypothetical protein |
