# Supplementary material for: The conserved transcription factor PrlP modulates colonization and pathogenicity of Streptococcus suis in response to environmental stress
Source: PLoS Pathog. 2025 Jul 18;21(7):e1013314. doi: 10.1371/journal.ppat.1013314 (PMC12273997; doi:10.1371/journal.ppat.1013314)
Supplement: S6 Table — (DOCX) [file ppat.1013314.s007.docx]

**Table S6.** Strains and plasmids used in this study.

| **Name** | **Characteristics** | **Source** |
| --- | --- | --- |
| **Strains** | | |
| SC19 | Isolated from a diseased pig in Sichuan, China | Lab stocks |
| Δ*prlP* | Isogenic *plrP* mutant of SC19 | This study |
| *prlP*-ΔN | Isogenic *plrP*-N domain mutant of SC19 | This study |
| *prlP*-ΔC | Isogenic *plrP*-C domain mutant of SC19 | This study |
| C(Δ*prlP)* | Complemented strain of SC19 Δ*prlP* | This study |
| C(*prlP*-ΔN) | Complemented strain of SC19 *plrP*-ΔN domain | This study |
| ENO- *prlP*-3×Flag | The strain of Δp*rlP* overexpressing *prlP* with 3×Flag | This study |
| Δ*prlP*-Δ8740 | Isogenic *B9H01_08740* mutant of Δ*prlP* | This study |
| Δ*prlP*-Δ0920 | Isogenic *B9H01_00920* mutant of Δ*prlP* | This study |
| Δ*prlP*-Δ3190 | Isogenic *B9H01_03190* mutant of Δ*prlP* | This study |
| Δ*prlP*-Δ5145 | Isogenic *B9H01_05145* mutant of Δ*prlP* | This study |
| *prlP*-ΔC ***_self-cleavage_*** | Isogenic *prlP*-ΔC ***_self-cleavage_*** domain mutant of SC19 | This study |
| *E. coli*DH5α | Cloing host for maintaining the recombinant plasmids | Lab stocks |
| *E. coli* BL21 | The expression host of recombinant proteins | Lab stocks |
| **Plasmids** | | |
| pSET4s | thermosensitive suicide vector; Spc^R^ | Lab stocks |
| pSET2 | *E. coli*–*S. suis* shuttle vector; Spc^R^ | Lab stocks |
| pET28a | Prokaryotic expression | Lab stocks |
| pSET4s-*PrlP* | Recombinant vector designed to knock out PrlP, Spc^R^ | This study |
| pSET4s-*PrlP*-N | Recombinant vector designed to knock out *PrlP*-N domain, Spc^R^ | This study |
| pSET4s-*PrlP*-C | Recombinant vector designed to knock out *PrlP*-N domain, Spc^R^ | This study |
| pSET4s-8740 | Recombinant vector designed to knock out B9H01_08740, Spc^R^ | This study |
| pSET4s-0920 | Recombinant vector designed to knock out B9H01_00920, Spc^R^ | This study |
| pSET4s-3190 | Recombinant vector designed to knock out B9H01_03190, Spc^R^ | This study |
| pSET4s-5145 | Recombinant vector designed to knock out B9H01_05145, Spc^R^ | This study |
| pET28a-*PrlP*-His8 | Cloning expression of recombinant *PrlP* | This study |
| pSET2-*PrlP* | pSET2 containing *PrlP*, Spc^R^ | This study |
| pSET2-ENO-*PrlP*-3×Flag | pSET2 containing *PrlP-*3×Flag, Spc^R^ | This study |
| pSET2-*PrlP*-N | pSET2 containing *PrlP*-N, Spc^R^ | This study |
