# Supplementary material for: The conserved transcription factor PrlP modulates colonization and pathogenicity of Streptococcus suis in response to environmental stress
Source: PLoS Pathog. 2025 Jul 18;21(7):e1013314. doi: 10.1371/journal.ppat.1013314 (PMC12273997; doi:10.1371/journal.ppat.1013314)
Supplement: S7 Table — (DOCX) [file ppat.1013314.s008.docx]

**Table S7.** **Primers used in this study**

| **Primers** | **Sequence (5′–3′)** |
| --- | --- |
| **Mutation and complementary primers** | |
| pSET4S-F | GGATCCTCTAGAGTCGACCTG |
| pSET4S-R | GGGTACCGAGCTCGAATTCAC |
| *PrlP*-L-F | TCGAGCTCGGTACCCTACTATTAGCCATCTTTCTC |
| *PrlP*-L-R | GGAGACAATCTTTTTAAGCCTAGTTAATCACT |
| *PrlP*-R-F | GGCTTAAAAAGATTGTCTCCTTATCTGTTTAAC |
| *PrlP*-R-R | GACTCTAGAGGATCCAAGATACAAATAACTTCGGT |
| *PrlP*-DN-L-F | TCGAGCTCGGTACCCCAAACGATAAGATACAAATAAC |
| *PrlP*-DN-L-R | ATCCTGATTCATGATTGTCTCCTTATCTGTT |
| *PrlP*-DN-R-F | ACAATCATGAATCAGGATATAGCTTTAACGA |
| *PrlP*-DN-R-R | GACTCTAGAGGATCCCCAATAGGTCGAATATCGTCTT |
| *PrlP*-DC-L-F | TCGAGCTCGGTACCCTAATCCTTTTCATTTGCGACTC |
| *PrlP*-DC-L-R | ATCTGGACTGATTCGCTCCCGATAGTCATCC |
| *PrlP*-DC-R-F | GAGCGAATCAGTCCAGATTGATTTTTAAGCC |
| *PrlP*-DC-R-R | GACTCTAGAGGATCCATACTATTAGCCATCTTTCTC |
| 8740-L-F | CAAGCTTGCATGCCTGCAGGTCGACTCTAGAGGATCCCATGACCTCTTGGTCTTCATTG |
| 8740-L-R | GTACTTATATGGAGGACTTTATATTAACACAGGTTAAAGATGACG |
| 8740-R-F | AAGTCCTCCATATAAGTACTTCTG |
| 8740-R-R | AAAACGACGGCCAGTGAATTCGAGCTCGGTACCCGTATCAATGAATGGTAATGGATTTG |
| 0920-L-F | CTCTAGAGGATCCCAGTTATCCAATTGATGGTTGA |
| 0920-L-R | GGTTTCTGTCCTTTCTTTTTATTG |
| 0920-R-F | GAAAGGACAGAAACCAAGATTATAGAGGTGTCTGCGGTC |
| 0920-R-R | CTCGGTACCCGATAACACCGATCTTATCAGATAC |
| 3190-L-F | GAGGATCCTGTGCTATACTGTTACTACGTTTC |
| 3190-L-R | GAAATGTCTCCTTTTACAAAGTATTAG |
| 3190-R-F | GGAGACATTTCGGTTTCTATTTTTCGTTGACTTC |
| 3190-R-R | CGGTACCCCAACTCTCGATCGAACTGCTCCA |
| 5145-L-F | CTAGAGGATCCCCAATCATGGCCACTAGTCAAGAG |
| 5145-L-R | CCGATACAGTTGTAGGAAAACATC |
| 5145-R-F | CAACTGTATCGGTTTCATTCTCCTACTATAATTAC |
| 5145-R-R | GGTACCCGAACGCCATCTGAATTGCTGAAAGGAG |
| pSET2-F | GGGTACCGAGCTCGAATTCAC |
| pSET2-R | GGATCCTCTAGAGTCGACCTG |
| C(*PrlP*)-L-F | TCGAGCTCGGTACCCGTGCTTTTCAATTTGTTTTC |
| C(*PrlP*)-L-R | GACTCTAGAGGATCCTTAGCATAGGTTTCCCACT |
| C(*PrlP*-N)-L-F | TCGAGCTCGGTACCCTTAGCATAGGTTTCCCACTC |
| C(*PrlP*-N)-L-R1 | CTTATCGTCGTCATCCTTGTAATCGGAATCTAGTTGCTCGATTAG |
| C(*PrlP*-N)-L-R2 | GACTCTAGAGGATCCTCACTTATCGTCGTCATCCT |
| PrlP-C*_self-cleavage_*-DN-PPE-F | GGGGAATTTGGTGATTTTTAAGCCTAGTTAATCAC |
| PrlP-C*_self-cleavage_*-DN-R | CTGAATTGCTGAAAGGAGTTC |
| PrlP- C*_self-cleavage_* -UP-F | CATTTGCGACTCCTATTTTTC |
| PrlP-C*_self-cleavage_*-UP-PPE-R | CTGGCGAAACAGGCAGAAACCTTACCAGG |
| PPE-F | TGTTTCGCCAGAGGCTTTCTG |
| PPE-R | CCAAATTCCCCGTAGGCGCTA |
| Protein expression primers | |
| *PrlP*-Flag-F | GCTCGGTACCCTCACTTATCATCATCATCCTTGTAATCGATATCGTGATCCTTGTAATCGCCATCGTGATCCTTGTAATCATCTGGAC |
| *PrlP*-R | ATTTTAAAAGAATACCGAAGCAAG |
| *PrlP*-F | ATCTGGACTATAAATATCTAC |
| ENO-F | TATTCTTTTAAAATCATTATATTACTCTCCTTTGAGTTTAAAATTGTTAC |
| ENO-R | CAGGTCGACTCTAGAGGATCCCCTGTTTCGCCAGAGGCTTTCTG |
| pET28a-F | GGTATATCTCCTTCTTAAAG |
| pET28a-R | CACCACCACCACCACCACTGAGAT |
| *PrlP*-His8-F1 | CTTTAAGAAGGAGATATACCATGATTTTAGGTGATATTTTAAAAG |
| *PrlP*-His8-R | GTGGTGGTGGTGGTGGTGGTGGTGATCTGGACTATAAATATCTACAAC |
| qRT-PCR Primers | |
| RT-7600-F | GACAGCACCTTGAAAGAAGA |
| RT-7600-R | CCAAGAGAGCAGAGGGAA |
| RT-0535-F | ACGTACACTAAATATTTCAGAAATC |
| RT-0535-R | TCGCAACCTTCTTTCTCCAG |
| RT-5335-F | TAGACTTTCCTTGGCTGTTG |
| RT-5335-R | TTTCTTAGATGATTTTGCGG |
| RT-5225-F | TGTAGGTGTTGGGAATATGG |
| RT-5225-R | TGAGAGTTTGCTTCGCTGAT |
| RT-2130-F | CCAAGAGAGCAGAGGGAA |
| RT-2130-R | TTCCCTCTGCTCTCTTGG |
| RT-8740-F | GCTGATGCTTATTCCCTGT |
| RT-8740-R | GGCTTCCTGCTTATGTTGT |
| RT- *PrlP*-F | CTCAGGTTGTGGGAGATTCTATGGC |
| RT- *PrlP*-R | AGGTCGAATATCGTCTTCTGGGCTA |
| RT-16S-F | GATAGGGTTTCTCTTCGGAGC |
| RT-16S-R | CATAAGGGGCATGATGATTTG |
| RT-GAPDH-F | ATGGCGTAGATATCGTTTTG |
| RT-GAPDH-R | AAGGATGTCGTGGTTAGTGT |
|  | EMSA primers |
| *PrlP*-motif-30-F | TCGGCAGAATTAGTTTCCGGTGAAGTTTGA |
| *PrlP*-motif-30-R | TCAAACTTCACCGGAAACTAATTCTGCCGA |
| *PrlP*-motif-30-F-FAM | FAM’-TCGGCAGAATTAGTTTCCGGTGAAGTTTGA |
| 0951-motif-non-F | TCGGCAGAATTcccccaaaaTGAAGTTTGA |
| *PrlP*- moti-non-R | TCAAACTTCAttttgggggAATTCTGCCGA |
